# Photolysis of CO_2_ Carbamate for Hydrocarboxylation Reactions

**DOI:** 10.1021/jacs.5c21208

**Published:** 2026-01-12

**Authors:** Emanuele Azzi, Manuel Rodríguez-Martínez, Sai Rohini Narayanan Kolusu, Jacopo Scarfiello, Jesus A. Varela, Manuel Nappi

**Affiliations:** Centro Singular de Investigación en Química Biolóxica e Materiais Moleculares (CiQUS), Departamento de Química Orgánica, 16780 Universidade de Santiago de Compostela, Rúa de Jenaro de la Fuente, s/n, 15705 Santiago de Compostela, A Coruña, Spain

## Abstract

The conversion of carbon dioxide into value-added products has emerged as an alternative method to achieve net-zero emissions. While technologies that transform CO_2_ into fuels and chemical feedstocks have made great strides, the direct use of CO_2_ as a C1 synthon for the formation of new carbon–carbon bonds remains a critical challenge. Herein, we present a new catalytic CO_2_ activation mode for hydrocarboxylation reactions. Key to this methodology is the formation of a CO_2_ carbamate with a phenothiazine catalyst, which sets the required trigonal geometry for the release of CO_2_^•–^ via photolysis upon absorption of visible light. The polarity-reversed CO_2_^•–^ is employed in the hydrocarboxylation reactions of alkenes and heterocycles. This protocol is distinguished by its mild reaction conditions, wide substrate scope and broad applicability, even in the context of pharmaceutical cores. Our chemistry can also be utilized for the synthesis of carbon-13 labeled spirolactones using ^13^CO_2_. Mechanistic experiments support the photolysis of the CO_2_ carbamate as the main productive pathway under our optimized reaction conditions.

## Graphical abstract

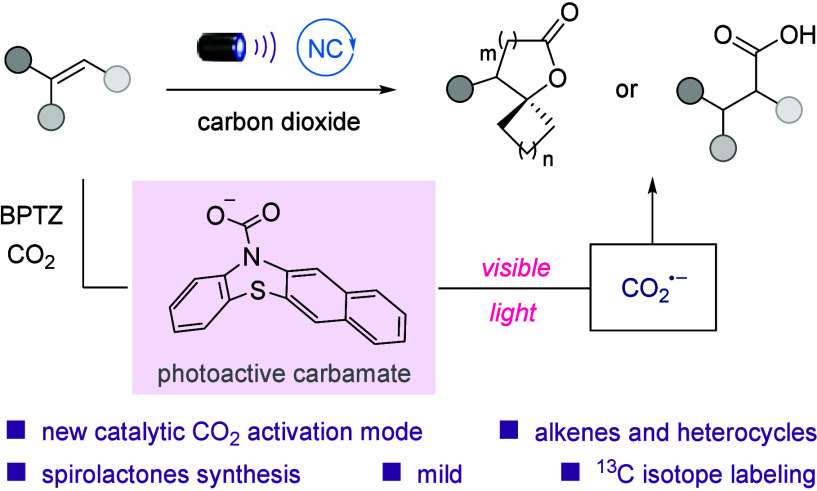

## Introduction

The direct application of CO_2_ as a C1 synthon for creating new carbon–carbon bonds continues to pose a significant challenge in synthetic chemistry.^1–3^ Although CO_2_ is considered an ideal feedstock because of its wide availability, the relative stability of this linear small molecule has stimulated chemists to design various activation strategies to exploit its synthetic potential. Traditionally, these methods required the use of strong nucleophiles such as organometallic reagents or transition-metal catalysts (Figure 1A).^4–6^ Lately, the single electron reduction of carbon dioxide to generate the CO_2_ radical anion (CO_2_^•–^) has attracted considerable attention as an alternative approach to access new reactivity. However, this is a thermodynamically and kinetically demanding process, due to the extremely low reduction potential of CO_2_ (*E*_1/2_ = −2.2 V vs SCE)^7^ and the high reorganization energy required to accommodate the new trigonal geometry of CO_2_^•–^ during the electron transfer.^8^ Given the high energetic requirement, researchers first utilized an electrochemical setup to achieve the single electron reduction of carbon dioxide. While various works reported the successful activation of CO_2_, high negative overpotentials and currents were crucial to overcome the slow kinetics and efficiently generate the CO_2_^•–^.^9–11^ Recently, several methods have emerged for the photochemical single-electron reduction of the CO_2_. High-energy UV light,^12,13^ stoichiometric photoreductants,^14^ iridium or heterogeneous photocatalysts,^15–17^ often in combination with stoichiometric bases and additives, were essential to enable the formation of CO_2_^•–^, arguably due to the high kinetic barrier caused by the required change in geometry. In a significant effort, Maiti and Audisio recently reported an elegant procedure to obtain CO_2_^•–^ from CO_2_ via in situ generation of formate, merging photoredox catalysis with stoichiometric hydride and hydrogen atom transfer (Figure 1A).^18^

**Figure 1:**
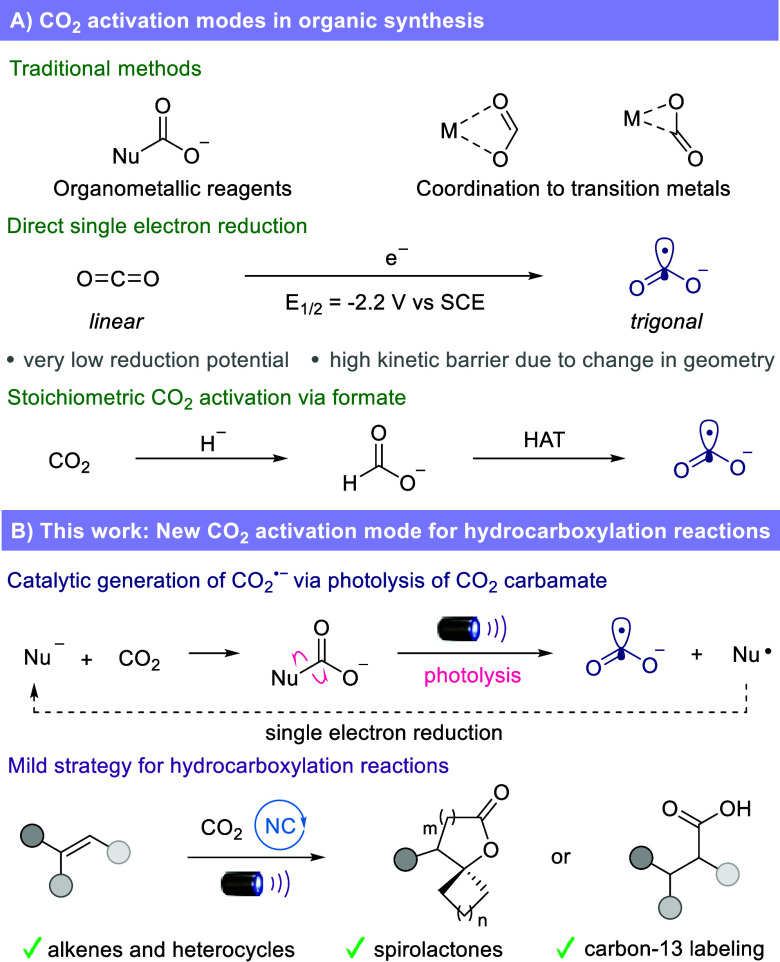
CO_2_ as a feedstock in carboxylation reactions. (A) CO_2_ activation modes in organic synthesis. (B) This work: New CO_2_ activation mode for hydrocarboxylation reactions.

Herein, we report a new CO_2_ activation mode for hydrocarboxylation reactions (Figure 1B). Exploiting the classical polar reactivity of carbon dioxide with nucleophiles, we designed a catalytic system in which a photoactive carbamate is formed in situ between CO_2_ and a phenothiazine catalyst. Upon irradiation with visible light, this transient carbamate is photolyzed to release CO_2_^•–^, which is then employed in hydrocarboxylation reactions. A variety of alkenes and heterocycles react smoothly with CO_2_ to afford the corresponding carboxylic acids. Feedstock and complex cyclic ketones can be readily converted with a two-step process into valuable spirocyclic structures in good yields, even in the context of pharmaceutical cores. Finally, this new method was utilized to synthesize ^13^C-labeled lactones using ^13^CO_2_, showcasing the potential for isotopic labeling applications.

## Design plan

From the outset of our investigation, we recognized that the formation of a carbamate intermediate through the covalent interaction between CO_2_ and a N-centered nucleophile would already set the trigonal geometry for the potential generation of CO_2_^•–^, avoiding the high kinetic barrier due to the geometry transition in the case of direct electron transfer reduction.

We hypothesized that if a proper photoactive molecule is employed as the nucleophile, then the carbamate could undergo photolysis to liberate the CO_2_^•–^, along with the radical species of the nucleophile. However, given the propensity of CO_2_^•–^ to form undesired products such as oxalate and multicarboxylated species, the concentration of this transient carbamate must be kept low for its application in synthetic reactions. Therefore, we proposed the use of a photoactive nucleophilic catalyst, which would comprise the following features: 1) easily obtainable in the reaction media from a stable precursor; 2) exhibits absorption in the visible region; 3) able to perform catalytic redox cycles and 4) deliver a persistent radical after the photolysis process, providing a driving force for the homolytic fragmentation. We identified benzophenothiazine (BPTZ) anion **1** as a suitable photoactive nucleophile for our novel activation mode toward the generation of CO_2_^•–^ (Figure 2). Previous work showed the ability of phenothiazine derivatives to absorb visible light and participate in redox catalytic cycles.^19–21^ We anticipated that in a saturated atmosphere of carbon dioxide, a carbamate intermediate is initially obtained between BPTZ anion **1** and CO_2_. It is reasonable to assume that the absorption properties of carbamate **2** would be similar to those of neutral BPTZ. Therefore, upon absorption of visible light, excited carbamate **3** undergoes photolysis to generate CO_2_^•–^ along with open-shell species **4**, which is known to be a persistent radical.^22,23^ The BPTZ anion **1** is then restored via a single electron transfer by an appropriate reductant.

**Figure 2:**
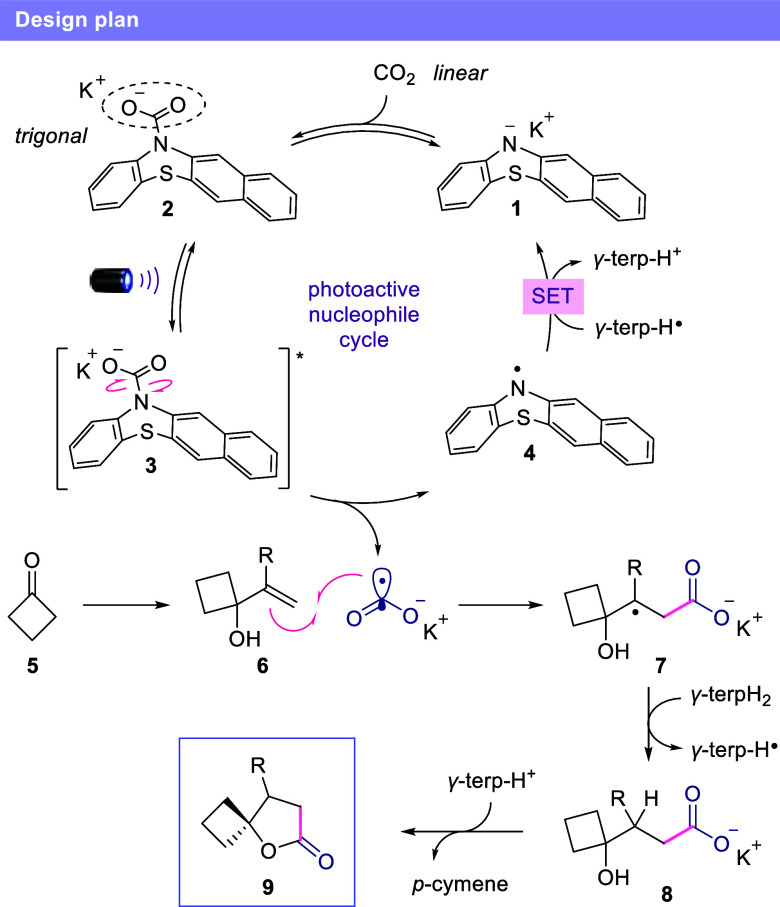
Design plan.

To demonstrate this conceptually distinct activation mode as a generic platform for installation of CO_2_ into organic molecules, we have initially selected to perform a carboxylation reaction of hydroxyalkenes **6** for the direct synthesis of *γ-* and *δ-*spirolactones. Spirolactones, especially the *γ-* and *δ-* analogues, have attracted considerable interest in scientific research due to their diverse biological activities and potential therapeutic applications.^24–26^ Several synthetic methods are available for the construction of the *γ-*spirolactones: (i) esterification, (ii) Baeyer−Villiger oxidation, (iii) radical cyclization of oxalates,^27,28^ (iv) alkenes reaction with carboxylic acid derivatives^29–31^ or CO_2_^•–^/carbonyl compounds,^32^ (v) alcohols/ketones addition-cyclization with α,β-unsaturated carbonyl compounds.^33,34^ On the contrary, only a few protocols allow the spirolactonization in the case of the *δ-*spirocyclic structures: (i) esterification, (ii) Baeyer–Villiger oxidation, and (iii) organocatalytic cyclization of isatins with in situ generated conjugated enolates.^35^ Despite the considerable number of synthetic reactions, the lactone carbonyl group is always preinstalled in the starting materials, normally by multistep synthesis and oxidation, except for limited examples of metal-catalyzed carbonylation using high pressure of toxic carbon monoxide.^36,37^ Therefore, the development of a general unified strategy for the synthesis of *γ-* and *δ-*spirolactones using simple starting materials such as cyclic ketones and CO_2_ is highly desirable yet elusive.

We sought to address this synthetic challenge with a novel catalytic activation strategy. Initially, cyclic ketone **5** is converted to the corresponding hydroxyalkene **6** via a high-yielding Grignard or Barbier reaction, depending on whether the final molecule is a *γ-* and *δ-*spirolactone (Figure 2). The addition of highly reactive CO_2_^•–^ to alkene **6** in an anti-Markovnikov fashion furnishes carbon-centered radical intermediate **7**. The hydroxycarboxylate **8** is then obtained via hydrogen atom transfer (HAT) with *γ-*terpinene, followed by an intramolecular esterification to provide the desired spirolactone **9**. Simultaneously, the persistent radical **4** (*E*_1/2_ = 0.18 V vs SCE, please see Supporting Information (SI) for details) is reduced by the γ-terpinene radical (*E*_1/2_ = −0.1 V vs SCE),^38^ restoring the benzophenothiazine anion **1**.

## Results and Discussion

Initially, we performed a series of experiments to verify the feasibility of our new activation mode. We conducted UV–vis absorption and emission studies to characterize and compare the spectroscopic properties of benzophenothiazine (BPTZ) **10**, the corresponding anion **1** and CO_2_ carbamate **2** (Figure 3A and 3B). When an excess of potassium *tert*-butoxide was added to a solution of benzophenothiazine **10**, we observed a change in color from pale green to wine red, indicating the formation of the benzophenothiazine anion **1**. As expected, when CO_2_ was bubbled through solution containing anion **1**, the wine-red color changed to bright green, showing a relatively similar yet different absorption spectra to that of the neutral benzophenothiazine **10**, revealing the formation of carbamate **2**. Once certain of the ability of carbamate **2** to absorb visible light, we exposed presynthesized carbamate **11** to 390 nm LED light in the presence of *γ-*terpinene and DMF under argon atmosphere (Figure 3C). After 16 h, we were pleased to observe the formation of formate in 30% yield, supporting the generation of CO_2_^•–^. Formate is the result of only one of the possible CO_2_^•–^ termination pathways and therefore does not represent the complete mass balance of CO_2_^•–^ formation. Importantly, when the same experiment was performed without light, no formate was detected. Next, we sought to gain additional evidence for the formation of CO_2_^•–^ without preforming the carbamate, but simply using a solution of benzophenothiazine **10**, potassium *tert*-butoxide, *γ-*terpinene and CO_2_ (Figure 3D). The reaction was monitored over time with an FT-IR spectrometer. After 10 s, the IR spectra clearly showed the characteristics bands of carbamate **2** (1661 and 1644 cm^–1^, see SI for details), confirming its formation in solution. Over the course of the reaction, carbamate **2** and CO_2_ (2360 cm^–1^) IR bands gradually disappeared in favor of a new band at 1610 cm^–1^, assigned to the CO_2_^•–^ or formate.^39–42^ The presence of formate was also confirmed by NMR studies at the end of the reaction (see SI for details). When the same experiment was repeated in the dark, the only detected species was carbamate **2**. Moreover, we were able to detect oxalate using FT-IR in the absence of γ-terpinene. Taken together, these preliminary experiments strongly suggest the formation of CO_2_^•–^ via photolysis of carbamate **2** under visible light irradiation.

**Figure 3:**
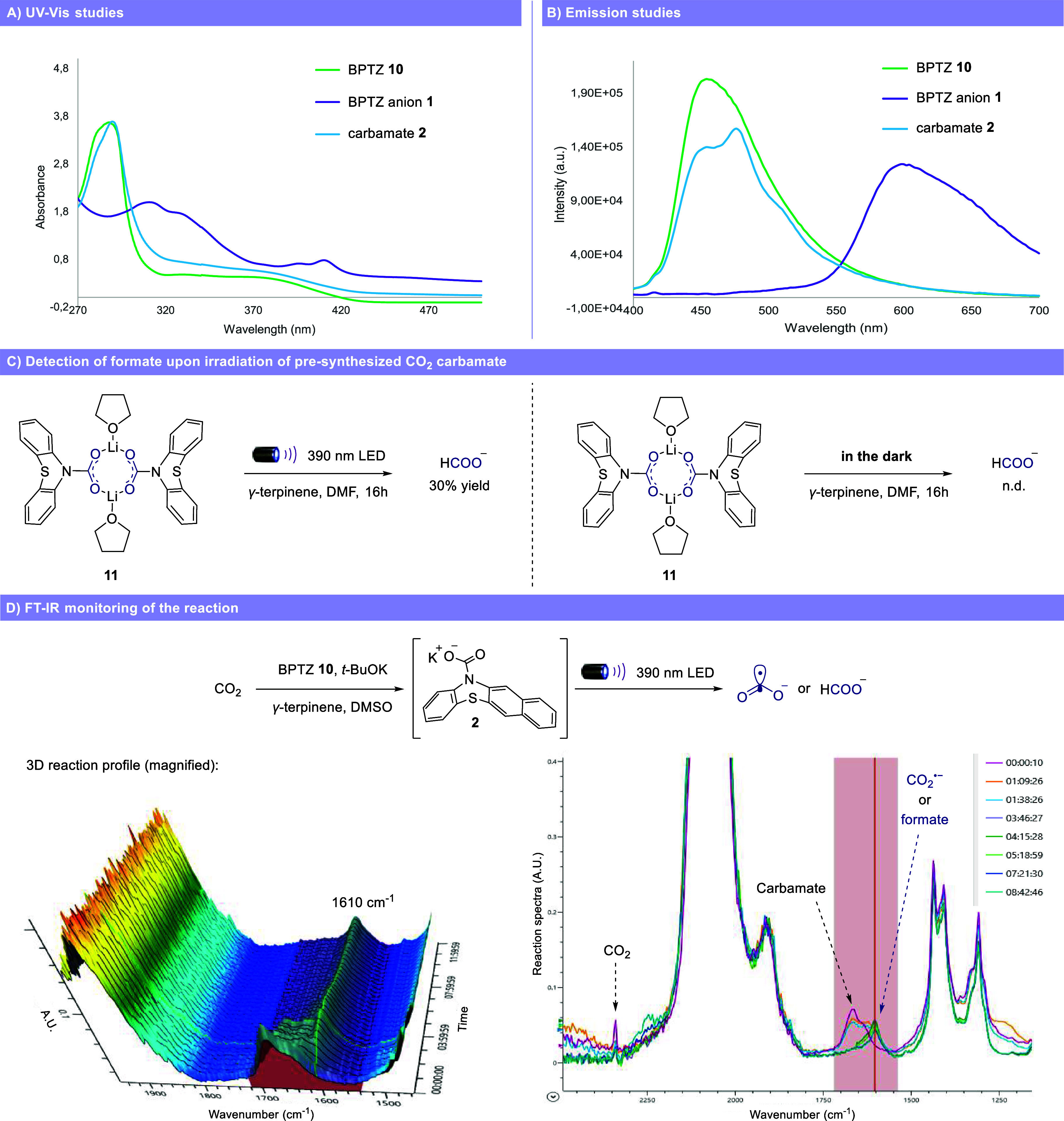
Preliminary experiments. (A) UV–vis studies of benzophenothiazine **10**, corresponding anion **1** and CO_2_ carbamate **2**. (B) Emission studies of benzophenothiazine **10**, corresponding anion **1** and CO_2_ carbamate **2**. (C) Detection of formate upon irradiation of presynthesized CO_2_ carbamate **11**. (D) FT-IR monitoring of the reaction. BPTZ = benzophenothiazine.

Having confirmed the generation of CO_2_^•–^ using benzophenothiazine **10** and carbon dioxide, we proceeded to study the catalytic anti-Markovnikov carboxylation of hydroxyalkene **12** (Table 1). To our delight, when a DMF solution of **12**, benzophenothiazine **10** (10 mol %), *t*-BuOK (20 mol %) and *γ-*terpinene was backfilled with 1 atm of CO_2_ and exposed to 390 nm LED light, the desired *γ-*spirolactone **13** was obtained in 75% isolated yield (entry 1). Catalyst, solvent, base, and HAT source were key parameters for the optimization studies. Variation on the phenothiazine core revealed the superiority of conjugated BPTZ **10** compared to analogues **14**–**16** in terms of yield (entries 2–4). Polar solvents such as DMSO and NMP showed the formation of the product in comparable yields with DMF, while in acetonitrile the *γ-*spirolactone was only detected in 37% yield (entries 5,6). *t*-BuOK was found to be the best base in terms of reaction outcome (entries 7, 8), while full conversion of the starting hydroxylalkene was achieved at 0.2 M concentration (please see SI for details). As expected, γ-terpinene and 1,4-cyclohexadiene were the only competent HAT sources under the reaction conditions, arguably because of their matched potential to reduce persistent radical **4** (entries 9, 10). Additional control reactions demonstrated that purple light and catalyst are fundamental for the observed reactivity (entries 11 and 12), consistent with the mechanistic blueprint outlined in Figure 2. The reaction without base provided the product in 38% yield (entry 13), probably due to the inefficient formation of the carbamate. Other well-established CO_2_ carboxylation methods have failed to furnish product **13** in yields higher than 30%, demonstrating the superiority of our protocol for the synthesis of spirolactones (please see SI for details).

**Table 1:**
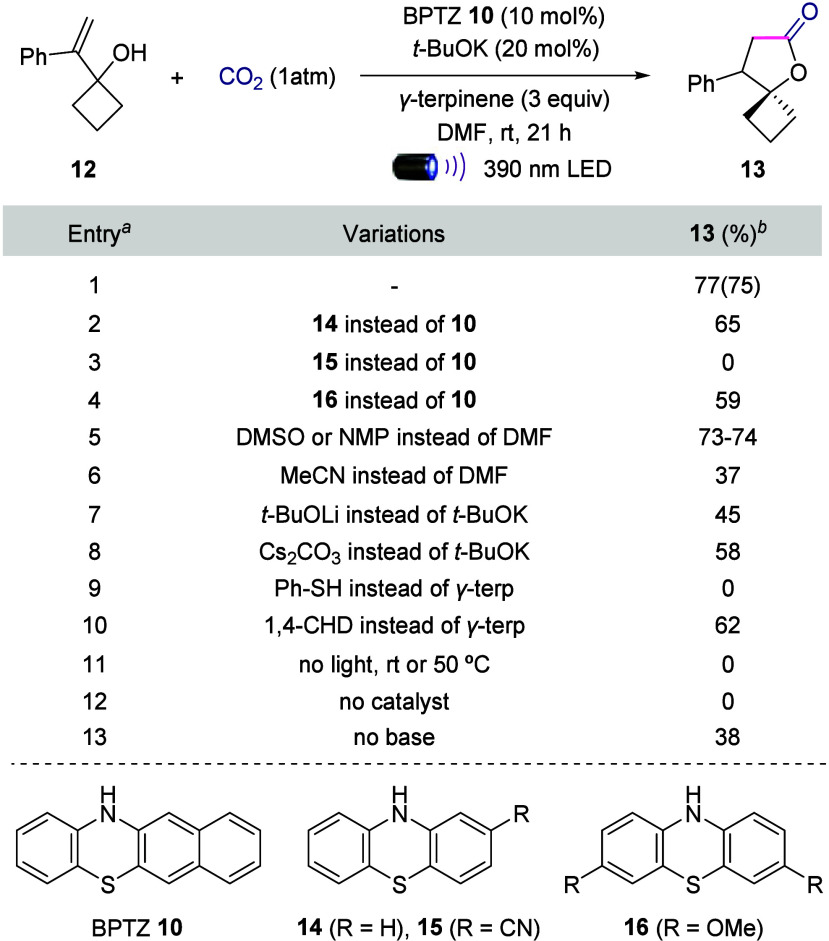
Optimization and Control Reactions^a^

a Reaction conditions: **12** (0.2 mmol), photocatalyst (10 mol %), base (20 mol %), HAT donor (0.6 mmol), in solvent (0.2 M) at rt for 21 h, 390 nm LED.

b NMR yields using methyl 3,5-dinitrobenzoate or 1,1,2,2-tetrachloroethane as internal standard. BPTZ = benzophenothiazine.

With optimal conditions in hand, we first examined the generality of our new activation mode in terms of radical carboxylation for the synthesis of *γ-* and *δ-*spirolactones. As is evident from the results compiled in Figure 4, our mild carboxylation via CO_2_^•–^ could be conducted on a wide variety of hydroxyalkenes derived from feedstock cyclic ketones. Starting ketones of different sizes (**13**, **17**–**20**) and heterocyclic analogues (**23**–**26**) were readily converted to the corresponding γ-spirolactones in moderate to good yields. Substituents on the starting cyclic ketone (**21**) and sterically hindered structures such as adamantanone (**22**) were also tolerated. Viable motifs in this transformation include *N*-Boc protected azetidinone (**23**), thiethanone (**24**) and tetrahydro­(thio)­pyranone (**25**, **26**) derivatives. Notably, the use of molecules already characterized by the presence of a spirocenter allows the synthesis of geometrically intricated structures bearing multiple spirocenters (**27**, **28**), thus elevating molecular three-dimensionality and complexity, key attributes known to enhance the potential of new structures for pharmaceutical applications.^43^ Both electron-rich and electron-poor substituents on the phenyl moiety posed no problems (**30**–**34**), including a heteroaromatic ring such as thiophene (**35**), providing the corresponding spirocyclic product in moderate yields. Interestingly, we also observed that the vinylsulfonylbenzene motif can function as a masked C2-synthon for the synthesis of an unsubstituted spirolactone. When this feature was installed on 3,3-diphenylcyclobutanone, the corresponding hydroxyalkene (**36**) smoothly underwent radical carboxylation and reductive desulfonylation, resulting in the formation of the unsubstituted γ-spirolactone (**37**) in 42% yield after only 3 h.

**Figure 4:**
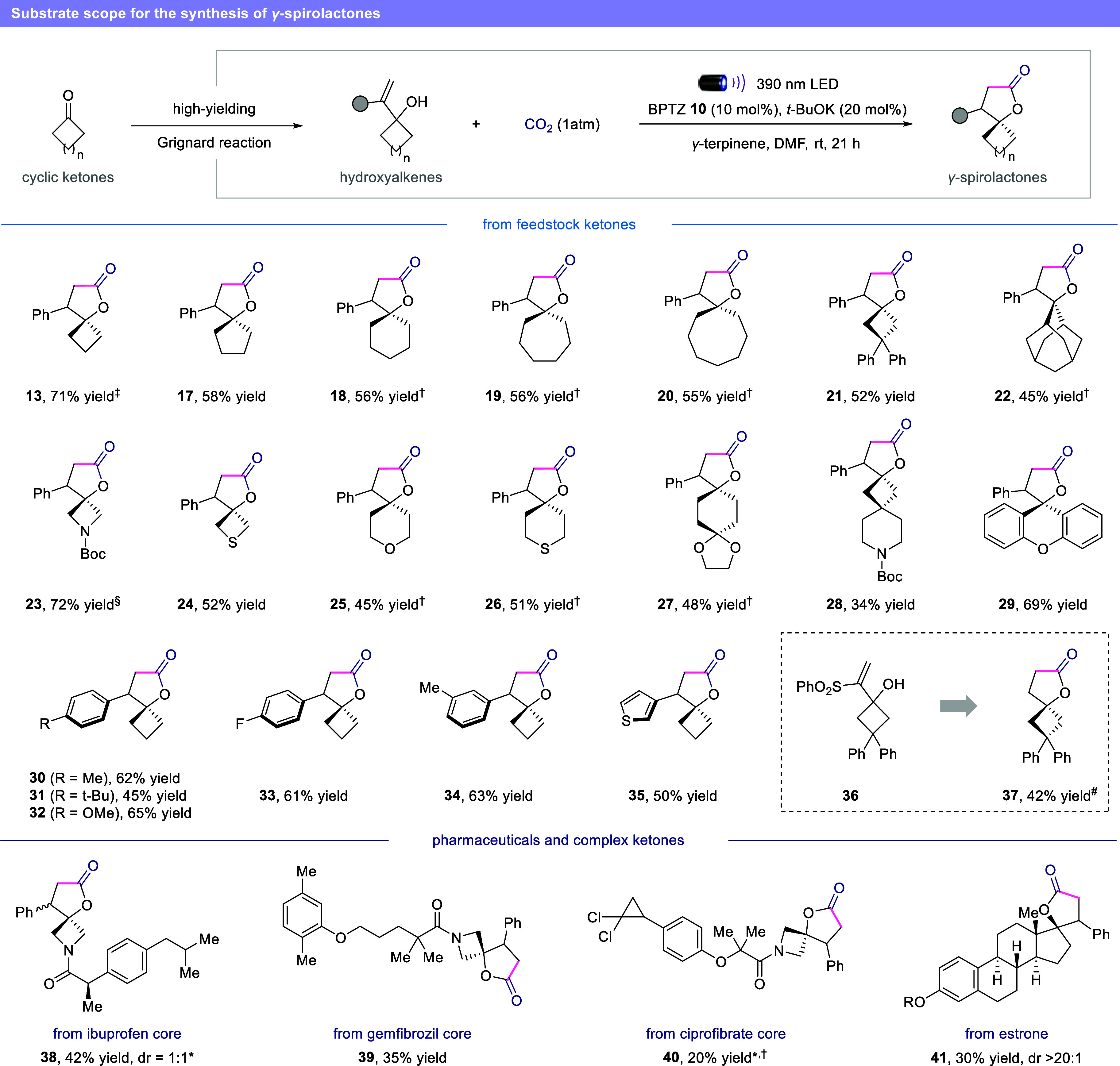
Synthesis of *γ*-spirolactones. Reaction conditions as in Table 1, entry 1, 0.2 mmol scale. Isolated yield unless otherwise noted. ^‡^5 mmol scale. ^†^2 lamps, 60 °C, 48 h. ^§^NMR yield using 1,1,2,2-tetrachloroethane as an internal standard. ^#^0.05 M, 3 h. *Isolated as mixture of diastereoisomers. BPTZ = benzophenothiazine.

Importantly, we were pleased to find that this method is also amenable to substrates containing pharmaceutical cores such as ibuprofen (**38**), gemfibrozil (**39**) and ciprofibrate (**40**). Our reaction could also be applied to the steroid derivative estrone (**41**), leading to the synthesis of a new potential candidate in the class of the antimineralocorticoid 17*α*-spirolactosteroids.^44,45^ The excellent diastereoselectivity observed using estrone is arguably under kinetic control due to the irreversibility of the diastereodetermining HAT process.

Remarkably, our protocol was found to be applicable to the more challenging synthesis of *δ-*spirolactones (Figure 5). To prevent polymerization and degradation of the starting material, a combination of higher dilution and catalytic amount of tetrabutylammonium bromide was necessary. Pleasingly, we were able to derivatize most of the cyclic ketones used in Figure 4 and convert them to the desired *δ-*spirolactones. Ring sizes up to 12 members (**42**–**47**), molecules bearing multiple spirocenters (**49**) and different heterocyclic structures (**53**–**55**) were obtained in moderate to good yields. The methodology showed good compatibility with a variety of moieties such as geminal difluoro substituents (**50**), ketone (**51**) and unactivated alkene (**52**). Our method could be successfully employed for the synthesis of fused lactone **58** and the derivatization of linear acyclic ketones (**59**–**61**), showing progressive improvements in yields with an increase in the alkyl chain length.

**Figure 5:**
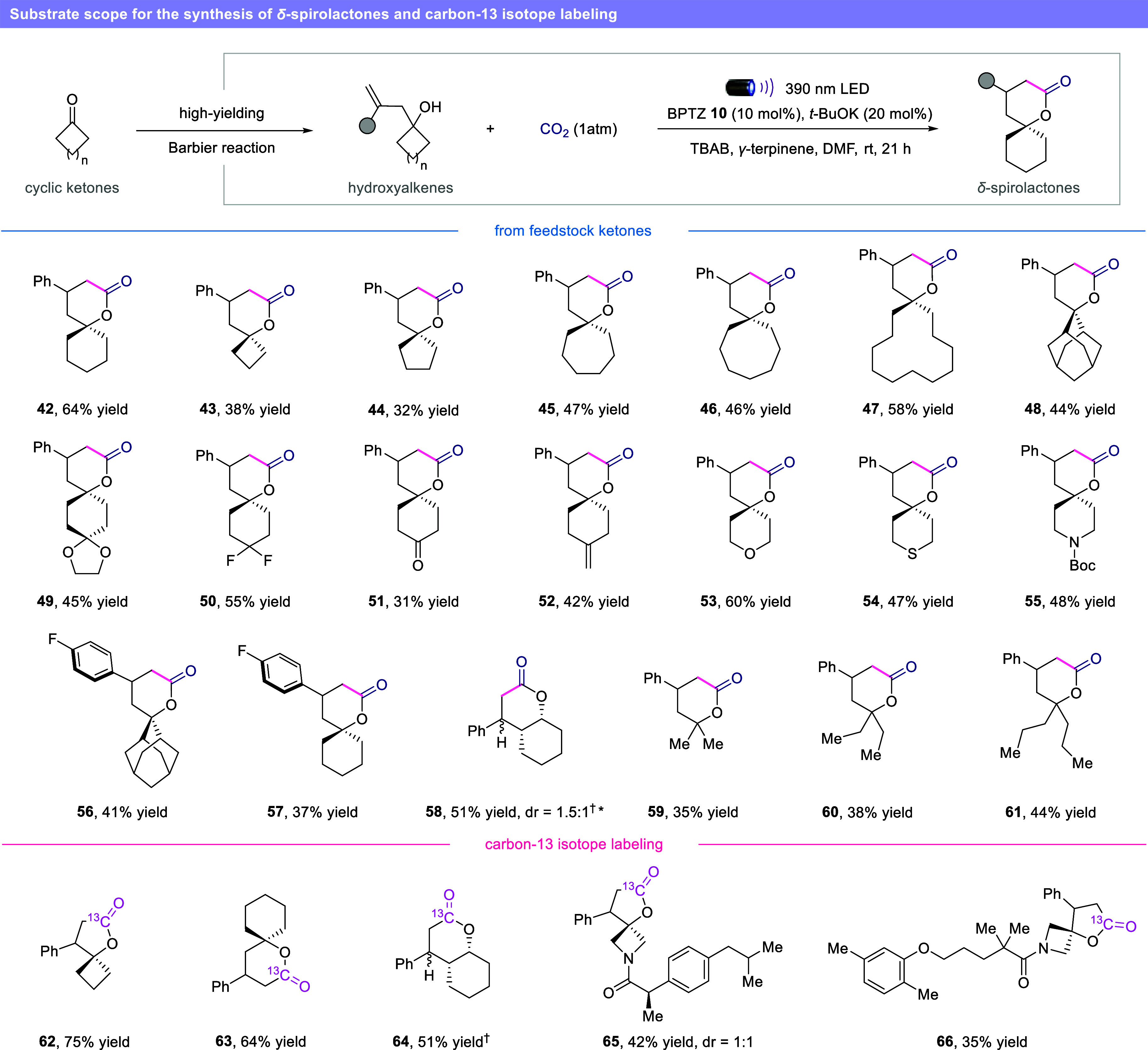
Synthesis of *δ*-spirolactones and carbon-13 labeling. Reaction conditions: hydroxyalkene (0.2 mmol), BPTZ **10** (10 mol %), *t*-BuOK (20 mol %), TBAB (30 mol %) *γ*-terpinene (0.6 mmol), in DMF (0.05 M) at rt for 21 h, 390 nm LED. Isolated yield unless otherwise noted. ^†^Reaction conditions are the same as Table 1, entry 1. *Isolated as mixture of diasteroisomers. BPTZ = benzophenothiazine. TBAB = tetrabutylammonium bromide.

Given the simplicity of our newly developed method, we set out to explore the possible utilization of ^13^CO_2_ for the synthesis of carbon-13 labeled spirolactones. In particular, the direct generation of labeled reactive species such as ^13^CO_2_^•–^ represents a powerful tool toward direct and versatile site-specific incorporation of ^13^CO_2_ into organic backbones, that only recently has shown its first applications with the synthesis of labeled carboxylic acids.^18,46–54^ The isolation of ^13^C-labeled organic compounds is indeed crucial for a precise tracking of the molecular transformations of a target molecule in fields such as fundamental biology, metabolomics, and hyperpolarized magnetic resonance imaging.^55–58^ Given the practical flexibility of the reaction setup, we anticipated that our new radical carboxylation protocol could be used for the streamline synthesis of ^13^C enriched spirolactones. Replacing the atmosphere of CO_2_ with ^13^CO_2_, we successfully provided the ^13^C-labeled products via direct generation of ^13^CO_2_^•–^, including *γ-* and *δ-*spirolactones (**62**, **63**), fused *δ-*lactone (**64**) and spirolactones derived of pharmaceutical cores (**65**, **66**).

Finally, we were delighted to see that our protocol is not limited to hydroalkenes, demonstrating the broad applicability of our CO_2_ activation mode (Figure 6). More conventional alkenes such as styrenes (**67**–**71**), acrylates (**74**–**76**) and acrylamides (**77**) effectively undergo hydrocarboxylation. Carboxylic acid and the fluoxetine core were also well tolerated, furnishing desired products **72** and **73** in synthetically useful yields.

**Figure 6:**
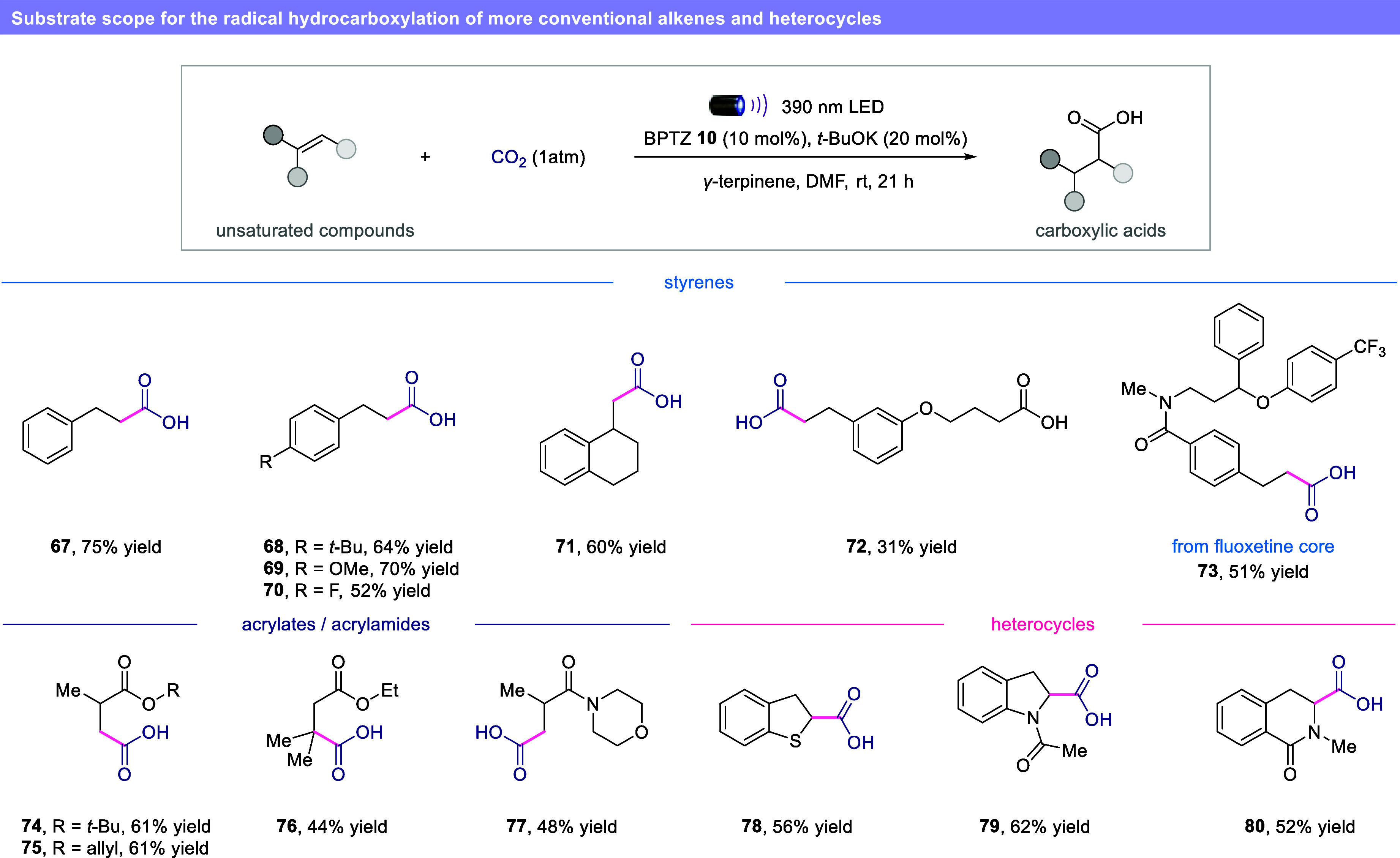
Substrate scope for the radical hydrocarboxylation of more conventional alkenes and heterocycles. Reaction conditions: hydroxyalkene (0.2 mmol), BPTZ **10** (10 mol %), *t*-BuOK (20 mol %), *γ*-terpinene (0.6 mmol), in DMF (0.2 M) at rt for 21 h, 390 nm LED. Isolated yield unless otherwise noted. BPTZ = benzophenothiazine.

Unfortunately, only traces of the product were observed when unactivated alkenes were used, likely due to the combination of slow and reversible addition of the CO_2_^•–^ to unactivated olefins and the polarity mismatch of the HAT process. However, electron-rich and electron-poor heterocycles could be readily converted to the corresponding semiunsaturated carboxylic acids (**78**–**80**) in good yields.

## Mechanistic Studies

While the preliminary studies described in Figure 3 strongly suggest that the formation of CO_2_^•–^ via photolysis of carbamate **2** is feasible, we conducted additional experiments to confirm that this process is indeed the main pathway for the generation of CO_2_^•–^ and the hydrocarboxylated product under our optimized reaction conditions.

Formate was detected both in the absence and in the presence of hydroalkene **12**, with TON of 5 and 35 respectively, supporting the catalytic generation of CO_2_^•–^ (Figure 7A, entries 1 and 2). Control experiments in the dark without CO_2_ or *γ-*terpinene did not show any formate. These results confirm that (i) formate is generated only as a consequence of the CO_2_^•–^ and (ii) *γ-*terpinene is not a hydride donor under our reaction conditions, excluding any possible formation of formate directly from CO_2_.^18^ To rule out the possibility of formate as productive intermediate in our hydrocarboxylation strategy, we performed a control experiment where 2 equivalents of sodium formate were used instead of CO_2_ (Figure 7B). As expected, we were unable to detect product **13**. The formation of product **82** in a radical clock experiment using the cyclopropyl styrene substrate **81** confirmed the radical nature of our method (Figure 7C).

**Figure 7:**
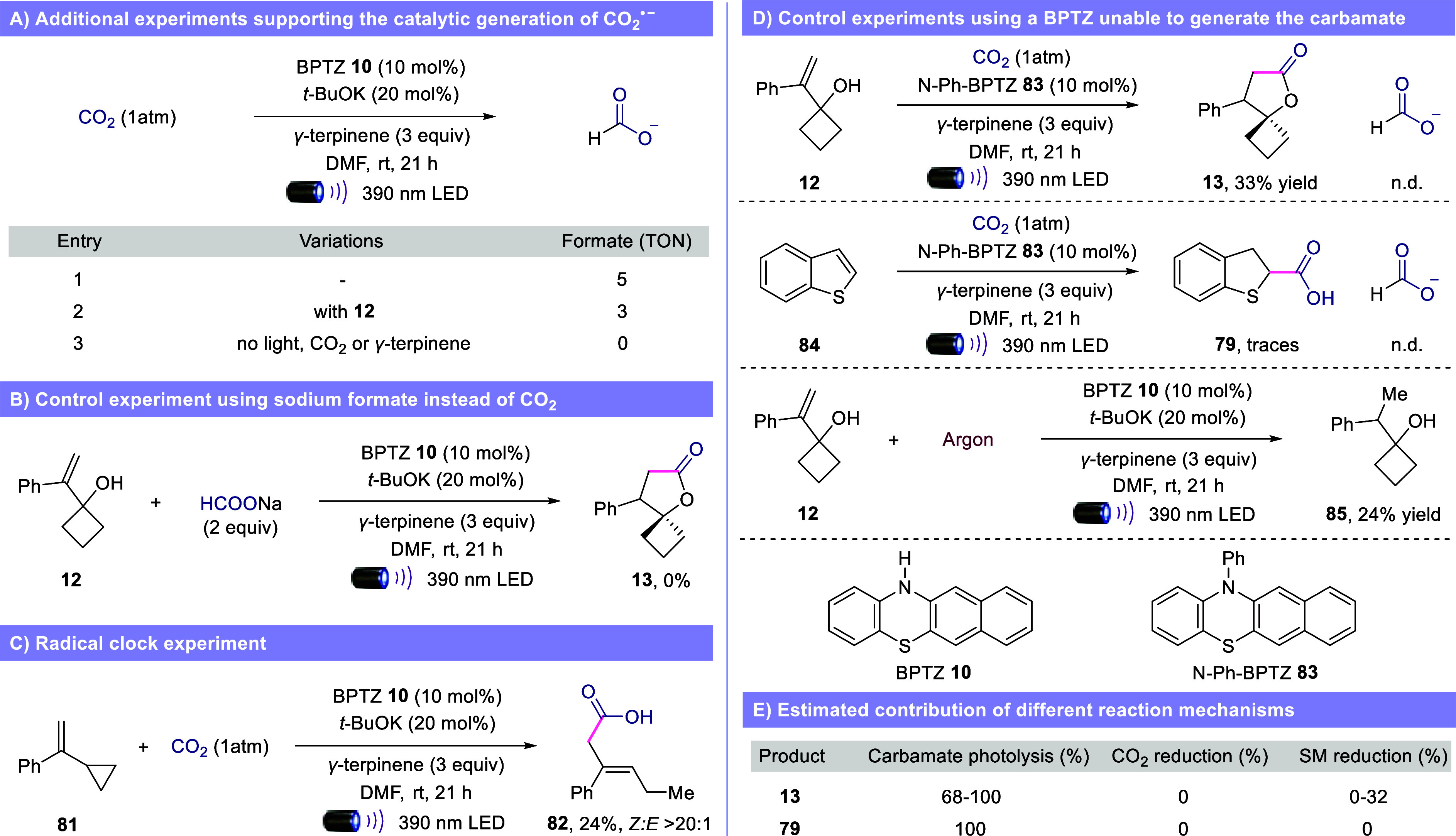
Mechanistic studies. (A) Additional experiments supporting the catalytic generation of CO_2_^•–^. (B) Control experiment using sodium formate instead of CO_2_. (C) Radical clock experiment. (D) Control experiments using a BPTZ unable to generate the carbamate. (E) Estimated contribution of different reaction mechanisms. BPTZ = benzophenothiazine. TON = turnover number.

Once it was established that CO_2_^•–^ is catalytically generated under our optimized reaction conditions, we conducted a series of control experiments to confirm that carbamate photolysis is the main pathway for the formation of the hydrocarboxylated products (Figure 7D). When N–H benzophenothiazine **10** was replaced with *N*-phenyl benzophenothiazine **83**, which is unable to generate the key photoactive carbamate **2**, we detected *γ-*spirolactone **13** in a low 33% yield. The same experiment was repeated using benzothiophene **84** as the starting material, where we could only detect traces of the corresponding hydrocarboxylated product **79**. While the second experiment clearly supports the crucial role of the carbamate and the proposed photolysis, the first experiment indicates that another minor mechanism is contributing to the formation of product **13**. Formate was not observed in either reaction, suggesting that the alternative mechanism for the formation of product **13** is not the direct reduction of CO_2_, but the reduction of styrene **12**.^59^ Notably, although styrene substrates having a lower reduction potential than CO_2_ (−2.58 V vs −2.2 V vs SCE respectively),^60^ the photocatalyst is only able to reduce **12**, confirming the prohibitive kinetic requirement to directly reduce CO_2_. When the optimized reaction was repeated under argon, we observed the formation of product **85** in only a 24% yield (Figure 7D).

Since product **85** is generated via direct reduction of substrate **12**, we can estimate that the maximum contribution of this pathway to the formation of spirolactone **13** is 32%, confirming that the photolysis of carbamate **2** is the major productive mechanism under our optimized reaction conditions (Figure 7E). Finally, a quantum yield of 0.07 was obtained using the ferrioxalate chemical actinometer, consistent with the proposed mechanism (see SI for details).

To further evaluate the kinetic feasibility of the proposed activation mode, we performed DFT calculations on the photoinduced formation of **4** and CO_2_^•–^K^+^ from potassium carbamate **2**, involving excitation from the ground state (S_0_) to the singlet excited state (S_1_), intersystem crossing to the triplet state (T_1_) and subsequent evolution along the T_1_ surface (Figure 8, please see SI for details). Formation of potassium carbamate **2** from the corresponding BPTZ anion **1** and CO_2_ was found to be almost barrierless and exergonic at the employed level of theory, affording four conformers (**2**_**A‑D**_) in fast equilibrium, depending on the position of the potassium cation. This energetic profile indicates that, under a CO_2_ atmosphere and steady-state photocatalytic conditions, the most stable conformer, carbamate **2**_**A**_, is the dominant ground-state resting species and therefore the main photoactive intermediate, while the free BPTZ anion **1** is likely present only transiently as it is rapidly intercepted by CO_2_. Upon light irradiation, carbamate **2**_**A**_ undergoes vertical excitation to the lowest singlet excited state (S_1_) which after vibrational relaxation yields the corresponding S_1_ minimum.

**Figure 8:**
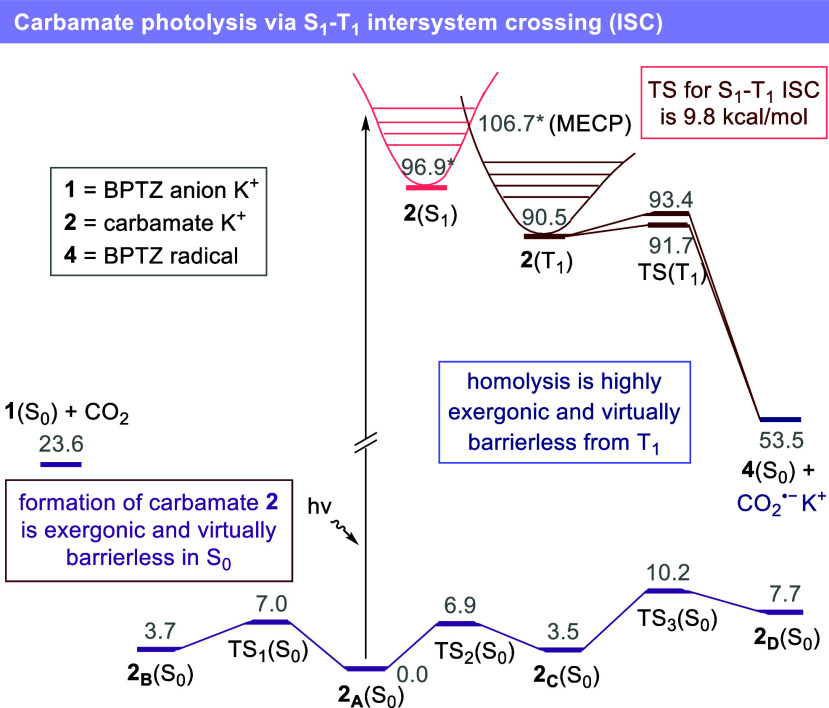
Computed free energy profile for the formation of potassium carbamate **2** in the ground state (S_0_, violet), photoinduced excitation to the singlet excited state (S_1_, pink), intersystem crossing to the triplet excited state (T_1_, red), and subsequent evolution to form **4** and CO_2_^•–^K^+^. Computational studies were performed at wB97X-D/6-31G­(SMD = DMF)// wB97X-D/6-31G theory level. All energies are DG_sol_ reported in kcal mol^–1^ relative to those of **2**_**A**_(S_0_). Energy values marked with an asterisk correspond to electronic energies (DE_sol_) reported in kcal mol^–1^ relative to **2**_**A**_(S_0_). BPTZ = benzophenothiazine. hν = photon. MECP = minimum energy crossing point. ISC = intersystem crossing.

A minimum energy crossing point (MECP) between the S_1_ and T_1_ potential energy surface was located 9.8 kcal mol^–1^ above the **2**(S_1_) minimum, demonstrating the feasibility of the S_1_–T_1_ intersystem crossing. Optimization of the potassium carbamate from the MECP geometry on the triplet potential energy surface afforded species **2**(T_1_), from which two nearly barrierless pathways were identified for homolytic cleavage of the C–N bond leading to **4**(S_0_) and CO_2_^•–^K^+^ (ΔG^‡^ = 2.9 and 1.2 kcal mol^–1^, respectively; ΔG^0^ = −37.0 kcal mol^–1^). These computational results, together with the experimental findings presented in Figure 3 and 7, support a mechanism in which the photolysis of carbamate **2** constitutes the main pathway for the hydrocarboxylation of unsaturated systems.

## Conclusions

In summary, we have developed a new catalytic CO_2_ activation mode for hydrocarboxylation reactions. The formation of photoactive CO_2_ carbamate, which set the required trigonal geometry, allows the generation of CO_2_^•–^ under mild reaction conditions. The protocol exhibits a wide functional group tolerance and broad substrate scope, even in the context of biologically active molecules. Furthermore, this technology was successfully applied to the carbon-13 isotope labeling of *γ-* and *δ-*spirolactones. The general principle of photolytic nucleophilic activation of electrophiles to generate radicals is expected to pave the way for the development of new synthetic reactions. Exploration of new avenues is currently underway in our laboratories.

## Supplementary Material



## Notes

General information; experimental procedures; characterization data for all new compounds; mechanistic experiments; DFT calculations; and relevant coordinates of optimized geometries. (PDF)
