# Supplementary material for: Photolysis of CO2 Carbamate for Hydrocarboxylation Reactions
Source: J Am Chem Soc. 2026 Jan 12;148(3):3801–10. doi: 10.1021/jacs.5c21208 (PMC12856901; doi:10.1021/jacs.5c21208)
Supplement: Supplementary file 1 [file ja5c21208_si_001.pdf]

Photolysis of CO_2 carbamate for hydrocarboxylation reactions

Emanuele Azzi,[1] Manuel Rodríguez-Martínez,[1] Sai Rohini Narayanan Kolusu,[1] Jacopo Scarfiello,[1] Jesus A. Varela[1] and Manuel Nappi[1,*]

Email: manuel.nappi@usc.es

1) Center for Research in Biological Chemistry and Molecular Materials (CiQUS), University of Santiago de Compostela, Rúa de Jenaro de la Fuente, s/n, Santiago de Compostela 15705, Spain

Supplementary Information

Table of Contents

1. General Information 3

2. Reaction optimization and control experiments 4

3. Synthesis of Starting Materials 10

4. Photochemical Reactions 37

5. Mechanistic Studies 68

  Cyclic Voltammetry Studies 68

  UV-Vis studies 69

  Emission studies 71

  Detection of formate upon irradiation of pre-synthetized CO_2 carbamate 71

  FT-IR monitoring of the reaction 76

  Additional experiments supporting the catalytic generation of $CO_2^{\bullet-}$ 79

  Control experiment using sodium formate instead of CO_2 84

  Radical Clock Experiment 84

  Control experiments using a BPTZ that is unable to generate the carbamate 86

  Quantum yield determination 87

  Comparison with previously reported β-hydrocarboxylation methodologies 89

6. DFT studies 91

7. NMR Traces 110

1. General Information

Proton nuclear magnetic resonance (^{1}H NMR) spectra were recorded at ambient temperature on Varian Mercury 300 MHz or Bruker AVIII 500 MHz spectrometers. Chemical shifts (δ) are reported in ppm and quoted to the nearest 0.01 ppm relative to the residual protons in $CDCl_3$ (7.26 ppm) and coupling constants (*J*) are quoted in Hertz (Hz). Data are reported as follows: Chemical shift (multiplicity, coupling constants, number of protons). Coupling constants were quoted to the nearest 0.1 Hz and multiplicity reported according to the following convention: s = singlet, d = doublet, t = triplet, q = quartet, qn = quintet, sp = septet, m = multiplet, br = broad. Where coincident coupling constants have been observed, the apparent (app) multiplicity of the proton resonance has been reported.

Carbon nuclear magnetic resonance (^{13}C NMR) spectra were recorded at ambient temperature on Varian Mercury 300 MHz or Bruker AVIII 500 MHz spectrometers. Chemical shift (δ) was measured in ppm and quoted to the nearest 0.1 ppm relative to the residual solvent peaks in $CDCl_3$ (77.16 ppm). DEPT135, NOE experiments and 2-dimensional experiments (COSY, HMBC and HSQC) were used to support assignments where appropriate.

High-resolution mass spectra (HRMS) were measured on Bruker microTOF spectrometer using direct injection APCI mode at the CACTUS facility of the University of Santiago de Compostela.

Analytical thin layer chromatography (TLC) was performed using pre-coated Merck glass backed silica gel plates (Silica gel 60 F254). Flash column chromatography was undertaken on silica gel (40-60 μm) under a positive pressure of air unless otherwise stated. Visualization was achieved using ultraviolet light (254 nm) and chemical staining with basic potassium permanganate solutions as appropriate.

Dichloromethane (DCM), Tetrahydrofuran (THF), dimethylformamide (DMF), Acetonitrile (MeCN) were dried and dispensed using solvent purification system. Dimethylsulfoxide (DMSO) was purchased from Sigma-Aldrich chemical company. All reagents were purchased at the highest commercial quality and used without further purification. Diethyl ether (Et_2O) used as an eluent in flash column containing 200ppm of 2,6-di-*tert*-butyl-4-methylphenol (BHT) as a stabilizer.

Reactions were carried out under an atmosphere of Argon unless otherwise stated. All reactions were monitored by TLC, ^{1}H NMR spectra taken from reaction samples (NMR yields determined by ^{1}H NMR with reference to 1,1,2,2-tetrachloroethane as an internal standard) or gas chromatography mass spectrometry (GC-MS) using Agilent 8890 (mass detector: Agilent 5977B GC/MSD).

Photochemical reactions were irradiated using Kessil PR160L 390 nm Purple Light Lamp (40 W) as light source (settings: 100% intensity). The estimated irradiance of the Kessil 390 nm LED is approximately 280 mW/cm^2 when placed 3 cm away from the reaction vessel. The temperature was kept constant (~ 20ºC) using a fan (50/60 Hz, 230 V, 2700/3200 r/min) placed on top of the reactions at 20 cm distance.

2. Reaction optimization and control experiments

In a 10 mL Schlenk tube equipped with a stir bar was added the ketone-derived tertiary alcohol (0.2 mmol, 1.0 equiv.), γ-terpinene (added with a 100 µL Hamilton syringe), the catalyst and the base (weighed on analytical balance, ± 0.1 mg tolerance). Subsequently 1 mL of the desired dry solvent was added.

The resulting mixture was subsequently degassed *via* freeze-pump-thaw method (3 cycles), backfilled with 1 atm CO_2 and irradiated with visible light. *The Schlenk tube was placed in a 3D-printed carousel, resulting in a final distance of 3 cm from the glass wall of the Schlenk vial to the Kessil lamp. The reaction temperature was kept constant using a fan placed on top of the reaction at 20 cm distance.*

Note about freeze-pump-thaw: To perform the freeze-pump-thaw and backfilling with CO_2, the CO_2 cylinder was connected to the Schlenk line by replacing the common Argon or Nitrogen line with a CO_2 line. For every cycle, the reaction mixture was frozen with liquid nitrogen and kept under vacuum leaving the stopcock of the Schlenk vial open for 4 minutes, Subsequently the stopcock was closed, the vial sealed and the mixture thawed with a 35°C water bath for 4 minutes. After the completion of three cycles, the Schlenk flask was sealed under vacuum, and the CO_2 gas valve on the Schlenk line was open. The stopcock of the Schlenk vial was opened to expose the reaction mixture to CO_2 while stirring, thus backfilling the Schlenk vial's atmosphere.

After 21 hours, EtOAc (3 mL) was added to the Schlenk vial and moved to a separating funnel. The Schlenk vial was rinsed with EtOAc (2x3 mL). Then this organic phase was washed three times with 2 M HCl water solution saturated with NaCl (5 mL). The organic phase was collected and separated, dried over $MgSO_4$, filtered and the solvents evaporated. Yield for optimization studies were determined from ^{1}H-NMR of the crude reaction mixtures using 1,1,2,2-tetrachloroethane and methyl 3,5-dinitrobenzoate as internal standards.

Supplementary Table. S-1 Evaluation of the Photocatalyst

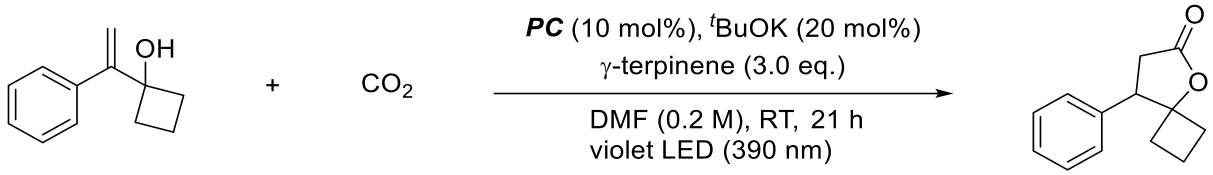

Entry	Photocatalyst (10 mol%)	Product (% yield)
1	PHT-H	70
2	(2-CF_3)PHT-H	0
3	(2-CN)PHT-H	47
4	$(OMe)_2$PHT-H	63
5	BPTZ **10**	77
6	N-Ph-BPTZ **83** (in absence of ^{t}BuOK)	33
7	PC-Acr (in absence of ^{t}BuOK)	0
8	4-CzIPN (in absence of ^{t}BuOK)	12

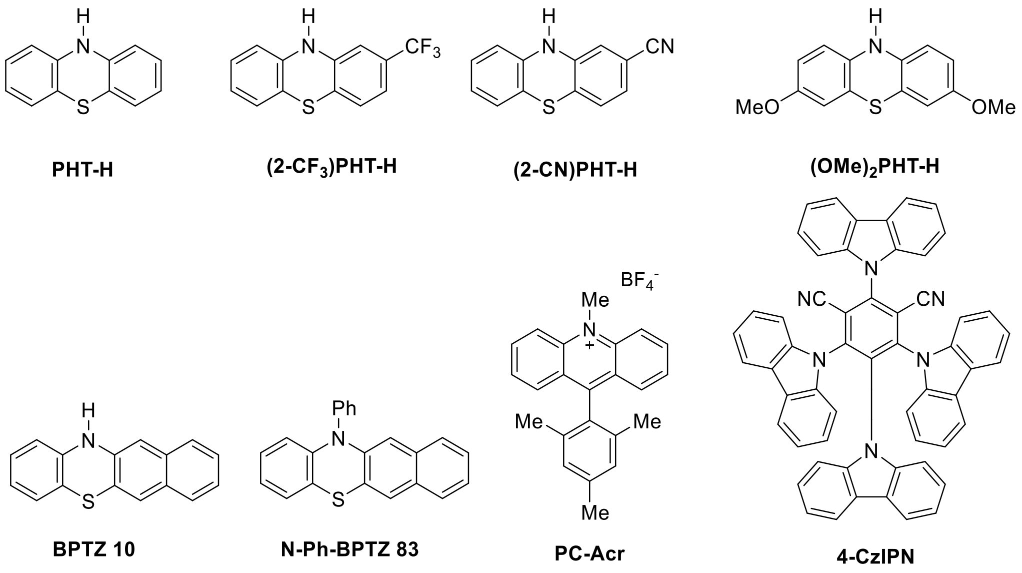

Supplementary Table. S-2 Evaluation of the Base

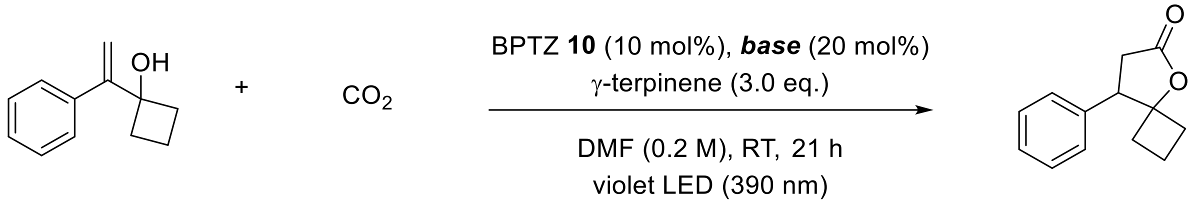

Entry	Base (20 mol%)	Product (% yield)
1	^tBuOLi	55
2	^tBuONa	64
3	^tBuOK	77
4	^tAmylOK	74
5	KOH	68
6	KCl	23
7	$KOOCCH_3$	69
8	K_3PO_4	65
9	Cs_2CO_3	63

Supplementary Table. S-3 Evaluation of the Loading and Ratio of PC/Base

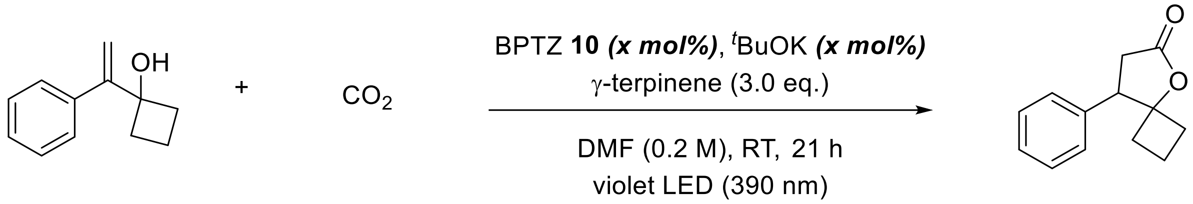

Entry	BPTZ (x mol%)	*t*BuOK (x mol%)	Ratio PC/base	Product (% yield)
1	5	5	1	54
2	10	10	1	63
3	15	15	1	63
4	20	20	1	65
5	20	100	0.02	70
6	20	40	0.5	73
7	5	10	0.5	74
8	**10**	**20**	**0.5**	**77**
9	5	15	0.33	65
10	10	30	0.33	69

Important note: once it was established that the best photocatalyst/base combination resulted to be 12H-benzo[b]phenothiazine and potassium tert-butoxide; for the subsequent optimization studies, a stock solution of the catalyst mixture was prepared. The procedure changed as follows

A stock solution of the catalyst mixture was prepared weighing 20 mg of 12*H*-benzo[*b*]phenothiazine and 18 mg of freshly sublimed potassium *tert*-butoxide and accurately dissolved in 4.0 mL of dry DMF (resulting molarity [12*H*-benzo[*b*]phenothiazine] = 0.02 M; ^{t}BuOK = 0.04 M). In a 10 mL Schlenk tube equipped with a stir bar, the ketone-derived tertiary alcohol (0.2 mmol, 1.0 equiv.), γ-terpinene and 1.0 mL of the freshly prepared catalyst stock solution was added under air. The resulting mixture was subsequently degassed *via* freeze-pump-thaw method (3 cycles), backfilled with 1 atm CO_2 and irradiated with visible light. *The Schlenk tube was placed in a 3D-printed carousel, resulting in a final distance of 3 cm from the glass wall of the Schlenk tube to the Kessil lamp. The reaction temperature was kept constant using a fan placed on top of the reaction at 20 cm distance.*

After 21 hours, EtOAc (3 mL) was added to the Schlenk vial, and the reaction mixture was moved to a separating funnel. The Schlenk vial was rinsed with EtOAc (2 x 3 mL). Then this organic phase was washed three times with 2 M HCl water solution saturated with NaCl (5 mL). The organic phase was collected and separated, dried over $MgSO_4$, filtered and the solvents evaporated. Yield for optimization studies were determined from ^{1}H NMR of the crude reaction mixtures using 1,1,2,2-tetrachloroethane and methyl 3,5-dinitrobenzoate as internal standards.

Supplementary Table. S-4 Evaluation of the HAT donor

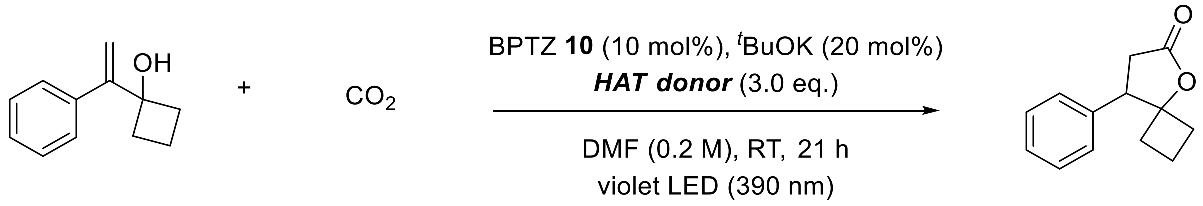

Entry	HAT donor (3.0 equiv)	Product (% yield)
1	γ-terpinene	77
2	1,4-CHD	62
3	Ar-SH	0
4	TIPS	8
5	DIPEA	0
6	H_2O	0
7	9,10-dihydroanthracene	0

Supplementary Table. S-5 Evaluation of the amount of γ-terpinene

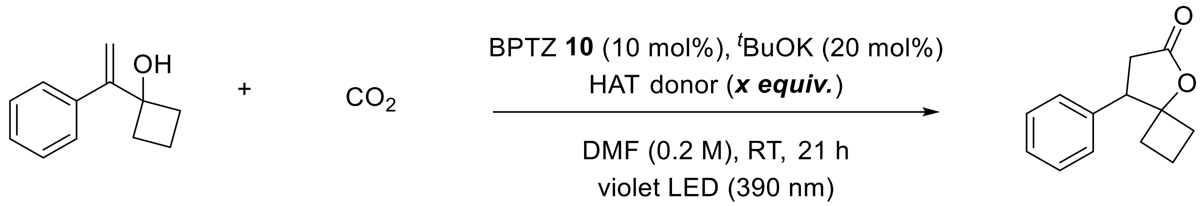

Entry	γ-terpinene (x equiv)	Product (% yield)
1	1.0 equiv.	12
2	2.0 equiv.	25
4	3.0 equiv.	77
5	4.0 equiv.	60

Supplementary Table. S-6 Solvent Screening

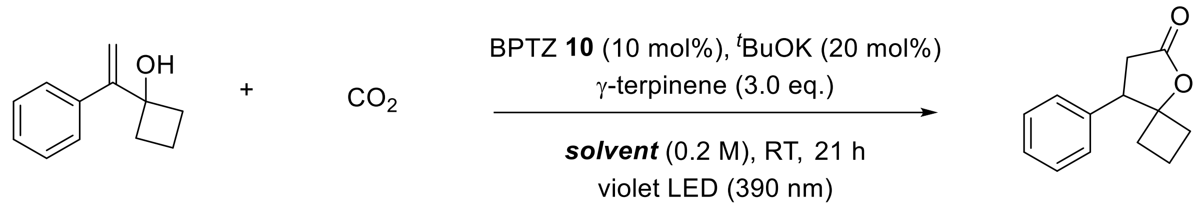

Entry	Solvent	Product (% yield)
1	DMF	77
2	DMSO	73
3	CH_3CN	36
4	NMP	74
5	DMA	58

Note: other solvents tested with no yield: MeOH, DCM, EtOAc, toluene, ethyl lactate.

Supplementary Table. S-7 Screening of the reaction concentration

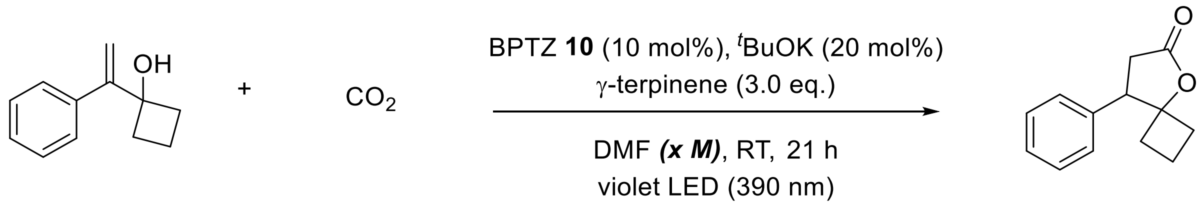

Entry	[SM]	Volume of DMF	Product (% yield)
1	0.40	0.5 mL	75
2	0.20	1 mL	77
3	0.10	2 mL	65
4	0.05	4 mL	45

Supplementary Table. S-8 Evaluation of reaction setup and delivery of CO_2

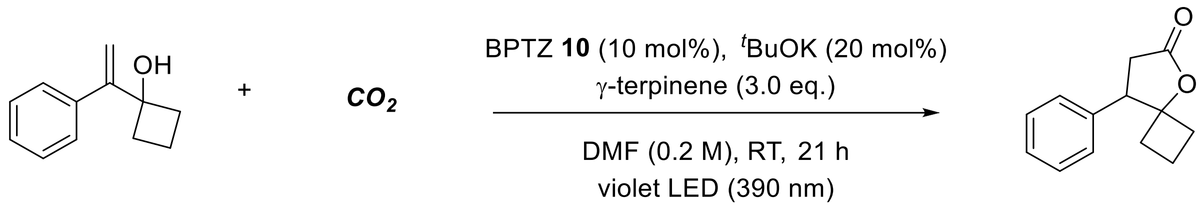

Entry	Delivery of CO_2	Reaction Vessel	Product (% yield)
1	Backfilling after freeze-pump-thaw	10 mL Schlenk Vial	77
2	Backfilling after freeze-pump-thaw	25 mL Schlenk Vial	76
3	Degassing reaction mixture with CO_2 for 10 min. through the septum of the MW vial	10 mL Microwave vial	58
4	Degassing reaction mixture with CO_2 for 10 min. through the septum, then leaving a 500 mL balloon of CO_2 pinched in during the reaction time	10 mL Microwave vial	54
5	Backfilling of 5 atm of CO_2 with high pressure tube after freeze-pump-thaw	Special 5 atm (pressured reactor)	76

Supplementary Table. S-9 control experiments

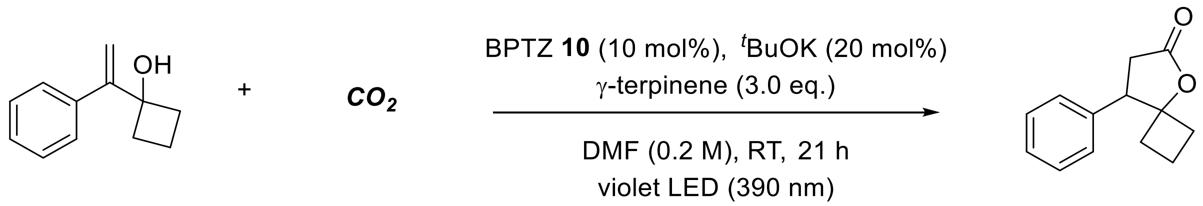

Entry	Variations	Product (% yield)
1	none	77
2	No light, at RT or at 50ºC	0
3	No catalyst	0
4	No base	38
5	No HAT donor	0

3. Synthesis of Starting Materials

General procedure A: synthesis of allyl alcohols from cyclic ketones *via* Grignard Reaction

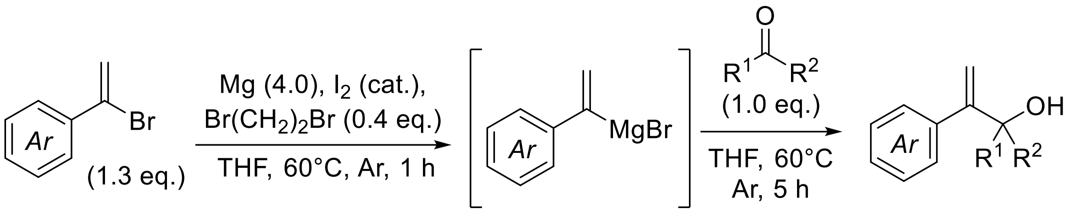

Allyl alcohols were synthesized adapting a procedure previously reported by Waser *et. al.*[1] A flame-dried 2-necked round bottom flask was connected to a reflux condenser and equipped with stir bar, magnesium (12 mmol, 4.0 equiv.), a crystal of iodine and 5 mL of THF under argon atmosphere. To the resulting suspension was added 1,2-dibromoethane (1.2 mmol, 0.4 equiv.), then a solution of the appropriate (1-bromovinyl)arene (3.9 mmol, 1.3 equiv.) in THF (1 M) was added dropwise. The reaction mixture was stirred for 1 hour at 60°C then the appropriate ketone (3.0 mmol, 1.0 equiv. for solid ketones a 1 M THF solution was prepared) was added dropwise at the same temperature. The reaction mixture was stirred at 60°C temperature for 5 hours (or until TLC shows the complete conversion of the ketone), cooled down to room temperature, quenched with NH_4Cl saturated aqueous solution, diluted with water (15 mL) and EtOAc (15 mL). The organic phase was separated, and the aqueous phase extracted three times with EtOAc (10 mL). The combined organic phases were washed once with brine (15 mL), dried over $MgSO_4$, filtered and concentrated in vacuo. The residue was purified by column chromatography on silica gel (Hexane:EtOAc 100:0 to 70:30, according to the substrate).

General procedure B: synthesis of 1-(2-arylallyl)cyclic alcohols from cyclic ketones *via* Barbier Reaction

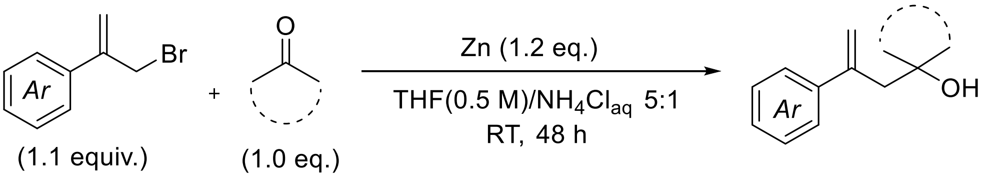

In a 50 mL round-bottomed flask equipped with a stir bar the appropriate cyclic ketone (5.0 mmol, 1.0 equiv.) was added to a solution of the corresponding allyl bromide (5.5 mmol, 1.1 equiv.) in THF (0.5 M) under air. Then, a saturated water solution of NH_4Cl (one fifth of the volume of THF used) was added to this mixture that was stirred vigorously for 2 minutes. Zinc dust (6.0 mmol, 1.2 equiv.) was added to the stirred solution and stirring was maintained for 48 hours at room temperature. At the end of the reaction time, 10 mL of saturated NH_4Cl water solution was added, and the mixture was extracted with EtOAc (3x15 mL). The

[1] S. Alazet, J. Preindl, R. Simonet-Davin, S. Nicolai, A. Nanchen, T. Meyer, J. Waser, *J. Org. Chem.* **2018**, *83*, 12334-12356.

combined organic phases were washed with brine (30 mL), dried over sodium sulfate, the drying agent was filtered off, and the solvent was evaporated. The residue was purified by column chromatography (silica gel, hexane/EtOAc from 95/05 to 70/30 according to the substrate) to provide the desired compound.

General procedure C: synthesis of 1-(2-arylallyl)cyclic alcohols from sterically demanding cyclic ketones *via* Barbier Reaction

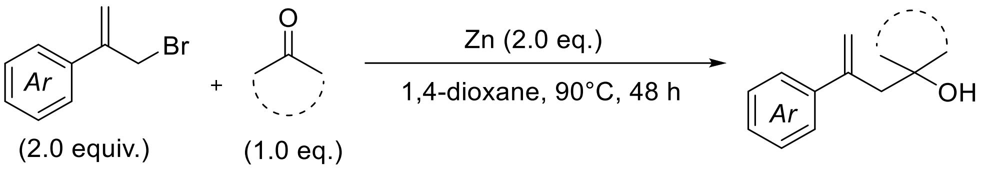

In a sealed vial equipped with a stir bar the appropriate cyclic ketone (5.0 mmol, 1.0 equiv.) was added to a solution of the corresponding allyl bromide (5.5 mmol, 1.1 equiv.) in 1,4-Dioxane (0.5 M) under air. Then zinc dust (6.0 mmol, 1.2 equiv.) was added to the stirred solution, and the sealed vial was placed in a silicone bath pre-heated at 90°C. Stirring was maintained for 48 hours at 90°C. At the end of the reaction time, the reaction mixture was moved to a separatory funnel, rinsing the vial with EtOAc, then 10 mL of saturated NH_4Cl water solution was added. The mixture was extracted with EtOAc (3x15 mL). The combined organic phases were washed with brine (30 mL), dried over sodium sulfate, the drying agent was filtered off, and the solvent was evaporated. The residue was purified by column chromatography (silica gel, hexane/EtOAc from 95:5 to 70:30 according to the substrate) to provide the desired compound.

General procedure D: synthesis of 3-arylbut-3-en-1-ols from linear ketones *via* Barbier Reaction

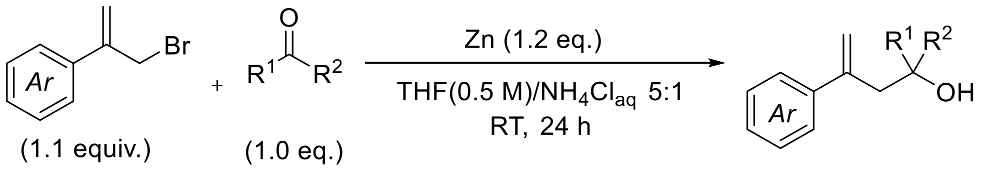

In a 50 ml round-bottomed flask equipped with a stir bar the appropriate linear ketone (5.0 mmol, 1.0 equiv.) was added to a solution of the corresponding allyl bromide (5.5 mmol, 1.1 equiv.) in THF (0.5 M) under air. Then, a saturated water solution of NH_4Cl (one fifth of the volume of THF used) was added to this mixture that was stirred vigorously for 2 minutes. Zinc dust (6.0 mmol, 1.2 equiv.) was added to the stirred solution and stirring was maintained for 24 hours at room temperature. At the end of the reaction time, 10 mL of saturated NH_4Cl water solution was added, and the mixture was extracted with EtOAc (3x15 mL). The combined organic phases were washed with brine (30 mL), dried over sodium sulfate, the drying agent was filtered off, and the solvent was evaporated. The residue was purified by column chromatography (silica gel, hexane/EtOAc from 95:5 to 90:10 according to the substrate) to provide the desired compound.

General procedure E: synthesis of 1-(1-bromovinyl)-arenes from α-methyl ketones

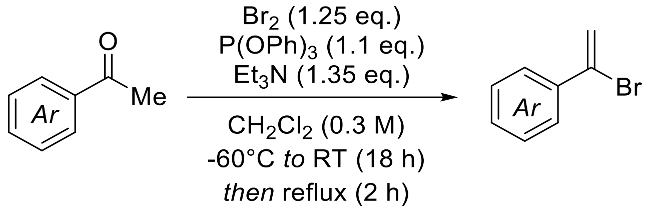

1-(1-bromovinyl)-arenes were synthesized from the required acetophenones adapting a previously reported procedure by Waser *et al.*[1] A flame-dried 100 mL round-bottom flask was backfilled with argon, equipped with a stir bar, then bromine (12.5 mmol, 1.25 equiv.) was added dropwise to a cold solution of triphenyl phosphite (11 mmol, 1.1 equiv.) in CH_2Cl_2 (0.3 M) at -60°C. Subsequently, a solution of triethylamine (13.5 mmol, 1.25 equiv.) and the corresponding acetophenone (10 mmol, 1.0 equiv.) in CH_2Cl_2 (1 M) was added dropwise to the orange solution. After that, the reaction was stirred for 18 hours while warming to room temperature. Then the mixture was heated to reflux for another 2 hours. The reaction was quenched with a saturated water solution of $Na_2S_2O_3$ (50 mL) and extracted with CH_2Cl_2 (3x30 mL). The combined organic extracts were washed with brine (150 mL), dried on sodium sulfate, filtered and the solvent was evaporated. The residue was purified by column chromatography deactivating the silica gel with triethylamine (under the stated conditions) to provide the desired compound which, due to low stability, was immediately used in the next step.

General procedure F: synthesis of 1-(1-bromovinyl)-arenes from styrenes

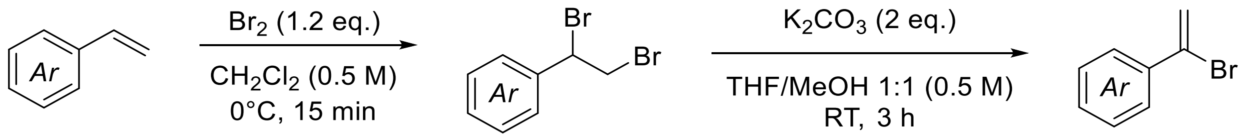

1-(1-bromovinyl)-arenes were synthesized from the corresponding styrenes adapting a previously reported procedure by Waser *et al.*[1] In a 100 mL round-bottom flask, backfilled with argon, equipped with a stir bar, bromine (12 mmol, 1.2 equiv.) was added dropwise to a cold solution of the styrene (10 mmol, 1.0 equiv.) in CH_2Cl_2 (0.5 M) over 5 minutes at 0°C. Thereafter, the reaction mixture was stirred for 15 minutes at 0°C and then quenched with a saturated water solution of $Na_2S_2O_3$ (50 mL). The mixture was extracted with CH_2Cl_2 (3x30 mL), and the combined organic extracts were washed with brine (150 mL) and dried over sodium sulfate. The drying agent was filtered off and the solvent evaporated. The residue was dissolved in THF/MeOH 1:1 (0.5 M), K_2CO_3 (20 mmol, 2.0 equiv.) was added, and the mixture was stirred for 3 hours at room temperature. Then, the solvent was evaporated, and the crude was suspended in water (50 mL) and extracted with hexane (3x50 mL). The combined organic extracts were washed with brine (150 mL) and dried over sodium sulfate. The drying agent was filtered off and the solvents evaporated. The residue was purified by column chromatography deactivating the silica gel with triethylamine (under the stated conditions) to provide the desired compound which, due to low stability, was immediately used in the next step.

General procedure G: synthesis of 1-(3-bromoprop-1-en-2-yl)-arenes *via* α-methyl styrenes

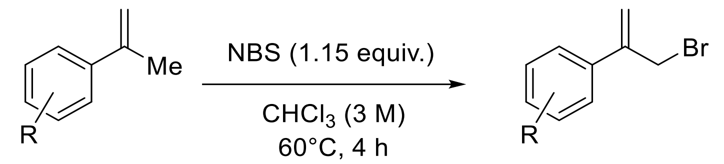

Adapting the procedure by Liu *et al.*[2], in a flame-dried 20 mL sealed tube, backfilled with argon, equipped with a stir bar, N-Bromosuccinimide (11.5 mmol, 1.15 equiv.) was added to a $CHCl_3$ solution (3 M) of the corresponding α-methyl styrene (10 mmol, 1.0 equiv.). The mixture was stirred and heated to 60°C for 4 hours. The reaction mixture was concentrated and CH_2Cl_2 was added. Next, the solution was dried over sodium sulfate, filtered off and evaporated. The crude product was purified by column chromatography deactivating the silica gel with triethylamine (under the stated conditions) to provide the desired compound which, due to low stability, was used immediately in the next step.

General Procedure for the amidation of pharmaceutically active carboxylic acid

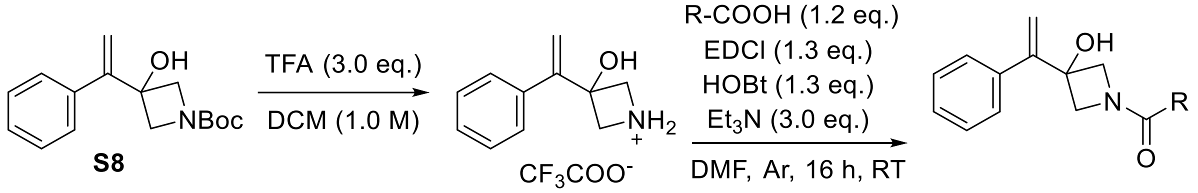

Step 1: 3-hydroxy-3-(1-phenylvinyl)azetidinium 2,2,2-trifluoroacetate. In a round-bottomed flask, *tert*-butyl 3-hydroxy-3-(1-phenylvinyl)azetidine-1-carboxylate **S8** was dissolved in a 1.0 M DCM solution, then Trifluoroacetic acid (3.0 eq.) was added dropwise and stirring was maintained for 30 min, until completion of the reaction. The DCM was removed with rotatory evaporator, then the residual TFA was eliminated under high vacuum. The resulting orange oil was used without further purification in the next step (92%yield by ^{1}H-NMR).

Step 2: amidation of carboxylic acid. In a 100 mL flame-dried round-bottomed flask, the carboxylic acid (1.2 equiv.), *N*-(3-dimethylaminopropyl)-*N*-ethylcarbodiimide hydrochloride (1.3 equiv.) and 1-Hydroxybenzotriazole hydrate (1.3 equiv.) were added under Ar then DMF was added to obtain a 1.0 M solution with respect to the acid. This resulting mixture was stirred under Ar for 30 minutes, then a 1.0 M solution of the azetidinium salt was added to the stirring solution followed by dropwise addition of Et_3N (3.0 equiv.). The resulting mixture was stirred for 16h at room temperature under Ar, then 15 mL of NaCl were added to quench the reaction, followed by 30 mL of EtOAc. The biphasic mixture was stirred for 15 minutes, then the organic phase was washed with brine (3x30 mL). The organic phase was reunited, dried over $MgSO_4$ filtered and evaporated. The resulting residue was purified by silica gel column chromatography.

---

[2] X. Dong, Y. Han, F. Yan, Q. Liu, P. Wang, K. Chen, Y. Li, Z. Zhao, Y. Dong and H. Liu, *Org. Lett.* **2016**, *18*, 3774-3777.

Synthesis of (3-bromoprop-1-en-2-yl)benzene (S1)

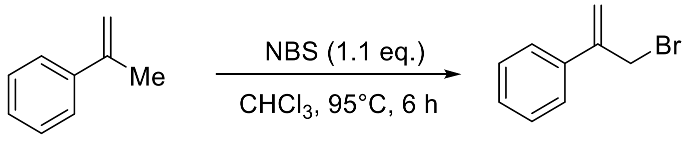

A flame-dried 50 mL sealed tube was charged, under an Argon atmosphere, with a stirring bar, *N*-Bromosuccinimide (9.8 g, 55.0 mmol, 1.1 equiv.), α-methylstyrene (6.5 mL, 5.9 g, 50.0 mmol, 1.0 equiv.) and dry Chloroform (10 mL). The tube was sealed and placed in a pre-heated 95°C oil bath and the reaction was stirred at this temperature for 6 hours. Then it was removed from the bath, let cool to room temperature, and the reaction mixture was moved to a 500 mL Erlenmeyer flask, rinsing the tube with the minimal amount of DCM. Then 400 mL of hexane were added, and the resulting mixture stirred for 5 minutes before filtering off the white solid on Buchner filtering funnel with fine frit. The solid was discarded and the filtrate liquid was collected and evaporated to afford a brown oil that was purified by flash column chromatography (100% Hexane) to provide the title compound as a colorless oil (5 g, 52% yield). The final product is stable enough to be used for weeks when stored between 2-8°C under Ar. Characterization data are in accordance with previous reports.[3] ^{1}H NMR (300 MHz, $CDCl_3$) δ 7.54 – 7.45 (m, 2H), 7.44 – 7.30 (m, 3H), 5.56 (s, 1H), 5.50 (s, 1H), 4.39 (s, 2H).

Note: some of the byproducts resulting from this reaction are lachrymatory and eye-irritant, we recommend caution in handling the crude mixture and during the purification. B) To achieve an optimal removal of byproducts traces eluting with the desired compound we recommend repeating the chromatography twice in 100% Hexanes.

Synthesis of the starting material for the model reaction: 1-(1-phenylvinyl)cyclobutan-1-ol (12)

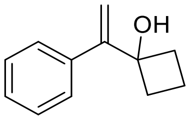

Prepared according to general procedure **A** using cyclobutanone (0.225 mL, 3 mmol). The crude product was purified by flash column chromatography (99/1 to 90/10 of Hexane/EtOAc) to provide the title compound as a colorless oil (436 mg, 83% yield). Characterization data are in accordance with previous reports.[1] ^{1}H NMR (300 MHz, $CDCl_3$) δ 7.52 – 7.42 (m, 2H), 7.39 – 7.23 (m, 3H), 5.38 (d, J = 1.0 Hz, 1H), 5.35 (d, J = 1.0 Hz, 1H), 2.56 – 2.40 (m, 2H), 2.39 – 2.16 (m, 2H), 2.08 – 1.89 (m, 2H), 1.72 – 1.58 (m, 1H).

[3] M. D. Levin and F. D. Toste, *Angew. Chem. Int. Ed.* **2014**, *53*, 6211-6215.

1-(1-phenylvinyl)cyclopentan-1-ol (S2)

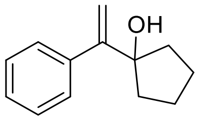

Prepared according to general procedure **A** using cyclopentanone (0.265 mL, 3 mmol). The crude product was purified by flash column chromatography (99/1 to 95/5 of Hexane/EtOAc) to provide the title compound as a yellow liquid (351 mg, 62% yield). Characterization data are in accordance with previous reports.[4] ^{1}H NMR (300 MHz, $CDCl_3$) δ 7.43 – 7.35 (m, 2H), 7.35 – 7.24 (m, 3H), 5.43 (d, *J* = 1.5 Hz, 1H), 5.07 (d, *J* = 1.5 Hz, 1H), 1.94 – 1.64 (m, 8H), 1.42 (s, 1H).

1-(1-phenylvinyl)cyclohexan-1-ol (S3)

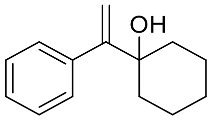

Prepared according to general procedure **A** using cyclohexanone (0.210 mL, 3 mmol). The crude product was purified by flash column chromatography (99/1 to 95/5 Hexane/EtOAc) to provide the title compound as a colorless liquid (439 mg, 72% yield). Characterization data are in accordance with previous reports.[2] ^{1}H NMR (300 MHz, $CDCl_3$) δ 7.35 – 7.23 (m, 5H), 5.44 (d, *J* = 1.3 Hz, 1H), 5.02 (d, *J* = 1.3 Hz, 1H), 1.74 – 1.50 (m, 10H), 1.45 (s, 1H).

1-(1-phenylvinyl)cycloheptan-1-ol (S4)

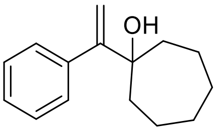

Prepared according to general procedure **A** using cycloheptanone (0.350 mL, 3 mmol). The crude product was purified by flash column chromatography (99/1 to 95/5 Hexane/EtOAc) to provide the title compound as a yellow oil (482 mg, 74% yield). Characterization data are in accordance with previous reports.[2] ^{1}H NMR (300 MHz, $CDCl_3$) δ 7.35 – 7.24 (m, 5H), 5.39 (d, *J* = 2.0 Hz, 1H), 4.96 (d, *J* = 2.0 Hz, 1H), 2.03 – 1.89 (m, 2H), 1.83 – 1.45 (m, 10H), 1.44 (s, 1H).

1-(1-phenylvinyl)cyclooctan-1-ol (S5)

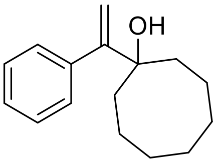

---

[4] Z. Shen, X. Pan, Y. Lai, J. Hu, X. Wan, X. Li, H. Zhang, W. Xie *Chem. Sci.*, **2015**, *6*, 6986–6990

Prepared according to general procedure **A** using cyclooctanone (380 mg, 3 mmol). The crude product was purified by flash column chromatography (99/1 to 95/5 Hexane/EtOAc) to provide the title compound as a white solid (361 mg, 52% yield). Characterization data are in accordance with previous reports.[5] ^{1}H NMR (300 MHz, $CDCl_3$) δ 7.36 – 7.23 (m, 5H), 5.36 (d, J = 1.4 Hz, 1H), 5.03 (d, J = 1.4 Hz, 1H), 1.96 – 1.75 (m, 4H), 1.68 – 1.46 (m, 10H), 1.37 (s, 1H).

3,3-diphenyl-1-(1-phenylvinyl)cyclobutan-1-ol (S6)

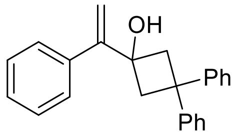

Step 1: 3,3-diphenylcyclobutan-1-one. Prepared according to a reported procedure[6] using ethene-1,1-diyldibenzene (540 mg, 3 mmol). The crude product was purified by flash column chromatography (99/1 to 95/5 Hexane/EtOAc) to provide the title compound as a white solid (546 mg, 82% yield). For a larger scale synthesis of the title compound, see below **36-Int-1**. Characterization data are in accordance with previous reports.[6] ^{1}H NMR (300 MHz, $CDCl_3$) δ 7.37 – 7.25 (m, 8H), 7.25 – 7.17 (m, 2H), 3.80 (s, 4H).

Step 2: Prepared according to general procedure **A** using 3,3-diphenylcyclobutan-1-one (665 mg, 3 mmol). The crude product was purified by flash column chromatography (99/1 to 95/5 Hexane/EtOAc) to provide the title compound as a yellow oil (710 mg, 72% yield). Characterization data are in accordance with previous reports.[7] ^{1}H NMR (300 MHz, $CDCl_3$) δ 7.47 – 7.09 (m, 15H), 5.31 (s, 1H), 5.30 (s, 1H), 3.43 – 3.31 (m, 2H), 3.27 – 3.15 (m, 2H), 2.00 (s, 1H).

(1*r*,3*r*,5*r*,7*r*)-2-(1-phenylvinyl)adamantan-2-ol (S7)

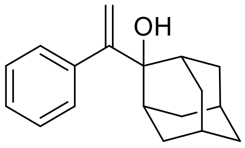

Prepared according to general procedure **A** using (1*r*,3*r*,5*r*,7*r*)-adamantan-2-one (450 mg, 3 mmol). The crude product was purified by flash column chromatography (99/01 to 98/02 Hexane/EtOAc) to provide the title compound as a white solid (221 mg, 29% yield). ^{1}H NMR (300 MHz, $CDCl_3$) δ 7.56 – 7.45 (m, 2H), 7.35 – 7.22 (m, 3H), 5.44 (d, J = 1.1 Hz, 1H), 5.20 (d, J = 1.1 Hz, 1H), 2.34 – 2.22 (m, 2H), 2.10 (apparent s, 2H), 1.89 – 1.75 (m, 4H), 1.67 – 1.53 (m, 7H). ^{13}C NMR (75 MHz, $CDCl_3$) δ 154.3, 141.7, 129.1, 127.8, 127.1, 115.7, 76.9, 37.8, 35.1, 34.9, 32.9, 27.5, 27.0. HRMS (APCI) [M+H]+ found 255.1735, $C_{18}H_{23}O$ requires 255.1743.

---

[5] Y. Liu, Y.Y. Yeung *Org. Lett.* **2019**, *19*, 1422–1425.
[6] Sietmann, J.; Tenberge, M.; Wahl, J. M. *Angew. Chem. Int. Ed.* **2023**, *62*, e202215381
[7] X.F. Song, A.H. Ye, Y.Y. Xie, J.W. Dong, C. Chen, Y. Zhang, Z.M. Chen *Org. Lett.*, **2019,** *21*, 9550–9554.

***tert*-butyl 3-hydroxy-3-(1-phenylvinyl)azetidine-1-carboxylate (S8)**

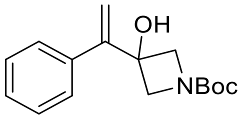

Prepared according to general procedure **A** using *tert*-butyl 3-oxoazetidine-1-carboxylate (515 mg, 3 mmol). The crude product was purified by flash column chromatography (80/20 to 70/30 of Hexane/EtOAc) to provide the title compound as a white solid (446 mg, 54% yield). Characterization data are in accordance with previous reports.[1] ^{1}H NMR (300 MHz, $CDCl_3$) δ 7.46 – 7.23 (m, 5H), 5.50 (d, J = 1.1 Hz, 1H), 5.40 (d, J = 1.1 Hz, 1H), 4.21 (d, J = 9.3 Hz, 2H), 4.04 (d, J = 9.3 Hz, 2H), 2.70 (s, 1H), 1.42 (s, 9H).

3-(1-phenylvinyl)thietan-3-ol (S9)

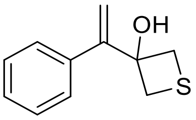

Prepared according to general procedure **A** using thietan-3-one (260 mg, 3 mmol). The crude product was purified by flash column chromatography (99/01 to 90/10 of Hexane/EtOAc) to provide the title compound as a yellow oil (319 mg, 55% yield). ^{1}H NMR (300 MHz, $CDCl_3$) δ 7.48 – 7.36 (m, 2H), 7.36 – 7.21 (m, 3H), 5.51 (d, J = 1.5 Hz, 1H), 5.41 (d, J = 1.5 Hz, 1H), 3.65 (d, J = 10.0 Hz, 2H), 3.38 (d, J = 10.0 Hz, 2H), 2.75 (s, 1H). ^{13}C NMR (75 MHz, $CDCl_3$) δ 151.5, 138.2, 128.5, 128.1, 127.9, 114.7, 80.4, 40.8. HRMS (APCI) [M+H]+ found 193.0674, $C_{11}H_{13}OS$ requires 193.0682

4-(1-phenylvinyl)tetrahydro-2*H*-pyran-4-ol (S10)

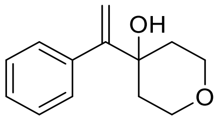

Prepared according to general procedure **A** using tetrahydro-4*H*-pyran-4-one (0.280 mL, 3 mmol). The crude product was purified by flash column chromatography (80/20 of Hexane/EtOAc) to provide the title compound as a white solid (418 mg, 68% yield). ^{1}H NMR (300 MHz, $CDCl_3$) δ 7.31 (s, 5H), 5.42 (s, 1H), 5.09 (s, 1H), 3.90 – 3.69 (m, 4H), 2.06 – 1.90 (m, 2H), 1.65 – 1.56 (m, 2H), 1.53 (s, 1H). ^{13}C NMR (75 MHz, $CDCl_3$) δ 156.1, 140.7, 129.1, 128.0, 127.4, 113.8, 71.1, 63.9, 37.0. HRMS (ESI) $[M+Na]^+$ found 227.1035, $C_{13}H_{16}NaO_2$ requires 227.1043

4-(1-phenylvinyl)tetrahydro-2*H*-thiopyran-4-ol (S11)

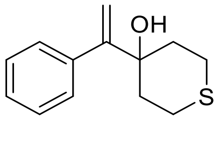

Prepared according to general procedure **A** using tetrahydro-4*H*-thiopyran-4-one (350 mg, 3 mmol). The crude product was purified by flash column chromatography (90/10 of Hexane/EtOAc) to provide the title compound

as a white solid (502 mg, 76% yield). ^{1}H NMR (300 MHz, $CDCl_3$) δ 7.35 – 7.19 (m, 5H), 5.42 (d, J = 1.1 Hz, 1H), 5.04 (d, J = 1.1 Hz, 1H), 3.14 – 2.99 (m, 2H), 2.47 – 2.33 (m, 2H), 2.11 – 1.90 (m, 4H), 1.34 (s, 1H). ^{13}C NMR (75 MHz, $CDCl_3$) δ 157.3, 140.8, 129.1, 128.1, 127.4, 113.8, 72.4, 37.9, 24.2. HRMS (APCI) $[M+H]^+$ found 221.0987, $C_{13}H_{17}OS$ requires 221.0995

8-(1-phenylvinyl)-1,4-dioxaspiro[4.5]decan-8-ol (S12)

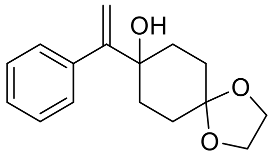

Prepared according to general procedure **A** using 1,4-dioxaspiro[4.5]decan-8-one (470 mg, 3 mmol). The crude product was purified by flash column chromatography (80/20 of Hexane/EtOAc) to provide the title compound as a colorless oil (546 mg, 70% yield). ^{1}H NMR (300 MHz, $CDCl_3$) δ 7.33 – 7.25 (m, 5H), 5.44 (d, J = 1.2 Hz, 1H), 5.04 (d, J = 1.2 Hz, 1H), 4.00 – 3.84 (m, 4H), 2.05 – 1.89 (m, 4H), 1.83 – 1.60 (m, 4H), 1.38 (s, 1H). ^{13}C NMR (75 MHz, $CDCl_3$) δ 156.3, 141.3, 129.1, 128.0, 127.2, 113.9, 108.6, 72.8, 64.4, 64.3, 34.6, 30.8. HRMS (APCI) $[M+H]^+$ found 261.1477, $C_{16}H_{21}O_3$ requires 261.1485

***tert*-butyl 2-hydroxy-2-(1-phenylvinyl)-7-azaspiro[3.5]nonane-7-carboxylate (S13)**

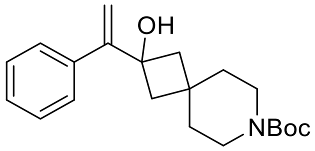

Prepared according to general procedure **A** using *tert*-butyl 2-oxo-7-azaspiro[3.5]nonane-7-carboxylate (720 mg, 3 mmol). The crude product was purified by flash column chromatography (95/05 to 90/10 of Hexane/EtOAc) to provide the title compound as a colorless oil (701 mg, 69% yield). ^{1}H NMR (300 MHz, $CDCl_3$) δ 7.52 – 7.42 (m, 2H), 7.39 – 7.23 (m, 3H), 5.37 (s, 1H), 5.34 (s, 1H), 3.40 – 3.30 (m, 2H), 3.30 – 3.20 (m, 2H), 2.36 – 2.26 (m, 2H), 2.17 – 2.07 (m, 2H), 1.92 (s, 1H), 1.80 – 1.70 (m, 2H), 1.44 (s, 9H), 1.42 – 1.38 (m, 2H). ^{13}C NMR (75 MHz, $CDCl_3$) δ 155.1, 154.2, 138.9, 128.4, 127.8, 127.6, 113.4, 79.4, 73.8, 45.2, 38.1, 37.9, 30.1, 28.6. HRMS (APCI) $[M+H]^+$ found 344.2214, $C_{21}H_{30}NO_3$ requires 344.2220

9-(1-phenylvinyl)-9*H*-xanthen-9-ol (S14)

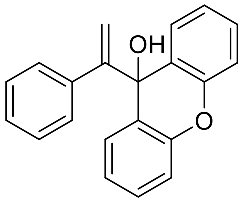

Prepared according to general procedure **A** using 9*H*-xanthen-9-one (590 mg, 3 mmol). The crude product was purified by flash column chromatography (90/10 of Hexane/EtOAc) to provide the title compound as a white solid (585 mg, 65% yield). ^{1}H NMR (300 MHz, $CDCl_3$) δ 7.68 – 7.58 (m, 2H), 7.37 – 7.22 (m, 2H), 7.18 –

6.92 (m, 7H), 6.70 – 6.61 (m, 2H), 6.03 (d, *J* = 1.7 Hz, 1H), 5.41 (d, *J* = 1.7 Hz, 1H), 2.56 (s, 1H). ^{13}C NMR (75 MHz, $CDCl_3$) δ 153.1, 150.1, 140.3, 129.5, 128.5, 128.5, 127.6, 127.0, 124.7, 123.5, 116.5, 114.1, 71.0.

1-(1-(*p*-tolyl)vinyl)cyclobutanol (S15)

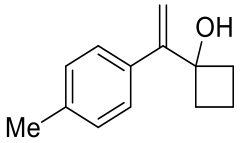

Prepared according to the general procedure **A** using 1-(1-bromovinyl)-4-methylbenzene (1.050 g, 5.33 mmol), cyclobutanone (287 mg, 4.1 mmol), magnesium (399 mg, 16.4 mmol), iodine (crystal), 1,2-DBE (308 mg, 1.64 mmol). The crude was purified by flash column chromatography (100/0 to 95/05 of Hexane/EtOAc) to provide the title compound as a colorless oil (404 mg, 52%). Characterization data are in accordance with the previous reports.[1] ^{1}H NMR (300 MHz, $CDCl_3$) δ 7.37 (d, *J* = 8.0 Hz, 2H), 7.13 (d, *J* = 8.0 Hz, 2H), 5.33 (s, 2H), 2.54-2.42 (m, 2H), 2.35 (s, 3H), 2.30-2.19 (m, 2H), 2.03-1.93 (m, 1H), 1.92 (s, 1H), 1.71-1.57 (m, 1H).

1-(1-(4-(*tert*-butyl)phenyl)vinyl)cyclobutanol (S16)

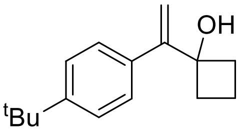

Prepared according to the general procedure **A** using 1-(1-bromovinyl)-4-(*tert*-butyl)benzene (995 mg, 4.16 mmol), cyclobutanone (224 mg, 3.2 mmol), magnesium (327 mg, 13.4 mmol), iodine (crystal), 1,2-DBE (241 mg, 1.28 mmol). The crude was purified by flash column chromatography (100/0 to 95/05 of Hexane/EtOAc) to provide the title compound as a colorless oil (339 mg, 46%). Characterization data are in accordance with the previous reports.[1] ^{1}H NMR (300 MHz, $CDCl_3$) δ 7.42 (d, *J* = 8.7 Hz, 2H), 7.38-7.32 (d, *J* = 8.7 Hz, 2H), 5.36 (s, 1H), 5.34 (s, 1H), 2.56-2.44 (m, 2H), 2.33-2.19 (m, 2H), 2.03-1.90 (m, 2H), 1.71-1.57 (m, 1H), 1.32 (m, 9H).

1-(1-(4-methoxyphenyl)vinyl)cyclobutanol (S17)

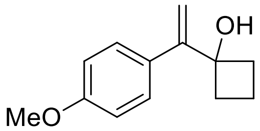

Prepared according to the general procedure **A** using 1-(1-bromovinyl)-4-methoxybenzene (873 mg, 4.01 mmol), cyclobutanone (221 mg, 3.15 mmol), magnesium (306 mg, 12.6 mmol), iodine (crystal), 1,2-DBE (237 mg, 1.26 mmol). The crude was purified by flash column chromatography (95/05 to 85/15 of Hexane/EtOAc) to provide the title compound as a colorless oil (450 mg, 70%). Characterization data are in accordance with the previous reports.[1] ^{1}H NMR (300 MHz, $CDCl_3$) δ 7.44 (d, *J* = 8.8 Hz, 2H), 6.86 (d, *J* = 8.7 Hz, 2H), 5.30 (s, 1H), 5.30 (s, 1H) 3.80 (s, 3H), 2.57-2.42 (m, 2H), 2.31-2.19 (m, 2H), 2.04-1.89 (m, 2H), 1.71-1.57 (m, 1H).

1-(1-(4-fluorophenyl)vinyl)cyclobutanol (S18)

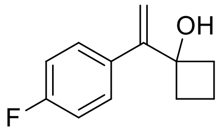

Prepared according to the general procedure **A** using 1-(1-bromovinyl)-4-fluorobenzene (1.555 g, 7.74 mmol), cyclobutanone (417 mg, 6.0 mmol), magnesium (578 mg, 23.8 mmol), iodine (crystal), 1,2-DBE (447 mg, 2.38 mmol). The crude was purified by flash column chromatography (95/05 to 90/10 of Hexane/EtOAc) to provide the title compound as a colorless oil (318 mg, 28%). Characterization data are in accordance with the previous reports.[1] ^{1}H NMR (300 MHz, $CDCl_3$) δ 7.53-7.41 (m, 2H), 7.08-6.95 (m, 2H), 5.36 (s, 1H), 5.32 (s, 1H) 2.51-2.37 (m, 2H), 2.29-2.14 (m, 2H), 2.06-1.92 (m, 1H), 1.86 (s, 1H), 1.71-1.51 (m, 1H).

1-(1-(*m*-tolyl)vinyl)cyclobutanol (S19)

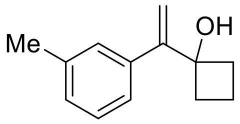

Prepared according to the general procedure **A** using 1-(1-bromovinyl)-3-methylbenzene (999 mg, 5.07 mmol), cyclobutanone (273 mg, 3.9 mmol), magnesium (398 mg, 16.4 mmol), iodine (crystal), 1,2-DBE (293 mg, 1.56 mmol). The crude was purified by flash column chromatography (100/0 to 95/05 of Hexane/EtOAc) to provide the title compound as a colorless oil (323 mg, 44%). Characterization data are in accordance with the previous reports.[1] ^{1}H NMR (300 MHz, $CDCl_3$) δ 7.32-7.18 (m, 3H), 7.15-7.08 (m, 1H), 5.36 (s, 1H), 5.33 (s, 1H), 2.54-2.43 (m, 2H), 2.36 (s, 3H), 2.32-2.19 (m, 2H), 2.06-1.91 (m, 1H), 1.71-1.55 (m, 2H).

1-(1-(thiophen-3-yl)vinyl)cyclobutanol (S20)

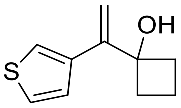

Prepared according to the general procedure **A** using 3-(1-bromovinyl)thiophene (280 mg, 1.48 mmol), cyclobutanone (80 mg, 1.14 mmol), magnesium (110 mg, 4.56 mmol), iodine (crystal), 1,2-DBE (86 mg, 0.46 mmol). The crude was purified by flash column chromatography (90/10 of Hexane/EtOAc) to provide the title compound as a white solid (560 mg, 49%). ^{1}H NMR (300 MHz, $CDCl_3$) δ 7.44-7.36 (m, 1H), 7.31-7.22 (m, 2H), 5.47 (s, 1H), 5.30 (s, 1H), 2.56-2.40 (m, 2H), 2.32-2.16 (m, 2H), 2.07-1.89 (m, 2H), 1.71-1.52 (m, 1H). ^{13}C NMR (75 MHz, $CDCl_3$) δ 146.5, 139.1, 127.3, 125.2, 122.5, 111.4, 78.2, 35.5, 13.6. HRMS (APCI) found $[M+H]^+$ 181.0677 m/z, $C_{10}H_{13}OS$ requires 181.0682 m/z.

Synthesis of 3,3-diphenyl-1-(1-(phenylsulfonyl)vinyl)cyclobutanol (36)

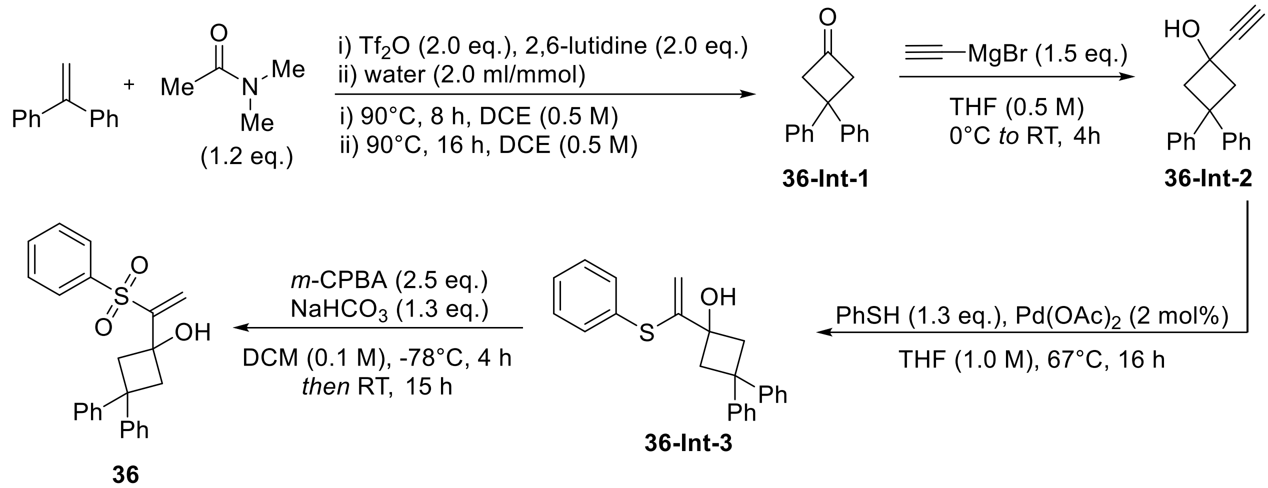

1st step. 3,3-diphenylcyclobutan-1-one (36-Int-1): A two-neck round-bottom 100 mL flask equipped with a stir bar and a reflux condenser was backfilled with argon. Dimethylacetamide (3.3 mL, 36 mmol) was dissolved in 1,2-dichloroethane (60 mL). Triflic anhydride (9.9 mL, 60 mmol) was added dropwise, and the reaction mixture was stirred for 10 minutes. A solution of 1,1-Diphenylethylene (5.3 mL, 30 mmol) and 2,6-lutidine (6.95 mL, 60 mmol) in 1,2-dichloroethane (2 M) was added dropwise to the reaction mixture and stirred at 90°C for 8 hours. The reaction was allowed to cool to room temperature and water (1 mL/equiv.) was added. Then, the reaction mixture was stirred at 90°C for 16 hours. The mixture was allowed to cool to room temperature, and the organic phase was separated. The aqueous phase was extracted with CH_2Cl_2 (3x50 mL). The combined organic extracts were dried over Na_2SO_4, filtered and the solvent was evaporated. The residue was purified by column chromatography (silica gel) (95/05 of hexane/EtOAc) to provide the titled compound as a pale-yellow solid (4.21 g, 63%). ^{1}H NMR (300 MHz, $CDCl_3$) δ 7.38 – 7.28 (m, 8H), 7.25 – 7.18 (m, 2H), 3.81 (s, 4H). The ^{1}H NMR data is in accordance with the literature.[6]

2nd step. **1-ethynyl-3,3-diphenylcyclobutan-1-ol (36-Int-2)**: A 50 mL round-bottom flask equipped with a stir bar was backfilled with argon. 3,3-Diphenylcyclobutanone (1.56 g, 7 mmol) was dissolved in THF (17.5 mL) and cooled to 0°C. Ethynylmagnesium bromide (28 mL, 14 mmol, 0.5 M in THF) was added dropwise. The reaction was stirred for 3 hours at 0°C and was quenched with a saturated solution of NH_4Cl (20 mL). The mixture was extracted with Et_2O (3x20 mL). The combined organic phases were dried over Na_2SO_4, filtered and the solvent was evaporated. The residue was purified by column chromatography (silica gel) (93/07 to 91/09 of hexane/EtOAc) to provide the titled compound as a pale-yellow solid (1.6 g, 92%). ^{1}H NMR (300 MHz, $CDCl_3$) δ 7.39 – 7.26 (m, 8H), 7.22 – 7.08 (m, 2H), 3.43 (d, J = 12.4 Hz, 2H), 3.13 (d, J = 11.9 Hz, 2H), 2.42 (s, 1H), 2.16 (s, 1H). The ^{1}H NMR is in accordance with the literature.[8]

[8] Kim, K.-D.; Yeom, H.-S.; Shin, S.; Shin, S. *Tetrahedron* **2012**, *68*, 5241-5247.

3rd step. 3,3-diphenyl-1-(1-(phenylthio)vinyl)cyclobutan-1-ol (36-Int-3): A two-necked round bottom flask equipped with a reflux condenser and a stir bar was backfilled with argon. $Pd(OAc)_2$ (43 mg, 0.19 mmol), 1-ethynyl-3,3-diphenylcyclobutanol (1.56 g, 6.28 mmol) and thiophenol (5.52 mmol, 1.3 equiv.) were dissolved in THF (4 mL). The reaction mixture immediately deposited brown precipitate. The mixture was stirred at 67°C for 16 hours. After the reaction was completed, the mixture was filtered through Celite pad using ethyl acetate, and the filtrate was evaporated under reduced pressure. The residue was purified by column chromatography (silica gel) (97/03 of hexane/EtOAc) to provide the titled compound as white solid (1.08 g, 48%). ^{1}H NMR (300 MHz, $CDCl_3$) δ 7.46 – 7.22 (m, 13H), 7.20 – 7.05 (m, 2H), 5.34 (t, *J* = 1.0 Hz, 1H), 4.78 (t, *J* = 0.9 Hz, 1H), 3.43 (d, *J* = 13.2 Hz, 2H), 3.19 – 3.05 (m, 2H), 2.26 (d, *J* = 1.7 Hz, 1H). ^{13}C NMR (75 MHz, $CDCl_3$) δ 150.9, 149.6, 149.1, 133.8, 129.4, 128.6, 128.4, 128.3, 126.4, 125.8, 125.8, 112.9, 74.39, 48.11, 43.7. HRMS (APCI) found $[M+H]^+$ 359.1450, $C_{24}H_{23}OS$ requires 359.1464.

4th step. 3,3-diphenyl-1-(1-(phenylsulfonyl)vinyl)cyclobutan-1-ol (36): A 50 mL round-bottom flask equipped with a stir bar was backfilled with argon. To a mixture of 3,3-diphenyl-1-(1-(phenylthio)vinyl)cyclobutanol (257 mg, 0.72 mmol) and sodium bicarbonate (150 mg, 1.79 mmol) in dichloromethane (7.2 mL) was added dropwise a dichloromethane solution of *m*-chloroperbenzoic acid (309 mg, 1.79 mmol) at -78°C and stirred at this temperature for 4 hours and the solution was stirred at room temperature for 15 hours. The resulting solution was quenched with a saturated solution of potassium carbonate, and the mixture was extracted with dichloromethane (3x30 mL). The combined organic layers were washed with brine, dried over sodium sulfate, filtered and concentrated *in vacuo*. The residue was purified by column chromatography (silica gel) (95/05 of hexane/EtOAc) to provide the titled compound as a white solid (185 mg, 66%). ^{1}H NMR (300 MHz, $CDCl_3$) δ 7.88 (d, *J* = 7.7 Hz, 2H), 7.66 (t, *J* = 7.4 Hz, 1H), 7.55 (t, *J* = 7.6 Hz, 2H), 7.33 – 7.01 (m, 10H), 6.22 (s, 1H), 5.83 (s, 1H), 3.50 (s, 1H), 3.26 (d, *J* = 12.6 Hz, 2H), 3.14 (d, *J* = 12.6 Hz, 2H). ^{13}C NMR (75 MHz, $CDCl_3$) δ 153.4, 149.1, 148.3, 141.1, 133.7, 129.3, 128.5, 128.2, 126.1, 126.1, 125.9, 125.9, 125.5, 71.6, 48.0, 43.8.

(*R*)-1-(3-hydroxy-3-(1-phenylvinyl)azetidin-1-yl)-2-(4-isobutylphenyl)propan-1-one (S21)

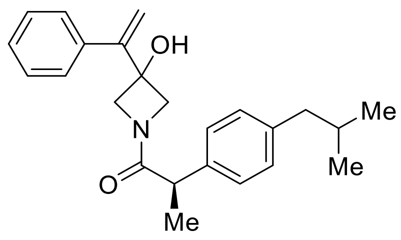

Prepared according to general procedure for the amidation of carboxylic acid using (*R*)-2-(4-isobutylphenyl)propanoic acid (680 mg, 3.3 mmol). The crude product was purified by flash column chromatography (50/50 of Hexane/EtOAc) to provide the title compound as a white solid (584 mg, 53% yield). ^{1}H NMR (300 MHz, $CDCl_3$, mixture of diastereoisomers) δ 7.46 – 7.26 (m, 10H), 7.21 – 7.02 (m, 8H), 5.51 (s, 1H), 5.41 (s, 1H), 5.34 (s, 1H), 5.16 (s, 1H), 4.36 – 3.98 (m, 8H), 3.56 – 3.45 (m, 2H), 2.50 – 2.41 (m, 4H),

1.96 – 1.75 (m, 2H), 1.45 – 1.33 (apparent triplet, 6H), 0.96 – 0.86 (apparent triplet, 12H). ^{13}C NMR (75 MHz, $CDCl_3$, mixture of diastereoisomers) δ 174.2, 174.1, 149.6, 149.4, 140.4, 140.4, 138.2, 138.2, 137.9, 137.8, 129.6, 129.5, 128.6, 128.5, 128.2, 128.1, 127.3, 127.3, 127.1, 127.1, 115.2, 114.9, 72.4, 72.3, 63.1, 62.9, 61.2, 61.0, 45.2, 45.1, 42.3, 42.2, 30.2, 22.5, 22.5, 22.4, 19.3, 19.2. HRMS (APCI) found $[M+H]^+$ 364.2275 m/z, $C_{24}H_{30}NO_2$ requires 364.2271 m/z.

5-(2,5-dimethylphenoxy)-1-(3-hydroxy-3-(1-phenylvinyl)azetidin-1-yl)-2,2-dimethylpentan-1-one (S22)

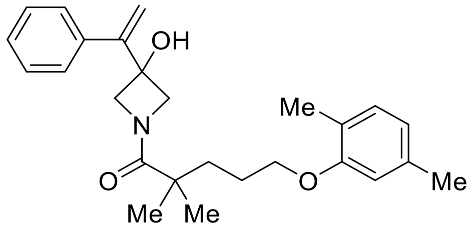

Prepared according to general procedure for the amidation of carboxylic acid using 5-(2,5-dimethylphenoxy)-2,2-dimethylpentanoic acid (826 mg, 3.3 mmol). The crude product was purified by flash column chromatography (50/50 of Hexane/EtOAc) to provide the title compound as a white solid (650 mg, 53% yield). ^{1}H NMR (300 MHz, $CDCl_3$) δ 7.46 – 7.25 (m, 5H), 6.99 (d, J = 7.4 Hz, 1H), 6.66 (d, J = 7.4 Hz, 1H), 6.61 (s, 1H), 5.49 (d, J = 1.7 Hz, 1H), 5.41 (d, J = 1.7 Hz, 1H), 4.54 – 4.06 (m, 4H), 3.90 (t, J = 5.7 Hz, 2H), 2.97 (s, 1H), 2.31 (s, 3H), 2.15 (s, 3H), 1.81 – 1.60 (m, 4H), 1.18 (s, 6H). ^{13}C NMR (75 MHz, $CDCl_3$) δ 177.1, 157.1, 149.7, 137.8, 136.6, 130.4, 128.7, 128.2, 127.2, 123.6, 120.9, 115.0, 112.2, 72.7, 68.1, 42.1, 36.9, 25.5, 25.2, 21.5, 16.0. HRMS (APCI) found $[M+H]^+$ 408.2533 m/z, $C_{26}H_{34}NO_3$ requires 408.2533 m/z.

2-(4-(2,2-dichlorocyclopropyl)phenoxy)-1-(3-hydroxy-3-(1-phenylvinyl)azetidin-1-yl)-2-methylpropan-1-one (S23)

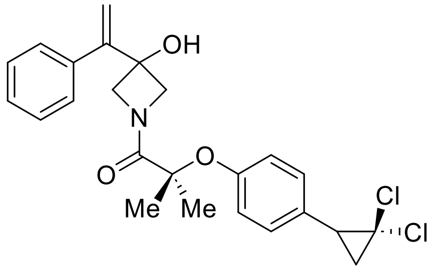

Prepared according to general procedure for the amidation of carboxylic acid using 2-(4-(2,2-dichlorocyclopropyl)phenoxy)-2-methylpropanoic acid (680 mg, 3.3 mmol). The crude product was purified by flash column chromatography (70/30 of Hexane/EtOAc) to provide the title compound as a white solid (855 mg, 64% yield). ^{1}H NMR (300 MHz, $CDCl_3$) δ 7.33 – 7.23 (m, 5H), 7.12 (d, J = 8.3 Hz, 2H), 6.82 (d, J = 8.1 Hz, 2H), 5.36 (d, J = 3.4 Hz, 1H), 5.20 (d, J = 3.4 Hz, 1H), 4.49 (d, J = 10.4 Hz, 1H), 4.40 – 4.27 (m, 2H), 4.14 (d, J = 10.3 Hz, 1H), 2.83 (t, J = 9.6 Hz, 1H), 2.01 – 1.89 (m, 1H), 1.78 (t, J = 7.9 Hz, 1H), 1.53 (s, 3H), 1.51 (s, 3H). HRMS (APCI) found $[M+H]^+$ 446.1289 m/z, $C_{24}H_{26}Cl_2NO_3$ requires 446.1284 m/z.

(8*S*,9*R*,13*R*,14*R*)-3-(methoxymethoxy)-13-methyl-17-(1-phenylvinyl)-7,8,9,11,12,13,14,15,16,17-decahydro-6*H*-cyclopenta[a]phenanthren-17-ol (S24)

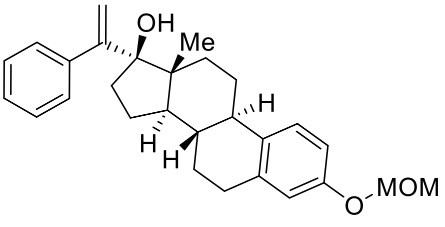

Prepared according to general procedure **A** using (8*S*,9*R*,13*R*,14*R*)-3-(methoxymethoxy)-13-methyl-6,7,8,9,11,12,13,14,15,16-decahydro-17*H*-cyclopenta[a]phenanthren-17-one (942 mg, 3 mmol). The crude product was purified by flash column chromatography (95/05 to 85/15 Hexane/EtOAc) to provide the title compound as a white solid (32 yield% of a mixture with one major diastereomer) The following NMR data refers to the major diastereomer. ^{1}H NMR (500 MHz, $CDCl_3$) δ 7.50 – 7.39 (m, 2H), 7.34 – 7.27 (m, 3H), 7.21 – 7.10 (m, 1H), 6.86 – 6.74 (m, 2H), 5.32 (d, *J* = 0.9 Hz, 1H), 5.19 (d, *J* = 1.0 Hz, 1H), 5.15 – 5.12 (m, 2H), 3.49 – 3.44 (m, 3H), 2.92 – 2.81 (m, 3H), 2.54 – 1.66 (m, 12H), 1.59 – 1.23 (m, 10H), 0.97 (s, 3H). ^{13}C NMR (126 MHz, $CDCl_3$) δ 155.6, 155.2, 142.2, 138.2, 134.1, 129.7, 127.9, 127.2, 126.4, 117.5, 116.4, 113.9, 94.7, 87.6, 56.0, 48.6, 43.7, 40.0, 39.7, 34.5, 29.9, 27.6, 26.60, 23.7. HRMS (APCI) found $[M+H]^+$ 419.2564 m/z, $C_{28}H_{35}O_3$ requires 419.2581 m/z.

1-(2-phenylallyl)cyclohexan-1-ol (S25)

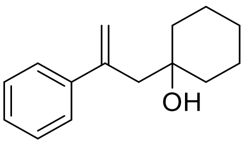

Prepared according to general procedure **B** using (3-bromoprop-1-en-2-yl)benzene (480 mg, 2.4 mmol) and cyclohexanol (200 mg, 2.0 mmol). The crude product was purified by flash column chromatography (98/02 to 95/5 of Hexane/EtOAc) to provide the title compound as a colorless oil (281.2 mg, 65% yield). Characterization data are in accordance with previous reports.[9] ^{1}H NMR (300 MHz, $CDCl_3$) δ 7.47 – 7.38 (m, 2H), 7.38 – 7.23 (m, 3H), 5.38 (s, 1H), 5.15 (s, 1H), 2.73 (s, 2H), 1.54 – 1.12 (m, 11H).

1-(2-phenylallyl)cyclobutan-1-ol (S26)

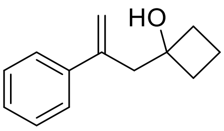

[9] M. Yoshida, K. Matsuda, Y. Shoji, T. Gotou, M. Ihara, K. Shishido *Org. Lett.* **2008** *10*, 5183-5186.

Prepared according to general procedure **B** using (3-bromoprop-1-en-2-yl)benzene (480 mg, 2.4 mmol) and cyclobutanol (145 mg, 2.0 mmol). The crude product was purified by flash column chromatography (98/02 to 95/05 of Hexane/EtOAc) to provide the title compound as a colorless oil (260 mg, 69% yield). Characterization data are in accordance with previous reports.[10] ^{1}H NMR (300 MHz, $CDCl_3$) δ 7.46 – 7.38 (m, 2H), 7.38 – 7.24 (m, 3H), 5.40 (s, 1H), 5.19 (s, 1H), 2.88 (s, 2H), 2.10 – 1.89 (m, 5H), 1.84 – 1.67 (m, 1H), 1.65 – 1.46 (m, 1H).

1-(2-phenylallyl)cyclopentan-1-ol (S27)

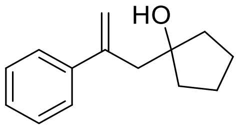

Prepared according to general procedure **B** using (3-bromoprop-1-en-2-yl)benzene (480 mg, 2.4 mmol) and cyclopentanol (172 mg, 2.0 mmol). The crude product was purified by flash column chromatography (98/02 to 95/5 of Hexane/EtOAc) to provide the title compound as a colorless oil (120 mg, 30% yield). Characterization data are in accordance with previous reports.[11] ^{1}H NMR (300 MHz, $CDCl_3$) δ 7.46 – 7.37 (m, 2H), 7.37 – 7.27 (m, 3H), 5.36 (d, J = 1.8 Hz, 1H), 5.19 – 5.15 (m, 1H), 2.86 (s, 2H), 1.79 – 1.65 (m, 2H), 1.60 – 1.43 (m, 8H).

1-(2-phenylallyl)cycloheptan-1-ol (S28)

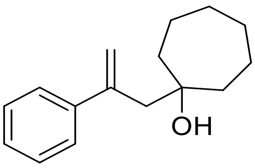

Prepared according to general procedure **C** using (3-bromoprop-1-en-2-yl)benzene (480 mg, 2.4 mmol) and cycloheptanol (225 mg, 2.0 mmol). The crude product was purified by flash column chromatography (97/03 to 95/5 Hexane/EtOAc) to provide the title compound as a colorless oil that turns to a white solid at low temperature (226 mg, 49% yield). ^{1}H NMR (300 MHz, $CDCl_3$) δ 7.50 – 7.41 (m, 2H), 7.41 – 7.25 (m, 3H), 5.40 (s, 1H), 5.18 (s, 1H), 2.77 (s, 2H), 1.81 – 1.56 (m, 8H), 1.53 – 1.28 (m, 5H). ^{13}C NMR (75 MHz, $CDCl_3$) δ 145.9, 128.6, 127.6, 126.7, 117.6, 75.6, 48.5, 41.4, 29.9, 22.4. HRMS (ESI) $[M+Na]^+$ found 253.1563, $C_{16}H_{22}NaO$ requires 253.1563

1-(2-phenylallyl)cyclooctan-1-ol (S29)

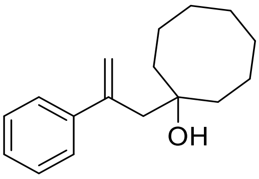

---

[10] R. Guo, G. Zhang *J. Am. Chem. Soc.* **2017**, *139*, 12891–12894.
[11] C. Zhao , Z. Tan , Z. Liang , W. Deng, H. Gong *Synthesis* **2014**, *46*, 1901-1907.

Prepared according to general procedure **C** using (3-bromoprop-1-en-2-yl)benzene (480 mg, 2.4 mmol) and cyclooctanol (252 mg, 2.0 mmol). The crude product was purified by flash column chromatography (97/03 to 90/10 Hexane/EtOAc) to provide the title compound as a white solid (271 mg, 55% yield). ^{1}H NMR (300 MHz, $CDCl_3$) δ 7.50 – 7.40 (m, 2H), 7.40 – 7.25 (m, 3H), 5.40 (s, 1H), 5.18 (s, 1H), 2.76 (s, 2H), 1.79 – 1.35 (m, 15H). ^{13}C NMR (75 MHz, $CDCl_3$) δ 145.9, 142.9, 128.5, 127.6, 126.7, 117.6, 75.1, 46.7, 36.4, 28.4, 25.0, 22.3. HRMS (ESI) $[M+Na]^+$ found 267.1719 $C_{17}H_{24}NaO$ requires 267.1719

1-(2-phenylallyl)cyclododecan-1-ol (S30)

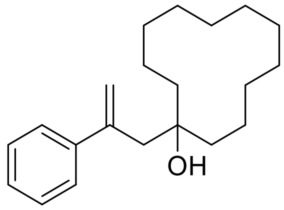

Prepared according to general procedure **C** using (3-bromoprop-1-en-2-yl)benzene (480 mg, 2.4 mmol) and cyclododecanone (360 mg, 2.0 mmol). The crude product was purified by flash column chromatography (97/03 to 90/10 of Hexane/EtOAc) to provide the title compound as a white solid (259 mg, 43% yield). ^{1}H NMR (300 MHz, $CDCl_3$) δ 7.48 – 7.40 (m, 2H), 7.40 – 7.25 (m, 3H), 5.38 (s, 1H), 5.20 (s, 1H), 2.70 (s, 2H), 1.60 – 1.46 (m, 2H), 1.40 – 1.26 (m, 18H). ^{13}C NMR (75 MHz, $CDCl_3$) δ 145.9, 128.5, 127.6, 126.8, 117.6, 75.5, 46.2, 35.1, 26.7, 26.2, 22.9, 22.5, 19.9. HRMS (ESI) $[M+Na]^+$ found 323.2351 $C_{21}H_{32}NaO$ requires 323.2345

2-(2-phenylallyl)adamantan-2-ol (S31)

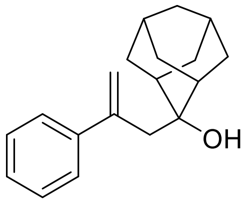

Prepared according to general procedure **C** using (3-bromoprop-1-en-2-yl)benzene (480 mg, 2.4 mmol) and 2-adamantanone (300 mg, 2.0 mmol). The crude product was purified by flash column chromatography (97/03 to 90/10 of Hexane/EtOAc) to provide the title compound as a white solid (217 mg, 41% yield). ^{1}H NMR (300 MHz, $CDCl_3$) δ 7.50 – 7.39 (m, 2H), 7.40 – 7.27 (m, 3H), 5.37 (d, J = 1.9 Hz, 1H), 5.21 (d, J = 1.9 Hz, 1H), 2.98 (s, 2H), 2.19 (apparent br d, 12.2 Hz, 1H), 1.94 – 1.73 (m, 4H), 1.73 – 1.53 (m, 8H), 1.47 (apparent br d, J = 12.2, Hz, 2H). ^{13}C NMR (75 MHz, $CDCl_3$) δ 146.3, 128.6, 127.6, 126.8, 118.0, 75.0, 43.6, 38.5, 37.5, 34.7, 33.2, 27.5. HRMS (ESI) $[M+Na]^+$ found 291.1722, $C_{19}H_{24}NaO$ requires 291.1719

8-(2-phenylallyl)-1,4-dioxaspiro[4.5]decan-8-ol (S32)

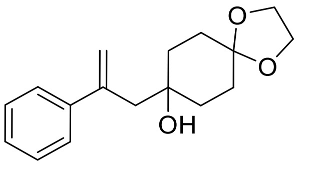

Prepared according to general procedure **B** using (3-bromoprop-1-en-2-yl)benzene (480 mg, 2.4 mmol) and 1,4-Cyclohexanedionemonoethylene acetal (315 mg, 2.0 mmol). Workup with ammonium chloride was avoided and the crude product was directly filtered on a Buchner filter with fine frit washed and rinsed with AcOEt. The resulting mixture was concentrated under reduced pressure and purified by flash column chromatography (95/05 to 80/20 Hexane/EtOAc) to provide the title compound as a white solid (190 mg, 35% yield). From the same crude also 4-hydroxy-4-(2-phenylallyl)cyclohexan-1-one could be isolated as byproduct as a colorless oil with the same chromatography (184 mg, 40% yield). ^{1}H NMR (300 MHz, $CDCl_3$) δ 7.49 – 7.39 (m, 2H), 7.39 – 7.27 (m, 3H), 5.43 (d, J = 1.8 Hz, 1H), 5.18 (d, J = 1.8 Hz, 1H), 4.00 – 3.85 (m, 4H), 2.78 (s, 2H), 1.97 – 1.76 (m, 2H), 1.68 – 1.58 (m, 4H), 1.56 – 1.48 (m, 2H), 1.38 (s, 1H). ^{13}C NMR (75 MHz, $CDCl_3$) δ 145.3, 128.7, 127.8, 126.6, 117.6, 108.9, 70.4, 64.4, 64.3, 47.7, 35.5, 30.6. HRMS (ESI) $[M+Na]^+$ found 297.1465 $C_{17}H_{22}NaO_3$ requires 297.1461

4,4-difluoro-1-(2-phenylallyl)cyclohexan-1-ol (S33)

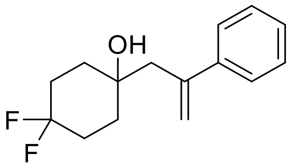

Prepared according to general procedure **B** using (3-bromoprop-1-en-2-yl)benzene (480 mg, 2.4 mmol) and 4,4-difluorocyclohexan-1-one (270 mg, 2.0 mmol). The crude product was purified by flash column chromatography (97/03 to 90/10 of Hexane/EtOAc) to provide the title compound as a white solid (348 mg, 69% yield). ^{1}H NMR (300 MHz, $CDCl_3$) δ 7.46 – 7.34 (m, 3H), 7.34 – 7.26 (m, 2H), 5.43 (s, 1H), 5.17 (s, 1H), 2.76 (s, 2H), 2.14 – 1.75 (m, 4H), 1.61 – 1.52 (m, 4H), 1.38 – 1.25 (m, 1H). ^{13}C NMR (75 MHz, $CDCl_3$) δ 144.9, 142.0, 128.9, 128.0, 126.6, 123.7 (app t, J = 240.9 Hz) 118.0, 69.9 (d, J = 1.4 Hz), 47.8 (d, J = 2.5 Hz), 34.4 (d, J = 9.2 Hz), 29.73 (app t, J = 23.8 Hz). ^{19}F NMR (282 MHz, $CDCl_3$) δ -92.3 (d, J = 234.6 Hz), -104.1 (dd, J = 235.0, 30.2 Hz). HRMS (APCI) [M-OH] found 235.1284 $C_{15}H_{17}F_2$ requires 235.1293

4-hydroxy-4-(2-phenylallyl)cyclohexan-1-one (S34)

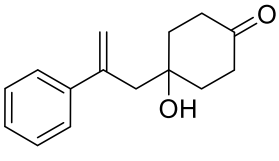

Prepared following the same procedure used to synthesize 8-(2-phenylallyl)-1,4-dioxaspiro[4.5]decan-8-ol, the title compound can be isolated from the same crude mixture with column chromatography (184 mg, 40% yield). Moreover, (2-phenylallyl)-1,4-dioxaspiro[4.5]decan-8-ol can be dissolved in a 1 M solution in acetone, and treated with vigorous stirring with the same volume of a 10% w/w HCl solution in water for 20 hour to recover the deprotected title compound upon evaporation of the solvents and isolation by column chromatography (96% yield recovered from 8-(2-phenylallyl)-1,4-dioxaspiro[4.5]decan-8-ol). ^{1}H NMR (300 MHz, $CDCl_3$) δ 7.45 – 7.28 (m, 5H), 5.44 (s, 1H), 5.20 (s, 1H), 2.84 (s, 2H), 2.64 (td, J = 14.0, 6.2 Hz, 2H),

2.23 – 2.09 (m, 2H), 1.91 – 1.82 (m, 2H), 1.70 (td, J = 13.5, 4.9 Hz, 2H), 1.60 (s, 1H). ^{13}C NMR (75 MHz, $CDCl_3$) δ 212.0, 144.9, 141.9, 128.9, 128.1, 126.6, 118.1, 70.2, 47.7, 37.6, 37.1. HRMS (APCI) $[M+Na]^+$ found 253.1200, $C_{15}H_{18}ONaO$ requires 253.1199

4-methylene-1-(2-phenylallyl)cyclohexan-1-ol (S35)

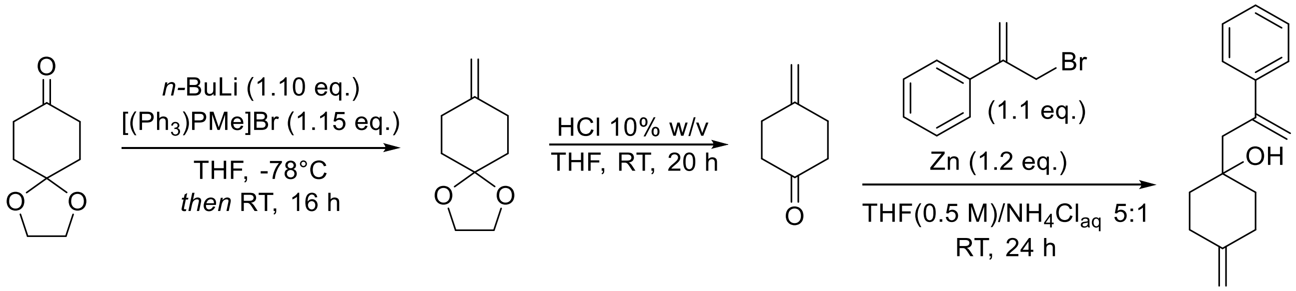

Step 1: **8-methylene-1,4-dioxaspiro[4.5]decane**: In a 100 mL flame-dried round-bottomed flask, *n*-BuLi (8.50 mL, 1.6 M in hexane, 21.15 mmol, 1.1 eq) was added to a solution of methyl triphenylphosphonium bromide (7.90 g, 22.11 mmol, 1.15 eq) in THF (40 mL) at -78° C. The resulting suspension was stirred for 10 min at -78° C., then for 30 min at 0° C. The orange mixture obtained was cooled again to -78° C., then a solution of the monoethylene ketal of 1,4-cyclohexanedione (3.0 g, 19.23 mmol) in THF (10 mL) was added dropwise under Argon. The resulting mixture was stirred at room temperature for 16 hours, then filtered on Celite rinsing the reaction vessel with hexane. After evaporating the solvents, the resulting crude product was purified by flash column chromatography (99/01 to 95/05 of Hexane/EtOAc) to provide the title compound as a colorless oil (2.7 g, 92%). Characterization data are in accordance with previous reports.[12] ^{1}H NMR (300 MHz, $CDCI_3$ δ 4.65 (s, 1H), 3.92 (s, 4 H), 2.27 (t, J = 6.4 Hz, 4 H), 1.70 (t, J = 6.4 Hz, 4 H).

Step 2: **4-methylenecyclohexan-1-one***:* To a solution of 8-methylene-1,4-dioxa-spiro[4.5]decane (2.5 g, 16 mmol) in tetrahydrofuran (20 mL) was added 10% aqueous hydrochloric acid (20 mL) and the resulting mixture was vigorously stirred for 20 h. The resulting solution was then extracted with ethyl ether (3x10 mL), and the combined organic extracts were dried over magnesium sulfate, filtered and concentrated to yield 1.45 g (80% yield) of 4-methylenecyclohexan-1-one as a yellowish oil that was used without further purification in the following step. Characterization data are in accordance with previous reports.[13] ^{1}H NMR (300 MHz, $CDCI_3$): 4.92 (s, 2H), 2.52-2.46 (m, 8H).

Step 3: **4-methylene-1-(2-phenylallyl)cyclohexan-1-ol (S35):** Prepared according to general procedure **B** using (3-bromoprop-1-en-2-yl)benzene (480 mg, 2.4 mmol) and 4-methylenecyclohexan-1-one (265 mg, 2.0 mmol). The crude product was purified by flash column chromatography (97/03 to 90/10 of Hexane/EtOAc) to provide the title compound as a colorless oil (297 mg, 65% yield). 1H NMR (300 MHz, $CDCl_3$) δ 7.45 – 7.38 (m, 2H), 7.37 – 7.28 (m, 3H), 5.40 (s, 1H), 5.16 (s, 1H), 4.58 (s, 2H), 2.74 (s, 2H), 2.30 (td, J = 12.7, 4.3

[12] A. Kapat, E. Nyfeler, G. T. Giuffredi, P. Renaud *J. Am. Chem. Soc.* **2009**, *131,* 17746–17747.
[13] B. Rickborn, M. T. Wuesthoff *J. Am. Chem. Soc.* **1970**, *92,* 6894–6904.

Hz, 2H), 2.05 (dt, J = 13.6, 4.3 Hz, 2H), 1.66 – 1.56 (m, 2H), 1.47 – 1.31 (m, 3H). ^{13}C NMR (75 MHz, $CDCl_3$) δ 148.9, 145.3, 142.5, 128.7, 127.8, 126.6, 117.6, 107.0, 71.1, 47.9, 39.2, 30.6. HRMS (ESI) $[M+Na]^+$ found 251.1407 $C_{16}H_{20}NaO$ requires 251.1406

4-(2-phenylallyl)tetrahydro-2*H*-pyran-4-ol (S36)

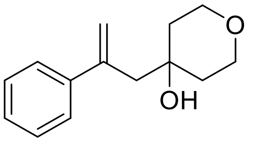

Prepared according to general procedure **B** using (3-bromoprop-1-en-2-yl)benzene (480 mg, 2.4 mmol) and tetrahydro-4*H*-pyran-4-one (200 mg, 2.0 mmol). The crude product was purified by flash column chromatography (98/02 to 85/15 Hexane/AcOEt) to provide the title compound as a colorless oil (327 mg, 75% yield). Characterization data are in accordance with previous reports.[14] ^{1}H NMR (300 MHz, $CDCl_3$) 7.45 – 7.38 (m, 2H), 7.37 – 7.26 (m, 3H), 5.42 (s, 1H), 5.17 (s, 1H), 3.70-3.65 (m, 3H), 3.66 (d, J = 2.3 Hz, 1H), 2.76 (s, 2H), 1.61 (ddd, J =14.0, 6.4, 3.4 Hz, 2H), 1.42 (s, 1H), 1.40-1.34 (m, 2H).

4-(2-phenylallyl)tetrahydro-2*H*-thiopyran-4-ol (S37)

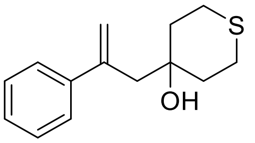

Prepared according to general procedure **B** using (3-bromoprop-1-en-2-yl)benzene (480 mg, 2.4 mmol) and tetrahydro-4*H*-thiopyran-4-one (235 mg, 2.0 mmol). The crude product was purified by flash column chromatography (98/2 to 85/15 of Hexane/EtOAc) to provide the title compound as a colorless oil (235 mg, 50% yield). Characterization data are in accordance with previous reports.[14] ^{1}H NMR (300 MHz, $CDCl_3$) δ 7.45 – 7.38 (m, 2H), 7.37 – 7.26 (m, 3H), 5.42 (d, J = 1.1 Hz, 1H), 5.16 (t, J = 1.1 Hz, 1H), 2.91 (t, J = 12.7 Hz, 2H), 2.72 (s, 2H), 2.32 (d, J = 13.6 Hz, 2H), 1.89 – 1.73 (m, 2H), 1.63 (ddd, J = 14.0, 11.7, 3.4 Hz, 2H), 1.32 (s, 1H).

1-(2-(4-fluorophenyl)allyl)cyclohexanol (S38)

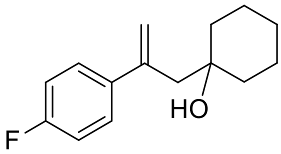

Prepared according to the general procedure **B** using 1-(3-bromoprop-1-en-2-yl)-4-fluorobenzene (653 mg, 3.04 mmol), cycloexanone (271 mg, 2.76 mmol) and zinc (217 mg, 3.31 mmol). The crude was purified by flash column chromatography (90/10 of Hexane/EtOAc) to provide the title compound as a colorless oil (376

[14] L. A.T. Cleghorn, R. Grigg, V. Savic, M. Simic, *Tetrahedron* **2008**, *64*, 8731-8737

mg, 58%). Characterization data are in accordance with the previous reports.[11] ^{1}H NMR (300 MHz, $CDCl_3$) δ 7.46-7.32 (m, 2H), 7.07-6.94 (m, 2H), 5.33 (s, 1H), 5.12 (s, 1H), 2.69 (s, 2H), 1.59-1.09 (m, 11H).

2-(2-(4-fluorophenyl)allyl)adamantan-2-ol (S39)

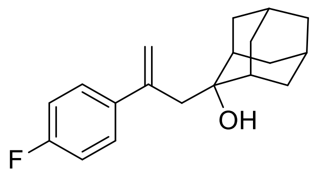

Prepared according to the general procedure **E** using 1-(3-bromoprop-1-en-2-yl)-4-fluorobenzene (613 mg, 2.84 mmol), 2-adamantanone (385 mg, 2.57 mmol) and zinc (201 mg, 3.08 mmol). The crude was purified by flash column chromatography (90/10 of Hexane/EtOAc) to provide the title compound as a colorless oil (510 mg, 69%). ^{1}H NMR (300 MHz, $CDCl_3$) δ 7.43-7.30 (m, 2H), 7.07-6.92 (m, 2H), 5.30 (s, 1H), 5.16 (s, 1H), 2.91 (s, 2H), 2.23-2.11 (m, 2H), 1.91-1.70 (m, 5H), 1.70-1.58 (m, 6H) 1.50-1.42 (m, 2H). ^{13}C NMR (75 MHz, $CDCl_3$) δ 164.1, 160.8, 145.2, 139.25, 139.21, 128.4, 128.3, 117.9, 115.5, 115.2, 75.0, 43.6, 38.5, 37.5, 34.7, 33.1, 27.4. ^{19}F NMR (282 MHz, $CDCl_3$) δ -115.09. HRMS (APCI) found [M-OH] 269.1700 m/z, $C_{19}H_{22}F$ requires 269.1700 m/z.

Synthesis of 2-(1-phenylvinyl)cyclohexan-1-ol (S40)

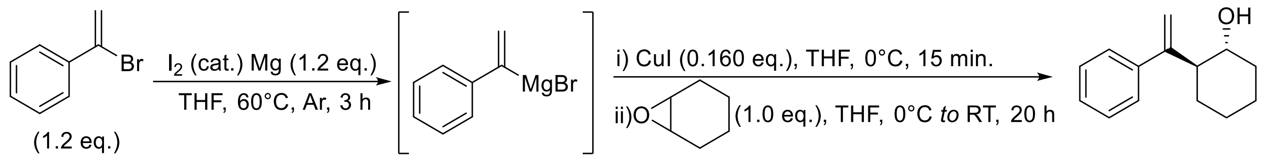

Two flame-dried two-necked flasks were prepared. In the first one, a stirring bar, magnesium powder (0.260 g, 10.80 mmol, 1.20 equiv.) and THF (2 mL) were added and the mixture stirred under Argon atmosphere. To this suspension, a crystal of iodine and a solution of α-bromostyrene (1.4 mL, 1.977 g, 10.80 mmol, 1.20 equiv.) in THF (1 M) were added under Ar, and this reaction mixture was stirred for 3 hours at 60°C, to obtain the corresponding Grignard reagent. Meanwhile, in the second flask a suspension of copper(I) iodide (0.275 g, 1.44 mmol, 0.16 equiv.) in THF (10 mL) was prepared under Ar atmosphere and cooled in a 0°C bath. Thereafter, the freshly prepared 1-(phenylvinyl)magnesium bromide from the first flask was added to this second flask at 0°C, and the reaction mixture was stirred for 15 min at the same temperature. Finally, a 2 M solution of the epoxide (cyclohexene oxide, 1.48 mL, 14.63 mmol) in THF (2 mL) was added dropwise under protected atmosphere at the same temperature, then the mixture was allowed warm up to room temperature for 20 hours. The reaction was then quenched with saturated aqueous NH_4Cl solution and diluted with EtOAc and the resulting biphasic mixture stirred for 5 minutes. The water phase was extracted three times with EtOAc. The combined organic layers were washed with brine, dried over $MgSO_4$, filtered and concentrated in vacuo

to obtain the resulting crude residue that was purified by flash column chromatography (95/05 of Hexane/EtOAc). Further purification by distillation (120–125°C, 2 mbar) provided the title compound as a colorless oil (1.18 g, 65% yield). Characterization data are in accordance with previous reports[15] ^{1}H NMR (300 MHz, $CDCl_3$): δ(ppm) 7.41–7.23 (m, 5H), 5.33 (s, 1H), 5.20 (s, 1H), 3.66 (m, 1H), 2.50–2.31 (m, 1H), 2.19–2.05 (m, 1H), 2.01 (br s, 1H), 1.90–1.74 (m, 2H), 1.70–1.64 (m, 1H), 1.43 – 1.30 (m, 2H), 1.30 – 1.14 (m, 2H).

2-methyl-4-phenylpent-4-en-2-ol (S41)

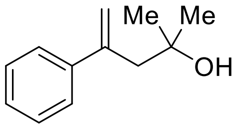

Prepared according to general procedure **D** using (3-bromoprop-1-en-2-yl)benzene (480 mg, 2.4 mmol) and acetone (116 mg, 0.150 mL, 2.0 mmol). The crude product was purified by flash column chromatography (98/02 to 95/5 of Hexane/EtOAc) to provide the title compound as a colorless oil (223 mg, 63% yield). Characterization data are in accordance with previous reports.[16] ^{1}H NMR (300 MHz, $CDCl_3$) δ 7.46 – 7.37 (m, 2H), 7.35 – 7.21 (m, 3H), 5.38 (d, J = 1.8 Hz, 1H), 5.15 (d, J = 1.8 Hz, 1H), 2.75 (s, 2H), 1.59 (s, 1H), 1.12 (s, 6H).

3-ethyl-5-phenylhex-5-en-3-ol (S42)

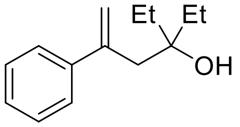

Prepared according to general procedure **D** using (3-bromoprop-1-en-2-yl)benzene (480 mg, 2.4 mmol) and 3-pentanone (170 mg, 0.210 mL, 2.0 mmol). The crude product was purified by flash column chromatography (98/02 to 95/05 Hexane/EtOAc) to provide the title compound as a colorless oil (274 mg, 67% yield). ^{1}H NMR (300 MHz, $CDCl_3$) δ 7.48 – 7.39 (m, 2H), 7.39 – 7.27 (m, 3H), 5.38 (d, J = 1.1 Hz, 1H), 5.18 (d, J = 1.1 Hz, 1H), 2.74 (s, 2H), 1.43 (q, J = 7.4 Hz, 4H), 1.32 (bs, 1H), 0.83 (t, J = 7.4 Hz, 6H). ^{13}C NMR (75 MHz, $CDCl_3$) δ 146.0, 128.5, 127.6, 126.7, 117.5, 74.9, 44.2, 31.0, 8.1. HRMS (ESI) $[M+Na]^+$ found 227.1407 $C_{14}H_{20}ONa$ requires 227.1406.

[15] B. Wang, D. A. Gandamana, D. F. León Rayo, F. Gagosz, S. Chiba, *Org. Lett.* **2019**, *21*, 9179–9182.
[16] M. Lübbesmeyer, E.G. Mackay, M.A. R. Raycroft, J. Elfert, D. A. Pratt, A. Studer *J. Am. Chem. Soc.* **2020**, *142, 5*, 2609–2616.

2-phenyl-4-propylhept-1-en-4-ol (S43)

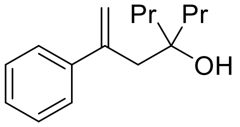

Prepared according to general procedure **D** using (3-bromoprop-1-en-2-yl)benzene (480 mg, 2.4 mmol) and 4-heptanone (230 mg, 0.280 mL, 2.0 mmol). The crude product was purified by flash column chromatography (98/02 to 95/5 of Hexane/EtOAc) to provide the title compound as a colorless oil (320 mg, 69% yield). ^{1}H NMR (300 MHz, $CDCl_3$) δ 7.47 – 7.40 (m, 2H), 7.40 – 7.26 (m, 3H), 5.37 (s, 1H), 5.17 (s, 1H), 2.73 (s, 2H), 1.62 (s, 1H), 1.43 – 1.21 (m, 8H), 0.80 (t, *J* = 6.5 Hz, 6H). ^{13}C NMR (75 MHz, $CDCl_3$) δ 146.1, 143.0, 128.5, 127.6, 126.8, 117.5, 74.6, 45.0, 41.6, 17.1, 14.6. HRMS (ESI) $[M+Na]^+$ found 255.1721, $C_{16}H_{24}NaO$ requires 255.1719.

3-(1-bromovinyl)thiophene (S44)

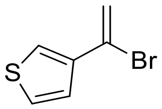

Prepared according to the general procedure **E** using 1-(thiophen-3-yl)ethanone (0.593 g, 4.7 mmol), Br_2 (0.939 g, 5.88 mmol), triphenyl phosphite (1.604 g, 5.17 mmol) and triethylamine (0.642 g, 6.34 mmol). The crude was purified by flash column chromatography (hexane) deactivating the silica gel with triethylamine to provide the title compound as a yellow oil (0.280 g, 32%). Characterization data in accordance with the previous reports.[17] ^{1}H NMR (300 MHz, $CDCl_3$) δ 7.51 (dd, *J* = 3.2, 1.3 Hz, 1H), 7.33 (dd, *J* = 3.0, 5.2 Hz, 1H), 7.28-7.23 (m, 1H), 6.10 (d, *J* = 2.0 Hz, 1H), 5.69 (d, *J* = 2.0 Hz, 1H).

1-(1-bromovinyl)-4-methylbenzene (S45)

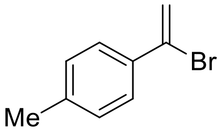

Prepared according to the general procedure **E** using 1-(*p*-tolyl)ethanone (1.342 g, 10 mmol), Br_2 (1.998 g, 12.5 mmol), triphenyl phosphite (3.413 g, 11 mmol) and triethylamine (1.366 g, 13.5 mmol). The crude was purified by flash column chromatography (hexane) deactivating the silica gel with triethylamine to provide the title compound as a colorless oil (1.050 g, 57%). Characterization data in accordance with the previous reports.[1] ^{1}H NMR (300 MHz, $CDCl_3$) δ 7.47 (d, *J* = 8.1 Hz, 2H), 7.15 (d, *J* = 7.9 Hz, 2H), 6.08 (d, *J* = 1.9 Hz, 1H), 5.73 (d, *J* = 1.9 Hz, 1H), 2.37 (s, 3H).

---

[17] Y. Bai, Z. Lin, Z. Ye, D. Dong, J. Wang, L. Chen, F. Xie, Y. Li, P.H. Dixneuf, M. Zhang *Org. Lett.* **2022**, *24*, 7988–7992

1-(1-bromovinyl)-4-methoxybenzene (S46)

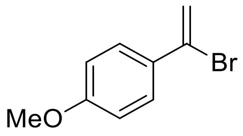

Prepared according to the general procedure **E** using 1-(4-methoxyphenyl)ethanone (1.502 g, 10 mmol), Br_2 (1.998 g, 12.5 mmol), triphenyl phosphite (3.413 g, 11 mmol) and triethylamine (1.366 g, 13.5 mmol). The crude was purified by flash column chromatography (100/0 to 95/05 of Hexane/EtOAc) deactivating the silica gel with triethylamine to provide the title compound as a colorless oil (1.191 g, 60%). Characterization data in accordance with the previous reports.[1] ^{1}H NMR (300 MHz, $CDCl_3$) δ 7.54 (d, *J* = 8.7 Hz, 2H), 6.87 (d, *J* = 8.9 Hz, 2H), 6.01 (d, *J* = 2.0 Hz, 1H), 5.67 (d, *J* = 2.0 Hz, 1H), 3.83 (s, 3H).

1-(1-bromovinyl)-3-methylbenzene (S47)

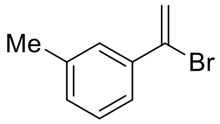

Prepared according to the general procedure **F** using 1-methyl-3-vinylbenzene (1.182 g, 10 mmol), Br_2 (1.918 g, 12 mmol) and K_2CO_3 (2.764 g, 20 mmol). The crude was purified by flash column chromatography (hexane) deactivating the silica gel with triethylamine to provide the title compound as a colorless oil (1.004 g, 51% yield). Characterization data in accordance with the previous reports.[1] ^{1}H NMR (300 MHz, $CDCl_3$) δ 7.42-7.37 (m, 2H), 7.27-7.20 (m, 1H), 7.17-7.12 (m, 1H), 6.10 (d, *J* = 2.0 Hz, 1H), 5.76 (d, *J* = 2.0 Hz, 1H), 2.38 (s, 3H).

1-(1-bromovinyl)-4-(*tert*-butyl)benzene (S48)

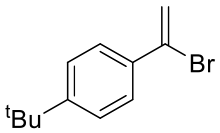

Prepared according to the general procedure **F** using 1-(*tert*-butyl)-4-vinylbenzene (1.603 g, 10 mmol), Br_2 (1.918 g, 12 mmol) and K_2CO_3 (2.764 g, 20 mmol). The crude was purified by flash column chromatography (hexane) deactivating the silica gel with triethylamine to provide the title compound as a colorless oil (2.031 g, 85%). Characterization data in accordance with the previous reports.[1] ^{1}H NMR (300 MHz, $CDCl_3$) δ 7.53 (d, *J* = 8.3 Hz, 2H), 7.36 (d, *J* = 8.4 Hz, 2H), 6.09 (d, *J* = 1.9 Hz, 1H), 5.74 (d, *J* = 1.9 Hz, 1H), 1.33 (s, 9H).

1-(1-bromovinyl)-4-fluorobenzene (S49)

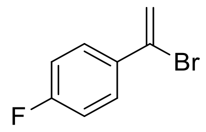

Prepared according to the general procedure **F** using 1-fluoro-4-vinylbenzene (1.221 g, 10 mmol), Br_2 (1.918 g, 12 mmol) and K_2CO_3 (2.764 g, 20 mmol). The crude was purified by flash column chromatography (hexane) deactivating the silica gel with triethylamine to provide the title compound as a colorless oil (1.556 g, 77% yield). Characterization data in accordance with the previous reports.[1] ^{1}H NMR (300 MHz, $CDCl_3$) δ 7.60-7.55 (m, 2H), 7.06-6.99 (m, 2H), 6.06 (d, *J* = 2.0 Hz, 1H), 5.76 (d, *J* = 2.0 Hz, 1H).

1-(3-bromoprop-1-en-2-yl)-4-fluorobenzene (S50)

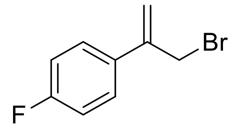

Prepared according to the general procedure **G** using 1-fluoro-4-(prop-1-en-2-yl)benzene (1.362 g, 10 mmol) and NBS (2.047 g, 11.5 mmol). The crude was purified by flash column chromatography (hexane) deactivating the silica gel with triethylamine to provide the title compound as a colorless oil (1.4 g, 65%). Characterization data in accordance with the previous reports.[18] ^{1}H NMR (300 MHz, $CDCl_3$) δ 7.52-7.42 (m, 2H), 7.12-7.00 (m, 2H), 5.50 (s, 1H), 5.47 (s, 1H), 4.35 (s, 2H).

***N*-methyl-*N*-(3-phenyl-3-(4-(trifluoromethyl)phenoxy)propyl)-4-vinylbenzamide (S51)**

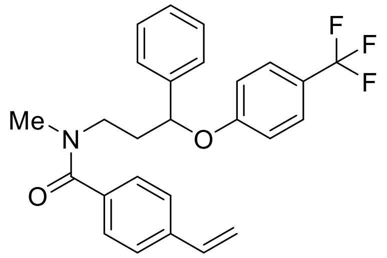

A 100 mL round bottomed flask was charged with Fluoxetine hydrogen chloride (1.38 g, 4 mmol), DMF (25 mL, 0.16M), 4-vinyl benzoic acid (770 mg, 5.2 mmol), EDC·HCl (997 mg, 5.2 mmol), 1-hydroxybenzotriazole (702 mg, 5.2 mmol). To this mixture triethylamine (0.83 mL, 6.0 mmol) was added dropwise at room temperature and the reaction was let to run 16 hours. After completion the crude was diluted with EtOAc (30 mL) and transferred to a separating funnel, and the organic phase was washed 4 times with brine (20 mL each) and dried using Na_2SO_4 and evaporated in vacuo. The crude was purified using flash column chromatography (80/20 to 70/30 of hexane/EtOAc) to provide the titled compound as a colorless liquid (1.26 g, 72%). ^{1}H NMR

[18] W.C. Jang, M. Jung, H.M. Ko *Org. Lett.* **2021**, *23,* 1510–1515

(500 MHz, $CDCl_3$) δ 7.47 – 7.18 (m, 10H), 6.93 (brs, 1H), 6.77 – 6.59 (m, 2H), 5.78 (d, *J* = 17.6 Hz, 1H), 5.32 (d, *J* = 10.9 Hz, 1.5H), 4.99 (brs, 0.5H), 3.89 – 3.34 (m, 2H), 3.04 (m, 3H), 2.45 – 2.06 (m, 2H). ^{13}C NMR (75 MHz, $CDCl_3$) δ 140.70, 138.79, 136.14, 135.29, 130.33, 128.99, 128.12, 127.37, 127.12, 126.82, 126.14, 125.79, 125.50, 123.64, 123.20, 122.77, 122.62, 122.34, 115.68, 115.24, 78.60, 47.85, 45.15, 37.98, 37.43, 36.12, 33.12. HRMS (APCI) $[M+H]^+$ found 440.1826 $C_{26}H_{25}F_3NO_2$ requires 440.1832.

4-(3-vinylphenoxy)butanoic acid (S52)

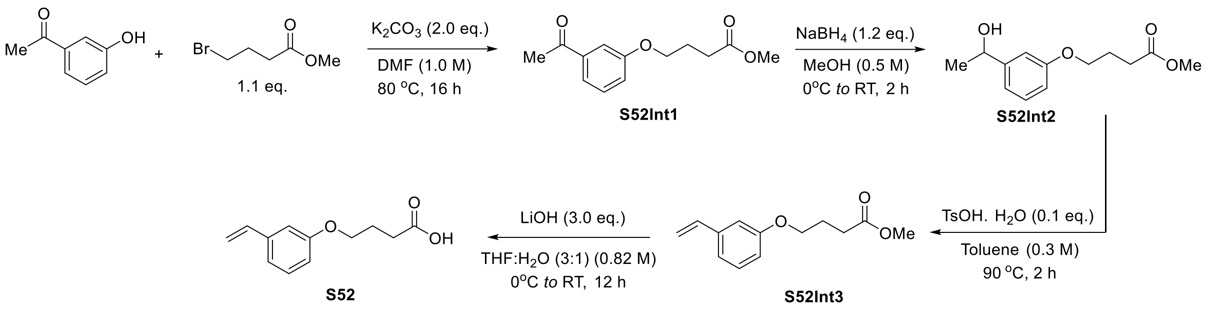

Step 1 - methyl 4-(3-acetylphenoxy)butanoate (S52Int1): To a 25 mL round bottomed flask was added 3-hydroxy acetophenone (2.45 g, 18 mmol) and DMF (18 mL, 1.0 M). K_2CO_3 (4.98 g, 36 mmol) and methyl 4-bromobutanoate (2.47 mL, 19.8 mmol) were sequentially added to the flask and the reaction mixture was run at a temperature of 80° C for 16 hours. After completion, the mixture was transferred to a separating funnel, and the organic phase was washed with brine 3 times (15 mL each). The separated organic phase was dried using a Na_2SO_4 and evaporated *in vacuo*. The crude was purified using flash column chromatography (96/04 of DCM/EtOAc) to provide **S52Int1** as a colorless liquid (3.49 g, 82% yield). ^1H NMR (300 MHz, $CDCl_3$) δ 7.52 (ddt, *J* = 7.6, 1.9, 0.9 Hz, 1H), 7.45 (dd, *J* = 2.7, 1.5 Hz, 1H), 7.35 (t, *J* = 7.8 Hz, 1H), 7.08 (ddt, *J* = 8.1, 1.9, 0.9 Hz, 1H), 4.05 (t, *J* = 6.0 Hz, 2H), 3.68 (s, 3H), 2.58 (s, 3H), 2.53 (t, *J* = 7.3 Hz, 2H), 2.18 – 2.06 (m, 2H). ^{13}C NMR (75 MHz, $CDCl_3$) δ 198.0, 173.6, 159.2, 138.6, 129.7, 121.3, 120.1, 113.2, 67.1, 51.7, 30.6, 26.8, 24.7. HRMS (APCI) $[M+H]^+$ found 237.1123 $C_{13}H_{17}O_4$ requires 237.1121.

Step 2 - methyl 4-(3-(1-hydroxyethyl)phenoxy)butanoate (S52Int2): A 50 mL round bottomed flask containing methyl 4-(3-acetylphenoxy)butanoate **S52Int1** (2.96 g, 12.5 mmol) and MeOH (25 mL) was maintained at a temperature of 0°C. Sodium borohydride (568 mg, 15.0 mmol) was added in portions, and the reaction was let to run at room temperature for 2 hours. After completion, 15 mL of NH_4Cl and 15 mL of EtOAc were added to the reaction mixture and was transferred to a separating funnel and the organic phase was washed 2 times with brine (15 mL each). The separated organic phase was dried using a Na_2SO_4 and evaporated *in vacuo*. The crude was purified using flash column chromatography (94/06 of DCM/EtOAc) to provide **S52Int1** as a colorless liquid (2.73 g, 92% yield). ^1H NMR (300 MHz, $CDCl_3$) δ 7.30 – 7.22 (m, 1H), 6.99 – 6.91 (m, 2H), 6.80 (ddd, *J* = 8.1, 2.6, 1.3 Hz, 1H), 4.93 – 4.82 (m, 1H), 4.03 (t, *J* = 6.1 Hz, 2H), 3.70 (s, 3H), 2.55 (t, *J* = 7.3 Hz, 2H), 2.19 – 2.07 (m, 2H), 1.95 (d, *J* = 3.0 Hz, 1H), 1.50 (d, *J* = 6.3 Hz, 3H). ^{13}C NMR (75 MHz, $CDCl_3$) δ 173.8, 159.2, 147.8, 129.6, 117.9, 113.5, 111.6, 70.4, 66.8, 51.7, 30.7, 25.3, 24.8. HRMS (APCI) $[M+H]^+$ found 238.1208 $C_{13}H_{18}O_4$ requires 238.1200.

Step 3 - methyl 4-(3-vinylphenoxy)butanoate (S52Int3): A 100 mL round bottomed flask was charged with methyl 4-(3-(1-hydroxyethyl)phenoxy)butanoate **S52Int2** (2.20g, 9.23 mmol) and toluene (31 mL, 0.3 M). *p*-Toluenesulfonic acid monohydrate (176 mg, 0.92 mmol) was added, and the reaction was let to run for 2 hours. After completion, the reaction was quenched with triethylamine and transferred to a separating funnel, and the organic phase was washed 2 times with brine (15 mL each). The separated organic phase was dried using a Na_2SO_4 and evaporated *in vacuo*. The crude was purified using flash column chromatography (96/04 of hexane/EtOAc) to provide **S52Int3** as a colorless liquid (691 mg, 34% yield). ^{1}H NMR (300 MHz, $CDCl_3$) δ 7.29 – 7.19 (m, 1H), 7.02 (ddd, *J* = 7.6, 1.7, 0.9 Hz, 1H), 6.96 (q, *J* = 2.3 Hz, 1H), 6.81 (dtd, *J* = 6.6, 3.3, 2.0 Hz, 1H), 6.76 – 6.63 (m, 1H), 5.76 (dt, *J* = 17.6, 0.7 Hz, 1H), 5.27 (dd, *J* = 10.9, 0.9 Hz, 1H), 4.05 (t, *J* = 6.1 Hz, 2H), 3.72 (s, 3H), 2.56 (t, *J* = 7.3 Hz, 2H), 2.20 – 2.08 (m, 2H). ^{13}C NMR (75 MHz, $CDCl_3$) δ 173.6, 159.1, 139.1, 136.8, 129.5, 119.0, 114.1, 114.0, 112.2, 66.6, 51.6, 30.5, 24.7. HRMS (ESI) $[M+Na]^+$ found 243.0984 $C_{13}H_{16}NaO_3$ requires 243.0992.

Step 3 - 4-(3-vinylphenoxy)butanoic acid (S52): LiOH (100 mg, 4.1 mmol) was added to a solution of the methyl 4-(3-vinylphenoxy)butanoate **S52Int3** (300 mg, 1.36 mmol) in THF-H_2O (3:1, 2.2 mL) at 0°C. The reaction was stirred at room temperature for 8 h. After completion (TLC analysis), the mixture was acidified with 2 N HCl to pH ≈2 and extracted with EtOAc (8 mL x 4). The combined organics were washed with brine (4 mL), dried over Na_2SO_4, filtered and concentrated *in vacuo*. The product was obtained as a colorless liquid (281 mg, 98%). ^{1}H NMR (300 MHz, $CDCl_3$) δ 7.30 – 7.21 (m, 1H), 7.02 (dt, *J* = 7.7, 1.3 Hz, 1H), 6.96 (t, *J* = 2.0 Hz, 1H), 6.82 (ddd, *J* = 8.2, 2.6, 1.0 Hz, 1H), 6.70 (dd, *J* = 17.6, 10.9 Hz, 1H), 5.76 (dd, *J* = 17.5, 1.0 Hz, 1H), 5.27 (dd, *J* = 10.9, 0.9 Hz, 1H), 4.06 (t, *J* = 6.1 Hz, 2H), 2.62 (t, *J* = 7.2 Hz, 2H), 2.22 – 2.09 (m, 2H). ^{13}C NMR (75 MHz, $CDCl_3$) δ 179.1, 159.1, 139.2, 136.9, 129.7, 119.3, 114.3, 114.1, 112.3, 66.6, 30.6, 24.6. HRMS (ESI) $[M+H]^+$ found 207.1012 $C_{12}H_{15}O_3$ requires 207.1016.

4. Photochemical Reactions

General Procedure H: Synthesis of γ-spirolactones

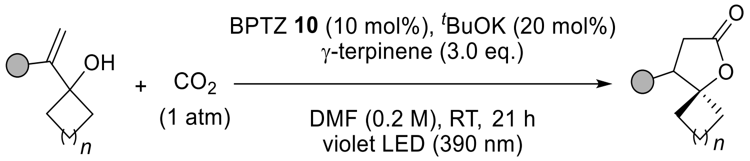

A stock solution of the catalyst mixture was prepared weighing 20 mg of 12*H*-benzo[*b*]phenothiazine and 18 mg of freshly sublimed potassium *tert*-butoxide and accurately dissolved in 4.0 mL of dry DMF (resulting molarity [12*H*-benzo[*b*]phenothiazine] = 0.02 M; ^{t}BuOK = 0.04 M). In a 10 mL Schlenk tube equipped with a stir bar, the ketone-derived tertiary alcohol (0.2 mmol, 1.0 equiv.), γ-terpinene (96 µL, 0.6 mmol, 3.0 equiv.) and 1.0 mL of the catalyst stock solution was added under air. *The use of anhydrous DMF serves to maximize the reproducibility of the reaction, although a small amount of water is tolerated*. The solution was subsequently degassed *via* freeze-pump-thaw method (3 cycles) and backfilled with 1 atm CO_2 and irradiated with visible light. The reaction mixture turned from red to yellow after backfilling. *The Schlenk tube was placed in a 3D-printed carousel, resulting in a final distance of 3 cm of the glass wall from the Kessil lamp. The reaction temperature was kept constant using a fan placed on top of the reaction at 20 cm distance.*

Note about freeze-pump-thaw: To perform the freeze-pump-thaw and backfilling with CO_2, the CO_2 cylinder was connected to the Schlenk line in order to replace the common Argon or Nitrogen line with a CO_2 line. For every cycle, the reaction mixture was frozen with liquid nitrogen and kept under vacuum leaving the stopcock of the Schlenk vial open for 4 minutes, Subsequently the stopcock was closed, the vial sealed and the mixture thawed with a 35°C water bath for 4 minutes. After the completion of three cycles, the Schlenk flask was sealed under vacuum, and the CO_2 gas valve on the Schlenk line was open. The stopcock of the Schlenk vial was opened to expose the reaction mixture to CO_2 while stirring, thus backfilling the Schlenk vial's atmosphere.

Important note: The Schlenk tube and the freeze-pump-thaw method were chosen to maximize the reproducibility of the reaction, given its sensitivity to oxygen. However, traces of oxygen can be tolerated (with lower yields) for example when the reaction was conducted in microwave vial, as noted in the optimization table S-8.

Two general procedures were adopted for the workup of the reaction:

WORKUP A: After 21 hours, EtOAc (3 mL) was added to the Schlenk vial and moved to a separating funnel. The Schlenk vial was rinsed with EtOAc (2x3 mL). Then this organic phase was washed three times with 2 M HCl water solution saturated with NaCl (5 mL). The organic phase was collected and separated. The aqueous phase was washed once with EtOAc (3 mL), and this second organic phase washed three times with 2 M HCl water solution saturated with NaCl (3 mL). The organic phase was reunited, dried over $MgSO_4$, filtered and the solvents evaporated. The resulting crude mixture was purified by silica flash column chromatography.

WORKUP B: After 21 hours, EtOAc (3 mL) was added to the Schlenk vial and moved to a separating funnel. The Schlenk vial was rinsed with EtOAc (2x3 mL). Then this organic phase was washed three times with 2 with NaCl (5 mL). The organic phase was reunited, dried over $MgSO_4$, filtered and the solvents evaporated. The resulting crude mixture was purified by silica flash column chromatography.

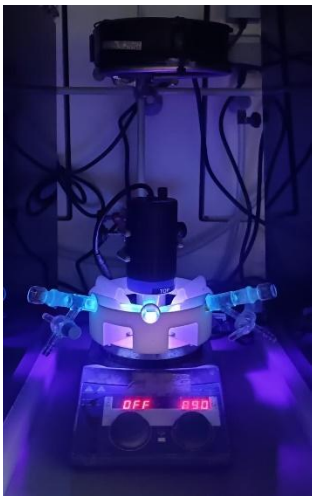

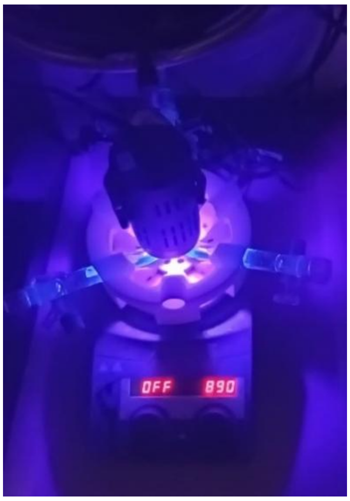

General Procedure I: Synthesis of sterically hindered γ-spirolactones

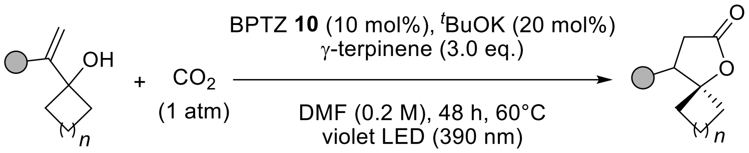

A stock solution of the catalyst mixture was prepared weighing 20 mg of 12*H*-benzo[*b*]phenothiazine and 18 mg of freshly sublimed potassium *tert*-butoxide and accurately dissolved in 4.0 mL of dry DMF (resulting molarity [12*H*-benzo[*b*]phenothiazine] = 0.02 M; ^{t}BuOK = 0.04 M). In a 10 mL Schlenk tube equipped with a stir bar, the ketone-derived tertiary alcohol (0.2 mmol, 1.0 equiv.), γ-terpinene (96 µL, 0.6 mmol, 3.0 equiv.) and 1.0 mL of the catalyst stock solution was added under air. *The use of anhydrous DMF serves to maximize the reproducibility of the reaction, although a small amount of water is tolerated*. The solution was subsequently degassed *via* freeze-pump-thaw method (3 cycles) and backfilled with 1 atm CO_2 and irradiated with visible light. The reaction mixture turned from red to yellow after backfilling.

The Schlenk tube was placed in a pre-heated glass-wall oil bath with temperature controlled at 60°C (a standard laboratory beaker with stirring bar was used). The reaction was irradiated through the glass wall with two 390 nm lights. Ventilation with a fan placed on top of the reaction at 20 cm distance was maintained to avoid overheating the 2 lamps and the reaction vessel (See figure for setting up details).

Two general procedures were adopted for the workup of the reaction:

WORKUP A: After 21 hours, EtOAc (3 mL) was added to the Schlenk vial and moved to a separating funnel. The Schlenk vial was rinsed with EtOAc (2x3 mL). Then this organic phase was washed three times with 2 M

HCl water solution saturated with NaCl (5 mL). The organic phase was collected and separated. The aqueous phase was washed once with EtOAc (3 mL), and this second organic phase washed three times with 2 M HCl water solution saturated with NaCl (3 mL). The organic phase was reunited, dried over $MgSO_4$, filtered and the solvents evaporated. The resulting crude mixture was purified by silica flash column chromatography.

WORKUP B: After 21 hours, EtOAc (3 mL) was added to the Schlenk vial and moved to a separating funnel. The Schlenk vial was rinsed with EtOAc (2x3 mL). Then this organic phase was washed three times with 2 with NaCl (5 mL). The organic phase was reunited, dried over $MgSO_4$, filtered and the solvents evaporated. The resulting crude mixture was purified by silica flash column chromatography.

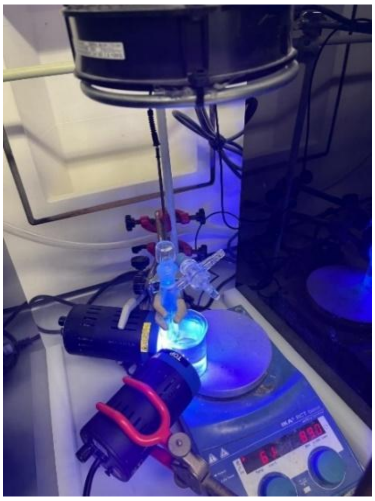

General Procedure J: Synthesis of δ-spirolactones

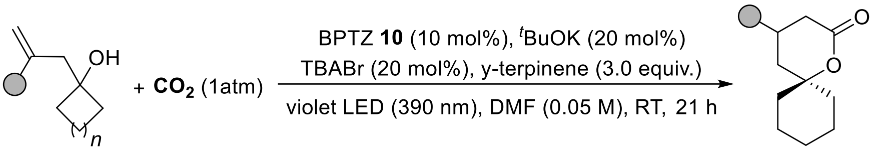

A stock solution of the catalyst mixture was prepared weighing 20 mg of 12*H*-benzo[*b*]phenothiazine and 27 mg of freshly sublimed potassium *tert*-butoxide and accurately dissolved in 4.0 mL of dry DMF (resulting molarity [12*H*-benzo[*b*]phenothiazine] = 0.02 M; ^{t}BuOK = 0.06 M). In a 10 mL Schlenk tube equipped with a stir bar, the ketone-derived tertiary alcohol (0.2 mmol, 1.0 equiv.), γ-terpinene (96 µL, 0.6 mmol, 3.0 equiv.), tetrabutylammonium bromide (13 mg, 0.04 mmol, 0.20 equiv.) and 1.0 mL of the catalyst stock solution and 3 mL of dry DMF was added under air. *The use of anhydrous DMF serves to maximize the reproducibility of the reaction, although a small amount of water is tolerated*. The solution was subsequently degassed *via* freeze-pump-thaw method (3 cycles) and backfilled with 1 atm CO_2 (the reaction mixture turned from red to yellow after backfilling) and irradiated with visible light. *The Schlenk tube was placed in a 3D-printed carousel, resulting in a final distance of 3 cm of the glass wall from the Kessil lamp. The reaction temperature was kept constant using a fan placed on top of the reaction at 20 cm distance.*

After 21 hours, EtOAc (3 mL) was added to the Schlenk vial and moved to a separating funnel. The Schlenk

vial was rinsed with EtOAc (2x3 mL). Then this organic phase was washed three times with 2 M HCl water solution saturated with NaCl (5 mL). The organic phase was collected and separated. The aqueous phase was washed once with EtOAc (3 mL), and this second organic phase washed three times with 2 M HCl water solution saturated with NaCl (3 mL). The organic phase was reunited, dried over $MgSO_4$, filtered and the solvents evaporated. The resulting crude mixture was purified by silica flash column chromatography.

General Procedure K: Synthesis of ^{13}C-labeled lactones

Note: the setup for these reactions used a 1 Liter, $^{13}CO_2$ cylinder purchased by Sigma (catalog code: 364592). As suggested with the provider, the use of a common manometer regulator with this gas cylinder is not recommended. The product is supplied at a filling pressure slightly above atmospheric pressure (20 psig for the 1-liter cylinder, thus a common manometer regulator will only allow to draw gas until the pressure equals the external pressure. The provider suggested using a flow control valve and removing the gas under vacuum to ensure a more complete extraction. The cylinder has a double threaded fitting (CGA 180M / CGA 110F), and suggested valves are referred to catalog code in Sigma with number Z146951 or Z187224, with Teflon ring CGA110 catalog code Z146986.

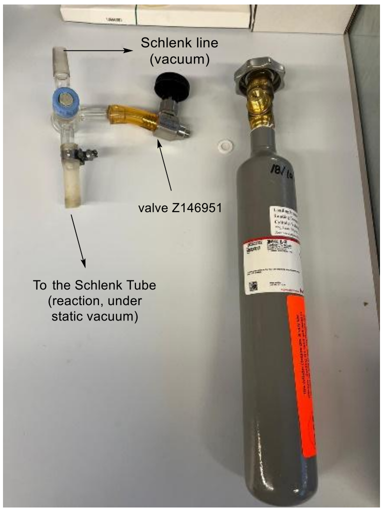

In our case we made us of the valve Z146951 with Teflon ring CGA110 Z146986. At the end of the freeze-pump-thaw, with a static vacuum maintained in Schlenk tube closing the glass stopper, the Schlenk tube was connected through a plastic tube to a three-way key system connected on the three ways to: *i*) the Schlenk tube; *ii*) the vacuum line; *iii*) the $^{13}CO_2$ cylinder. *To perform the backfilling*: 1) vacuum was stabilized and maintained in the system until the Schlenk tube stopper. 2) The three-way key was moved to connect only the cylinder and the Schlenk tube. 3) The main regulator of the cylinder was open. 4) The black valve was opened and subsequently the Schlenk tube stopper was opened to connect to the three-ways key system, thus allowing the $^{13}CO_2$ cylinder to backfill the system. 5) The Schlenk tube stopper was closed ensuring the reaction was sealed, then the main valve of the cylinder was closed, the three-ways key was moved to ventilate the system and remove the connected items.

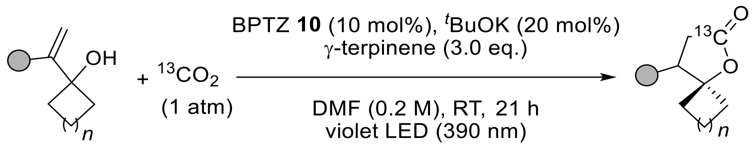

A stock solution of the catalyst mixture was prepared weighing 20 mg of 12*H*-benzo[*b*]phenothiazine and 27 mg of freshly sublimed potassium *tert*-butoxide and accurately dissolved in 4.0 mL of dry DMF (resulting molarity [12*H*-benzo[*b*]phenothiazine] = 0.02 M; ^{t}BuOK = 0.06 M). In a 10 mL Schlenk tube equipped with a stir bar, the ketone-derived tertiary alcohol (0.2 mmol, 1.0 equiv.), γ-terpinene (96 µL, 0.6 mmol, 3.0 equiv.), 1.0 mL of the catalyst stock solution was added under air. *The use of anhydrous DMF serves to maximize the reproducibility of the reaction, although a small amount of water is tolerated.* The solution was subsequently degassed *via* freeze-pump-thaw method (3 cycles) and backfilled with 1 atm of $^{13}CO_2$ (the reaction mixture turned from red to yellow after backfilling) and irradiated with visible light. *The Schlenk tube was placed in a 3D-printed carousel, resulting in a final distance of 3 cm of the glass wall from the Kessil lamp. The reaction temperature was kept constant using a fan placed on top of the reaction at 20 cm distance.*

Two general procedures were adopted for the workup of the reaction:

WORKUP A: After 21 hours, EtOAc (3 mL) was added to the Schlenk vial and moved to a separating funnel. The Schlenk vial was rinsed with EtOAc (2x3 mL). Then this organic phase was washed three times with 2 M HCl water solution saturated with NaCl (5 mL). The organic phase was collected and separated. The aqueous phase was washed once with EtOAc (3 mL) and this second organic phase washed three times with 2 M HCl water solution saturated with NaCl (3 mL). The organic phase was reunited, dried over $MgSO_4$, filtered and the solvents evaporated. The resulting crude mixture was purified by silica flash column chromatography.

WORKUP B: After 21 hours, EtOAc (3 mL) was added to the Schlenk vial and moved to a separating funnel. The Schlenk vial was rinsed with EtOAc (2x3 mL). Then this organic phase was washed three times with 2 with NaCl (5 mL). The organic phase was reunited, dried over $MgSO_4$, filtered and the solvents evaporated. The resulting crude mixture was purified by silica flash column chromatography.

For ^{13}C-NMR of the enriched molecules, showing the high peak of the labeled carbonyl carbon, traces are reported at the end of this file (assignations for ^{1}H and ^{13}C-NMR correspond to the signals of the not-labeled molecules), HRMS identification is also reported.

General Procedure L: Carboxylation of more conventional olefins

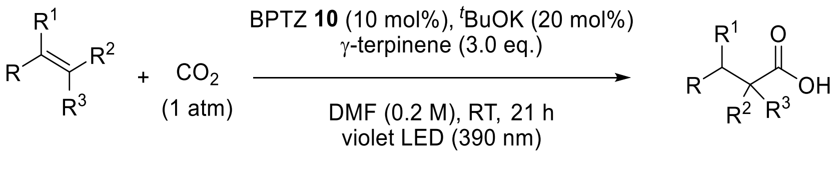

A stock solution of the catalyst mixture was prepared weighing 20 mg of 12*H*-benzo[*b*]phenothiazine and 18

mg of freshly sublimed potassium *tert*-butoxide and accurately dissolved in 4.0 mL of dry DMF (resulting molarity [12*H*-benzo[*b*]phenothiazine] = 0.02 M; ^{t}BuOK = 0.04 M). In a 10 mL Schlenk tube equipped with a stir bar, the alkene (0.2 mmol, 1.0 equiv.), γ-terpinene (96 µL, 0.6 mmol, 3.0 equiv.) and 1.0 mL of the catalyst stock solution was added under air. *The use of anhydrous DMF serves to maximize the reproducibility of the reaction, although a small amount of water is tolerated*. The solution was subsequently degassed *via* freeze-pump-thaw method (3 cycles) and backfilled with 1 atm CO_2 and irradiated with visible light. The reaction mixture turned from red to yellow after backfilling. *The Schlenk tube was placed in a 3D-printed carousel, resulting in a final distance of 3 cm of the glass wall from the Kessil lamp. The reaction temperature was kept constant using a fan placed on top of the reaction at 20 cm distance.*

After 21 hours, EtOAc (3 mL) was added to the Schlenk vial and moved to a separating funnel. The Schlenk vial was rinsed with EtOAc (2x3 mL). Then this organic phase was washed three times with 2 M HCl water solution saturated with NaCl (5 mL). The organic phase was collected and separated. The aqueous phase was washed once with EtOAc (3 mL), and this second organic phase washed three times with 2 M HCl water solution saturated with NaCl (3 mL). The organic phase was reunited, dried over $MgSO_4$, filtered and the solvents evaporated. The resulting crude mixture was purified by silica flash column chromatography.

Scaled-up Photochemical reaction

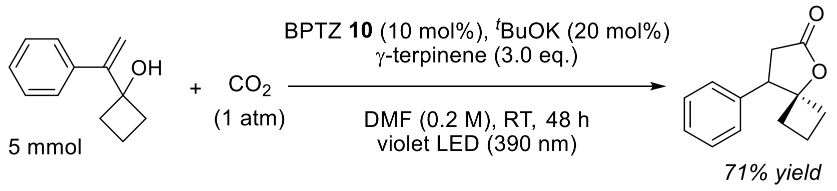

A flame-dried 200 mL Schlenk bottle (15 cm height, 4.5 cm diameter) with a stirring bar was dried under vacuum with a heat gun. 0.875 g of 1-(1-phenylvinyl)cyclobutan-1-ol (5.0 mmol), 0.115 g of *t*BuOk and 0.125 g of 12*H*-benzo[*b*]phenothiazine were weighed in three different vials. The liquid starting material was dissolved in 12 mL of dry DMF and this mixture was added to the Schlenk bottle followed by mL of γ-terpinene (2.5 mL, 15 mmol, 3.0 equiv.). Then *t*BuOK was dissolved in 13 mL of dry DMF and this mixture was added to the vial containing the 12*H*-benzo[*b*]phenothiazine photocatalyst. The resulting red solution was transferred to the Schlenk bottle. The solution was subsequently degassed *via* freeze-pump-thaw method (3 cycles) and backfilled with 1 atm CO_2 (the reaction mixture turned from red to yellow after backfilling) and irradiated with visible light for 48 hours. *The Schlenk Bottle was irradiated with 2 Kessil Lamps of 390 nm, placed at 0.5 cm distance from the glass wall of both sides of the vessel.*

After 21 hours, EtOAc (15 mL) was added to the Schlenk vial and moved to a separating funnel. The Schlenk vial was rinsed with EtOAc (2x5 mL). Then this organic phase was washed three times with 2 M HCl water solution saturated with NaCl (3x15 mL). The organic phase was collected and separated. The aqueous phase was washed once with EtOAc (15 mL), and this second organic phase washed three times with 2 M HCl water

solution saturated with NaCl (3x10 mL). The organic phase was reunited, dried over $MgSO_4$, filtered and the solvents evaporated. The resulting crude mixture was purified by silica flash column chromatography yielding compound **13** in 71% yield as a white solid (0.717 g).

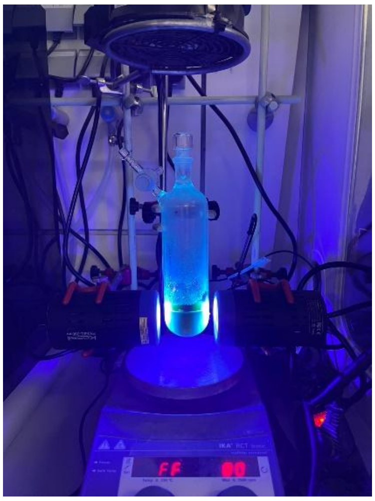

8-phenyl-5-oxaspiro[3.4]octan-6-one (13)

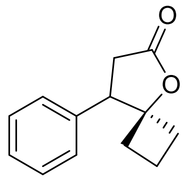

Prepared according to general procedure **H** using 1-(1-phenylvinyl)cyclobutan-1-ol (35 mg, 0.2 mmol). Pure product was obtained using workup A and flash column chromatography (99/01 to 95/05 Hexane/EtOAc) to provide the title compound as a white solid (30 mg, 75% yield). ^{1}H NMR (300 MHz, $CDCl_3$) δ 7.44 – 7.28 (m, 3H), 7.25 – 7.15 (m, 2H), 3.57 (dd, J = 8.3, 6.4 Hz, 1H), 2.93 (dd, J = 17.4, 8.3 Hz, 1H), 2.73 (dd, J = 17.4, 6.4 Hz, 1H), 2.62 – 2.46 (m, 1H), 2.39 – 2.16 (m, 2H), 2.01 – 1.70 (m, 2H), 1.40 – 1.26 (m, 1H). ^{13}C NMR (75 MHz, $CDCl_3$) δ 175.9, 138.5, 129.0, 127.9, 127.6, 89.0, 49.7, 35.6, 34.9, 30.3, 12.6. HRMS (APCI) found $[M+H]^+$ 203.1071 m/z, $C_{13}H_{15}O_2$ requires 203.1067 m/z.

4-phenyl-1-oxaspiro[4.4]nonan-2-one (17)

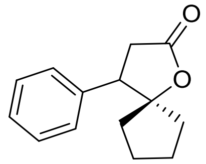

Prepared according to general procedure **H** using 1-(1-phenylvinyl)cyclopentan-1-ol (38 mg, 0.2 mmol). Pure product was obtained using workup A and flash column chromatography (99/01 to 95/5 of Hexane/EtOAc) to provide the title compound as a yellow solid (25 mg, 58% yield). ^{1}H NMR (300 MHz, $CDCl_3$) δ 7.42 – 7.25 (m, 3H), 7.25 – 7.15 (m, 2H), 3.62 (t, J = 8.4 Hz, 1H), 3.03 – 2.81 (m, 2H), 2.14 – 1.99 (m, 1H), 1.95 – 1.70 (m, 3H), 1.69 – 1.47 (m, 3H), 1.38 – 1.24 (m, 1H). ^{13}C NMR (75 MHz, $CDCl_3$) δ 176.0, 138.2, 128.9, 128.0, 127.8, 98.6, 48.9, 38.3, 36.3, 34.1, 23.5, 23.2. HRMS (APCI) $[M+H]^+$ found 217.1223, $C_{14}H_{17}O_2$ requires 217.1223

4-phenyl-1-oxaspiro[4.5]decan-2-one (18)

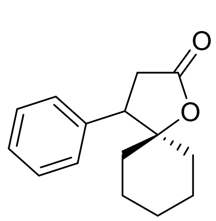

Prepared according to general procedure **I** using 1-(1-phenylvinyl)cyclohexan-1-ol (40 mg, 0.2 mmol). Pure product was obtained using workup A and flash column chromatography (99/01 to 95/05 of Hexane/EtOAc) to provide the title compound as an orange solid (26 mg, 56% yield). ^{1}H NMR (300 MHz, $CDCl_3$) δ 7.41 – 7.25 (m, 3H), 7.23 – 7.13 (m, 2H), 3.40 (t, J = 8.9 Hz, 1H), 2.94 (d, J = 8.9 Hz, 2H), 1.97 – 1.84 (m, 1H), 1.75 – 1.45 (m, 7H), 1.18 – 1.04 (m, 1H), 1.00 – 0.80 (m, 1H). ^{13}C NMR (75 MHz, $CDCl_3$) δ 175.9, 137.3, 128.8,

128.3, 127.8, 88.7, 51.4, 37.0, 34.9, 32.5, 25.1, 22.8, 21.9. HRMS (APCI) [M+H]+ found 231.1379, $C_{15}H_{19}O_2$ requires 231.1380

4-phenyl-1-oxaspiro[4.6]undecan-2-one (19)

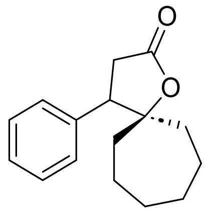

Prepared according to general procedure **I** using 1-(1-phenylvinyl)cycloheptan-1-ol (43 mg, 0.2 mmol). Pure product was obtained using workup A and flash column chromatography (99/01 Hexane/AcOEt to 95/05 Hexane/EtOAc) to provide the title compound as a yellow solid (27 mg, 56% yield). ^{1}H NMR (300 MHz, $CDCl_3$) δ 7.42 – 7.27 (m, 3H), 7.26 – 7.17 (m, 2H), 3.46 (t, J = 8.9 Hz, 1H), 3.02 – 2.80 (m, 2H), 2.17 – 1.92 (m, 2H), 1.80 – 1.24 (m, 10H). ^{13}C NMR (75 MHz, $CDCl_3$) δ 175.9, 137.2, 128.8, 128.3, 127.9, 92. 6, 51.9, 40.9, 35.3, 35.0, 29.7, 29.4, 22.9, 22.1. HRMS (APCI) [M+H]+ found 245.1535, $C_{16}H_{21}O_2$ requires 245.1536

4-phenyl-1-oxaspiro[4.7]dodecan-2-one (20)

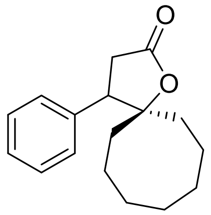

Prepared according to general procedure **I** using 1-(1-phenylvinyl)cyclooctan-1-ol (46 mg, 0.2 mmol). Pure product was obtained using workup A and flash column chromatography (99/01 to 95/05 Hexane/AcOEt) to provide the title compound as a yellow solid (28 mg, 55% yield). ^{1}H NMR (300 MHz, $CDCl_3$) δ 7.41 – 7.26 (m, 3H), 7.24 – 7.15 (m, 2H), 3.49 (t, J = 8.0 Hz, 1H), 3.01 (dd, J = 17.9, 8.8 Hz, 1H), 2.87 (dd, J = 17.9, 7.2 Hz, 1H), 2.19 – 1.92 (m, 2H), 1.86 – 1.76 (m, 1H), 1.71 – 1.53 (m, 6H), 1.49 – 1.35 (m, 5H). ^{13}C NMR (75 MHz, $CDCl_3$) δ 176.2, 138.4, 128.8, 128.3, 127.7, 92.5, 50.8, 36.0, 36.0, 31.9, 28.4, 27.3, 24.7, 22.6, 21.7. HRMS (APCI) [M+H]+ found 259.1688, $C_{17}H_{23}O_2$ requires 259.1693

2,2,8-triphenyl-5-oxaspiro[3.4]octan-6-one (21)

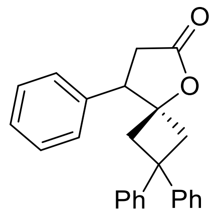

Prepared according to general procedure **H** using 3,3-diphenyl-1-(1-phenylvinyl)cyclobutan-1-ol (65 mg, 0.2 mmol). Pure product was obtained using workup A and flash column chromatography (99/01 to 95/05 of Hexane/EtOAc) to provide the title compound as a yellow solid (37 mg, 52% yield). ^{1}H NMR (300 MHz,

$CDCl_3$) δ 7.34 – 7.06 (m, 13H), 6.77 – 6.66 (m, 2H), 3.35 (dd, *J* = 12.3, 4.6 Hz, 1H), 3.29 – 3.20 (m, 2H), 3.17 – 3.09 (m, 1H), 3.09 – 2.97 (m, 1H), 2.84 (dd, *J* = 13.0, 4.6 Hz, 1H), 2.57 (dd, *J* = 17.5, 2.2 Hz, 1H). ^{13}C NMR (75 MHz, $CDCl_3$) δ 176.1, 149.6, 146.3, 139.7, 128.9, 128.6, 128.6, 127.7, 127.6, 127.0, 126.3, 126.0, 125.7, 85.0, 49.9, 48.4, 43.2, 42.8, 36.4. HRMS (APCI) [M+H]+ found 355.1697, $C_{25}H_{23}O_2$ requires 355.1693

(1R,3S,5r,7r)-3'-phenyldihydro-5'H-spiro[adamantane-2,2'-furan]-5'-one (22)

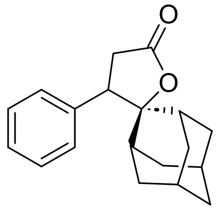

Prepared according to general procedure **H** using (1R,3S,5r,7r)-2-(1-phenylvinyl)adamantan-2-ol (51 mg, 0.2 mmol). Pure product was obtained using workup A and flash column chromatography (99/1 Hexane/AcOEt to 98/2 Hexane/AcOEt) to provide the title compound as a white solid (25 mg, 45% yield). ^{1}H NMR (300 MHz, $CDCl_3$) δ 7.37 – 7.16 (m, 5H), 3.67 (d, *J* = 8.9 Hz, 1H), 3.29 – 3.13 (m, 1H), 2.58 – 2.45 (m, 1H), 2.31 – 2.16 (m, 2H), 2.05 – 1.88 (m, 3H), 1.85 – 1.66 (m, 6H), 1.58 – 1.45 (m, 2H), 1.38 – 1.27 (m, 1H). ^{13}C NMR (75 MHz, $CDCl_3$) δ 176.8, 140.4, 128.7, 128.4, 127.4, 93.4, 46.9, 38.6, 37.5, 36.7, 35.9, 34.1, 33.1, 33.1, 32.9, 26.7, 26.6. HRMS (APCI) [M+H]+ found 283.1692, $C_{19}H_{23}O_2$ requires 283.1693

***tert*-butyl 6-oxo-8-phenyl-5-oxa-2-azaspiro[3.4]octane-2-carboxylate (23)**

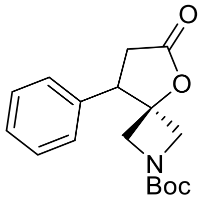

Prepared according to general procedure **H** using *tert*-butyl 3-hydroxy-3-(1-phenylvinyl)azetidine-1-carboxylate (55 mg, 0.2 mmol). The yield of the desired product (yield = (1.45/2)x100 = 72%) was obtained *via* ^{1}H NMR analysis (300 MHz, $CDCl_3$) using 0.05 mmol of 1,1,2,2-tetrachloroethane as an internal standard (title compound proved to be unstable on silica or when exposed to water, even traces).

8-phenyl-5-oxa-2-thiaspiro[3.4]octan-6-one (24)

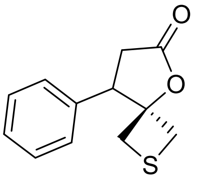

Prepared according to general procedure **H** using 3-(1-phenylvinyl)thietan-3-ol (38 mg, 0.2 mmol). Pure product was obtained using workup A and flash column chromatography (99/01 to 95/05 Hexane/EtOAc) to provide the title compound as a yellow solid (23 mg, 52% yield). ^{1}H NMR (300 MHz, $CDCl_3$) δ 7.49 – 7.32

(m, 3H), 7.32 – 7.21 (m, 2H), 4.01 (dd, *J* = 8.5, 3.3 Hz, 1H), 3.83 (d, *J* = 9.4 Hz, 1H), 3.60 (d, *J* = 9.9 Hz, 1H), 3.26 (dd, *J* = 9.4, 2.8 Hz, 1H), 3.08 (dd, *J* = 17.8, 8.6 Hz, 1H), 2.83 – 2.69 (m, 2H). ^{13}C NMR (75 MHz, $CDCl_3$) δ 174.9, 138.6, 129.3, 128.3, 127.4, 87.5, 49.3, 40.6, 35.3, 35.2. HRMS (APCI) [M+H]+ found 221.0624, $C_{12}H_{13}O_2S$ requires 221.0631

4-phenyl-1,8-dioxaspiro[4.5]decan-2-one (25)

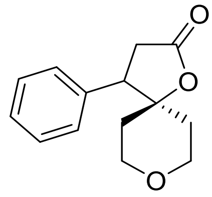

Prepared according to general procedure **I** using 4-(1-phenylvinyl)tetrahydro-2*H*-pyran-4-ol (41 mg, 0.2 mmol). Pure product was obtained using workup B and flash column chromatography (85/15 to 80/20 Hexane/EtOAc) to provide the title compound as a yellow solid (21 mg, 45% yield). ^{1}H NMR (300 MHz, $CDCl_3$) δ 7.44 – 7.28 (m, 3H), 7.25 – 7.13 (m, 2H), 3.88 – 3.64 (m, 4H), 3.43 (t, *J* = 9.0 Hz, 1H), 2.97 (d, *J* = 9.0 Hz, 2H), 2.12 – 1.96 (m, 1H), 1.88 – 1.77 (m, 1H), 1.41 – 1.30 (m, 2H). ^{13}C NMR (75 MHz, $CDCl_3$) δ 175.4, 136.4, 129.0, 128.2, 85.7, 64.3, 63.9, 51.1, 36.8, 34.5, 32.9.

4-phenyl-1-oxa-8-thiaspiro[4.5]decan-2-one (26)

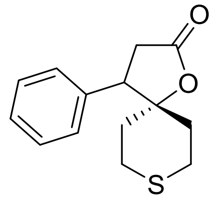

Prepared according to general procedure **I** using 4-(1-phenylvinyl)tetrahydro-2*H*-thiopyran-4-ol (44 mg, 0.2 mmol). Pure product was obtained using workup B and flash column chromatography (95/05 to 90/10 Hexane/EtOAc) to provide the title compound as a yellow solid (25 mg, 51% yield). ^{1}H NMR (300 MHz, $CDCl_3$) δ 7.43 – 7.26 (m, 3H), 7.22 – 7.13 (m, 2H), 3.40 (t, *J* = 9.2 Hz, 1H), 3.18 – 3.04 (m, 1H), 3.03 – 2.86 (m, 3H), 2.56 – 2.42 (m, 1H), 2.41 – 2.28 (m, 1H), 2.27 – 2.02 (m, 2H), 1.90 – 1.77 (m, 1H), 1.37 – 1.21 (m, 1H). ^{13}C NMR (75 MHz, $CDCl_3$) δ 175.1, 136.1, 129.0, 128.2, 128.2, 86.5, 52.0, 38.3, 34.2, 34.1, 24.9, 24.2. HRMS (APCI) [M+H]+ found 249.0941, $C_{14}H_{17}O_2S$ requires 249.0944

12-phenyl-1,4,9-trioxadispiro[4.2.48.25]tetradecan-10-one (27)

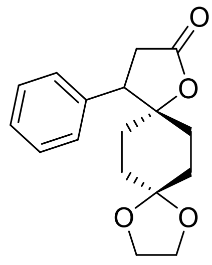

Prepared according to general procedure **I** using 8-(1-phenylvinyl)-1,4-dioxaspiro[4.5]decan-8-ol (52 mg, 0.2 mmol). Pure product was obtained using workup B and flash column chromatography (90/10 to 80/20 Hexane/EtOAc) to provide the title compound as a white solid (28 mg, 48% yield). ^{1}H NMR (300 MHz, $CDCl_3$) δ 7.43 – 7.29 (m, 3H), 7.29 – 7.15 (m, 2H), 4.01 – 3.76 (m, 4H), 3.47 (t, *J* = 8.6 Hz, 1H), 3.08 – 2.87 (m, 2H), 2.12 – 1.83 (m, 4H), 1.83 – 1.53 (m, 4H). ^{13}C NMR (75 MHz, $CDCl_3$) δ 175.7, 137.3, 128.9, 128.1, 127.9, 107.8, 87.4, 64.5, 64.3, 50.4, 35.1, 34.9, 31.3, 30.6, 30.3. HRMS (APCI) $[M+H]^+$ found 289.1434, $C_{17}H_{21}O_4$ requires 289.1434

***tert*-butyl 2-oxo-4-phenyl-1-oxa-10-azadispiro[4.1.57.15]tridecane-10-carboxylate (28)**

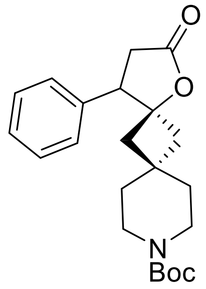

Prepared according to general procedure **H** using *tert*-butyl 2-hydroxy-2-(1-phenylvinyl)-7-azaspiro[3.5]nonane-7-carboxylate (69 mg, 0.2 mmol). Pure product was obtained using workup B and flash column chromatography (95/5 to 90/10 Hexane/EtOAc) to provide the title compound as a yellow solid (25 mg, 34% yield). ^{1}H NMR (300 MHz, $CDCl_3$) δ 7.43 – 7.25 (m, 3H), 7.20 – 7.11 (m, 2H), 3.47 (dd, *J* = 8.3, 6.0 Hz, 1H), 3.39 – 3.03 (m, 4H), 2.94 (dd, *J* = 17.5, 8.3 Hz, 1H), 2.69 (dd, *J* = 17.5, 6.0 Hz, 1H), 2.32 (d, *J* = 13.1 Hz, 1H), 2.17 (d, *J* = 13.1 Hz, 1H), 2.02 (d, *J* = 13.6 Hz, 1H), 1.82 (d, *J* = 13.6 Hz, 1H), 1.64 (t, *J* = 5.7 Hz, 2H), 1.42 (s, 9H), 1.28 – 1.15 (m, 2H). ^{13}C NMR (75 MHz, $CDCl_3$) δ 175.8, 155.0, 138.4, 129.2, 128.0, 127.7, 86.2, 79.6, 51.2, 44.4, 39.9, 37.3, 37.1, 35.8, 30.7, 28.6. HRMS (APCI) $[M+H]^+$ found 372.2165, $C_{22}H_{30}NO_4$ requires 372.2169

3-phenyl-3,4-dihydro-5*H*-spiro[furan-2,9'-xanthen]-5-one (29)

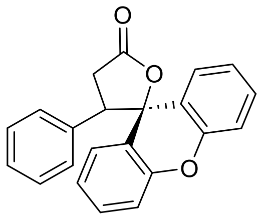

Prepared according to general procedure **H** using 9-(1-phenylvinyl)-9*H*-xanthen-9-ol (60 mg, 0.2 mmol). Pure product was obtained using workup A and flash column chromatography (95/05 to 80/20 Hexane/EtOAc) to provide the title compound as a yellow solid (45 mg, 69% yield). ^{1}H NMR (300 MHz, $CDCl_3$) δ 7.71 (dd, *J* = 7.8, 1.7 Hz, 1H), 7.45 – 7.20 (m, 4H), 7.19 – 7.05 (m, 3H), 6.99 (t, *J* = 7.5 Hz, 2H), 6.85 (d, *J* = 8.1 Hz, 1H), 6.44 (d, *J* = 7.6 Hz, 2H), 3.80 (dd, *J* = 12.3, 8.4 Hz, 1H), 3.24 (dd, *J* = 17.6, 12.3 Hz, 1H), 2.99 (dd, *J* = 17.6, 8.4 Hz, 1H). ^{13}C NMR (75 MHz, $CDCl_3$) δ 175.8, 150.9, 150.6, 133.8, 129.8, 129.7, 128.1, 128,0 127.7, 124.9,

124.7, 124.3, 123.8, 123.3, 120.2, 116.7, 116.5, 83.8, 58.3, 33.7. HRMS (APCI) found $[M+H]^+$ 329.1176 m/z, $C_{22}H_{17}O_3$ requires 329.1172 m/z.

8-(*p*-tolyl)-5-oxaspiro[3.4]octan-6-one (30)

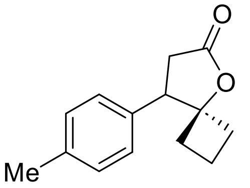

Prepared according to the general procedure **H** using 1-(1-(p-tolyl)vinyl)cyclobutanol (37.7 mg, 0.2 mmol). Pure product was obtained using workup A and flash column chromatography (100/0 to 90/10 of Hexane/EtOAc) to provide the title compound as a white solid (26.8 mg, 62%). ^1H NMR (300 MHz, $CDCl_3$) δ 7.17 (d, *J* = 8.0 Hz, 2H), 7.10 (d, *J* = 8.0 Hz, 2H), 3.52 (t, *J* = 7.3 Hz, 1H), 2.95-2.66 (m, 2H), 2.60-2.45 (m, 1H), 2.35 (s, 3H), 2.33-2.16 (m, 2H), 2.00-1.88 (m, 1H), 1.87-1.72 (m, 1H), 1.34-1.24 (m, 1H). ^{13}C NMR (75 MHz, $CDCl_3$) δ 176.0, 137.6, 135.4, 129.7, 127.5, 89.0, 49.3, 35.6, 34.8, 30.3, 21.2), 12.6. HRMS (APCI) found $[M+H]^+$ 217.1216 m/z, $C_{14}H_{17}O_2$ requires 217.1223 m/z.

8-(4-(*tert*-butyl)phenyl)-5-oxaspiro[3.4]octan-6-one (31)

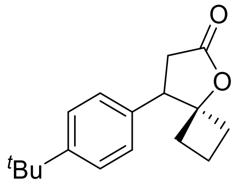

Prepared according to the general procedure **H** using 1-(1-(4-(*tert*-butyl)phenyl)vinyl)cyclobutanol (46.1 mg, 0.2 mmol). Pure product was obtained using workup A and flash column chromatography (100/0 to 90/10 of Hexane/EtOAc) to provide the title compound as a white solid (23.5 mg, 45%). ^1H NMR (300 MHz, $CDCl_3$) δ 7.36 (d, *J* = 7.6 Hz, 2H), 7.18-7.09 (d, *J* = 7.8 Hz, 2H), 3.53 (t, *J* = 7.2 Hz, 1H), 2.94-2.66 (m, 2H), 2.60-2.45 (m, 1H), 2.37-2.15 (m, 2H), 2.02-1.89 (m, 1H), 1.86-1.71 (m, 1H), 1.32 (s, 9H), 1.40-1.23 (m, 1H). ^{13}C NMR (75 MHz, $CDCl_3$) δ 176.0, 150.8, 135.3, 127.3, 125.9, 89.1, 49.2, 35.6, 34.7, 31.4, 30.3, 12.6. HRMS (APCI) found $[M+H]^+$ 259.1693 m/z, $C_{17}H_{23}O_2$ requires 259.1693 m/z.

8-(4-methoxyphenyl)-5-oxaspiro[3.4]octan-6-one (32)

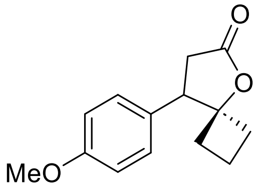

Prepared according to the general procedure **H** using 1-(1-(4-methoxyphenyl)vinyl)cyclobutanol (40.9 mg, 0.2 mmol). Pure product was obtained using workup A and flash column chromatography (100/0 to 90/10 of Hexane/EtOAc) to provide the title compound as a white solid (30.2 mg, 65%). ^1H NMR (300 MHz, $CDCl_3$)

δ 7.13 (d, J = 8.6 Hz, 2H), 6.89 (d, J = 8.6 Hz, 2H), 3.81 (s, 3H), 3.51 (t, J = 7.6 Hz, 1H), 2.94-2.63 (m, 2H), 2.58-2.46 (m, 1H), 2.34-2.15 (m, 2H), 2.00-1.88 (m, 1H), 1.84-1.73 (m, 1H), 1.38-1.21 (m, 1H). ^{13}C NMR (75 MHz, $CDCl_3$) δ 175.9, 159.3, 130.4, 128.6, 114.4, 89.1, 55.4, 49.0, 35.7, 34.7, 30.2, 12.5. HRMS (APCI) found $[M+H]^+$ 233.1172 m/z, $C_{14}H_{17}O_3$ requires 233.1171 m/z.

8-(4-fluorophenyl)-5-oxaspiro[3.4]octan-6-one (33)

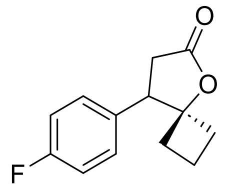

Prepared according to the general procedure **H** using 1-(1-(4-fluorophenyl)vinyl)cyclobutanol (38.4 mg, 0.2 mmol). Pure product was obtained using workup A and flash column chromatography (100/0 to 90/10 of Hexane/EtOAc) to provide the title compound as a white solid (26.9 mg, 61%). ^{1}H NMR (300 MHz, $CDCl_3$) δ 7.24-7.12 (m, 2H), 7.12-6.96 (m, 2H), 3.55 (t, J = 6.8 Hz, 1H), 3.00-2.85 (m, 1H), 2.74-2.59 (m, 1H), 2.59-2.44 (m, 1H), 2.35-2.16 (m, 2H), 1.96-1.73 (m, 2H), 1.38-1.22 (m, 1H). ^{13}C NMR (75 MHz, $CDCl_3$) δ 175.4, 160.6, 134.2, 129.0, 128.9, 116.0, 115.7, 88.7, 48.8, 35.6, 34.7, 30.1, 12.4. ^{19}F NMR (282 MHz, $CDCl_3$) δ -114.52. HRMS (APCI) found $[M+H]^+$ 221.0972 m/z, $C_{13}H_{14}FO_2$ requires 221.0972 m/z.

8-(*m*-tolyl)-5-oxaspiro[3.4]octan-6-one (34)

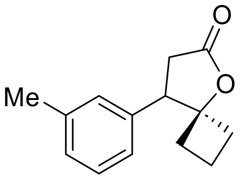

Prepared according to the general procedure **H** using 1-(1-(*m*-tolyl)vinyl)cyclobutanol (37.7 mg, 0.2 mmol). Pure product was obtained using workup A and flash column chromatography (100/0 to 90/10 of Hexane/EtOAc) to provide the title compound as a white solid (28.1 mg, 65%). ^{1}H NMR (300 MHz, $CDCl_3$) δ 7.30-7.21 (m, 1H), 7.16-7.08 (m, 1H), 7.06-6.96 (m, 2H), 3.53 (t, J = 7.0 Hz, 1H), 2.98-2.66 (m, 2H), 2.59-2.46 (m, 1H), 2.36 (s, 3H), 2.35-2.16 (m, 2H), 2.00-1.88 (m, 1H), 1.88-1.73 (m, 1H), 1.43-1.23 (m, 1H). ^{13}C NMR (75 MHz, $CDCl_3$) δ 176.0, 138.7, 138.5, 128.9, 128.6, 128.2, 124.7, 89.0, 49.6, 35.6 (CH_2), 34.9, 30.3, 21.6, 12.6. HRMS (APCI) found $[M+H]^+$ 217.1224 m/z, $C_{14}H_{17}O_2$ requires 217.1223 m/z.

8-(thiophen-3-yl)-5-oxaspiro[3.4]octan-6-one (35)

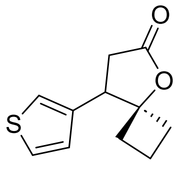

Prepared according to the general procedure **H** using 1-(1-(thiophen-3-yl)vinyl)cyclobutanol (36.1 mg, 0.2

mmol). Pure product was obtained using workup A and flash column chromatography (90/10 to 85/15 of Hexane/EtOAc) to provide the title compound as a white solid (26.9 mg, 50%). ^{1}H NMR (300 MHz, $CDCl_3$) δ 7.38-7.30 (m, 1H), 7.13-7.07 (m, 1H), 7.02-6.96 (m, 1H), 3.69 (t, *J* = 7.6 Hz, 1H), 2.95-2.83 (m, 1H), 2.82-2.61 (m, 1H), 2.59-2.46 (m, 1H), 2.38-2.16 (m, 2H), 2.12-1.91 (m, 1H), 1.90-1.74 (m, 1H), 1.44-1.31 (m, 1H). ^{13}C NMR (75 MHz, $CDCl_3$) δ 175.5, 139.1, 126.9, 126.6, 121.9, 88.6, 45.3, 35.8, 34.4, 30.2, 12.6. HRMS (APCI) found $[M+H]^+$ 209.0631 m/z, $C_{11}H_{13}O_2S$ requires 209.0631 m/z.

2,2-diphenyl-5-oxaspiro[3.4]octan-6-one (37)

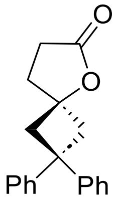

Synthesized using 3,3-diphenyl-1-(1-(phenylsulfonyl)vinyl)cyclobutan-1-ol (78 mg, 0.2 mmol), 12*H*-benzo[*b*]phenothiazine (5 mg, 0.02 mmol), potassium *tert*-butoxide (4.5 mg, 0.04 mmol), γ-terpinene (96 µL, 0.6 mmol) were dissolved in anhydrous DMF (4.0 mL) in a 10 mL Schlenk tube. Three freeze-pump-thaw cycles were performed, and CO_2 was then introduced *via* backfilling. The Schlenk tube was irradiated with 390nm light for 3 hours. The crude was purified using flash column chromatography (97/03 of hexane/EtOAc) to provide the titled compound as a pale-yellow liquid (22 mg, 42%). ^{1}H NMR (300 MHz, $CDCl_3$) δ 7.47 – 7.04 (m, 10H), 3.33 – 3.24 (m, 2H), 3.21 – 3.13 (m, 2H), 2.48 (t, *J* = 8.0 Hz, 2H), 2.03 (t, *J* = 8.0 Hz, 2H). ^{13}C NMR (75 MHz, $CDCl_3$) δ 176.3, 149.6, 146.8, 128.8, 128.6, 126.8, 126.3, 126.0, 125.7, 81.4, 47.5, 42.8, 34.5, 28.8. HRMS (APCI) found $[M+H]^+$ 279.1380, $C_{19}H_{19}O_2$ requires 279.1380.

2-((*R*)-2-(4-isobutylphenyl)propanoyl)-8-phenyl-5-oxa-2-azaspiro[3.4]octan-6-one (38)

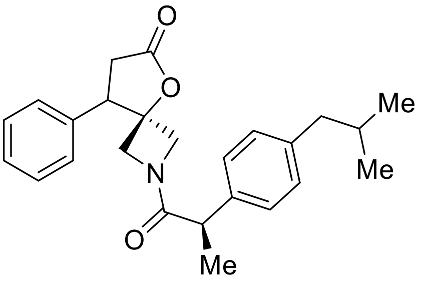

Prepared according to general procedure **H** using (*R*)-1-(3-hydroxy-3-(1-phenylvinyl)azetidin-1-yl)-2-(4-isobutylphenyl)propan-1-one (72 mg, 0.2 mmol). Pure product was obtained using workup B and flash column chromatography (50/50 of Hexane/EtOAc) to provide the title compound as a white solid (33 mg, 42% yield). ^{1}H NMR (500 MHz, $CDCl_3$, summary of rotamers and diastereomers) δ 7.45 – 7.19 (m, 4H), 7.19 – 7.01 (m, 4H), 6.98 – 6.90 (m, 1H), 4.41 – 3.19 (m, 6H), 3.10 – 2.67 (m, 2H), 2.52 – 2.40 (m, 2H), 1.93 – 1.79 (m, 1H), 1.44 – 1.32 (m, 3H), 0.94 – 0.86 (m, 6H). ^{13}C NMR (126 MHz, $CDCl_3$, summary of rotamers and diastereomers) δ 174.4, 174.3, 174.2, 174.1, 174.0, 173.9, 173.8, 173.6, 140.8, 140.7, 140.7, 140.5, 140.5, 140.4, 138.5, 138.16, 137.7, 137.7, 137.1, 136.9, 136.8, 136.4, 129.8, 129.8, 129.8, 129.7, 129.7, 129.6, 129.6,

129.6, 129.5, 129.37, 128.9, 128.9, 128.8, 128.7, 128.6, 128.5, 128.4, 128.4, 128.3, 128.2, 127.5, 127.5, 127.4, 127.3, 127.3, 127.2, 127.2, 127.1, 127.1, 127.0, 126.9, 126.9, 82.4, 82.3, 82.2, 73.0, 72.95, 72.9, 62.1, 61.9, 61.8, 61.7, 61.5, 61.1, 60.0, 59.97, 59.8, 59.7, 59.6, 59.5, 57.9, 57.7, 56.1, 55.9, 48.4, 48.3, 48.0, 47.9, 46.6, 46.4, 46.3, 46.2, 45.2, 45.17, 45.15, 42.9, 42.8, 42.7, 42.6, 42.5, 42.45, 42.43, 35.5, 35.1, 35.0, 34.9, 30.4, 30.38, 30.36, 30.32, 30.30, 30.273 29.8, 22.57, 22.56, 22.54, 22.51, 22.5, 19.5, 19.4, 19.3, 19.2, 19.1, 19.091 14.7, 14.6, 14.404 14.4. The NMR was also repeated in DMSO-d^6 at 70°C to confirm that the multitude of signals were due to the rotamers of the amide bond, observing coalescence of signals: ^{1}H NMR (500 MHz, 70°C, DMSO-d_6) δ 7.40 – 7.27 (m, 2H), 7.27 – 7.12 (m, 3H), 7.08 (d, J = 1.4 Hz, 3H), 7.04 (s, 1H), 4.24 – 4.18 (m, 1H), 3.91 – 3.84 (m, 1H), 3.81 – 3.76 (m, 1H), 3.72 – 3.67 (m, 1H), 3.66 – 3.49 (m, 1H), 2.90 – 2.81 (m, 1H), 2.48 – 2.42 (m, 2H), 1.87 (dq, J = 13.5, 6.8 Hz, 1H), 1.28 (d, J = 4.1 Hz, 1H), 1.26 – 1.17 (m, 3H), 0.89 (ddd, J = 6.5, 3.6, 2.6 Hz, 6H). ^{13}C NMR (126 MHz, 70°C, DMSO-d_6) δ 173.7, 173.8, 173.7, 172.5, 173.7, 172.5, 138.9, 128.4, 128.3, 128.2, 127.20, 127.1, 126.9, 126.6, 126.4, 125.6, 81.4, 46.1, 43.8, 40.3, 28.8, 28.8, 21.6, 21.6, 18.1. HRMS (APCI) found [M+H]$^+$ 392.2227, $C_{25}H_{30}NO_3$ requires 392.2220

2-(5-(2,5-dimethylphenoxy)-2,2-dimethylpentanoyl)-8-phenyl-5-oxa-2-azaspiro[3.4]octan-6-one (39)

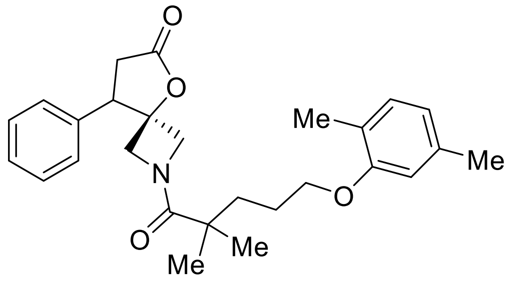

Prepared according to general procedure **H** using 5-(2,5-dimethylphenoxy)-1-(3-hydroxy-3-(1-phenylvinyl)azetidin-1-yl)-2,2-dimethylpentan-1-one (81 mg, 0.2 mmol). Pure product was obtained using workup B and flash column chromatography (50/50 of Hexane/EtOAc) to provide the title compound as a white solid (31 mg, 35% yield). ^{1}H NMR (500 MHz, $CDCl_3$) δ 7.39 – 7.29 (m, 3H), 7.20 – 7.16 (m, 2H), 7.00 (d, J = 7.5 Hz, 1H), 6.67 (d, J = 7.5 Hz, 1H), 6.61 (s, 1H), 4.54 – 4.33 (m, 2H), 4.28 – 4.14 (m, 1H), 4.01 – 3.82 (m, 3H), 3.72 (t, J = 7.8 Hz, 1H), 2.96 (dd, J = 17.6, 7.8 Hz, 1H), 2.80 (dd, J = 17.6, 7.8 Hz, 1H), 2.32 (s, 3H), 2.16 (s, 3H), 1.70 – 1.53 (m, 4H), 1.10 (s, 3H), 1.08 (s, 3H). ^{13}C NMR (126 MHz, $CDCl_3$) δ 176.9, 174.0, 157.1, 136.8, 136.7, 130.6, 129.6, 128.7, 127.2, 123.7, 121.1, 112.4, 82.6, 68.2, 48.3, 42.1, 36.7, 35.1, 25.4, 25.3, 25.2, 21.5, 15.9. HRMS (APCI) found [M+H]$^+$ 436.2466, $C_{27}H_{34}NO_4$ requires 436.2482.

2-(2-(4-(2,2-dichlorocyclopropyl)phenoxy)-2-methylpropanoyl)-8-phenyl-5-oxa-2-azaspiro[3.4]octan-6-one (40)

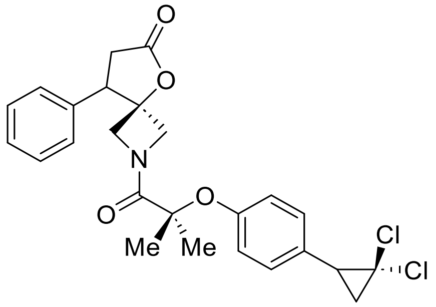

Prepared according to general procedure **I** using 2-(4-(2,2-dichlorocyclopropyl)phenoxy)-1-(3-hydroxy-3-(1-phenylvinyl)azetidin-1-yl)-2-methylpropan-1-one (89 mg, 0.2 mmol). Pure product was obtained using workup B and flash column chromatography (60/40 of Hexane/EtOAc) to provide the title compound as a yellow oil (20 mg, 20% yield). ^{1}H NMR (300 MHz, $CDCl_3$, mixture of diastereoisomers) δ 7.33 – 7.11 (m, 6H), 7.01 – 6.91 (m, 1H), 6.91 – 6.78 (m, 2H), 4.33 – 4.00 (m, 3H), 3.93 – 3.61 (m, 2H), 2.97 – 2.65 (m, 3H), 2.00 – 1.88 (m, 1H), 1.85 – 1.73 (m, 1H), 1.59 – 1.46 (m, 6H). ^{13}C NMR (75 MHz, $CDCl_3$, mixture of diastereoisomers) δ 173.4, 154.9, 140.6, 130.1, 130.1, 129.5, 128.9, 128.8, 128.6, 128.3, 127.5, 127.4, 117.5, 117.5, 117.3, 80.5, 73.3, 64.0, 63.6, 61.0, 60.5, 60.2, 46.3, 46.1, 34.9, 26.0, 25.7, 25.4, 24.4, 23.8, 14.6, 14.4. HRMS (APCI) found $[M+H]^+$ 474.1245 m/z, $C_{25}H_{26}Cl_2NO_4$ requires 474.1233 m/z.

(8*S*,9*R*,13*R*,14*R*,17*S*)-3-(methoxymethoxy)-13-methyl-3'-phenyl-3',4',6,7,8,9,11,12,13,14,15,16-dodecahydro-5'*H*-spiro[cyclopenta[a]phenanthrene-17,2'-furan]-5'-one (41)

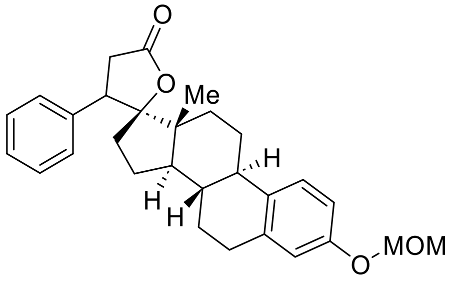

Prepared according to general procedure **H** using (8*S*,9*R*,13*R*,14*R*,17*R*)-3-(methoxymethoxy)-13-methyl-17-(1-phenylvinyl)-7,8,9,11,12,13,14,15,16,17-decahydro-6*H*-cyclopenta[a]phenanthren-17-ol (83 mg, 0.2 mmol). Pure product was obtained using workup B and flash column chromatography (95/5 Hexane/AcOEt to 85/15 Hexane/AcOEt) to provide the title compound as a white solid (27 mg, 30% yield). Only one diasteroisomer was observed. ^{1}H NMR (500 MHz, $CDCl_3$) δ 7.35 – 7.30 (m, 5H), 7.05 (d, J = 8.6 Hz, 2H), 6.76 (d, J = 9.0 Hz, 2H), 6.70 (s, 1H), 5.10 (s, 2H), 3.81 (t, J = 8.9 Hz, 1H), 3.44 (s, 3H), 3.12 – 3.03 (m, 2H), 2.93 – 2.84 (m, 3H), 2.79 – 2.72 (m, 3H), 2.53 – 2.44 (m, 2H), 2.32 – 2.27 (m, 2H), 2.06 – 2.00 (m, 2H), 1.84 – 1.72 (m, 4H), 1.52 – 1.45 (m, 7H), 0.97 (s, 3H). ^{13}C NMR (126 MHz, $CDCl_3$) δ 175.9, 155.2, 137.9, 137.8, 133.3, 128.7, 128.4, 127.9, 126.4, 116.4, 113.9, 98.8, 94.6, 56.0, 53.0, 48.4, 48.3, 43.3, 39.1, 36.8, 35.8, 31.8, 29.7, 27.3, 26.1, 23.1, 16.6. HRMS (APCI) found $[M+H]^+$ found 447.2517 $C_{29}H_{35}O_4$ requires 447.2530.

4-phenyl-1-oxaspiro[5.7]tridecan-2-one (42)

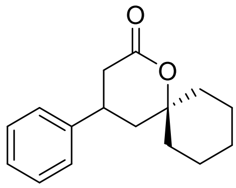

Prepared according to general procedure **J** using 1-(2-phenylallyl)cyclohexan-1-ol (43 mg, 0.2 mmol). The crude product was purified by flash column chromatography (98/02 to 950/5 Hexane/EtOAc) to provide the title compound as a white solid (32 mg, 65% yield). ^{1}H NMR (300 MHz, $CDCl_3$): δ 7.42-7.33 (m, 2H), 7.32-7.27 (m, 1H), 7.26 – 7.20 (m, 2H), 3.29 (tt, *J* = 12.5, 5.5 1H), 2.89 (ddm, *J* = 17.8, 5.5 1H), 2.52 (dd, *J* = 17.8, 12.5 Hz, 1H), 2.21-2.11 (m, 1H), 2.09-1.97 (m, 1H), 1.94 – 1.70 (m, 4H), 1.70 – 1.48 (m, 5H), 1.46 – 1.31 (m, 1H). ^{13}C NMR (75 MHz, $CDCl_3$) δ 170.9, 142.9, 129.1, 127.3, 126.7, 83.0, 40.8, 39.7, 37.7, 36.3, 34.2, 25.4, 22.1, 21.8. HRMS (APCI) $[M+H]^+$ found 245.1536, $C_{16}H_{21}O_2$ requires 245.1536

8-phenyl-5-oxaspiro[3.5]nonan-6-one (43)

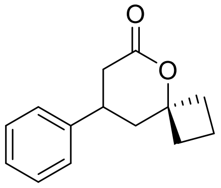

Prepared according to general procedure **J** using 1-(2-phenylallyl)cyclobutan-1-ol (38 mg, 0.2 mmol). The crude product was purified by flash column chromatography (98/02 to 95/05 Hexane/EtOAc) to provide the title compound as a colorless oil (15 mg, 35% yield). ^{1}H NMR (300 MHz, $CDCl_3$) δ 7.43 – 7.32 (m, 2H), 7.32 – 7.26 (m, 1H), 7.26 – 7.17 (m, 2H), 3.17 (tdd, *J* = 11.8, 5.8, 3.7 Hz, 1H), 2.83 (ddd, *J* = 17.7, 5.8, 1.9 Hz, 1H), 2.62 – 2.40 (m, 3H), 2.40 – 2.31 (m, 1H), 2.31 – 2.09 (m, 2H), 2.05 – 1.83 (m, 2H), 1.75 – 1.53 (m, 1H). ^{13}C NMR (75 MHz, $CDCl_3$) δ 170.4, 142.8, 129.0, 127.2, 126.6, 83.1, 38.7, 37.6, 35.5, 34.8, 34.6, 12.5. HRMS (APCI) $[M+H]^+$ found 217.1223, $C_{14}H_{17}O_2$ requires 217.1223

9-phenyl-6-oxaspiro[4.5]decan-7-one (44)

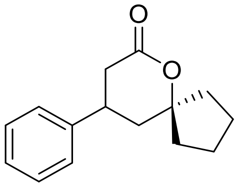

Prepared according to general procedure **J** using 1-(2-phenylallyl)cyclopentan-1-ol (40 mg, 0.2 mmol). The crude product was purified by flash column chromatography (98/02 to 95/05 Hexane/EtOAc) to provide the title compound as a colorless oil (15 mg, 32% yield). ^{1}H NMR (300 MHz, $CDCl_3$) δ δ 7.42 – 7.33 (m, 2H), 7.32 – 7.17 (m, 3H), 3.27 (tdd, *J* = 12.1, 5.7, 3.9 Hz, 1H), 2.92 (ddd, *J* = 17.9, 5.7, 1.9 Hz, 1H), 2.54 (dd, *J* = 17.9, 12.1 Hz, 1H), 2.26 – 2.09 (m, 2H), 2.10 – 1.90 (m, 3H), 1.83 – 1.65 (m, 4H). ^{13}C NMR (75 MHz, $CDCl_3$)

δ 170.9, 143.0, 129.1, 127.3, 126.6, 92.8, 40.8, 40.4, 39.3, 37.6, 36.2, 24.2, 23.7. HRMS (APCI) $[M+H]^+$ found 231.1366, $C_{15}H_{19}O_2$ requires 231.1379

4-phenyl-1-oxaspiro[5.6]dodecan-2-one (45)

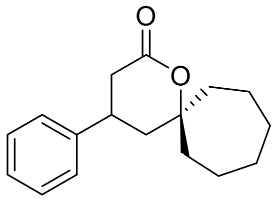

Prepared according to general procedure **J** using 1-(2-phenylallyl)cycloheptan-1-ol (46 mg, 0.2 mmol). The crude product was purified by flash column chromatography (98/02 to 95/05 of Hexane/EtOAc) to provide the title compound as a white solid (24 mg, 47% yield). ^{1}H NMR (300 MHz, $CDCl_3$) δ 7.41-7.30 (m, 2H), 7.33-7.28 (m, 1H), 7.27-7.20 (m 2H), 3.26 (tm, *J* = 12.4 Hz, 1H), 2.89 (ddm, *J* = 17.8, 1H), 2.50 (dd, *J* = 17.8, 12.4 Hz, 1H), 2.26-2.12 (m, 1H), 2.11-1.91 (m, 3H), 1.88-1.58 (m, 7H), 1.57-1.32 (m, 3H). ^{13}C NMR (75 MHz, $CDCl_3$) δ 171.0, 142.9, 129.1, 127.3, 126.7, 87.6, 43.6, 41.6, 39.1, 37.7, 34.6, 30.0, 29.8, 22.6, 22.2. HRMS (APCI) $[M+H]^+$ found 259.1693, $C_{17}H_{23}O_2$ requires 259.1687

4-phenyl-1-oxaspiro[5.7]tridecan-2-one (46)

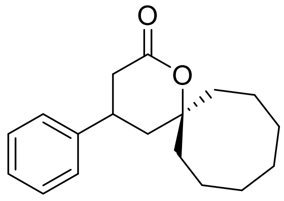

Prepared according to general procedure **J** using 1-(2-phenylallyl)cyclooctan-1-ol (49 mg, 0.2 mmol). The crude product was purified by flash column chromatography (98/02 to 95/05 Hexane/EtOAc) to provide the title compound as a white solid (25 mg, 46% yield). ^{1}H NMR (300 MHz, $CDCl_3$) δ 7.39 (m, 2H), 7.31 (m, 1H), 7.25 (m, 2H), 3.27 (tm, *J* = 12.6 Hz, 1H), 2.90 (dm, *J* = 17.8 Hz, 1H), 2.52 (dd, *J* = 17.8, 12.6 Hz, 1H), 2.32 – 2.09 (m, 3H), 1.87-1.66 (m, 9H), 1.53 – 1.35 (m, 4H). ^{13}C NMR (75 MHz, $CDCl_3$) δ 170.9, 142.9, 129.1, 127.4, 126.7, 87.2, 40.1, 39.2, 37.6, 34.6, 33.8, 28.6, 27.7, 25.3, 22.5, 21.9. HRMS (APCI) $[M+H]^+$ found 273.1849, $C_{18}H_{25}O_2$ requires 273.1843

4-phenyl-1-oxaspiro[5.7]tridecan-2-one (47)

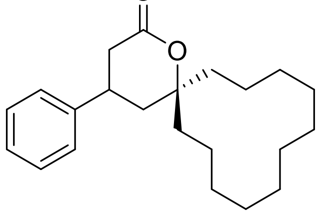

Prepared according to general procedure **J** using 1-(2-phenylallyl)cyclododecan-1-ol (60 mg, 0.2 mmol). The

crude product was purified by flash column chromatography (98/2 to 90/10 of Hexane/EtOAc) to provide the title compound as a white solid (38 mg, 58% yield). ^{1}H NMR (300 MHz, $CDCl_3$) 7.43 – 7.32 (m, 2H), 7.32 – 7.27 (m, 1H), 7.27 – 7.18 (m, 2H), 3.27 (tm, *J* = 12.6 Hz, 1H), 2.89 (dm, *J* = 17.8, 1H), 2.52 (dd, *J* = 17.8, 12.2 Hz, 1H), 2.18 – 1.95 (m, 2H), 1.94 – 1.69 (m, 2H), 1.69 – 1.60 (m, 2H), 1.56 – 1.46 (m, 4H), 1.39 (apparent br s, 14H). ^{13}C NMR (75 MHz, $CDCl_3$) δ 171.0, 129.1, 127.3, 126.7, 86.9, 40.0, 37.6, 36.8, 34.6, 32.6, 26.4, 26.3, 26.1, 22.7, 22.6, 22.2, 22.2, 19.9, 19.1. HRMS (APCI) $[M+H]^+$ found 329.2475, $C_{22}H_{33}O_2$ requires 329.2465

4'-phenyldihydrospiro[adamantane-2,2'-pyran]-6'(3'*H*)-one (48)

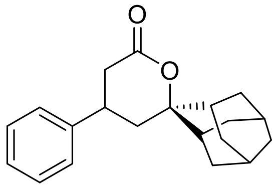

Prepared according to general procedure **J** using 2-(2-phenylallyl)adamantan-2-ol (54 mg, 0.2 mmol). The crude product was purified by flash column chromatography (98/2 to 90/10 Hexane/EtOAc) to provide the title compound as a white solid (26 mg, 44% yield). ^{1}H NMR (300 MHz, $CDCl_3$) δ 7.42 – 7.32 (m, 2H), 7.32 – 7.27 (m, 1H), 7.25 – 7.18 (m, 2H), 3.28 – 3.12 (m, 1H), 2.88 (dd, *J* = 17.9, 5.9, Hz, 1H), 2.71 (dm, *J* = 14.2, Hz, 1H), 2.57 (d, *J* = 11.8 Hz, 1H), 2.50 (br d, *J* = 11.8 Hz, 1H), 2.35 (br d, *J* = 12.8 Hz, 1H), 2.19 (vr s, 1H), 1.95 – 1.72 (m, 7H), 1.74 (apparent s, 1H), 1.72 – 1.51 (m, 4H). ^{13}C NMR (75 MHz, $CDCl_3$) δ 129.1, 127.3, 126.7, 87.1, 40.0, 38.2, 38.0, 37.4, 35.7, 34.9, 34.4, 34.0, 32.6, 32.5, 27.3, 27.1. HRMS (APCI) $[M+H]^+$ found 297.1849, $C_{20}H_{25}O_2$ requires 297.1844

12-phenyl-1,4,9-trioxadispiro[4.2.5^8.2^5]pentadecan-10-one (49)

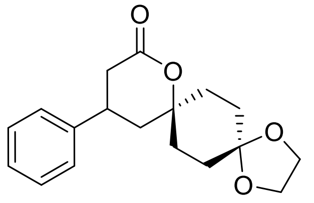

Prepared according to general procedure **J** using 8-(2-phenylallyl)-1,4-dioxaspiro[4.5]decan-8-ol (55 mg, 0.2 mmol). The crude product was purified by flash column chromatography (85/15 to 70/30 of Hexane/EtOAc) to provide the title compound as a white solid (27 mg, 45% yield). ^{1}H NMR (300 MHz, $CDCl_3$) δ 7.41 – 7.28 (m, 2H), 7.23 – 7.15 (m, 2H), 3.94 (dm, *J* = 5.9 Hz, 4H), 3.35 – 3.20 (m, 1H), 2.95 – 2.83 (m, 1H), 2.52 (dd, *J* = 17.8, 5.9 Hz, 1H), 2.19 – 1.97 (m, 5H), 1.94 – 1.73 (m, 4H), 1.69 – 1.61 (m, 2H). ^{13}C NMR (75 MHz, $CDCl_3$) δ 170.2, 142.3, 129.2, 127.5, 126.7, 81.4, 63.5, 63.2, 41.8, 39.1, 37.7, 37.1, 33.7, 31.6, 30.4, 29.9. HRMS (APCI) $[M+H]^+$ found 303.1585 $C_{18}H_{23}O_4$ requires 303.1591

9,9-difluoro-4-phenyl-1-oxaspiro[5.5]undecan-2-one (50)

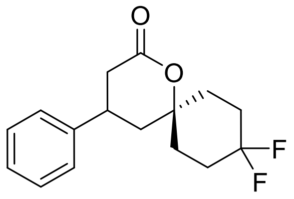

Prepared according to general procedure **J** using 4,4-difluoro-1-(2-phenylallyl)cyclohexan-1-ol (50 mg, 0.2 mmol). The crude product was purified by flash column chromatography (95/05 to 90/10 Hexane/EtOAc) to provide the title compound as a colorless oil (33 mg, 55% yield). ^{1}H NMR (300 MHz, $CDCl_3$) δ 7.44 – 7.27 (m, 3H), 7.25 – 7.15 (m, 2H), 3.29 (tt, *J* = 12.5, 4.7 Hz, 1H), 2.91 (ddd, *J* = 17.9, 5.4, 2.1 Hz, 1H), 2.54 (dd, *J* = 17.9, 12.5 Hz, 1H), 2.42 – 2.12 (m, 3H), 2.06 – 1.84 (m, 6H), 1.82 – 1.70 (m, 1H). ^{13}C NMR (75 MHz, $CDCl_3$) δ 170.0, 142.1, 129.2, 127.6, 126.6, 123.0 (app t, *J* = 241.6 Hz), 80.3 (d, *J* = 1.4 Hz), 41.0 (d, *J* = 2.3 Hz), 37.6, 35.9 (d, *J* = 9.5 Hz), 34.6, 33.2 (d, *J* = 9.5 Hz), 29.3 (app td, *J* = 24.9, 10.7 Hz). ^{19}F NMR (282 MHz, $CDCl_3$) δ -93.60 (d, *J* = 237.1 Hz), -103.79 (dt, *J* = 236.9, 34.0 Hz). HRMS (APCI) $[M+H]^+$ found 281.1344 $C_{16}H_{19}F_2O_2$ requires 281.1348.

4-phenyl-1-oxaspiro[5.5]undecane-2,9-dione (51)

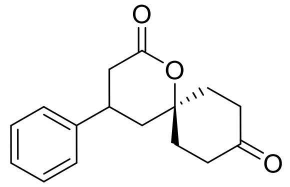

Prepared according to general procedure **J** using 4-hydroxy-4-(2-phenylallyl)cyclohexan-1-one (46 mg, 0.2 mmol). The crude product was purified by flash column chromatography (85/15 to 70/30 Hexane/EtOAc) to provide the title compound as a colorless oil (16 mg, 31% yield). ^{1}H NMR (300 MHz, $CDCl_3$) δ 7.41 – 7.33 (m, 2H), 7.33 – 7.28 (m, 1H), 7.24 – 7.18 (m, 2H), 3.32 (tt, *J* = 12.4, 4.9 Hz, 1H), 3.03 – 2.87 (m, 2H), 2.87 – 2.76 (m, 1H), 2.59 (dd, *J* = 17.7, 12.5 Hz, 1H), 2.57 – 2.44 (m, 1H), 2.42 – 2.30 (m, 2H), 2.30 – 2.18 (m, 1H), 2.14 – 2.07 (m, 2H), 2.04 – 2.01 (m, 1H), 1.91 (dt, *J* = 13.7, 4.7 1H). ^{13}C-NMR (75 MHz, $CDCl_3$) δ 209.8, 170.1, 142.0, 129.2, 127.7, 126.6, 80.4, 40.8, 39.0, 37.7, 36.6, 36.5, 36.2, 34.9. HRMS (APCI) $[M+H]^+$ found 259.1320, $C_{16}H_{19}O_3$ requires 259.1329

9,9-difluoro-4-phenyl-1-oxaspiro[5.5]undecan-2-one (52)

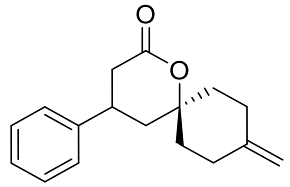

Prepared according to general procedure **J** using4-methylene-1-(2-phenylallyl)cyclohexan-1-ol (46 mg, 0.2 mmol). The crude product was purified by flash column chromatography (95/05 to 90/10 Hexane/EtOAc) to

provide the title compound as a colorless oil (22 mg, 42% yield). ^{1}H NMR (300 MHz, $CDCl_3$) δ 7.42 – 7.33 (m, 2H), 7.33 – 7.27 (m, 1H), 7.26 – 7.19 (m, 2H), 4.72 (s, 2H), 3.31 (tt, *J* = 12.6, 4.5 Hz, 1H), 2.91 (dd, *J* = 17.6, 5.5 Hz, 1H), 2.70 – 2.45 (m, 3H), 2.27 – 2.15 (m, 3H), 2.14 – 2.05 (m, 1H), 2.05 – 1.95 (m, 1H), 1.88 (t, *J* = 13.5 Hz, 1H), 1.78 – 1.58 (m, 2H). ^{13}C NMR (75 MHz, $CDCl_3$) δ 170.7, 147.0, 142.7, 129.1, 127.4, 126.7, 108.3, 82.1, 41.0, 40.6, 37.7, 37.5, 34.4, 30.1, 30.0. HRMS (APCI) [M+H]$^+$ found 257.1563 $C_{17}H_{21}O_2$ requires 257.1536

4-phenyl-1,9-dioxaspiro[5.5]undecan-2-one (53)

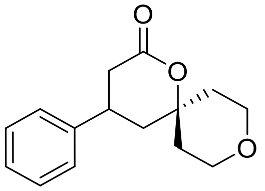

Prepared according to general procedure **J** using 4-(2-phenylallyl)tetrahydro-*2H*-pyran-4-ol (43 mg, 0.2 mmol). The crude product was purified by flash column chromatography (95/05 to 80/20 Hexane/EtOAc) to provide the title compound as a colorless oil (30 mg, 60% yield). ^{1}H NMR (300 MHz, $CDCl_3$) δ 7.33 – 7.22 (m, 2H), 7.22 – 7.17 (m, 1H), 7.14 – 7.08 (m, 2H), 3.93 – 3.62 (m, 4H), 3.21 (tm, *J* = 12.5 Hz, 1H), 2.81 (dd, *J* = 17.8, 4.4 Hz, 1H), 2.44 (dd, *J* = 17.8, 12.4 Hz, 1H), 2.06 – 1.91 (m, 2H), 1.88 – 1.60 (m, 4H). ^{13}C NMR (75 MHz, $CDCl_3$) δ 170.2, 142.3, 129.1, 127.5, 126.6, 79.6, 63.5, 63.2, 41.7, 39.1, 37.7, 37.0, 33.7. HRMS (APCI) [M+H]$^+$ found 247.1321 $C_{15}H_{19}O_3$ requires 247.1329

4-phenyl-1-oxa-9-thiaspiro[5.5]undecan-2-one (54)

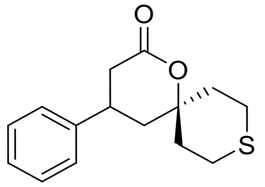

Prepared according to general procedure **J** using 4-(2-phenylallyl)tetrahydro-*2H*-thiopyran-4-ol (47 mg, 0.2 mmol). The crude product was purified by flash column chromatography (95/05 to 80/20 Hexane/EtOAc) to provide the title compound as a colorless oil (25 mg, 47% yield). ^{1}H NMR (300 MHz, $CDCl_3$) δ 7.40 – 7.32 (m, 2H), 7.32 – 7.27 (m, 1H), 7.23 – 7.16 (m, 2H), 3.36 – 3.23 (m, 1H), 3.23 – 2.97 (m, 2H), 2.94 – 2.81 (m, 1H), 2.60 – 2.35 (m, 4H), 2.19 – 2.05 (m, 2H), 2.02 – 1.74 (m, 3H). ^{13}C NMR (75 MHz, $CDCl_3$) δ 170.2, 142.3, 129.2, 127.6, 126.7, 80.7, 42.0, 40.7, 37.9, 37.6, 33.8, 24.0, 23.7. HRMS (APCI) [M+H]$^+$ found 263.1095, $C_{15}H_{19}O_2S$ requires 263.1100

tert-butyl 2-oxo-4-phenyl-1-oxa-9-azaspiro[5.5]undecane-9-carboxylate (55)

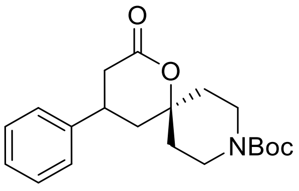

Prepared according to general procedure **J** using *tert*-butyl 4-hydroxy-4-(2-phenylallyl)piperidine-1-carboxylate (62 mg, 0.2 mmol) and performing the workup with NaCl instead of the acidic solution saturated with NaCl. The crude product was purified by flash column chromatography (80/20 to 70/30 Hexane/EtOAc) to provide the title compound as a colorless oil (33 mg, 48% yield). ^{1}H NMR (500 MHz, $CDCl_3$) 7.39-7.33 (m, 2H), 7.32 – 7.26 (m, 1H), 7.23 – 7.17 (m, 2H), 3.88 (s, 2H), 3.37 (s, 1H), 3.34 – 3.23 (m, 2H), 2.90 (ddd, *J* = 17.9, 5.4, 2.1 Hz, 1H), 2.54 (dd, *J* = 17.9, 12.3 Hz, 1H), 2.08 – 2.01 (m, 2H), 1.90 (t, *J* = 13.5 Hz, 1H), 1.85 – 1.78 (m, 1H), 1.76 – 1.53 (m, 2H), 1.47 (s, 9H). ^{13}C NMR (125 MHz, $CDCl_3$) δ 170.2, 154.8, 142.3, 129.2, 127.5, 126.6, 80.5, 79.9, 41.5, 37.7, 34.0, 28.6.

4'-(4-fluorophenyl)dihydrospiro[adamantane-2,2'-pyran]-6'(3'H)-one (56)

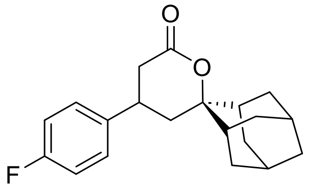

Prepared according to the general procedure **J** using 2-(2-(4-fluorophenyl)allyl)adamantan-2-ol (57.3 mg, 0.2 mmol). The crude was purified by flash column chromatography (100/0 to 90/10 of Hexane/EtOAc) to provide the title compound as a white solid (25.8 mg, 41%). ^{1}H NMR (300 MHz, $CDCl_3$) δ 7.24-7.11 (m, 2H), 7.10-6.99 (m, 2H), 3.27-3.10 (m, 1H), 2.93-2.80 (m, 1H), 2.73-2.64 (m, 1H), 2.53-2.41 (m, 2H), 2.40-2.30 (m, 1H), 2.21-2.14 (m, 1H), 1.97-1.76 (m, 8H), 1.76-1.71 (m, 2H), 1.66-1.61 (m, 2H). ^{13}C NMR (75 MHz, $CDCl_3$) δ 170.7, 160.2, 139.0, 128.1, 128.0, 115.9, 115.6, 86.9, 39.8, 38.1, 37.9), 37.4, 35.6, 34.8, 34.3, 33.2, 32.4, 32.3, 27.1, 26.9. ^{19}F NMR (282 MHz, $CDCl_3$) δ -115.7. HRMS (APCI) found $[M+H]^+$ 315.1757 m/z, $C_{24}H_{24}FO_2$ requires 315.1755 m/z.

4-(4-fluorophenyl)-1-oxaspiro[5.5]undecan-2-one (57)

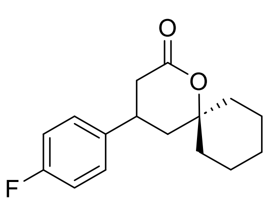

Prepared according to the general procedure **J** using 1-(2-(4-fluorophenyl)allyl)cyclohexanol (46.9 mg, 0.2 mmol), The crude was purified by flash column chromatography (100/0 to 90/10 of Hexane/EtOAc) to provide the title compound as a white solid (19.4 mg, 37%). ^{1}H NMR (300 MHz, $CDCl_3$) δ 7.23-7.10 (m, 2H), 7.09-

6.96 (m, 2H), 3.33-3.14 (m, 1H), 2.92-2.79 (m, 1H), 2.44 (dd, J = 18.0, 12.2 Hz, 1H), 2.18-2.06 (m, 1H), 2.05-1.95 (m, 1H), 1.88-1.68 (m, 4H), 1.68-1.55 (m, 5H), 1.43-1.31 (m, 1H). ^{13}C NMR (75 MHz, $CDCl_3$) δ 170.7, 160.3, 138.6, 128.2, 128.1, 116.1, 115.8, 82.9, 41.0, 39.7, 37.9, 36.4, 33.6, 25.5, 22.0, 21.7.^{19}F NMR (282 MHz, $CDCl_3$) δ -115.4. HRMS (APCI) found $[M+H]^+$ 263.1441 m/z, $C_{16}H_{20}FO_2$ requires 263.1442 m/z.

4-phenyloctahydro-2H-chromen-2-one (58)

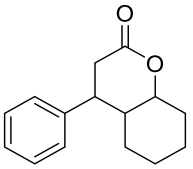

Prepared according to general procedure **H** using 2-(1-phenylvinyl)cyclohexan-1-ol (40.5 mg, 0.2 mmol). Pure product was obtained using workup A and flash column chromatography (90/10 Hexane/EtOAc to 70/30 Hexane/EtOAc) to provide the title compound as a colorless oil (23.5 mg, 51% yield of 60:40 *d r* mixture). ^{1}H NMR (300 MHz, $CDCl_3$) δ 7.50 – 7.23 (m, 3H), 7.21 – 7.04 (m, 2H), 4.21 – 3.90 (m, 1H), 3.23 (s, 0.5 H), 3.09 – 2.90 (m, 1.5H), 2.91 – 2.77 (m, 0.5H), 2.75 – 2.52 (m, 0.5H), 2.33 – 2.10 (m, 1H), 1.87 (m, 2H), 1.83 – 1.59 (m, 2.5H), 1.58-1.42 (m, 1H), 1.41 – 1.17 (m, 1H), 1.17 – 1.00 (m, 0.5H), 1.02 – 0.86 (m, 0.5H), 0.86 – 0.62 (m, 0.5H). ^{13}C NMR (75 MHz, $CDCl_3$) δ 171.3, 171.1, 142.2, 139.9, 129.07, 129.08, 128.6, 128.5, 127.4, 127.3, 83.2, 78.6, 45.3, 44.8, 41.4, 38.3, 36.9, 32.9, 32.6, 29.1, 29.0, 25.4, 25.2, 24.5, 23.9. HRMS (APCI) $[M+H]^+$ found 231.1386 $C_{15}H_{19}O_2$ requires 231.1380

6,6-dimethyl-4-phenyltetrahydro-*2H*-pyran-2-oneone (59)

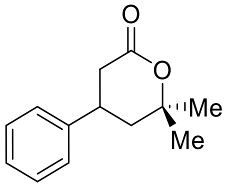

Prepared according to general procedure **J** using 2-methyl-4-phenylpent-4-en-2-ol (35 mg, 0.2 mmol). The crude product was purified by flash column chromatography (95/05 to 90/10 Hexane/EtOAc) to provide the title compound as a colorless oil (14 mg, 35% yield). ^{1}H NMR (300 MHz, $CDCl_3$) δ 7.37 (dd, J = 8.2, 6.7 Hz, 2H), 7.32 – 7.18 (m, 3H), 3.29 (tt, J = 12.3, 4.5 Hz, 1H), 2.89 (ddd, J = 17.8, 5.4, 2.0 Hz, 1H), 2.50 (dd, J = 17.8, 12.3 Hz, 1H), 2.06 (m, H), 1.93 (t, J = 13.5 Hz, 1H), 1.52 (s, 6H). ^{13}C NMR (75 MHz, $CDCl_3$) δ 170.8, 142.7, 129.1, 127.4, 126.7, 82.0, 42.1, 37.3, 35.0, 31.0, 27.7. HRMS (APCI) $[M+H]^+$ found 205.1222 $C_{13}H_{17}O_2$ requires 205.1223

6,6-diethyl-4-phenyltetrahydro-*2H*-pyran-2-on (60)

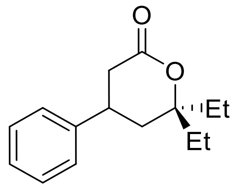

Prepared according to general procedure **J** 3-methyl-5-phenylpent-4-en-2-ol (41 mg, 0.2 mmol). The crude product was purified by flash column chromatography (95/5 to 90/10 Hexane/EtOAc) to provide the title compound as a colorless oil (18 mg, 38% yield). ^{1}H NMR (300 MHz, $CDCl_3$) δ 7.47-7.39 (m, 2H), 7.41 – 7.25 (m, 3H), 5.38 (s, 1H), 5.18 (s, 1H), 2.77 – 2.70 (m, 2H), 1.43 (q, J = 7.5 Hz, 4H), 1.31 (s, 1H), 0.83 (d, J = 7.5 Hz, 6H). ^{13}C NMR (75 MHz, $CDCl_3$) δ 146.0, 143.0, 128.5, 127.6, 126.7 117.6, 74.9, 44.2, 31.0, 8.1. HRMS (APCI) $[M+H]^+$ found 233.1527, $C_{15}H_{21}O_2$ requires 233.1536

4-phenyl-6,6-dipropyltetrahydro-2*H*-pyran-2-one (61)

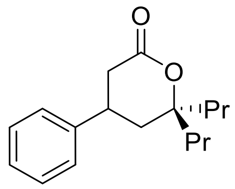

Prepared according to general procedure **J** using 2-phenyl-4-propylhept-1-en-4-o (47 mg, 0.2 mmol). The crude product was purified by flash column chromatography (95/05 to 90/10 of Hexane/EtOAc) to provide the title compound as a colorless oil (23 mg, 38% yield). ^{1}H NMR (300 MHz, $CDCl_3$) δ 7.42 – 7.33 (m, 2H), 7.32-7.26 (m, 1H), 7.25-7.22 (m, 2H), 3.25 (tt, J = 12.6, 4.5 Hz, 1H), 2.87 (ddd, J = 17.8, 5.1, 2.2 Hz, 1H), 2.48 (dd, J = 17.8, 12.6 Hz, 1H), 1.94-1.64 (m, 5H), 1.50-1.37 (m, 4H), 0.97 (q, J = 17.8 Hz, 6H). ^{13}C NMR (75 MHz, $CDCl_3$) δ 171.1, 142.7, 128.9, 127.2, 126.5, 86.1, 42.4, 40.9, 38.2, 37.3, 34.4, 17.2, 16.4, 14.5, 14.3. HRMS (APCI) $[M+H]^+$ found 261.1845 $C_{17}H_{25}O_2$ 261.1849

8-phenyl-5-oxaspiro[3.4]octan-6-one-6-^{13}C (62)

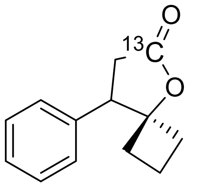

Prepared according to general procedure **K** using 1-(1-phenylvinyl)cyclobutan-1-ol (35 mg, 0.2 mmol). Pure product was obtained using workup A and flash column chromatography (99/01 to 95/05 Hexane/EtOAc) to provide the title compound as a white solid (32 mg, 75% yield). HRMS (APCI) $[M+H]^+$ found 204.1106 $C_{12}H_{15}O_2{}^{13}C$ requires 204.1100

4-phenyl-1-oxaspiro[5.5]undecan-2-one-3-^{13}C (63)

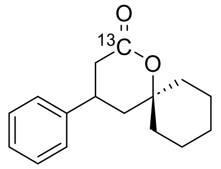

Prepared according to general procedure **K** using 1-(2-phenylallyl)cyclohexan-1-ol (43 mg, 0.2 mmol). Pure product was obtained using workup A and flash column chromatography (98/02 to 95/05 Hexane/EtOAc) to provide the title compound as a white solid (34 mg, 65% yield). $[M+H]^+$ found 245.1567, $C_{15}H_{21}O_2{}^{13}C$ requires 245.1570

4-phenyloctahydro-2*H*-chromen-2-one-2-^{13}C (64)

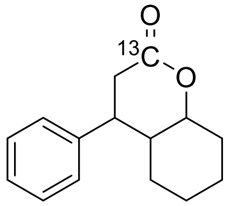

Prepared according to general procedure **K** using 2-(1-phenylvinyl)cyclohexan-1-ol (40.5 mg, 0.2 mmol). Pure product was obtained using workup A and flash column chromatography (90/10 to 70/30 Hexane/EtOAc) to provide the title compound as a colorless oil (25 mg, 51% yield of 60:40 *d r* mixture)

2-((*R*)-2-(4-isobutylphenyl)propanoyl)-8-phenyl-5-oxa-2-azaspiro[3.4]octan-6-one-6-^{13}C (65)

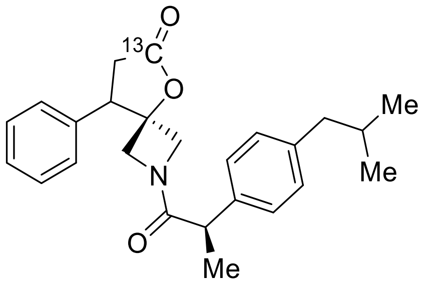

Prepared according to general procedure **K** using (*R*)-1-(3-hydroxy-3-(1-phenylvinyl)azetidin-1-yl)-2-(4-isobutylphenyl)propan-1-one (72 mg, 0.2 mmol). Pure product was obtained using workup B and flash column chromatography (50/50 of Hexane/EtOAc) to provide the title compound as a white solid (36 mg, 42% yield). $[M+H]^+$ found 393.2264 1 $C_{24}H_{30}NO_3{}^{13}C$ 393.2254.

2-(5-(2,5-dimethylphenoxy)-2,2-dimethylpentanoyl)-8-phenyl-5-oxa-2-azaspiro[3.4]octan-6-one-6-^{13}C (66)

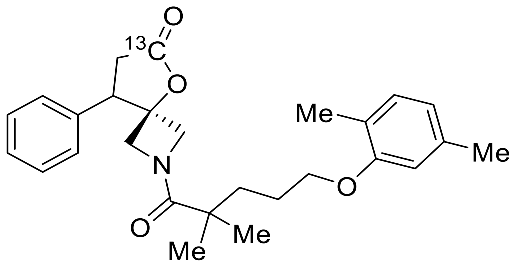

Prepared according to general procedure **K** using 5-(2,5-dimethylphenoxy)-1-(3-hydroxy-3-(1-phenylvinyl)azetidin-1-yl)-2,2-dimethylpentan-1-one (81 mg, 0.2 mmol). Pure product was obtained using workup B and flash column chromatography (50/50 of Hexane/EtOAc) to provide the title compound as a white solid (36 mg, 35% yield). $[M+H]^+$ found 437.2513 $C_{26}H_{34}NO_4{}^{13}C$ requires 437.2516

3-phenylpropanoic acid (67)

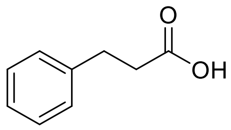

Prepared according to general procedure **L** using styrene (23 μL, 0.2 mmol). Pure product was obtained using workup A and flash column chromatography (99/01 to 90/10 Hexane/EtOAc) to provide the title compound as a white solid (22.6 mg, 75% yield). Characterization data are in accordance with previous reports.[19] ^{1}H NMR (300 MHz, $CDCl_3$) δ 7.35 – 7.20 (m, 5H), 2.98 (t, *J* = 7.8 Hz, 2H), 2.70 (t, *J* = 7.8 Hz, 2H).

3-(4-(*tert*-butyl)phenyl)propanoic acid (68)

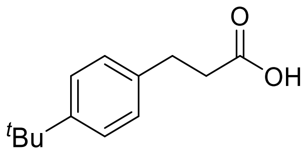

Prepared according to general procedure **L** using 4-*tert*-Butylstyrene (37 μL, 0.2 mmol). Pure product was obtained using workup A and flash column chromatography (99/01 to 90/10 Hexane/EtOAc) to provide the title compound as a white solid (26.8 mg, 64% yield). Characterization data are in accordance with previous reports.[19] ^{1}H NMR (300 MHz, $CDCl_3$) δ 7.38 – 7.33 (m, 2H), 7.21 – 7.16 (m, 2H), 2.98 – 2.91 (m, 2H), 2.74 – 2.66 (m, 2H), 1.35 (s, 9H).

---

[19] H. Seo, A. Liu, T. F. Jamison *J. Am. Chem. Soc.* **2017**, *139, 40*, 13969–13972

3-(4-methoxyphenyl)propanoic acid (69)

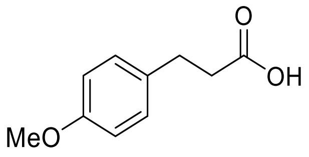

Prepared according to general procedure **L** using 4-Metoxystyrene (27 µL, 0.2 mmol). Pure product was obtained using workup A and flash column chromatography (99/01 to 90/10 Hexane/EtOAc) to provide the title compound as a yellow solid (25.1 mg, 70% yield). Characterization data are in accordance with previous reports.[19] ^{1}H NMR (300 MHz, $CDCl_3$) δ 7.11 (d, J = 8.6 Hz, 2H), 6.82 (d, J = 8.6 Hz, 2H), 3.78 (s, 3H), 2.89 (t, J = 7.7 Hz, 2H), 2.63 (t, J = 7.7 Hz, 2H).

3-(4-fluorophenyl)propanoic acid (70)

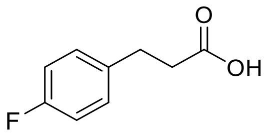

Prepared according to general procedure **L** using 4-Fluorostyrene (25 µL, 0.2 mmol). Pure product was obtained using workup A and flash column chromatography (99/01 to 90/10 Hexane/EtOAc) to provide the title compound as a white solid (17.4 mg, 52% yield). Characterization data are in accordance with previous reports.[19] ^{1}H NMR (300 MHz, $CDCl_3$) δ 7.19 – 7.13 (m, 2H), 7.00 – 6.94 (m, 2H), 2.92 (t, J = 7.6 Hz, 2H), 2.65 (t, J = 7.6 Hz, 2H).

2-(1,2,3,4-tetrahydronaphthalen-1-yl)acetic acid (71)

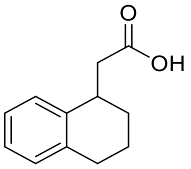

Prepared according to general procedure **L** using 1-methylene-1,2,3,4 tetrahydronaphthalene (28.8 mg, 0.2 mmol). Pure product was obtained using workup A and flash column chromatography (95/05 to 90/10 Hexane/EtOAc) to provide the title compound as a white solid (22.9 mg, 60% yield). Characterization data are in accordance with previous reports.[19] ^{1}H NMR (300 MHz, $CDCl_3$) δ 7.24 – 7.01 (m, 4H), 3.42 – 3.30 (m, 1H), 2.86 – 2.73 (m, 3H), 2.66 – 2.53 (m, 1H), 2.03 – 1.72 (m, 4H).

4-(3-(2-carboxyethyl)phenoxy)butanoic acid (72)

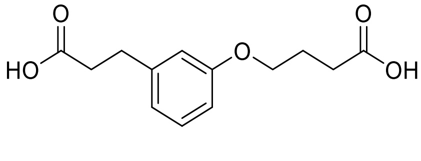

Prepared according to general procedure **L** using 4-(3-vinylphenoxy)butanoic acid (41.2 mg, 0.2 mmol). Pure

product was obtained using workup A and flash column chromatography (40/60 to 20/80 Hexane/EtOAc) to provide the title compound as a yellow liquid (16 mg, 31% yield). ^{1}H NMR (300 MHz, $CDCl_3$) δ 7.24 – 7.15 (m, 1H), 6.86 – 6.71 (m, 3H), 4.07 – 3.95 (m, 2H), 3.00 – 2.84 (m, 2H), 2.77 – 2.48 (m, 4H), 2.16 – 2.08 (m, 2H).

3-(4-(methyl(3-phenyl-3-(4-(trifluoromethyl)phenoxy)propyl)carbamoyl)phenyl)propanoic acid (73)

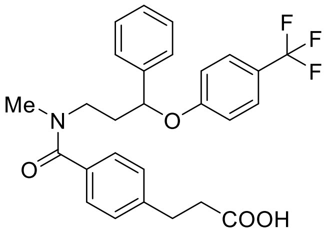

Prepared according to general procedure **L** using N-methyl-N-(3-phenyl-3-(4-(trifluoromethyl)phenoxy)propyl)-4-vinylbenzamide (88 mg, 0.2 mmol). Pure product was obtained using workup A and flash column chromatography (70/30 to 50/50 of Hexane/EtOAc) to provide the title compound as a yellow liquid (52 mg, 54% yield). ^{1}H NMR (300 MHz, $CDCl_3$) δ 7.52 – 7.27 (m, 7H), 7.25 – 6.60 (m, 6H), 5.17 (d, J = 90.9 Hz, 1H), 3.63 (d, J = 51.5 Hz, 2H), 3.21 – 2.62 (m, 5H), 2.57 – 1.84 (m, 4H). ^{13}C NMR (75 MHz, $CDCl_3$) δ 171.28, 159.97, 136.09, 128.95, 128.07, 126.08, 122.74, 115.70, 115.18, 78.54, 47.80, 45.11, 37.95, 37.41, 36.08, 33.10.

4-(*tert*-butoxy)-3-methyl-4-oxobutanoic acid (74)

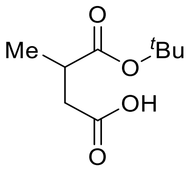

Prepared according to general procedure **L** using *tert*-butyl methacrylate (33 µL, 0.2 mmol). Pure product was obtained using workup A and flash column chromatography (98/02 to 90/10 Hexane/EtOAc) to provide the title compound as a yellow liquid (22.9 mg, 61% yield). Characterization data are in accordance with previous reports.[20] ^{1}H NMR (300 MHz, $CDCl_3$) δ 2.86 – 2.68 (m, 2H), 2.47 – 2.37 (m, 1H), 1.46 (s, 9H), 1.22 (d, J = 6.8 Hz, 3H).

4-(allyloxy)-3-methyl-4-oxobutanoic acid (75)

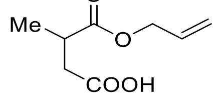

Prepared according to general procedure **L** using allyl methacrylate (26 mg, 0.2 mmol). Pure product was

[20] He, H., Jian-Heng, Y., Lei, Z., Chuan-Kun, R., Meng, M., Wei, W., Hanjiao, C., Wen-Jun, Z., Yu, L., Bo, Y., & Da-Gang, Y. *CCS Chemistry* **2020**, *3, 6*, 1746–1756.

obtained using workup A and flash column chromatography (70/30/00 to 70/28/02 of Hexane/EtOAc/AcOH) to provide the title compound as a colorless liquid (21 mg, 61% yield). Characterization data are in accordance with previous reports.[21] ^{1}H NMR (300 MHz, $CDCl_3$) δ 5.96 – 5.82 (m, 1H), 5.43 – 5.2.8 (m, 1H), 5.21 (dt, *J* = 10.6, 1.3 Hz, 1H), 4.60 (dt, *J* = 5.8, 1.5 Hz, 2H), 2.98 – 2.87 (m, 1H), 2.81 (dd, *J* = 16.9, 8.2 Hz, 1H), 2.47 (dd, *J* = 16.9, 5.9 Hz, 1H), 1.26 (d, *J* = 7.1 Hz, 3H).

4-ethoxy-2,2-dimethyl-4-oxobutanoic acid (76)

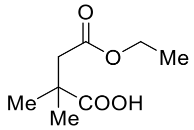

Prepared according to general procedure **L** using ethyl 3-methylbut-2-enoate (26 mg, 0.2 mmol). Pure product was obtained using workup A and flash column chromatography (75/25/00 to 75/23/02 of Hexane/EtOAc/AcOH) to provide the title compound as a pale-yellow liquid (15 mg, 61% yield). Characterization data are in accordance with previous reports.[22] ^{1}H NMR (300 MHz, $CDCl_3$) δ 4.17 – 4.08 (m, 2H), 2.60 (s, 2H), 1.29 (s, 6H), 1.24 (t, *J* = 7.5 Hz, 3H).

3-methyl-4-morpholino-4-oxobutanoic acid (77)

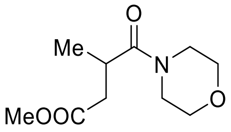

Prepared according to general procedure **L** using 2-methyl-1-morpholinoprop-2-en-1-one (31 mg, 0.2 mmol). The carboxylic acid product was obtained using workup A. The crude was subjected to methylation reaction using methyl iodide (0.19 mL, 3 mmol) and KOH (18 mg, 0.3 mmol) and stirred for 1hr. The final pure product was obtained by flash column chromatography (90/10 of Acetone/AcOH) to provide the title compound as a yellow liquid (19 mg, 48% yield). ^{1}H NMR (300 MHz, $CDCl_3$) δ 3.76 – 3.43 (m, 11H), 3.20 – 3.04 (m, 1H), 2.85 (dd, *J* = 16.8, 8.9 Hz, 1H), 2.32 (dd, *J* = 16.9, 5.1 Hz, 1H), 1.10 (d, *J* = 7.1 Hz, 3H). ^{13}C NMR (75 MHz, $CDCl_3$) δ 173.88, 172.87, 66.84, 66.67, 51.59, 46.08, 42.23, 37.71, 31.68, 17.49.

2,3-dihydrobenzo[b]thiophene-2-carboxylic acid (78)

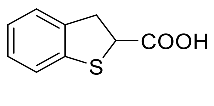

Prepared according to general procedure **L** using benzo[b]thiophene (27 mg, 0.2 mmol). Pure product was obtained using workup A and flash column chromatography (78/20/02 of Hexane/EtOAc/AcOH) to provide the title compound as a brown liquid (20 mg, 56% yield). Characterization data are in accordance with previous

---

[21] Sheta, A. M.: Alkayal, A.; Mashaly, M. A.; Said, S. B.; Elmorsy, S. S.; Malkov, A. V. Buckley, B. R. *Angew. Chem. Int. Ed.* **2021**, *60*, 21832.
[22] Alektiar, S. N.; Wickens, Z. K. *J. Am. Chem. Soc.* **2021**, *143*, 13022.

reports.[23] ^{1}H NMR (300 MHz, $CDCl_3$) δ 7.58 (brs, 1H), 7.22 – 7.10 (m, 3H), 7.09 – 7.00 (m, 1H), 4.43 (dd, *J* = 8.7, 4.9 Hz, 1H), 3.66 (dd, *J* = 16.0, 4.8 Hz, 1H), 3.48 (dd, *J* = 16.0, 8.7 Hz, 1H).

1-acetylindoline-2-carboxylic acid (79)

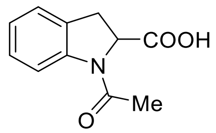

Prepared according to general procedure **L** using 1-(1H-indol-1-yl)ethan-1-one (32 mg, 0.2 mmol). Pure product was obtained using workup A and flash column chromatography (50/50/00 to 50/48/02 of Hexane/EtOAc/AcOH) to provide the title compound as a pale brown liquid (25 mg, 62% yield). Characterization data are in accordance with previous reports.[24] ^{1}H NMR (300 MHz, DMSO-d6) δ 7.86 – 7.92 (m, 1H), 7.23 – 7.17 (m, 2H), 6.99 (dt, *J* = 7.5, 1.2 Hz, 1H), 5.19 (dd, *J* = 6.9, 1.9 Hz, 1H), 3.62-3.55 (m, 1H), 3.18-3.14 (m, 1H), 2.17 (brs, 3H).

2-methyl-1-oxo-1,2,3,4-tetrahydroisoquinoline-3-carboxylic acid (80)

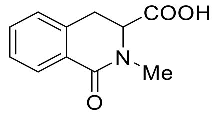

Prepared according to general procedure **L** using 2-methylisoquinolin-1(2H)-one (32 mg, 0.2 mmol). Pure product was obtained using workup A and flash column chromatography (50/50/00 to 50/48/02 of Hexane/EtOAc/AcOH) to provide the title compound as a colorless liquid (21 mg, 52% yield). ^{1}H NMR (300 MHz, DMSO-d6) δ 7.85 (dd, *J* = 7.6, 1.4 Hz, 1H), 7.44 (td, *J* = 7.4, 1.5 Hz, 1H), 7.37 – 7.24 (m, 2H), 4.42 (dd, *J* = 6.9, 1.9 Hz, 1H), 3.50 (brs, 1H), 3.41 (dd, *J* = 16.3, 6.9 Hz, 1H), 3.19 (dd, *J* = 16.3, 1.9 Hz, 1H), 3.01 (s, 3H). ^{13}C NMR (75 MHz, DMSO-d6) δ 172.5, 163.8, 135.9, 131.7, 128.8, 127.5, 127.1, 126.9, 59.6, 34.6, 30.3. HRMS (ESI) $[M-H]^+$ found 204.0669, $C_{11}H_{10}NO_3$ requires 204.0666

---

[23] Bains, A. K.; Sau, A.; Portela, B.S.; Kajal, K.; Green, A.R.; Wolff, A. M.; Patin, L. F.; Paton, R. S.; Damrauer, N. H.; Miyake, G. M. *Science* **2025**, *388*, 1294.
[24] Mangaonkar, S. R.; Hayashi, H.; Takano, H.; Kanna, W.; Maeda, S.; Mita, T. *ACS Catal.* **2023,** *13*, 2482.

5. Mechanistic Studies

Cyclic Voltammetry Studies

CV measurements were performed with a three-electrode potentiostat by using a glassy carbon working electrode, platinum wire counter electrode and Ag/$AgNO_3$ (0.01 M) in MeCN as a reference electrode. The voltammograms were taken in a degassed DMF solution ([n-Bu_4NBF_4] = 0.1 M, [substrate] = 1 mM, referenced by Ag/ AgNO3 (0.01 M) in MeCN) under Ar atmosphere. The scan rate was kept at 500 mV/s.

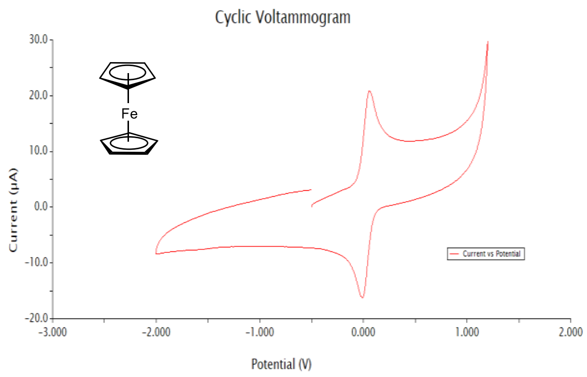

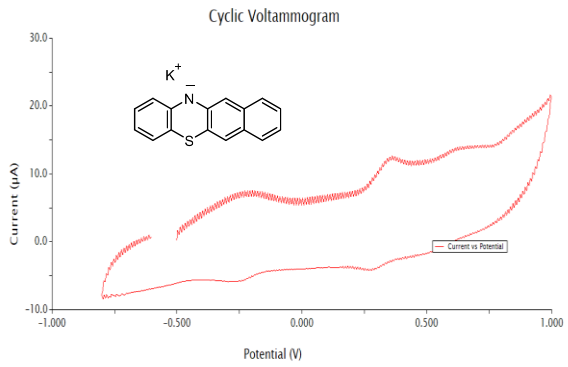

For better comparability with the literature, CVs were measured relative to ferrocene (Fc^+ /Fc) and converted to the saturated calomel electrode (SCE). According to the literature,[25] $E_{1/2}$ (Fc^+ /Fc) in DMF has a value of +0.436 V against SCE. Our measurement against Ag/$AgNO_3$ was $E_{1/2}$ (Fc^+ /Fc) = 0.027 V. Thus, for the conversion to SCE, +0.409 V were added to our values.

$$E_{1/2}\,(BPTZ^{\bullet}/BPTZ^{-}) = -0.232\ V + 0.409\ V = +\ 0.177\ V \text{ vs SCE in DMF.}$$

Note: The benzophenothiazine anion solution was prepared by mixing 5 mg of KH (30% wt. dispersion in mineral oil) with 1 mM DMF solution of BPTZ.

[25] D. Bao, B. Millare, W. Xia, B. G. Steyer, A. A. Gerasimenko, A. Ferreira, A. Contreras, V. I. Vullev *J. Phys. Chem. A* **2009**, *113*, 1259–1267.

UV-Vis studies

The UV-Vis absorption spectra were recorded using a Jasco V-770 spectrophotometer equipped with photomultiplier detector, monochromator and deuterium/halogen light source. The samples were prepared in a 1 mL quartz cuvette with a path length of 1 cm.

The absorption spectra of benzophenothiazine **10**, benzophenothiazine anion **1** and the benzophenothiazine anion solution bubbled with CO_2 were recorded in DMF. 100 µM solution of benzophenothiazine **10** was prepared and its absorption spectra was recorded and indicated (blue line). To a 100 µM solution of benzophenothiazine **10** was added 5 mg of *ᵗ*BuOK, a change in color from pale green to wine red was observed. The absorption spectrum of this corresponding solution was recorded and indicated (orange line). CO_2 was bubbled through the benzophenothiazine anion **1** solution for 5 minutes, a change in color from wine red to bright green was observed. The absorption spectrum of the corresponding solution was recorded and indicated (green line).

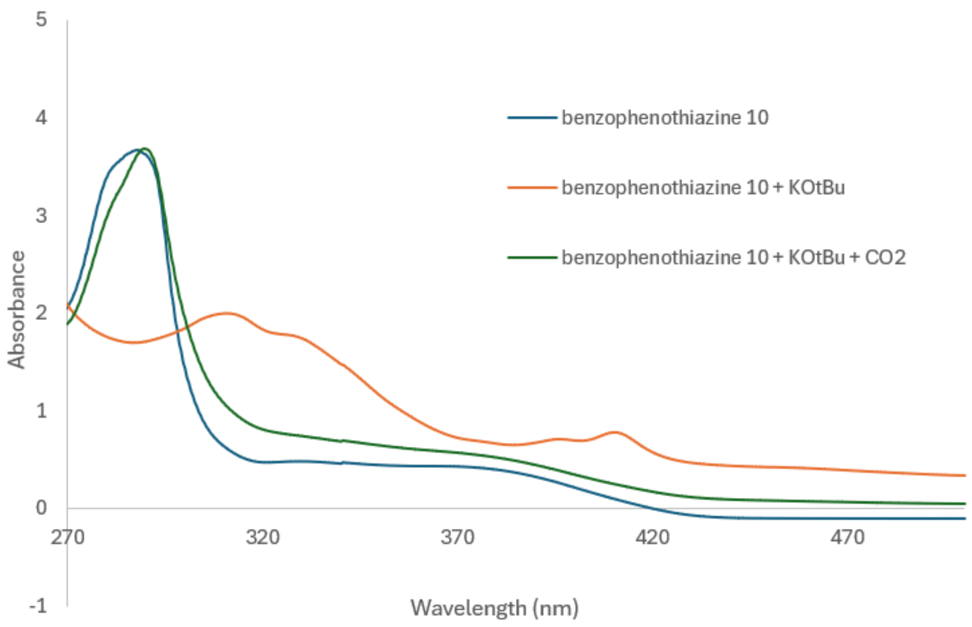

As can be seen above, the orange line indicates the formation of benzophenothiazine anion **1** when 5 mg of *ᵗ*BuOK was dissolved in a 100 µM solution of benzophenothiazine **10** in DMF, whereas the green line indicates the formation of carbamate when CO_2 is bubbled for 5 minutes through the solution of 100 µM benzophenothiazine anion **1** in DMF.

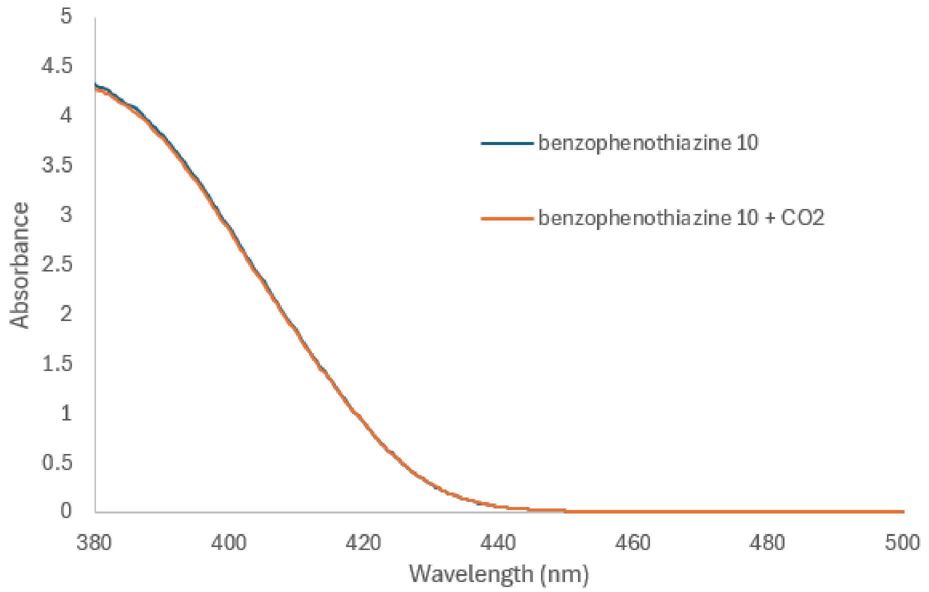

The UV-Vis spectrum above shows no interaction or the formation of transient carbamate when CO_2 was bubbled for 5 min through 1000 µM solution of benzophenothiazine **10** in DMF.

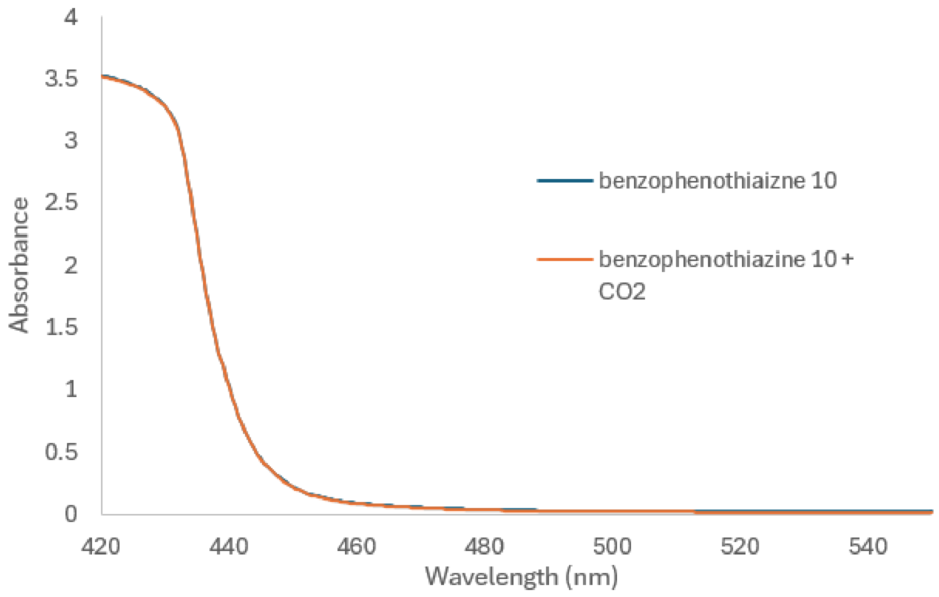

The UV-Vis spectrum above shows no interaction or the formation of transient carbamate when CO_2 was bubbled for 5 min through a 100 mM solution of benzophenothiazine **10** in DMF.

Emission studies

The emission spectra were recorded using a FS5 Spectrofluorometer equipped with photomultiplier detector, double monochromator and 150 W xenon light source. The fluorescence emission was recorded using a 3 mL of a 50 µM solution in DMF in a quartz fluorescence cuvette (10x10 mm light path) equipped with septum. The sample was degassed with a stream of Argon for 5 minutes, in the case of benzophenothiazine **10** and its corresponding anion **1**. In the case of the carbamate, 50 µM benzophenothiazine anion **1** solution in DMF was degassed with a stream of CO_2 for 5 minutes. The excitation wavelength was fixed at 370 nm (bandwidth = 3.5 nm), while the emission spectra was acquired from 400 nm to 700 nm.

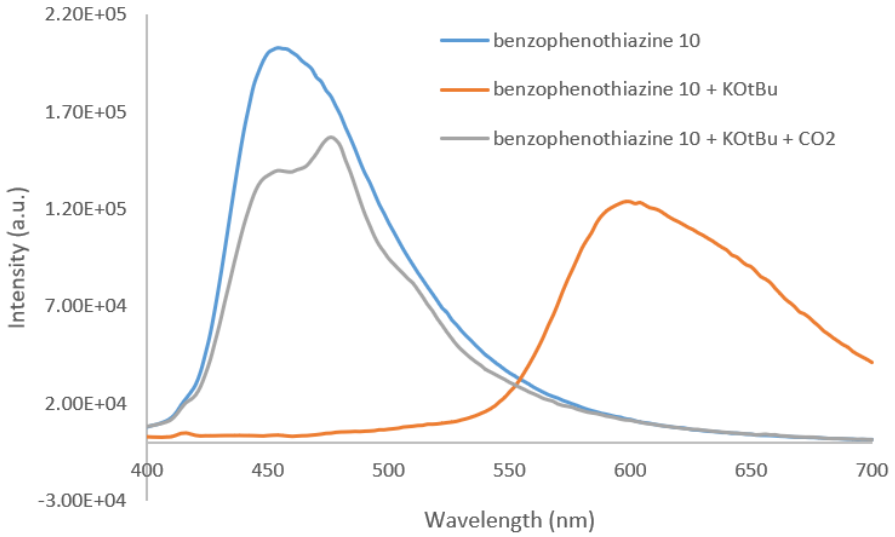

The fluorescence emission studies also suggest the formation of the carbamate evidenced by the new characteristic emission curve observed between the benzophenothiazine anion **1** and CO_2.

Detection of formate upon irradiation of pre-synthetized CO_2 carbamate

Synthesis and Reaction of Phenothiazine Lithium Carbamate•2THF complex 11

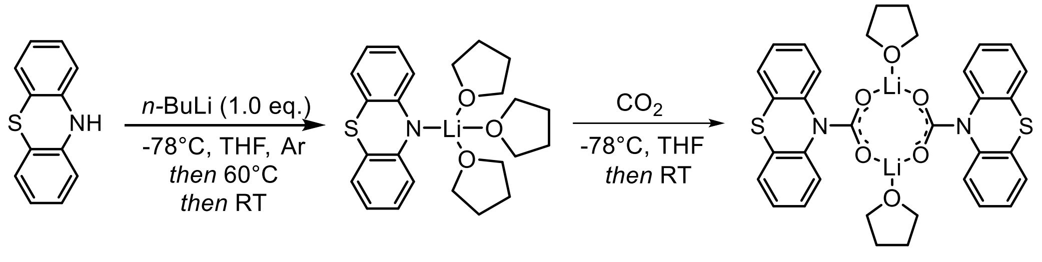

The Phenothiazine Lithium Carbamate•2THF complex **11** was synthesized according to a procedure reported by Ball.[26] In a flame-dried 10 mL Schlenk were dissolved 2.5 mmol of phenothiazine in 5 mL of THF. Then the reaction was cooled to -78°C and *n*-BuLi (2.5 M in hexanes, 1.0 eq.) was added dropwise. The reaction turned from yellow to orange and was stirred at this temperature for 10 minutes, then warmed up to 60°C until the solids were all dissolved. The mixture was allowed to rest for 2 hours at room temperature, then cooled

---

[26] S. C. Ball, I. Cragg-Hine, M. G. Davidson, R. P. Davies, A. J. Edwards, I. Lopez-Solera, P. R. R. Raithby, R. Snaith, *Angew Chem Int Ed.* ***1995****. 34, 8*, 921-923

again to -78°C. At this temperature, CO_2 was bubbled for 5 minutes, and the mixture turned from orange to bright yellow. The reaction was warmed to room temperature for 16 hours and a white solid started to form. The solvent was then removed under vacuum with the help of the Schlenk line. To the resulting residue was added 8 mL of dry toluene under argon, a white microcrystalline solid started forming. After 1 hour, the solid was decanted, the toluene removed under Argon and the resulting residue rinsed again with 4 mL of dry toluene. The solid was decanted again, the toluene removed and the resulting microcrystalline solid dried under high vacuum. The white solid obtained in a final yield of 38% was identified as the Phenothiazine Lithium Carbamate·2THF complex **11** and characterized with ^{1}H-NMR and ^{13}C-NMR in benzene-d^6 at 10°C. However, as the authors previously reported the CRMM (cryoscopisc relative molecular mass measurements) performed by their group reported an association state (n) values of 2.01± 0.09 meaning that the dominant species in solution (so also for the NMR studies) should be the dimer (the authors also did only report only the ^{1}H characterization of this carbamate species, that is consistent with our observations: ^{1}H-NMR: C_6D_6 250 MHz, 25C =7.1-6.6 (m, 8H) 3.35 (s, 4H, THF), 1.37 (s, 4H. THF); suggesting that the solid represents a mixture of free lithium carbamate and THF-coordinated complex species.

The presence of two peaks at 158 ppm and 160 ppm in the ^{13}C-NMR is also consistent with the plausible formation of carbamate species that is only stabilized at low temperatures.

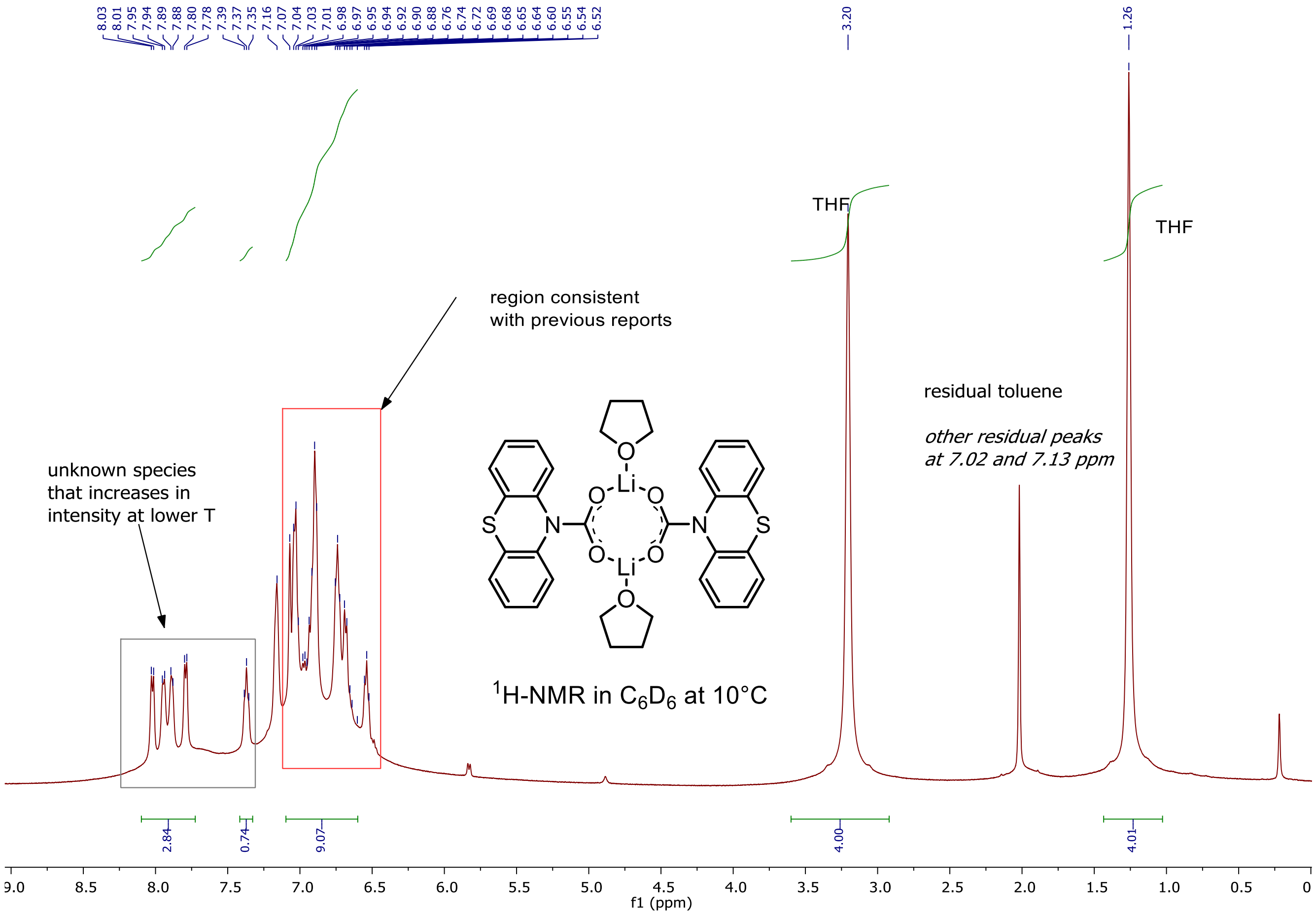

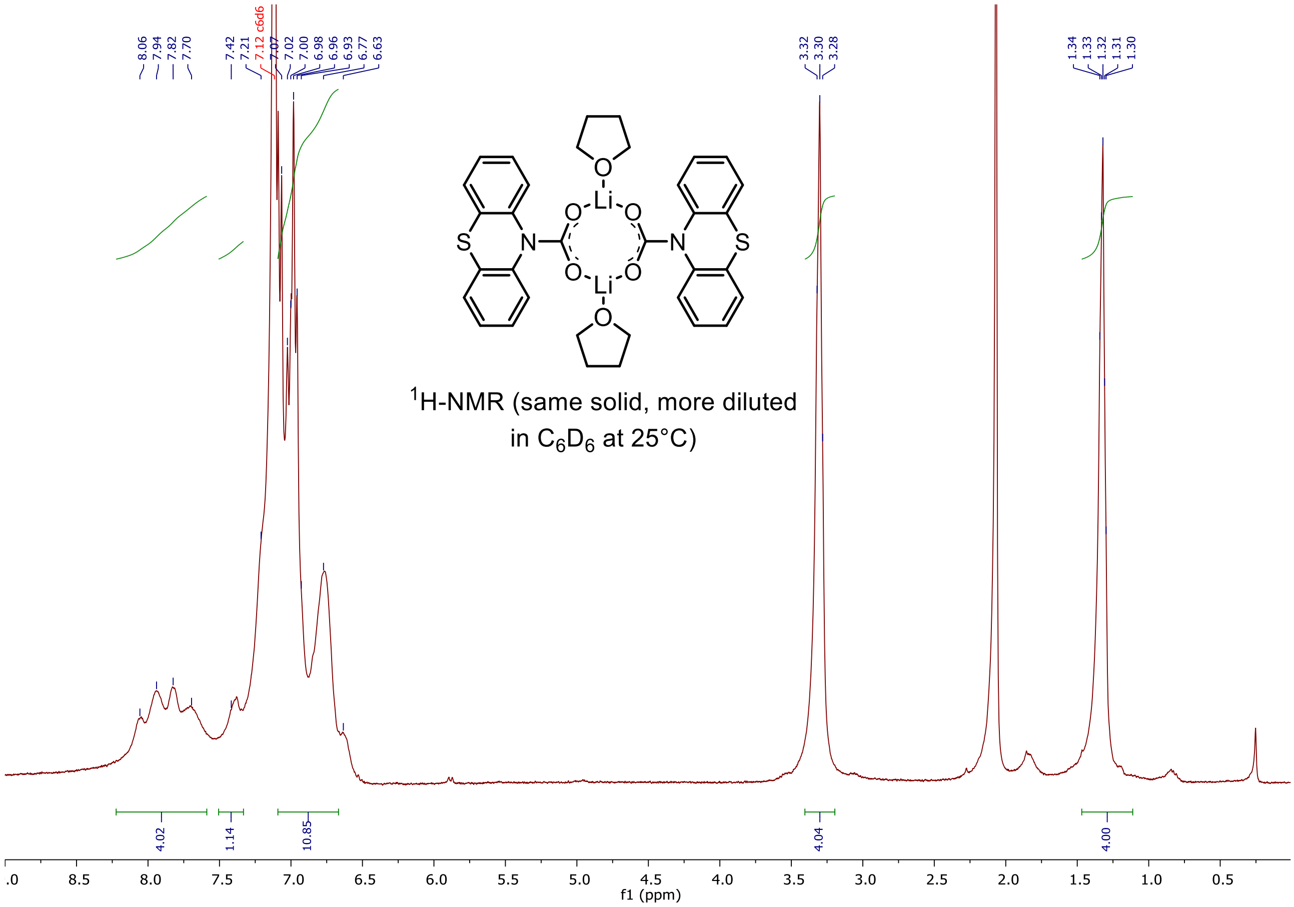

8.06
7.94
7.82
7.70
7.42
7.21
7.12 c6d6
7.07
7.02
7.00
6.98
6.96
6.93
6.77
6.63
3.32
3.30
3.28
1.34
1.33
1.32
1.31
1.30
^{1}H-NMR (same solid, more diluted in C_6D_6 at 25°C)
4.02
1.14
10.85
4.04
4.00
f1 (ppm)

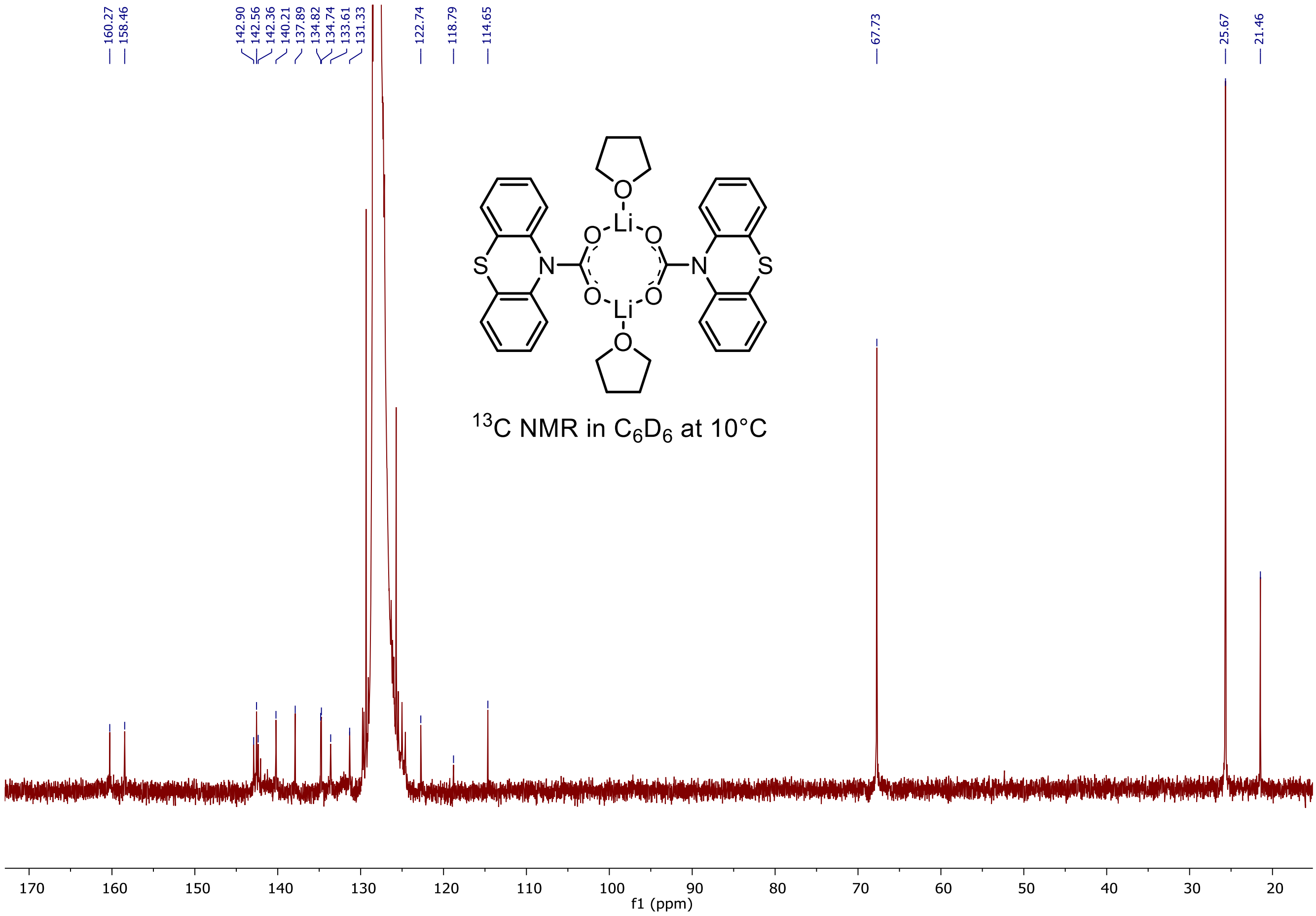

160.27
158.46
142.90
142.56
142.36
140.21
137.89
134.82
134.74
133.61
131.33
122.74
118.79
114.65
67.73
25.67
21.46
^{13}C NMR in C_6D_6 at 10°C
f1 (ppm)

AT-IR spectrum of the Phenothiazine Lithium Carbamate•2THF complex 11

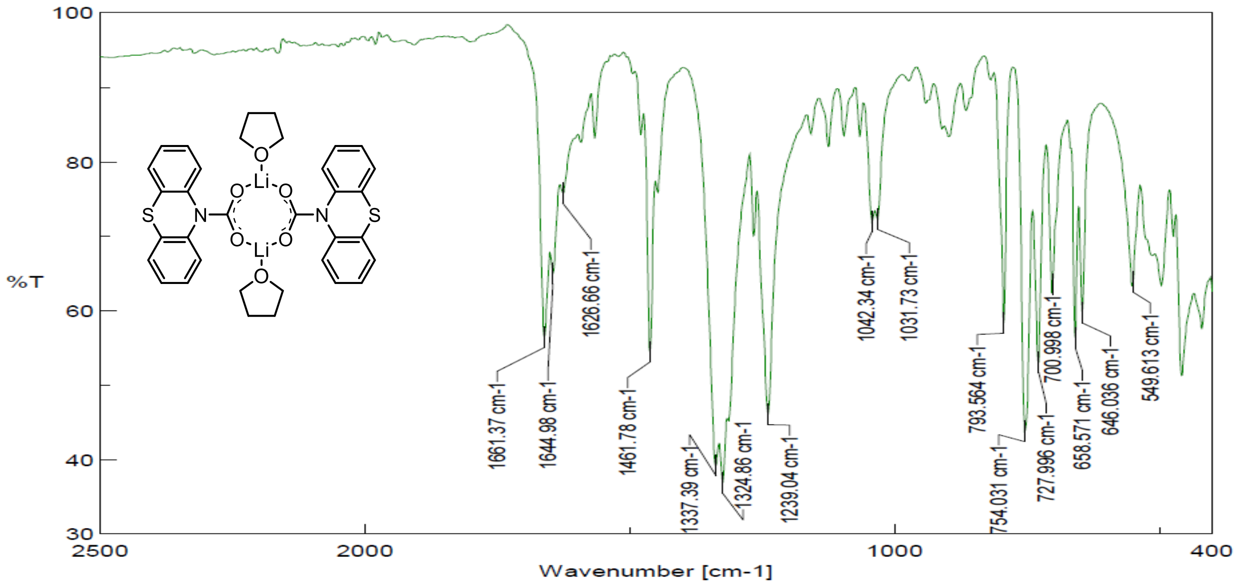

Signals attributed to the carbamate species can be identified at 1661.37 cm^{-1} and 1644 cm^{-1}

Detection of Formate for the reaction of Phenothiazine Lithium Carbamate•2THF complex 11 under irradiation at 390 nm

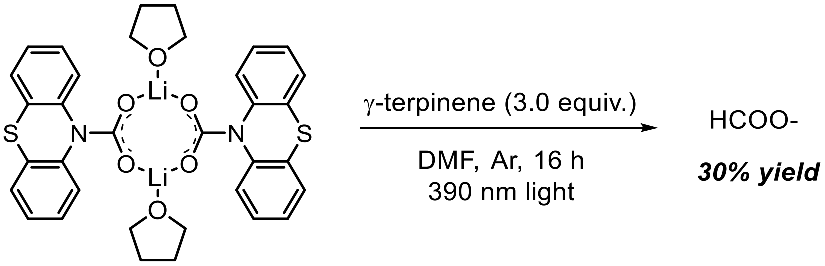

A flame-dried 10 mL Schlenk tube was backfilled with Ar, then 65 mg (0.1 mmol of the dimer, 0.2 mmol of carbamate) of the Phenothiazine Lithium Carbamate·2THF complex **11** was added together with γ-terpinene (96 µL, 0.6 mmol, 3.0 eq.) and 1 mL of DMF under Ar atmosphere. The Schlenk was then irradiated for 16 hours with 390 nm lamp (placed in the carousel as described in Chapter 4. Photochemical reactions). Then 2 mL of a 0.05 M solution of sodium *para*-toluensulfonate in water was added to the reaction mixture. The resulting solution was evaporated to remove the solvents, then the resulting crude was dissolved in $CDCl_3$ and D_2O and the water phase collected and analyzed by ^{1}H-NMR to provide detection and quantification of formate yield. The reaction provided production of formate in 30% yield with respect to the starting amount of carbamate thus suggesting the possible generation of $CO_2^{\bullet-}$ upon photolysis of the carbamate species

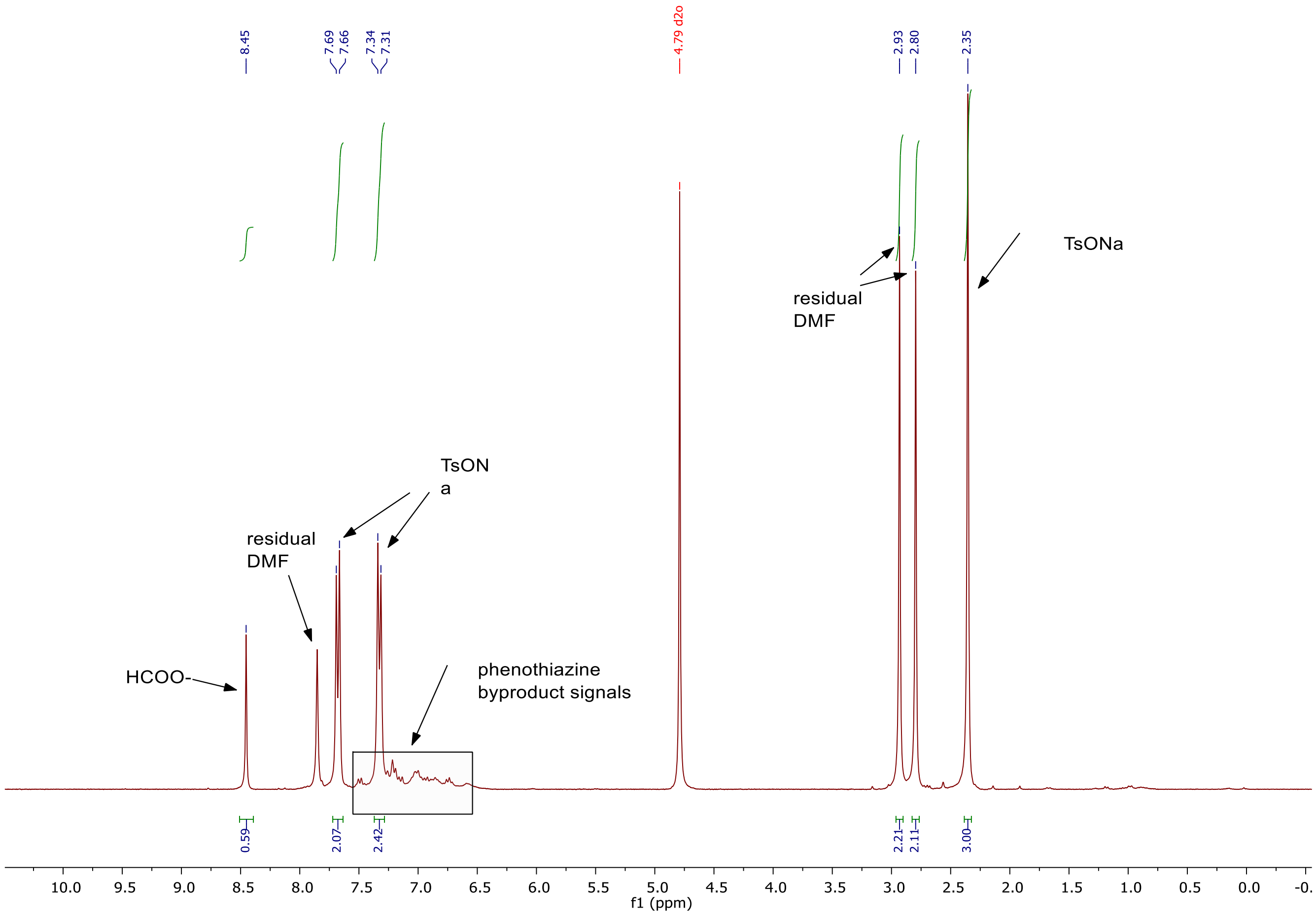

Detection of Formate for the reaction of Phenothiazine Lithium Carbamate· 2THF complex 11, in the dark

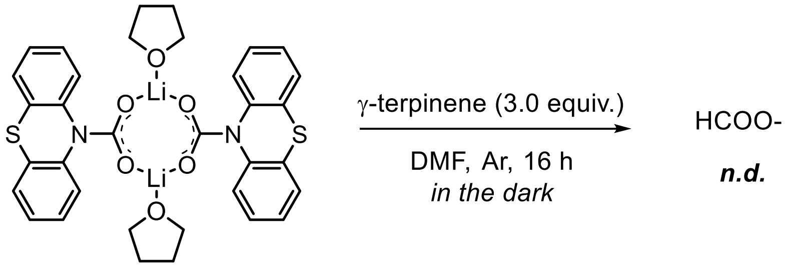

A flame-dried 10 mL Schlenk tube was backfilled with Ar, then the Phenothiazine Lithium Carbamate· 2THF complex **11** was added together with γ-terpinene and 1 mL of DMF under Ar atmosphere. The Schlenk was then kept in the dark while stirring for 16 hours.

Then 2 mL of a 0.05 M solution of sodium *para*-toluensulfonate in water was added to the reaction mixture. The resulting solution was evaporated to remove the solvents, then the resulting residue was dissolved in $CDCl_3$ and D_2O and the water phase collected and analyzed by ^{1}H-NMR and ^{13}C-NMR to provide detection and quantification of formate yield. The formate was not detected by ^{1}H-NMR in D_2O, thus confirming that the interaction between the carbamate and visible light is necessary to provide formation of $CO_2^{\bullet-}$.

FT-IR monitoring of the reaction

Real-time *in-situ* reaction analysis were carried out using Mettler-Toledo ReactIR, System ReactIR 702L (optical range 4000 cm^{-1} to 650 cm^{-1}), TE MCT as detector and iC IR software to obtain mechanistic insights of the reaction. Since the reaction proved to be effective also in DMSO (see optimization tables), DMSO was used instead of DMF, to avoid interference of the carbonyl of the amide in the region of the carbamate, $CO_2^{\bullet-}$ and formate signals. Since the reaction proved to be effective under and atmosphere of CO_2 due to a balloon, with the solvent degassed with CO_2 (see optimization tables) and since FT-IR probe should be submerged in the reaction media, instead of using freeze-pump-thaw, the reaction was conducted in a 2-necked flask. One neck dedicated to fit a septum around the FT-IR probe and the other sealed with a septum and pinched with a balloon of CO_2 instead (see figure below). The reaction was also scaled up to 1.2 mmol of the photocatalyst to properly monitor the evolution of the intermediate species.

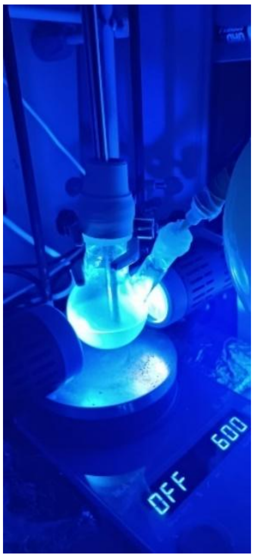

Reaction setup for FT-IR monitoring of the reaction

FT-IR monitoring of the reaction with irradiation, detection of CO_2 radical anion/formate

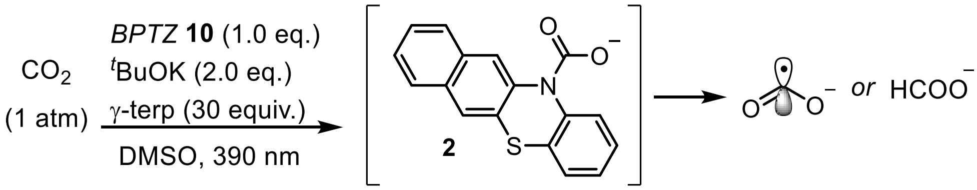

Under a CO_2 atmosphere, a flame-dried 100 mL 2-necked flask was charged with 60 mL of dry DMSO previously degassed with CO_2 for 20 minutes. Then γ-terpinene (4.9 g, 5.77 mL, 30.0 eq.) was added and further degassed with CO_2 for 5 minutes. Finally, benzophenothiazine (300 mg, 1.0 eq.), 2.4 mmol of freshly sublimed ^{t}BuOK (270 mg, 2.0 eq.) were added and the resulting mixture was further degassed for 10 minutes. Then the 2-necked flask was sealed and connected to the FT-IR probe through the central septum while maintaining the atmosphere of CO_2 with a balloon pinched in the lateral septum. The reaction was then subjected to stirring and irradiation with two 390 nm lamps while the *in-situ* FTIR data was monitored at 15

seconds intervals for 12 hours.

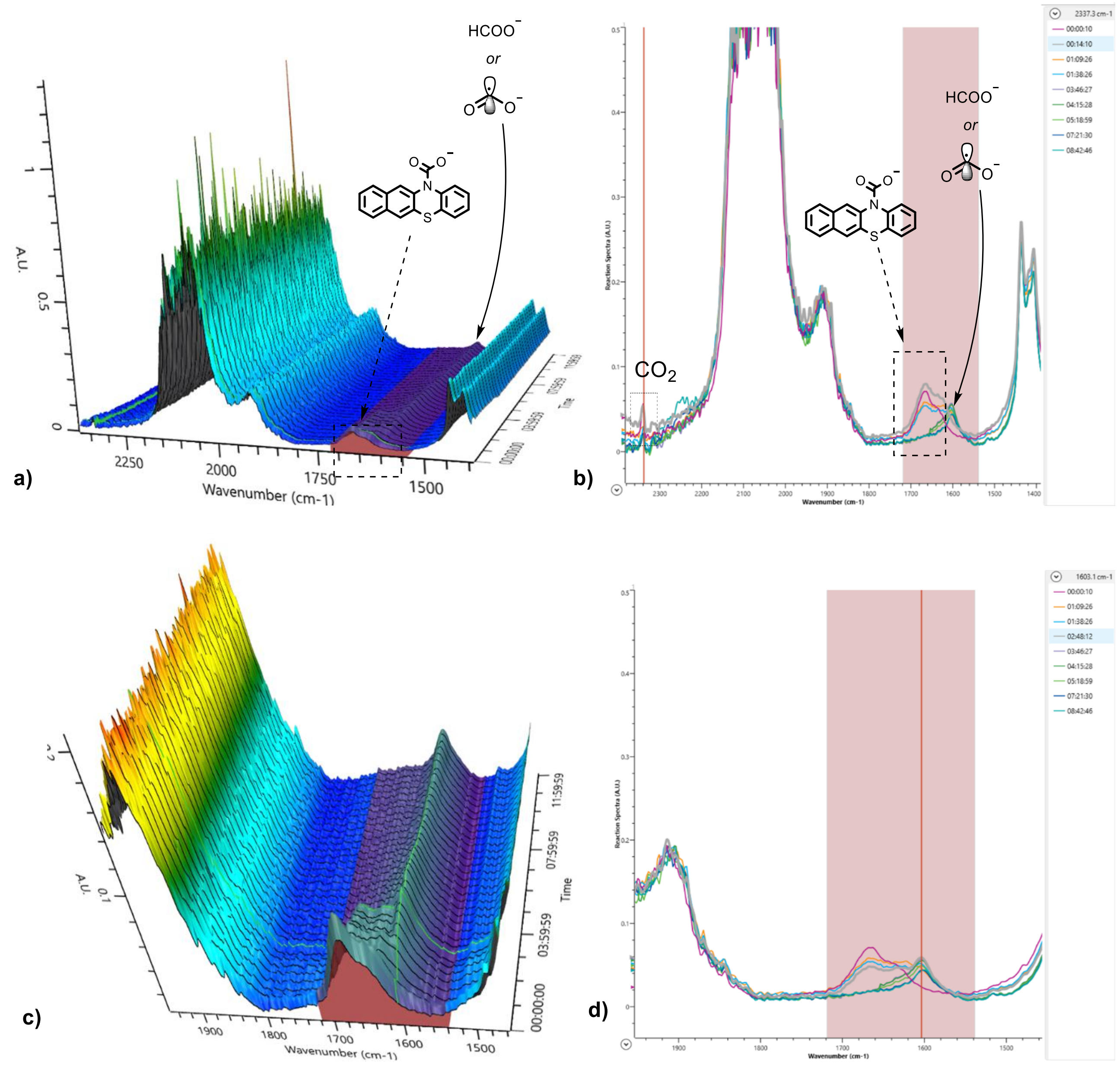

Figure: FT-IR monitoring of the reaction under irradiation**. a)** The surface of FT-IR **b)** FT-IR spectra of the reaction under irradiation, the red line indicates the peak of CO_2 at 2335 cm^{-1}. **c)** The surface of the FT-IR magnified in the carbamate-formate-$CO_2^{\bullet -}$ region. **d)** FT-IR spectra of the reaction under irradiation, magnified in the carbamate-formate-$CO_2^{\bullet -}$ region, the red line indicates the peak of formate or $CO_2^{\bullet -}$ at 1610 cm^{-1}.

The formation of the typical peaks of the carbamate (1660 cm^{-1}, 1620 cm^{-1}, consistent with the AT-IR of the parent species of the Lithium complex derived from phenothiazine, see above) was observed already in the initial seconds of the reaction, confirming its formation in solution. The consumption of the CO_2 (peak ~ 2325 cm^{-1}) was observed over the time, together with lowering in the intensity of the carbamate peaks in favor of a

new IR band at 1610 cm^{-1}, assigned to the $CO_2^{\bullet-}$ or formate,[27] suggesting the *in-situ* generation of the nucleophilic $CO_2^{\bullet-}$.

FT-IR monitoring of the reaction with irradiation, detection of oxalate

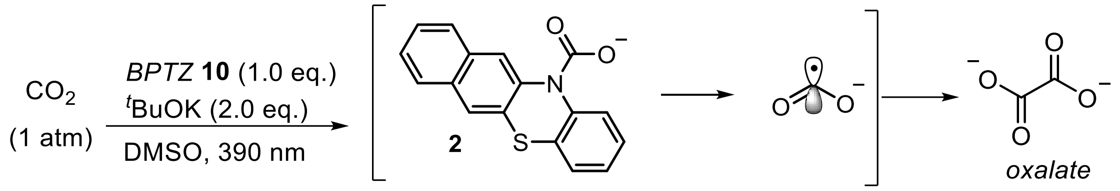

Under a CO_2 atmosphere, a flame-dried 100 mL 2-necked flask was charged with 60 mL of dry DMSO previously degassed with CO_2 for 20 minutes. *In this case, no γ-terpinene was added to reaction mixture*. The benzophenothiazine (300 mg, 1.0 eq.) and 2.4 mmol of freshly sublimed potassium *tert*-butoxide (270 mg, 2.0 eq.) were added and the resulting mixture was further degassed for 10 minutes. Then the 2-necked flask was sealed and connected to the FT-IR probe through the central septum while maintaining the atmosphere of CO_2 with a balloon pinched in the lateral septum. The reaction was then subjected to stirring and irradiation with two 390 nm lamps while the *in-situ* FTIR data was monitored at 15 seconds intervals for 12 hours.

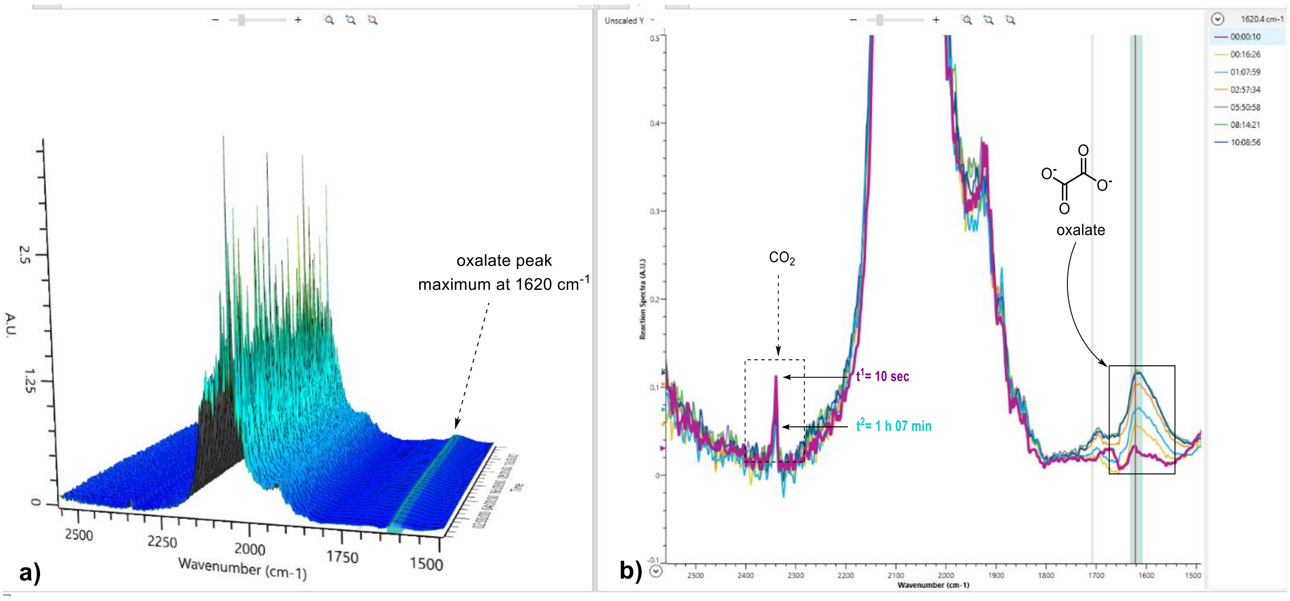

Figure: FT-IR monitoring of the reaction under irradiation. **a)** The surface of FT-IR **b)** FT-IR spectra of the reaction under irradiation, with an increasing intensity of the peak at 1620 cm^{-1} over time and a decreasing intensity of the peak at 2325 cm^{-1} indicating consumption of CO_2

This experiment was performed in the absence of γ-terpinene to circumvent the formate generation upon HAT

[27] a) J. Wu, X. Liu, Y. Hao, S. Wang, R. Wang, W. Du, S. Cha, X.Y. Ma, X. Yang, M. Gong *Angew. Chem. Int. Ed*. **2023**, *62*, e202216083 b) N. Ansmann, D .Hartmann, S. Sailer, P. Erdmann, R. Maskey, M. Schorpp, L. Greb *Angew. Chem. Int. Ed*. **2022**, *61*, e202203947 c) L. González-Sebastián, M. Flores-Alamo, J.J. García *Organometallics* **2013**, *32*, 7186−7194 d) J. Sheng, Y. He, J. Li, C. Yuan, H. Huang, S. Wang, Y. Sun, Z. Wang, F. Dong *ACS Nano* **2020**, *14*, 13103

reaction from γ-terpinene to the $CO_2^{\bullet-}$ ($BDE_{H\text{-}COOH}$ = 112±3 kcal/mol;[28] $BDE_{cyclohexadiene}$ ~75-80 kcal/mol [29]). In the absence of γ-terpinene, the generation of $CO_2^{\bullet-}$ should lead to the formation of oxalate by dimerization. Typical peaks of the oxalate species are reported as a signal with a peak maximum at 1621 cm^{-1}.[30] The consumption of the CO_2 (peak ~ 2325 cm^{-1}) was observed over time, together with increased intensity of a new sharp IR band with a peak maximum at 1620 cm^{-1}, that was attributed to the oxalate, confirming the *in-situ* generation of $CO_2^{\bullet-}$ and its dimerization to the oxalate dianion.

Additional experiments supporting the catalytic generation of $CO_2^{\bullet-}$

Detection of formate in the model reaction

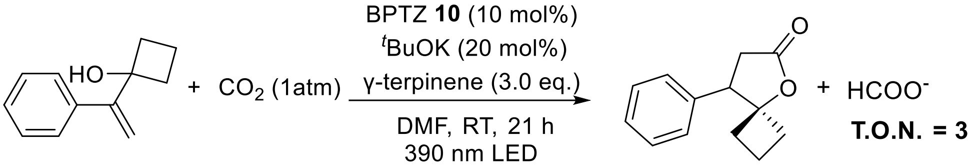

12 *75% yield*

A stock solution of the catalyst mixture was prepared weighing 20 mg of 12*H*-benzo[*b*]phenothiazine and 18 mg of freshly sublimed potassium *tert*-butoxide and accurately dissolved in 4.0 mL of dry DMF (resulting molarity [12*H*-benzo[*b*]phenothiazine] = 0.02 M; ^{t}BuOK = 0.04 M). In a 10 mL Schlenk tube equipped with a stir bar, 1-(1-phenylvinyl)cyclobutan-1-ol (0.2 mmol, 1.0 equiv.), γ-terpinene (96 µL, 0.6 mmol, 3.0 equiv.) and 1.0 mL of the catalyst stock solution was added under air. The solution was subsequently degassed *via* freeze-pump-thaw method (3 cycles) and backfilled with 1 atm CO_2 and irradiated with visible light. The reaction mixture turned from red to yellow after backfilling. *The Schlenk tube was placed in a 3D-printed carousel, resulting in a final distance of 3 cm of the glass wall from the Kessil lamp. The reaction temperature was kept constant using a fan placed on top of the reaction at 20 cm distance.* At the end of the reaction, 50 mg of ^{t}BuOK were added to the reaction mixture, to be sure to convert all to carboxylate or formate species to the corresponding potassium salt and stirred for 5 minutes. Then 2 mL of a 0.05M solution of sodium *para*-toluensulfonate in water was added to the reaction mixture. The resulting solution was evaporated to remove the solvents, then the resulting crude was dissolved in DCM and D_2O and the water phase collected and analyzed by ^{1}H-NMR and ^{13}C-NMR to provide detection and quantification of formate yield.

Formate was detected in the ^{1}H-NMR of the D_2O phase (peak at 8.48 ppm, referencing residual water at 4.79 ppm, **T.O.N = 3** based on the amount of photocatalyst employed).

At the end of this analysis, the water phase evaporated again, and the crude was redissolved in $CDCl_3$ then 1,1,2,2-tetrachloroethane was added to quantify the yield for the model product, resulting in 75% calculated yield.

---

[28] S. J. Blanksby, G. B. Ellison *Acc. Chem. Res.* **2003**, *36, 4*, 255–263

[29] Y. Gao, N. J. DeYonker, E. C. Garrett, III, A. K. Wilson, T. R. Cundari, P. Marshall *J. Phys. Chem. A* **2009**, *113, 25,* 6955–6963

[30] a) R. E. Dinnebier, S. Vensky, M. Panthöfer, M. Jansen *Inorg. Chem.* **2003**, *42, 5*, 1499–1507 b) K. I. Peterson, D. P. Pullman *J. Chem. Educ.* **2016**, *93*, *6*, 1130–1133

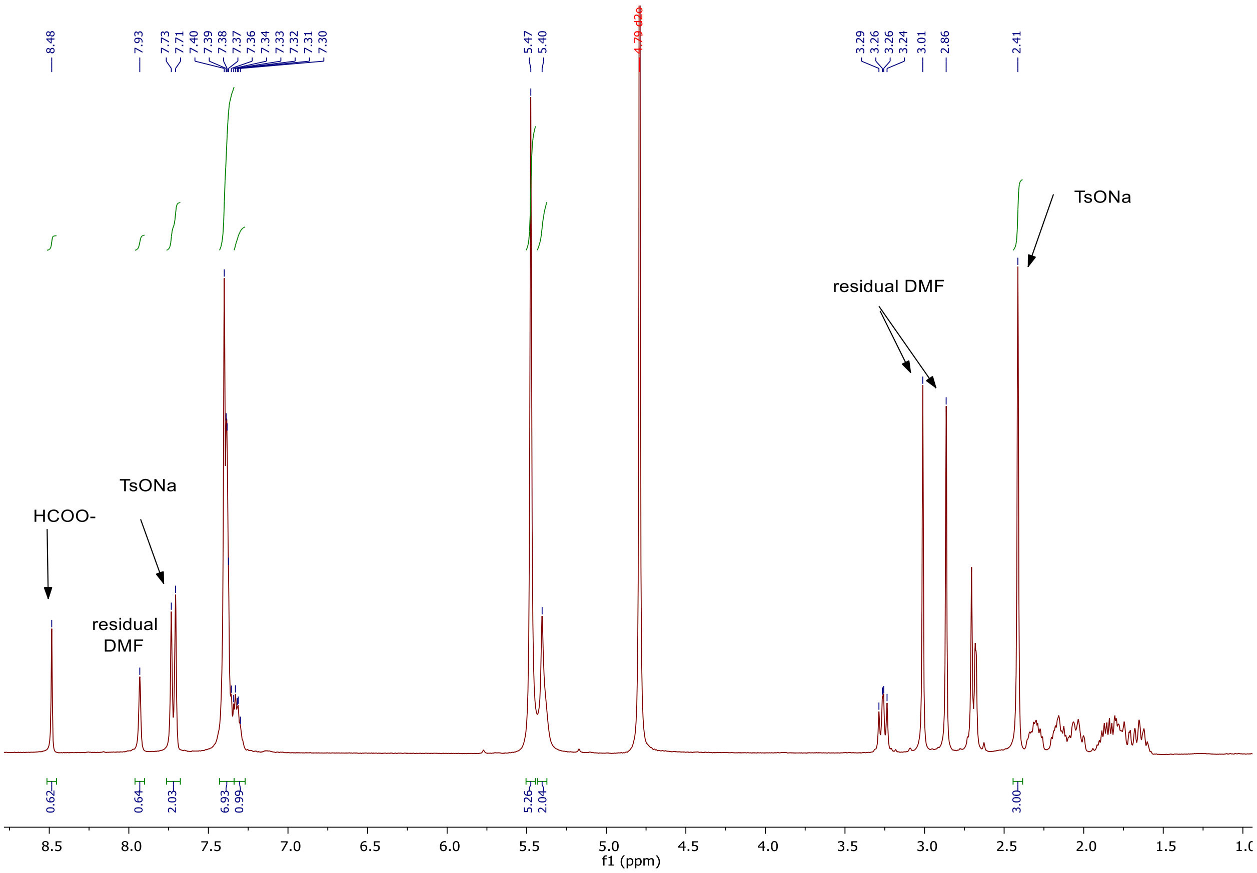

Detection of formate in the model reaction, in the absence of the starting material

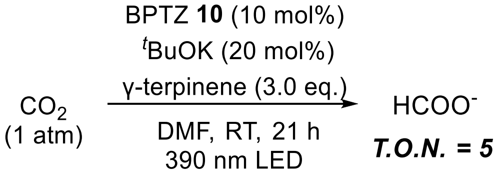

A stock solution of the catalyst mixture was prepared weighing 20 mg of 12*H*-benzo[*b*]phenothiazine and 18 mg of freshly sublimed potassium *tert*-butoxide and accurately dissolved in 4.0 mL of dry DMF (resulting molarity [12*H*-benzo[*b*]phenothiazine] = 0.02 M; ^{t}BuOK = 0.04 M). In a 10 mL Schlenk tube equipped with a stir bar, γ-terpinene (96 µL, 0.6 mmol) and 1.0 mL of the catalyst stock solution was added under air. The solution was subsequently degassed *via* freeze-pump-thaw method (3 cycles) and backfilled with 1 atm CO_2 and irradiated with visible light. *The Schlenk tube was placed in a 3D-printed carousel, resulting in a final distance of 3 cm of the glass wall from the Kessil lamp. The reaction temperature was kept constant using a fan placed on top of the reaction at 20 cm distance.*

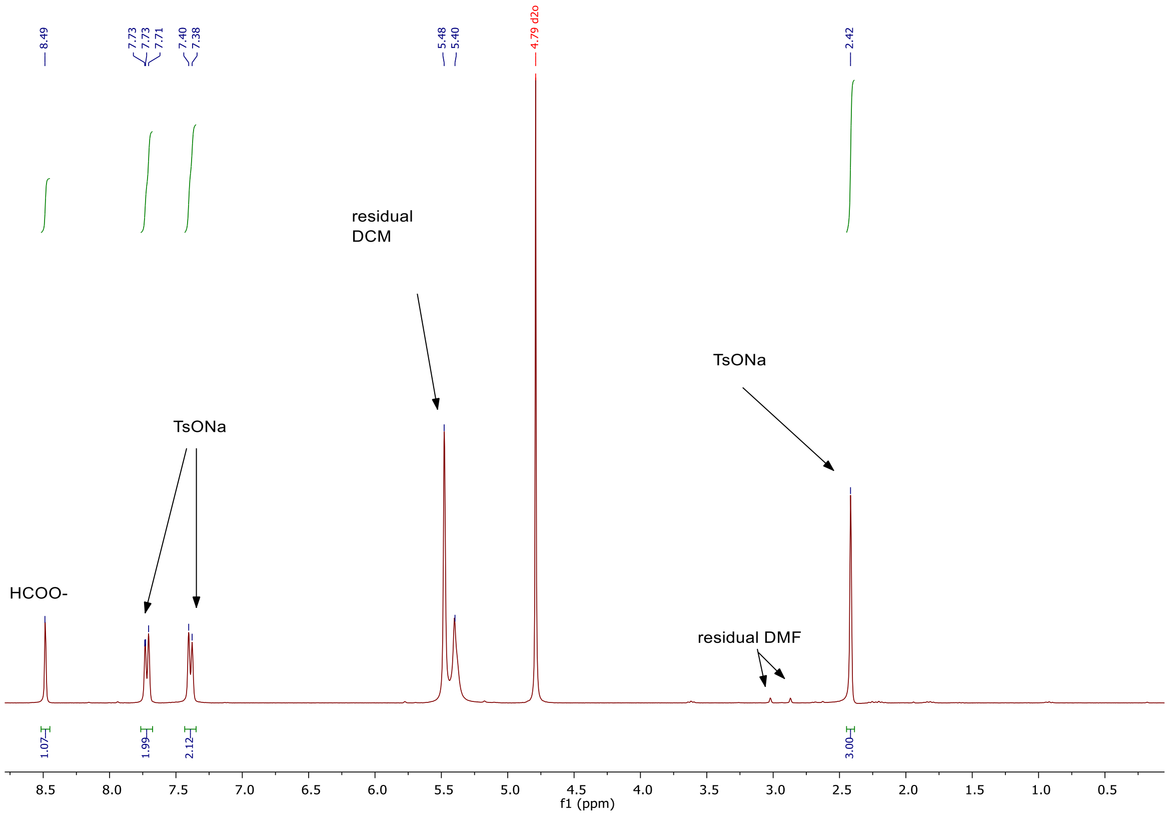

At the end of the reaction, 50 mg of ^{t}BuOK were added to the reaction mixture, to be sure to convert all to carboxylate or formate species to the corresponding potassium salt and stirred for 5 minutes. Then 2 mL of a 0.05M solution of sodium *para*-toluensulfonate in water was added to the reaction mixture. The resulting solution was evaporated to remove the solvents, then the resulting crude was dissolved in DCM and D_2O and the water phase collected and analyzed by ^{1}H-NMR and ^{13}C-NMR to provide detection and quantification of formate yield. **Formate was detected** in the ^{1}H-NMR of the D_2O phase (peak at 8.48, referencing residual water at 4.79ppm, with **T.O.N = 5** based on the amount of photocatalyst employed). This observation is consistent with the fact that in the absence of the starting material, and in the presence of an HAT agent the generation of formate is favored.

Detection of formate in the model reaction, in the dark

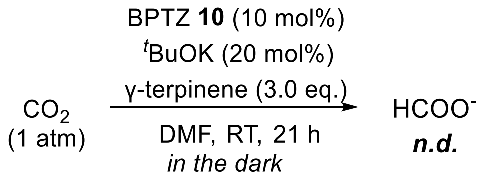

A stock solution of the catalyst mixture was prepared weighing 20 mg of 12*H*-benzo[*b*]phenothiazine and 18 mg of freshly sublimed potassium *tert*-butoxide and accurately dissolved in 4.0 mL of dry DMF (resulting molarity [12*H*-benzo[*b*]phenothiazine] = 0.02 M; ^{t}BuOK = 0.04 M). In a 10 mL Schlenk tube equipped with a stir bar, γ-terpinene (96 μL, 0.6 mmol) and 1.0 mL of the catalyst stock solution was added under air. The solution was subsequently degassed *via* freeze-pump-thaw method (3 cycles) and backfilled with 1 atm CO_2.

The Schlenk tube was covered with aluminum foil and let to stir for 21 hours. At the end of the reaction, 62 µL of Et_3N were added to the reaction mixture, to be sure to convert all to carboxylate or formate species to the corresponding ammonium salt and stirred for 5 minutes. Then 2 mL of a 0.05M solution of sodium *para*-toluensulfonate in water was added to the reaction mixture. The resulting solution was evaporated to remove the solvents, then the resulting crude was dissolved in $CDCl_3$ and D_2O and the water phase collected and analyzed by NMR.

Formate was not detected in the ^{1}H-NMR of the D_2O phase. These results confirm that γ-terpinene is not a hydride donor under our reaction conditions, excluding any possible generation of formate directly from CO_2.

Detection of formate in the model reaction, in the absence of CO_2

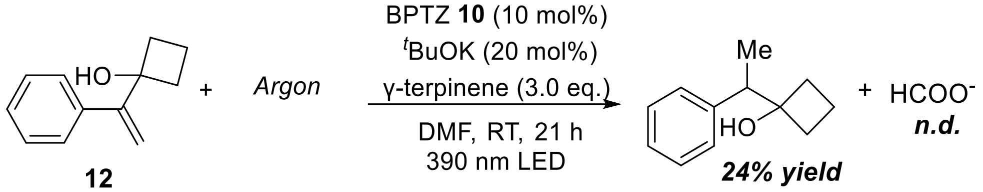

A stock solution of the catalyst mixture was prepared weighing 20 mg of 12*H*-benzo[*b*]phenothiazine and 18 mg of freshly sublimed potassium *tert*-butoxide and accurately dissolved in 4.0 mL of dry DMF (resulting molarity [12*H*-benzo[*b*]phenothiazine] = 0.02 M; ^{t}BuOK = 0.04 M). In a 10 mL Schlenk tube equipped with a stir bar, 1-(1-phenylvinyl)cyclobutan-1-ol (0.2 mmol, 1.0 equiv.), γ-terpinene (96 µL, 0.6 mmol, 3.0 equiv.) and 1.0 mL of the catalyst stock solution was added under air. The solution was subsequently degassed *via* freeze-pump-thaw method (3 cycles) and backfilled with Ar (instead of CO_2) and irradiated with visible light. No turning in color was observed. *The Schlenk tube was placed in a 3D-printed carousel, resulting in a final distance of 3 cm of the glass wall from the Kessil lamp. The reaction temperature was kept constant using a fan placed on top of the reaction at 20 cm distance.* At the end of the reaction, 50 mg of ^{t}BuOK were added to the reaction mixture, to be sure to convert all to carboxylate or formate species to the corresponding potassium salt and stirred for 5 minutes. Then 2 mL of a 0.05M solution of sodium *para*-toluensulfonate in water was added to the reaction mixture. The resulting solution was evaporated to remove the solvents, then the resulting crude was dissolved in $CDCl_3$ and D_2O and the water phase collected and analyzed by NMR.

Formate was not detected in the ^{1}H-NMR of the D_2O phase. However, the ^{1}H-NMR in $CDCl_3$ showed the formation of 1-(1-phenylethyl)cyclobutan-1-ol, the product of hydrogenation of the double bond isolated in 24% yield.

1-(1-phenylethyl)cyclobutan-1-ol (85): ^{1}H NMR (300 MHz, $CDCl_3$) δ: 7.35 – 7.27 (m, 4H), 7.25 – 7.21 (m, 1H), 2.94 (1H, q, *J* = 7.2 Hz, 4), 2.29 – 2.20 (m, 1H), 2.18 – 2.10 (m, 1H), 2.07 – 1.98 (m, 1H), 1.92-1.81 (m, 1H), 1.80 – 1.71 (m, 1H), 1.67 – 1.51 (m, 1H), 1.32 (d, *J* = 7.2 Hz, 3H), consistent with previous reports.[31]

---

[31] R.C. Mykura, P. Songara, E. Luc, J. Rogers, E. Stammers, V. K. Aggarwal *Angew. Chem. Int. Ed.* **2021**, *60*, 11436 – 11441

Detection of formate in the model reaction, in the absence of the starting material and CO_2

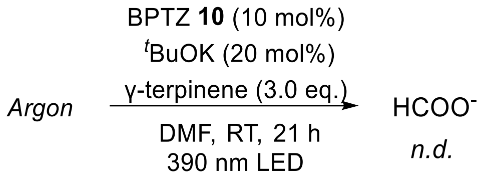

A stock solution of the catalyst mixture was prepared weighing 20 mg of 12*H*-benzo[*b*]phenothiazine and 18 mg of freshly sublimed potassium *tert*-butoxide and accurately dissolved in 4.0 mL of dry DMF (resulting molarity [12*H*-benzo[*b*]phenothiazine] = 0.02 M; ^{t}BuOK = 0.04 M). In a 10 mL Schlenk tube equipped with a stir bar, γ-terpinene (96 µL, 0.6 mmol) and 1.0 mL of the catalyst stock solution was added under air. The solution was subsequently degassed *via* freeze-pump-thaw method (3 cycles) and backfilled with Ar (instead of CO_2) and irradiated with visible light. No turning in color was observed. *The Schlenk tube was placed in a 3D-printed carousel, resulting in a final distance of 3 cm of the glass wall from the Kessil lamp. The reaction temperature was kept constant using a fan placed on top of the reaction at 20 cm distance.*

At the end of the reaction, 62 µL of Et_3N were added to the reaction mixture, to be sure to convert all to carboxylate or formate species to the corresponding ammonium salt and stirred for 5 minutes. Then 2 mL of a 0.05M solution of sodium *para*-toluensulfonate in water was added to the reaction mixture. The resulting solution was evaporated to remove the solvents, then the resulting crude was dissolved in DCM and D_2O and the water phase collected and analyzed by ^{1}H-NMR and ^{13}C-NMR. In this case, **no formate was detected.**

Detection of formate in the model reaction, in the absence of γ-terpinene

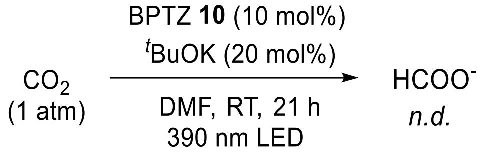

A stock solution of the catalyst mixture was prepared weighing 20 mg of 12*H*-benzo[*b*]phenothiazine and 18 mg of freshly sublimed potassium *tert*-butoxide and accurately dissolved in 4.0 mL of dry DMF (resulting molarity [12*H*-benzo[*b*]phenothiazine] = 0.02 M; ^{t}BuOK = 0.04 M). In a 10 mL Schlenk tube equipped with a stir bar and 1.0 mL of the catalyst stock solution was added under air. The solution was subsequently degassed *via* freeze-pump-thaw method (3 cycles) and backfilled with 1 atm CO_2 and irradiated with visible light. *The Schlenk tube was placed in a 3D-printed carousel, resulting in a final distance of 3 cm of the glass wall from the Kessil lamp. The reaction temperature was kept constant using a fan placed on top of the reaction at 20 cm distance.*

At the end of the reaction, 62 µL of Et_3N were added to the reaction mixture, to be sure to convert all to carboxylate or formate species to the corresponding ammonium salt and stirred for 5 minutes. Then 2 mL of a 0.05M solution of sodium *para*-toluensulfonate in water was added to the reaction mixture. The resulting solution was evaporated to remove the solvents, then the resulting crude was dissolved in DCM and D_2O and the water phase collected and analyzed by ^{1}H-NMR and ^{13}C-NMR. In this case, **no formate was detected.**

Control experiment using sodium formate instead of CO_2

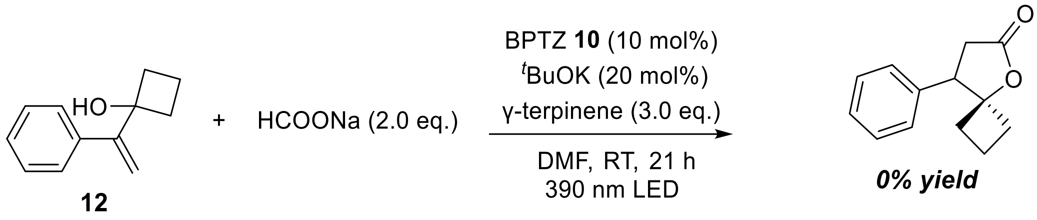

To completely rule out the possibility of formate as source of $CO_2^{\bullet-}$, we have performed a control experiment using General Procedure H where we replaced the CO_2 with 2.0 eq. of sodium formate (HCOONa). After the reaction, **no spirolactone was detected** confirming that formate is not an intermediate in the reaction for the generation of $CO_2^{\bullet-}$.

Radical Clock Experiment

Synthesis of starting materials for Radical Clock experiment

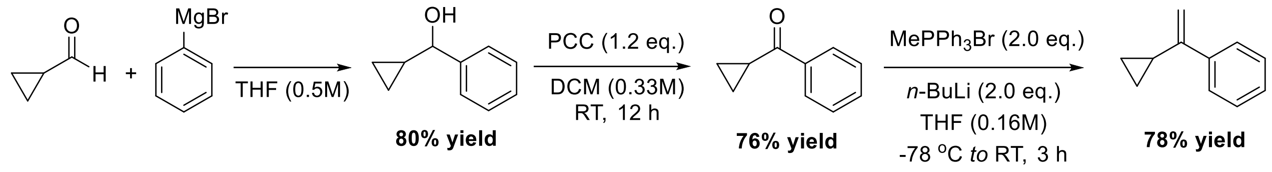

1st step. cyclopropyl(phenyl)methanol: In a 50 mL round bottom flask equipped with stir bar, cyclopropanecarboxaldehyde (0.38 mL, 5.0 mmol) was dissolved in anhydrous MeOH (10 mL) under argon atmosphere, followed by the dropwise addition of phenylmagnesium bromide (2 mL, 6.0 mmol, 3.0 M in Et_2O) at 0°C and the resulting mixture was stirred at room temperature for 1h and saturated NH_4Cl (aq.) was added to quench to reaction. The reaction mixture was then extracted with diethyl ether (Et_2O) and washed with NaCl saturated aqueous solution. The organic extract was then dried using $MgSO_4$ or Na_2SO_4, filtered and concentrated *in vacuo.* The crude was purified using flash column chromatography (silica gel) (93/07 to 89/11 of Hexane/EtOAc) to provide the titled compound as a colorless liquid (592 mg, 80%). ^{1}H NMR (300 MHz, $CDCl_3$) δ 7.47 – 7.27 (m, 5H), 4.02 (d, J = 8.3 Hz, 1H), 1.99 (s, 1H), 1.23 (qt, J = 8.1, 4.9 Hz, 1H), 0.71 – 0.31 (m, 4H). The ^{1}H NMR data is in accordance with the literature.[32]

2nd step. **cyclopropyl(phenyl)methanone**: In a 50 mL round bottom flask equipped with stir bar, cyclopropyl(phenyl)methanol (592 mg, 4.0 mmol) was dissolved in anhydrous DCM (12 mL) under argon atmosphere, followed by the addition of pyridinium chlorochromate (PCC) (1.03 mg, 4.8 mmol) and the resulting mixture was stirred at room temperature for 12 hours and the reaction mixture was then filtered through a pad of celite and concentrated *in vacuo.* The crude was purified using flash column chromatography (silica gel) (98/02 to 97/03 of Hexane/EtOAc) to provide the titled compound as a colorless liquid (444 mg,

[32] Wang, S.; Huang, H.; Tsareva, S.; Bruneau, C.; Fischmeister, C. *Adv. Synth. Catal.* **2019**, *361*, 786-790.

76%). ^{1}H NMR (300 MHz, $CDCl_3$) δ 8.02 (dd, *J* = 7.2, 1.6 Hz, 2H), 7.61 – 7.52 (m, 1H), 7.52 – 7.41 (m, 2H), 2.68 (ddt, *J* = 12.5, 8.2, 4.6 Hz, 1H), 1.24 (dt, *J* = 7.1, 3.3 Hz, 2H), 1.04 (dq, *J* = 7.4, 3.6 Hz, 2H). The ^{1}H NMR data is in accordance with the literature.[33]

3rd step. **(1-cyclopropylvinyl)benzene (81)**: A 50 mL round bottom flask was charged with methyltriphenylphosphonium bromide (2.15 g, 6.0 mmol) and placed under argon atmosphere. Anhydrous THF (19 mL) was added, and the mixture was cooled to -78°C. While stirring, *n*-BuLi (2.5 M in hexanes, 2.41 mL, 6.0 mmol) was added dropwise. After 10 minutes of stirring, cyclopropyl phenyl ketone (440 mg, 3.0 mmol) as solution in DCM was added dropwise. After stirring the solution for 3 hours at room temperature, the reaction mixture was quenched by the addition of saturated NH_4Cl (aq.). The reaction mixture was then extracted with diethyl ether (Et_2O) and washed with NaCl saturated aqueous solution. The organic extract was then dried using $MgSO_4$ or Na_2SO_4, filtered and concentrated *in vacuo.* The crude was purified using flash column chromatography (silica gel) (98/02 of Hexane/EtOAc) to provide the titled compound as a colorless liquid (337 mg, 78%). ^{1}H NMR (300 MHz, $CDCl_3$) δ 7.65 – 7.52 (m, 2H), 7.44 – 7.18 (m, 3H), 5.28 (d, *J* = 1.1 Hz, 1H), 4.94 (d, *J* = 1.4 Hz, 1H), 1.65 (tt, *J* = 8.5, 4.2 Hz, 1H), 0.90 – 0.78 (m, 2H), 0.65 – 0.53 (m, 2H). The ^{1}H NMR data is in accordance with the literature.[34]

Radical Clock Experiment

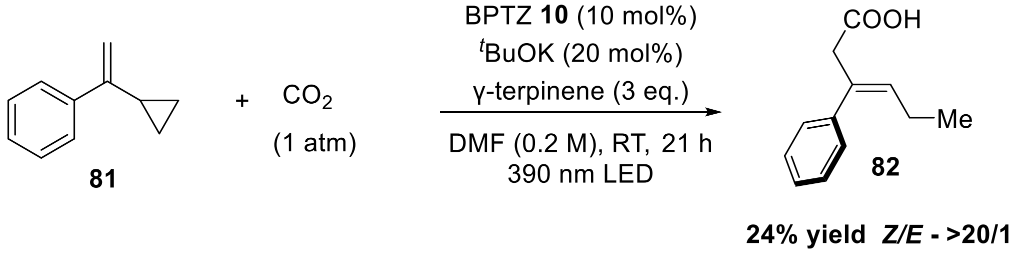

According to general procedure **H**, (1-cyclopropylvinyl)benzene **81** (29 mg, 0.2 mmol), 12*H*-benzo[*b*]phenothiazine (5 mg, 0.02 mmol), potassium *tert*-butoxide (4.5 mg, 0.04 mmol), γ-terpinene (96 µL, 0.6 mmol) were dissolved in anhydrous DMF (1.0 mL) in a 10 mL Schlenk tube. Three freeze-pump-thaw cycles were performed, and CO_2 was then introduced *via* backfilling. The Schlenk tube was irradiated with 390 nm light for 21 hours. The crude was purified using flash column chromatography (85/15 of hexane/EtOAc) to provide the ring opening product **82** as a pale-yellow liquid (9 mg, 24%). ^{1}H NMR (300 MHz, $CDCl_3$) δ 7.27 (m, 5H), 5.64 (t, *J* = 7.4 Hz, 1H), 3.37 (s, 2H), 2.02 (p, *J* = 7.5 Hz, 2H), 0.95 (t, *J* = 7.5 Hz, 3H). The ^{1}H NMR data is in accordance with the literature.[35] The formation of the ring opened carboxylate product demonstrates the open-shell nature of our reaction.

---

[33] Neff, R.-K.; Su. Y.-L.; Liu, S.; Rosado, M.; Zhang, X.; Doyle, M. P. *J. Am. Chem. Soc.* **2019**, *141*, 16643–16650.
[34] Bellotti, P.; Huang, H.-M.; Faber, T.; Laskar, R.; Glorius, F. *Chem. Sci.* **2022**, *13*, 7855-7862.
[35] Huang, H.; Ye, J.-H.; Zhu, L.; Ran, C.-K.; Miao, M.; Wang, W.; Chen, H.; Zhou, W.-J.; Lan, Y.; Yu, B.; Yu, D.-G. *CCS Chem.* **2021**, *3*, 1746–1756.

Control experiments using a BPTZ that is unable to generate the carbamate

Detection of formate in the model reaction, using a BPTZ that is unable to generate the carbamate, in the absence of starting material

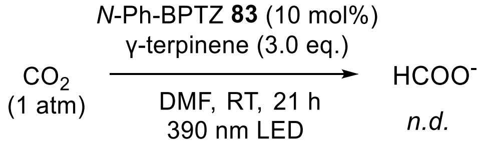

In a 10 mL Schlenk tube equipped with a stir bar, γ-terpinene (96 µL, 0.6 mmol), N-Ph-BPTZ **83** (6.5 mg, 0.02 mmol) and 1.0 mL of DMF. The solution was subsequently degassed *via* freeze-pump-thaw method (3 cycles) and backfilled with 1 atm CO_2 and irradiated with visible light (390 nm LED). *The Schlenk tube was placed in a 3D-printed carousel, resulting in a final distance of 3 cm of the glass wall from the Kessil lamp. The reaction temperature was kept constant using a fan placed on top of the reaction at 20 cm distance.*
At the end of the reaction, 62 µL of Et_3N were added to the reaction mixture, to be sure to convert all to carboxylate or formate species to the corresponding ammonium salt and stirred for 5 minutes. Then 2 mL of a 0.05M solution of sodium *para*-toluensulfonate in water was added to the reaction mixture. The resulting solution was evaporated to remove the solvents, then the resulting crude was dissolved in DCM and D_2O and the water phase collected and analyzed by ^{1}H-NMR and ^{13}C-NMR to provide detection and quantification of formate yield. In this case, **no formate was detected.**

Detection of formate in the hydrocarbozylation reaction of benzothiophene, using a BPTZ that is unable to generate the carbamate

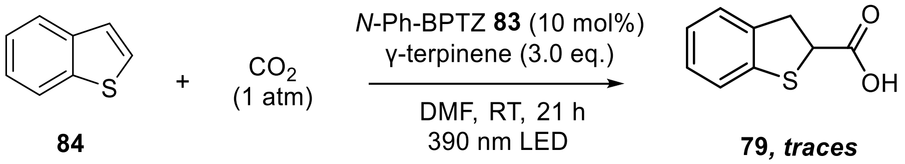

In a 10 mL Schlenk tube equipped with a stir bar were added benzothiophene **84** (0.2 mmol), γ-terpinene (96 µL, 0.6 mmol), N-Ph-BPTZ **83** (6.5 mg, 0.02 mmol) and 1.0 mL of DMF. The solution was subsequently degassed *via* freeze-pump-thaw method (3 cycles) and backfilled with 1 atm of CO_2 and irradiated with visible light (390 nm LED). *The Schlenk tube was placed in a 3D-printed carousel, resulting in a final distance of 3 cm of the glass wall from the Kessil lamp. The reaction temperature was kept constant using a fan placed on top of the reaction at 20 cm distance.* After the reaction time, a workup was done according to general procedure **H** and the ^{1}H NMR of the crude was performed. Only traces of product were obtained.
This experiment, taken together with the results in the previous paragraph (no formate detected when the reaction is conducted with N-Ph-BPTZ **83**), clearly support the crucial role of the carbamate and the proposed photolysis for the formation of the carboxylate product.

Quantum yield determination

Determination of photon flux

The photon flux was determined by standard ferrioxalate actinometry. Ferrioxalate actinometer solution was prepared according to literature procedure.[36] The ferrioxalate actinometer solution measures the decomposition of ferric ions to ferrous ions, which are complexed by 1,10-phenanthroline and monitored by UV/Vis absorbance at 510 nm. The moles of iron-phenanthroline complex formed are related to moles of photons absorbed.

The solutions were prepared and stored in the dark:

1. Potassium ferrioxalate solution: 294.7 mg of potassium ferrioxalate trihydrate (commercially available from Thermo Scientific) and 139 μL of sulfuric acid (95%) were added to a 50 mL volumetric flask and filled to the mark with water (milli Q grade).
2. Phenantroline solution: 0.2% by weight of 1,10-phenanthroline in water (100 mg in 50 mL volumetric flask).
3. Buffer solution: to a 50 mL volumetric flask, 2.47 g of NaOAc and 0.5 mL of sulfuric acid (95%) were added and filled to the mark with water (milli Q grade).

The actinometry measurements was done as follows:

1. 1 mL of the actinometer solution was added to a 10 mL Schlenk tube. The Schlenk tube was placed 3 cm away from the light source. It was irradiated for 30 seconds with a 40 W Kessil PR160L-390 nm Lamp operating at 100% of light intensity.
2. After irradiation all the actinometer solution was removed and placed in a 10 mL volumetric flask. 0.5 mL of 1,10-phenanthroline solution and 2 mL of buffer solution was added to this flask and filled to the mark with water (milli Q grade).
3. The UV-Vis spectrum of the complexed actinometer sample was recorded. The absorbance of the complexed actinometer solution was monitored at 510 nm.

The moles of Fe^{2+} formed is determined using the following Beers' Law:

$$\text{Moles of Fe}^{2+} = \frac{(V_1 \times V_3 \times \Delta A_{510\,nm})}{(10^3 \times V_2 \times l \times \varepsilon_{510\,nm})}$$

where V_1 is the irradiated volume (1 mL), V_2 is the aliquot of the irradiated solution taken for the determination of the ferrous ions (1 mL), V_3 is the final volume after complexation with phenanthroline (10 mL), l is the optical path-length of the irradiation cell (1 cm), ΔA (510 nm) the optical difference in absorbance between the irradiated solution and that taken in the dark, ε (510 nm) is that of the complex $Fe(phen)_3^{2+}$ (11100 L mol^{-1} cm^{1}). The optical difference ΔA (510 nm) was calculated from the experimental values from the absorption spectra and it was found out to be 1.3116. So, the **final moles of Fe^{2+}** are calculated to be **1.1182 x 10^{-6} moles**.

---

[36] S. Murov, L., *Handbook of Photochemistry*, Marcel Dekker, New York, **1973**.

The photon flux is then calculated using the following equation:

$$\text{photon flux} = \frac{\text{mol Fe}^{2+}}{\Phi \bullet t \bullet f}$$

Where Φ is the quantum yield for the ferrioxalate actinometer (~1.13 at λ = 390 nm)[37]; t is the time (30 secs), and f is the fraction of light absorbed at λ = 390 nm (~1.0).
The photon flux was calculated to be **3.4857 × 10^{-8} einstein s^{-1}.**

Determination of quantum yield:

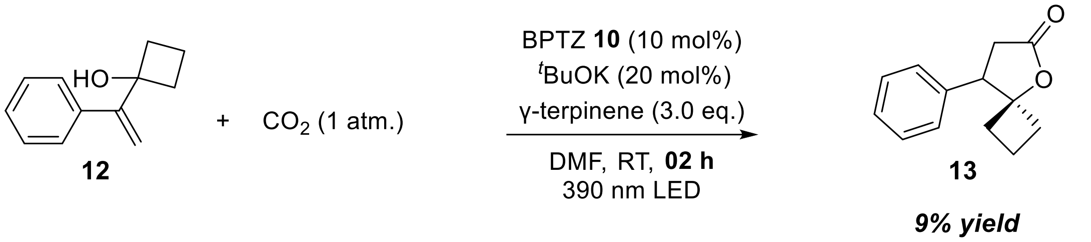

A stock solution of the catalyst mixture was prepared weighing 20 mg of 12*H*-benzo[*b*]phenothiazine and 18 mg of freshly sublimed potassium *tert*-butoxide and accurately dissolved in 4.0 mL of dry DMF (resulting molarity [12*H*-benzo[*b*]phenothiazine] = 0.02 M; ^{t}BuOK = 0.04 M). In a 10 mL Schlenk tube, 1-(1-phenylvinyl)cyclobutan-1-ol (0.2 mmol, 1.0 equiv.), γ-terpinene (96 uL, 0.6 mmol, 3.0 equiv.) and 1.0 mL of the catalyst stock solution was added under air. The solution was subsequently degassed *via* freeze-pump-thaw method (3 cycles) and backfilled with 1 atm of CO_2. The reaction mixture turned from red to yellow after backfilling. The Schlenk tube was placed 3 cm away from the light source. It was irradiated for 7200 secs (2 h) with a 40 W Kessil PR160L-390 nm Lamp operating at 100% of light intensity. After irradiation, workup was performed according to general procedure **H** and the ^{1}H NMR yield of the reaction was determined using methyl 3,5-dinitrobenzoate as the internal standard. The reaction yielded 9% of the desired product.
With this data in hand, the quantum yield of the reaction was measured using the following equation:

$$\Phi = \frac{\text{mol product}}{\text{flux} \bullet t \bullet f}$$

The quantum yield Φ was determined to be 0.07, indicating that no possible chain reaction was operative under the reaction conditions and continuous irradiation was required for the generation of CO_2 radical anion species.

[37] E. E. Wegner, A. W. Adamson, *J. Am. Chem. Soc.* **1966**, *88*, 394–404.

Comparison with previously reported β-hydrocarboxylation methodologies

We tested whether established β-selective hydrocarboxylation methods with CO_2 were competent in converting hindered substrate **12** into spirolactone **13**.

Two different reported methodologies were considered:

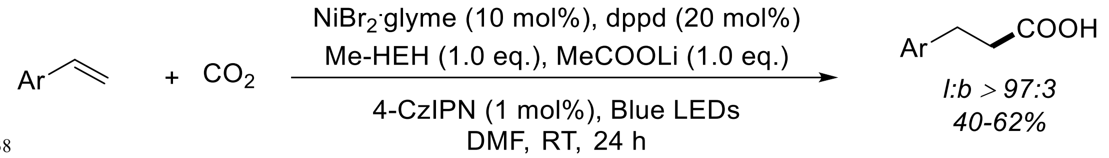

B. Konig[38]

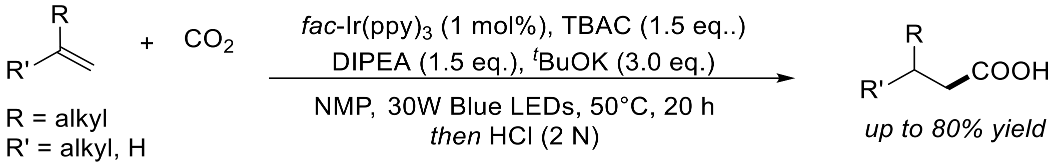

D.G. Yu[39]

1) Konig's conditions

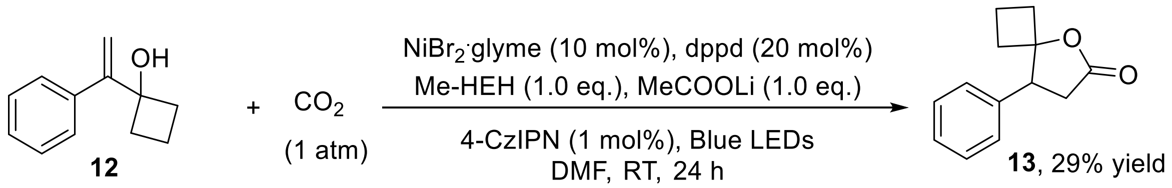

In a 10 mL flame-dried Schlenk tube were added in one portion, under Argon atmosphere, all five solid reagent were added: $NiBr_2$·glyme (6.17 mg, 0.02 mmol, 0.10 equiv.), dppd (14.25, 0.04 mmol, 0.40 equiv.) methyl Hantzsch Ester (53.47 mg, 0.200 mmol, 1.0 eq), lithium acetate (13.20 mg, 0.200 mmol, 1.0 equiv.) and 4-CzIPN (1.53 mg, 0.002 mmol, 0.01 equiv.). Then a solution of 1-(1-phenylvinyl)cyclobutan-1-ol **12** (35 mg, 0.2 mmol, 1.0 equiv.) in dry DMF (2.0 mL) was prepared and added under argon to the Schlenk tube. Then three freeze-pump-thaw cycles were performed, and CO_2 was then introduced *via* backfilling, as described in section 4. The Schlenk tube was irradiated with 456nm light Kessil Blue Light for 24 hours and the crude, after being subjected to the workup A of general procedure H was then analyzed by NMR showing 29% yield of product **13** and recovery of 48% of starting material **12.**

Note: *To ensure the repeatability of this protocol in our laboratory setup, we tried the reaction using styrene as starting material. Reported yield of styrene hydrocarboxylation product = 59% vs observed yield in 10 mL Schlenk vial with freeze-pump-thaw (our conditions and setup) = 60%.*

---

[38] Q. Y. Meng, S. Wang, G. S. Huff, B. König *J. Am. Chem. Soc.* **2018**, *140*, 3198-3201

[39] L. Song, W. Wang, J.P. Yue, Y.X. Jiang, M.K. Wei, H.P. Zhang, S.S. Yan, L.L. Liao, D.G. Yu *Nat. Catal.* **2022**, *5*, 832-838

2) D. G. Yu's conditions

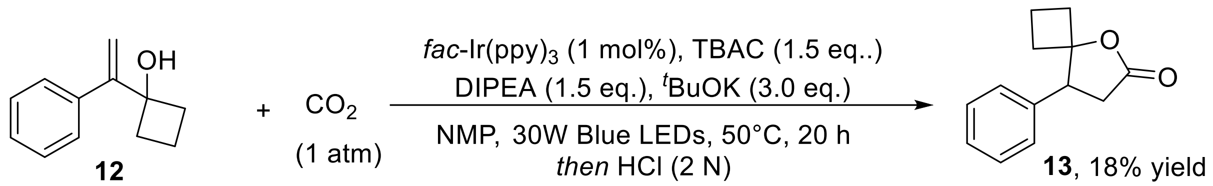

In a 10 mL flame-dried Schlenk tube were added in one portion, under Argon atmosphere, all the solid reagents were added *fac*-Ir(ppy)$_3$ (1.4 mg, 0.002 mmol, 0.01 equiv.), TBACl (85 mg, 0.300 mmol, 1.50 equiv.) and freshly sublimed ^{t}BuOK (70 mg, 0.600 mmol, 3.0 equiv). Then a solution of 1-(1-phenylvinyl)cyclobutan-1-ol **S2** (35 mg, 0.200 mmol, 1.0 equiv.) in dry NMP (1.0 mL) was prepared and added under argon to the Schlenk tube. Finally, DIPEA (52 µL, 39 mg, 0.300 mmol, 1.5 equiv.) was added to the resulting mixture. Then three freeze-pump-thaw cycles were performed, and CO_2 was then introduced *via* backfilling, as described in section 4. The Schlenk tube was submerged in a colorless oil bath at 50°C and irradiated with 456nm light Kessil Blue Light for 48 h (see the last comment below for explanation about this longer time). At then end of the reaction the crude was subjected to the workup A of general procedure H and was then analyzed by NMR showing 18% yield of product **13** and recovery of 35% of starting material **12.**

Note: *To ensure the repeatability of this protocol in our laboratory setup, we tried the reaction using allylbenzene as starting material. Reported yield of allylbenzene carboxylation product = 59% vs observed yield in 10 mL Schlenk vial with freeze-pump-thaw (our conditions and setup) = i) 34% in 24 hours with uncomplete conversion ii) 50% in 48 hours, with complete conversion of allylbenzene starting material. Given these results, we used 48 hours as reaction time.*

Literature reference	conditions and setup	Remaining starting material **12**	Product **13** (% yield)
Our method	**General procedure H**	0%	75%
B. Konig *et al.* [38]	**1)**	48%	29%
D.G. Yu *et al.* [39]	**2)**	35%	18%

The results summarized in the table demonstrate the superiority of our protocol in the hydrocarboxylation of hindered substrate **12**.

6. DFT studies

Computational details

All electronic structure calculations were performed using the Gaussian 16 software package[40] at the CESGA facilities. Potential energy surfaces (PESs) were explored for the ground state (S_0), the lowest triplet state (T_1), and the lowest singlet excited state (S_1). Geometry optimizations of all minima and transition states on these surfaces were carried out using the wB97X-D functional,[41] in conjunction with the 6-31+G basis set.[42]

The S_1 excited state was optimized by time-dependent density functional theory DFT (TD-DFT)[43] at the same level of theory, starting from the corresponding ground-state geometry. Frequency calculations were performed at the same level of theory for all stationary points (on S_0, T_1, and S_1) to confirm their nature, yielding zero imaginary frequencies for minima and one for each transition state, and to compute zero-point vibrational energies and thermal corrections to the Gibbs free energy at 298 K.

Each transition state was further validated by intrinsic reaction coordinate (IRC) calculations to verify its connection to the appropriate reactants and products. Single point energy calculations were performed using the wB97X-D functional with the 6-31G basis set, employing the self-consistent reaction field (SCRF) using the SMD implicit solvation model for *N*,*N*-dimethylformamide (DMF).[44] The resulting solvent-corrected energies were used to refine the gas-phase wB97X-D results, and reaction profiles were constructed in terms of DG_{sol}.

The minimum-energy crossing point (MECP) between the S_1 and T_1 excited states was located using the KST48 code[45] in combination with Gaussian 16. Calculations were performed with the *mode=stable* keyword and the wB97X-D functional with the 6-31G basis set. Solvent effects were included *via* the SCRF/SMD model for *N*,*N*-dimethylformamide (DMF). The S_1 excited state was described using TD-DFT at the same level of theory.

---

40 M. J. Frisch *et al.*, *Gaussian 16, Revision C.01*, Gaussian, Inc., Wallingford CT, 2016

41 a) J.-D. Chai, M. Head-Gordon, *Phys. Chem. Chem. Phys.* **2008**, *10*, 6615; b) J.-D. Chai, M. Head-Gordon, *J. Chem. Phys.* **2008**, *128*, 084106.

42 a) R. Ditchfield, W. J. Hehre, J. A. Pople, *J. Chem. Phys.* **1971**, *54*, 724; b) W. J. Hehre, R. Ditchfield, J. A. Pople, *J. Chem. Phys.* **1972**, *56*, 2257; c) M. M. Francl, W. J. Pietro, W. J. Hehre, J. S. Binkley, M. S. Gordon, D. J. DeFrees, J. A. Pople, *J. Chem. Phys.* **1982**, 77, 3654; d) M. S. Gordon, J. S. Binkley, J. A. Pople, W. J. Pietro, W. J. Hehre, *J. Am. Chem. Soc.* **1982**, *104*, 2797; e) G. W. Spitznagel, T. Clark, P. von Ragué Schleyer, W. J. Hehre, *J. Comput. Chem.* **1987**, *8*, 1109.

43 M. E. Casida, in "Time-Dependent Density Functional Response Theory for Molecules", *Recent Advances in Density Functional Methods, Vol. Volume 1*, WORLD SCIENTIFIC, **1995**, pp. 155.

44 A. V. Marenich, C. J. Cramer, D. G. Truhlar, *J. Phys. Chem. B* **2009**, *113*, 6378.

45 a) Y. Ma, A. A. Hussein, *ChemistrySelect* **2022**, *7*, e202202354; b) M. Yumiao. KST48: A Powerful Tool for MECP locating https://github.com/RimoAccelerator/KST48, accessed on 2025.09.01.

Free Energy Profile for the Formation of 4 and $CO_2^{\bullet-}K^+$

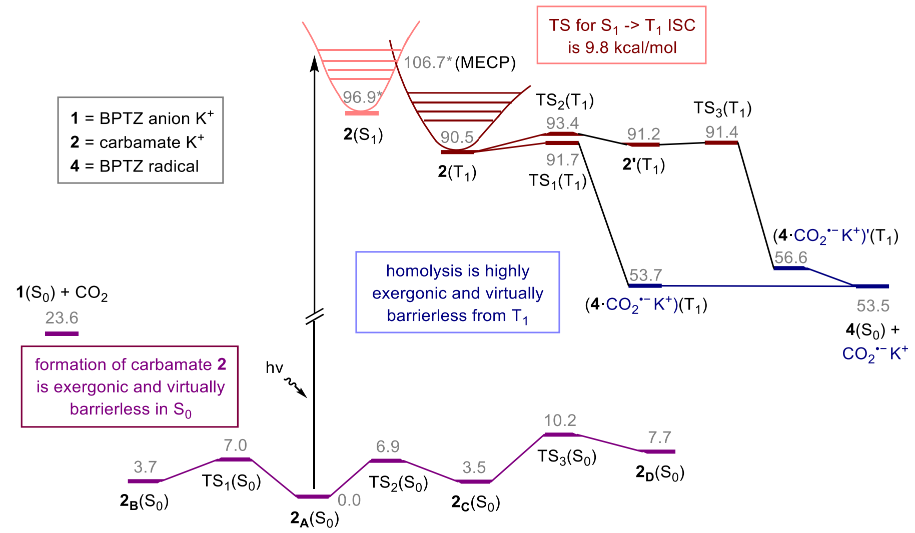

Figure: Computed free energy profile for the formation of potassium carbamate **2** in the ground state (S_0, violet), photoinduced excitation to the singlet excited state (S_1, pink), intersystem crossing to triplet excited state (T_1, red), and subsequent evolution to form **4** and $CO_2^{\bullet-}K^+$. Computational studies were performed at wB97X-D/6-31G(SMD=DMF)// wB97X-D/6-31G theory level. All energies are DG_{sol} reported in kcal mol^{-1} relative to $\mathbf{2_A}(S_0)$. Energy values marked with * correspond to electronic energies (DE_{sol}) reported in kcal mol^{-1} relative to $\mathbf{2_A}(S_0)$. BPTZ = benzophenothiazine. hv = photon. MECP = minimum energy crossing point.

Cartesian Coordinates

Cartesian Coordinates in Å of all intermediates and transition states involved in the formation of **4** and $\mathbf{CO_2^{\bullet-}K^+}$ in the ground state (S_0), singlet excited state (S_1) and triplet excited state (T_1).

a) Cartesian Coordinates of Intermediates and Transition States in S_0

CO_2

Electronic Energy ωB97X-D/6-31G = -188.434155693 Hartree

Electronic Energy ωB97X-D/6-31G(SMD=DMF) = -188.437104889 Hartree

Zero-point Energy Correction = 0.010909 Hartree

Thermal Correction to Enthalpy = 0.014572 Hartree

Thermal Correction to Free Energy = -0.009864 Hartree

Chemical symbol X, Y, Z

C0.0000000.0000000.000000

O0.0000000.0000001.182168

O0.0000000.000000-1.182168

1(S_0)

Electronic Energy ωB97X-D/6-31G = -1668.15690489 Hartree

Electronic Energy ωB97X-D/6-31G(SMD=DMF) = -1668.19712128 Hartree

Zero-point Energy Correction = 0.217008 Hartree

Thermal Correction to Enthalpy = 0.232579 Hartree

Thermal Correction to Free Energy = 0.173268 Hartree

Chemical symbol X, Y, Z

C3.776518-1.5864290.053755

C2.535123-0.973309-0.077785

C2.2910500.3361840.389954

C3.3910671.0121860.962212

C4.6444070.4175031.066270

C4.846659-0.8919560.621618

H3.910458-2.602810-0.303071

H3.2131852.0113561.347528

H5.4641630.9707031.514138

H5.816564-1.3670520.715091

N1.0733091.0022670.319866

S1.216895-1.836317-1.013652

C-0.254399-1.029229-0.286835

C-0.1142770.3120150.232685

C-1.3044750.9558170.601589

H-1.2245341.9120181.117768

C-1.466676-1.652616-0.378422

H-1.525265-2.666265-0.765180

C-2.5824210.3485280.482101

C-2.675014-0.986465-0.013064

C-3.7842671.0112040.858632

H-3.7215722.0211041.255932

C-5.0066460.3867240.739586

H-5.9117490.9065331.037054

C-3.945294-1.602776-0.129519

H-3.999726-2.619096-0.509718

C-5.093511-0.9332960.237182

H-6.061179-1.4147180.148594

K0.2899532.866786-1.249395

$2_B(S_0)$

Electronic Energy ωB97X-D/6-31G = -1856.64836621 Hartree

Electronic Energy ωB97X-D/6-31G(SMD=DMF) = -1856.68884788 Hartree

Zero-point Energy Correction = 0.232844 Hartree

Thermal Correction to Enthalpy = 0.250946 Hartree

Thermal Correction to Free Energy = 0.186329 Hartree

Chemical symbol X, Y, Z

C-2.807278-2.226380-0.771529

C-1.849748-1.347333-0.263576

C-1.745512-0.025626-0.722869

C-2.6604800.412689-1.694787

C-3.641395-0.448816-2.175107

C-3.717094-1.771663-1.723877

H-2.834271-3.252481-0.421521

H-2.6054001.436283-2.040915

H-4.342483-0.091180-2.920751

H-4.468842-2.444546-2.120051

N-0.7442060.829795-0.202032

S-0.722742-1.8569361.089604

C0.766339-0.9688780.481818

C0.5813730.323361-0.089233

C1.6963191.022323-0.503736

H1.5633822.014264-0.912564

C2.004544-1.5405160.611077

H2.108375-2.5377021.026014

C2.9981420.478495-0.345171

C3.163482-0.8243680.209865

C4.1563361.201572-0.748115

H4.0280822.194485-1.166736

C5.4098540.655880-0.607575

H6.2850331.216425-0.917471

C4.472264-1.3643950.343740

H4.589063-2.3576760.766881

C5.570622-0.641419-0.056218

H6.566053-1.0590970.048176
C-1.0685162.2774790.013323
O-0.1463683.105297-0.124171
O-2.2857292.4472380.374911
K-2.8754960.6888902.046599

$TS_1(S_0)$

Imaginary Freq = -47.8082 (cm^{-1})

Electronic Energy ωB97X-D/6-31G = -1856.64581659 Hartree

Electronic Energy ωB97X-D/6-31G(SMD=DMF) = -1856.68468977 Hartree

Zero-point Energy Correction = 0.232794 Hartree

Thermal Correction to Enthalpy = 0.250173 Hartree

Thermal Correction to Free Energy = 0.187393 Hartree

Chemical symbol X, Y, Z

C-2.891252-2.4178620.015445
C-1.948017-1.4022990.162368
C-1.838330-0.362256-0.767385
C-2.724661-0.334490-1.850929
C-3.692079-1.325467-1.985541
C-3.775402-2.372042-1.060926
H-2.930142-3.2292340.733566
H-2.6592660.484691-2.553788
H-4.379347-1.288659-2.823142
H-4.518929-3.151538-1.182312
N-0.8333070.625775-0.592017
S-0.822194-1.3547491.614271
C0.675516-0.7895880.710433
C0.4880780.192663-0.301315
C1.5927630.698358-0.948242
H1.4588621.469920-1.694522
C1.917589-1.2702421.034056
H2.030802-2.0410961.789203
C2.8985980.256922-0.612093
C3.069127-0.7514450.383536
C4.0527900.782666-1.258639
H3.9207971.553469-2.011095
C5.3082910.327009-0.935961
H6.1803140.735208-1.434959
C4.381113-1.2041270.695809
H4.503343-1.9731961.452581
C5.474989-0.6784070.051211
H6.471934-1.0290820.294644
C-1.1830152.048915-0.592265
O-2.4416472.277404-0.610315
O-0.2361772.859465-0.398321
K-2.1317961.9094881.967227

$2_A(S_0)$

Electronic Energy ωB97X-D/6-31G = -1856.65756934 Hartree

Electronic Energy ωB97X-D/6-31G(SMD=DMF) = -1856.69538839 Hartree

Zero-point Energy Correction = 0.233431 Hartree

Thermal Correction to Enthalpy = 0.251406 Hartree

Thermal Correction to Free Energy = 0.186970 Hartree

Chemical symbol X, Y, Z

C-2.367760-3.2467760.064887

C-1.594624-2.1138000.314764

C-1.777892-0.937199-0.417769

C-2.760115-0.911449-1.415234

C-3.553312-2.031011-1.648131

C-3.360553-3.202433-0.911313

H-2.189267-4.1524540.633444

H-2.9119990.002653-1.971277

H-4.319172-1.992024-2.414932

H-3.971357-4.077838-1.101766

N-0.9380380.187330-0.167985

S-0.329077-2.1365311.634718

C0.916427-1.1280640.754837

C0.462808-0.037898-0.038467

C1.3923600.754298-0.674556

H1.0541411.596051-1.263046

C2.253183-1.4046930.892145

H2.574661-2.2553051.484082

C2.7826440.517891-0.524926

C3.226381-0.5821530.266691

C3.7549591.341737-1.159127

H3.4141462.179472-1.759856

C5.0974641.085283-1.014979

H5.8293091.719817-1.503270

C4.622162-0.8234340.398238

H4.952670-1.6631171.002325

C5.536662-0.010049-0.227286

H6.599062-0.201549-0.121694

C-1.4916411.486641-0.020767

O-2.7665451.604008-0.102698

O-0.6905592.4631300.201763

K-2.6515154.0336330.705780

$TS_2(S_0)$

Imaginary Freq = -45.5696 (cm^{-1})

Electronic Energy ωB97X-D/6-31G = -1856.64562344 Hartree

Electronic Energy ωB97X-D/6-31G(SMD=DMF) = -1856.68450358 Hartree

Zero-point Energy Correction = 0.232558 Hartree

Thermal Correction to Enthalpy = 0.249972 Hartree

Thermal Correction to Free Energy = 0.187108 Hartree

Chemical symbol X, Y, Z

C3.080289-2.317479-0.295881

C2.099222-1.329552-0.326393

C1.984068-0.3759290.691003

C2.901851-0.4066481.747108

C3.906553-1.3701831.767197

C3.996680-2.3299680.754674

H3.125984-3.061269-1.083264

H2.8326000.3490272.516708

H4.619258-1.3782442.584048

H4.771914-3.0871110.785147

N0.9401460.5852400.622222

S0.921705-1.212335-1.734047

C-0.563165-0.782339-0.741029

C-0.3733970.1260030.337420

C-1.4723740.5398861.056860

H-1.3382471.2567861.856522

C-1.800789-1.280012-1.060782

H-1.914068-1.996180-1.868159

C-2.7734530.0804200.730196

C-2.945346-0.853623-0.335964

C-3.9215950.5140601.452356

H-3.7888991.2271962.259614

C-5.1717490.0411631.134724

H-6.0388110.3778541.692240

C-4.252392-1.326176-0.640960

H-4.375538-2.039293-1.450555

C-5.339662-0.8902060.077149

H-6.332455-1.256047-0.160693

C1.2313282.0179540.746352

O2.4365032.3201970.964066

O0.2554102.7841670.438323

K1.1609602.301517-1.975038

$2_C(S_0)$

Electronic Energy ωB97X-D/6-31G = -1856.64787452 Hartree

Electronic Energy ωB97X-D/6-31G(SMD=DMF) = -1856.68834871 Hartree

Zero-point Energy Correction = 0.232664 Hartree

Thermal Correction to Enthalpy = 0.250847 Hartree

Thermal Correction to Free Energy = 0.185529 Hartree

Chemical symbol X, Y, Z

C-3.377540-2.1176030.055442

C-2.321065-1.2280150.225927

C-2.198457-0.063724-0.545013

C-3.1893340.205171-1.500688

C-4.267804-0.664270-1.649474

C-4.367504-1.826508-0.881842

H-3.424365-3.0218970.651610

H-3.1112701.114547-2.077540

H-5.031789-0.434500-2.383774

H-5.202799-2.504539-1.014579

N-1.0729440.781956-0.341276

S-1.042072-1.5573801.503265

C0.397280-0.9832070.525470

C0.2084010.181595-0.275623

C1.3016090.706878-0.937277

H1.1800071.607113-1.526710

C1.618402-1.6041720.626706

H1.723441-2.5087841.217407

C2.5863300.119204-0.817738

C2.752800-1.063524-0.034727

C3.7240620.670720-1.474373

H3.5962861.569588-2.069002

C4.9570700.075650-1.361205

H5.8153230.502719-1.867986

C4.042212-1.6576680.062629

H4.159410-2.5608550.653994

C5.118899-1.102107-0.585347

H6.097874-1.562169-0.509556

C-1.1904372.276519-0.363327

O-2.1794512.765305-0.944092

O-0.2517532.8485400.293818

K0.1611431.6201462.423567

$TS_3(S_0)$

Imaginary Freq = -22.1478 (cm^{-1})

Electronic Energy ωB97X-D/6-31G = -1856.63343998 Hartree

Electronic Energy ωB97X-D/6-31G(SMD=DMF) = -1856.67616250 Hartree

Zero-point Energy Correction = 0.231600 Hartree

Thermal Correction to Enthalpy = 0.249443 Hartree

Thermal Correction to Free Energy = 0.183997 Hartree

Chemical symbol X, Y, Z

C-3.520757-1.8761570.034671

C-2.283610-1.305183-0.263680

C-2.1551200.077897-0.492106

C-3.3343480.853486-0.452041

C-4.5670580.275621-0.170846

C-4.670848-1.0921950.088179

H-3.579215-2.9457540.207736

H-3.2753921.910288-0.680477

H-5.4518930.901957-0.162666

H-5.629704-1.5455290.309319

N-0.9273640.721967-0.685869

S-0.871699-2.448359-0.475669

C0.514454-1.270004-0.317971

C0.3346040.128679-0.575379

C1.4711990.927105-0.609311

H1.3724381.977697-0.857381

C1.756323-1.789597-0.048027

H1.859554-2.8528590.147945

C2.7664890.407141-0.361192

C2.920222-0.975289-0.054181

C3.9252331.233218-0.392493

H3.8100042.284946-0.634575

C5.1677530.707296-0.126003

H6.0442051.345254-0.155997

C4.215248-1.4921670.217066

H4.322338-2.5487480.444219

C5.316652-0.6686720.183637

H6.303655-1.0682990.387970

C-0.9498212.269661-0.541355

O-1.0135292.923361-1.588257

O-0.8946062.6089900.685477

K-0.6058880.7335362.311269

$2_D(S_0)$

Electronic Energy ωB97X-D/6-31G = -1856.63503562 Hartree

Electronic Energy ωB97X-D/6-31G(SMD=DMF) = -1856.68061613 Hartree

Zero-point Energy Correction = 0.232115 Hartree

Thermal Correction to Enthalpy = 0.250595 Hartree

Thermal Correction to Free Energy = 0.184480 Hartree

Chemical symbol X, Y, Z

C3.3581492.0400530.448843

C2.2115661.463782-0.090894

C2.1282540.076916-0.311556

C3.257133-0.7086820.007497

C4.415036-0.1198480.513007

C4.4712571.2538460.748619

H3.3831813.1118830.612685

H3.228713-1.776899-0.176241

H5.277048-0.7445680.720146

H5.3692091.7116911.146185

N0.956160-0.536032-0.775976

S0.8451842.544615-0.635310

C-0.5309131.384264-0.337098

C-0.323906-0.016278-0.548010

C-1.426289-0.858694-0.458976

H-1.301824-1.922191-0.634906

C-1.7679351.881148-0.019374

H-1.8913962.9471460.144232

C-2.726678-0.359126-0.181543

C-2.9057061.0324250.064989

C-3.860542-1.216382-0.114591

H-3.728400-2.274616-0.318785

C-5.104541-0.7122460.187127

H-5.962606-1.3742550.228647

C-4.2013981.5258950.372719

H-4.3283852.5891250.553031

C-5.2787870.6724980.435441

H-6.2660841.0554830.668125

C1.061897-2.007032-1.235383

O1.252631-2.186921-2.440411

O0.937313-2.817236-0.251889

K0.622386-1.6971781.963535

b) Cartesian Coordinates of Intermediates and Transition States in S_1

2(S_1)

Electronic Energy (TD-HF/TD-DFT) ωB97X-D/6-31G = -1856.50243458 Hartree

Electronic Energy (TD-HF/TD-DFT) ωB97X-D/6-31G(SMD=DMF) = -1856.54096353 Hartree

Zero-point Energy Correction = 0.229018 Hartree

Thermal Correction to Enthalpy = 0.247741 Hartree

Thermal Correction to Free Energy = 0.181126 Hartree

Chemical symbol X, Y, Z

C2.593398-3.160416-0.079769

C1.691472-2.116230-0.294827

C1.829546-0.8993850.391119

C2.866286-0.7658341.329148

C3.759118-1.8089761.540298

C3.633741-3.0066500.829469

H2.460258-4.093107-0.615673

H2.9711800.1673671.859844

H4.552915-1.6894392.268814

H4.329933-3.8204630.997703

N0.9149420.1702010.153824

S0.338452-2.333699-1.482883

C-0.876876-1.297333-0.620628

C-0.452062-0.099182-0.003953

C-1.4154940.7914470.546015

H-1.0726821.6771391.061005

C-2.243902-1.592342-0.729839

H-2.559411-2.512257-1.209159

C-2.7946010.5131240.468933

C-3.224361-0.712446-0.205581

C-3.7692171.3663091.036717

H-3.4365242.2663141.545590

C-5.1361391.0756830.940111

H-5.8704001.7466891.370623

C-4.611681-0.977804-0.275890

H-4.948815-1.883328-0.770442

C-5.545527-0.0967620.283671

H-6.603255-0.3301450.209406

C1.4077201.525016-0.010981

O2.6207501.7687250.304425

O0.5947142.377795-0.497536

K2.4402494.158818-0.704609

MECP

Electronic Energy ωB97X-D/6-31G = -1856.52532143 Hartree

Chemical symbol X, Y, Z

C 2.571272-3.052568-0.100954

C 1.727199-1.965744-0.319647

C 1.851409-0.787090 0.429283

C 2.843137-0.719841 1.419065

C 3.709661-1.793645 1.615121

C 3.580239-2.961768 0.859783

H 2.447442-3.959523-0.682928

H 2.942220 0.178725 2.012899

H 4.481624-1.718771 2.372998

H 4.250314-3.798572 1.021754

N 0.956013 0.294552 0.192181

S 0.451127-2.028943-1.631883

C-0.836877-1.092510-0.726066

C-0.426322 0.012048 0.054660

C-1.396327 0.785639 0.698098

H-1.095994 1.628758 1.305906

C-2.169500-1.426232-0.857695

H-2.465427-2.289435-1.445562

C-2.775664 0.499166 0.543304

C-3.178009-0.630553-0.238487

C-3.776615 1.294918 1.163125

H-3.470742 2.152375 1.754912

C-5.118190 0.983766 1.017156

H-5.871528 1.599330 1.497411

C-4.556525-0.924701-0.369629

H-4.854538-1.785066-0.960962

C-5.511494-0.130576 0.248604

H-6.564895-0.366659 0.141923

C 1.358200 1.638578 0.515439

O 2.654492 1.940463 0.387804

O 0.611750 2.627191-0.006256

K 2.076457 2.205556-2.276485

c) Cartesian Coordinates of Intermediates and Transition States in T_1

2(T_1)

Electronic Energy ωB97X-D/6-31G = -1856.51412753 Hartree

Electronic Energy ωB97X-D/6-31G(SMD=DMF) = -1856.54741846 Hartree

Zero-point Energy Correction = 0.230434 Hartree

Thermal Correction to Enthalpy = 0.248686 Hartree

Thermal Correction to Free Energy = 0.183264 Hartree

Chemical symbol X, Y, Z

C-2.977514-2.425485-0.086237

C-2.011809-1.4499570.143372

C-1.924953-0.302888-0.656991

C-2.854972-0.131977-1.692382

C-3.844746-1.089136-1.900225

C-3.908812-2.238462-1.107586

H-3.002515-3.3155900.532212

H-2.8015820.754201-2.310998

H-4.563803-0.942222-2.698258

H-4.672708-2.986260-1.286348

N-0.9057340.644862-0.388625

S-0.835029-1.5863631.545702

C0.632791-0.9346260.656466

C0.4195260.172300-0.213206

C1.5059770.753521-0.833056

H1.3580371.601373-1.490578

C1.882732-1.4531530.869008

H2.016399-2.3127421.517581

C2.8193110.268747-0.600922

C3.016739-0.8580800.252346

C3.9551840.867707-1.215335

H3.8045371.725800-1.862541

C5.2175830.371234-0.994386

H6.0749530.836057-1.468642

C4.334512-1.3493280.462348

H4.475708-2.2082741.111528

C5.410852-0.749806-0.147025

H6.412730-1.1310530.016629

C-1.1135912.054002-0.801073

O-0.3227412.924936-0.112797

O-2.3683402.502325-0.520053

K-1.6434411.9158272.009508

$TS_1(T_1)$

Imaginary Freq = -408.7260 (cm^{-1})

Electronic Energy ωB97X-D/6-31G = -1856.50332248 Hartree

Electronic Energy ωB97X-D/6-31G(SMD=DMF) = -1856.54265655 Hartree

Zero-point Energy Correction = 0.228298 Hartree

Thermal Correction to Enthalpy = 0.246521 Hartree

Thermal Correction to Free Energy = 0.180400 Hartree

Chemical symbol X, Y, Z

C-2.7369802.4901280.250556

C-1.6486191.6392850.058507

C-1.6202310.3668100.656500

C-2.695803-0.0206471.482816

C-3.7928080.8231681.649445

C-3.8189822.0772851.029361

H-2.7326803.475233-0.202989

H-2.633944-0.9893871.961606

H-4.6098100.5181152.294825

H-4.6605432.7449461.177028

N-0.589643-0.5555040.434431

S-0.2890392.168627-1.024950

C1.0472021.106935-0.376864

C0.729715-0.1519560.209087

C1.754501-1.0097780.560473

H1.494679-1.9745420.980570

C2.3503651.494123-0.552320

H2.5798542.460991-0.988465

C3.109720-0.6487820.368929

C3.4179030.629606-0.186249

C4.177537-1.5193500.725922

H3.938497-2.4915451.144982

C5.485081-1.1376970.542524

H6.291892-1.8082730.816986

C4.7795170.996599-0.363170

H5.0091501.969748-0.786285

C5.7895370.133885-0.007148

H6.8257590.421914-0.146484

C-0.995766-2.082339-0.071510

O-1.991892-2.6279960.546481

O-1.090039-1.678783-1.382420

K-3.688770-1.674006-1.161434

($4{\cdot}CO_2^{\bullet-}K^+$)($T_1$)

Electronic Energy ωB97X-D/6-31G = -1856.55407155 Hartree

Electronic Energy ωB97X-D/6-31G(SMD=DMF) = -1856.59359841 Hartree

Zero-point Energy Correction = 0.227374 Hartree

Thermal Correction to Enthalpy = 0.247303 Hartree

Thermal Correction to Free Energy = 0.175494 Hartree

Chemical symbol X, Y, Z

C-3.2238441.745281-0.478862

C-1.9238751.402387-0.091009

C-1.7006100.6366291.082555

C-2.8337470.2703051.857079

C-4.1206430.6188201.473256

C-4.3206311.3571270.292009

H-3.3740392.328802-1.381173

H-2.638205-0.2958882.760261

H-4.9670060.3406362.091570

H-5.3206071.648089-0.010087

N-0.4908200.1874441.542996

S-0.5593141.993089-1.103871

C0.8430491.124622-0.350691

C0.6755690.3698110.861933

C1.807625-0.2511891.405748

H1.664759-0.8267382.312977

C2.0636781.228498-0.956776

H2.1712161.788282-1.880223

C3.074808-0.1618860.800253

C3.2120130.593018-0.406196

C4.222657-0.8026371.349583

H4.110423-1.3796382.261807

C5.445436-0.6973370.731839

H6.314174-1.1902711.153315

C4.4874850.682878-1.020117

H4.5894911.252703-1.938181

C5.5786850.052900-0.464412

H6.5488850.125428-0.943396

C-0.382410-2.535398-0.934406

O-1.157956-3.410793-0.451465

O-0.636765-1.386157-1.409814

K-3.146738-1.706332-0.715074

TS_2(T_1)

Imaginary Freq = -24.6269 (cm^{-1})

Electronic Energy ωB97X-D/6-31G = -1856.50723094 Hartree

Electronic Energy ωB97X-D/6-31G(SMD=DMF) = -1856.54299440 Hartree

Zero-point Energy Correction = 0.229798 Hartree

Thermal Correction to Enthalpy = 0.247382 Hartree

Thermal Correction to Free Energy = 0.183486 Hartree

Chemical symbol X, Y, Z

C-3.699255-1.9320540.188251

C-2.524392-1.1878400.263982

C-2.339510-0.043806-0.518866

C-3.3811950.373530-1.357474

C-4.571748-0.345502-1.407580

C-4.732535-1.505477-0.645082

H-3.808399-2.8275430.789825

H-3.2375721.268835-1.949992

H-5.372723-0.006409-2.054885

H-5.654035-2.073759-0.696907

N-1.1268230.680796-0.473736

S-1.234942-1.6433831.483662

C0.230536-1.1156240.531949

C0.1062990.002513-0.351421

C1.2332030.427357-1.036914

H1.1395441.244824-1.739316

C1.435476-1.7451770.724009

H1.497664-2.6069031.381231

C2.504123-0.169222-0.813393

C2.613595-1.2749760.081498

C3.6772690.301126-1.469389

H3.5891851.135460-2.158601

C4.895588-0.295192-1.242728

H5.7818570.067846-1.751445

C3.886279-1.8725070.294433

H3.961127-2.7180720.971582

C5.002185-1.393138-0.350892

H5.968452-1.856770-0.185995

C-1.2304572.144118-0.402222

O-0.1375672.855703-0.727398

O-1.5246562.6211750.866759

K0.9708822.3093291.627801

2'(T_1)

Electronic Energy ωB97X-D/6-31G = -1856.50794789 Hartree

Electronic Energy ωB97X-D/6-31G(SMD=DMF) = -1856.54512227 Hartree

Zero-point Energy Correction = 0.229977 Hartree

Thermal Correction to Enthalpy = 0.248352 Hartree

Thermal Correction to Free Energy = 0.182070 Hartree

Chemical symbol X, Y, Z

C-3.957353-1.7387990.067979

C-2.714572-1.1275570.219807
C-2.4430070.111135-0.370989
C-3.4613790.752443-1.088318
C-4.7159100.162029-1.209286
C-4.966506-1.089916-0.642151
H-4.136456-2.7083050.519512
H-3.2475531.715099-1.536744
H-5.4961030.674842-1.760619
H-5.938054-1.558210-0.750109
N-1.1697170.712619-0.261929
S-1.463611-1.9022931.309997
C0.043978-1.3121600.473943
C0.002496-0.071710-0.234564
C1.1567430.363857-0.864653
H1.1005131.253670-1.481048
C1.209629-2.0268390.585512
H1.215332-2.9712991.120470
C2.390306-0.327127-0.719556
C2.425202-1.5402460.029354
C3.5979170.156603-1.299741
H3.5629061.061544-1.901525
C4.782089-0.524955-1.130936
H5.694354-0.152292-1.584291
C3.661374-2.2240250.182247
H3.680441-3.1494670.749794
C4.815216-1.726526-0.379538
H5.753231-2.256644-0.258290
C-1.1109932.1417730.018561
O-0.2359712.907272-0.673110
O-0.8047092.4575001.322276
K1.7605922.6712301.091424

$TS_3(T_1)$

Imaginary Freq = -399.9685 (cm^{-1})

Electronic Energy ωB97X-D/6-31G = -1856.50500863 Hartree

Electronic Energy ωB97X-D/6-31G(SMD=DMF) = -1856.54303746 Hartree

Zero-point Energy Correction = 0.228124 Hartree

Thermal Correction to Enthalpy = 0.246379 Hartree

Thermal Correction to Free Energy = 0.180340 Hartree

Chemical symbol X, Y, Z

C-3.923604-1.7226230.142073
C-2.655880-1.1451840.177392
C-2.4026990.064049-0.484446
C-3.4554500.710147-1.150818
C-4.7245290.145118-1.169148
C-4.960042-1.076399-0.529891

H-4.099820-2.6661610.646445

H-3.2438151.655665-1.634365

H-5.5309530.652386-1.685974

H-5.946954-1.524261-0.551853

N-1.1372400.659901-0.507332

S-1.367514-1.9252851.206373

C0.122479-1.2780480.390993

C0.039859-0.087344-0.394806

C1.1870120.394886-1.010919

H1.0961611.275945-1.634324

C1.324913-1.9124020.580933

H1.372211-2.8244891.167611

C2.447705-0.221627-0.797383

C2.523892-1.3902180.019909

C3.6435430.297385-1.371586

H3.5763541.175779-2.006802

C4.855530-0.310331-1.138396

H5.7600100.088795-1.584085

C3.789978-1.9994760.236227

H3.843110-2.8924130.851586

C4.929148-1.470637-0.326042

H5.889745-1.944231-0.156181

C-1.0650242.270477-0.124212

O-0.0596652.916423-0.618123

O-1.1678232.0161181.222214

K1.4149352.4274351.458173

(4·$CO_2^{\bullet-}K^+$)'(T1)

Electronic Energy ωB97X-D/6-31G = -1856.55559333 Hartree

Electronic Energy ωB97X-D/6-31G(SMD=DMF) = -1856.59366721 Hartree

Zero-point Energy Correction = 0.227332 Hartree

Thermal Correction to Enthalpy = 0.247318 Hartree

Thermal Correction to Free Energy = 0.175475 Hartree

Chemical symbol X, Y, Z

C-3.962688-1.0893500.759595

C-2.661878-0.9989840.271325

C-2.389265-0.341869-0.954211

C-3.4851590.210168-1.666248

C-4.7745780.120498-1.179719

C-5.016707-0.5318810.040250

H-4.150501-1.5832251.706380

H-3.2548780.716942-2.595054

H-5.5966590.558941-1.732853

H-6.025315-0.5984380.431700

N-1.160494-0.178583-1.527843

S-1.349724-1.7831831.233188

C0.118194-1.2179900.354737

C0.003539-0.537511-0.909405

C1.187372-0.177184-1.565903

H1.0839090.330108-2.518703

C1.349248-1.4733660.910170

H1.417205-1.9800181.868251

C2.464042-0.423110-1.013361

C2.552421-1.0816620.256661

C3.666533-0.002201-1.655787

H3.5954490.487828-2.621838

C4.890327-0.217078-1.066394

H5.7982770.103849-1.564287

C3.833939-1.2925110.835971

H3.898605-1.8013011.793033

C4.975236-0.8686420.191917

H5.947314-1.0386610.641011

C-1.2345492.6888140.645935

O-0.4012083.5313210.202163

O-1.0473211.6012101.273209

K1.5525111.9435730.958952

d) Cartesian Coordinates of 4(S_0) and·$CO_2^{\bullet-}K^+$

4(S_0)

Electronic Energy ωB97X-D/6-31G = -1068.21336406 Hartree

Electronic Energy ωB97X-D/6-31G(SMD=DMF) = -1068.23313206 Hartree

Zero-point Energy Correction = 0.216730 Hartree

Thermal Correction to Enthalpy = 0.230103 Hartree

Thermal Correction to Free Energy = 0.176524 Hartree

Chemical symbol X, Y, Z

C3.9052011.054092-0.000630

C2.5897070.589968-0.000088

C2.309275-0.7986160.000337

C3.411659-1.6916880.000268

C4.714670-1.229695-0.000204

C4.9656440.151734-0.000620

H4.0985842.121386-0.001132

H3.177402-2.7492870.000463

H5.540564-1.931449-0.000251

H5.9844190.521956-0.001151

N1.066900-1.3796580.000577

S1.2678581.8278920.000408

C-0.2106470.7740700.000101

C-0.096653-0.6600680.000204

C-1.282506-1.4052510.000120

H-1.182802-2.4848600.000288

C-1.4362491.3875020.000133

H-1.5042642.4710950.000260

C-2.554289-0.799105-0.000047

C-2.6388570.6282020.000055

C-3.758179-1.561589-0.000259

H-3.688966-2.644734-0.000344

C-4.982313-0.937437-0.000376

H-5.893854-1.524444-0.000558

C-3.9182621.243290-0.000049

H-3.9797952.3270100.000041

C-5.0631100.479010-0.000259

H-6.0358620.958152-0.000293

$CO_2^{\bullet-}K^+$

Electronic Energy ωB97X-D/6-31G = -788.322158931 Hartree

Electronic Energy ωB97X-D/6-31G(SMD=DMF) = -788.347396768 Hartree

Zero-point Energy Correction = 0.009730 Hartree

Thermal Correction to Enthalpy = 0.015252 Hartree

Thermal Correction to Free Energy = -0.019077 Hartree

Chemical symbol X, Y, Z

C1.5382160.000963-0.000636

O1.009557-1.1499880.000292

O1.0066811.1505150.000293

K-1.334695-0.000526-0.000045

7. NMR Traces

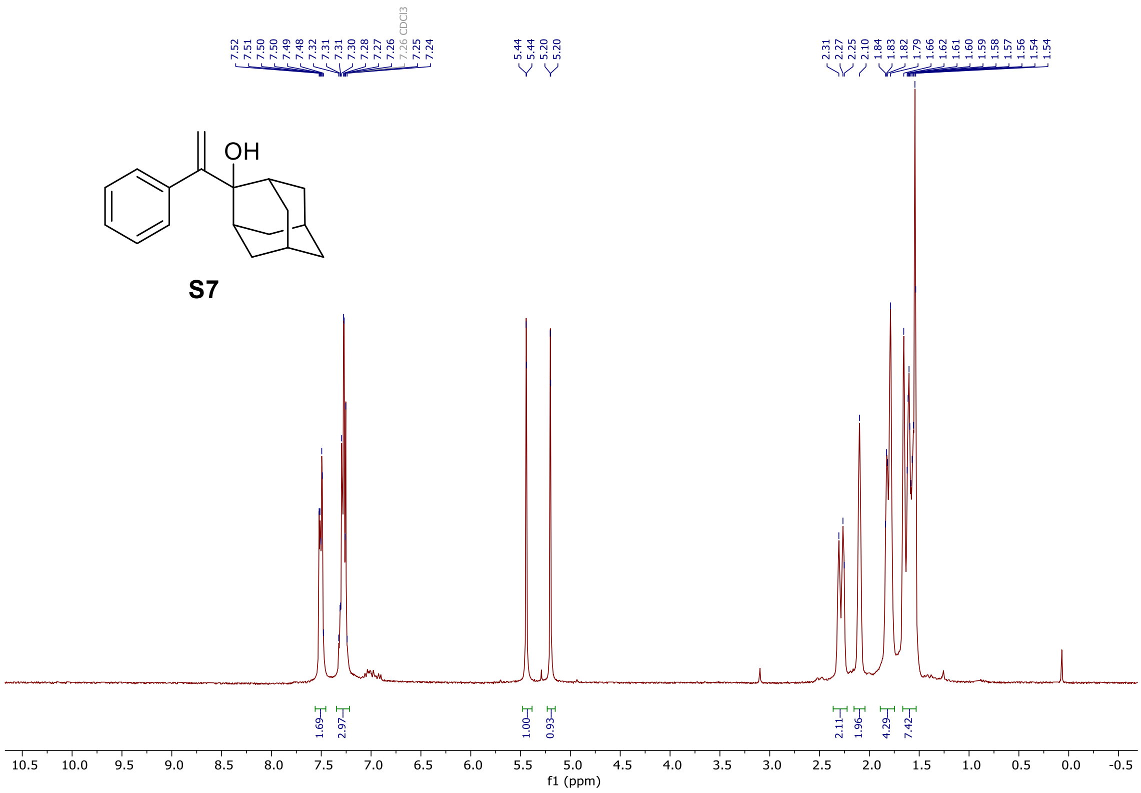

7.52 7.51 7.50 7.50 7.49 7.48 7.32 7.31 7.31 7.30 7.28 7.27 7.26 7.26 CDCl3 7.25 7.24
5.44 5.44 5.20 5.20
2.31 2.27 2.25 2.10 1.84 1.83 1.82 1.79 1.66 1.62 1.61 1.60 1.59 1.58 1.57 1.56 1.54 1.54
OH
S7
1.69 2.97 1.00 0.93 2.11 1.96 4.29 7.42
f1 (ppm)

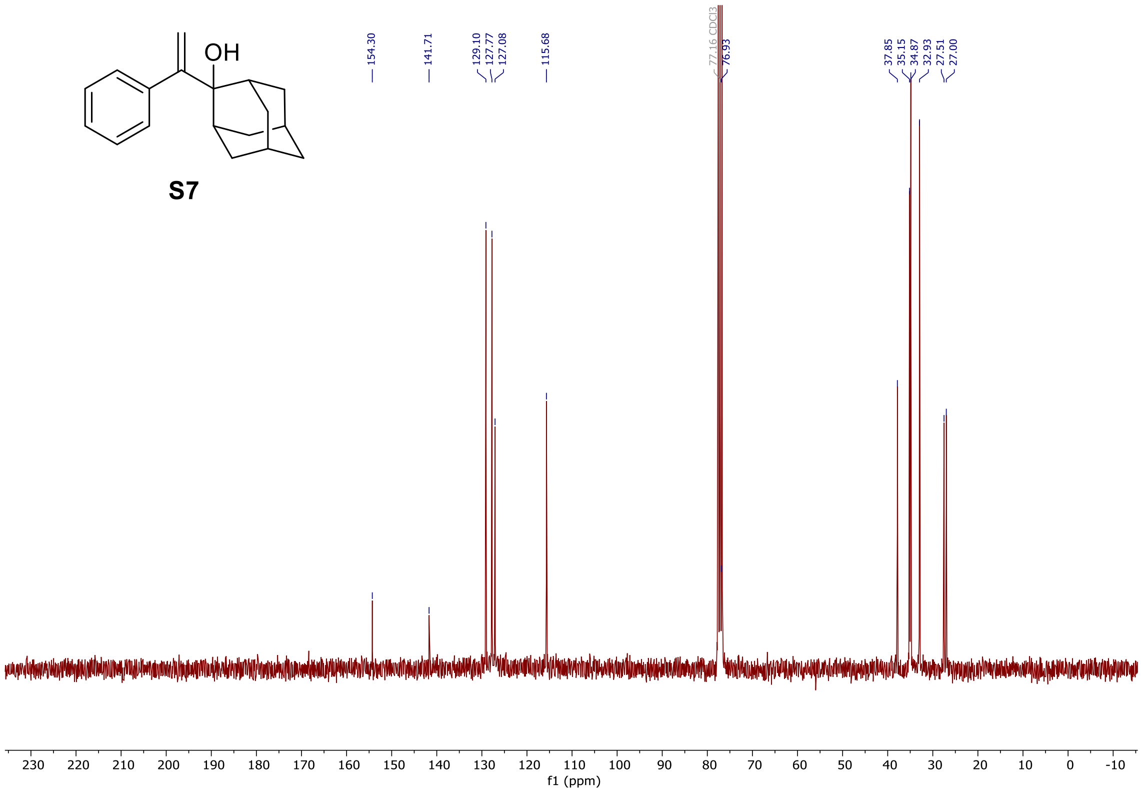

154.30
141.71
129.10 127.77 127.08
115.68
77.16 CDCl3 76.93
37.85 35.15 34.87 32.93 27.51 27.00
OH
S7
f1 (ppm)

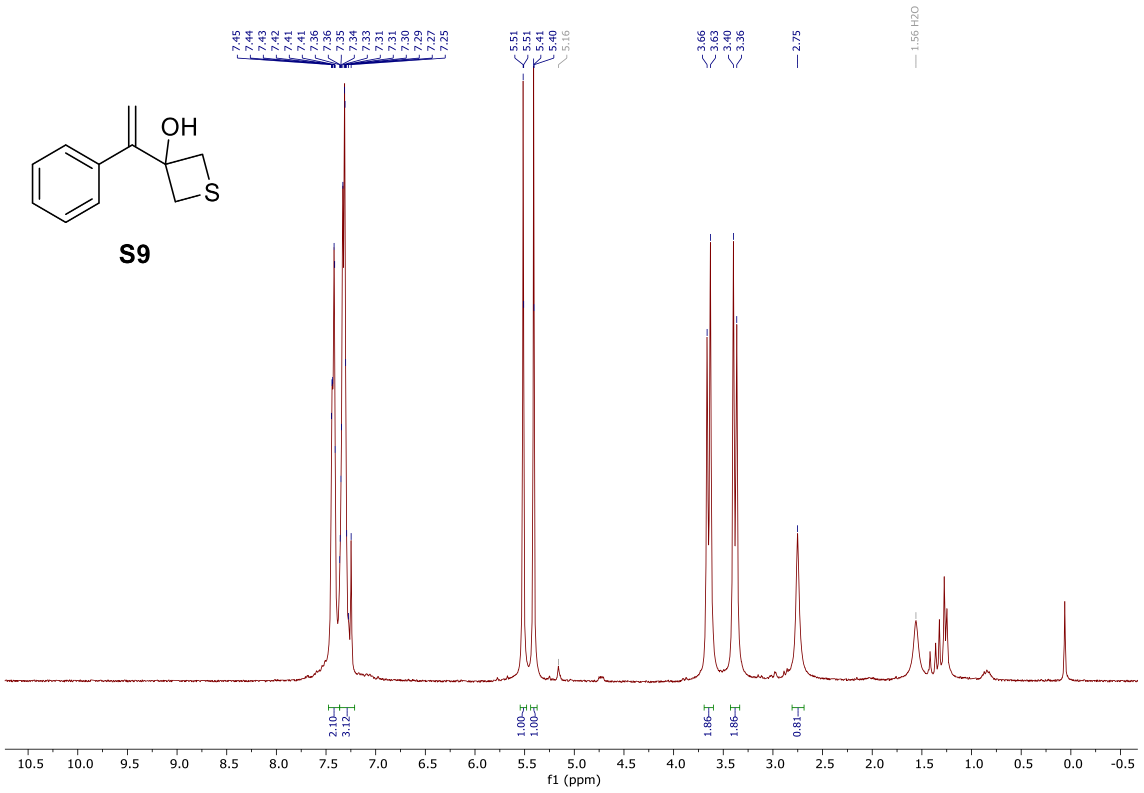

S9

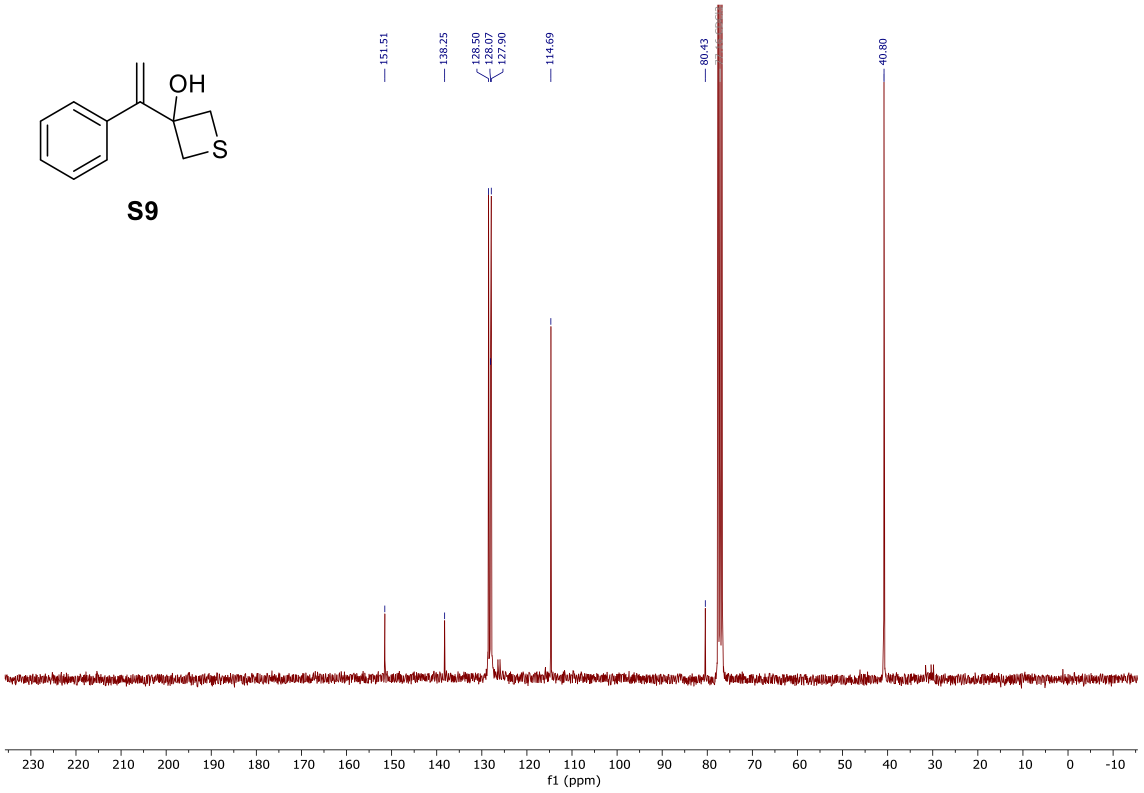

S9

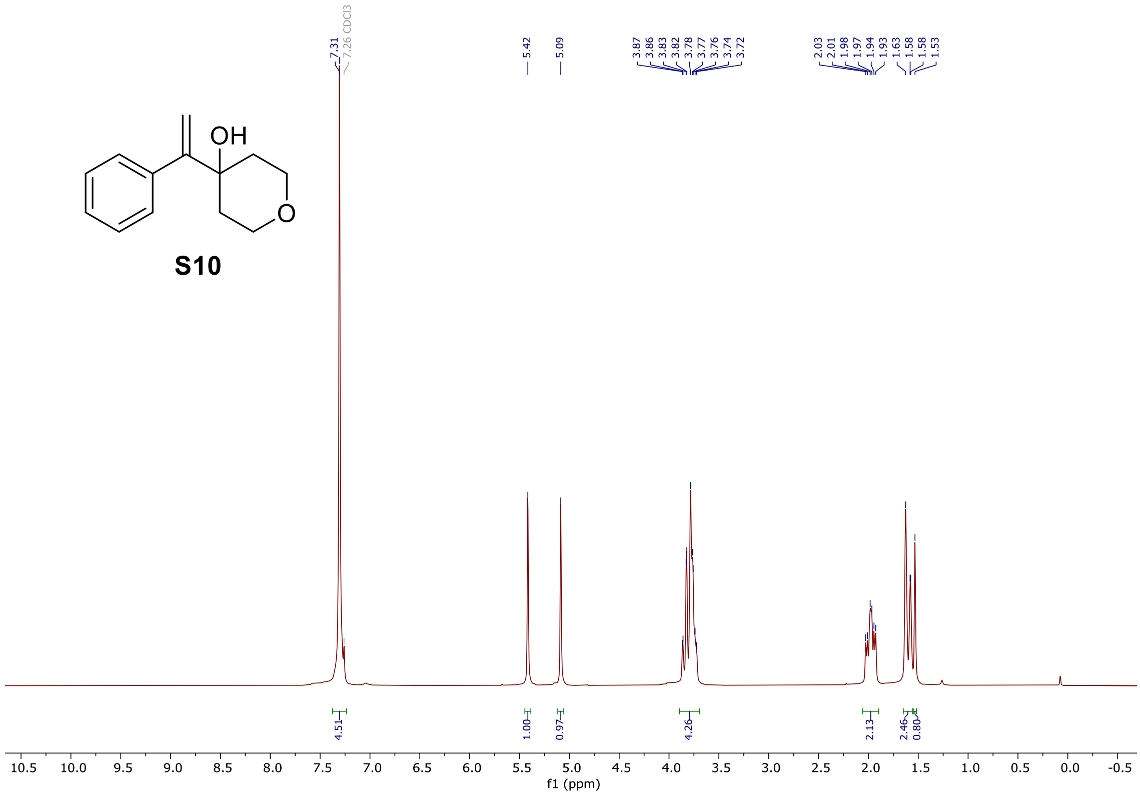
S10
OH
O
7.31
7.26 CDCl3
5.42
5.09
3.87
3.86
3.83
3.82
3.78
3.77
3.76
3.74
3.72
2.03
2.01
1.98
1.97
1.94
1.93
1.63
1.58
1.58
1.53
4.51
1.00
0.97
4.26
2.13
2.46
0.80
f1 (ppm)

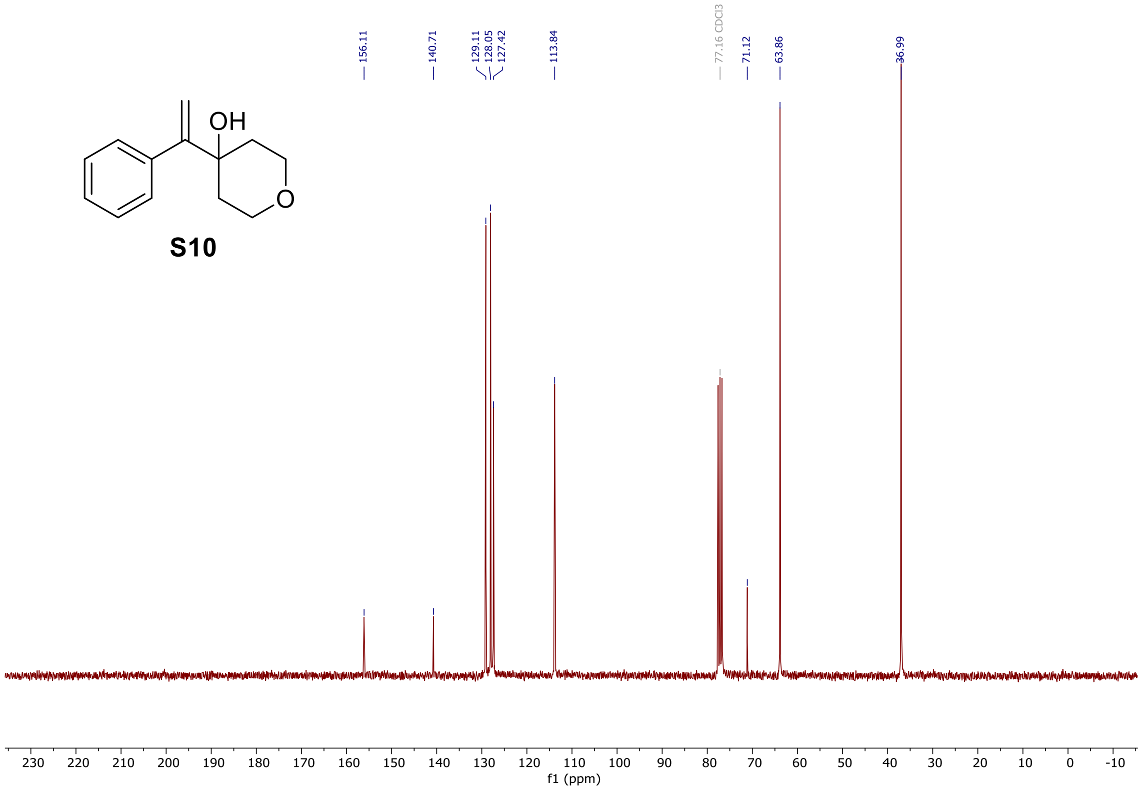
S10
OH
O
156.11
140.71
129.11
128.05
127.42
113.84
77.16 CDCl3
71.12
63.86
36.99
f1 (ppm)

S11

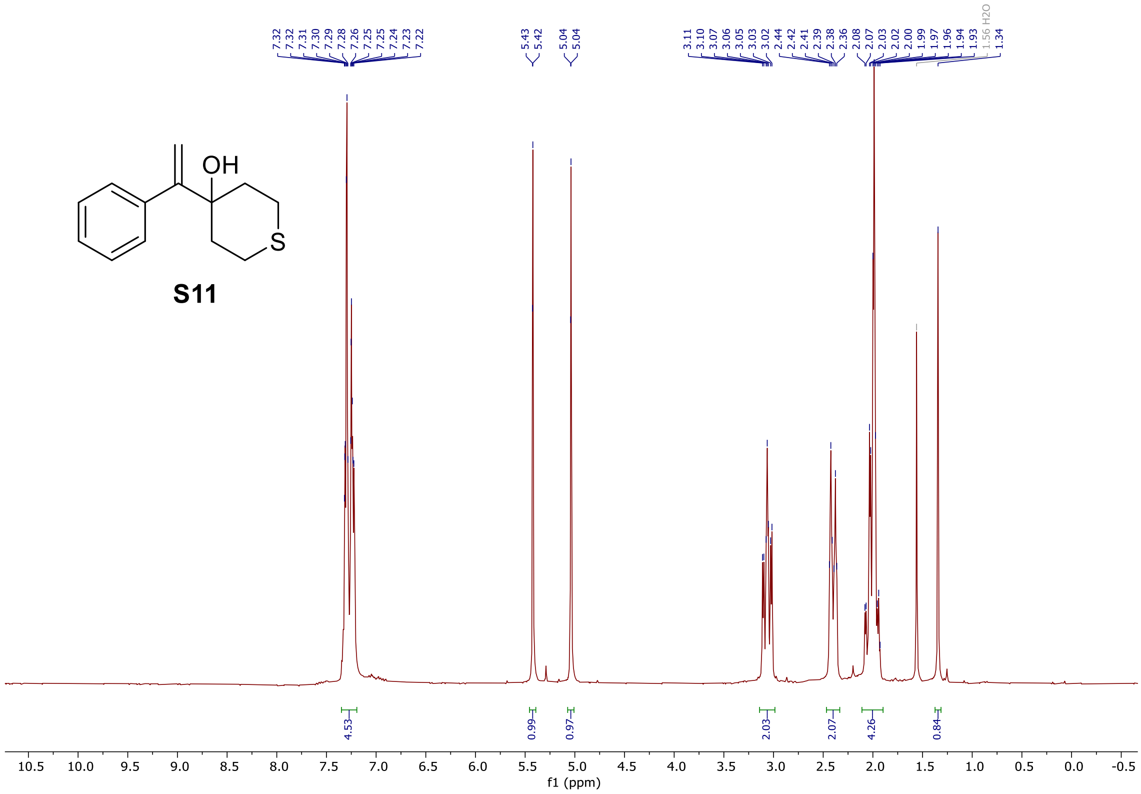
7.32
7.32
7.31
7.30
7.29
7.28
7.26
7.25
7.25
7.24
7.23
7.22
5.43
5.42
5.04
5.04
3.11
3.10
3.07
3.06
3.05
3.03
3.02
2.44
2.42
2.41
2.39
2.38
2.36
2.08
2.07
2.03
2.02
2.00
1.99
1.97
1.96
1.94
1.93
1.56 H2O
1.34
4.53
0.99
0.97
2.03
2.07
4.26
0.84
f1 (ppm)

S11

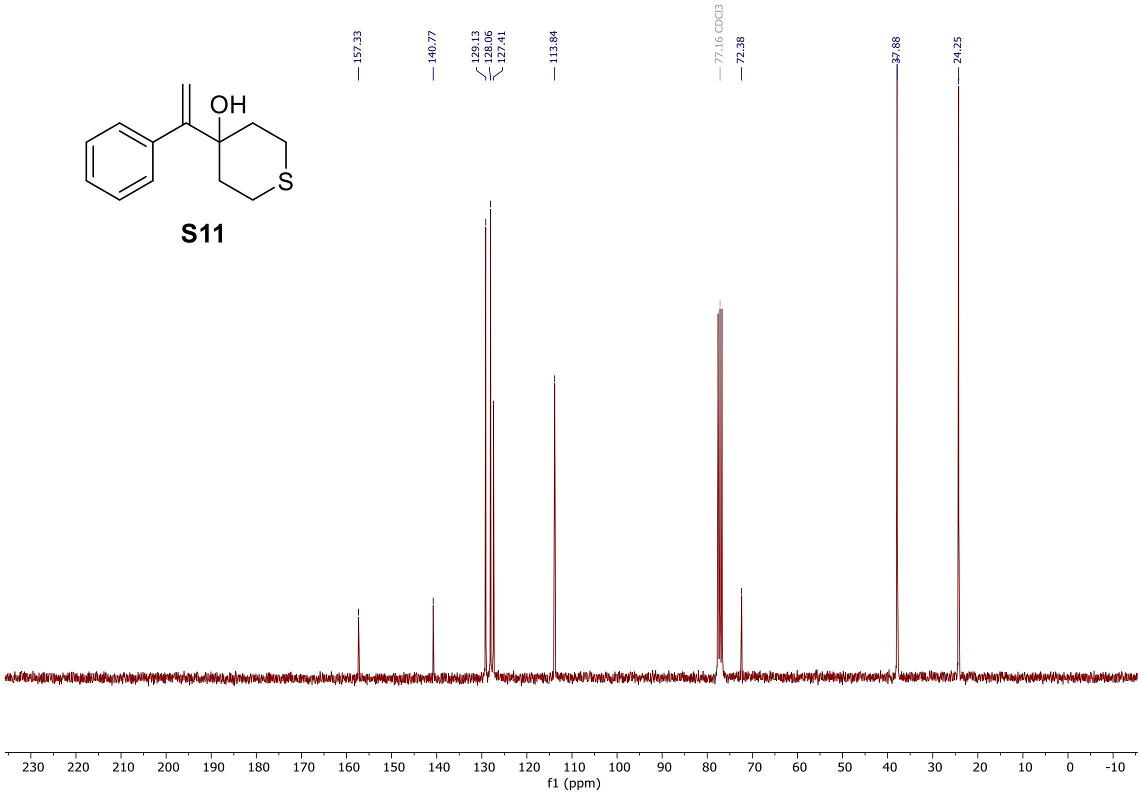
157.33
140.77
129.13
128.06
127.41
113.84
77.16 CDCl3
72.38
37.88
24.25
f1 (ppm)

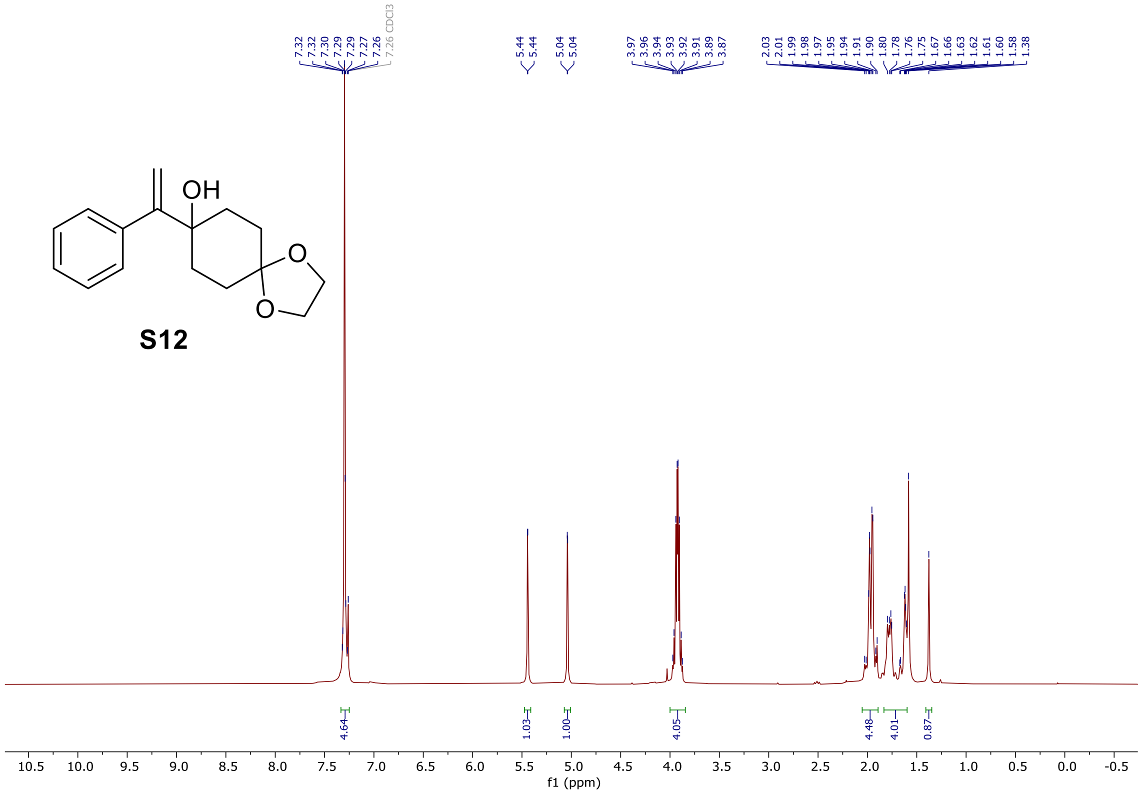
7.32 7.32 7.30 7.29 7.29 7.27 7.26 7.26 CDCl3
5.44 5.44
5.04 5.04
3.97 3.96 3.94 3.93 3.92 3.91 3.89 3.87
2.03 2.01 1.99 1.98 1.97 1.95 1.94 1.91 1.90 1.80 1.78 1.76 1.75 1.67 1.66 1.63 1.62 1.61 1.60 1.58 1.38
OH
O
O
S12
4.64
1.03
1.00
4.05
4.48
4.01
0.87
10.5 10.0 9.5 9.0 8.5 8.0 7.5 7.0 6.5 6.0 5.5 5.0 4.5 4.0 3.5 3.0 2.5 2.0 1.5 1.0 0.5 0.0 -0.5
f1 (ppm)

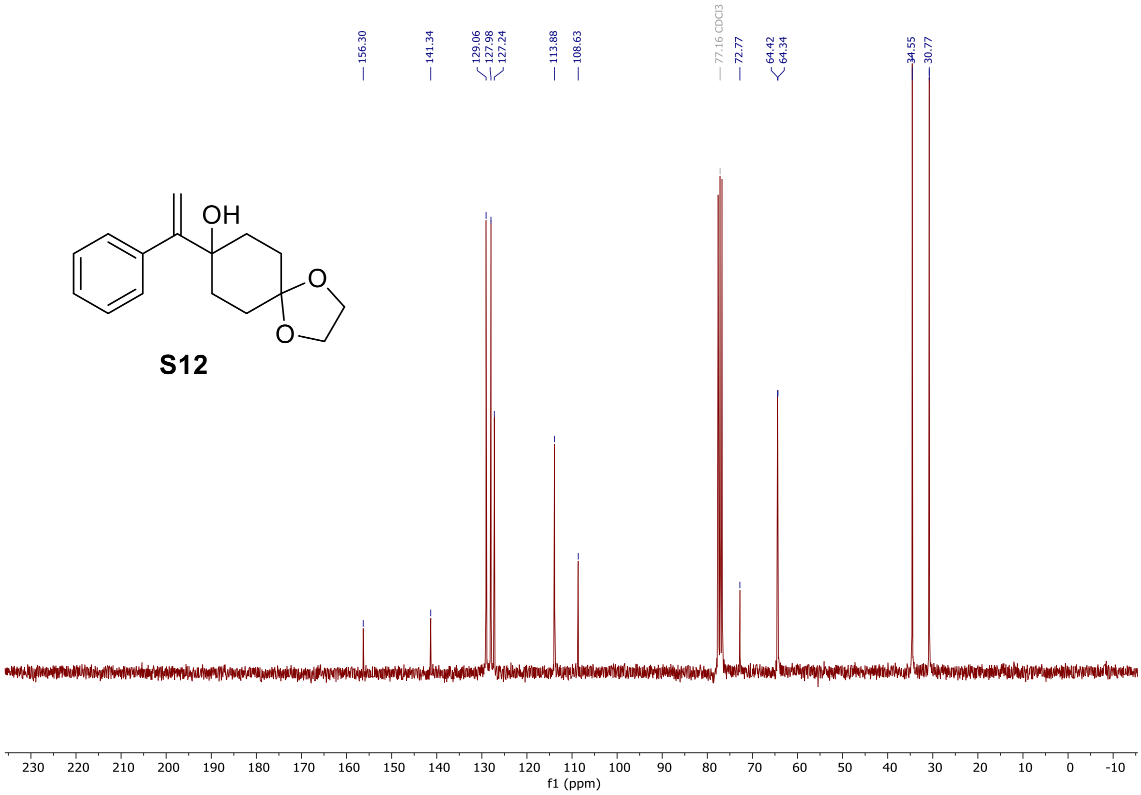
156.30
141.34
129.06 127.98 127.24
113.88
108.63
77.16 CDCl3
72.77
64.42 64.34
34.55
30.77
OH
O
O
S12
230 220 210 200 190 180 170 160 150 140 130 120 110 100 90 80 70 60 50 40 30 20 10 0 -10
f1 (ppm)

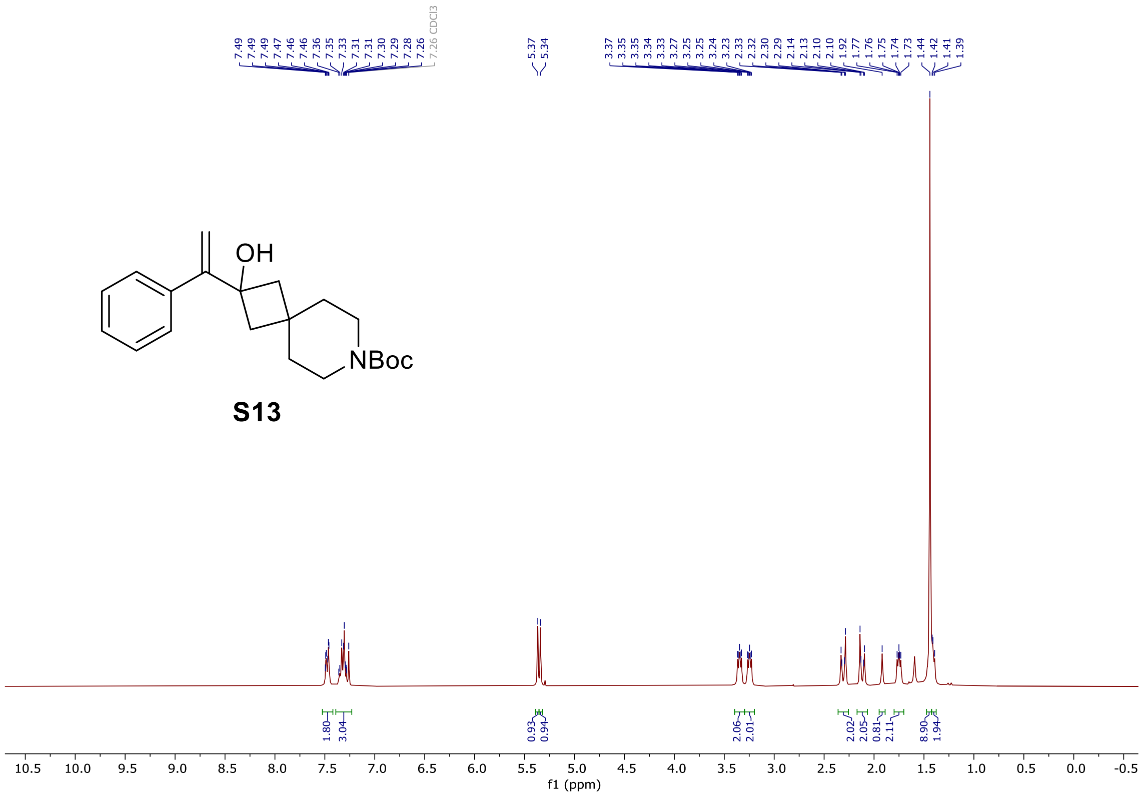
7.49 7.49 7.49 7.47 7.46 7.46 7.36 7.35 7.33 7.31 7.31 7.30 7.29 7.28 7.26 7.26 CDCl3
5.37 5.34
3.37 3.35 3.35 3.34 3.33 3.27 3.25 3.25 3.24 3.23 2.33 2.32 2.30 2.29 2.14 2.13 2.10 2.10 1.92 1.77 1.76 1.75 1.74 1.73 1.44 1.42 1.41 1.39
OH
NBoc
S13
1.80 3.04 0.93 0.94 2.06 2.01 2.02 2.05 0.81 2.11 8.90 1.94
10.5 10.0 9.5 9.0 8.5 8.0 7.5 7.0 6.5 6.0 5.5 5.0 4.5 4.0 3.5 3.0 2.5 2.0 1.5 1.0 0.5 0.0 -0.5
f1 (ppm)

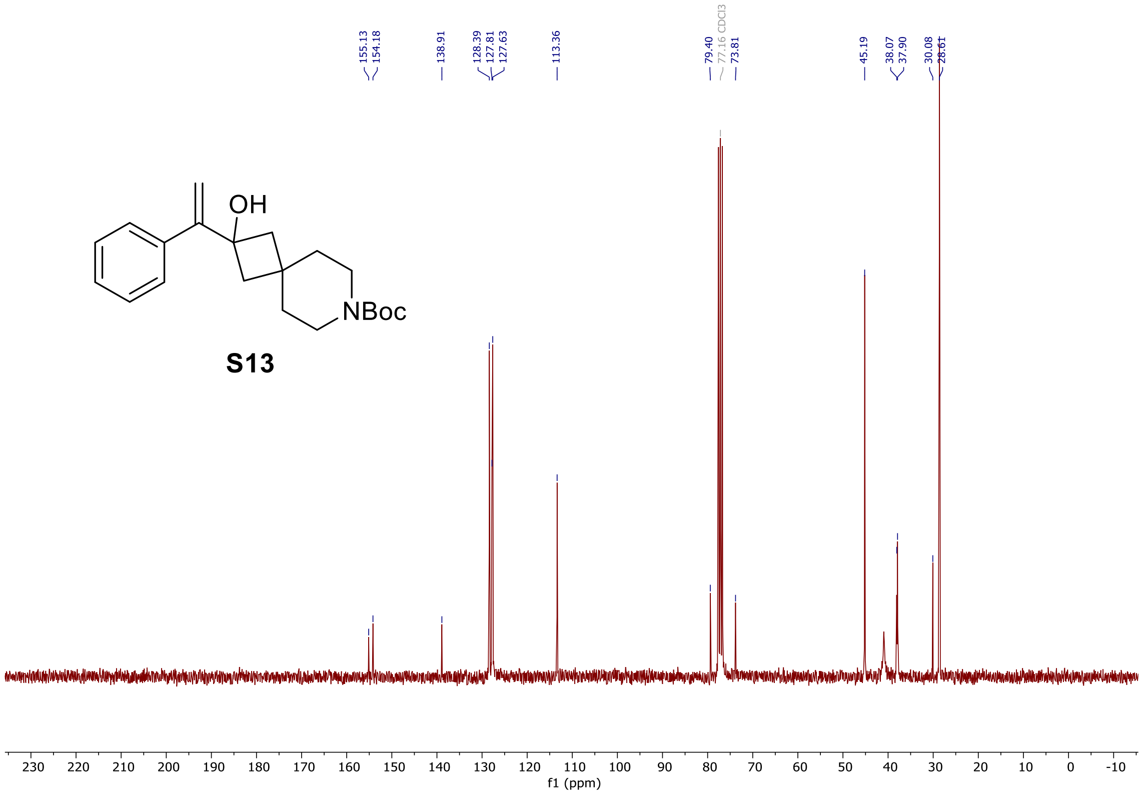
155.13 154.18 138.91 128.39 127.81 127.63 113.36 79.40 77.16 CDCl3 73.81 45.19 38.07 37.90 30.08 28.61
OH
NBoc
S13
230 220 210 200 190 180 170 160 150 140 130 120 110 100 90 80 70 60 50 40 30 20 10 0 -10
f1 (ppm)

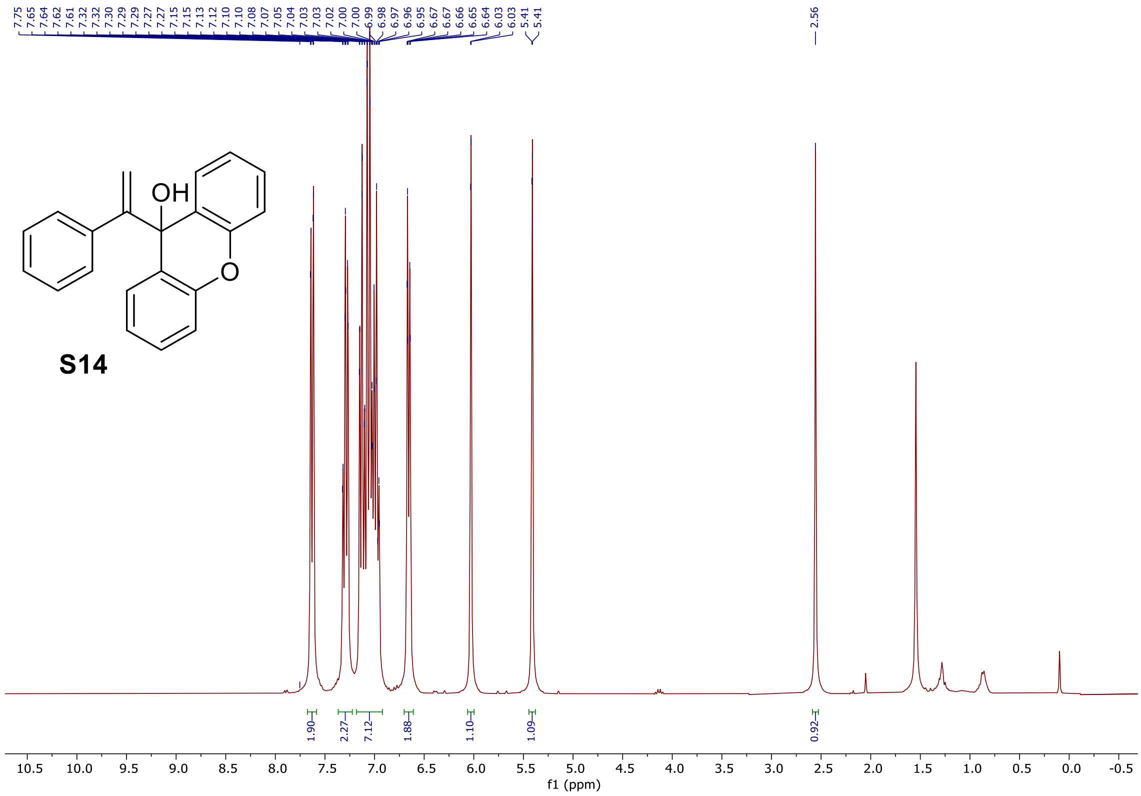
S14
f1 (ppm)

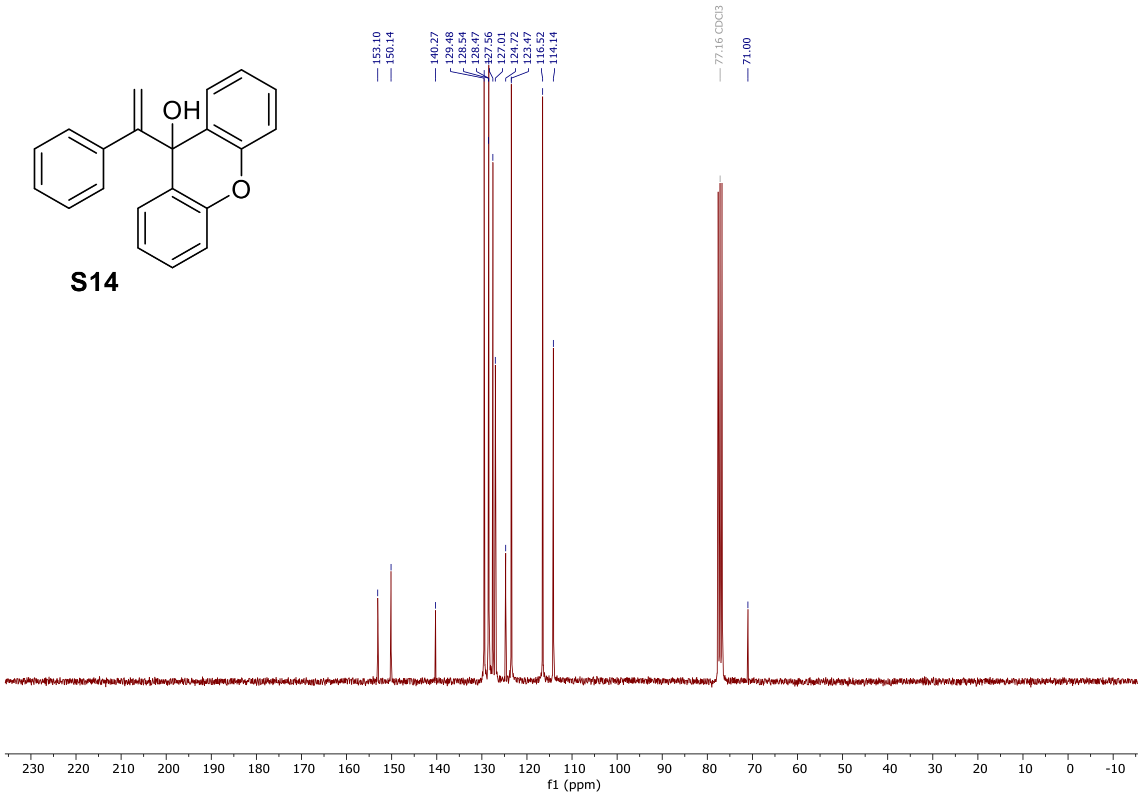
S14
77.16 CDCl3
f1 (ppm)

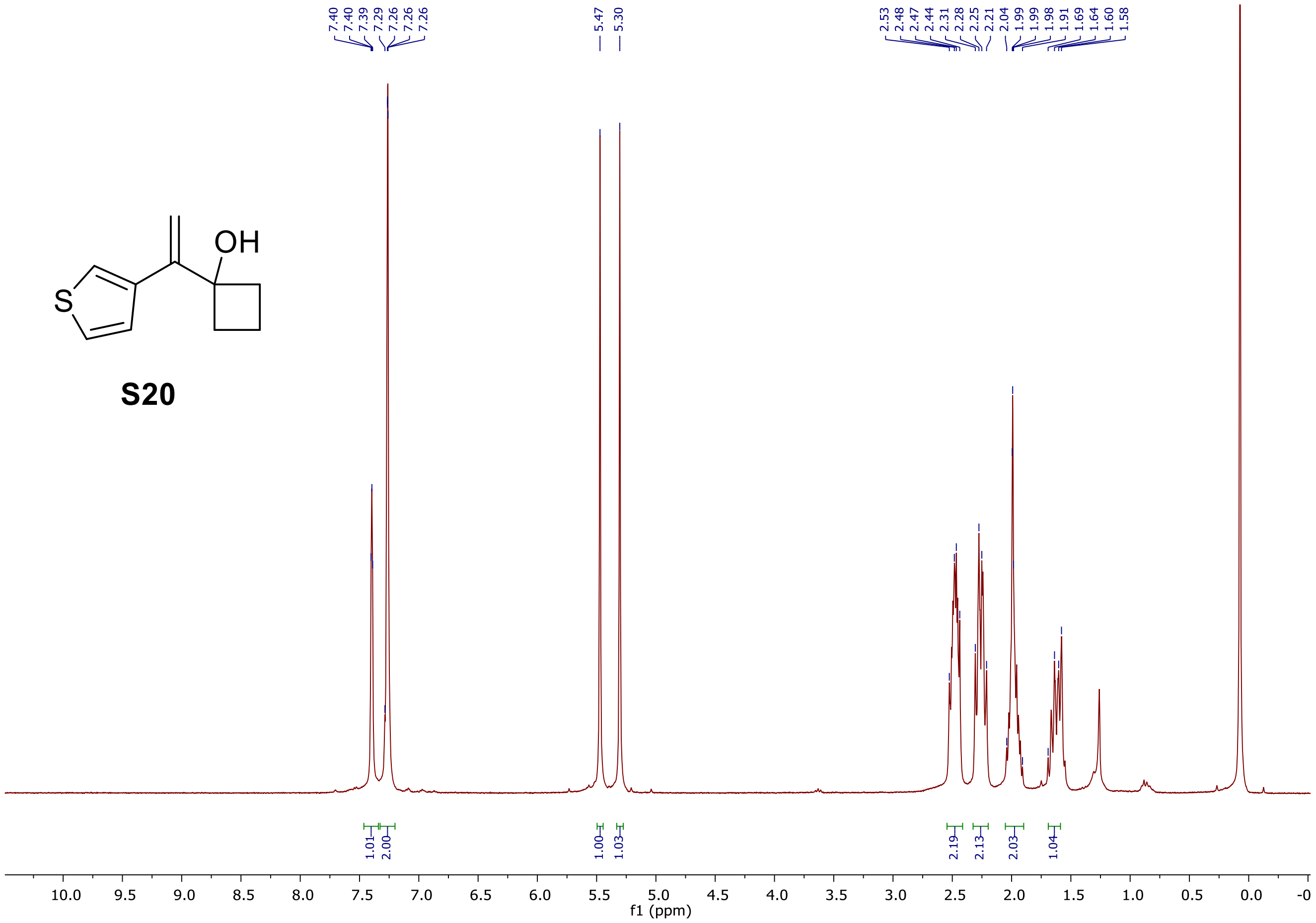
7.40
7.40
7.39
7.29
7.26
7.26
7.26
5.47
5.30
2.53
2.48
2.47
2.44
2.31
2.28
2.25
2.21
2.04
1.99
1.99
1.98
1.91
1.69
1.64
1.60
1.58
OH
S
S20
1.01
2.00
1.00
1.03
2.19
2.13
2.03
1.04
f1 (ppm)

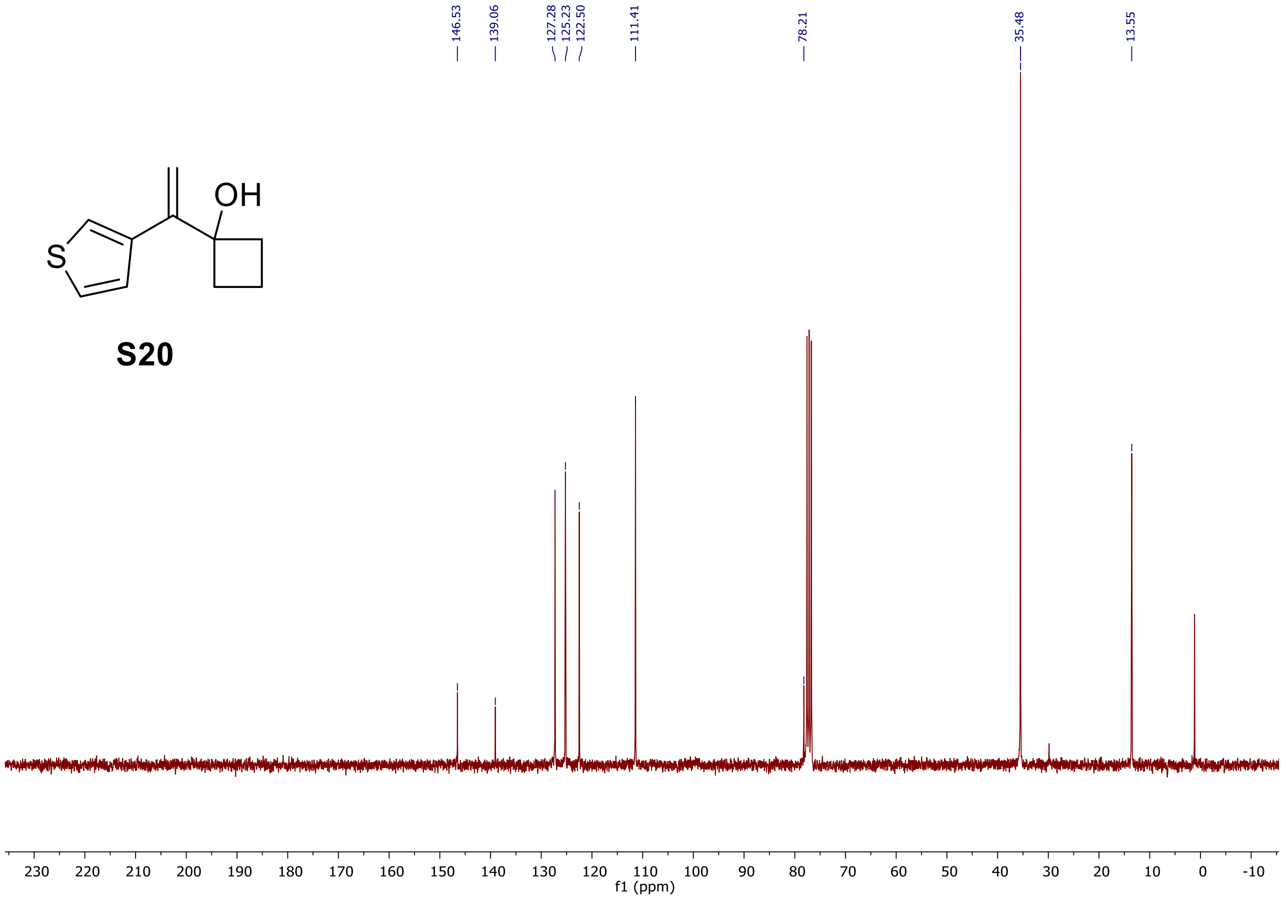
146.53
139.06
127.28
125.23
122.50
111.41
78.21
35.48
13.55
OH
S
S20
f1 (ppm)

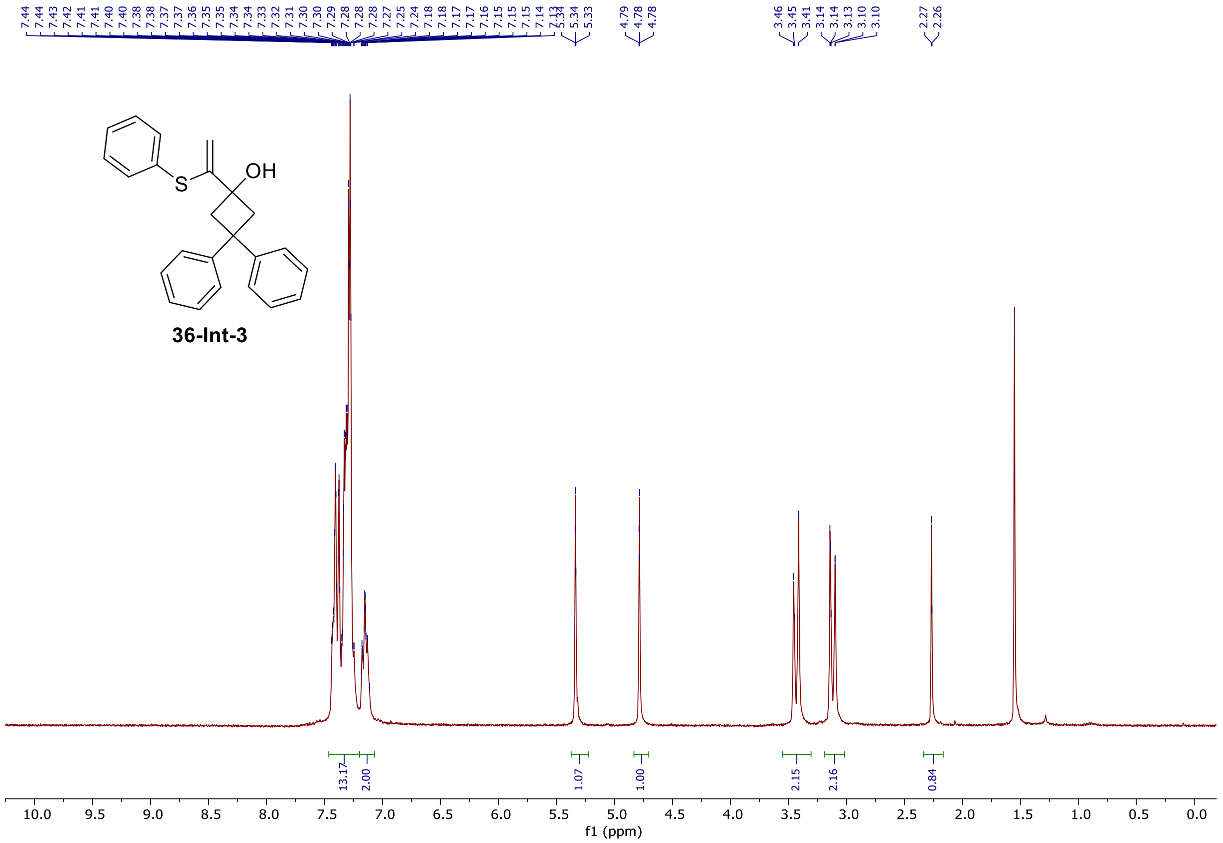
7.44 7.44 7.43 7.42 7.41 7.41 7.40 7.38 7.38 7.37 7.37 7.36 7.35 7.34 7.34 7.33 7.32 7.31 7.30 7.30 7.29 7.28 7.28 7.28 7.27 7.25 7.24 7.18 7.18 7.17 7.17 7.16 7.15 7.15 7.15 7.14 7.13 5.34 5.34 5.33
4.79 4.78 4.78
3.46 3.45 3.41 3.14 3.14 3.13 3.10 3.10
2.27 2.26
OH
S
36-Int-3
13.17 2.00 1.07 1.00 2.15 2.16 0.84
f1 (ppm)

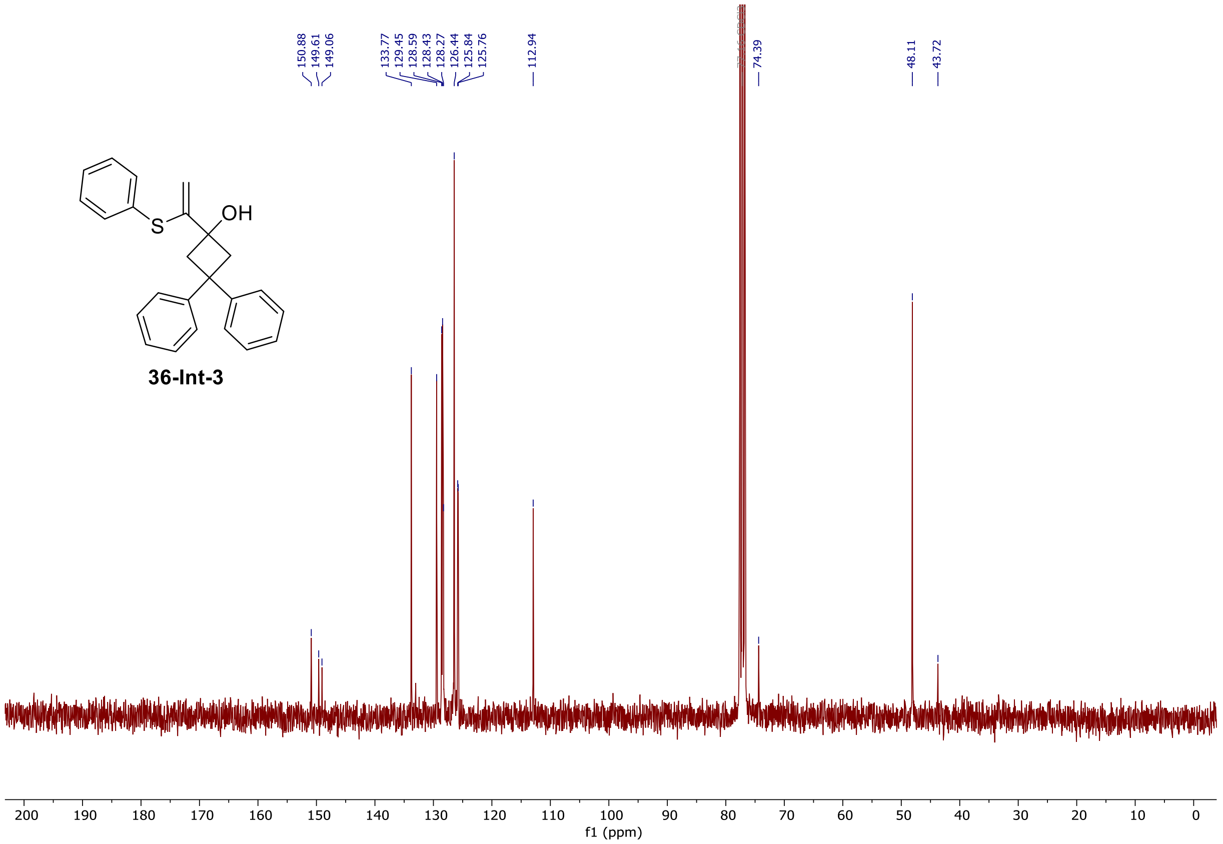
150.88 149.61 149.06
133.77 129.45 128.59 128.43 128.27 126.44 125.84 125.76
112.94
74.39
48.11
43.72
OH
S
36-Int-3
f1 (ppm)

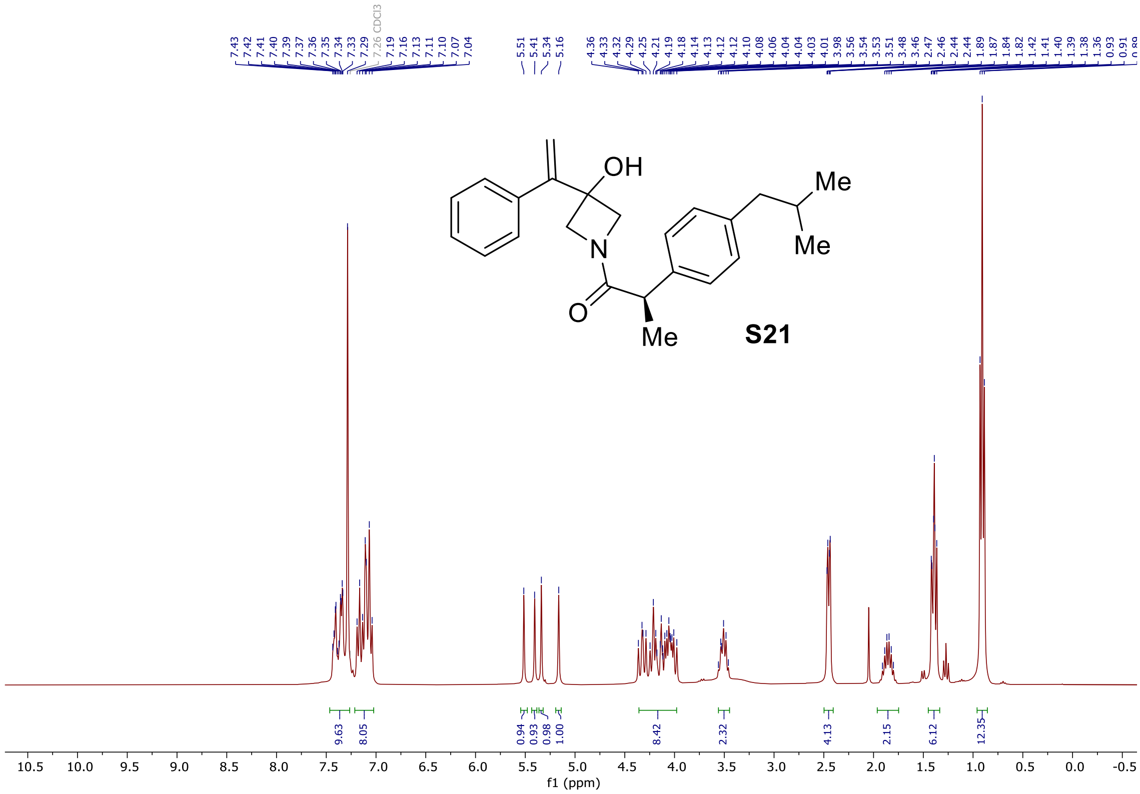
7.43 7.42 7.41 7.39 7.37 7.36 7.35 7.34 7.33 7.29 7.26 CDCl3 7.19 7.16 7.13 7.11 7.10 7.07 7.04
5.51 5.41 5.34 5.16
4.36 4.33 4.32 4.29 4.25 4.21 4.19 4.18 4.14 4.13 4.12 4.12 4.10 4.08 4.06 4.04 4.04 4.03 4.01 3.98 3.56 3.54 3.53 3.51 3.48 3.46 2.47 2.46 2.44 2.44 1.89 1.87 1.84 1.82 1.42 1.41 1.40 1.39 1.38 1.36 0.93 0.91
OH
Me
Me
N
O
Me
S21
9.63 8.05 0.94 0.93 0.98 1.00 8.42 2.32 4.13 2.15 6.12 12.35
10.5 10.0 9.5 9.0 8.5 8.0 7.5 7.0 6.5 6.0 5.5 5.0 4.5 4.0 3.5 3.0 2.5 2.0 1.5 1.0 0.5 0.0 -0.5
f1 (ppm)

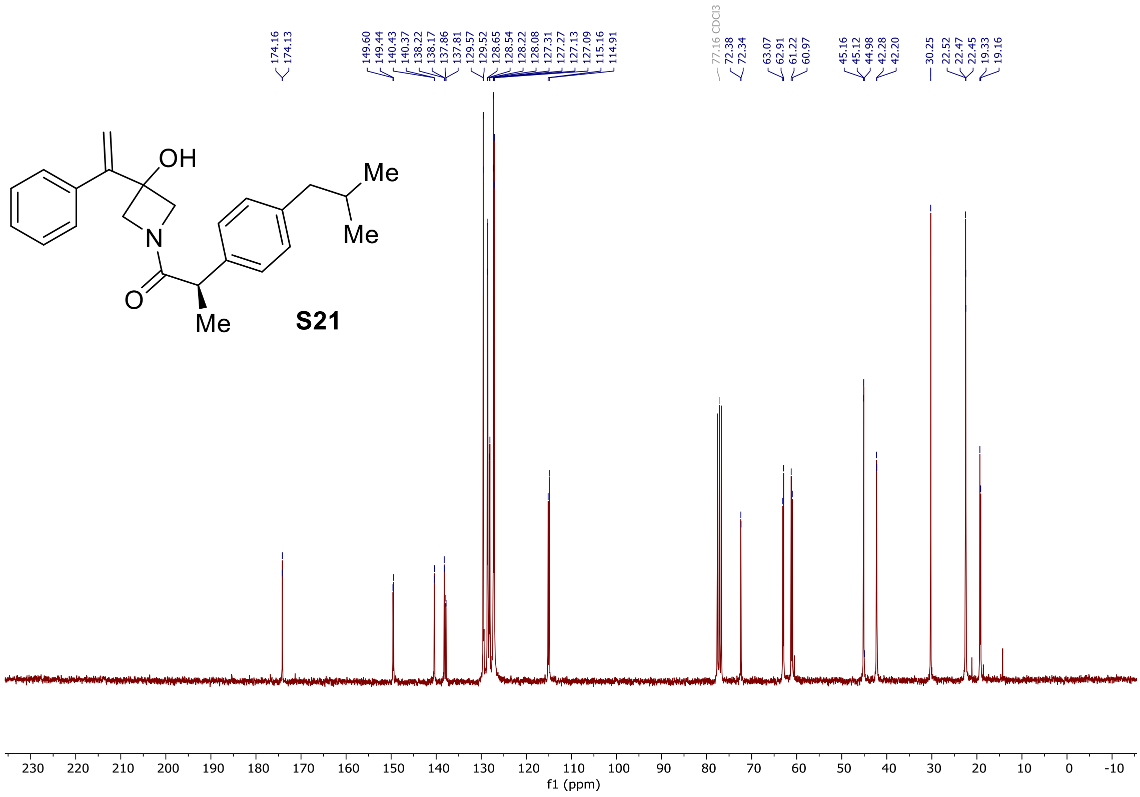
174.16 174.13
149.60 149.44 140.43 140.37 138.22 138.17 137.86 137.81 129.57 129.52 128.65 128.54 128.22 128.08 127.31 127.27 127.13 127.09 115.16 114.91
77.16 CDCl3 72.38 72.34 63.07 62.91 61.22 60.97
45.16 45.12 44.98 42.28 42.20 30.25 22.52 22.47 22.45 19.33 19.16
OH
Me
Me
N
O
Me
S21
230 220 210 200 190 180 170 160 150 140 130 120 110 100 90 80 70 60 50 40 30 20 10 0 -10
f1 (ppm)

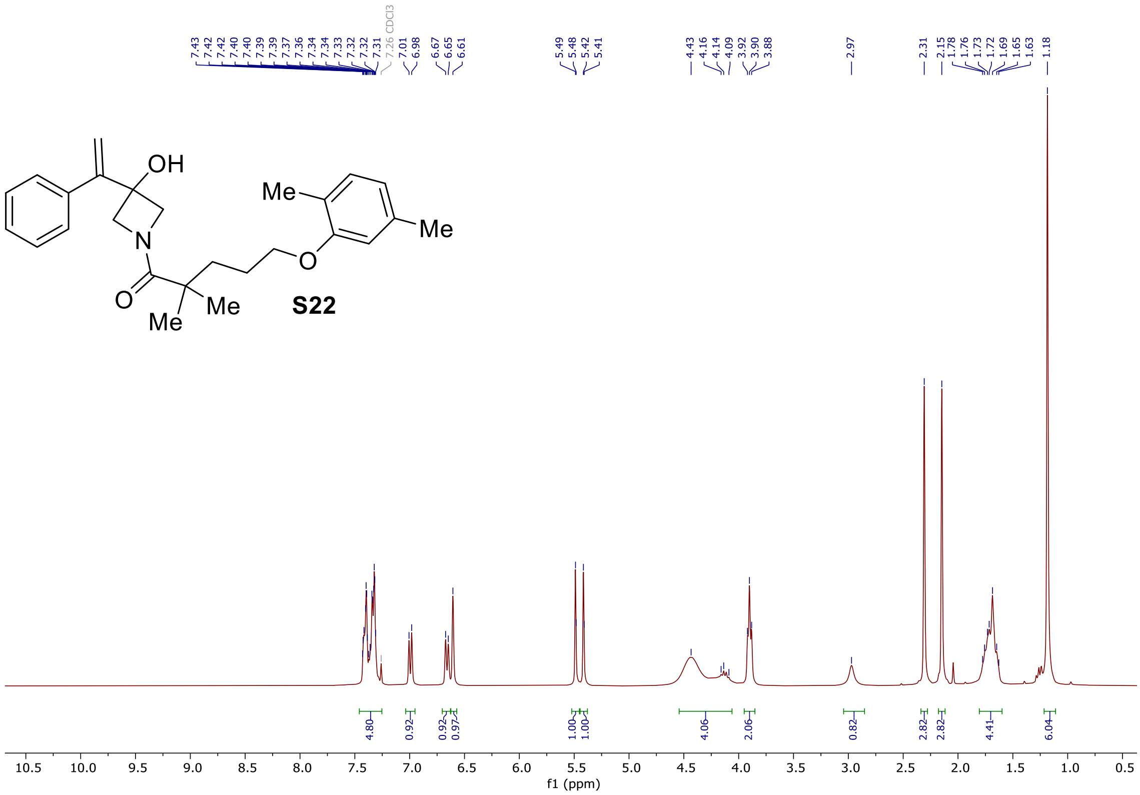
7.43 7.42 7.42 7.40 7.39 7.39 7.37 7.36 7.34 7.34 7.33 7.32 7.32 7.31
7.26 CDCl3
7.01 6.98 6.67 6.65 6.61
5.49 5.48 5.42 5.41
4.43 4.16 4.14 4.09 3.92 3.90 3.88
2.97
2.31 2.15 1.78 1.76 1.73 1.72 1.69 1.65 1.63
1.18
OH
Me
Me
N
O
O
Me
Me
S22
4.80 0.92 0.92 0.97 1.00 1.00 4.06 2.06 0.82 2.82 2.82 4.41 6.04
f1 (ppm)

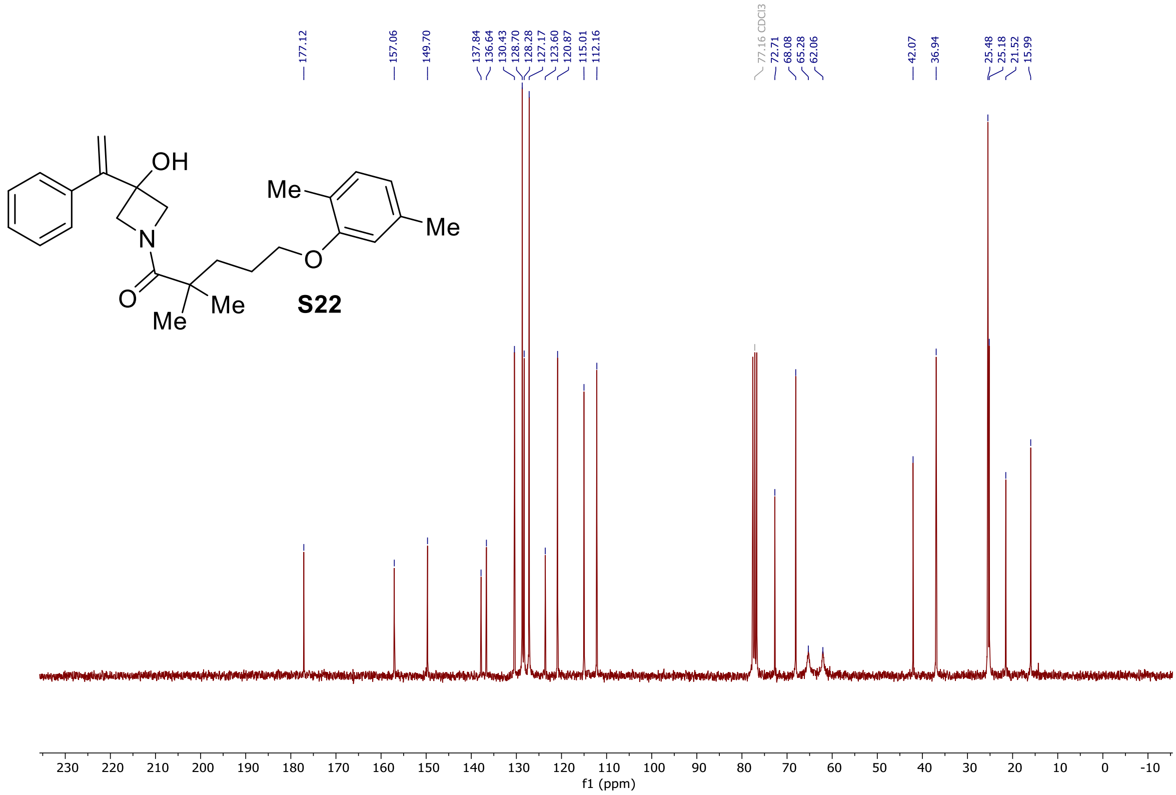
177.12
157.06
149.70
137.84 136.64 130.43 128.70 128.28 127.17 123.60 120.87 115.01 112.16
77.16 CDCl3
72.71 68.08 65.28 62.06
42.07 36.94
25.48 25.18 21.52 15.99
OH
Me
Me
N
O
O
Me
Me
S22
f1 (ppm)

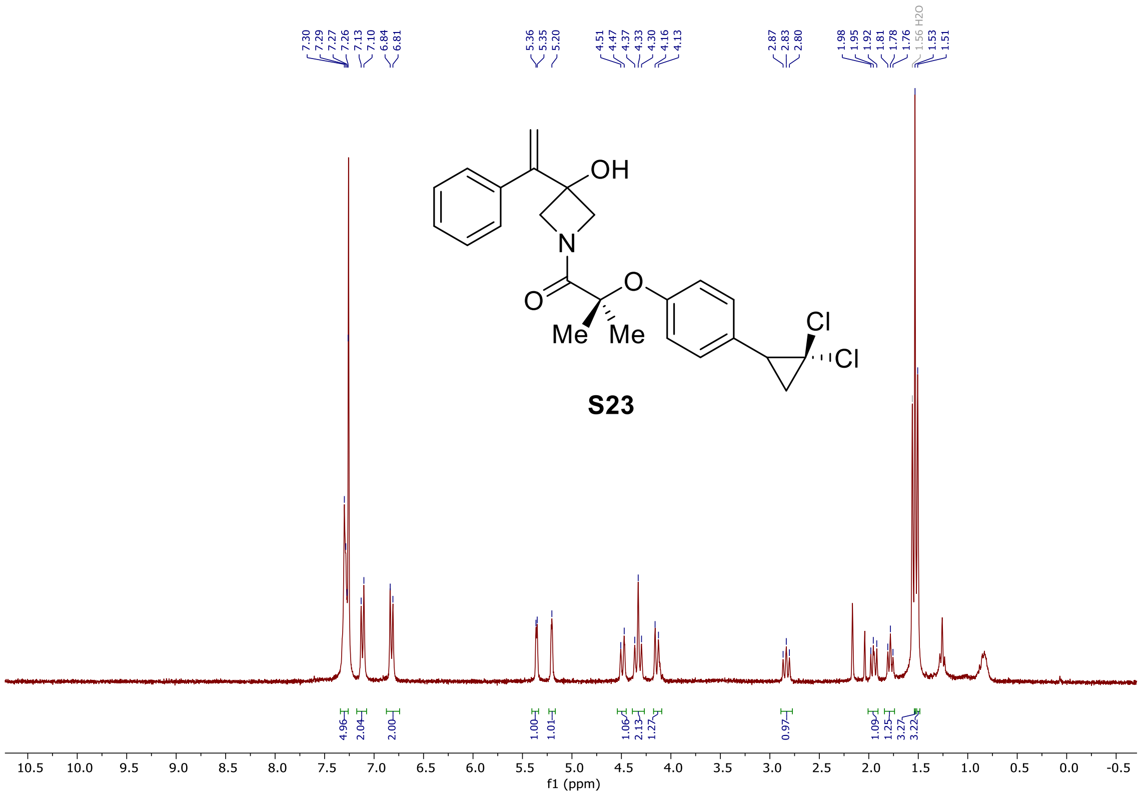
OH
N
O
O
Me
Me
Cl
Cl
S23
f1 (ppm)

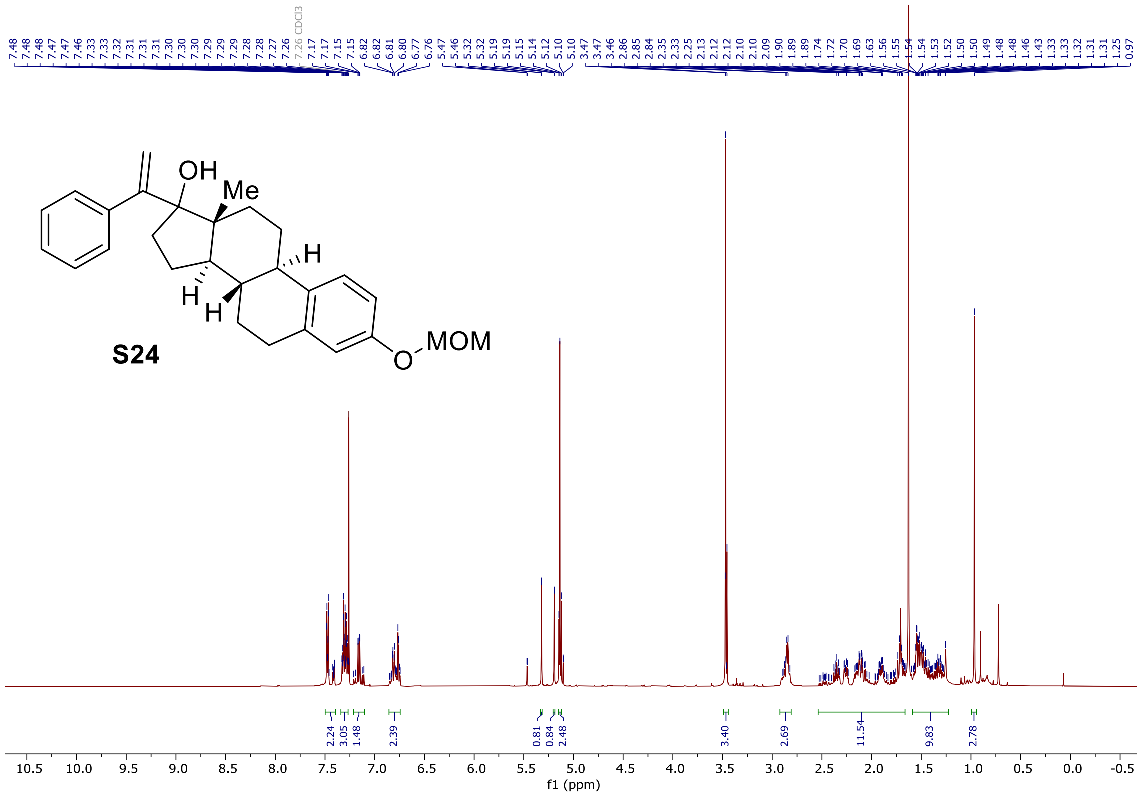

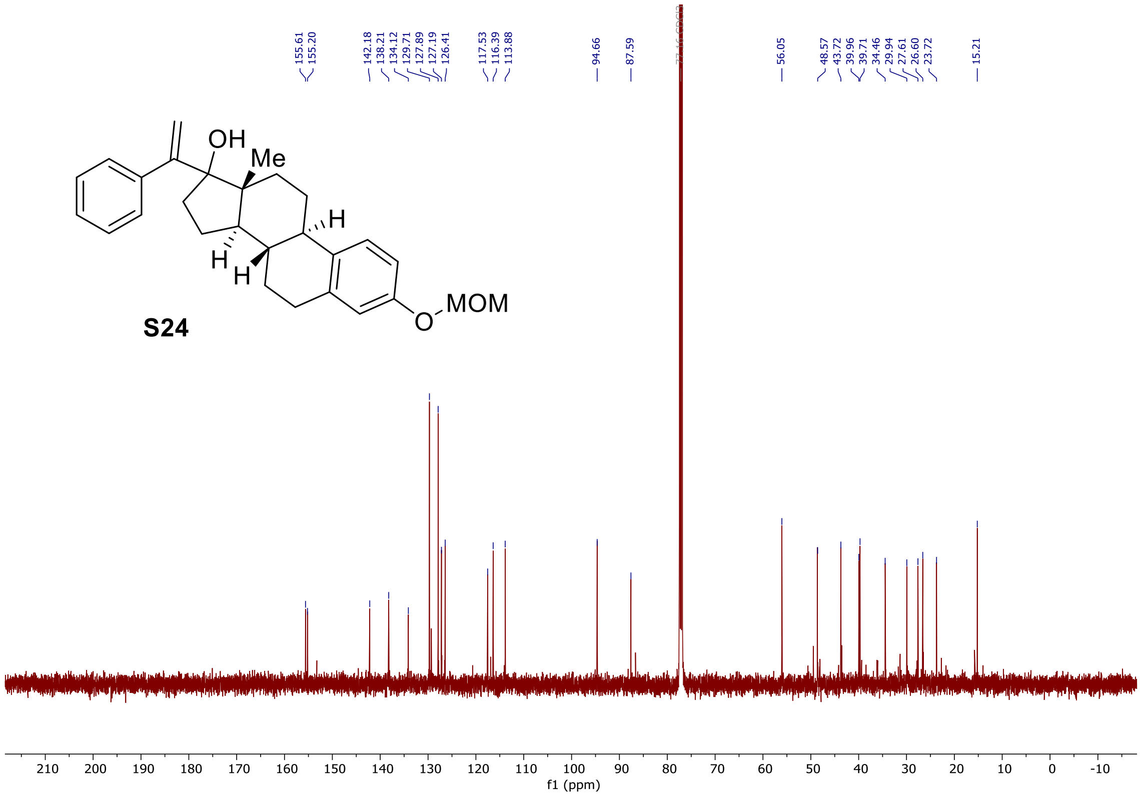

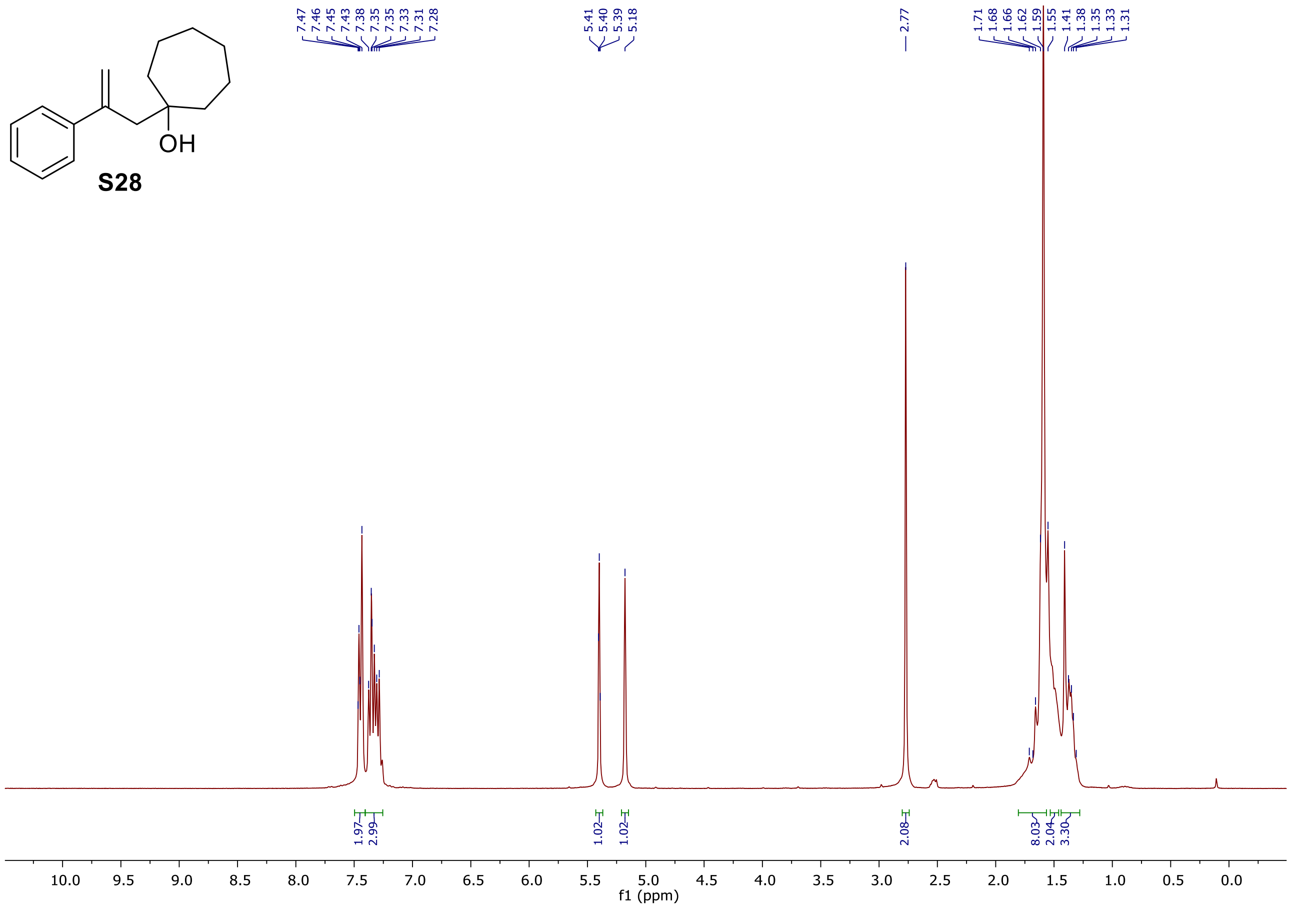
S28
OH
7.47 7.46 7.45 7.43 7.38 7.35 7.35 7.33 7.31 7.28
5.41 5.40 5.39 5.18
2.77
1.71 1.68 1.66 1.62 1.59 1.55 1.41 1.38 1.35 1.33 1.31
1.97 2.99 1.02 1.02 2.08 8.03 2.04 3.30
f1 (ppm)

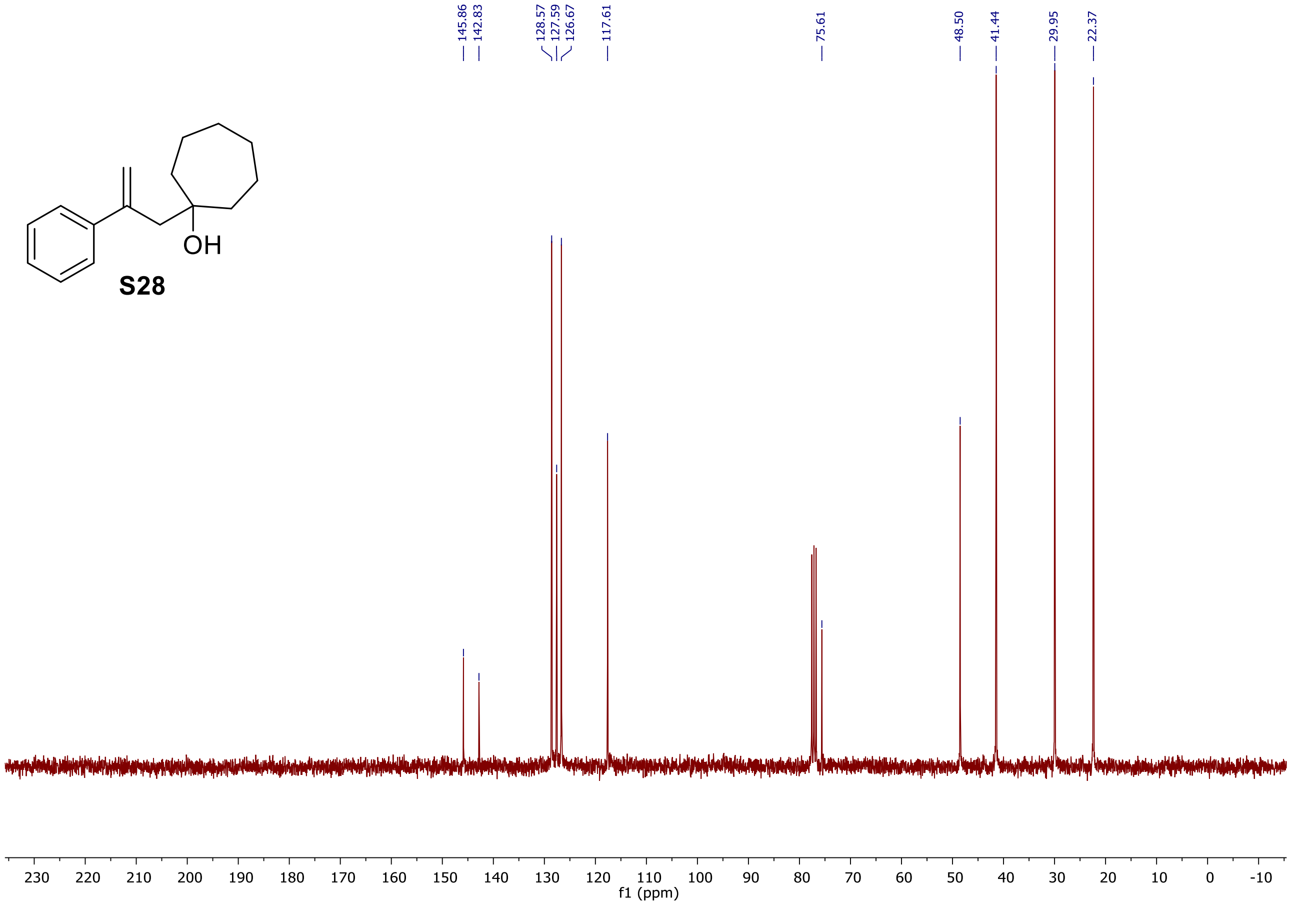
S28
OH
145.86 142.83 128.57 127.59 126.67 117.61 75.61 48.50 41.44 29.95 22.37
f1 (ppm)

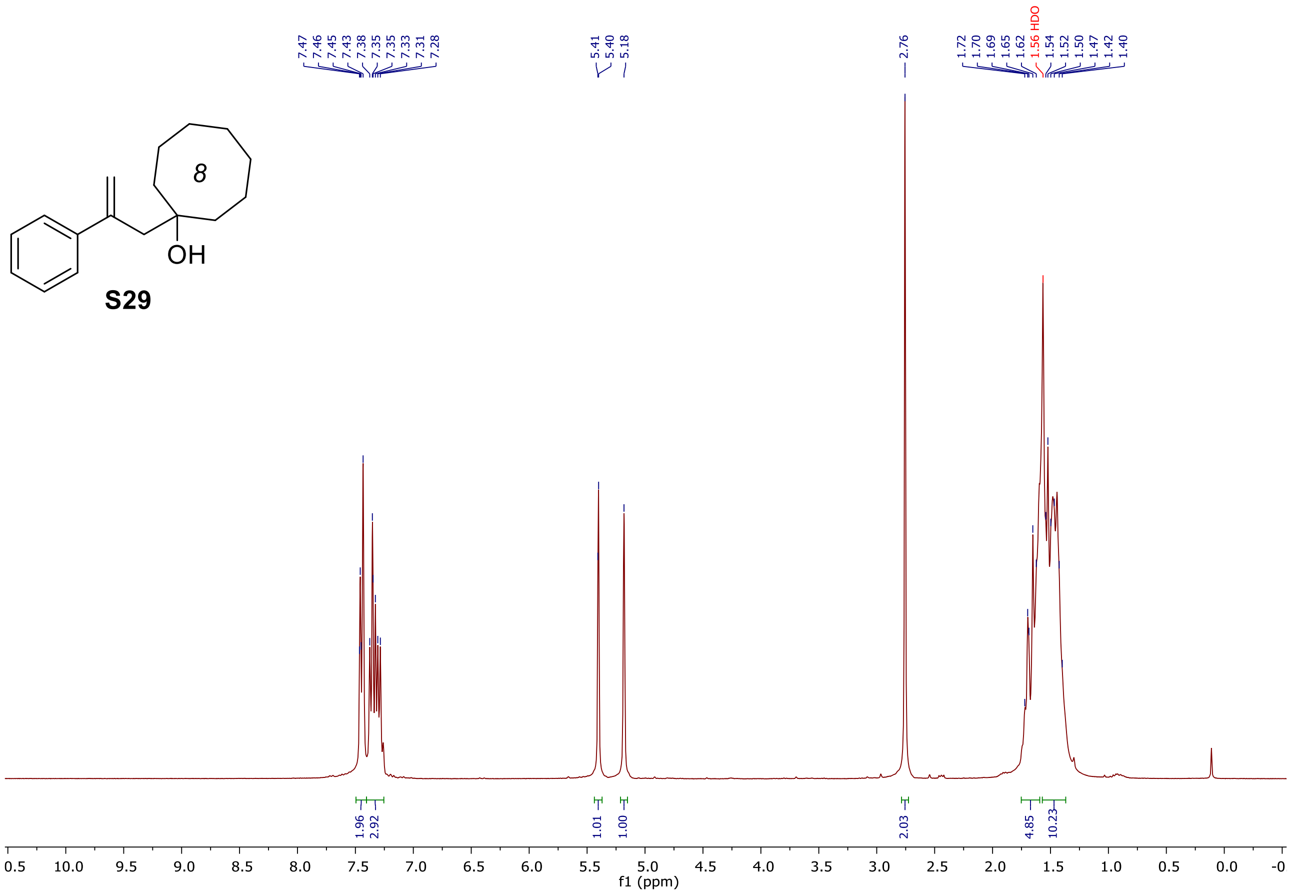

7.47
7.46
7.45
7.43
7.38
7.35
7.35
7.33
7.31
7.28
5.41
5.40
5.18
2.76
1.72
1.70
1.69
1.65
1.62
1.56 HDO
1.54
1.52
1.50
1.47
1.42
1.40
8
OH
S29
1.96
2.92
1.01
1.00
2.03
4.85
10.23
f1 (ppm)

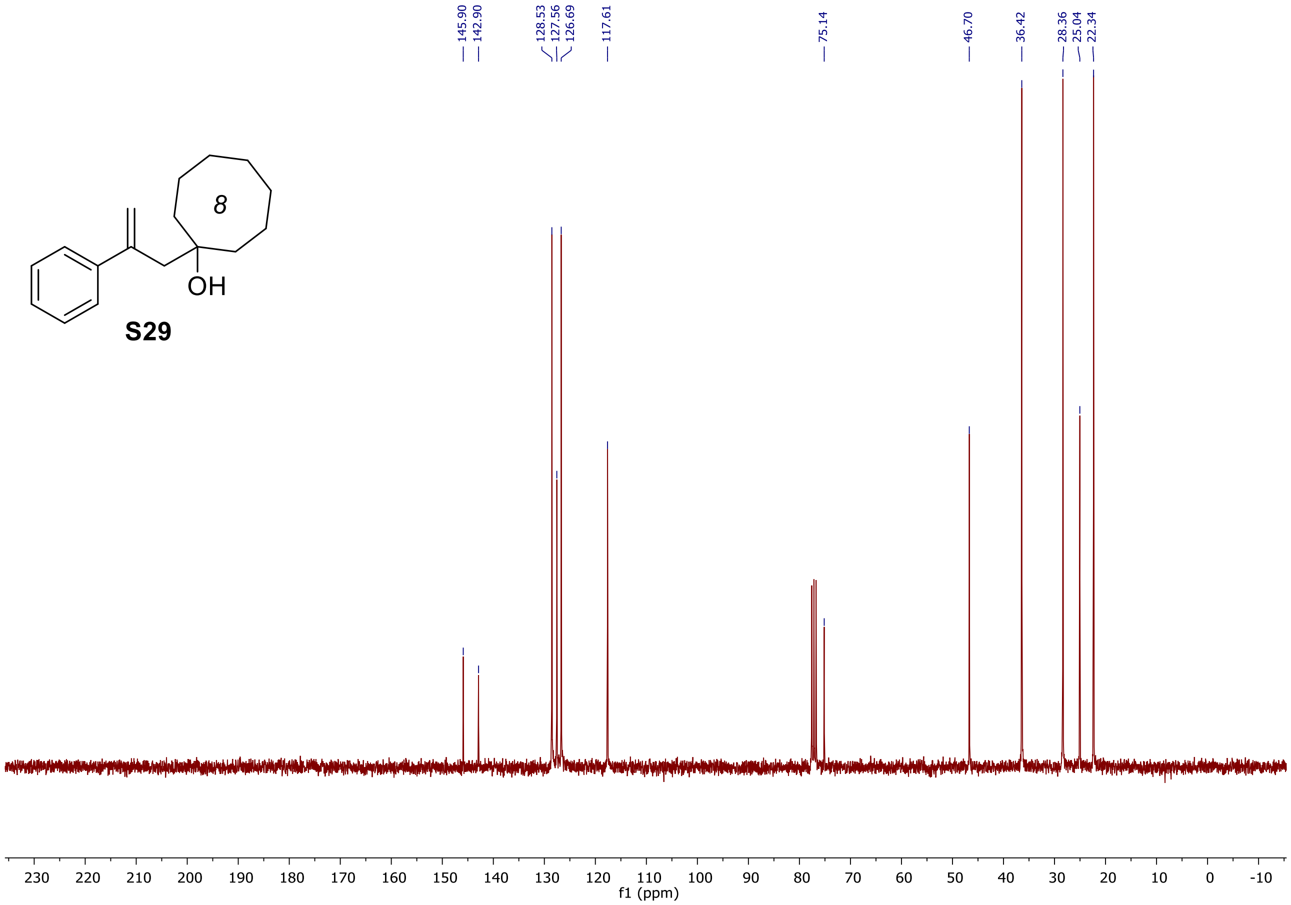

145.90
142.90
128.53
127.56
126.69
117.61
75.14
46.70
36.42
28.36
25.04
22.34
8
OH
S29
f1 (ppm)

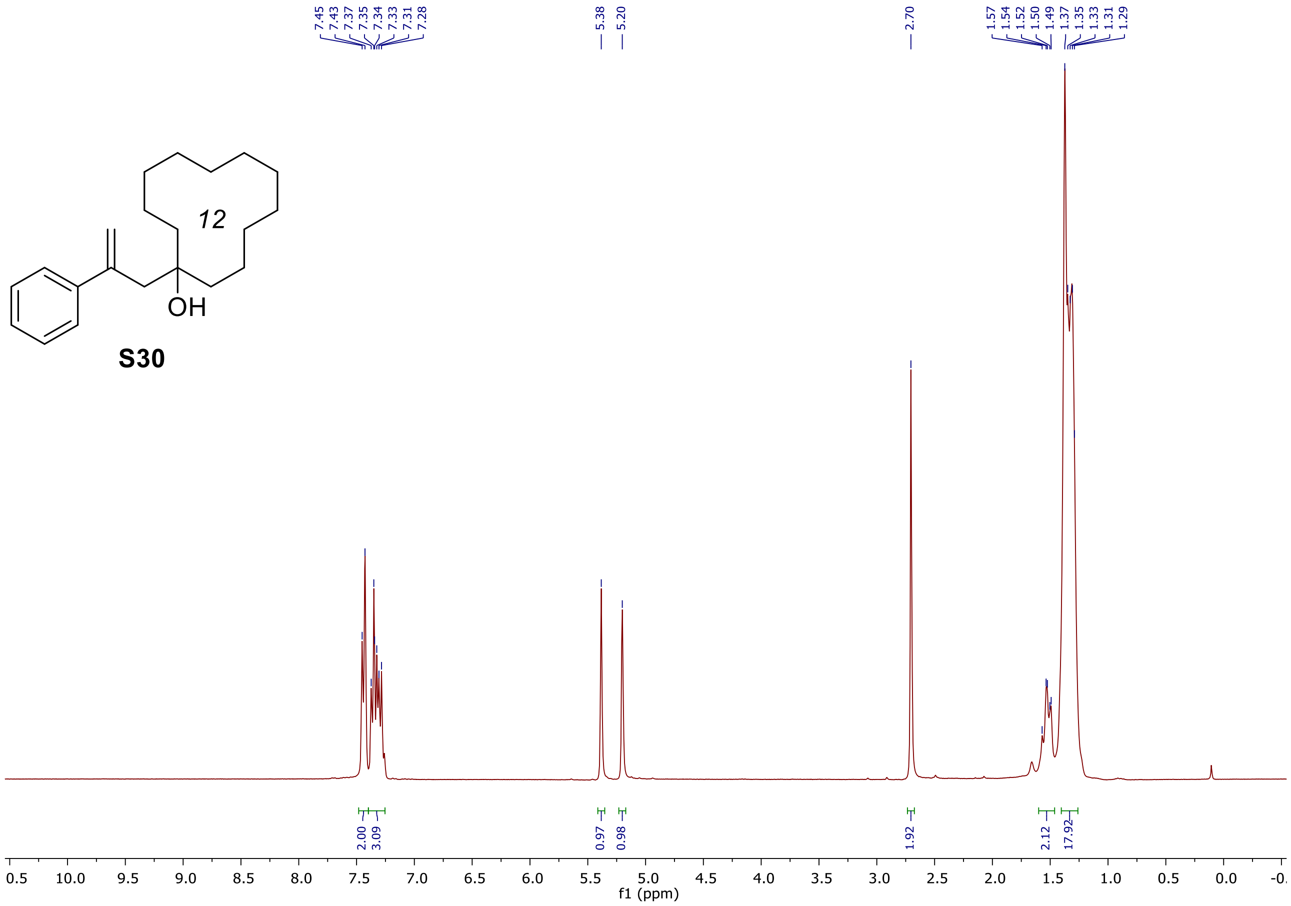
7.45 7.43 7.37 7.35 7.34 7.33 7.31 7.28
5.38 5.20
2.70
1.57 1.54 1.52 1.50 1.49 1.37 1.35 1.33 1.31 1.29
12
OH
S30
2.00 3.09 0.97 0.98 1.92 2.12 17.92
0.5 10.0 9.5 9.0 8.5 8.0 7.5 7.0 6.5 6.0 5.5 5.0 4.5 4.0 3.5 3.0 2.5 2.0 1.5 1.0 0.5 0.0
f1 (ppm)

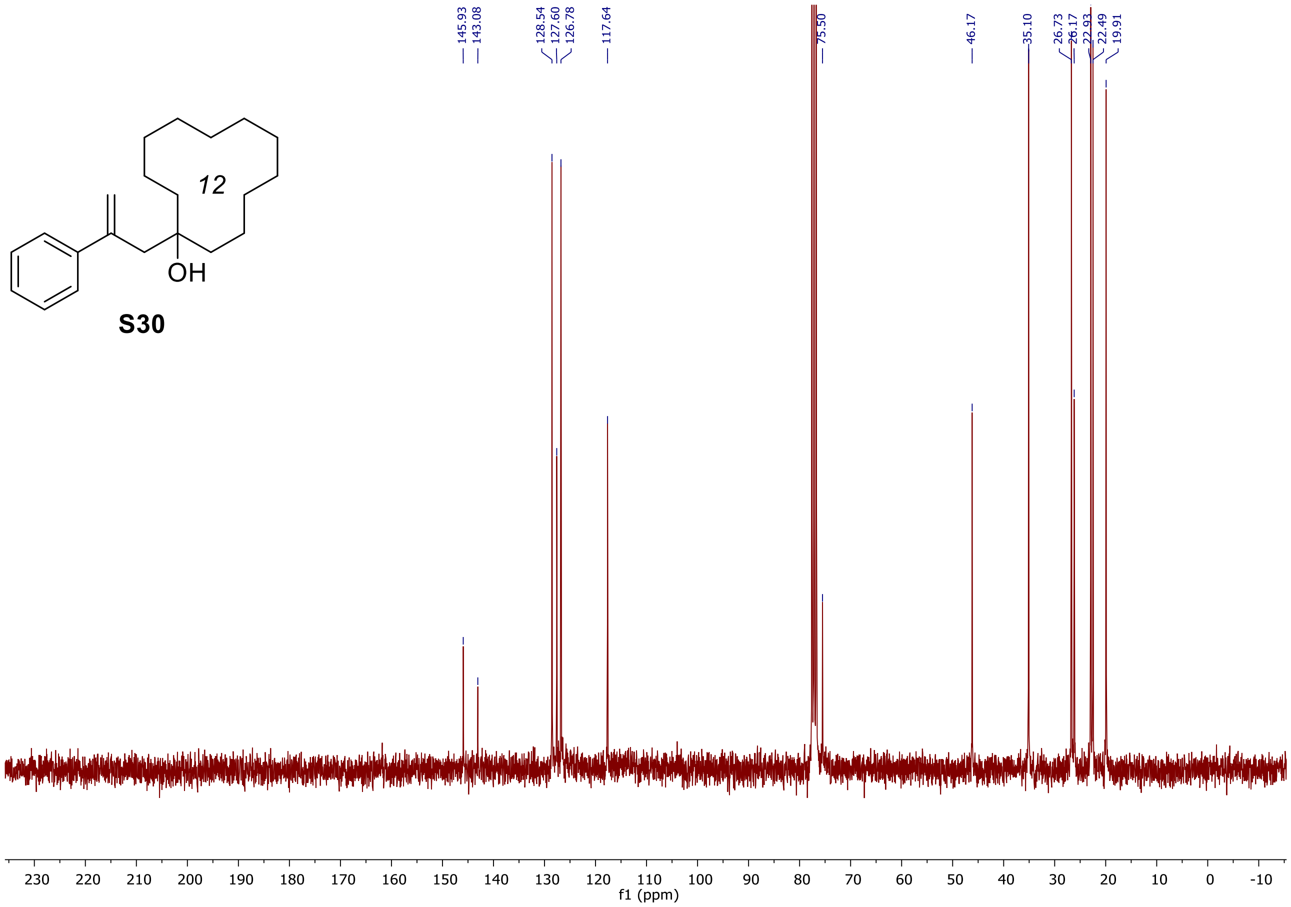
145.93 143.08
128.54 127.60 126.78
117.64
75.50
46.17
35.10 26.73 26.17 22.93 22.49 19.91
12
OH
S30
230 220 210 200 190 180 170 160 150 140 130 120 110 100 90 80 70 60 50 40 30 20 10 0 -10
f1 (ppm)

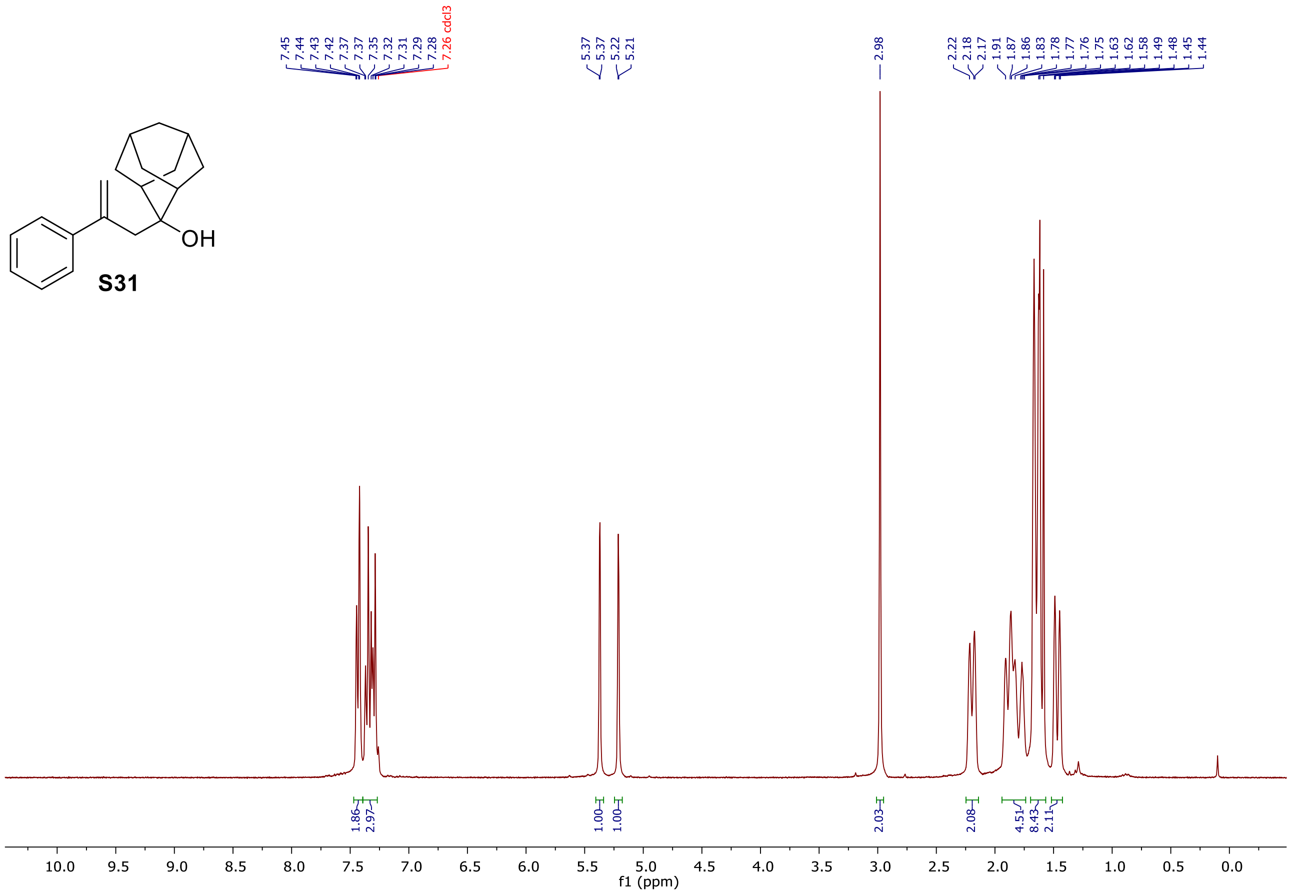
7.45
7.44
7.43
7.42
7.37
7.37
7.35
7.32
7.31
7.29
7.28
7.26 cdcl3
5.37
5.37
5.22
5.21
2.98
2.22
2.18
2.17
1.91
1.87
1.86
1.83
1.78
1.77
1.76
1.75
1.63
1.62
1.58
1.49
1.48
1.45
1.44
OH
S31
1.86
2.97
1.00
1.00
2.03
2.08
4.51
8.43
2.11
10.0 9.5 9.0 8.5 8.0 7.5 7.0 6.5 6.0 5.5 5.0 4.5 4.0 3.5 3.0 2.5 2.0 1.5 1.0 0.5 0.0
f1 (ppm)

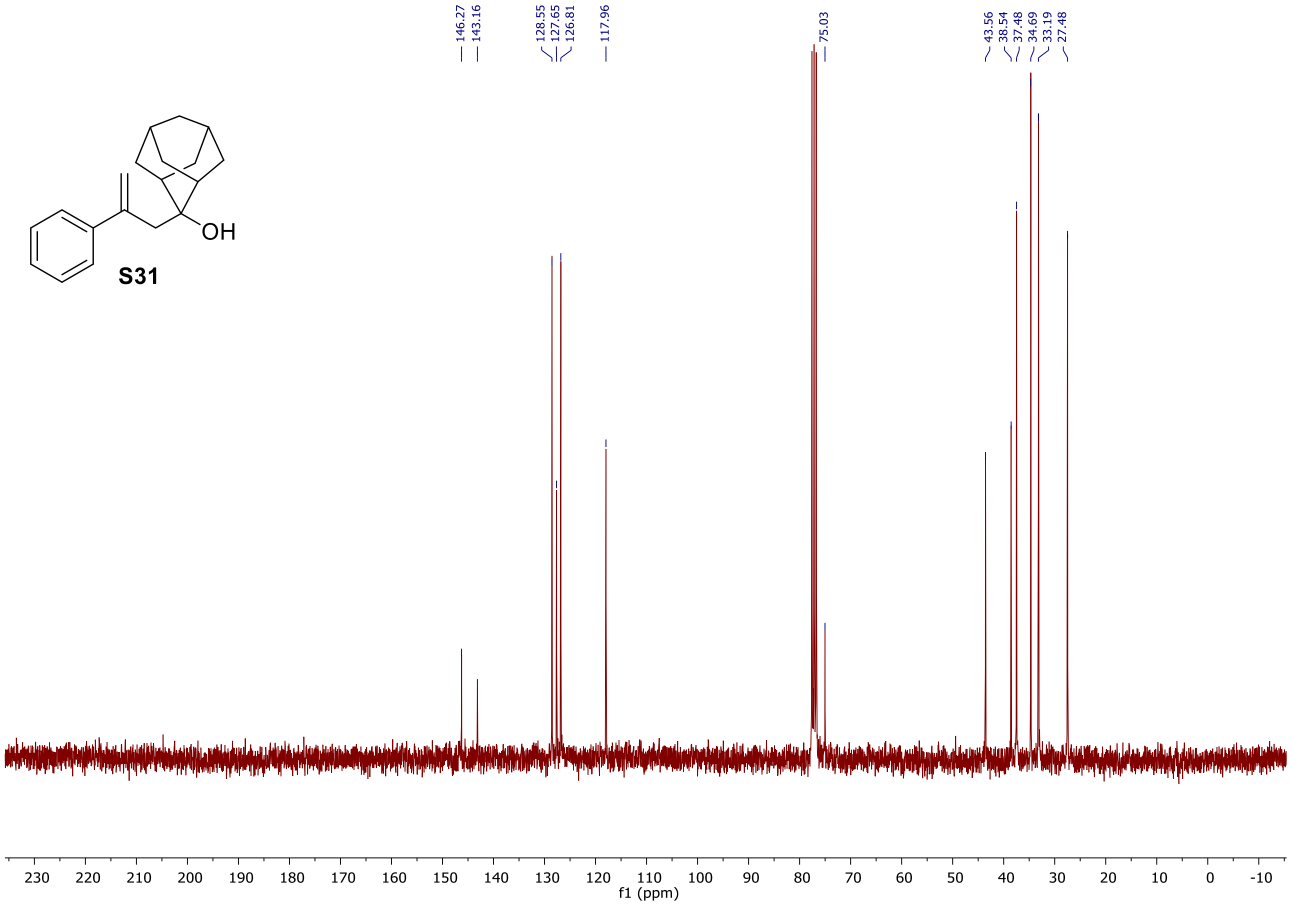
146.27
143.16
128.55
127.65
126.81
117.96
75.03
43.56
38.54
37.48
34.69
33.19
27.48
OH
S31
230 220 210 200 190 180 170 160 150 140 130 120 110 100 90 80 70 60 50 40 30 20 10 0 -10
f1 (ppm)

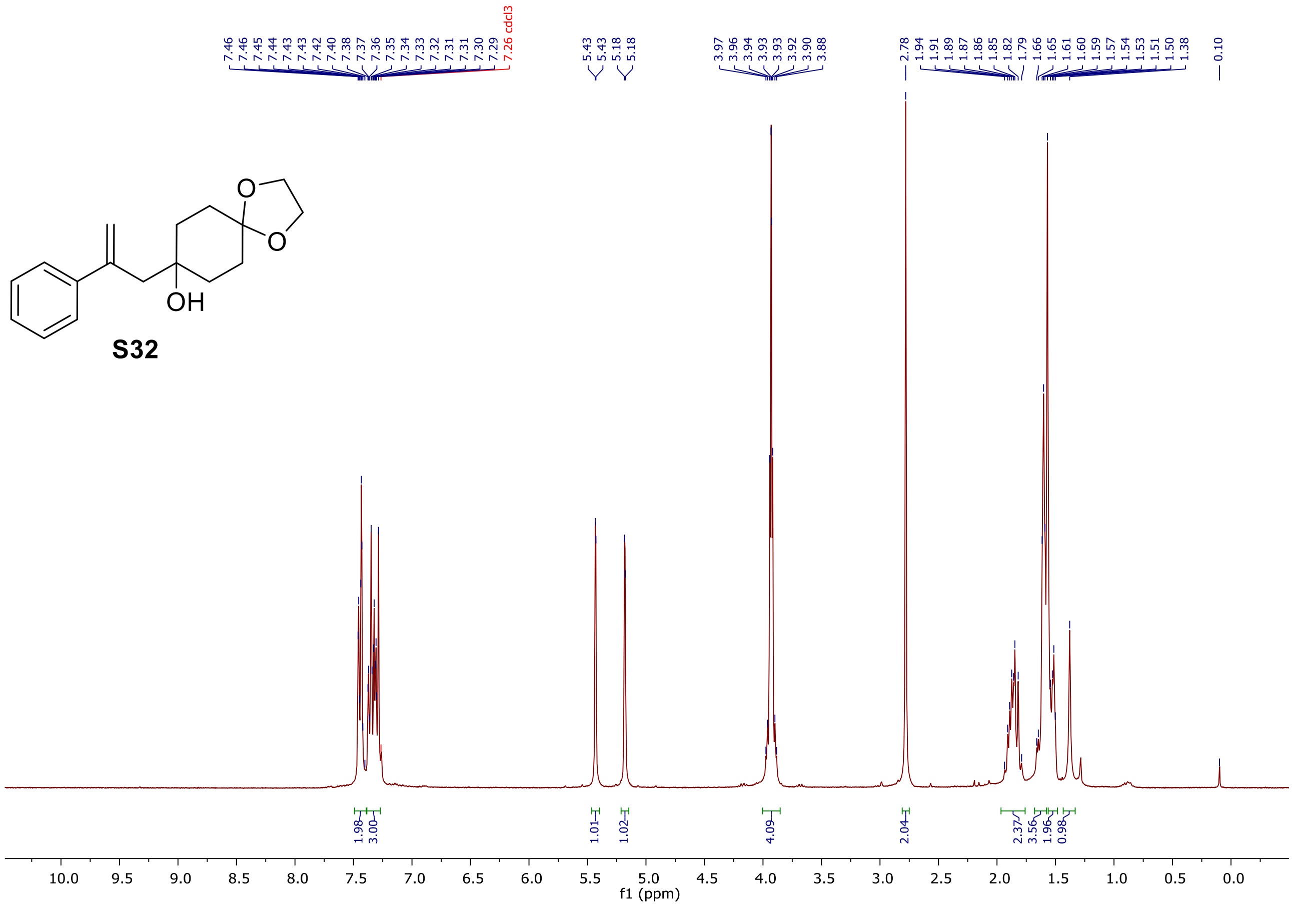
7.46
7.46
7.45
7.44
7.43
7.43
7.42
7.40
7.38
7.37
7.36
7.35
7.34
7.33
7.32
7.31
7.31
7.30
7.29
7.26 cdcl3
5.43
5.43
5.18
5.18
3.97
3.96
3.94
3.93
3.93
3.92
3.90
3.88
2.78
1.94
1.91
1.89
1.87
1.86
1.85
1.82
1.79
1.66
1.65
1.61
1.60
1.59
1.57
1.54
1.53
1.51
1.50
1.38
0.10
O
O
OH
S32
1.98
3.00
1.01
1.02
4.09
2.04
2.37
3.56
1.96
0.98
10.0
9.5
9.0
8.5
8.0
7.5
7.0
6.5
6.0
5.5
5.0
4.5
4.0
3.5
3.0
2.5
2.0
1.5
1.0
0.5
0.0
f1 (ppm)

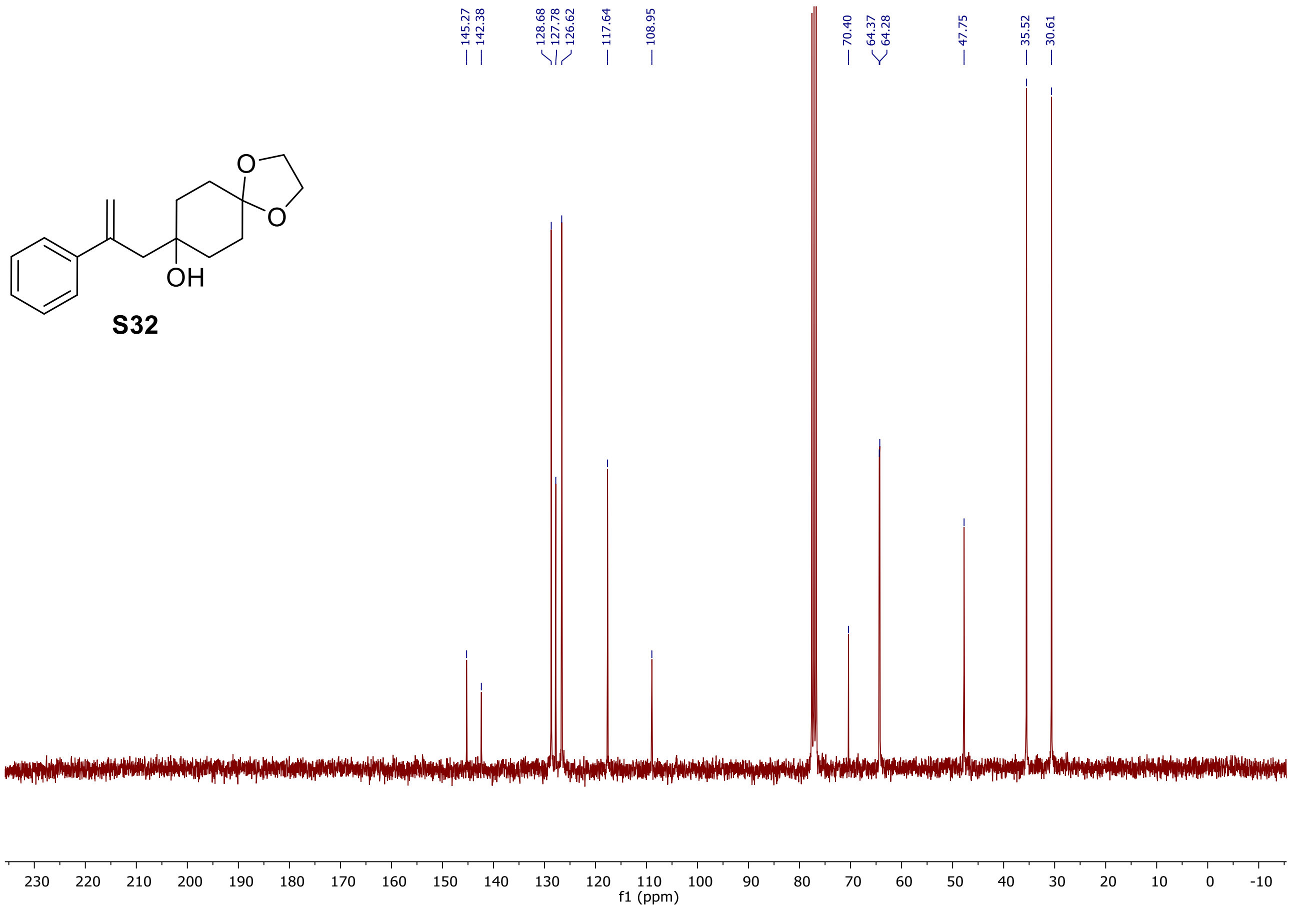
145.27
142.38
128.68
127.78
126.62
117.64
108.95
70.40
64.37
64.28
47.75
35.52
30.61
O
O
OH
S32
230
220
210
200
190
180
170
160
150
140
130
120
110
100
90
80
70
60
50
40
30
20
10
0
-10
f1 (ppm)

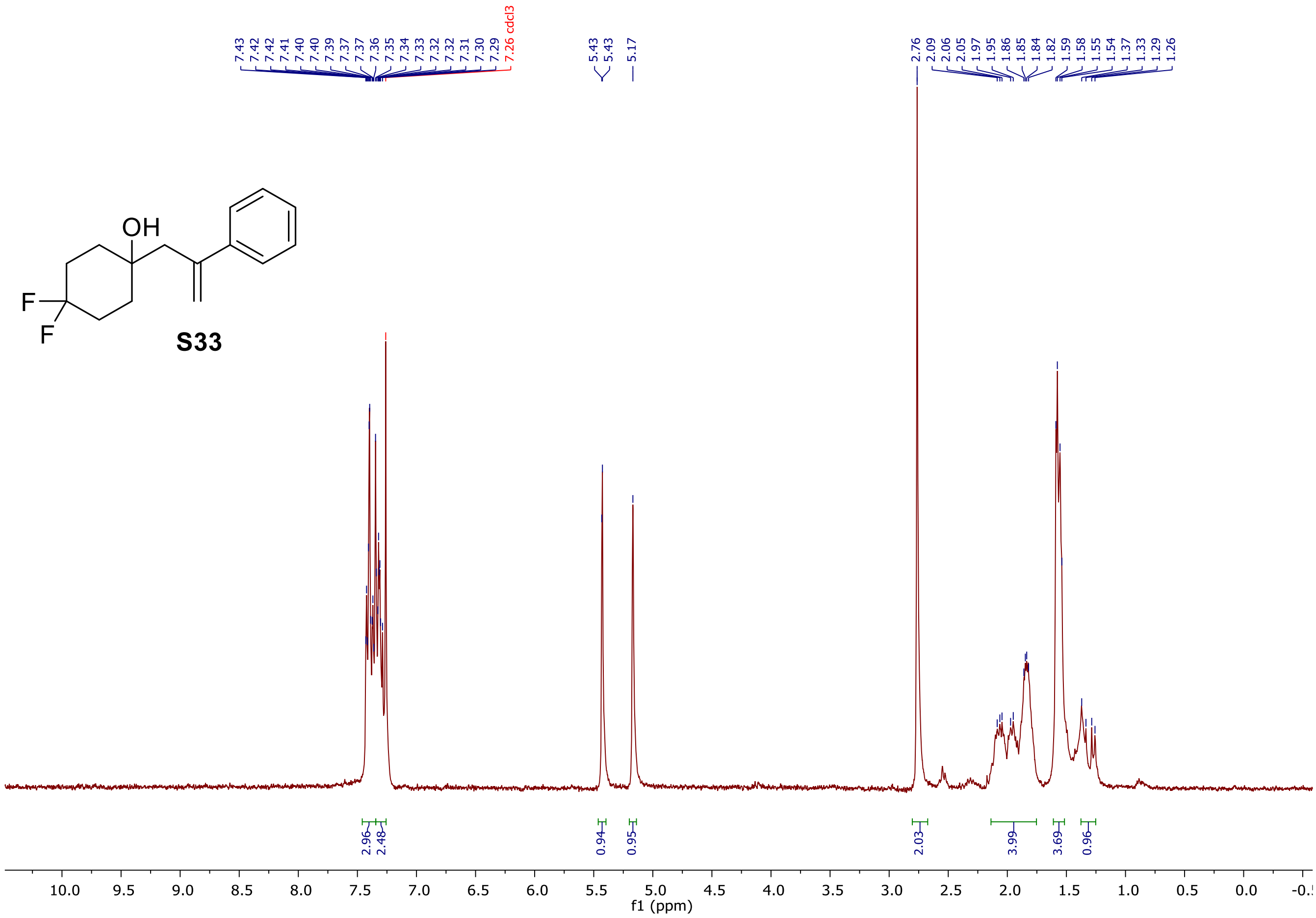

7.43
7.42
7.42
7.41
7.40
7.40
7.39
7.37
7.37
7.36
7.35
7.34
7.33
7.32
7.32
7.31
7.30
7.29
7.26 cdcl3
5.43
5.43
5.17
2.76
2.09
2.06
2.05
1.97
1.95
1.86
1.85
1.84
1.82
1.59
1.58
1.55
1.54
1.37
1.33
1.29
1.26
OH
F
F
2.96
2.48
0.94
0.95
2.03
3.99
3.69
0.96
f1 (ppm)

S33

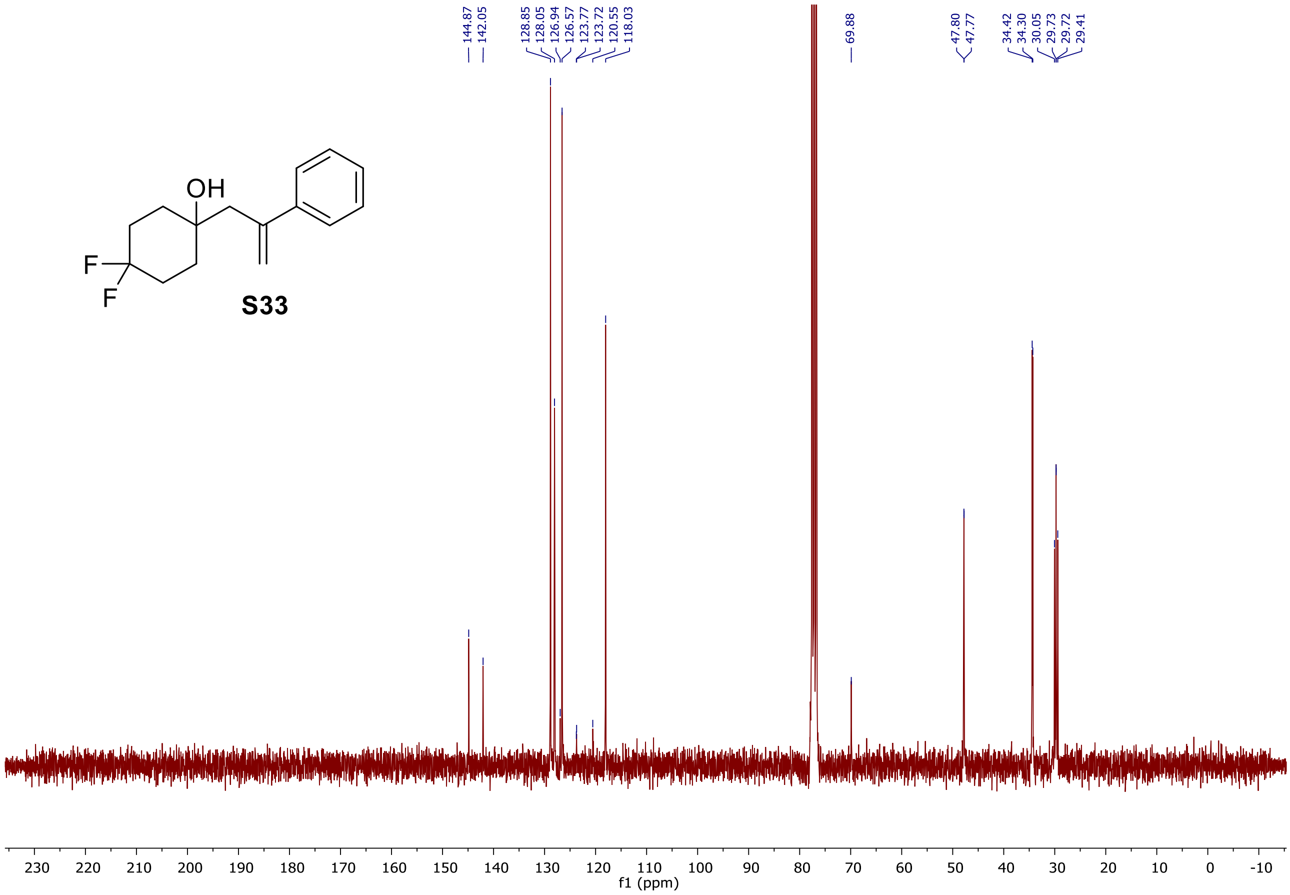

144.87
142.05
128.85
128.05
126.94
126.57
123.77
123.72
120.55
118.03
69.88
47.80
47.77
34.42
34.30
30.05
29.73
29.72
29.41
OH
F
F
f1 (ppm)

S33

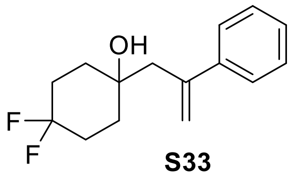

S33

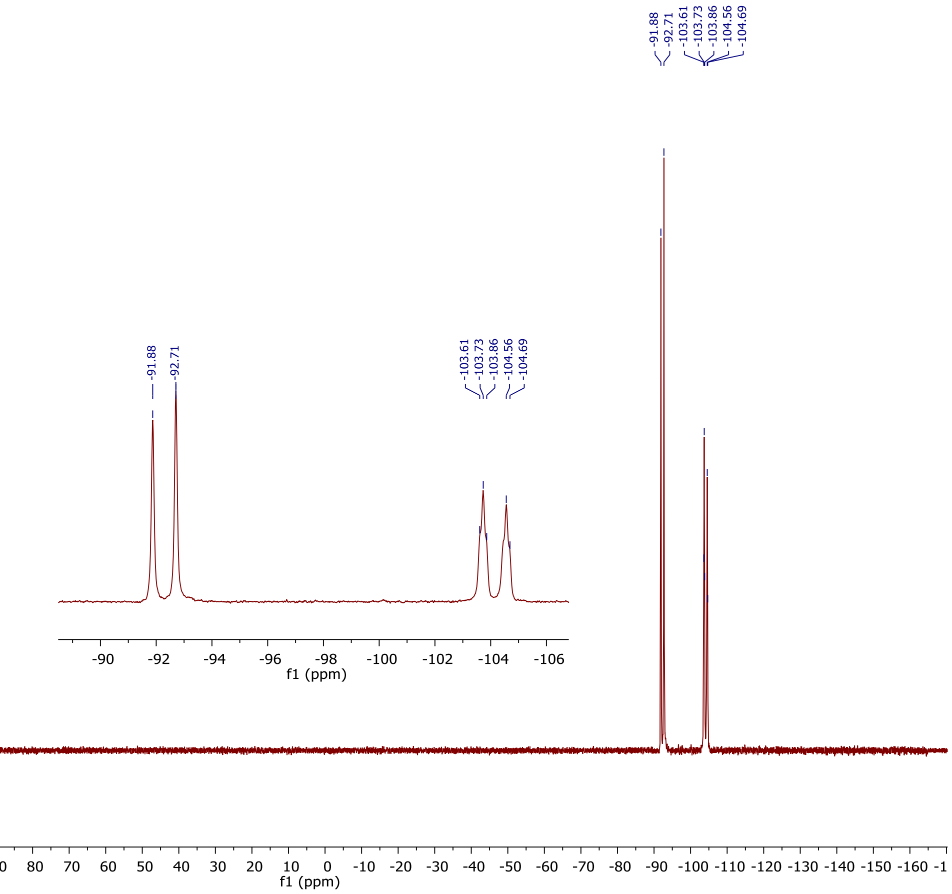

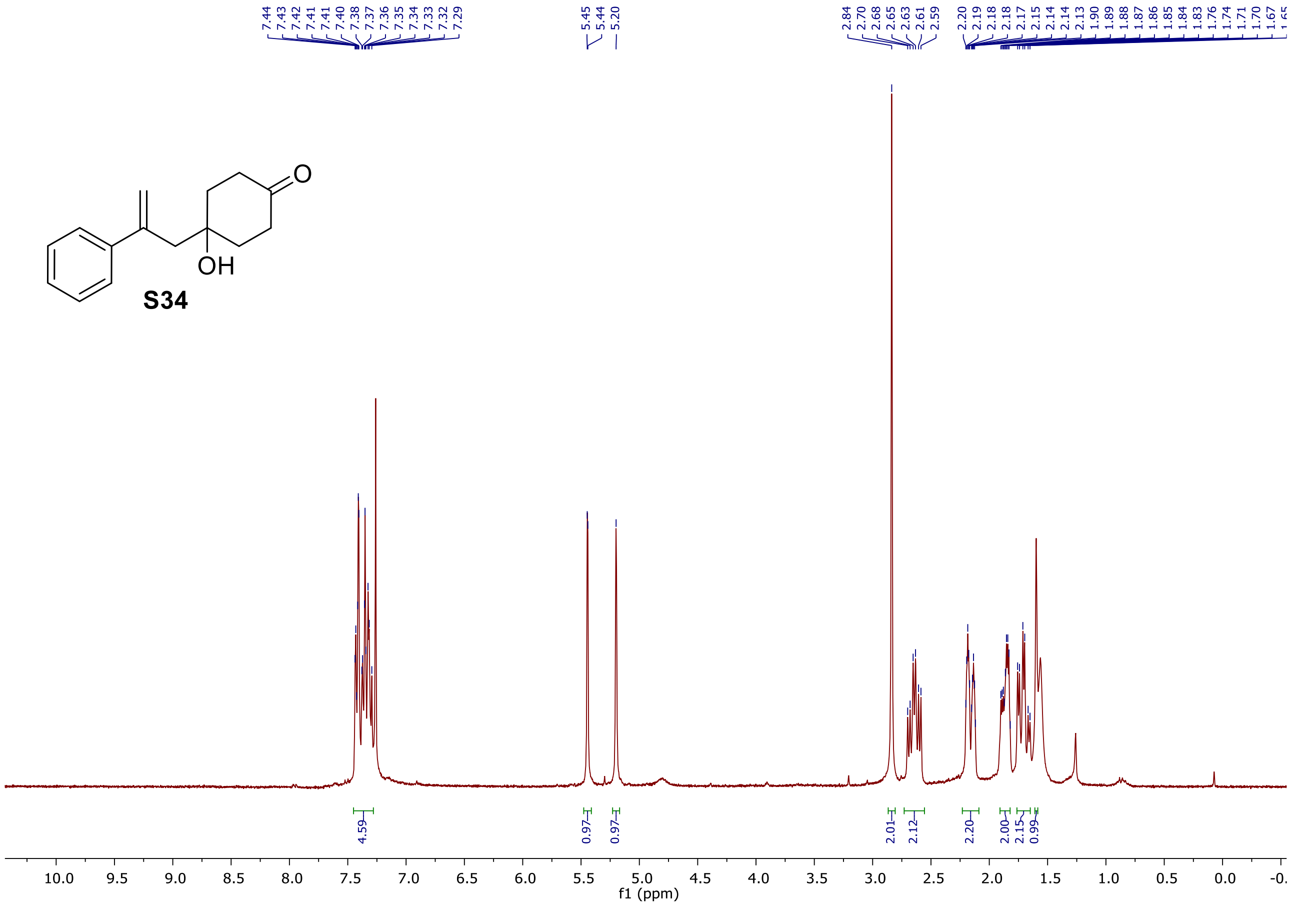
S34
OH
O
f1 (ppm)

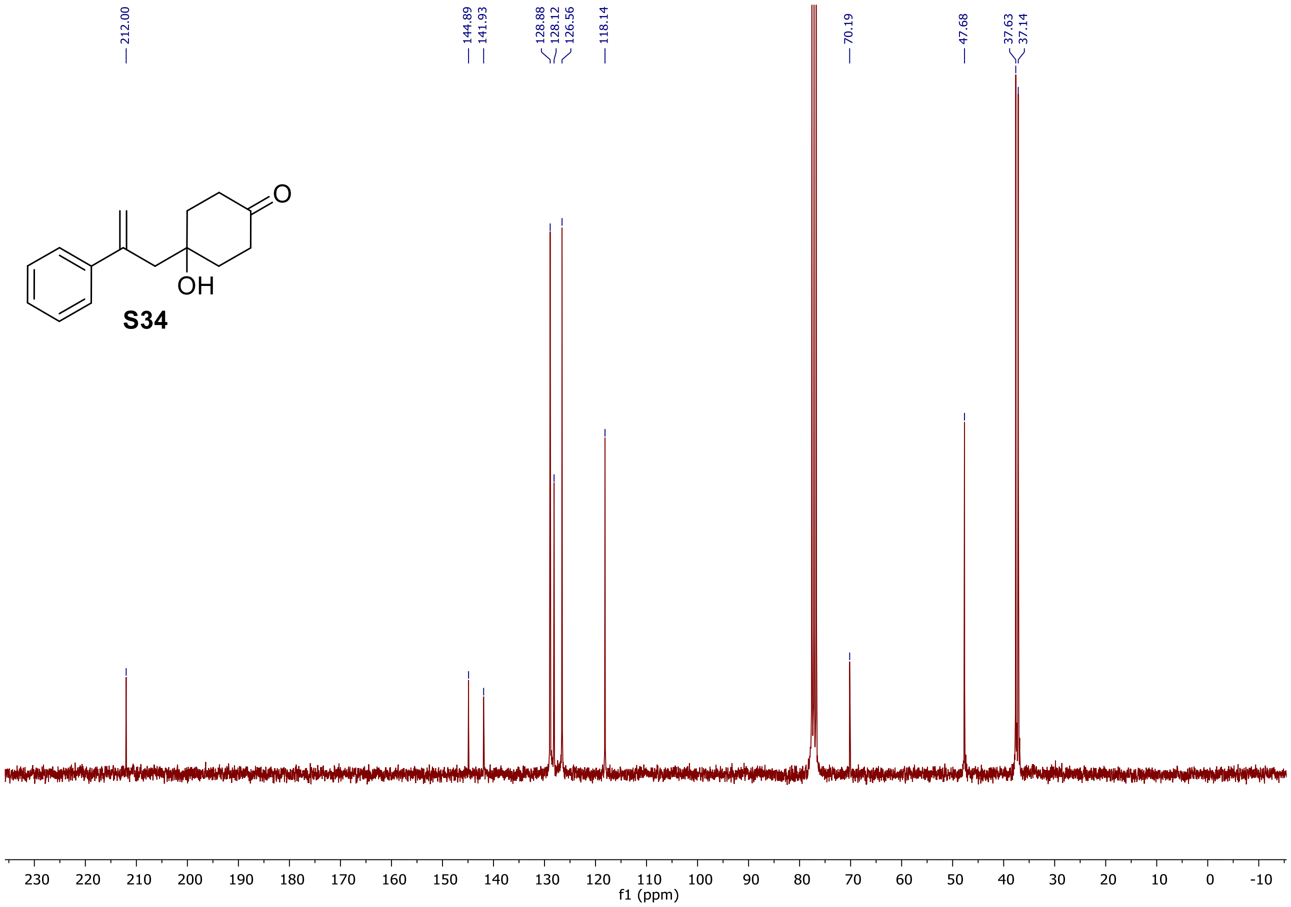
S34
OH
O
f1 (ppm)

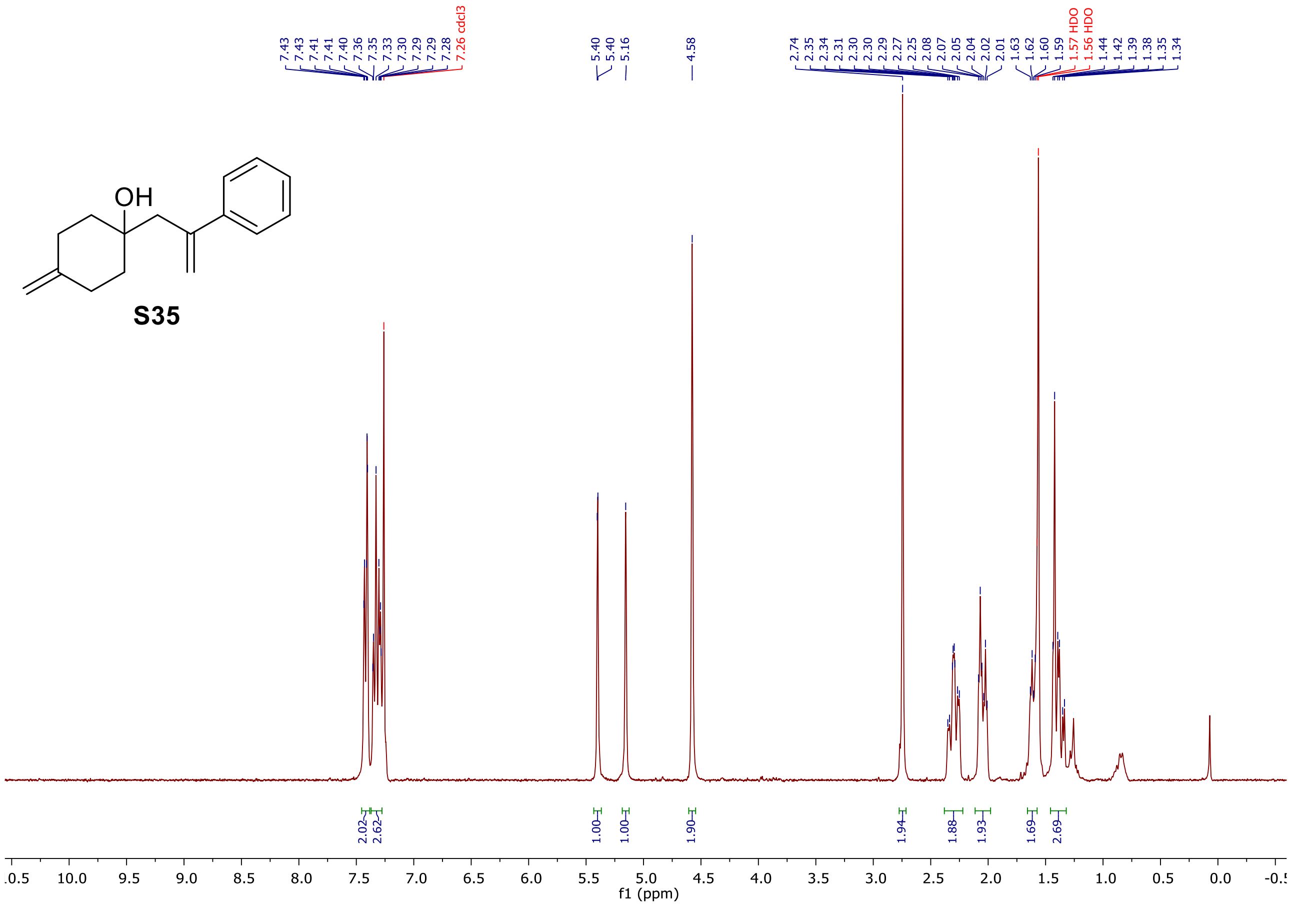
7.43 7.43 7.41 7.41 7.40 7.36 7.35 7.33 7.30 7.29 7.29 7.28 7.26 cdcl3
5.40 5.40 5.16
4.58
2.74 2.35 2.34 2.31 2.30 2.30 2.29 2.27 2.25 2.08 2.07 2.05 2.04 2.02 2.01 1.63 1.62 1.60 1.59 1.57 HDO 1.56 HDO 1.44 1.42 1.39 1.38 1.35 1.34
OH
S35
2.02 2.62 1.00 1.00 1.90 1.94 1.88 1.93 1.69 2.69
f1 (ppm)

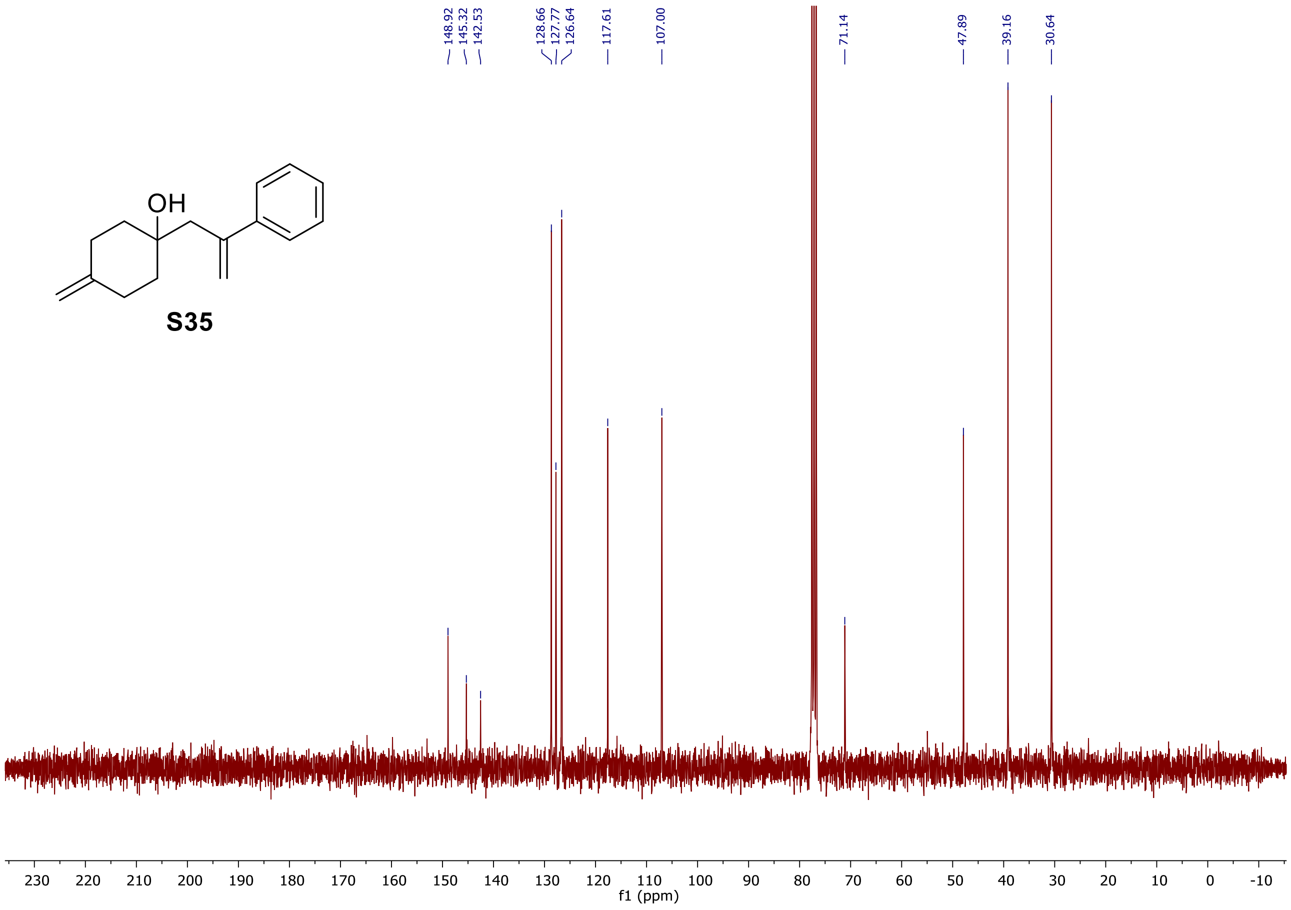
148.92 145.32 142.53
128.66 127.77 126.64
117.61
107.00
71.14
47.89
39.16
30.64
OH
S35
f1 (ppm)

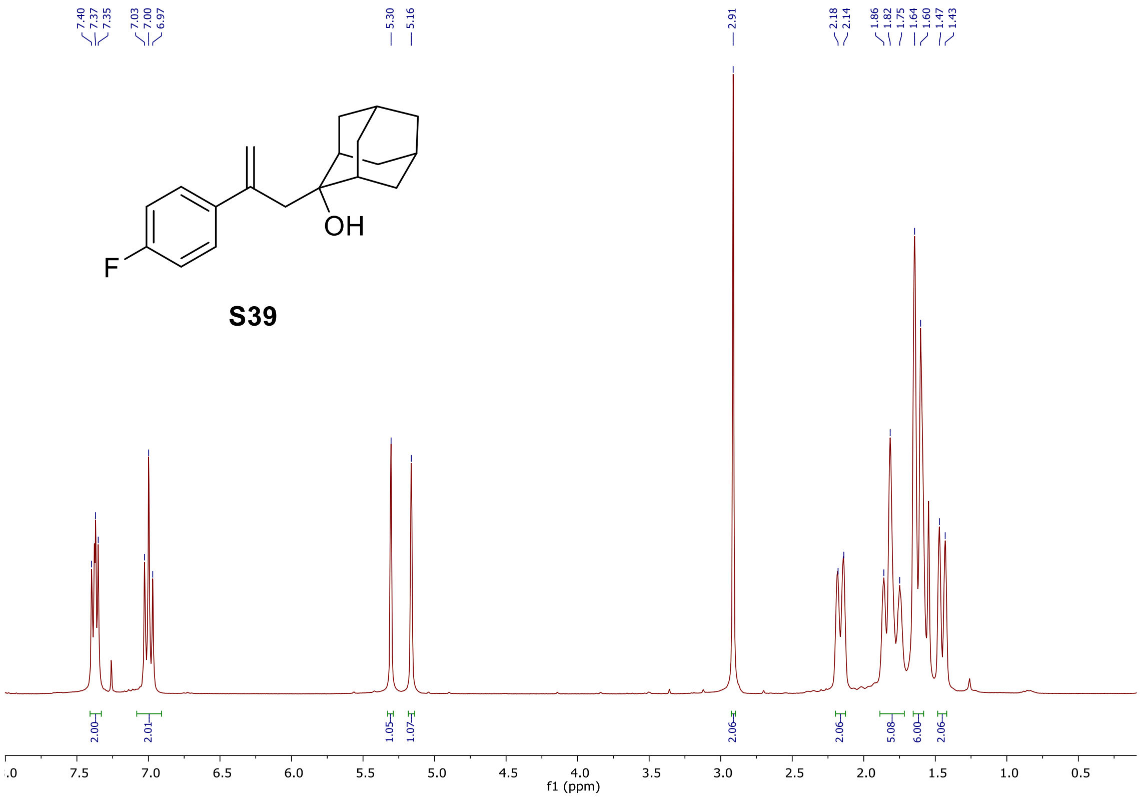

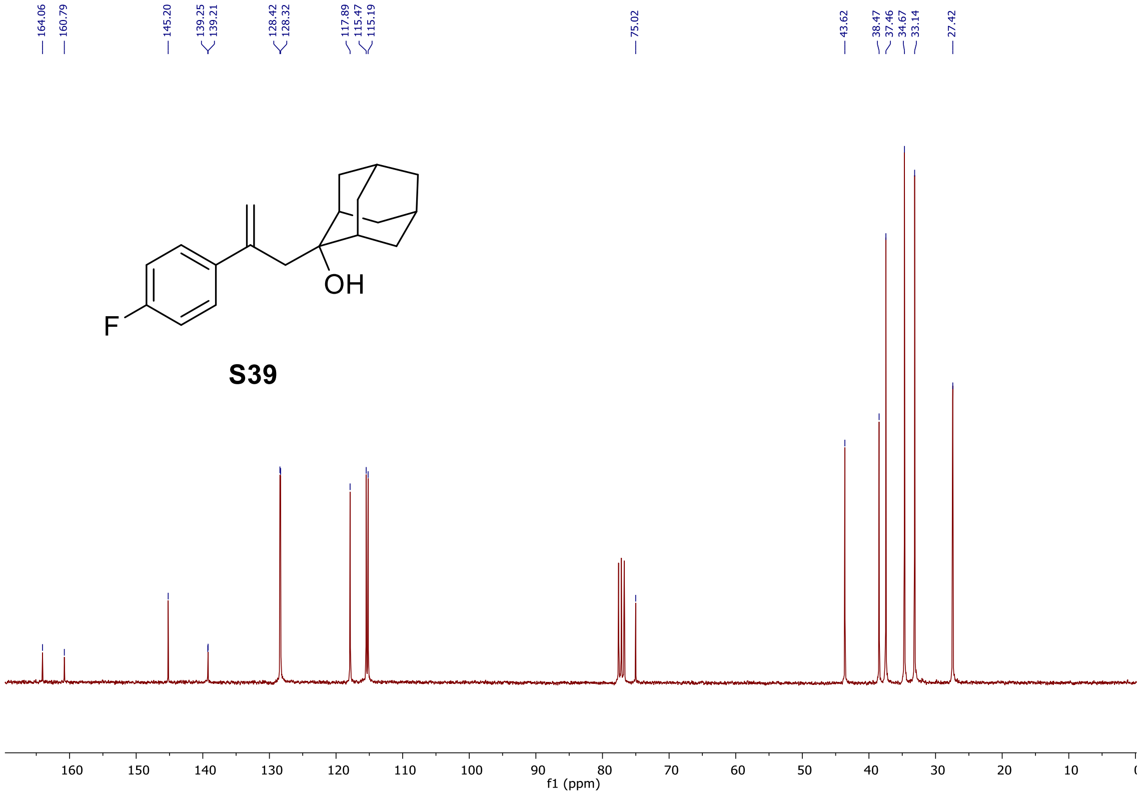

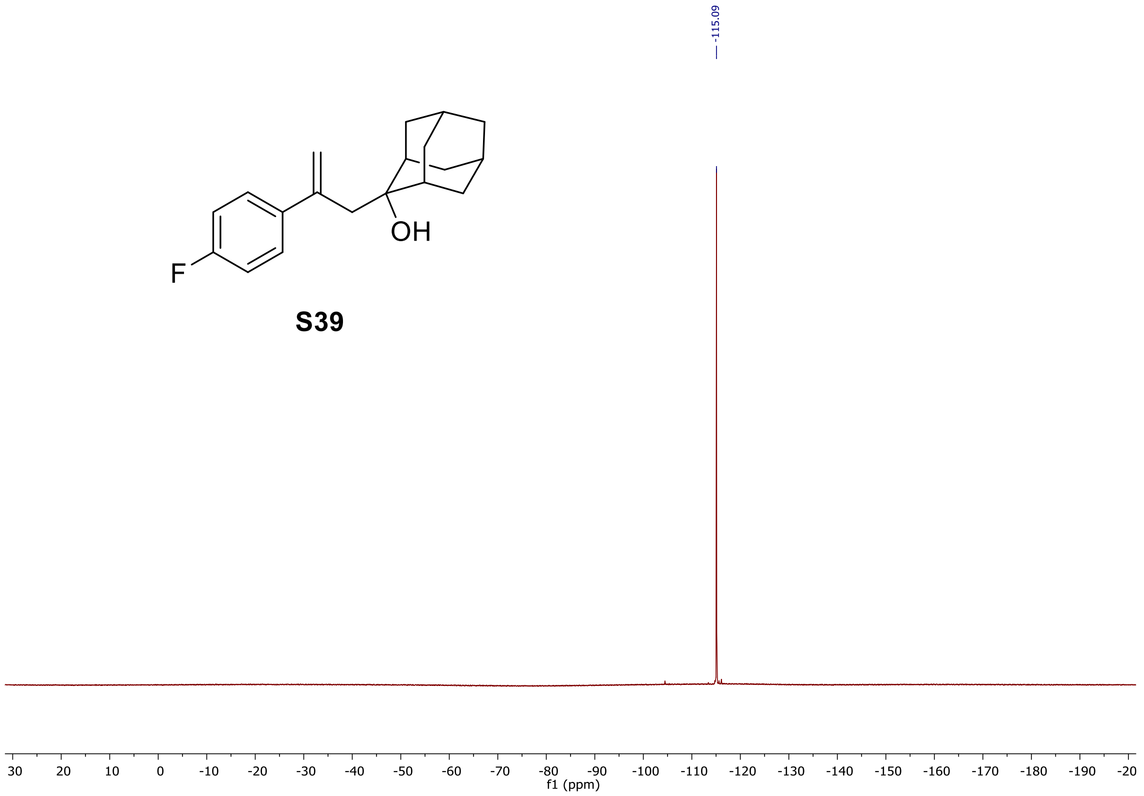

-115.09
F
OH
S39
30 20 10 0 -10 -20 -30 -40 -50 -60 -70 -80 -90 -100 -110 -120 -130 -140 -150 -160 -170 -180 -190 -20
f1 (ppm)

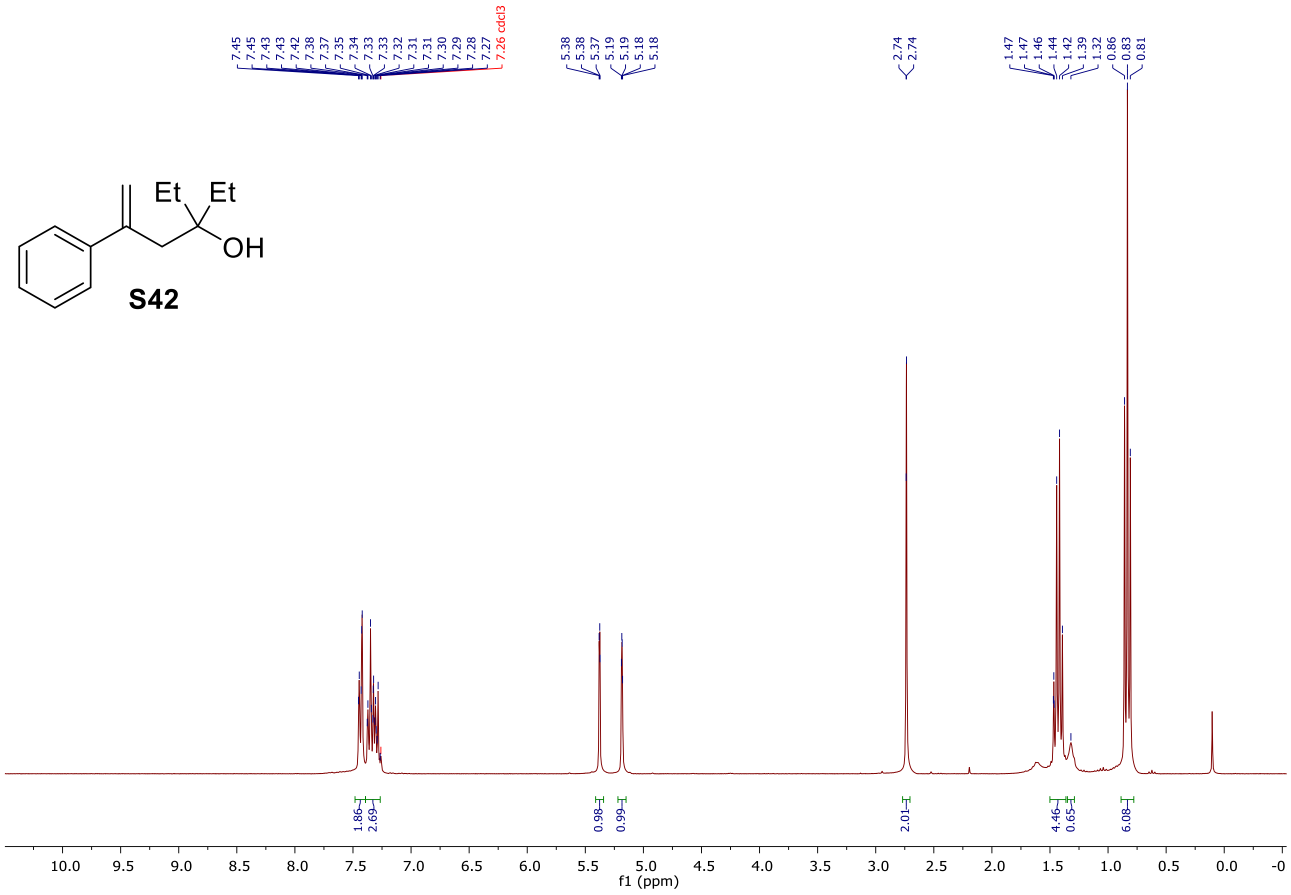

Et
Et
OH
S42
f1 (ppm)

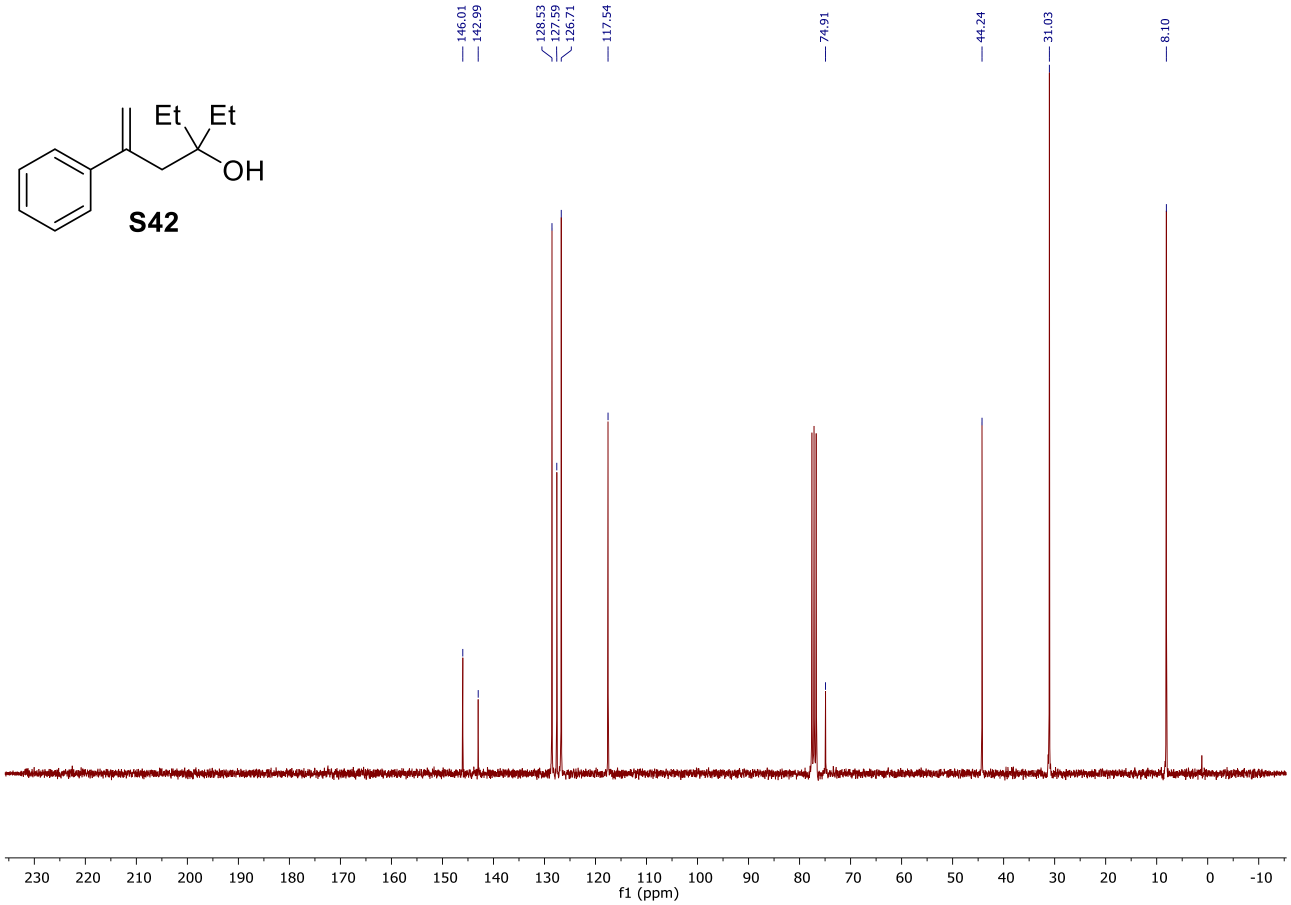

Et
Et
OH
S42
f1 (ppm)

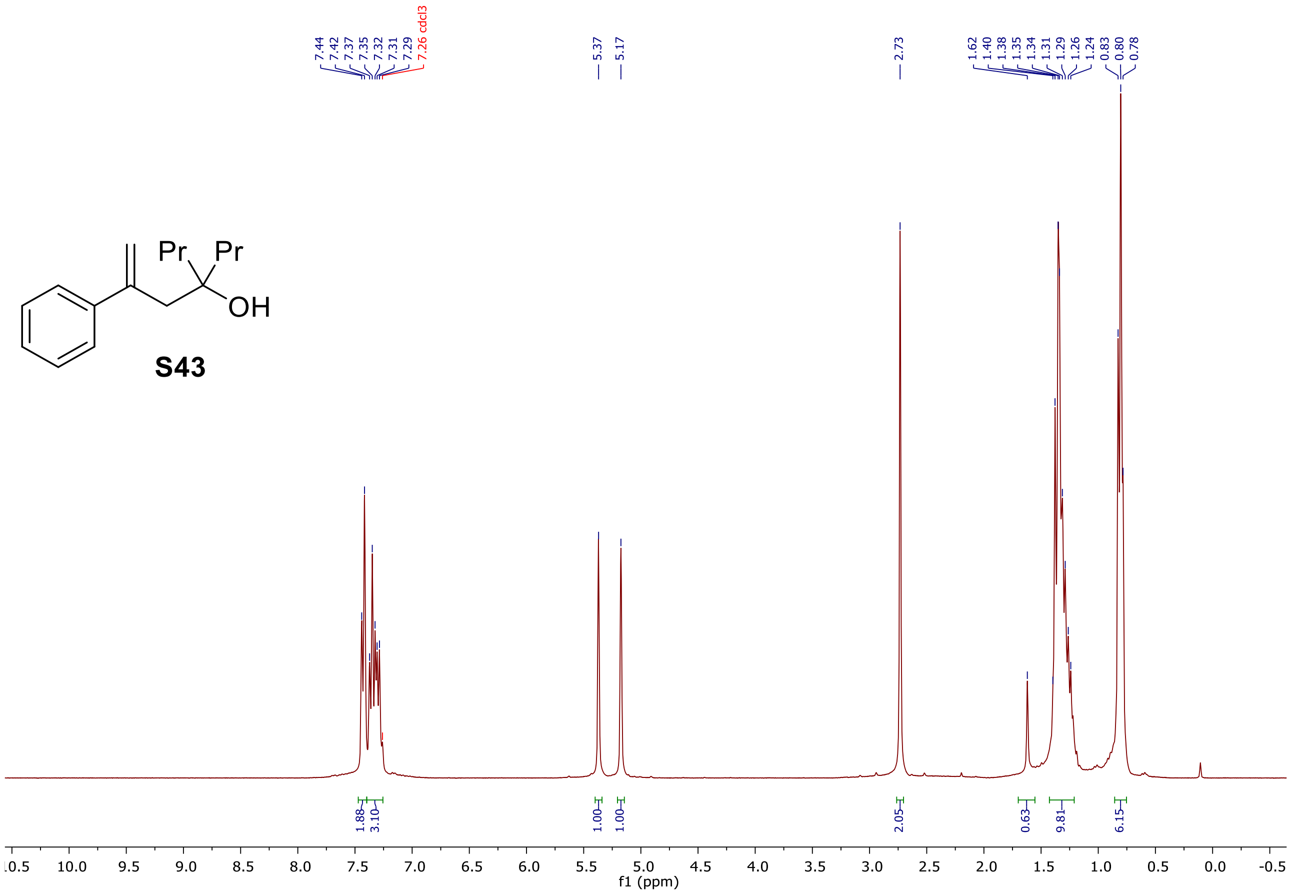
Pr
Pr
OH
S43
7.44
7.42
7.37
7.35
7.32
7.31
7.29
7.26 cdcl3
5.37
5.17
2.73
1.62
1.40
1.38
1.35
1.34
1.31
1.29
1.26
1.24
0.83
0.80
0.78
1.88
3.10
1.00
1.00
2.05
0.63
9.81
6.15
f1 (ppm)

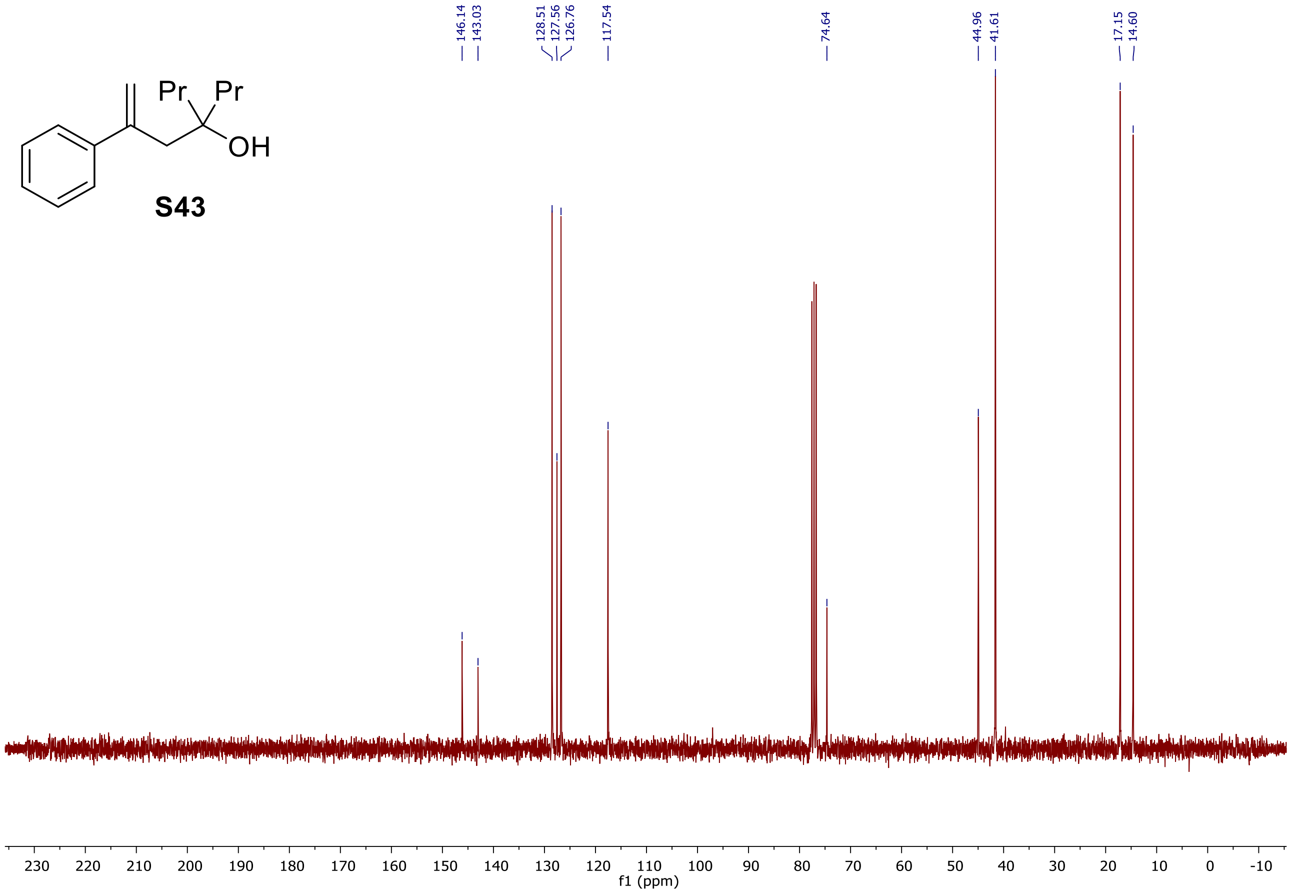
Pr
Pr
OH
S43
146.14
143.03
128.51
127.56
126.76
117.54
74.64
44.96
41.61
17.15
14.60
f1 (ppm)

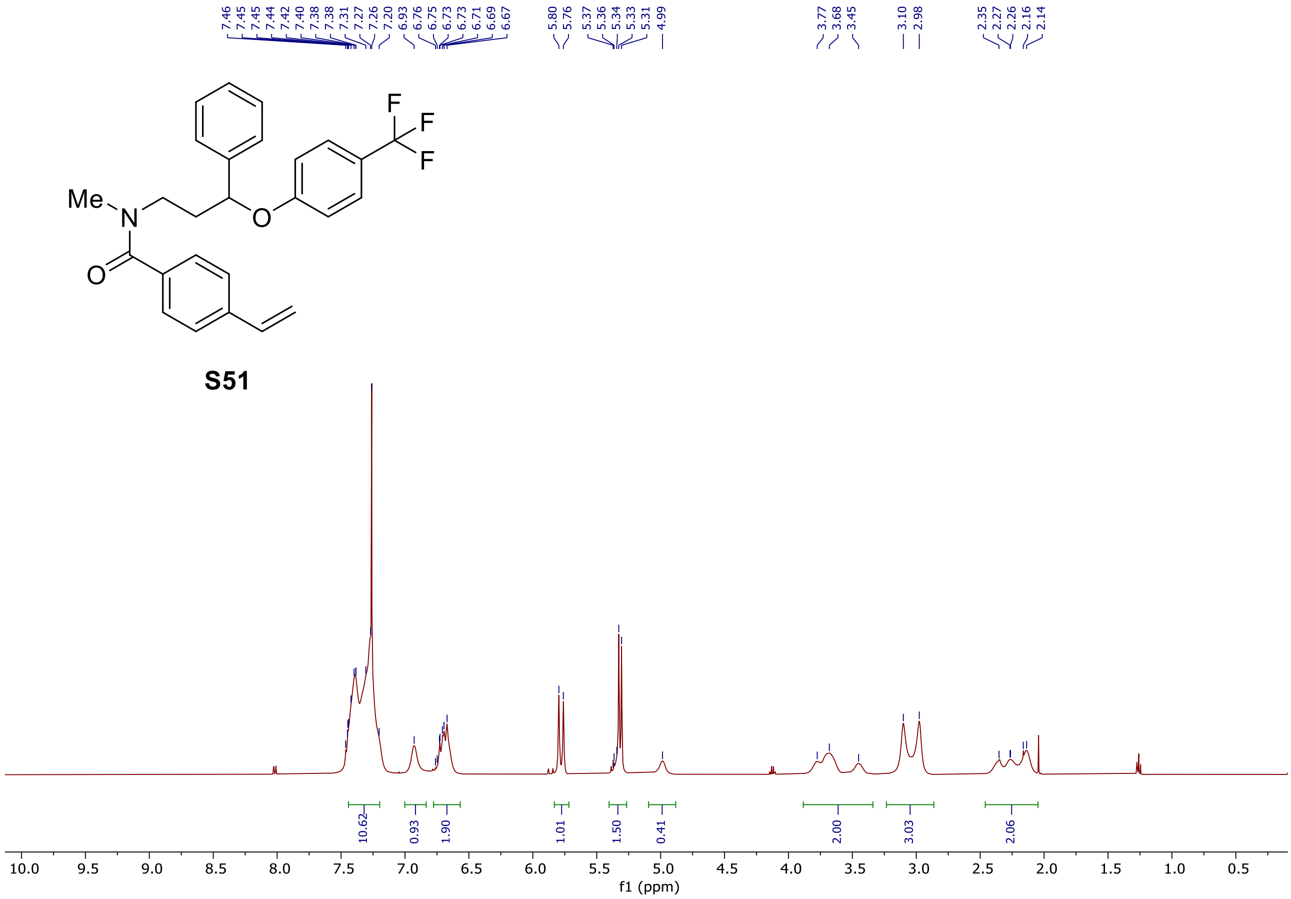
7.46 7.45 7.45 7.44 7.42 7.40 7.38 7.38 7.31 7.27 7.26 7.20 6.93 6.76 6.75 6.73 6.73 6.71 6.69 6.67 5.80 5.76 5.37 5.36 5.34 5.33 5.31 4.99 3.77 3.68 3.45 3.10 2.98 2.35 2.27 2.26 2.16 2.14
Me
N
O
O
F
F
F
S51
10.62 0.93 1.90 1.01 1.50 0.41 2.00 3.03 2.06
f1 (ppm)

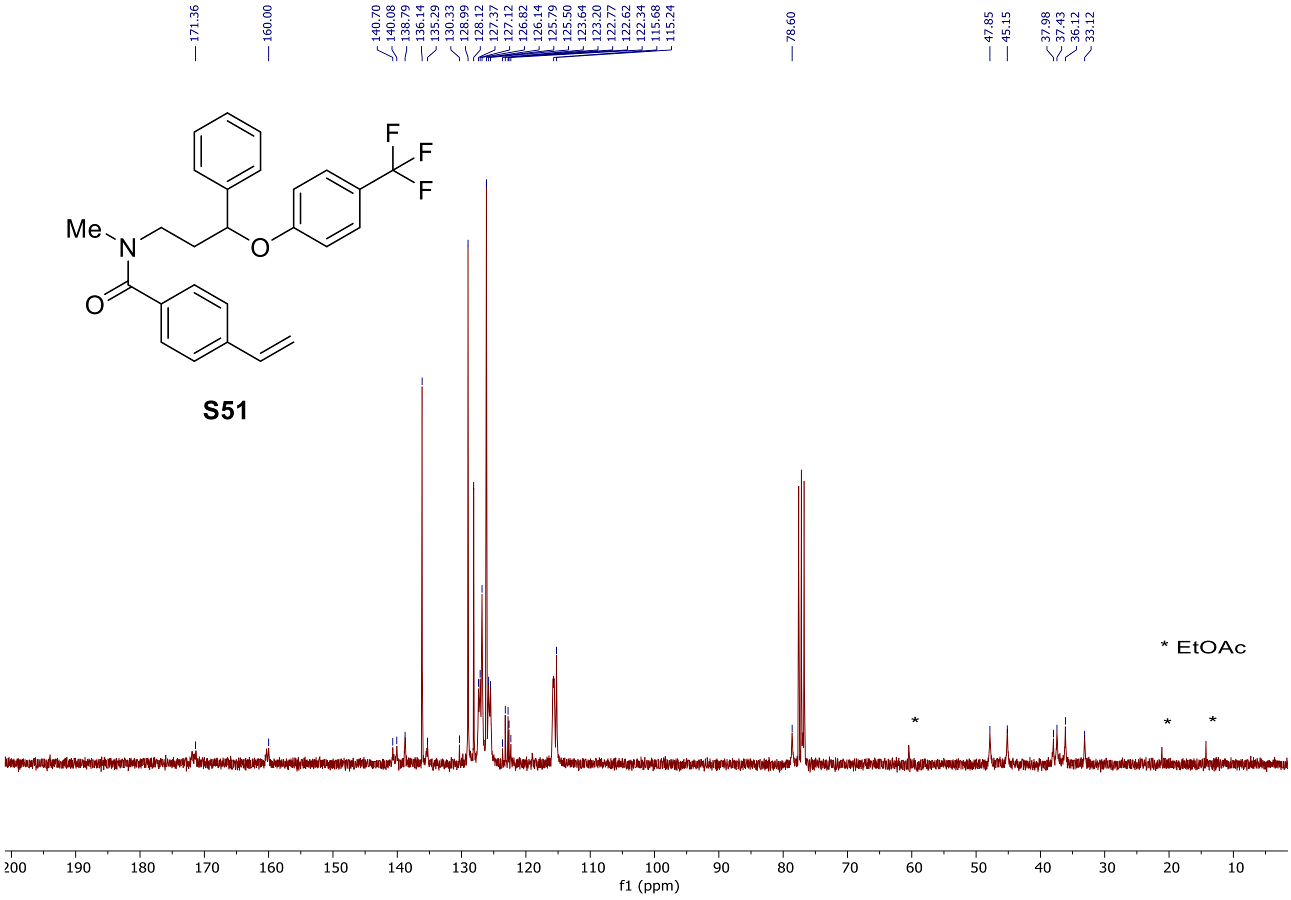
171.36 160.00 140.70 140.08 138.79 136.14 135.29 130.33 128.99 128.12 127.37 127.12 126.82 126.14 125.79 125.50 123.64 123.20 122.77 122.62 122.34 115.68 115.24 78.60 47.85 45.15 37.98 37.43 36.12 33.12
Me
N
O
O
F
F
F
S51
* EtOAc
f1 (ppm)

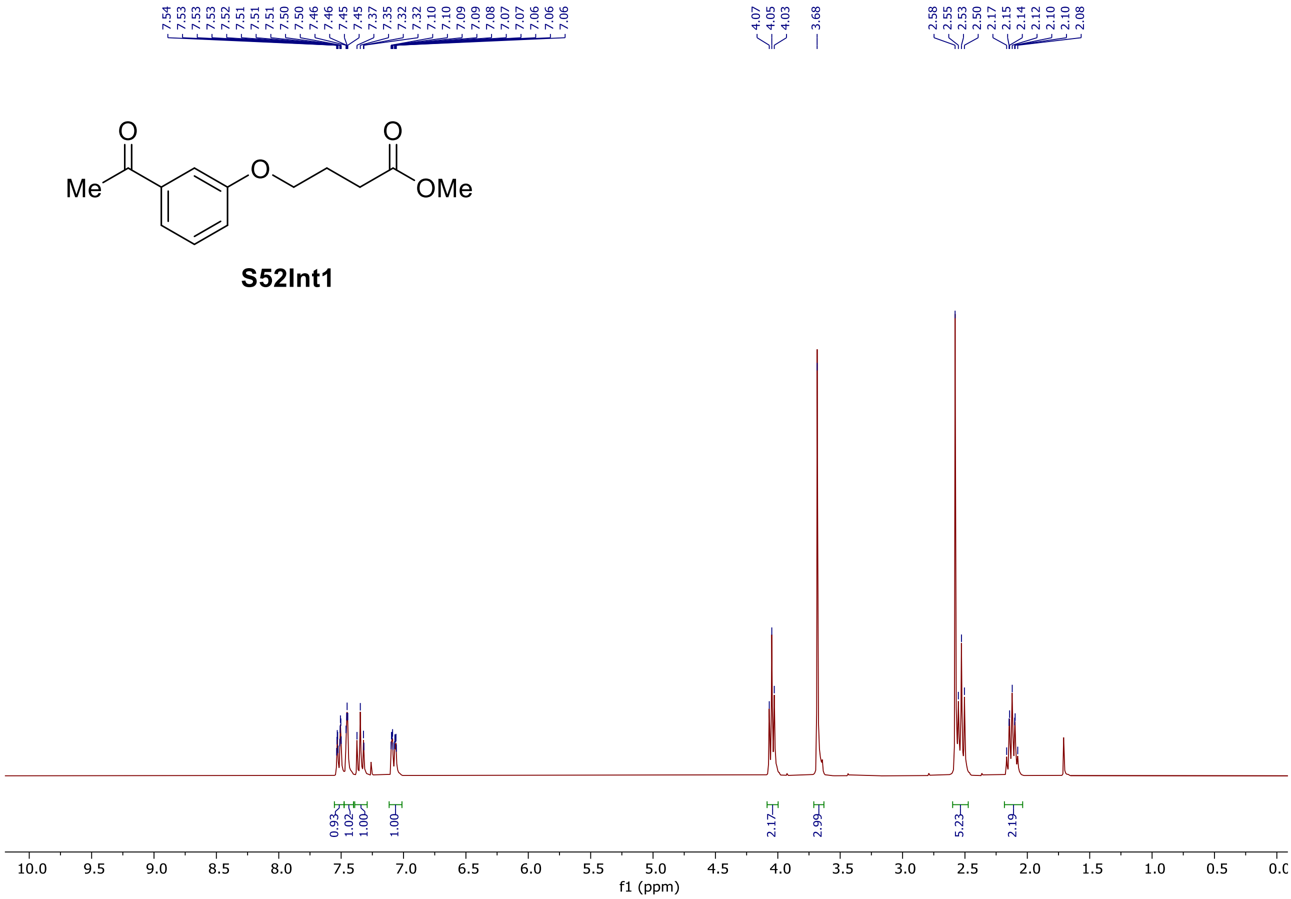
S52Int1
f1 (ppm)

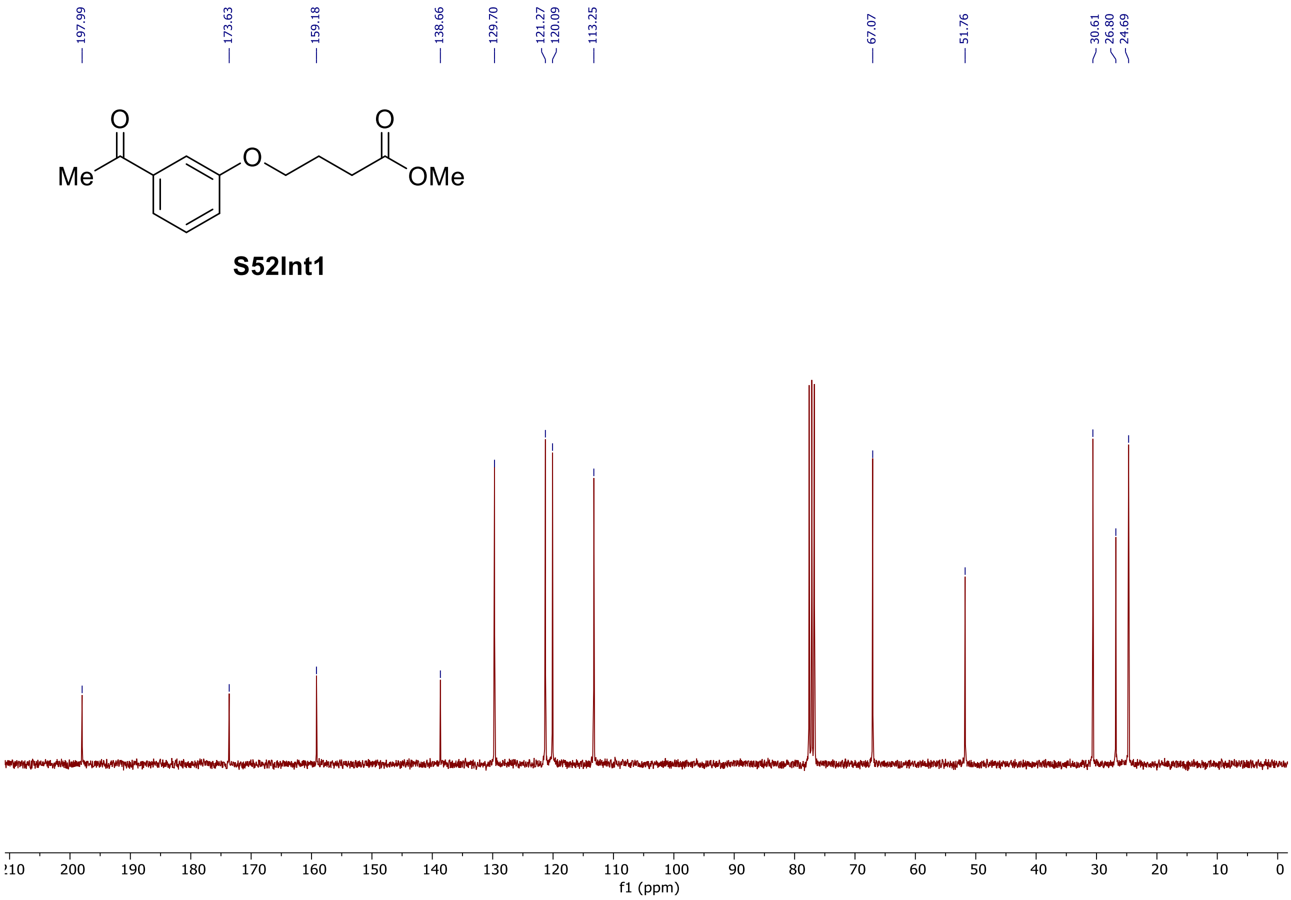
S52Int1
f1 (ppm)

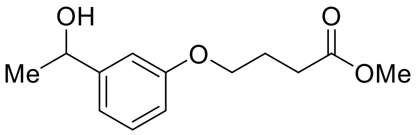

S52Int2

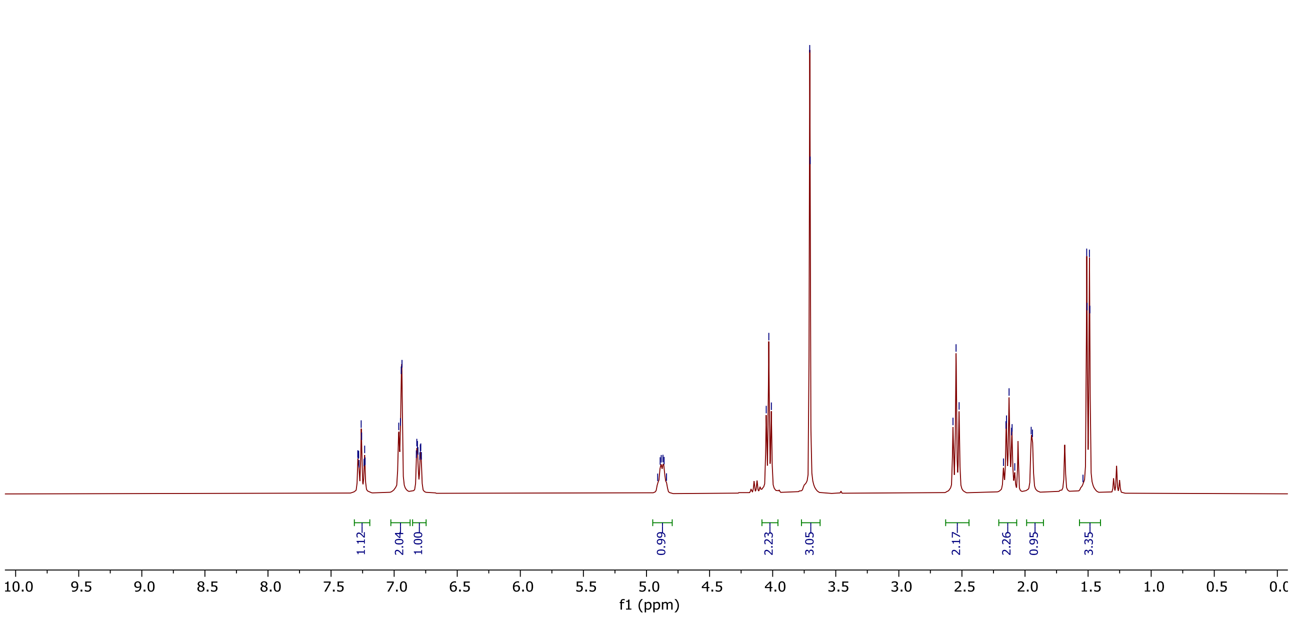

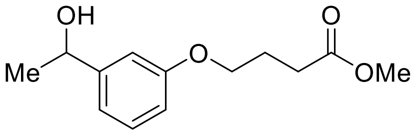

S52Int2

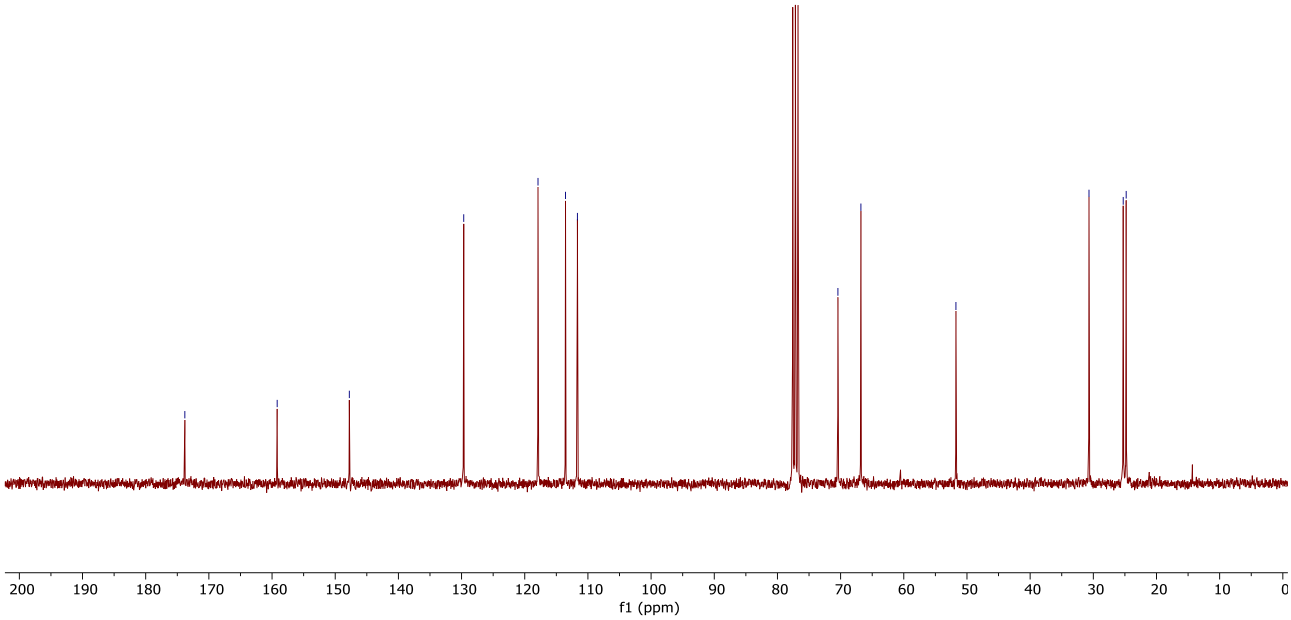

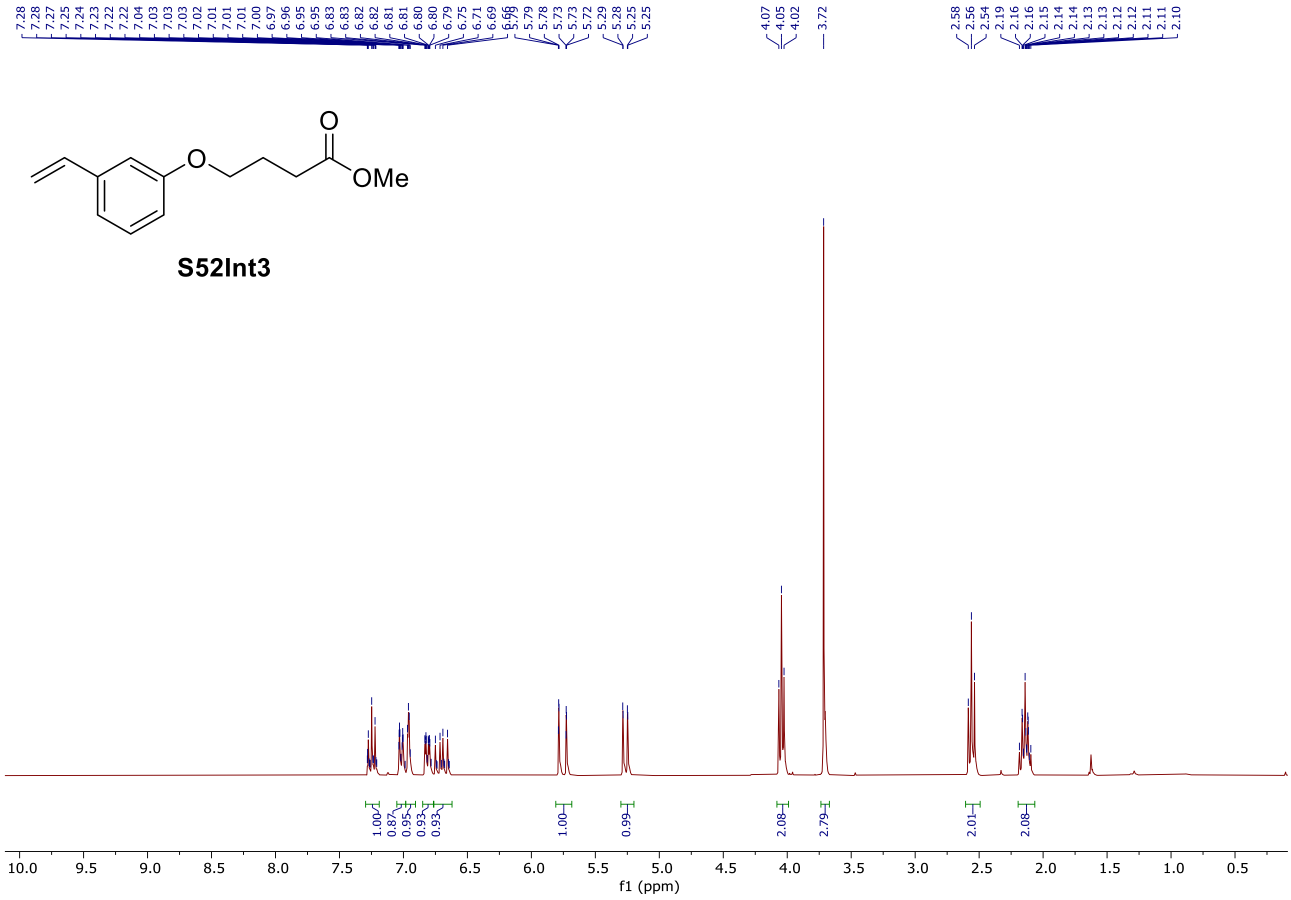
OMe
S52Int3
f1 (ppm)

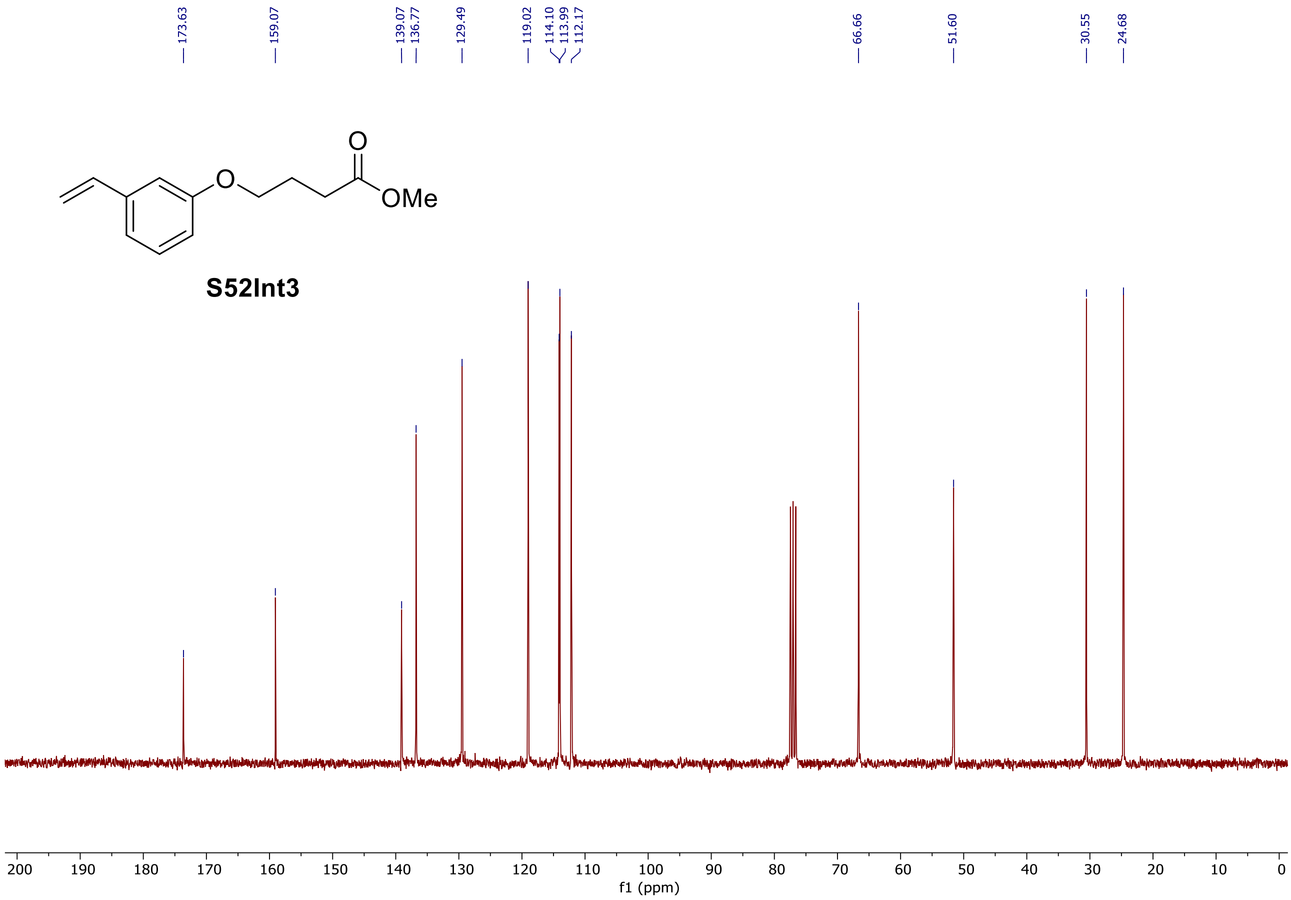
OMe
S52Int3
f1 (ppm)

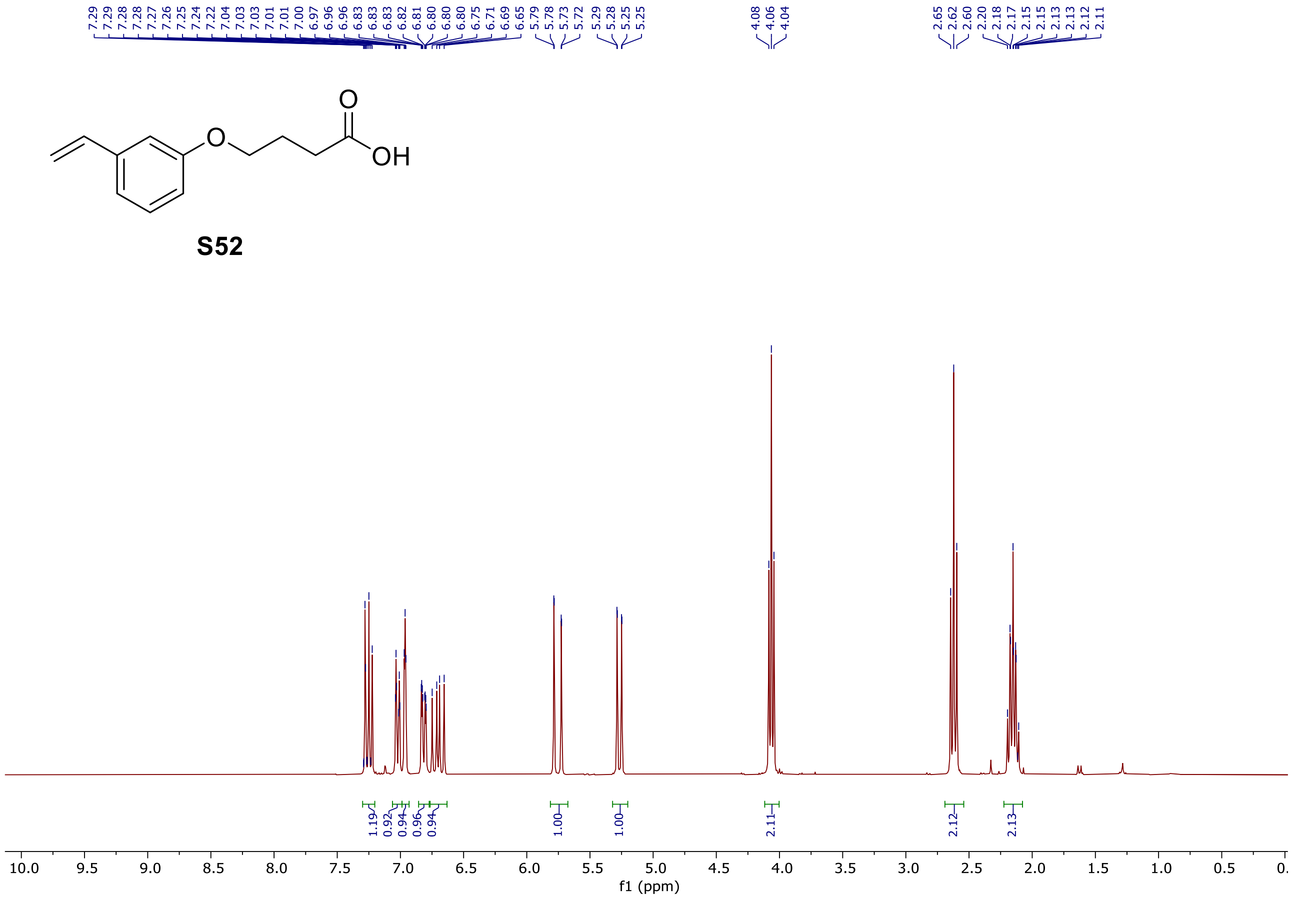

S52

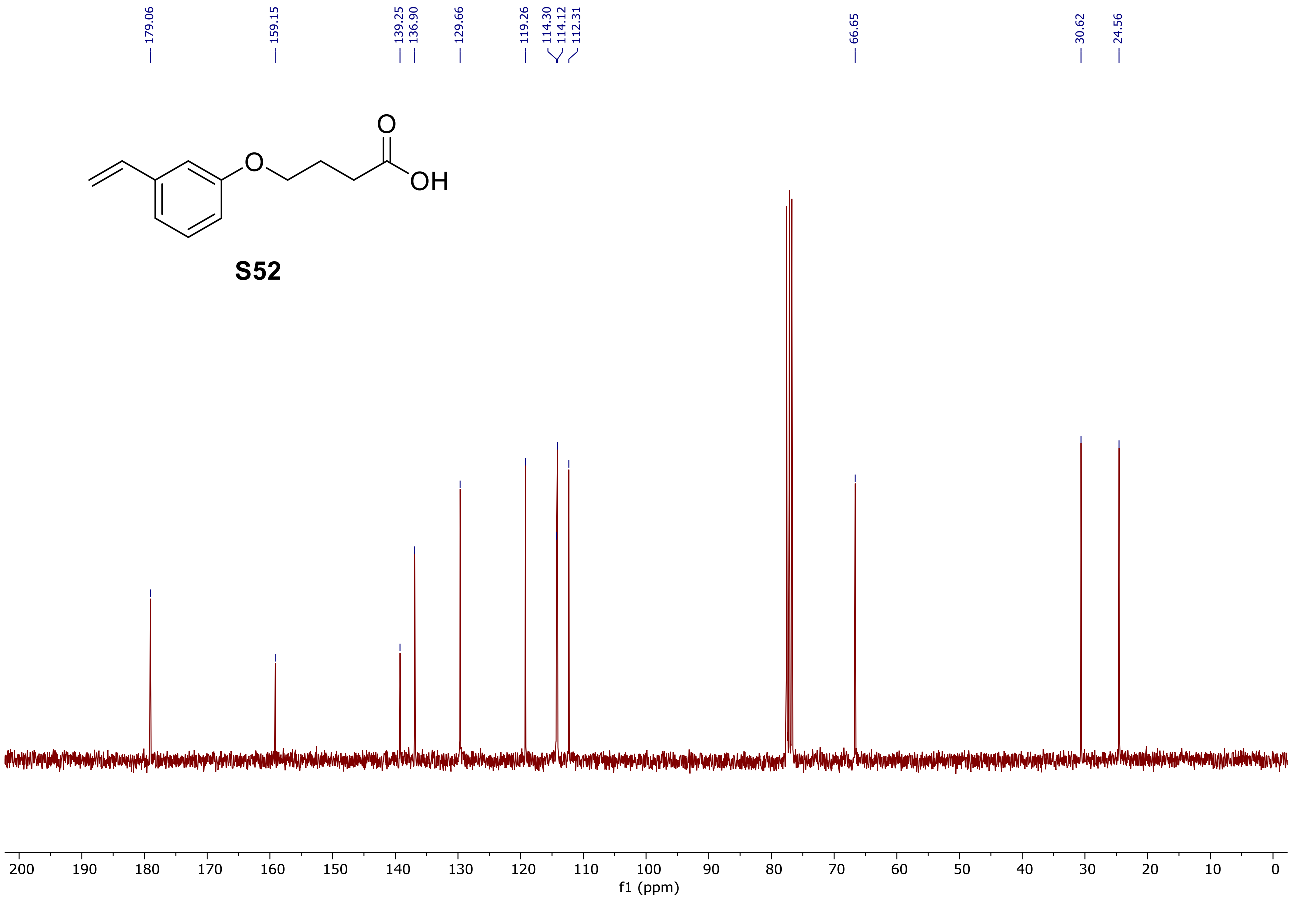

S52

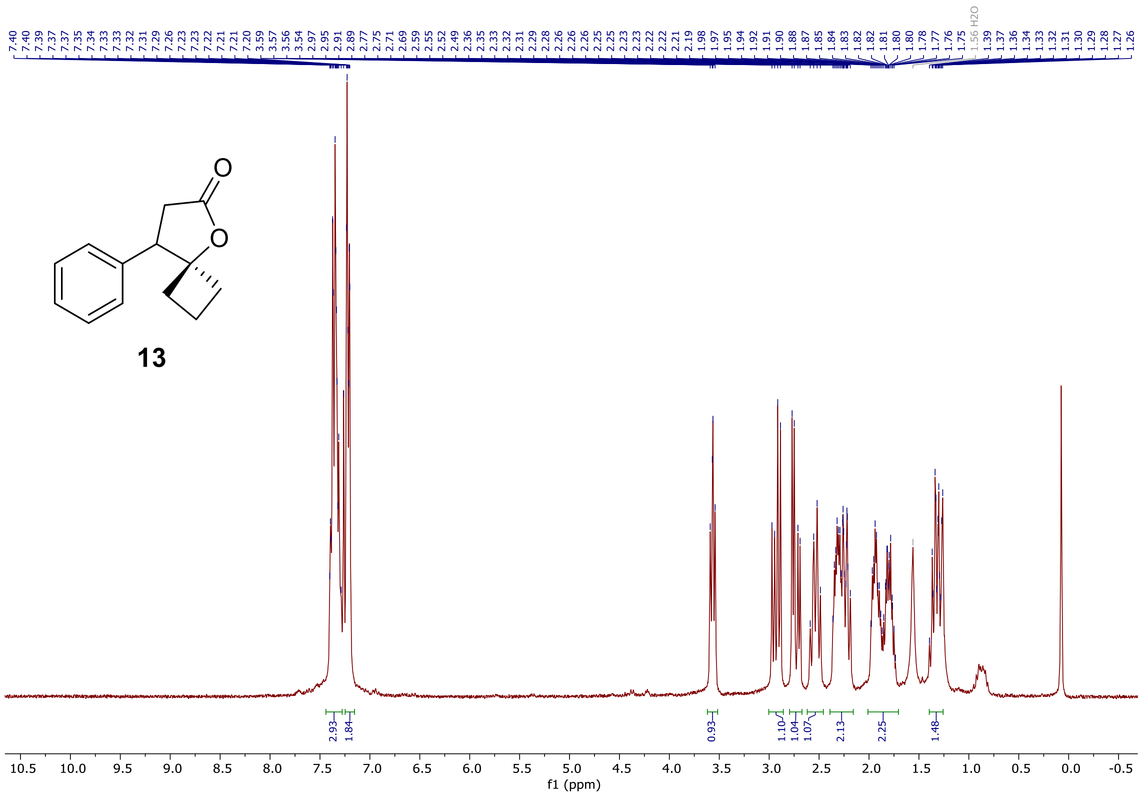
7.40 7.40 7.39 7.37 7.37 7.35 7.34 7.33 7.33 7.32 7.31 7.29 7.26 7.23 7.23 7.22 7.21 7.21 7.20 3.59 3.57 3.56 3.54 2.97 2.95 2.91 2.89 2.77 2.75 2.71 2.69 2.59 2.55 2.52 2.49 2.36 2.35 2.33 2.32 2.31 2.29 2.28 2.26 2.26 2.25 2.25 2.23 2.23 2.22 2.21 2.19 1.98 1.97 1.95 1.94 1.92 1.91 1.90 1.88 1.87 1.85 1.84 1.83 1.82 1.82 1.81 1.80 1.80 1.78 1.77 1.76 1.75 1.56 H2O 1.39 1.37 1.36 1.34 1.33 1.32 1.31 1.30 1.29 1.28 1.27 1.26
O
O
13
2.93
1.84
0.93
1.10
1.04
1.07
2.13
2.25
1.48
f1 (ppm)

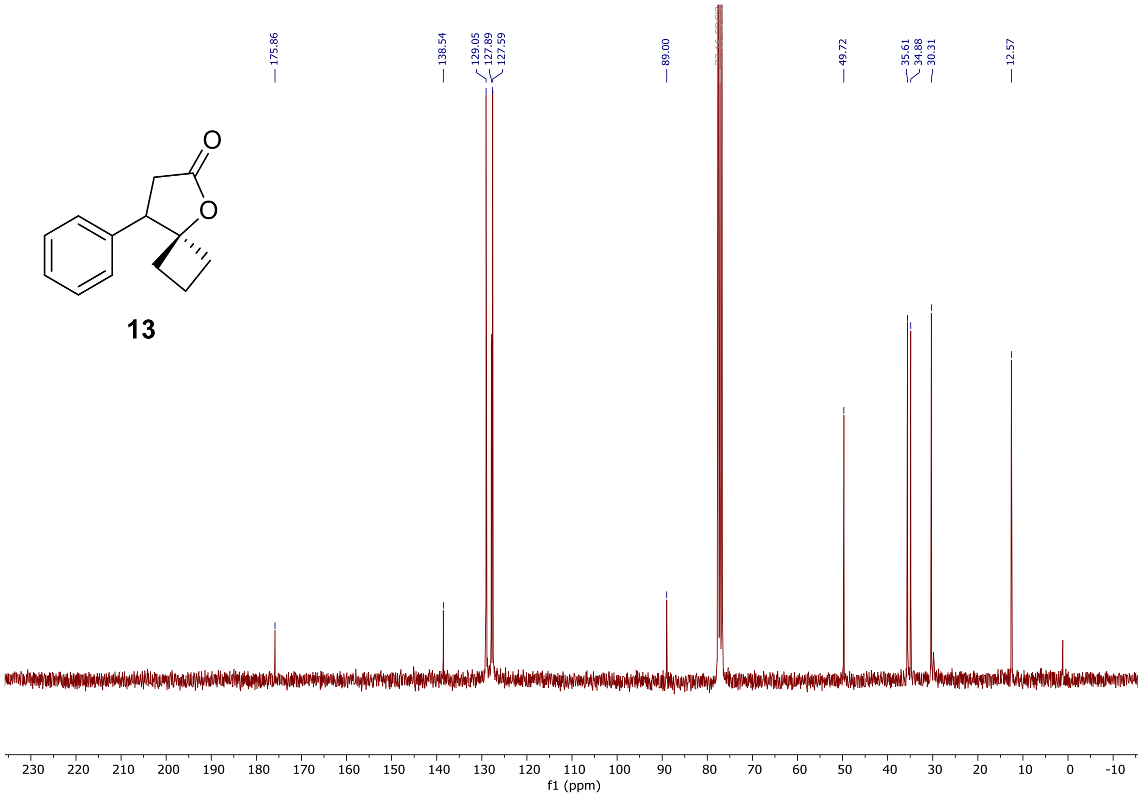
175.86
138.54
129.05
127.89
127.59
89.00
49.72
35.61
34.88
30.31
12.57
O
O
13
f1 (ppm)

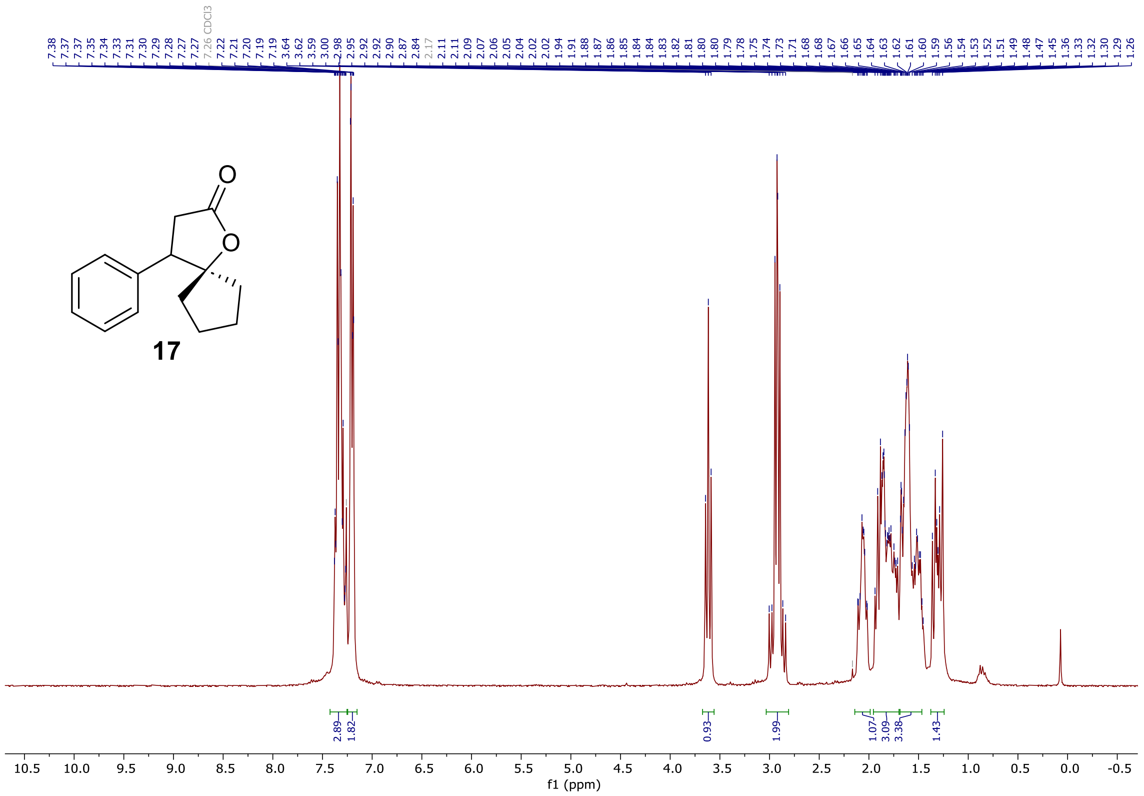
17
7.26 CDCl3
f1 (ppm)

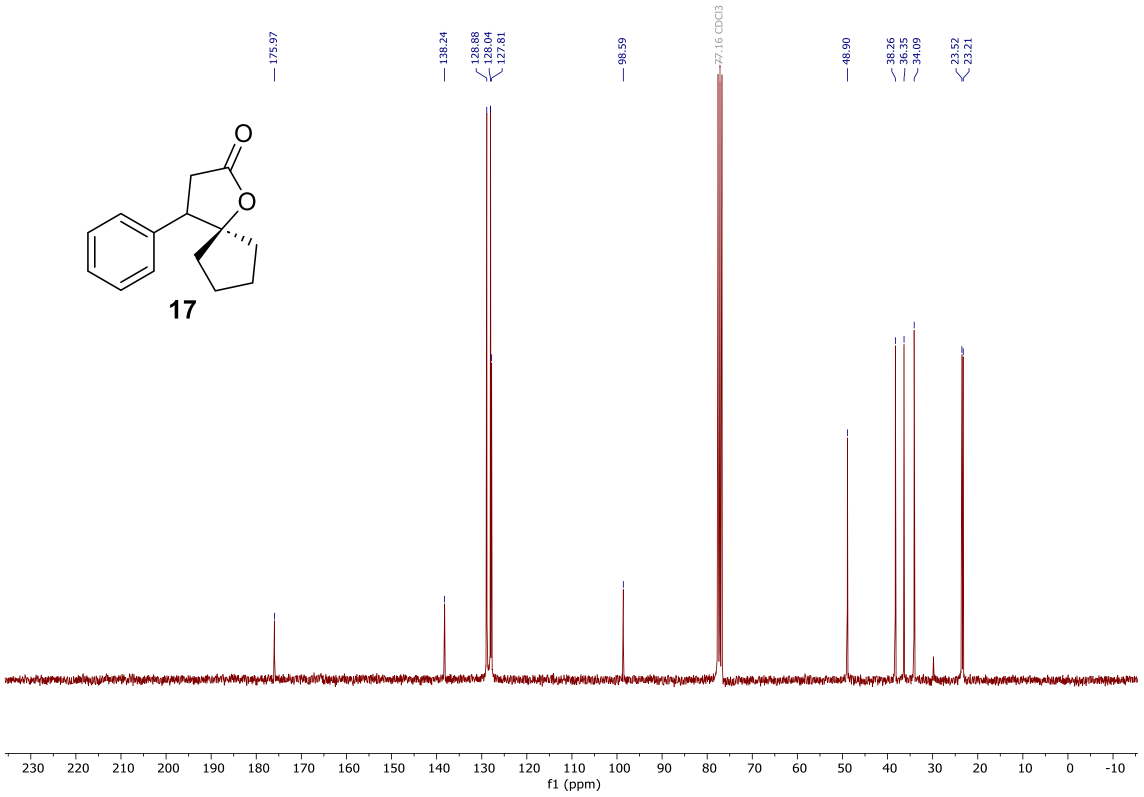
175.97
138.24
128.88
128.04
127.81
98.59
77.16 CDCl3
48.90
38.26
36.35
34.09
23.52
23.21
17
f1 (ppm)

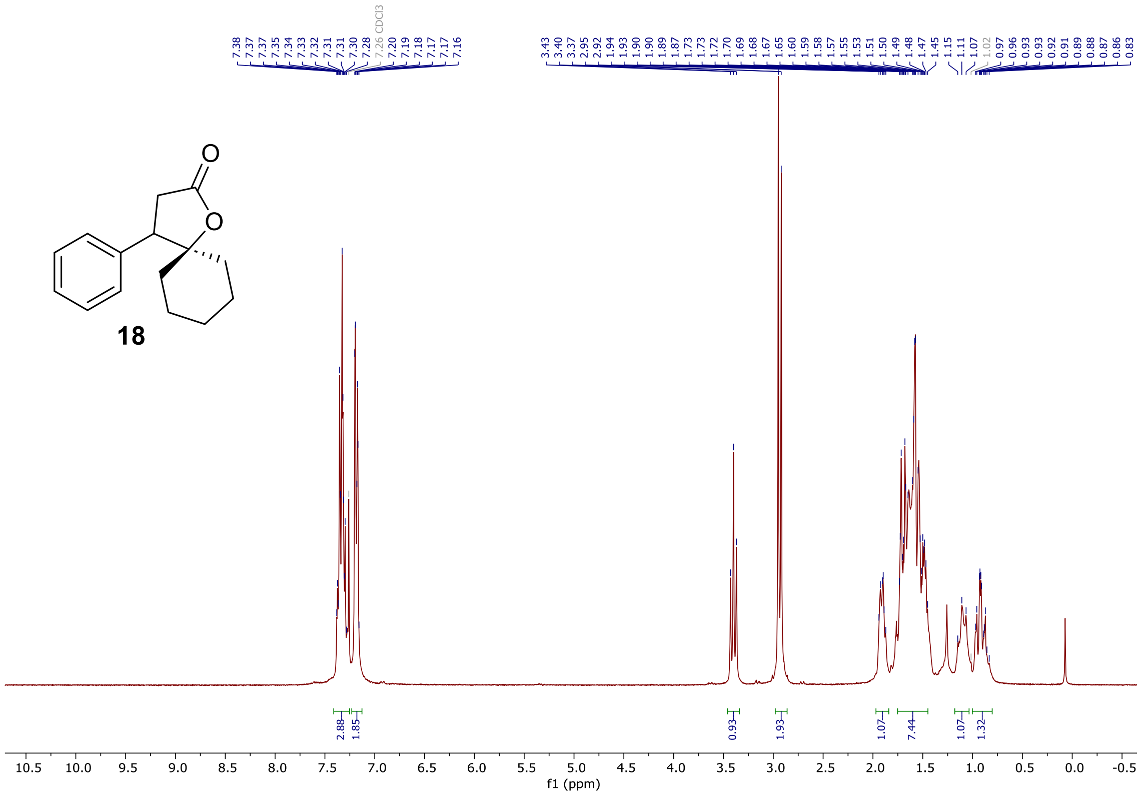
7.38 7.37 7.37 7.35 7.34 7.33 7.32 7.31 7.31 7.30 7.28 7.26 CDCl3 7.20 7.19 7.18 7.17 7.17 7.16
3.43 3.40 3.37 2.95 2.92 1.94 1.93 1.90 1.90 1.89 1.87 1.73 1.73 1.72 1.70 1.69 1.68 1.67 1.65 1.60 1.59 1.58 1.57 1.55 1.55 1.53 1.51 1.50 1.49 1.48 1.47 1.45 1.15 1.11 1.07 1.02 0.97 0.96 0.93 0.93 0.92 0.91 0.89 0.88 0.87 0.86 0.83
O
O
18
2.88 1.85 0.93 1.93 1.07 7.44 1.07 1.32
f1 (ppm)

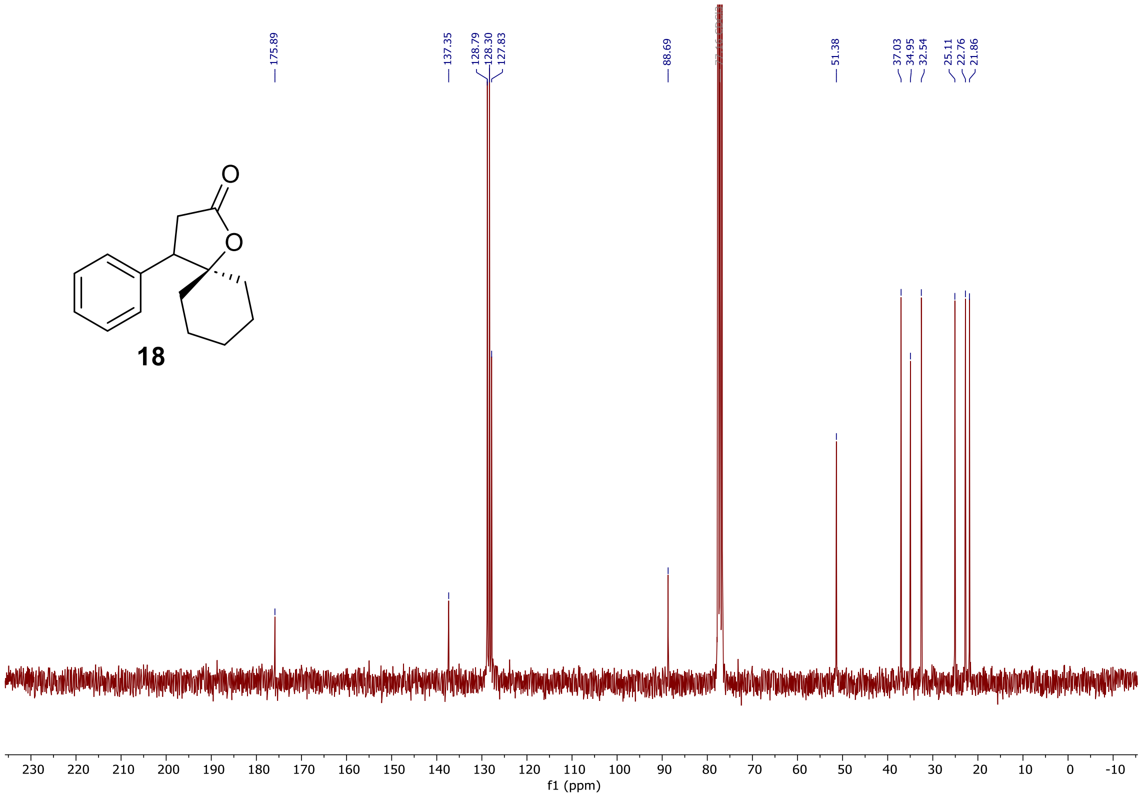
175.89
137.35 128.79 128.30 127.83
88.69
51.38
37.03 34.95 32.54 25.11 22.76 21.86
O
O
18
f1 (ppm)

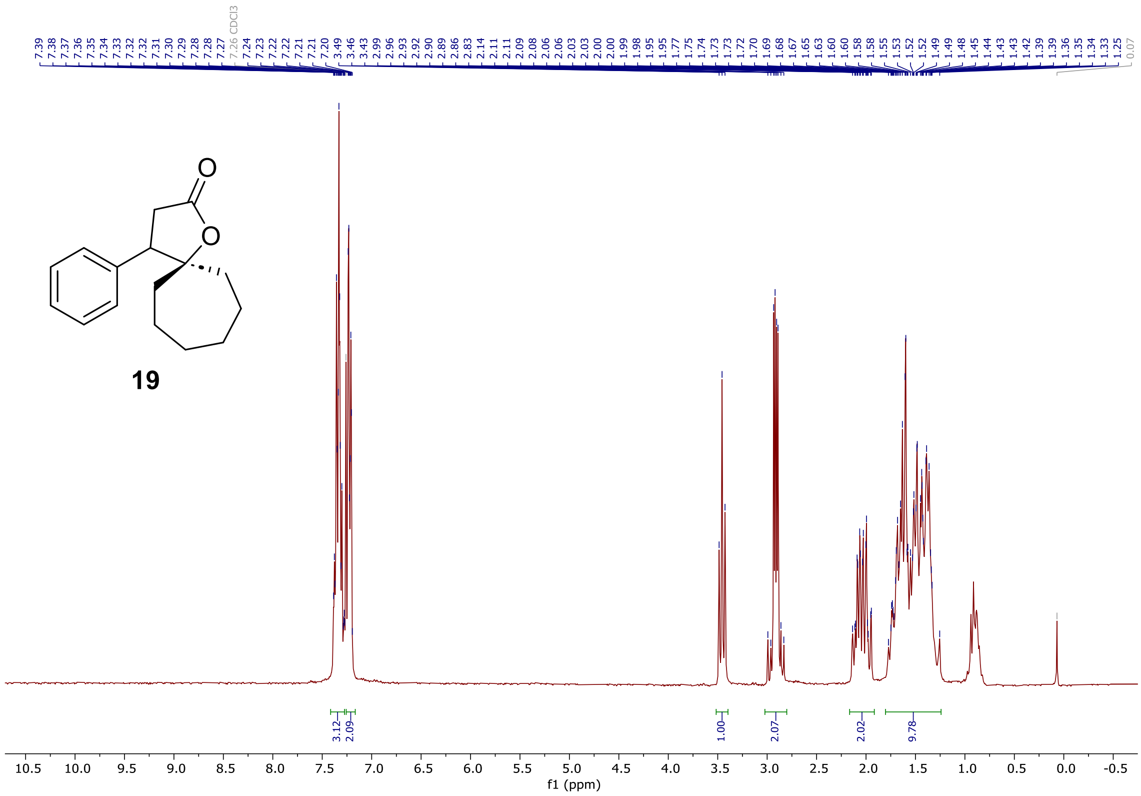
7.39 7.38 7.37 7.36 7.35 7.34 7.33 7.32 7.32 7.31 7.30 7.29 7.28 7.28 7.27 7.26 CDCl3 7.24 7.23 7.22 7.22 7.21 7.21 7.20 3.49 3.46 3.43 2.99 2.96 2.93 2.92 2.90 2.89 2.86 2.83 2.14 2.11 2.11 2.09 2.08 2.06 2.06 2.03 2.03 2.00 2.00 1.99 1.98 1.95 1.95 1.77 1.75 1.74 1.73 1.73 1.72 1.70 1.69 1.68 1.67 1.65 1.63 1.60 1.60 1.58 1.58 1.55 1.53 1.52 1.52 1.49 1.49 1.48 1.45 1.44 1.43 1.42 1.39 1.39 1.36 1.35 1.34 1.33 1.25 0.07
O
O
19
3.12
2.09
1.00
2.07
2.02
9.78
10.5 10.0 9.5 9.0 8.5 8.0 7.5 7.0 6.5 6.0 5.5 5.0 4.5 4.0 3.5 3.0 2.5 2.0 1.5 1.0 0.5 0.0 -0.5
f1 (ppm)

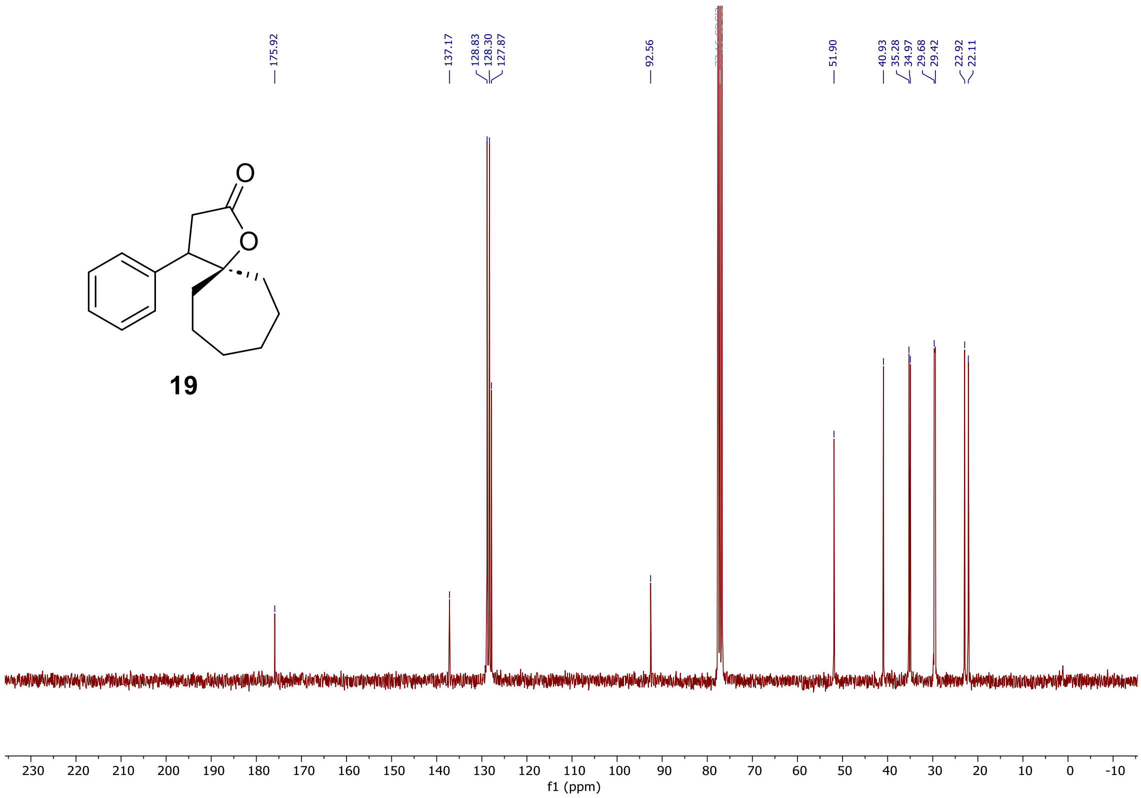
175.92
137.17
128.83
128.30
127.87
92.56
51.90
40.93
35.28
34.97
29.68
29.42
22.92
22.11
O
O
19
230 220 210 200 190 180 170 160 150 140 130 120 110 100 90 80 70 60 50 40 30 20 10 0 -10
f1 (ppm)

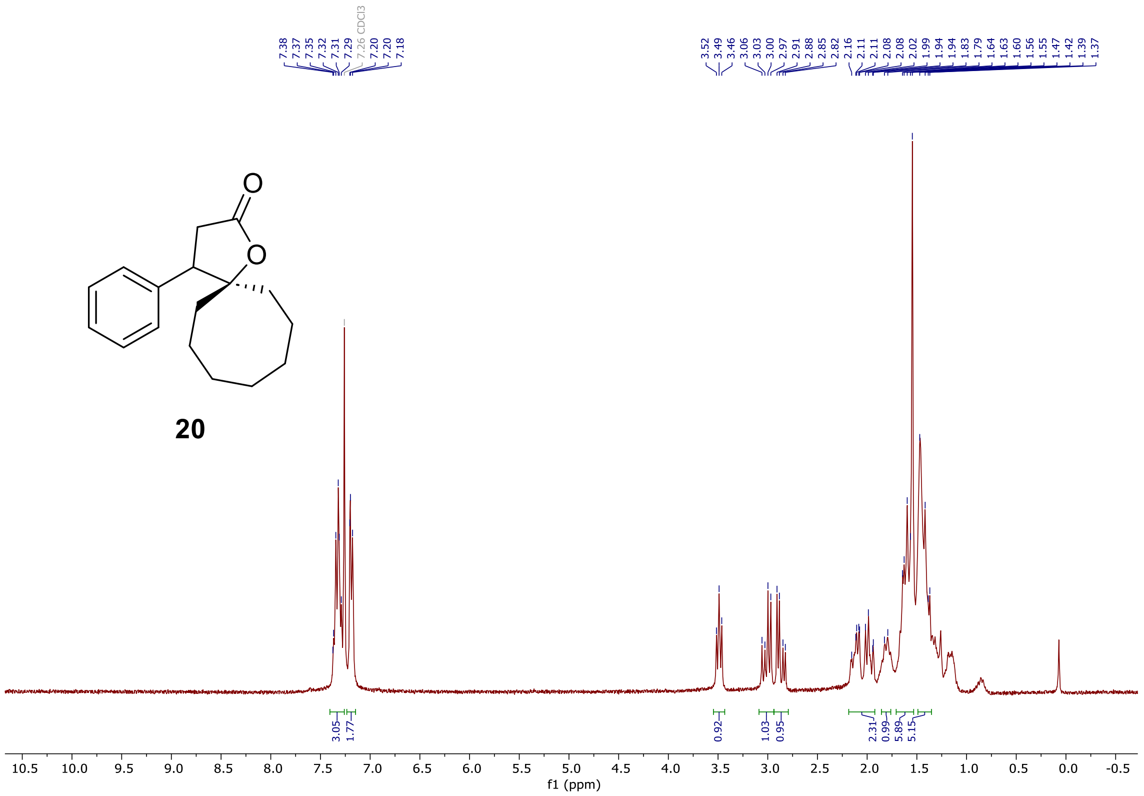
7.38
7.37
7.35
7.32
7.31
7.29
7.26 CDCl3
7.20
7.20
7.18
3.52
3.49
3.46
3.06
3.03
3.00
2.97
2.91
2.88
2.85
2.82
2.16
2.11
2.11
2.08
2.08
2.02
1.99
1.94
1.94
1.83
1.79
1.64
1.63
1.60
1.56
1.55
1.47
1.42
1.39
1.37
O
O
20
3.05
1.77
0.92
1.03
0.95
2.31
0.99
5.89
5.15
10.5 10.0 9.5 9.0 8.5 8.0 7.5 7.0 6.5 6.0 5.5 5.0 4.5 4.0 3.5 3.0 2.5 2.0 1.5 1.0 0.5 0.0 -0.5
f1 (ppm)

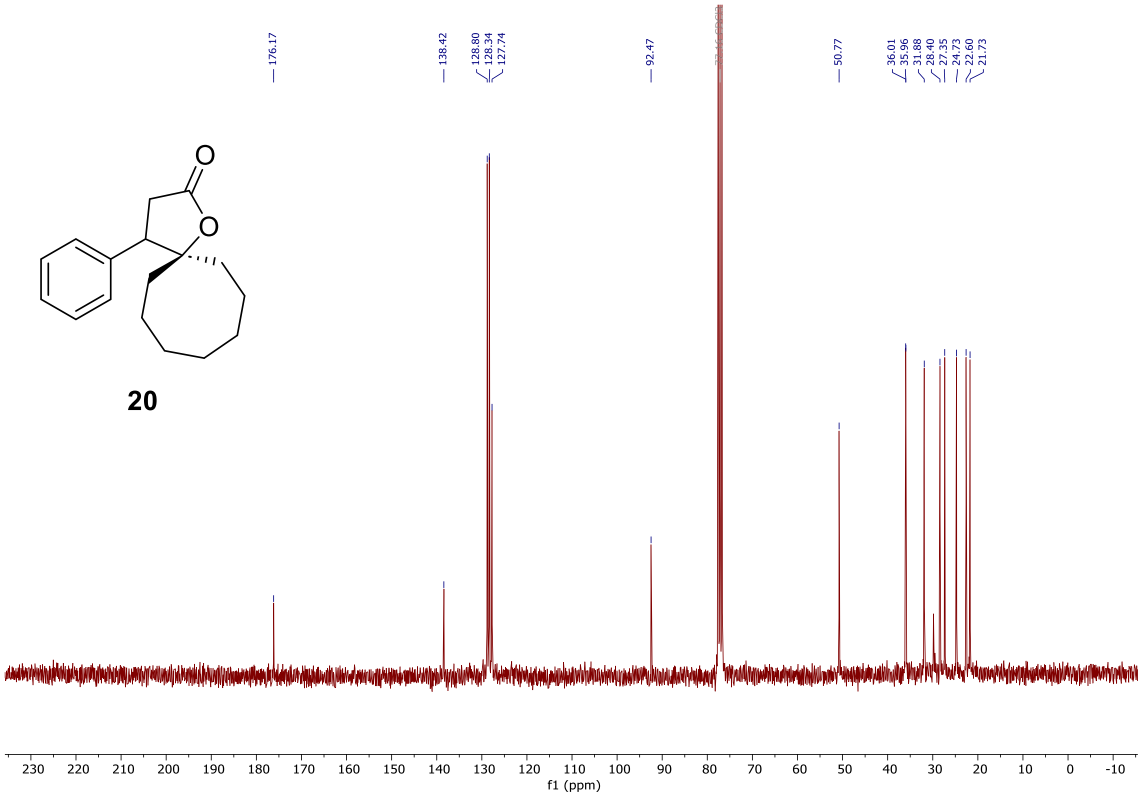
176.17
138.42
128.80
128.34
127.74
92.47
50.77
36.01
35.96
31.88
28.40
27.35
24.73
22.60
21.73
O
O
20
230 220 210 200 190 180 170 160 150 140 130 120 110 100 90 80 70 60 50 40 30 20 10 0 -10
f1 (ppm)

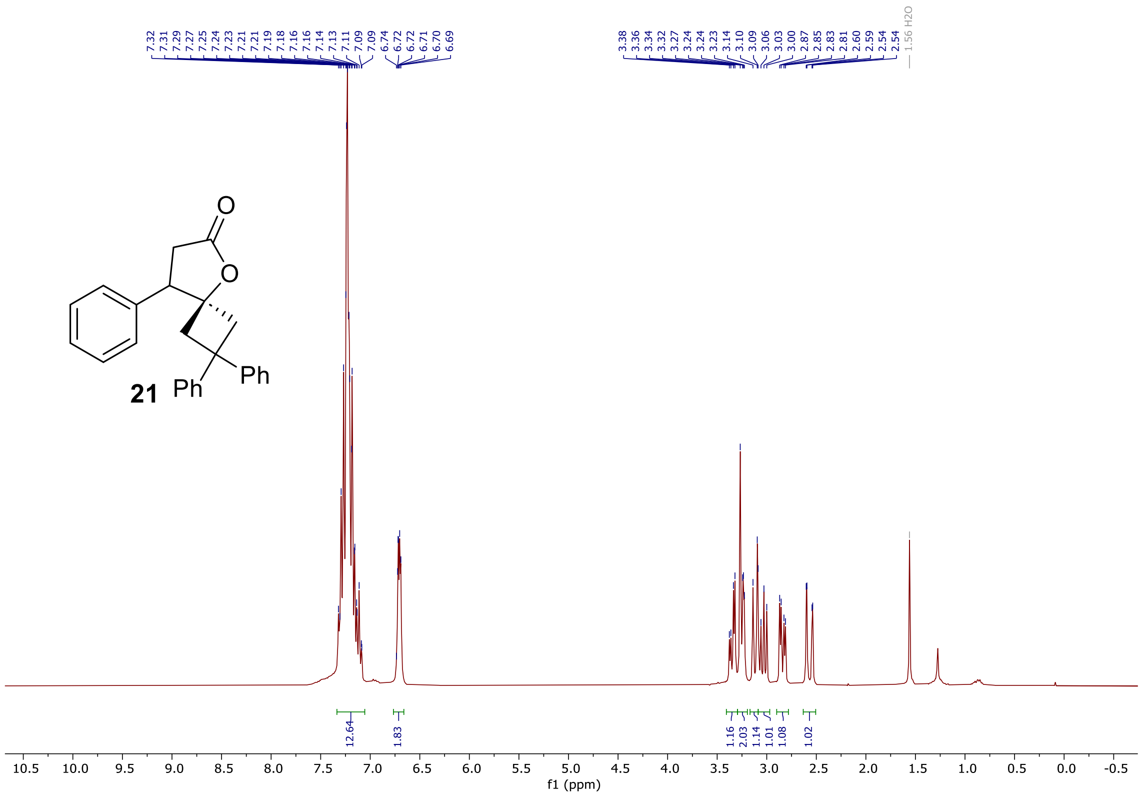
7.32 7.31 7.29 7.27 7.25 7.24 7.23 7.21 7.21 7.19 7.18 7.16 7.16 7.14 7.13 7.11 7.09 6.74 6.72 6.72 6.71 6.70 6.69
3.38 3.36 3.34 3.32 3.27 3.24 3.24 3.23 3.14 3.10 3.09 3.06 3.03 3.00 2.87 2.85 2.83 2.81 2.60 2.59 2.54
1.56 H2O
21
Ph
Ph
12.64 1.83 1.16 2.03 1.14 1.01 1.08 1.02
f1 (ppm)

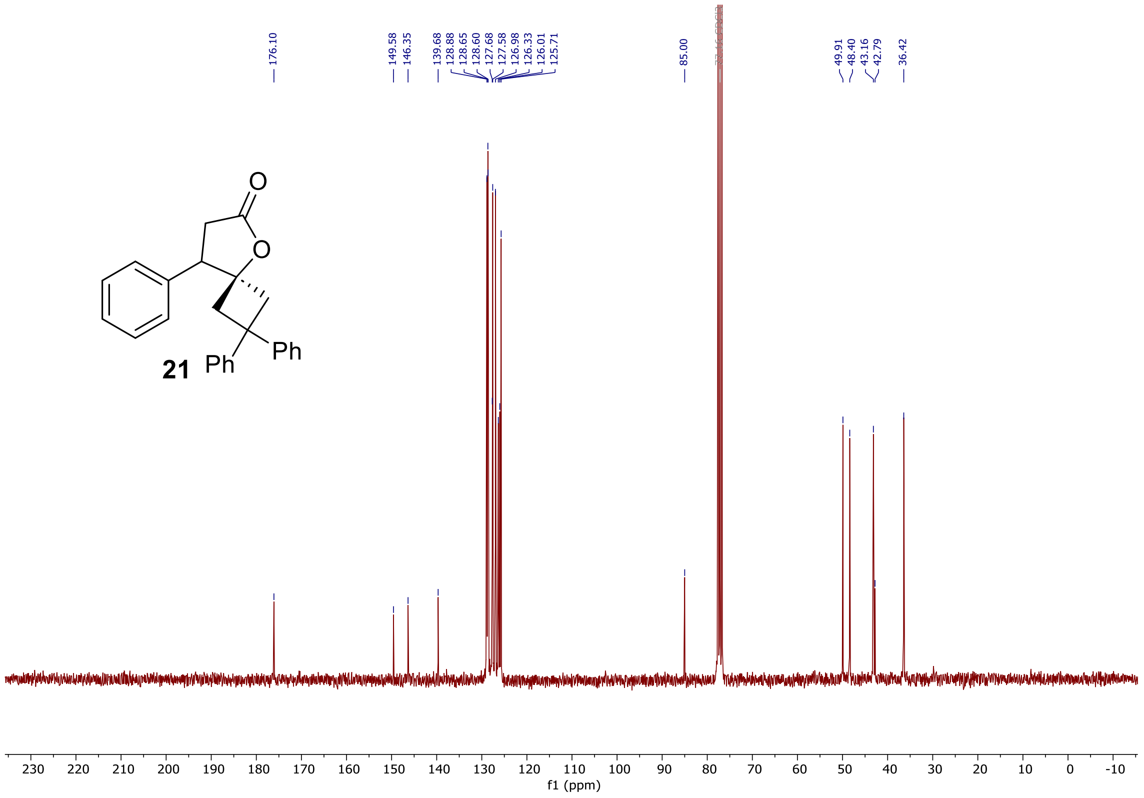
176.10
149.58 146.35 139.68 128.88 128.65 128.60 127.68 127.58 126.98 126.33 126.01 125.71
85.00
49.91 48.40 43.16 42.79 36.42
21
Ph
Ph
f1 (ppm)

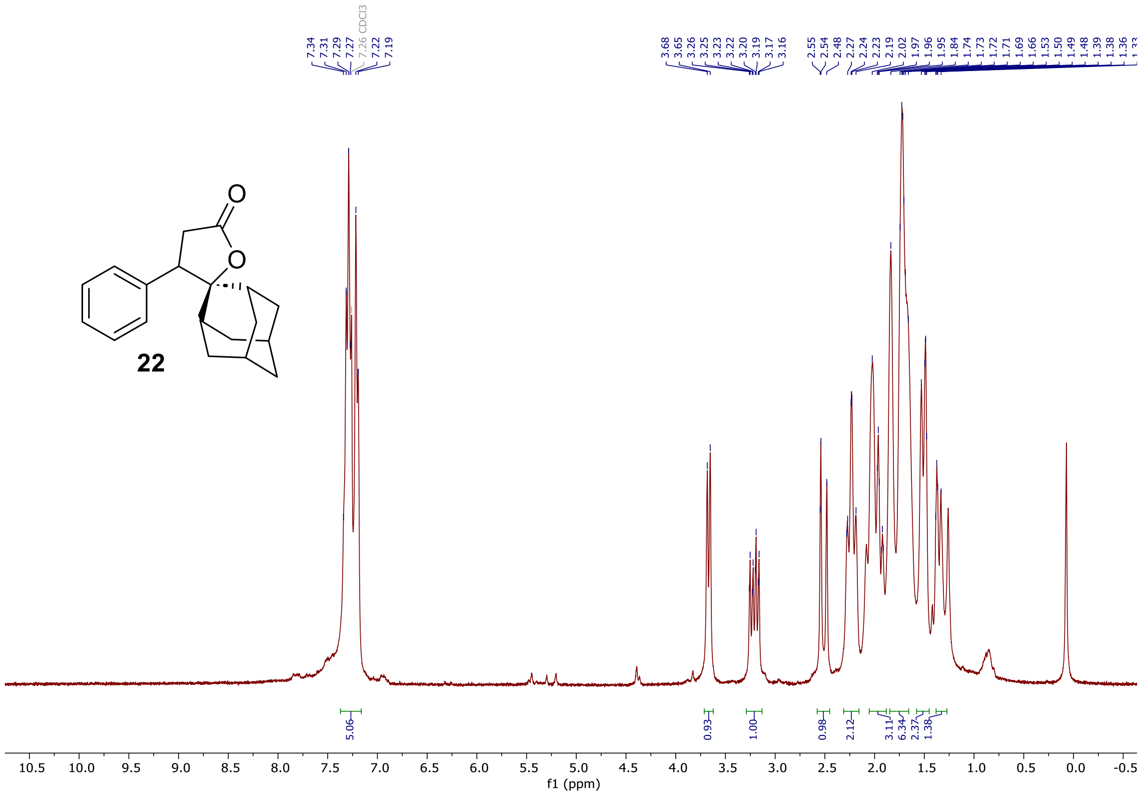
7.34
7.31
7.29
7.27
7.26 CDCl3
7.22
7.19
3.68
3.65
3.26
3.25
3.23
3.22
3.20
3.19
3.17
3.16
2.55
2.54
2.48
2.27
2.24
2.23
2.19
2.02
1.97
1.96
1.95
1.84
1.74
1.73
1.72
1.71
1.69
1.66
1.53
1.50
1.49
1.48
1.39
1.38
1.36
22
5.06
0.93
1.00
0.98
2.12
3.11
6.34
2.37
1.38
f1 (ppm)

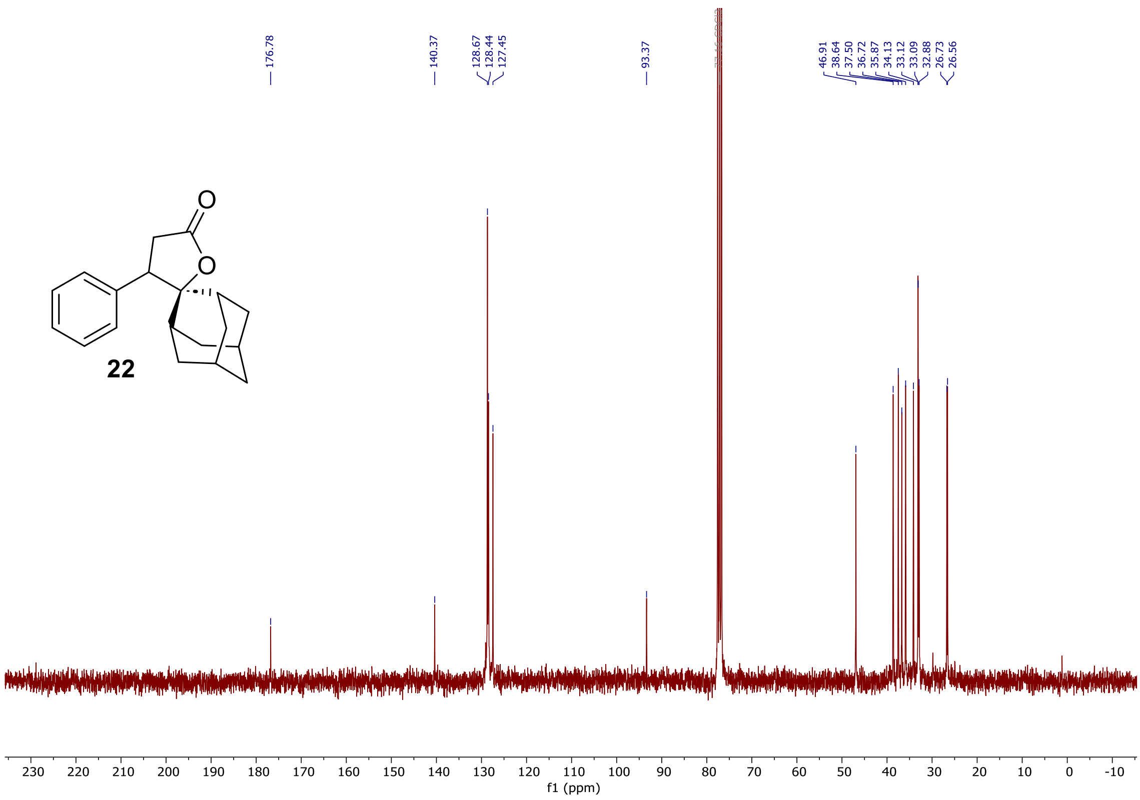
176.78
140.37
128.67
128.44
127.45
93.37
46.91
38.64
37.50
36.72
35.87
34.13
33.12
33.09
32.88
26.73
26.56
22
f1 (ppm)

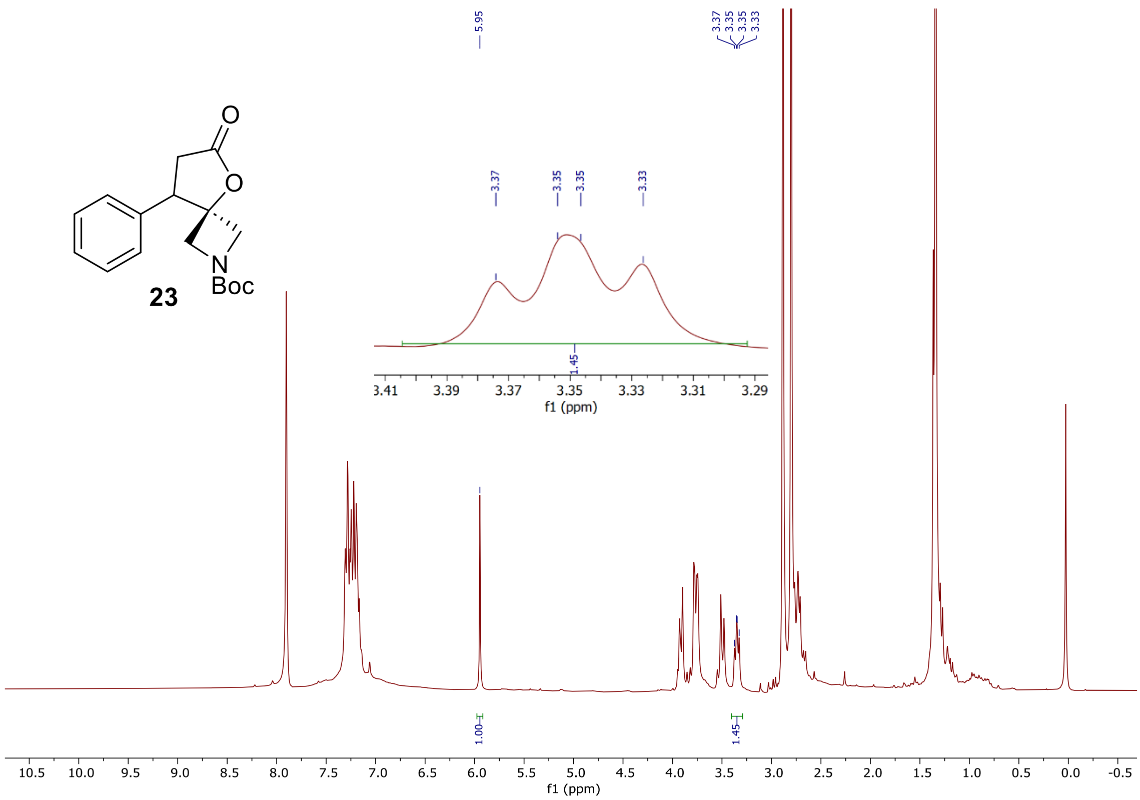

23
Boc
N
O
O
5.95
3.37
3.35
3.35
3.33
1.45
1.00
1.45
3.41 3.39 3.37 3.35 3.33 3.31 3.29
f1 (ppm)
10.5 10.0 9.5 9.0 8.5 8.0 7.5 7.0 6.5 6.0 5.5 5.0 4.5 4.0 3.5 3.0 2.5 2.0 1.5 1.0 0.5 0.0 -0.5
f1 (ppm)

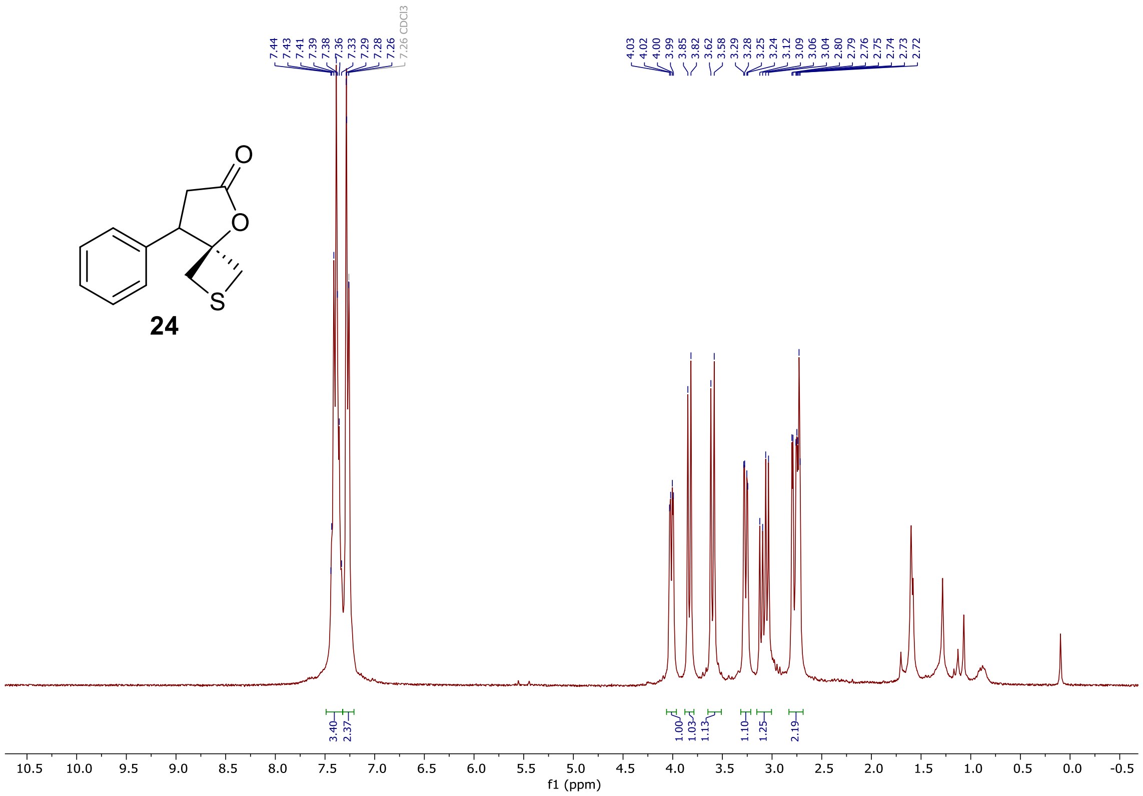

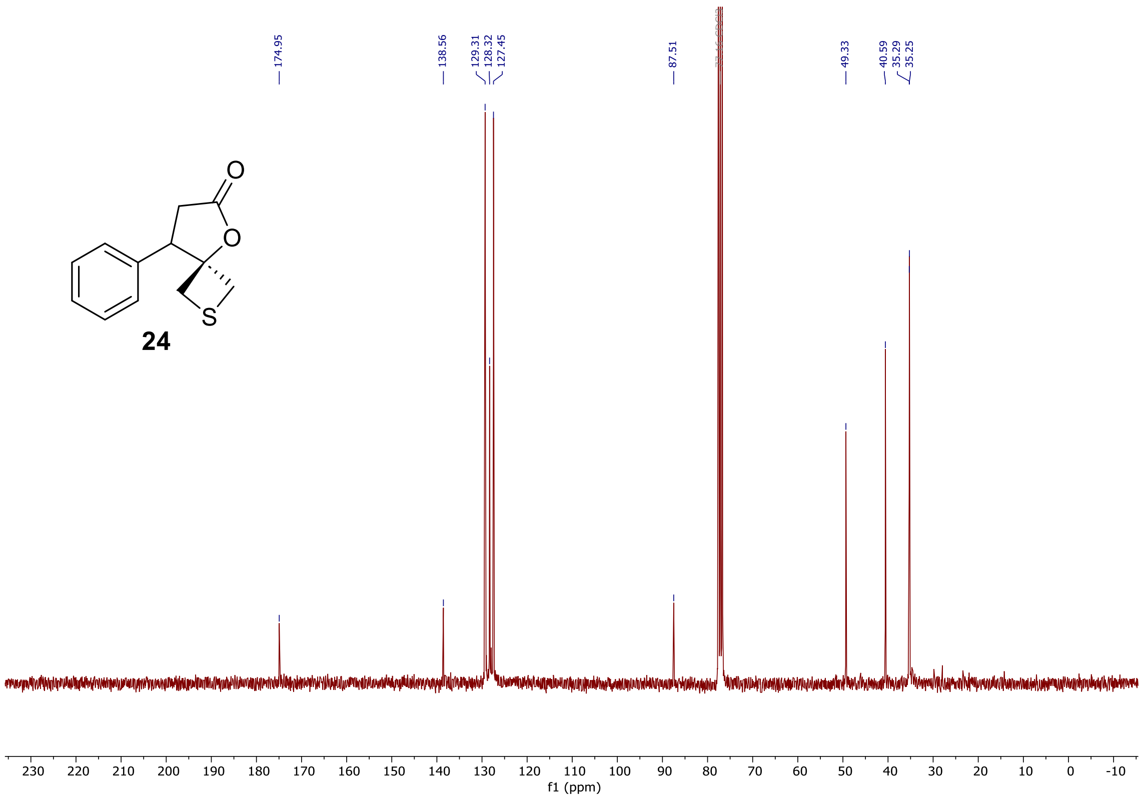

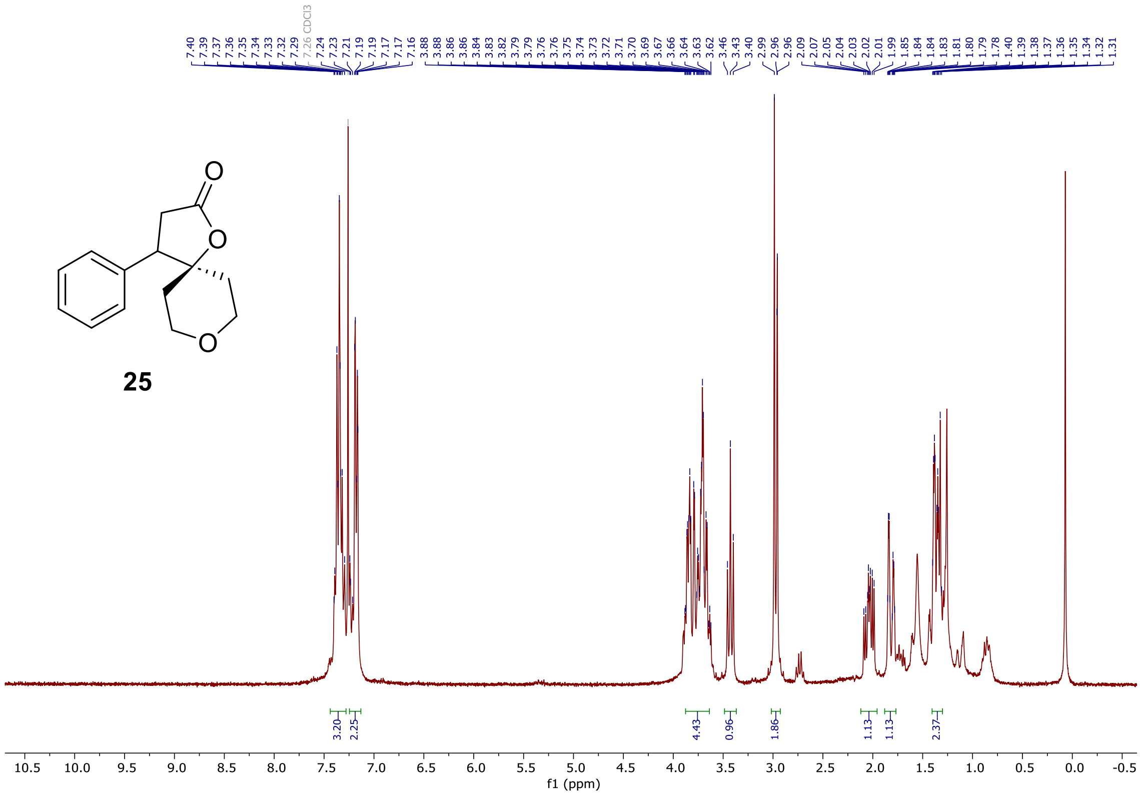
7.40 7.39 7.37 7.36 7.35 7.34 7.33 7.32 7.29 7.26 CDCl3 7.24 7.23 7.21 7.19 7.19 7.17 7.16 3.88 3.86 3.86 3.84 3.83 3.82 3.79 3.79 3.76 3.75 3.74 3.73 3.72 3.71 3.70 3.69 3.67 3.66 3.64 3.63 3.62 3.46 3.43 3.40 2.99 2.96 2.96 2.09 2.07 2.05 2.04 2.03 2.02 2.01 1.99 1.85 1.84 1.84 1.83 1.81 1.80 1.79 1.78 1.40 1.39 1.38 1.37 1.36 1.35 1.34 1.32 1.31
O
O
O
25
3.20
2.25
4.43
0.96
1.86
1.13
1.13
2.37
f1 (ppm)

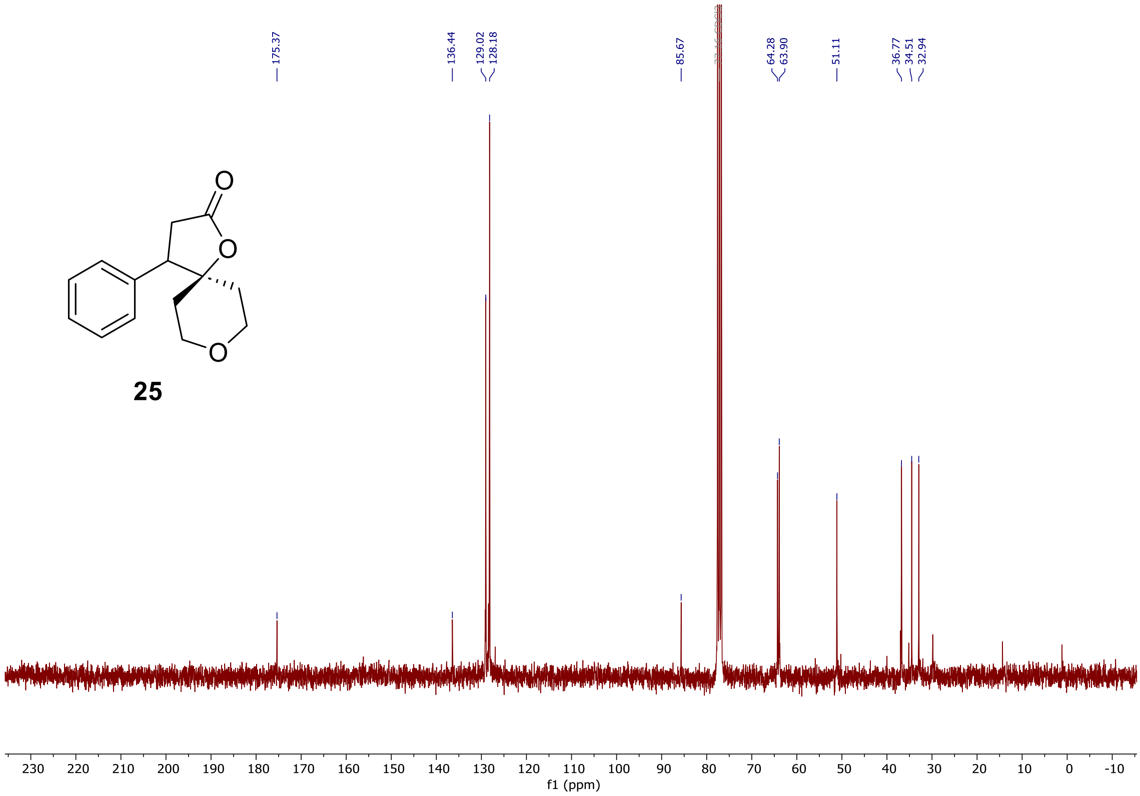
175.37
136.44
129.02
128.18
85.67
64.28
63.90
51.11
36.77
34.51
32.94
O
O
O
25
f1 (ppm)

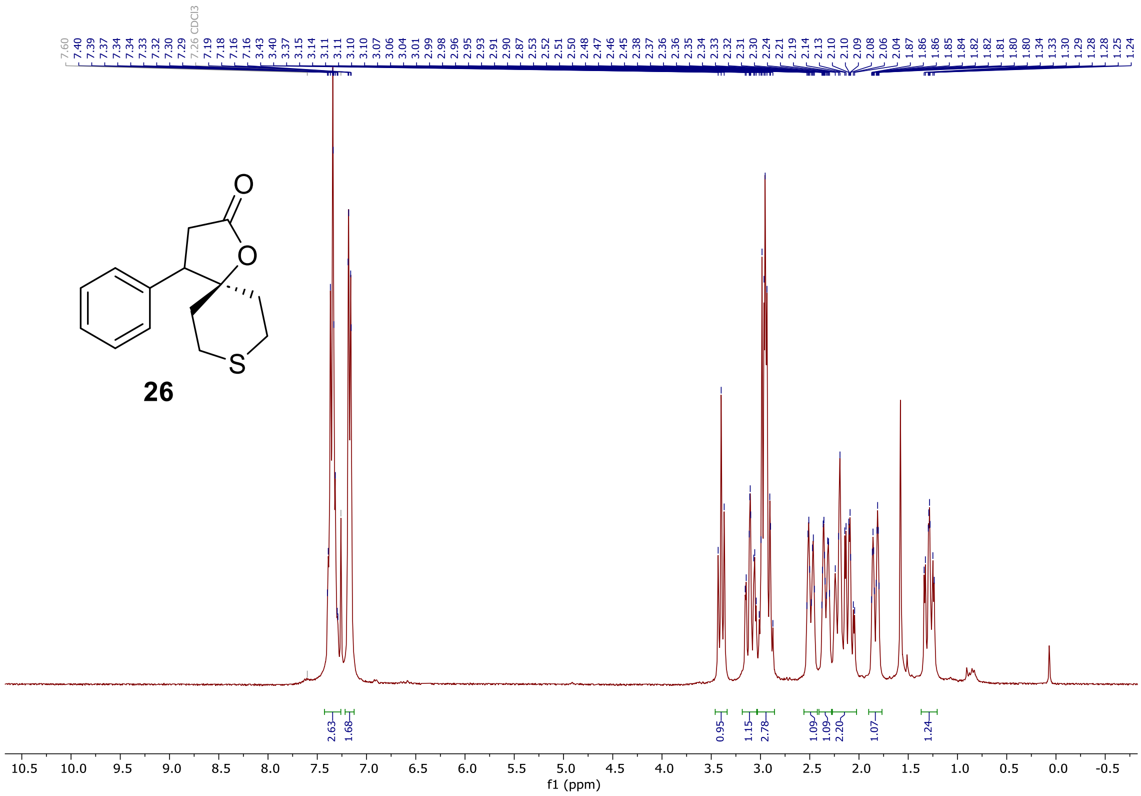
7.60
7.40
7.39
7.37
7.34
7.34
7.33
7.32
7.30
7.29
7.26 CDCl3
7.19
7.18
7.16
7.16
3.43
3.40
3.37
3.15
3.14
3.11
3.11
3.10
3.10
3.07
3.06
3.04
3.01
2.99
2.98
2.96
2.95
2.93
2.91
2.90
2.87
2.53
2.52
2.51
2.50
2.48
2.47
2.46
2.45
2.38
2.37
2.36
2.36
2.35
2.34
2.33
2.32
2.31
2.30
2.24
2.21
2.19
2.14
2.13
2.10
2.10
2.09
2.08
2.06
2.04
1.87
1.86
1.86
1.85
1.84
1.82
1.81
1.80
1.80
1.34
1.33
1.30
1.29
1.28
1.25
1.24
26
2.63
1.68
0.95
1.15
2.78
1.09
1.09
2.20
1.07
1.24
f1 (ppm)

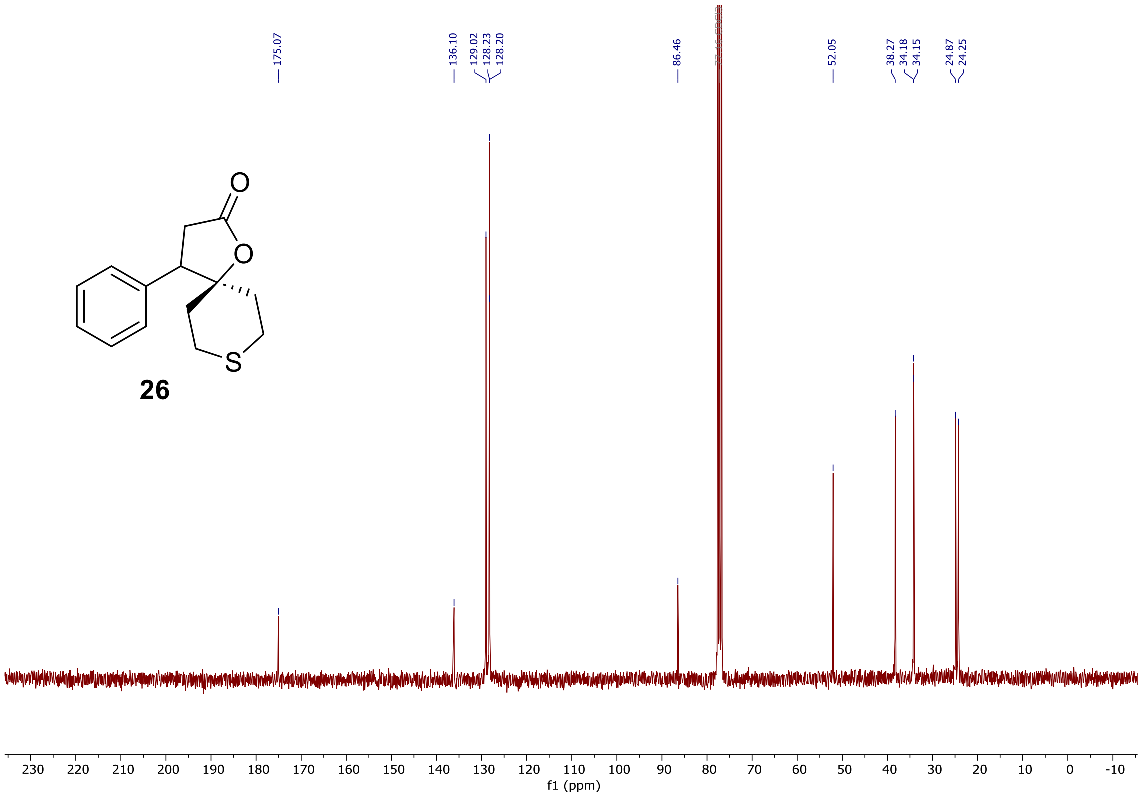
175.07
136.10
129.02
128.23
128.20
86.46
52.05
38.27
34.18
34.15
24.87
24.25
26
f1 (ppm)

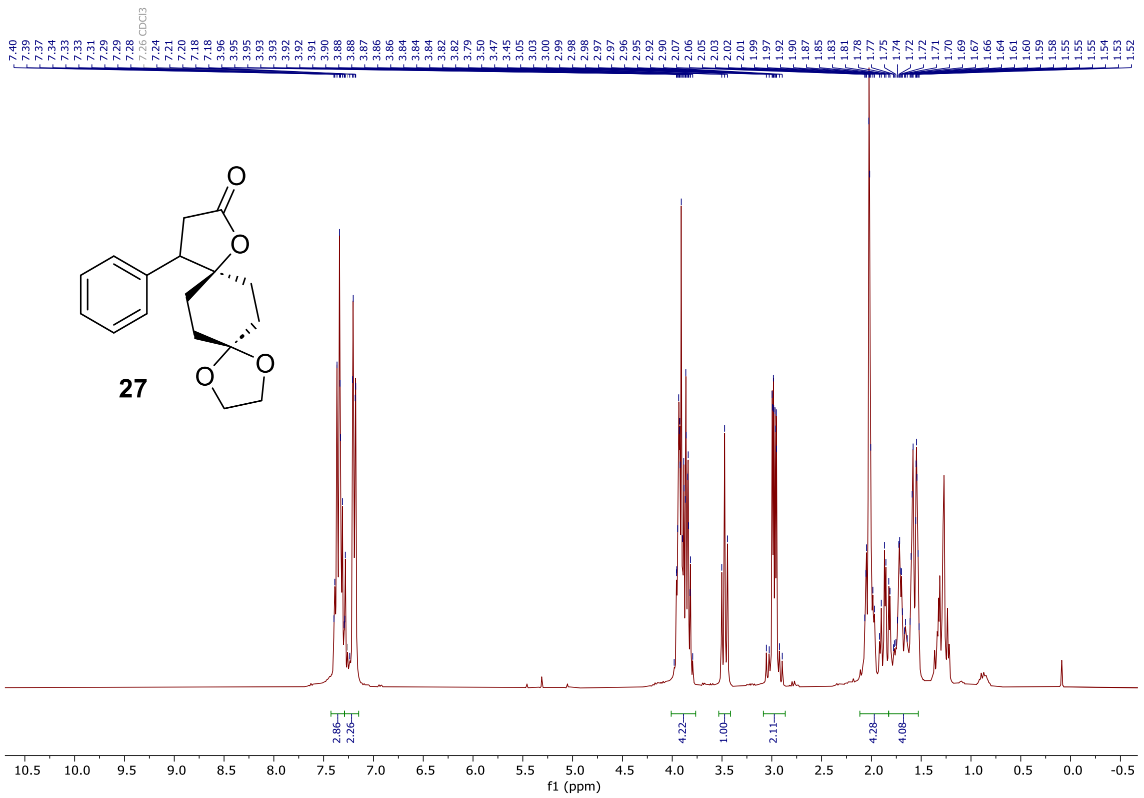
27
2.86
2.26
4.22
1.00
2.11
4.28
4.08
f1 (ppm)

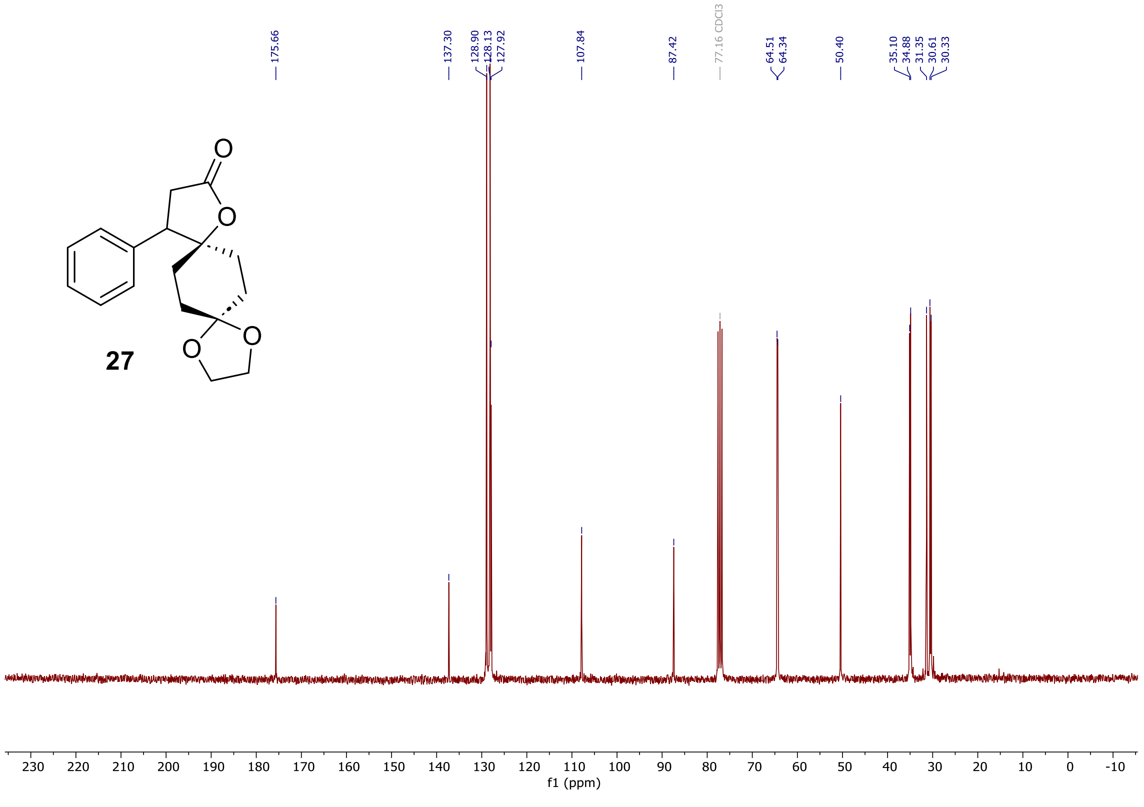
175.66
137.30
128.90
128.13
127.92
107.84
87.42
77.16 CDCl3
64.51
64.34
50.40
35.10
34.88
31.35
30.61
30.33
27
f1 (ppm)

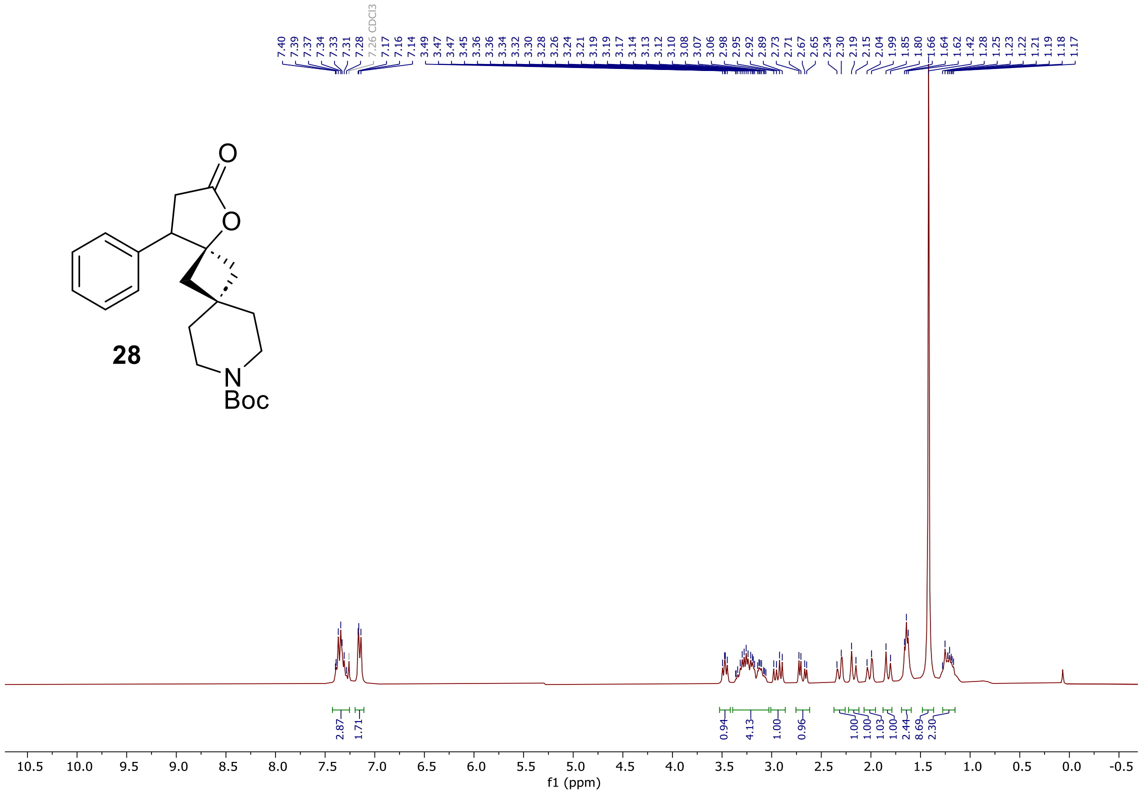
7.40 7.39 7.37 7.34 7.33 7.31 7.28 7.26 CDCl3 7.17 7.16 7.14 3.49 3.47 3.47 3.45 3.36 3.36 3.34 3.32 3.30 3.28 3.26 3.24 3.21 3.19 3.19 3.17 3.14 3.13 3.12 3.10 3.08 3.07 3.06 2.98 2.95 2.92 2.89 2.73 2.71 2.67 2.65 2.34 2.30 2.19 2.15 2.04 1.99 1.85 1.80 1.66 1.64 1.62 1.42 1.28 1.25 1.23 1.22 1.21 1.19 1.18 1.17
O
O
N
Boc
28
2.87 1.71 0.94 4.13 1.00 0.96 1.00 1.00 1.03 1.00 2.44 8.69 2.30
10.5 10.0 9.5 9.0 8.5 8.0 7.5 7.0 6.5 6.0 5.5 5.0 4.5 4.0 3.5 3.0 2.5 2.0 1.5 1.0 0.5 0.0 -0.5
f1 (ppm)

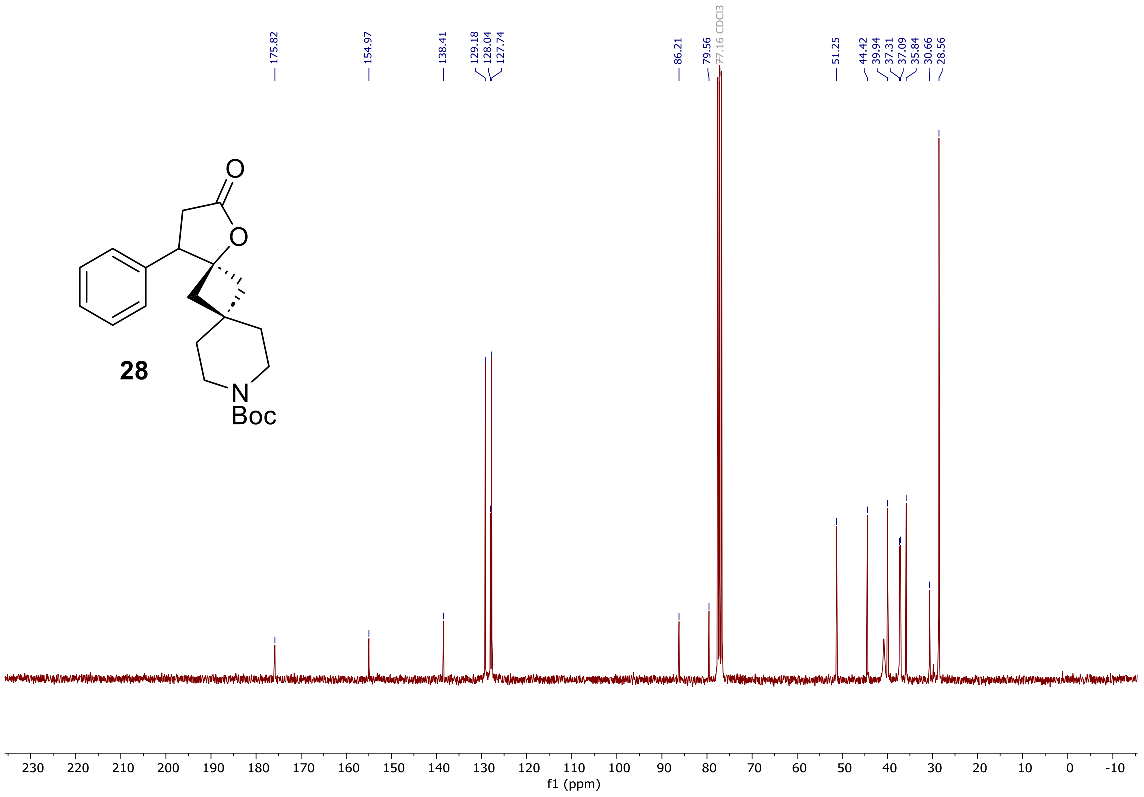
175.82
154.97
138.41
129.18 128.04 127.74
86.21
79.56 77.16 CDCl3
51.25
44.42 39.94 37.31 37.09 35.84 30.66 28.56
O
O
N
Boc
28
230 220 210 200 190 180 170 160 150 140 130 120 110 100 90 80 70 60 50 40 30 20 10 0 -10
f1 (ppm)

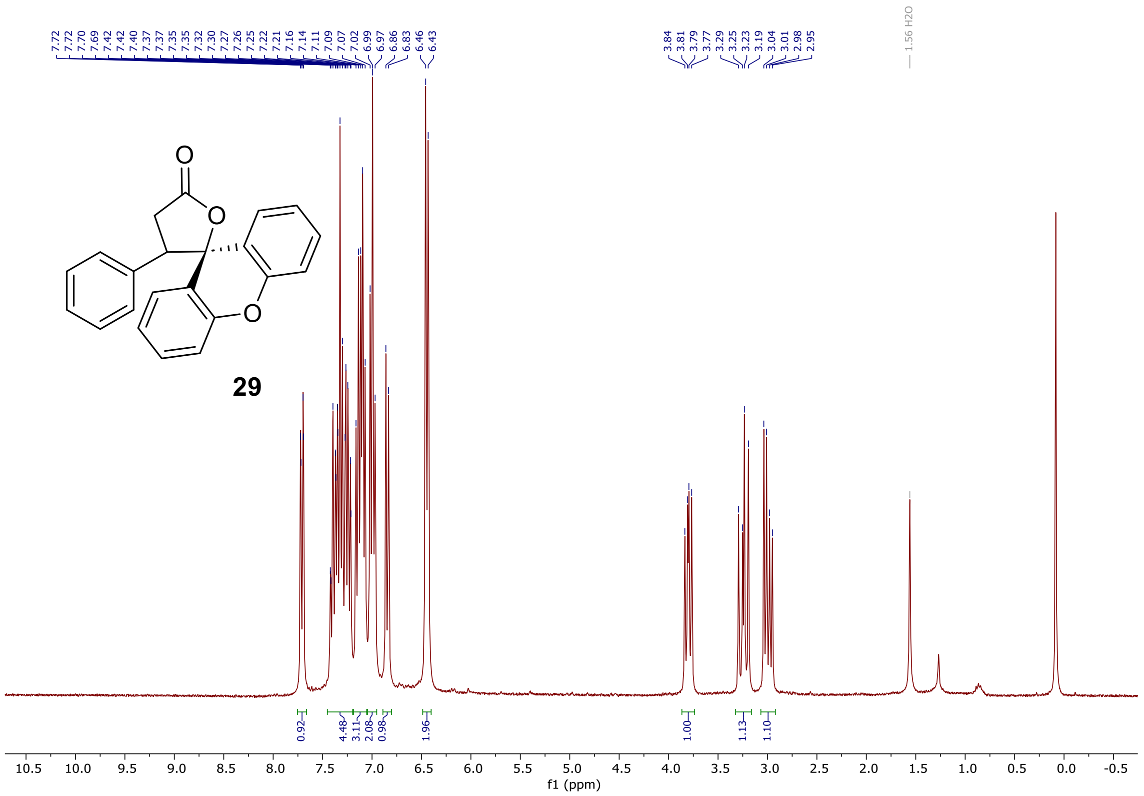
7.72 7.72 7.70 7.69 7.42 7.42 7.40 7.37 7.37 7.35 7.35 7.32 7.30 7.27 7.26 7.25 7.22 7.21 7.16 7.14 7.11 7.09 7.07 7.02 6.99 6.97 6.86 6.83 6.46 6.43
3.84 3.81 3.79 3.77 3.29 3.25 3.23 3.19 3.04 3.01 2.98 2.95
1.56 H2O
O
O
O
29
0.92
4.48
3.11
2.08
0.98
1.96
1.00
1.13
1.10
10.5 10.0 9.5 9.0 8.5 8.0 7.5 7.0 6.5 6.0 5.5 5.0 4.5 4.0 3.5 3.0 2.5 2.0 1.5 1.0 0.5 0.0 -0.5
f1 (ppm)

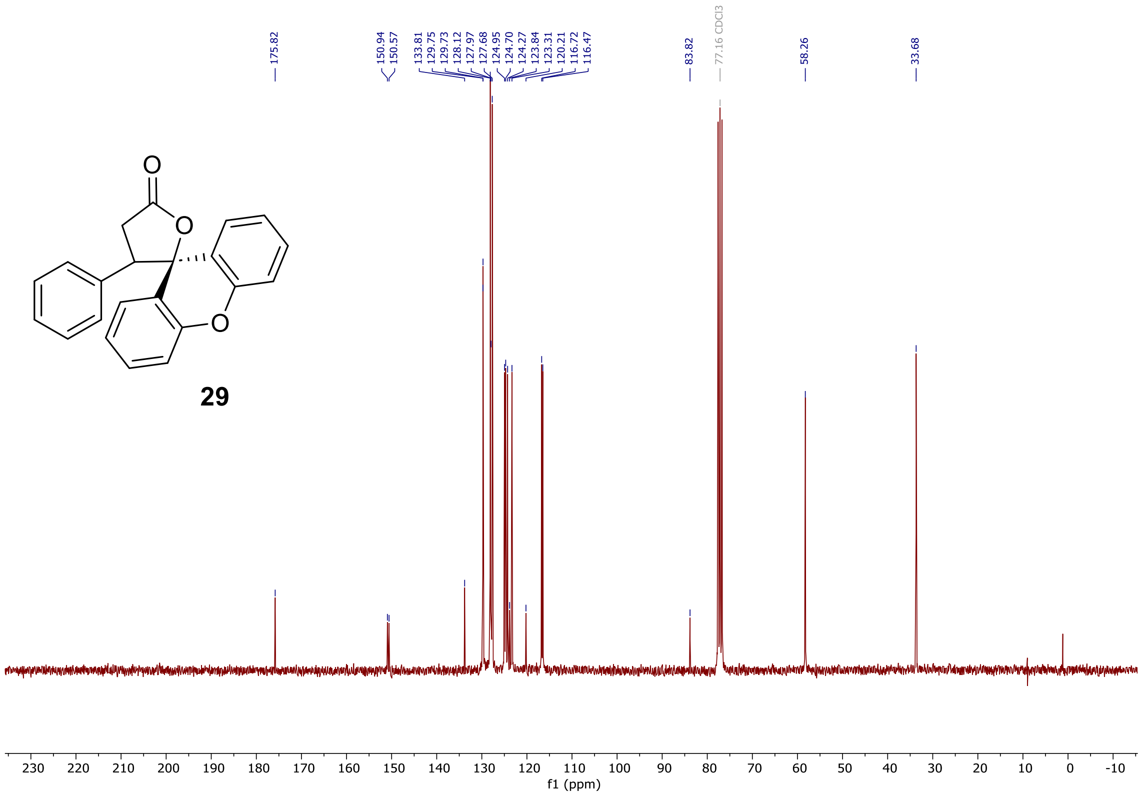
175.82
150.94 150.57
133.81 129.75 129.73 128.12 127.97 127.68 124.95 124.70 124.27 123.84 123.31 120.21 116.72 116.47
83.82
77.16 CDCl3
58.26
33.68
O
O
O
29
230 220 210 200 190 180 170 160 150 140 130 120 110 100 90 80 70 60 50 40 30 20 10 0 -10
f1 (ppm)

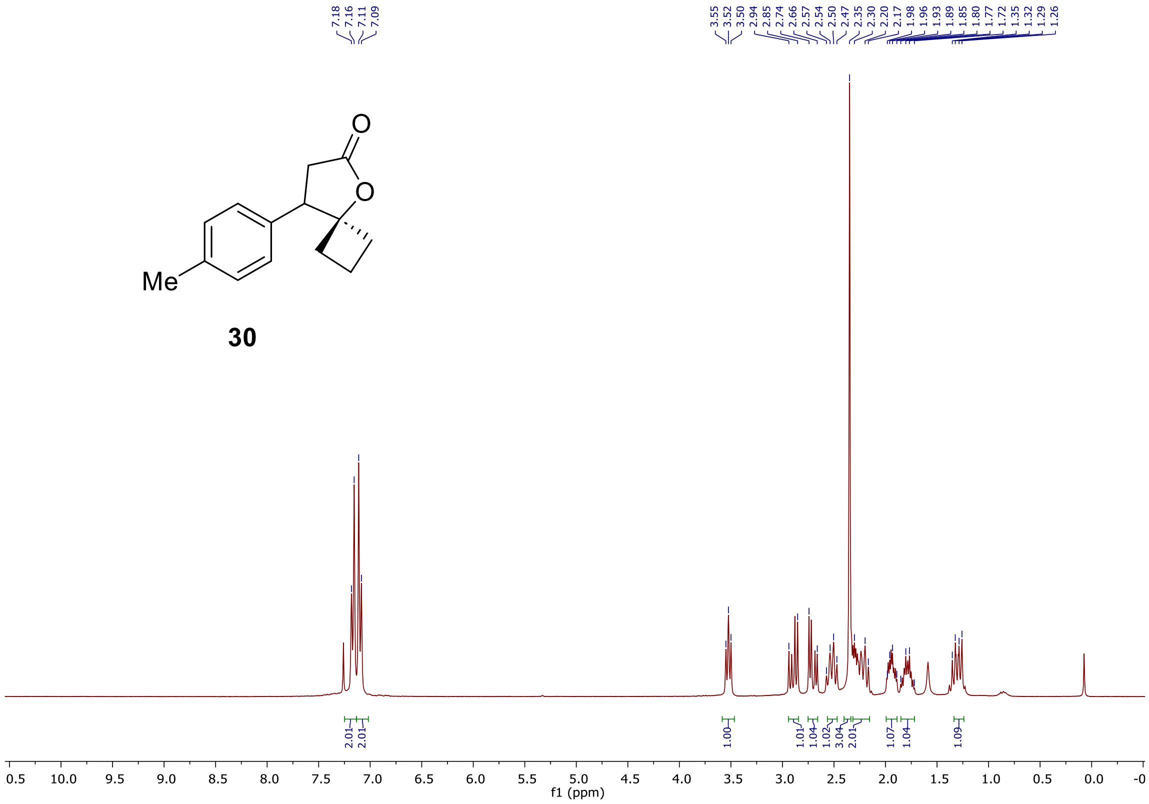

30

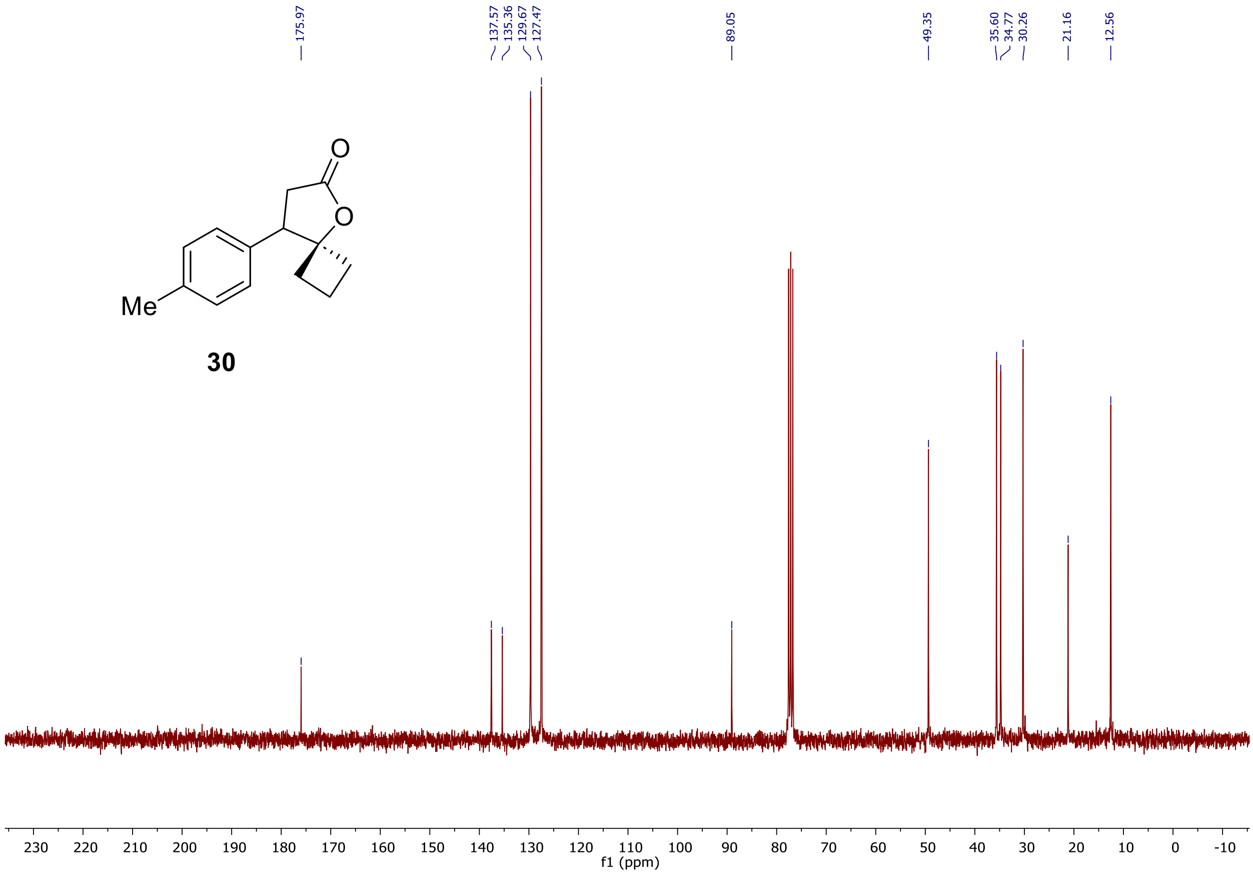

30

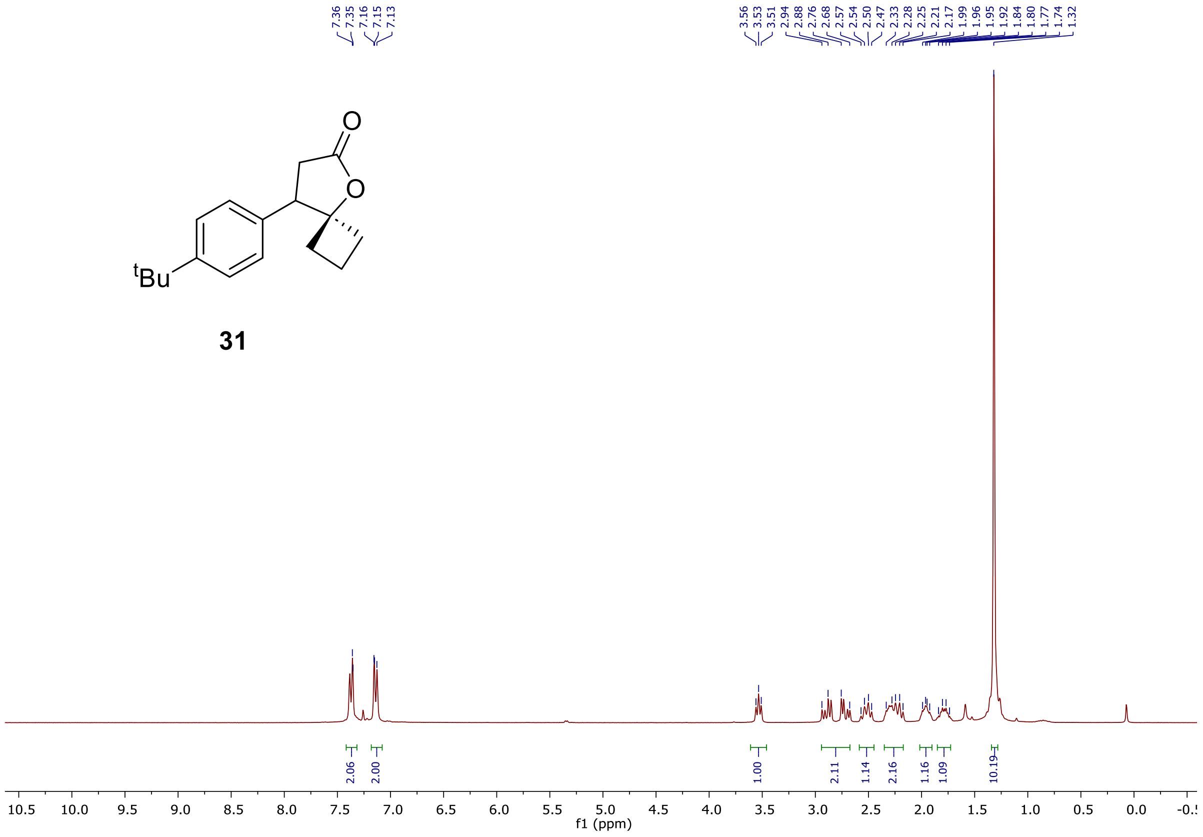

7.36
7.35
7.16
7.15
7.13
3.56
3.53
3.51
2.94
2.88
2.76
2.68
2.57
2.54
2.50
2.47
2.33
2.28
2.25
2.21
2.17
1.99
1.96
1.95
1.92
1.84
1.80
1.77
1.74
1.32
O
O
tBu
31
2.06
2.00
1.00
2.11
1.14
2.16
1.16
1.09
10.19
f1 (ppm)

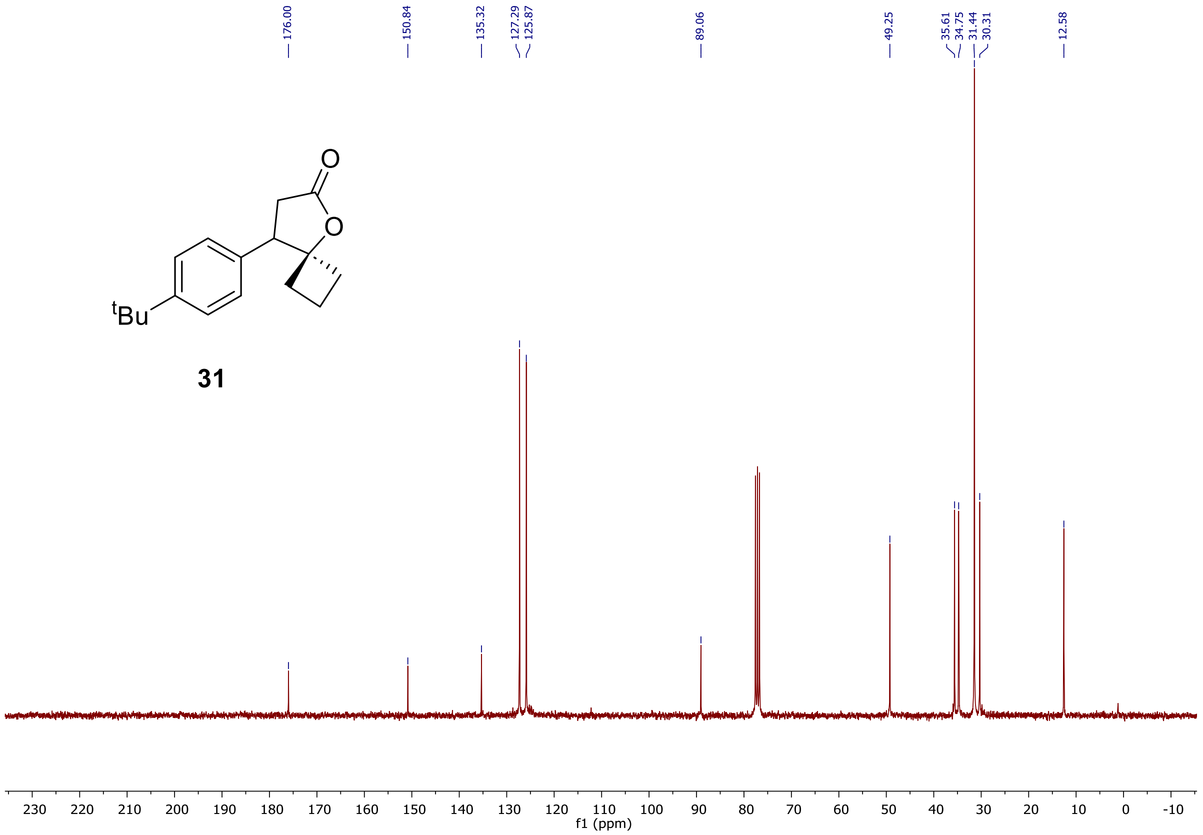

176.00
150.84
135.32
127.29
125.87
89.06
49.25
35.61
34.75
31.44
30.31
12.58
O
O
tBu
31
f1 (ppm)

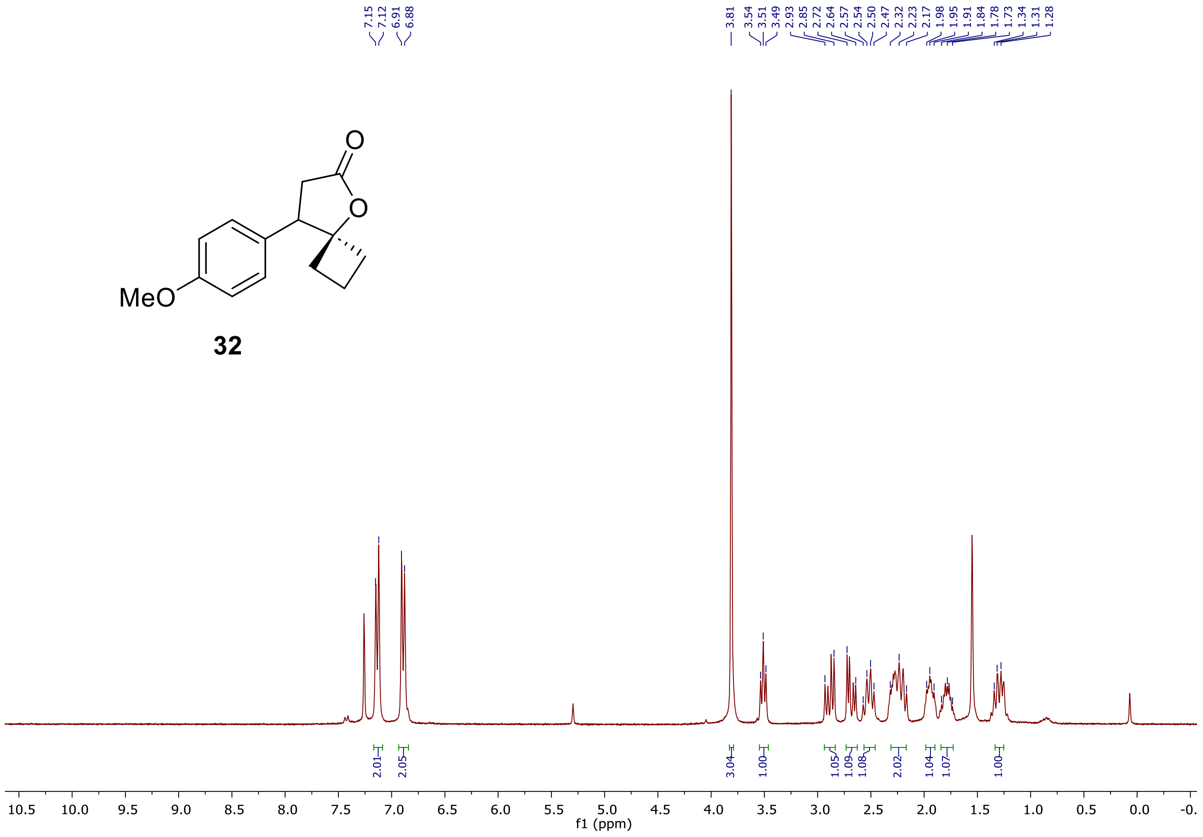

MeO
32
7.15
7.12
6.91
6.88
3.81
3.54
3.51
3.49
2.93
2.85
2.72
2.64
2.57
2.54
2.50
2.47
2.32
2.23
2.17
1.98
1.95
1.91
1.84
1.78
1.73
1.34
1.31
1.28
2.01
2.05
3.04
1.00
1.05
1.09
1.08
2.02
1.04
1.07
1.00
f1 (ppm)

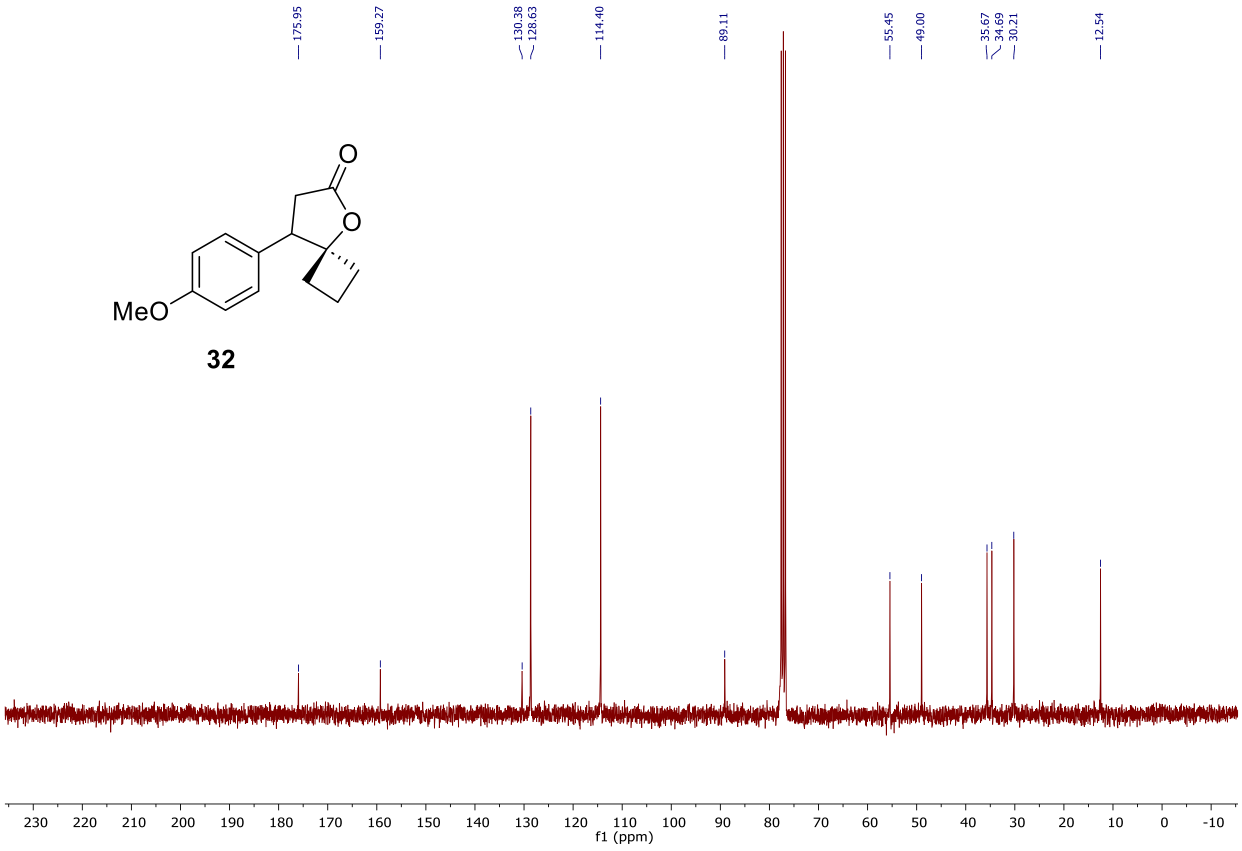

MeO
32
175.95
159.27
130.38
128.63
114.40
89.11
55.45
49.00
35.67
34.69
30.21
12.54
f1 (ppm)

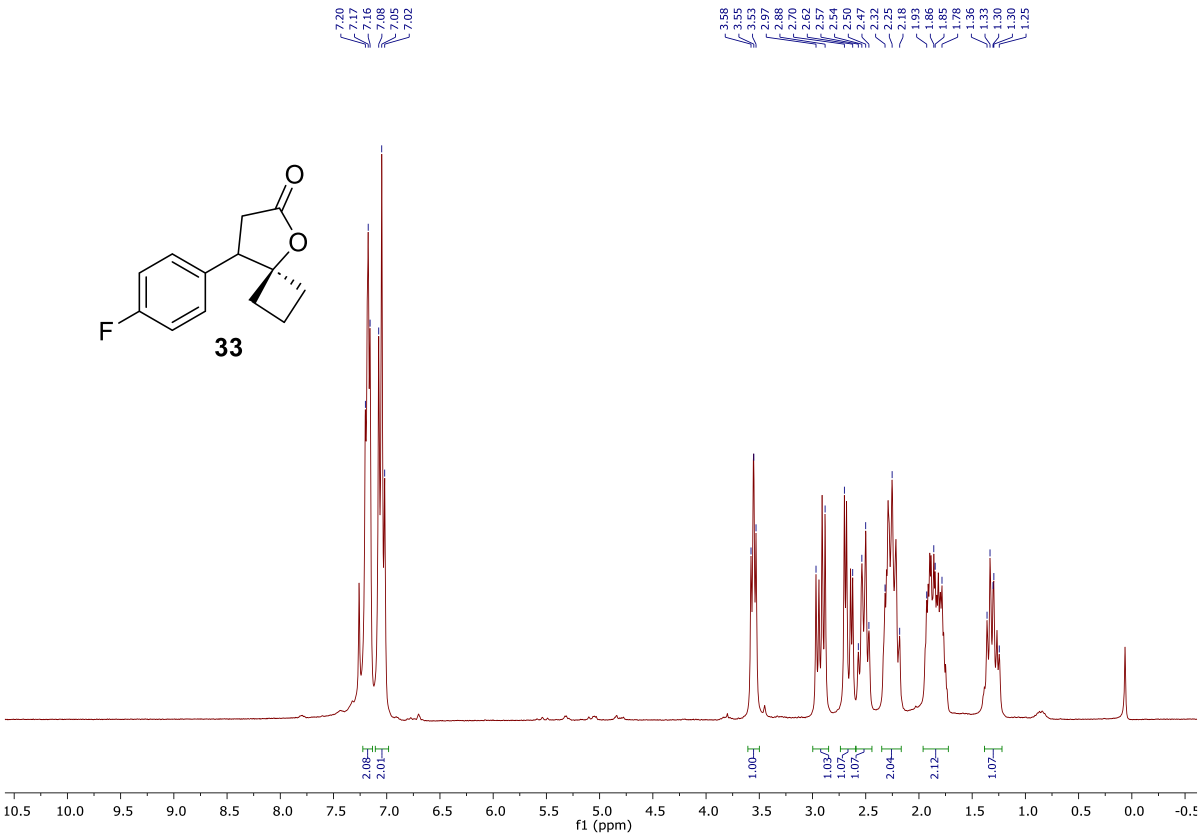
7.20
7.17
7.16
7.08
7.05
7.02
3.58
3.55
3.53
2.97
2.88
2.70
2.62
2.57
2.54
2.50
2.47
2.32
2.25
2.18
1.93
1.86
1.85
1.78
1.36
1.33
1.30
1.30
1.25
F
O
O
33
2.08
2.01
1.00
1.03
1.07
1.07
2.04
2.12
1.07
f1 (ppm)

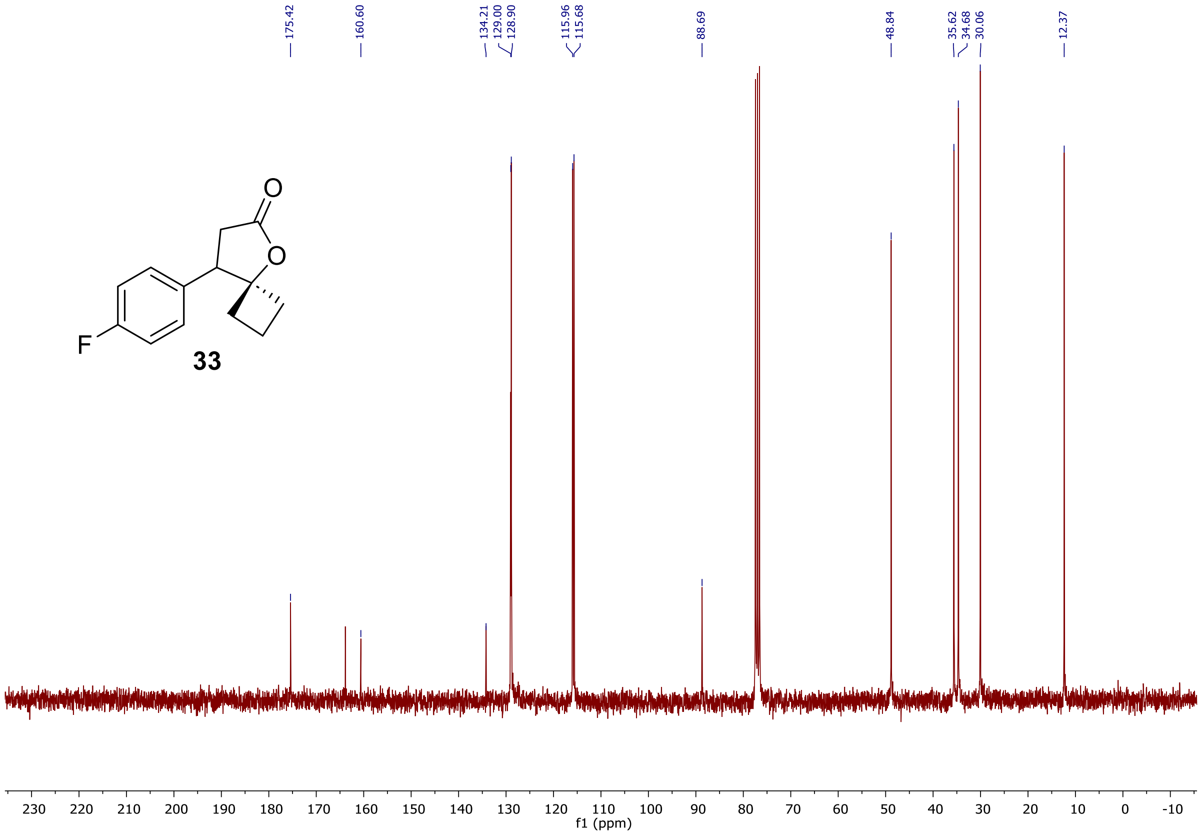
175.42
160.60
134.21
129.00
128.90
115.96
115.68
88.69
48.84
35.62
34.68
30.06
12.37
F
O
O
33
f1 (ppm)

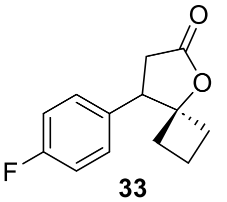

O
O
F

33

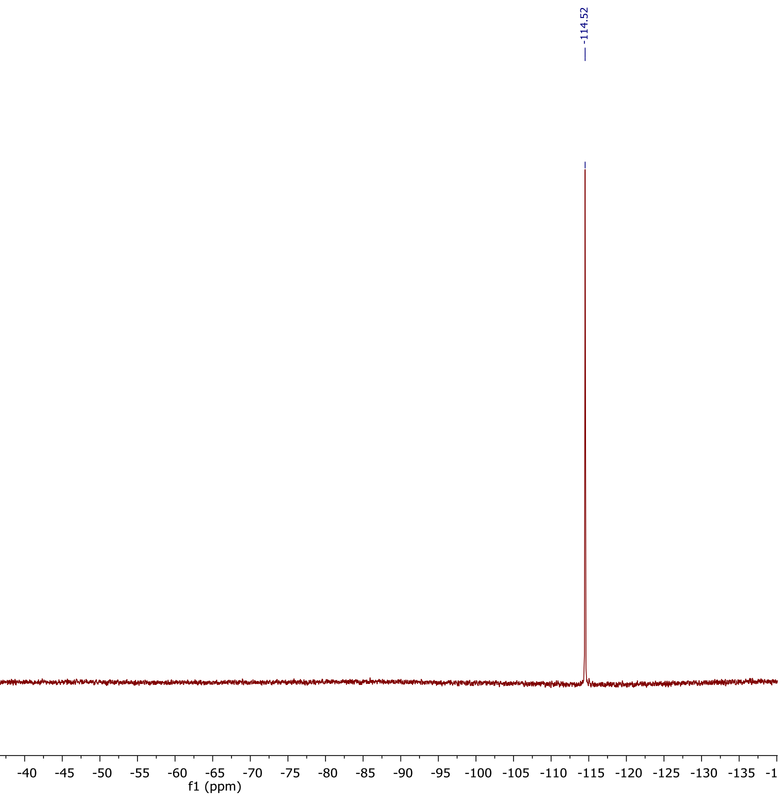

-114.52
10 5 0 -5 -10 -15 -20 -25 -30 -35 -40 -45 -50 -55 -60 -65 -70 -75 -80 -85 -90 -95 -100 -105 -110 -115 -120 -125 -130 -135
f1 (ppm)

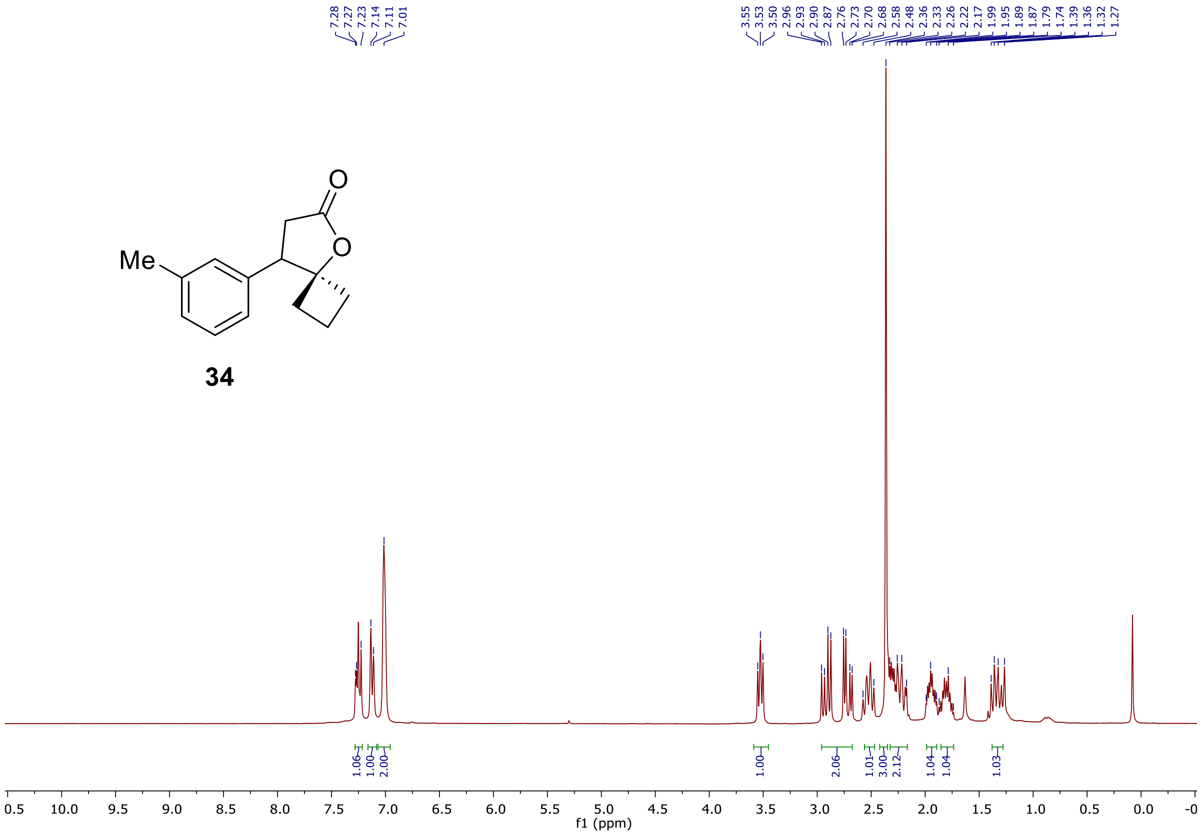
7.28
7.27
7.23
7.14
7.11
7.01
3.55
3.53
3.50
2.96
2.93
2.90
2.87
2.76
2.73
2.70
2.68
2.58
2.48
2.36
2.33
2.26
2.22
2.17
1.99
1.95
1.89
1.87
1.79
1.74
1.39
1.36
1.32
1.27
Me
O
O
34
1.06
1.00
2.00
1.00
2.06
1.01
3.00
2.12
1.04
1.04
1.03
f1 (ppm)

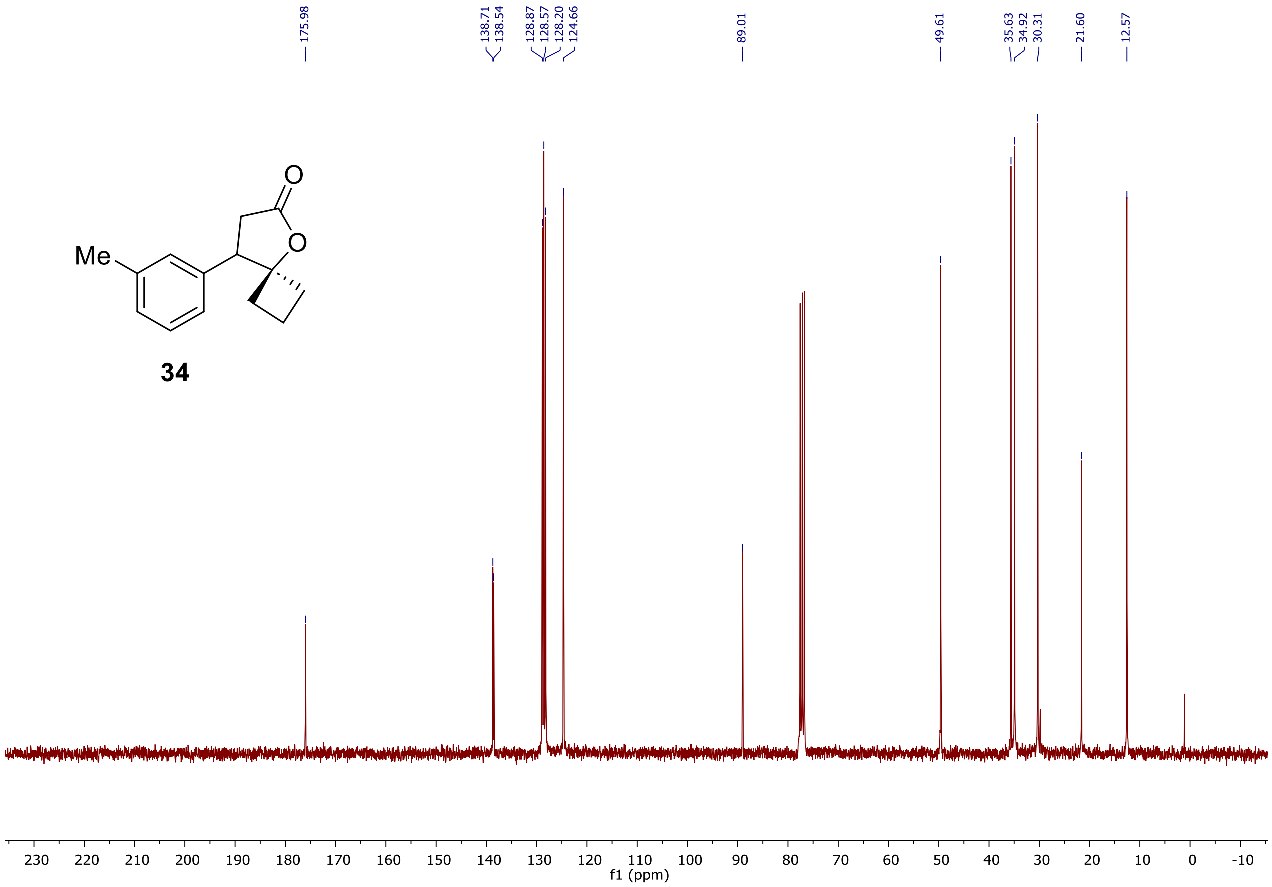
175.98
138.71
138.54
128.87
128.57
128.20
124.66
89.01
49.61
35.63
34.92
30.31
21.60
12.57
Me
O
O
34
f1 (ppm)

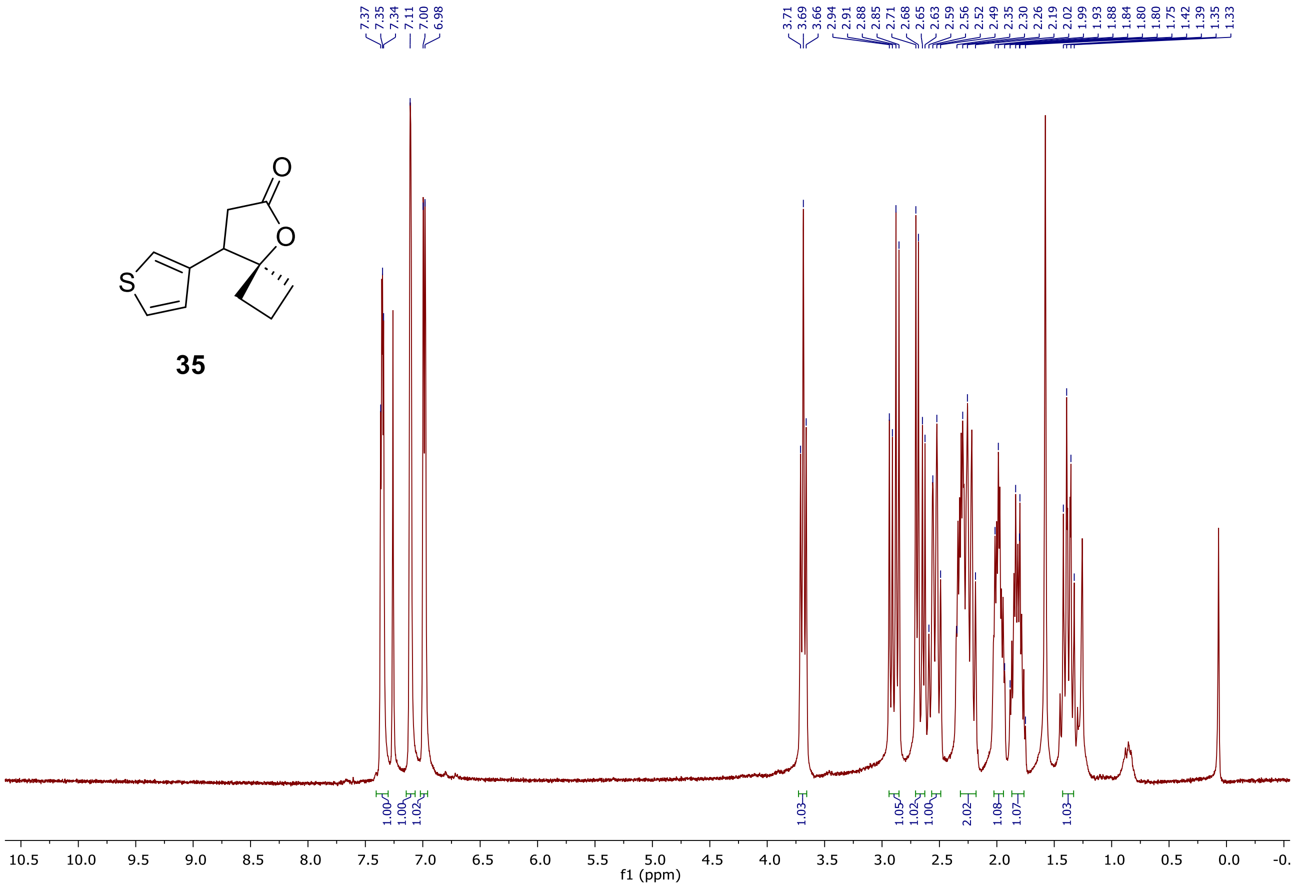

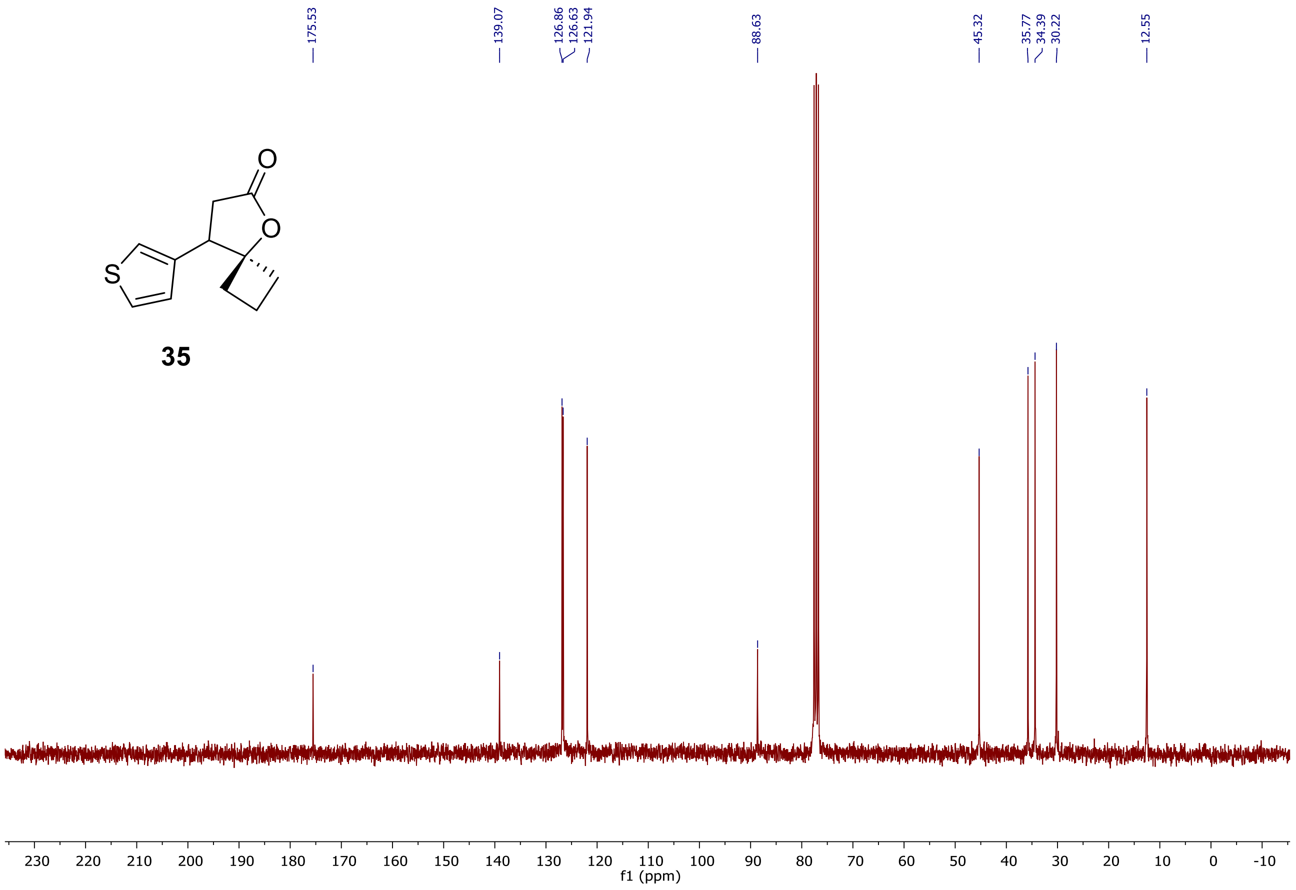

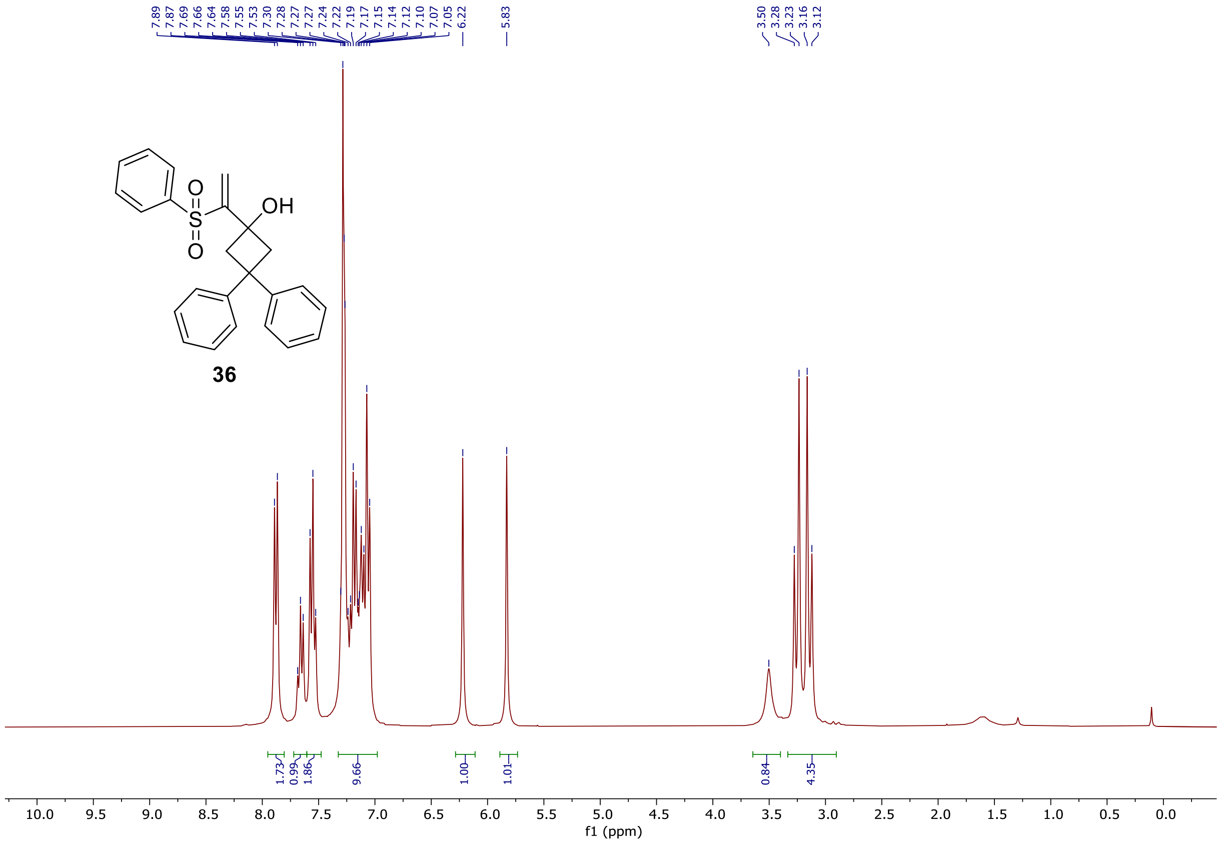
7.89
7.87
7.69
7.66
7.64
7.58
7.55
7.53
7.30
7.28
7.27
7.27
7.24
7.22
7.19
7.17
7.15
7.14
7.12
7.10
7.07
7.05
6.22
5.83
3.50
3.28
3.23
3.16
3.12
OH
36
1.73
0.99
1.86
9.66
1.00
1.01
0.84
4.35
f1 (ppm)

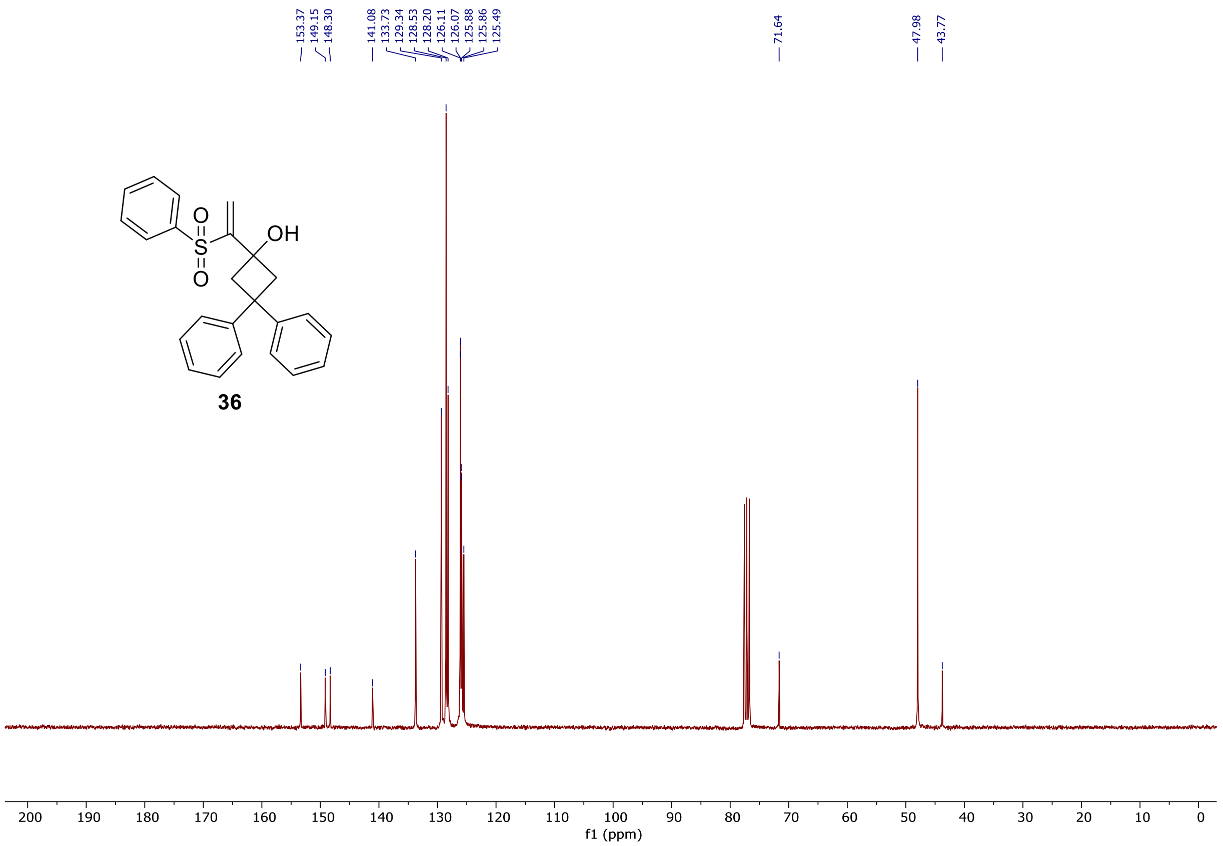
153.37
149.15
148.30
141.08
133.73
129.34
128.53
128.20
126.11
126.07
125.88
125.86
125.49
71.64
47.98
43.77
OH
36
f1 (ppm)

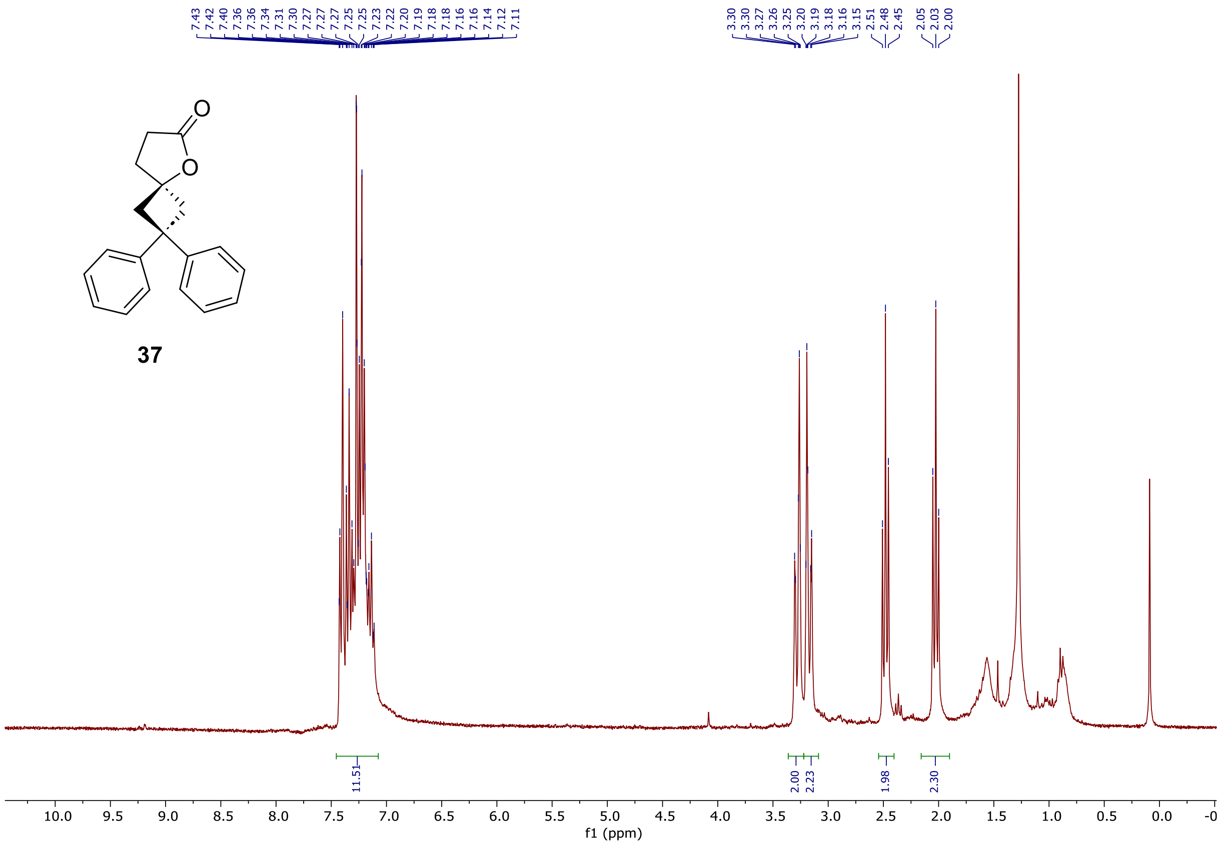
7.43 7.42 7.40 7.36 7.36 7.34 7.31 7.30 7.27 7.27 7.27 7.25 7.25 7.23 7.22 7.20 7.19 7.18 7.18 7.16 7.16 7.14 7.12 7.11
3.30 3.30 3.27 3.26 3.25 3.20 3.19 3.18 3.16 3.15 2.51 2.48 2.45 2.05 2.03 2.00
37
11.51
2.00
2.23
1.98
2.30
f1 (ppm)

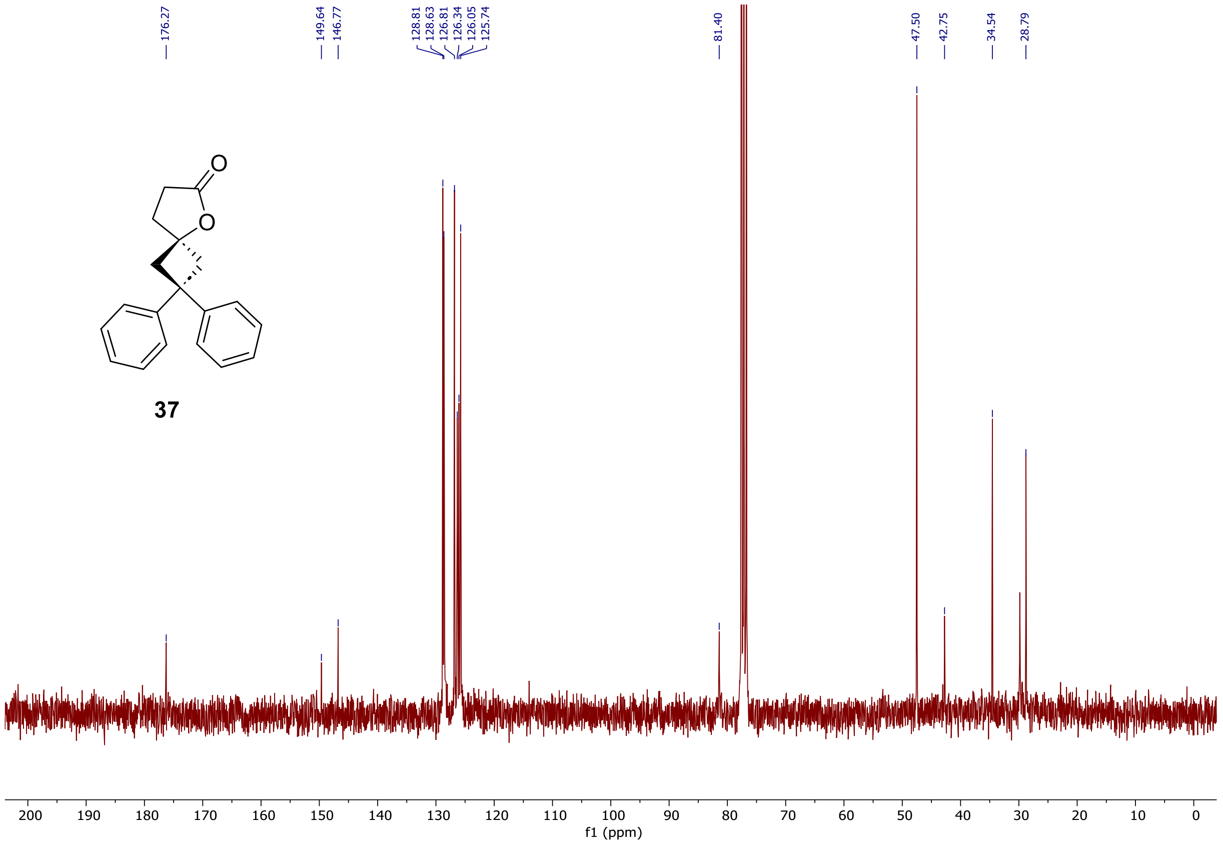
176.27
149.64
146.77
128.81
128.63
126.81
126.34
126.05
125.74
81.40
47.50
42.75
34.54
28.79
37
f1 (ppm)

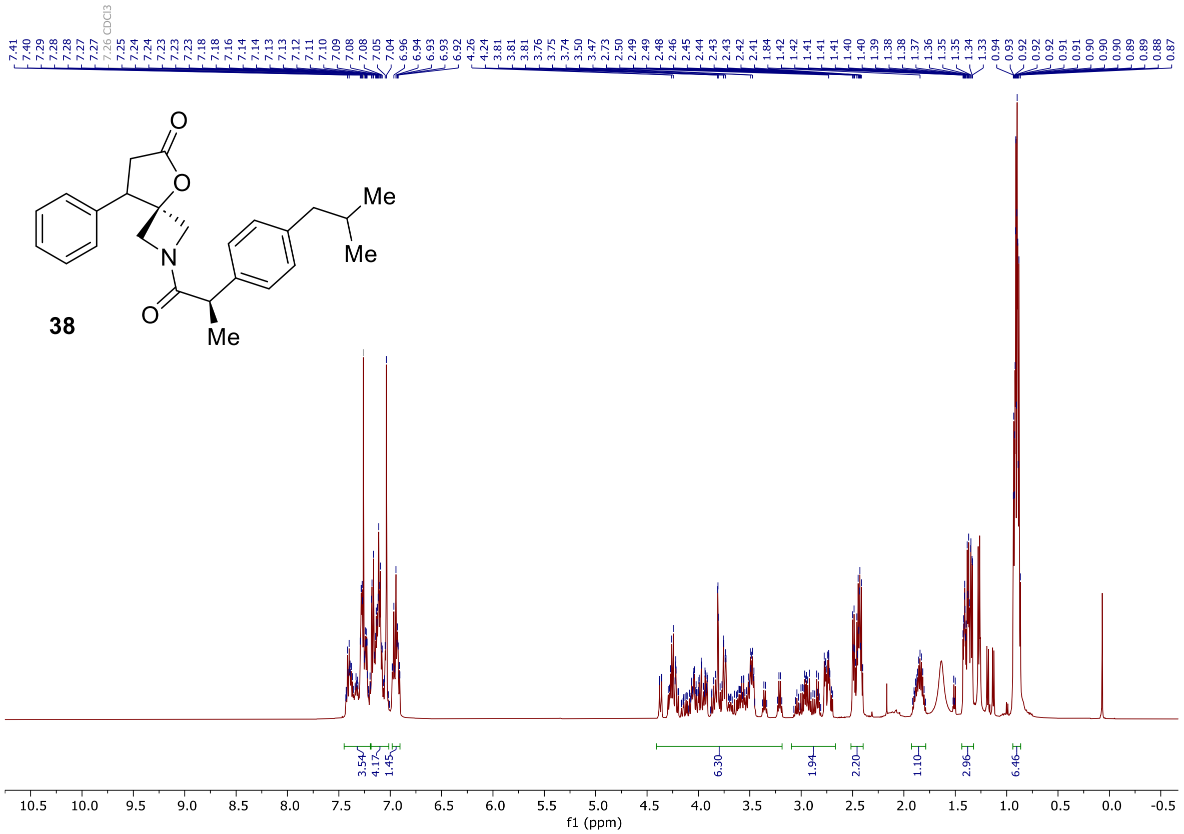
O
O
Me
Me
N
O
Me
38
CDCl3
3.54
4.17
1.45
6.30
1.94
2.20
1.10
2.96
6.46
10.5 10.0 9.5 9.0 8.5 8.0 7.5 7.0 6.5 6.0 5.5 5.0 4.5 4.0 3.5 3.0 2.5 2.0 1.5 1.0 0.5 0.0 -0.5
f1 (ppm)

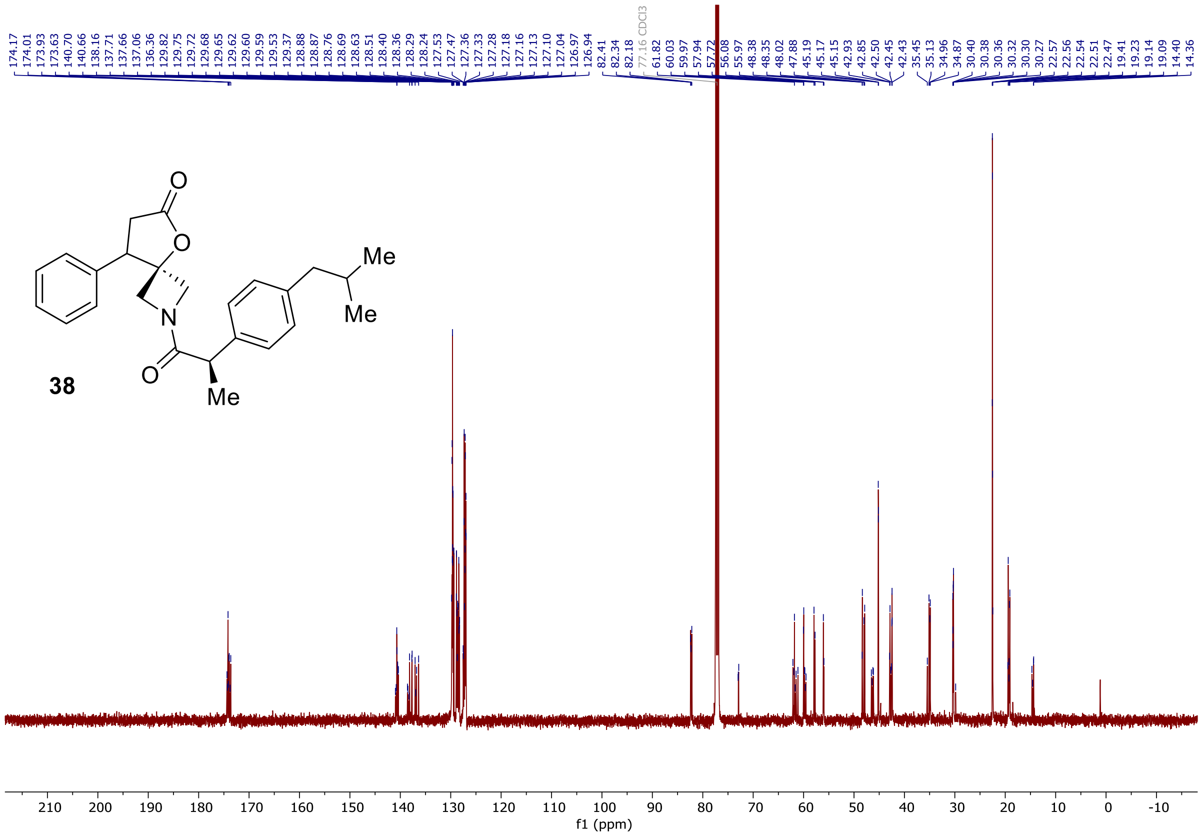
O
O
Me
Me
N
O
Me
38
CDCl3
210 200 190 180 170 160 150 140 130 120 110 100 90 80 70 60 50 40 30 20 10 0 -10
f1 (ppm)

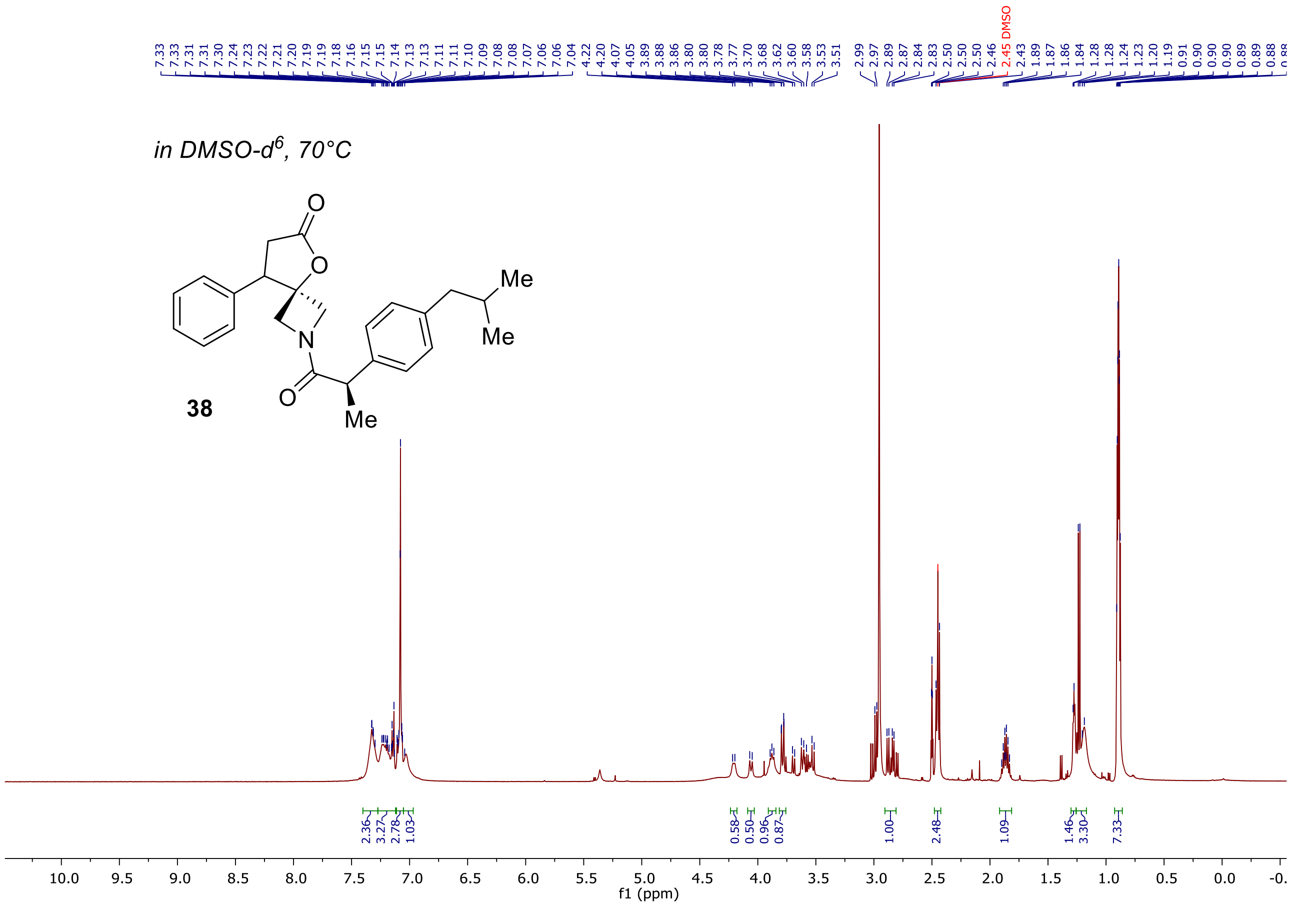
in DMSO-d6, 70°C
38
7.33 7.33 7.31 7.31 7.30 7.24 7.23 7.22 7.21 7.20 7.19 7.19 7.18 7.16 7.15 7.15 7.14 7.13 7.13 7.11 7.11 7.10 7.09 7.08 7.08 7.07 7.06 7.06 7.04 4.22 4.20 4.07 4.05 3.89 3.88 3.86 3.80 3.80 3.78 3.77 3.70 3.68 3.62 3.60 3.58 3.53 3.51 2.99 2.97 2.89 2.87 2.84 2.83 2.50 2.50 2.50 2.46 2.45 DMSO 2.43 1.89 1.87 1.86 1.84 1.28 1.28 1.24 1.23 1.20 1.19 0.91 0.90 0.90 0.90 0.89 0.89 0.88
2.36 3.27 2.78 1.03 0.58 0.50 0.96 0.87 1.00 2.48 1.09 1.46 3.30 7.33
f1 (ppm)

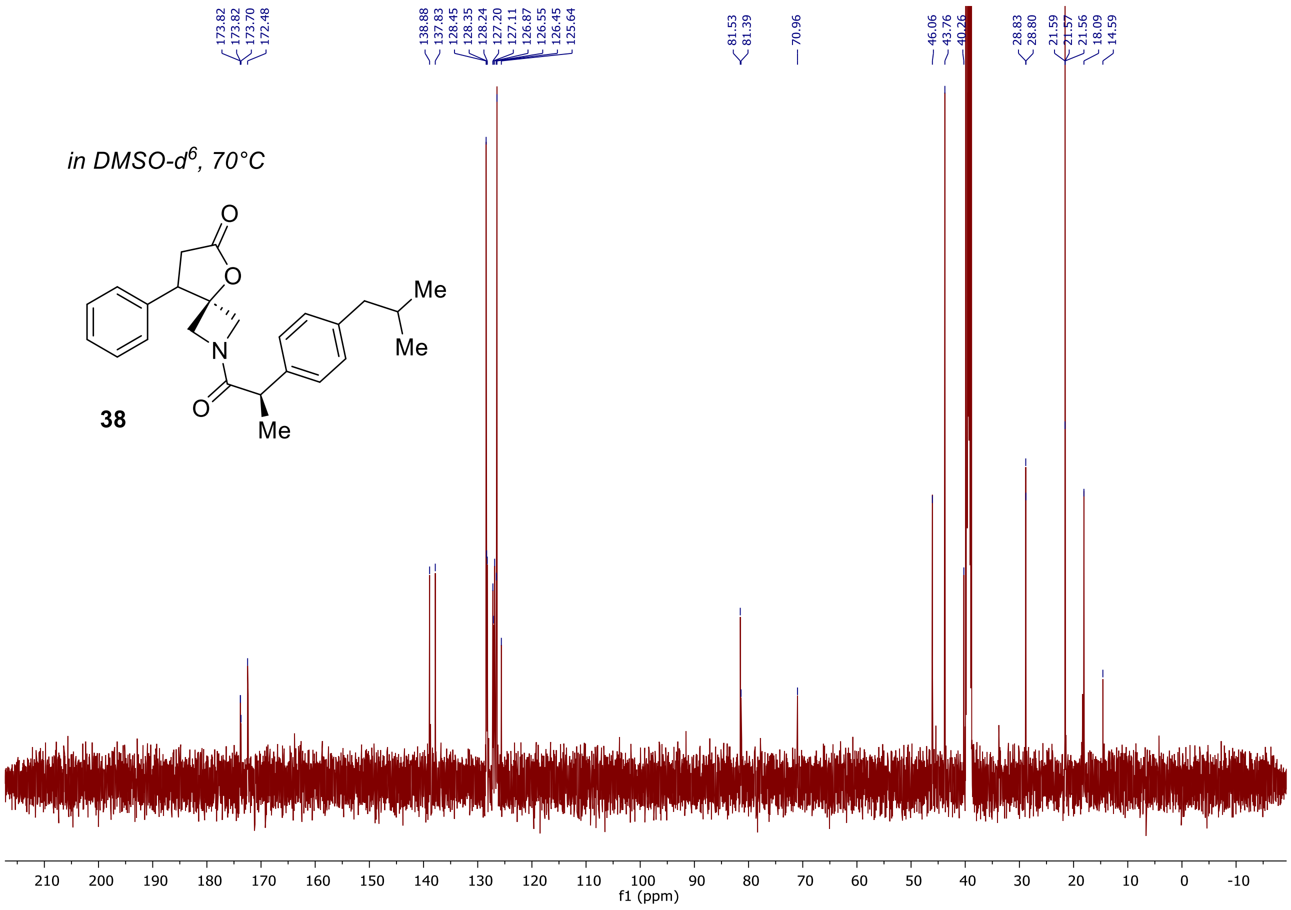
173.82 173.82 173.70 172.48 138.88 137.83 128.45 128.35 128.24 127.20 127.11 126.87 126.55 126.45 125.64 81.53 81.39 70.96 46.06 43.76 40.26 28.83 28.80 21.59 21.57 21.56 18.09 14.59
in DMSO-d6, 70°C
38
f1 (ppm)

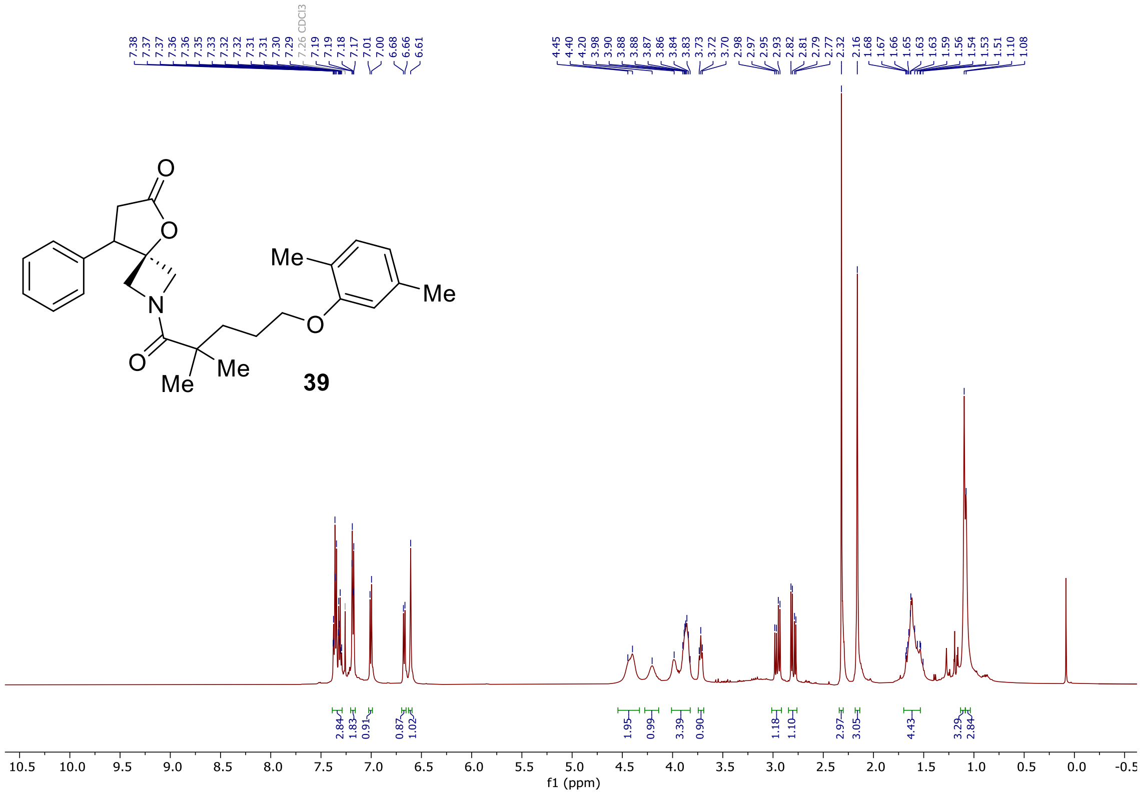
7.38 7.37 7.37 7.36 7.36 7.35 7.33 7.32 7.32 7.31 7.31 7.30 7.29 7.26 CDCl3 7.19 7.18 7.17 7.01 7.00 6.68 6.66 6.61
4.45 4.40 4.20 3.98 3.90 3.88 3.88 3.87 3.86 3.84 3.83 3.73 3.72 3.70 2.98 2.97 2.95 2.93 2.82 2.81 2.79 2.77 2.32 2.16 1.68 1.67 1.66 1.65 1.63 1.63 1.59 1.56 1.54 1.53 1.51 1.10 1.08
Me
Me
O
O
N
O
O
Me
Me
39
2.84 1.83 0.91 0.87 1.02 1.95 0.99 3.39 0.90 1.18 1.10 2.97 3.05 4.43 3.29 2.84
10.5 10.0 9.5 9.0 8.5 8.0 7.5 7.0 6.5 6.0 5.5 5.0 4.5 4.0 3.5 3.0 2.5 2.0 1.5 1.0 0.5 0.0 -0.5
f1 (ppm)

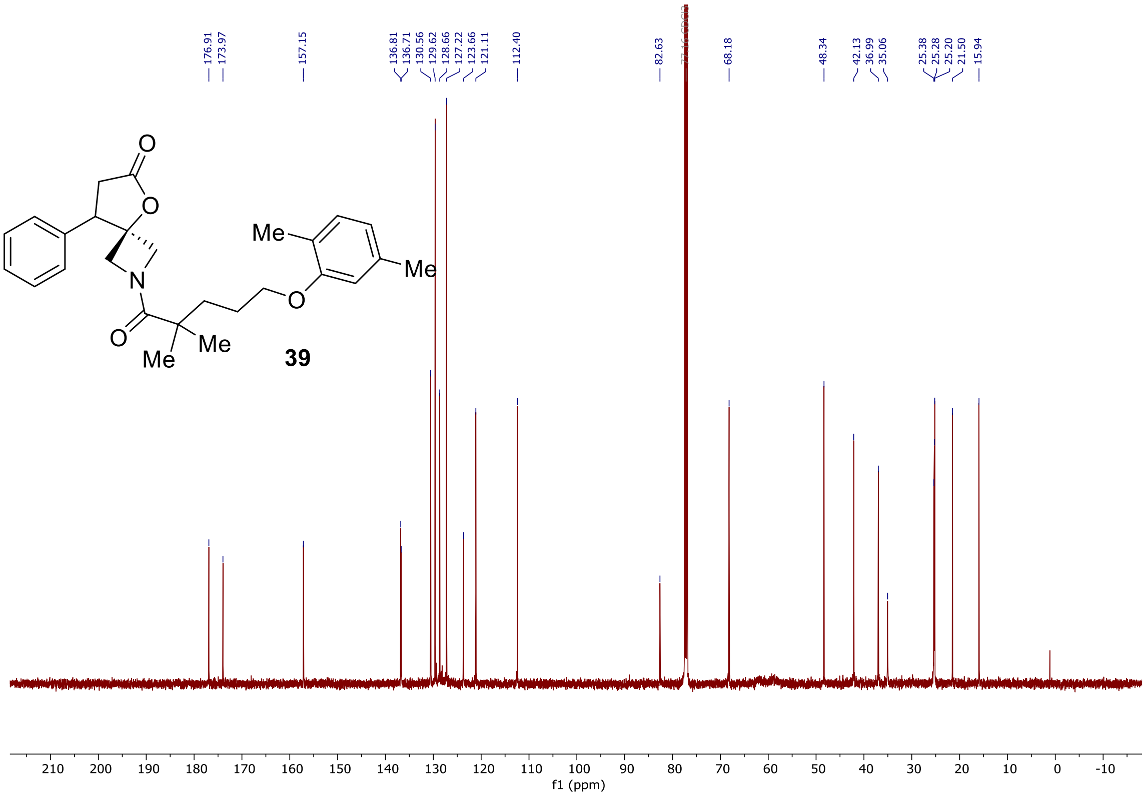
176.91 173.97 157.15 136.81 136.71 130.56 129.62 128.66 127.22 123.66 121.11 112.40 82.63 68.18 48.34 42.13 36.99 35.06 25.38 25.28 25.20 21.50 15.94
Me
Me
O
O
N
O
O
Me
Me
39
210 200 190 180 170 160 150 140 130 120 110 100 90 80 70 60 50 40 30 20 10 0 -10
f1 (ppm)

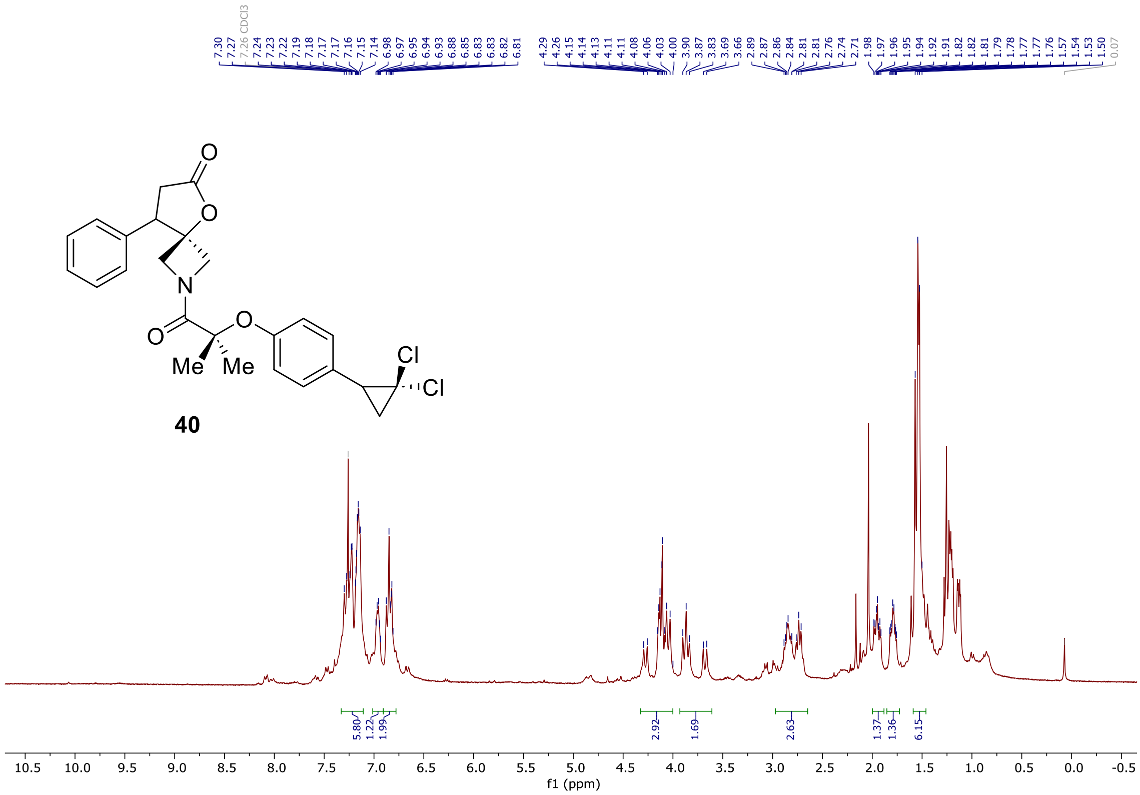
7.30 7.27 7.26 CDCl3 7.24 7.23 7.22 7.19 7.18 7.17 7.17 7.16 7.15 7.14 6.98 6.97 6.95 6.94 6.93 6.88 6.85 6.83 6.83 6.82 6.81
4.29 4.26 4.15 4.14 4.13 4.11 4.11 4.08 4.06 4.03 4.00 3.90 3.87 3.83 3.69 3.66 2.89 2.87 2.86 2.84 2.81 2.76 2.74 2.71
1.98 1.97 1.96 1.95 1.94 1.92 1.91 1.82 1.81 1.79 1.78 1.77 1.76 1.57 1.54 1.53 1.50 0.07
O
O
N
O
O
Me
Me
Cl
Cl
40
5.80 1.22 1.99 2.92 1.69 2.63 1.37 1.36 6.15
10.5 10.0 9.5 9.0 8.5 8.0 7.5 7.0 6.5 6.0 5.5 5.0 4.5 4.0 3.5 3.0 2.5 2.0 1.5 1.0 0.5 0.0 -0.5
f1 (ppm)

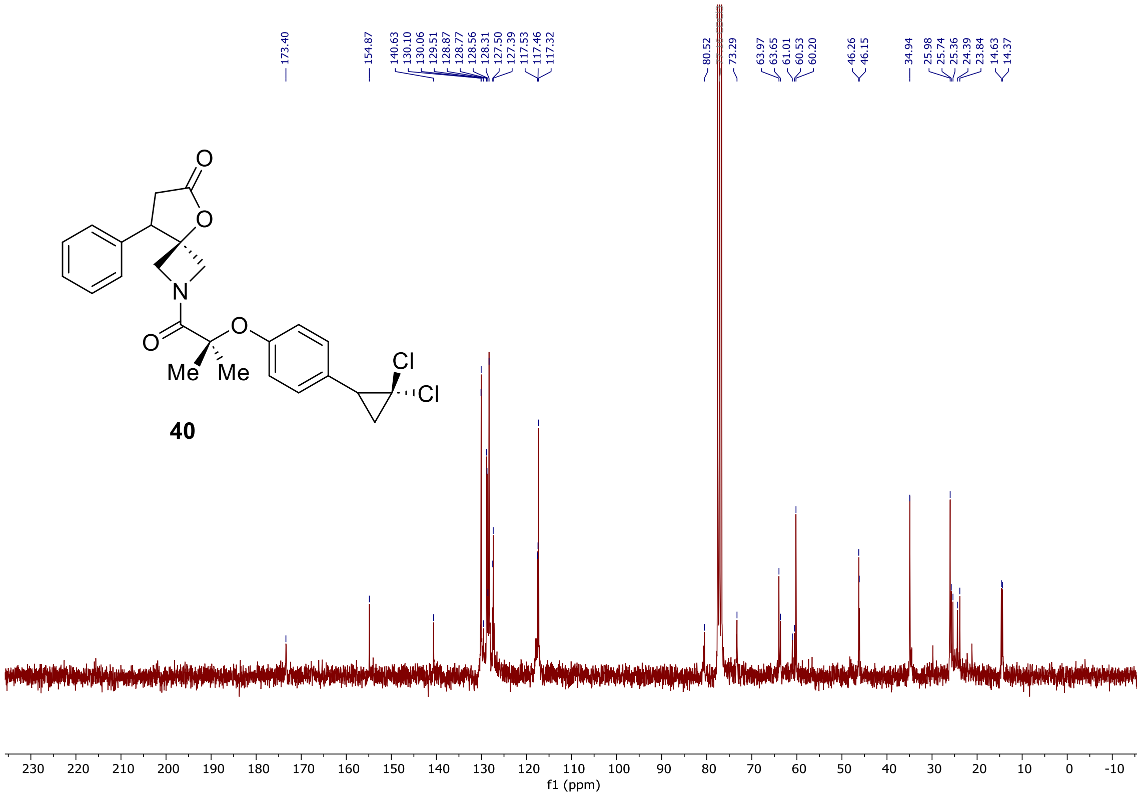
173.40
154.87
140.63 130.10 130.06 129.51 128.87 128.77 128.56 128.31 127.50 127.39 117.53 117.46 117.32
80.52
73.29
63.97 63.65 61.01 60.53 60.20
46.26 46.15
34.94
25.98 25.74 25.36 24.39 23.84 14.63 14.37
O
O
N
O
O
Me
Me
Cl
Cl
40
230 220 210 200 190 180 170 160 150 140 130 120 110 100 90 80 70 60 50 40 30 20 10 0 -10
f1 (ppm)

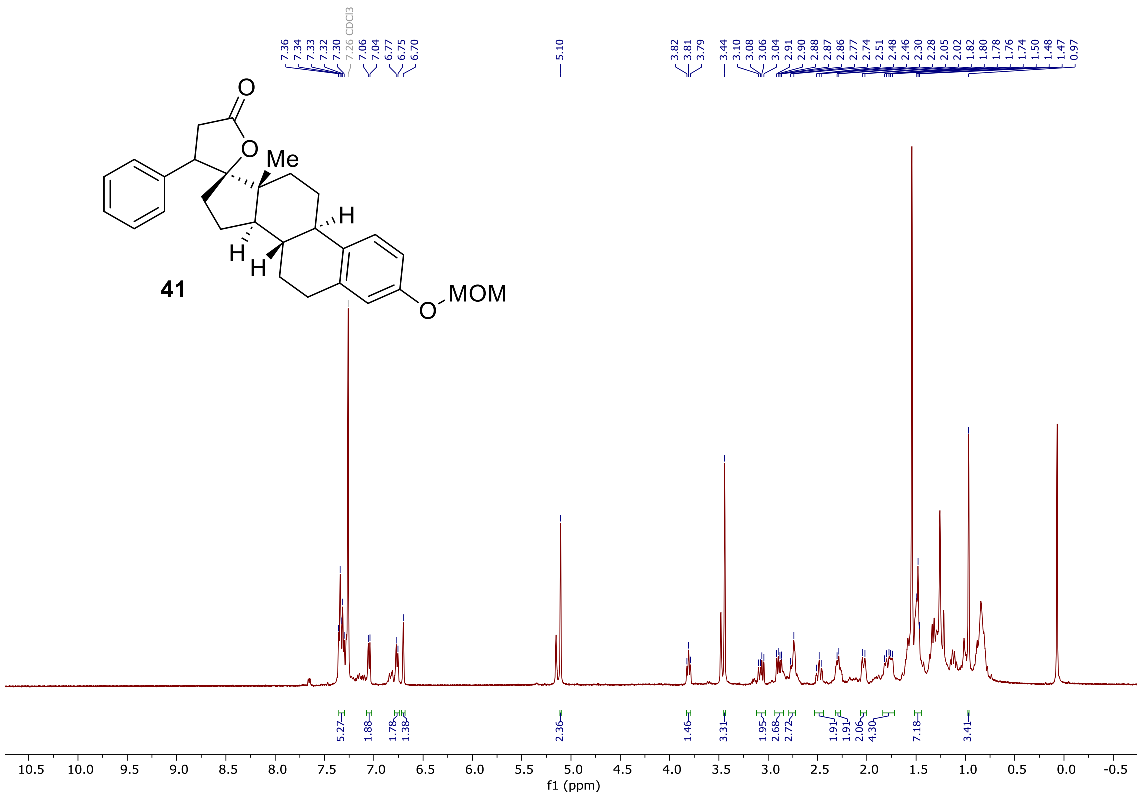
7.36 7.34 7.33 7.32 7.30 7.26 CDCl3 7.06 7.04 6.77 6.75 6.70
5.10
3.82 3.81 3.79 3.44 3.10 3.08 3.06 3.04 2.91 2.90 2.88 2.87 2.86 2.77 2.74 2.51 2.48 2.46 2.30 2.28 2.05 2.02 1.82 1.80 1.78 1.76 1.74 1.50 1.48 1.47 0.97
O
O
Me
H
H
H
MOM
O
41
5.27 1.88 1.78 1.38 2.36 1.46 3.31 1.95 2.68 2.72 1.91 1.91 2.06 4.30 7.18 3.41
10.5 10.0 9.5 9.0 8.5 8.0 7.5 7.0 6.5 6.0 5.5 5.0 4.5 4.0 3.5 3.0 2.5 2.0 1.5 1.0 0.5 0.0 -0.5
f1 (ppm)

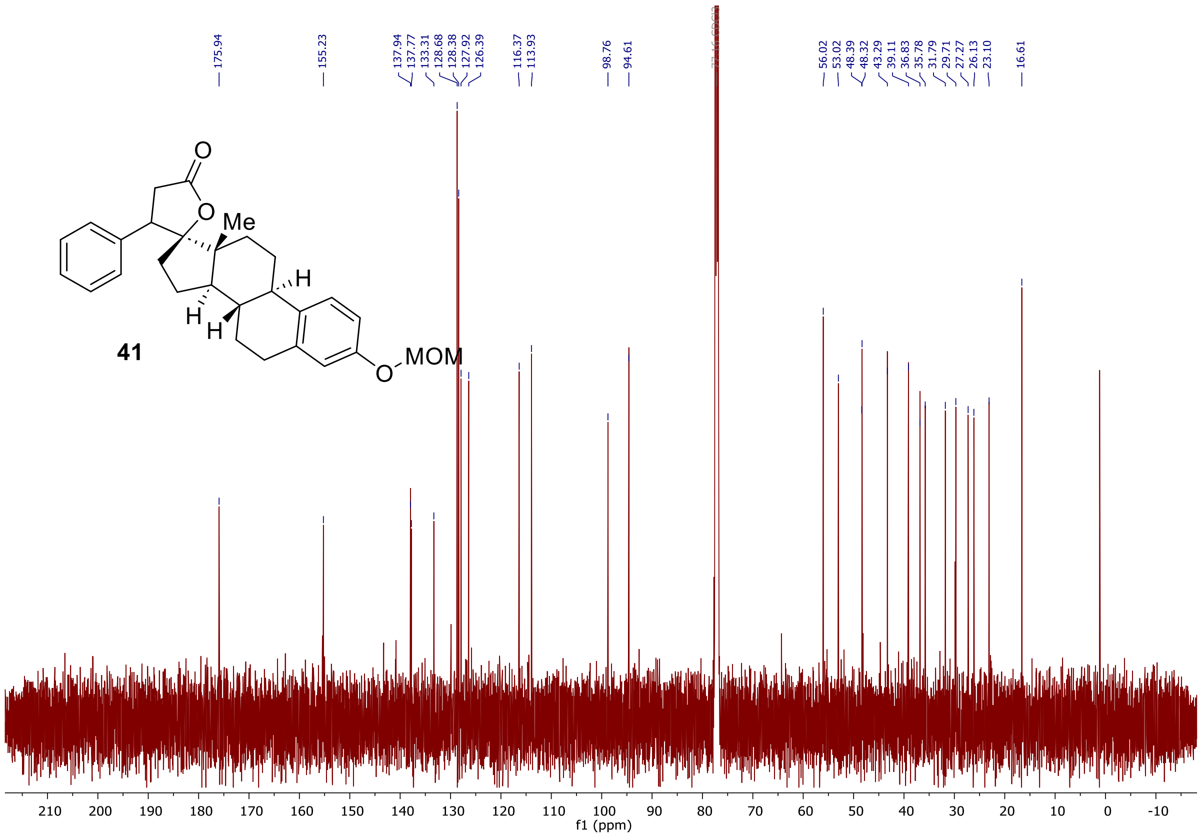
175.94
155.23
137.94 137.77 133.31 128.68 128.38 127.92 126.39
116.37 113.93
98.76 94.61
56.02 53.02 48.39 48.32 43.29 39.11 36.83 35.78 31.79 29.71 27.27 26.13 23.10
16.61
O
O
Me
H
H
H
MOM
O
41
210 200 190 180 170 160 150 140 130 120 110 100 90 80 70 60 50 40 30 20 10 0 -10
f1 (ppm)

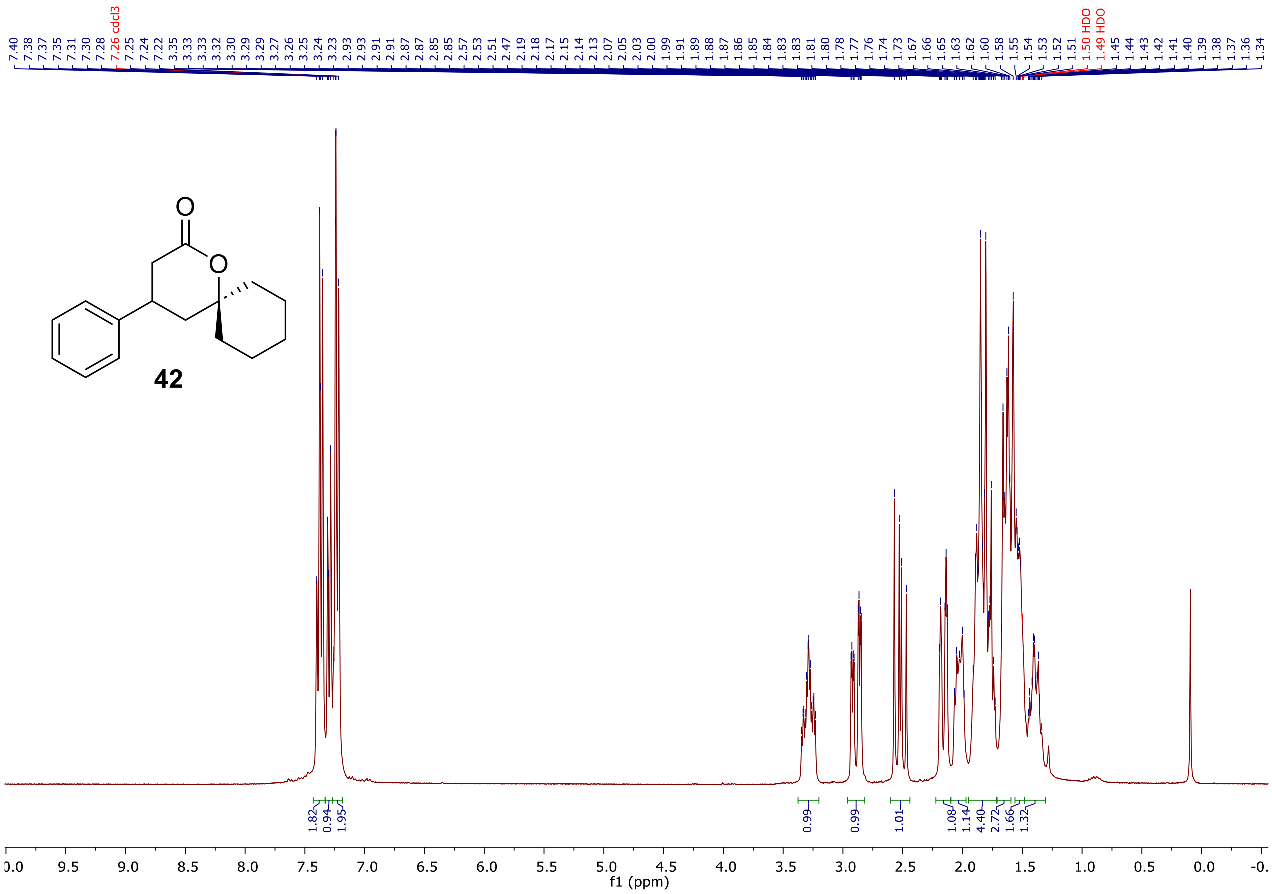

7.40 7.38 7.37 7.35 7.31 7.30 7.28 7.26 cdcl3 7.25 7.24 7.22 3.35 3.33 3.33 3.32 3.30 3.29 3.29 3.27 3.26 3.25 3.24 3.23 2.93 2.93 2.93 2.91 2.91 2.91 2.87 2.87 2.85 2.85 2.57 2.53 2.51 2.47 2.19 2.18 2.17 2.15 2.14 2.13 2.07 2.05 2.03 2.00 1.99 1.91 1.89 1.88 1.87 1.86 1.85 1.84 1.83 1.81 1.80 1.78 1.77 1.76 1.74 1.73 1.67 1.66 1.65 1.63 1.62 1.60 1.58 1.55 1.54 1.53 1.52 1.51 1.50 HDO 1.49 HDO 1.45 1.44 1.43 1.42 1.41 1.40 1.39 1.38 1.37 1.36 1.34
42
1.82 0.94 1.95 0.99 0.99 1.01 1.08 1.14 4.40 2.72 1.66 1.32
f1 (ppm)

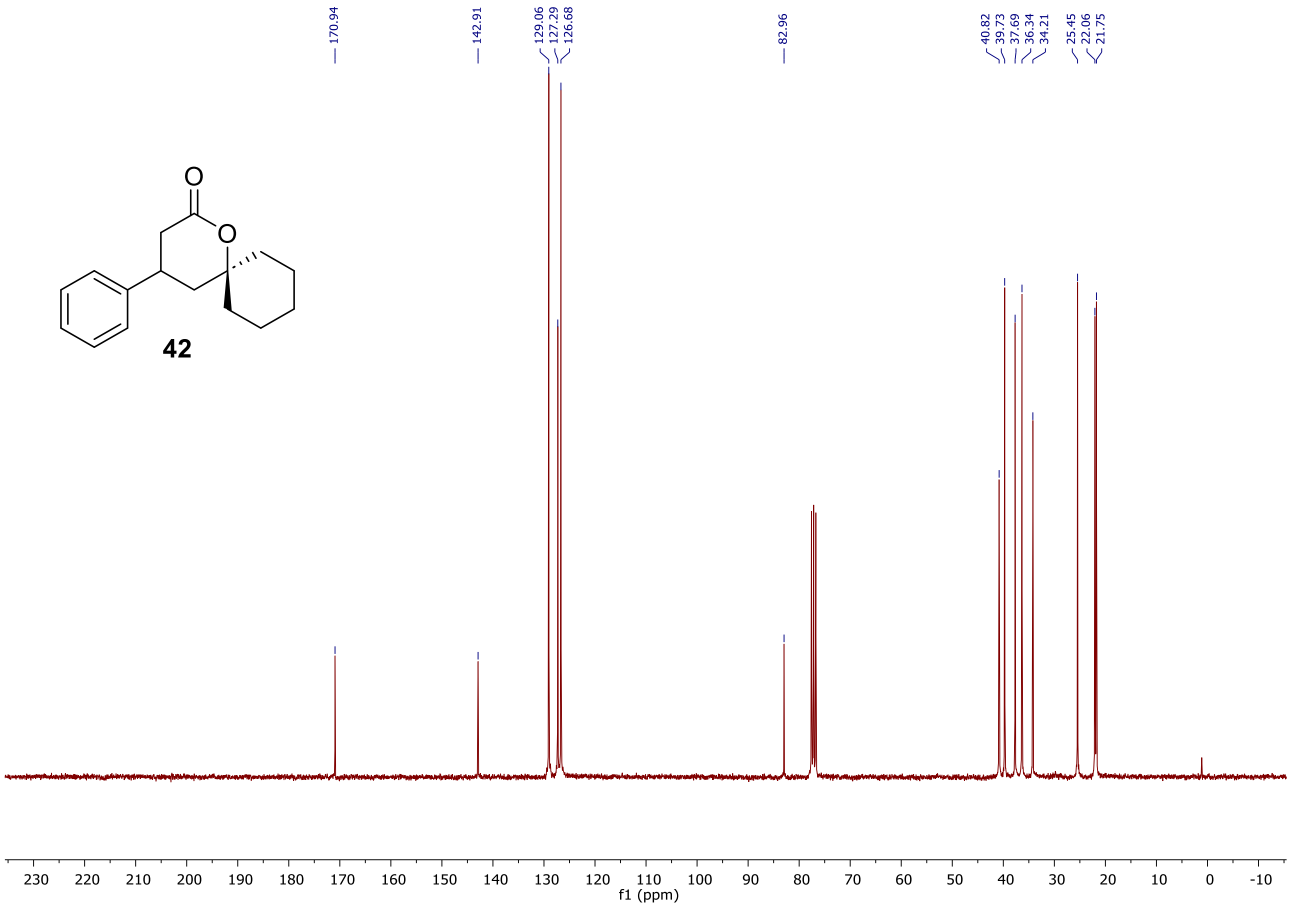

170.94 142.91 129.06 127.29 126.68 82.96 40.82 39.73 37.69 36.34 34.21 25.45 22.06 21.75
42
f1 (ppm)

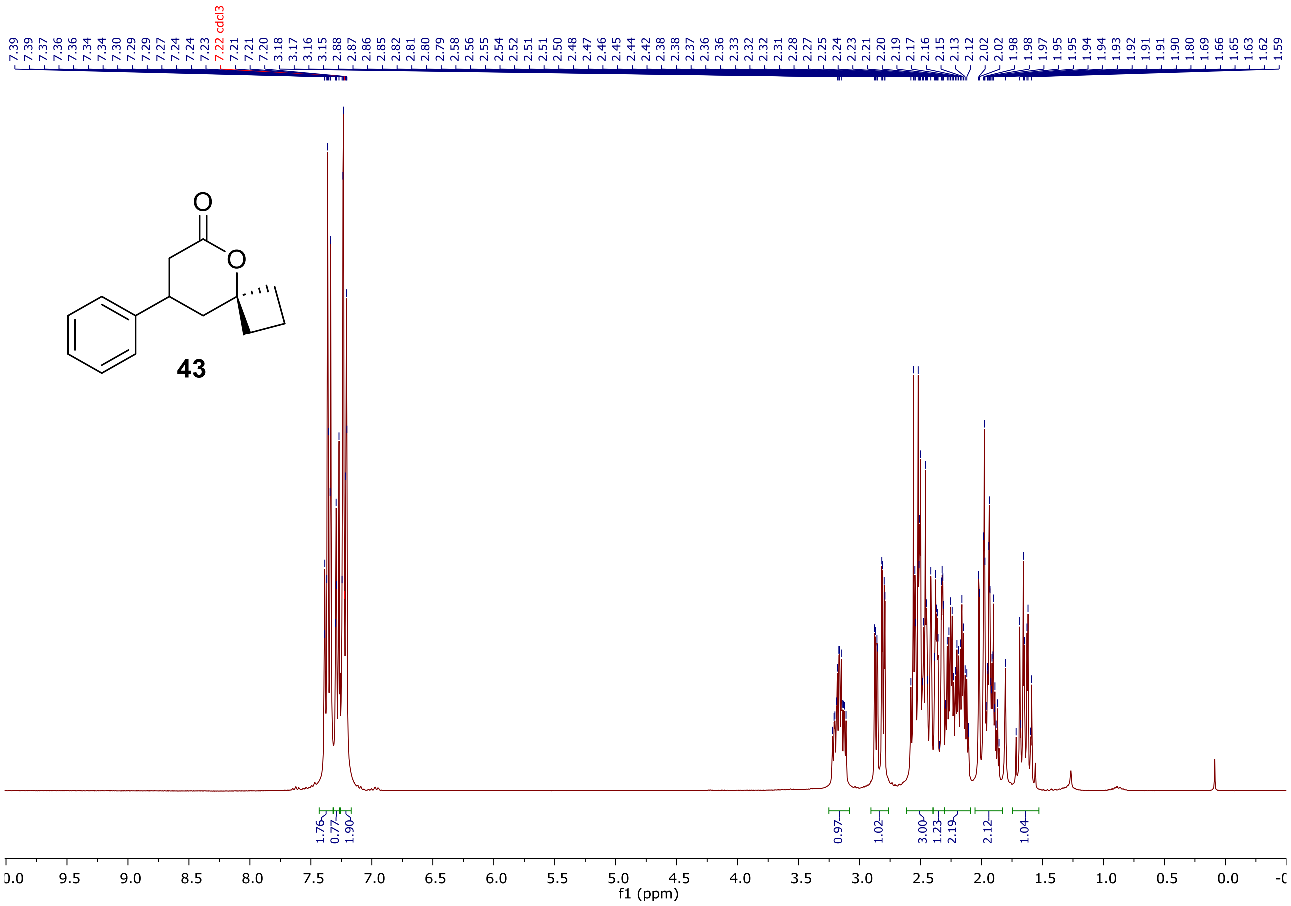
7.39 7.39 7.37 7.36 7.36 7.34 7.34 7.30 7.29 7.29 7.27 7.24 7.24 7.23 7.22 cdcl3 7.21 7.21 7.20 3.18 3.17 3.16 3.15 2.88 2.87 2.86 2.85 2.82 2.81 2.80 2.79 2.58 2.56 2.55 2.54 2.52 2.51 2.51 2.50 2.48 2.47 2.46 2.45 2.44 2.42 2.38 2.38 2.37 2.36 2.36 2.33 2.32 2.32 2.31 2.28 2.27 2.25 2.24 2.23 2.21 2.20 2.19 2.17 2.16 2.15 2.13 2.12 2.02 2.02 1.98 1.98 1.97 1.95 1.95 1.94 1.94 1.93 1.92 1.91 1.91 1.90 1.80 1.69 1.66 1.65 1.63 1.62 1.59
O
O
43
1.76
0.77
1.90
0.97
1.02
3.00
1.23
2.19
2.12
1.04
f1 (ppm)

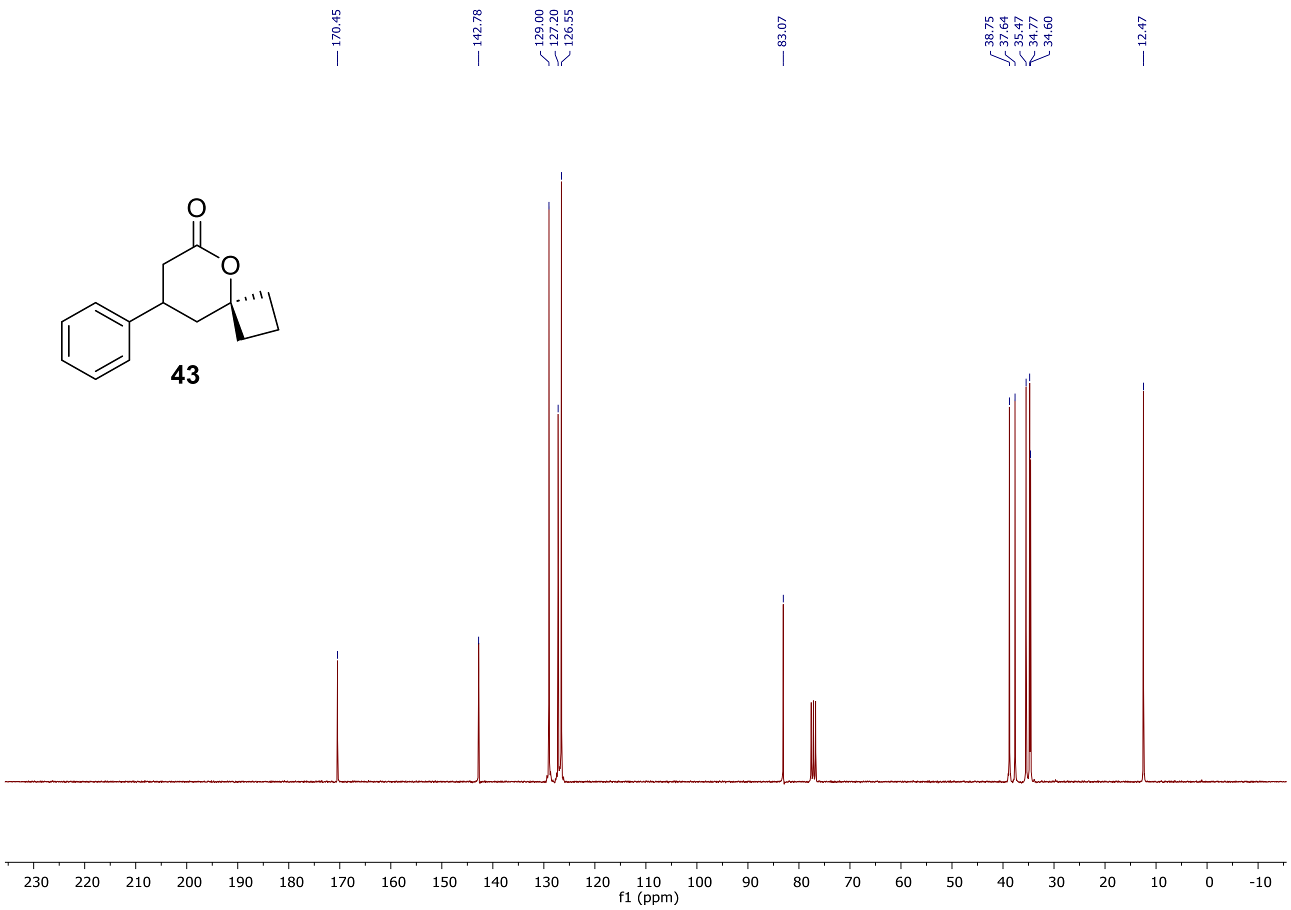
170.45
142.78
129.00
127.20
126.55
83.07
38.75
37.64
35.47
34.77
34.60
12.47
O
O
43
f1 (ppm)

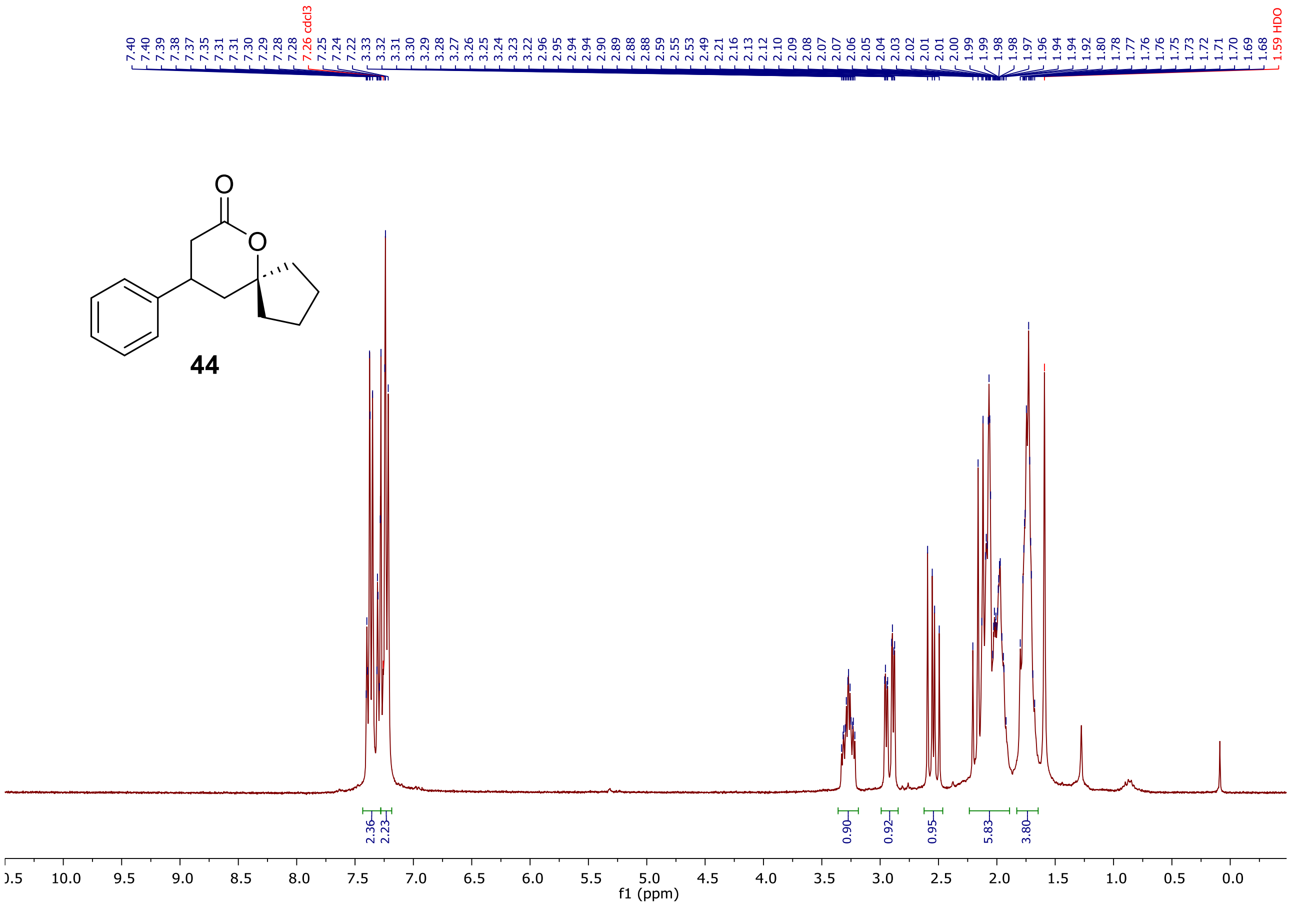
7.40
7.40
7.39
7.38
7.37
7.35
7.31
7.31
7.30
7.29
7.28
7.28
7.26 cdcl3
7.25
7.24
7.22
3.33
3.32
3.31
3.30
3.29
3.28
3.27
3.26
3.25
3.24
3.23
3.22
2.96
2.95
2.94
2.94
2.90
2.89
2.88
2.88
2.59
2.55
2.53
2.49
2.21
2.16
2.13
2.12
2.10
2.09
2.08
2.07
2.07
2.06
2.05
2.04
2.03
2.02
2.01
2.01
2.00
1.99
1.99
1.98
1.98
1.97
1.96
1.94
1.94
1.92
1.80
1.78
1.77
1.76
1.76
1.75
1.73
1.72
1.71
1.70
1.69
1.68
1.59 HDO
O
O
44
2.36
2.23
0.90
0.92
0.95
5.83
3.80
f1 (ppm)

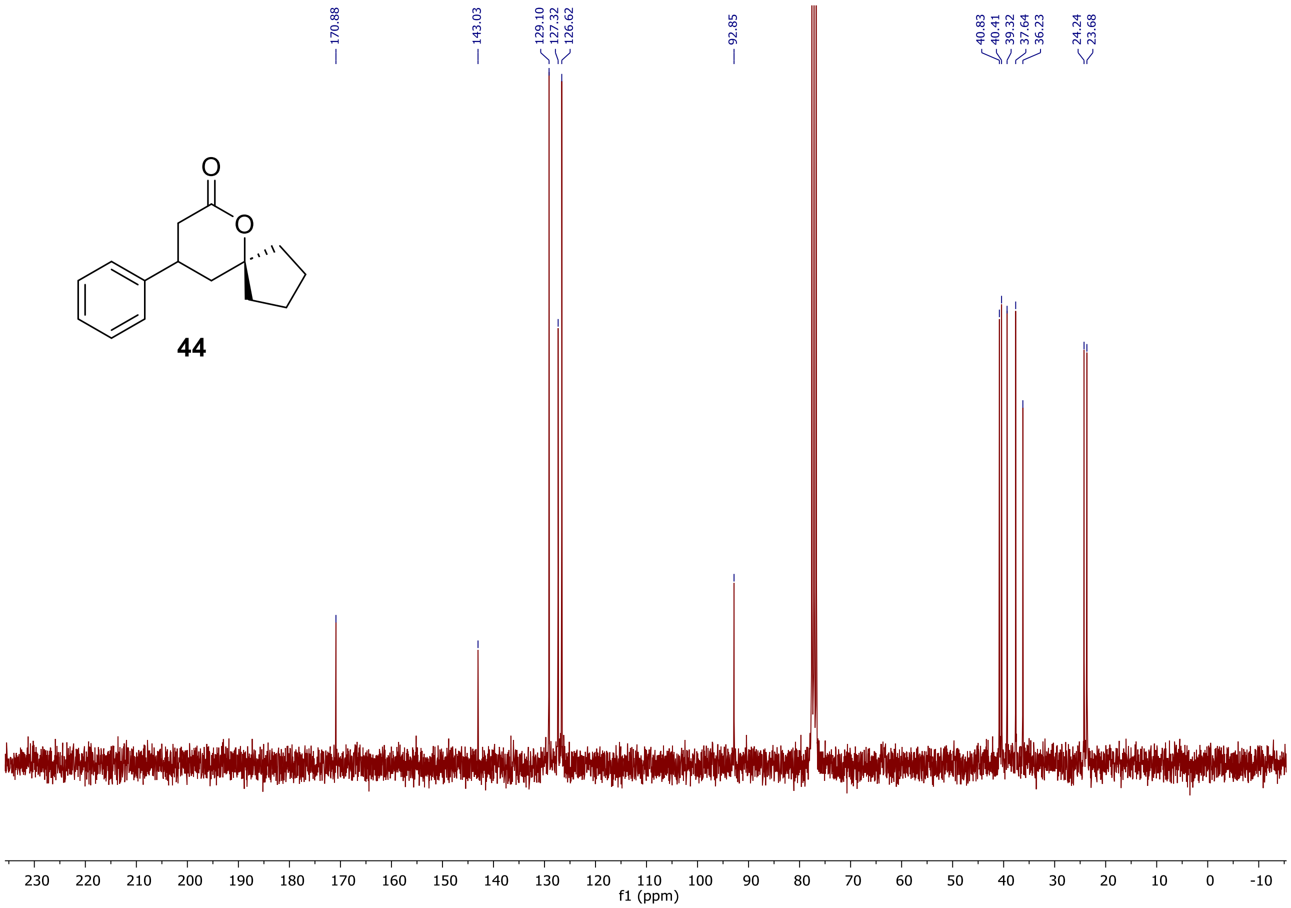
170.88
143.03
129.10
127.32
126.62
92.85
40.83
40.41
39.32
37.64
36.23
24.24
23.68
O
O
44
f1 (ppm)

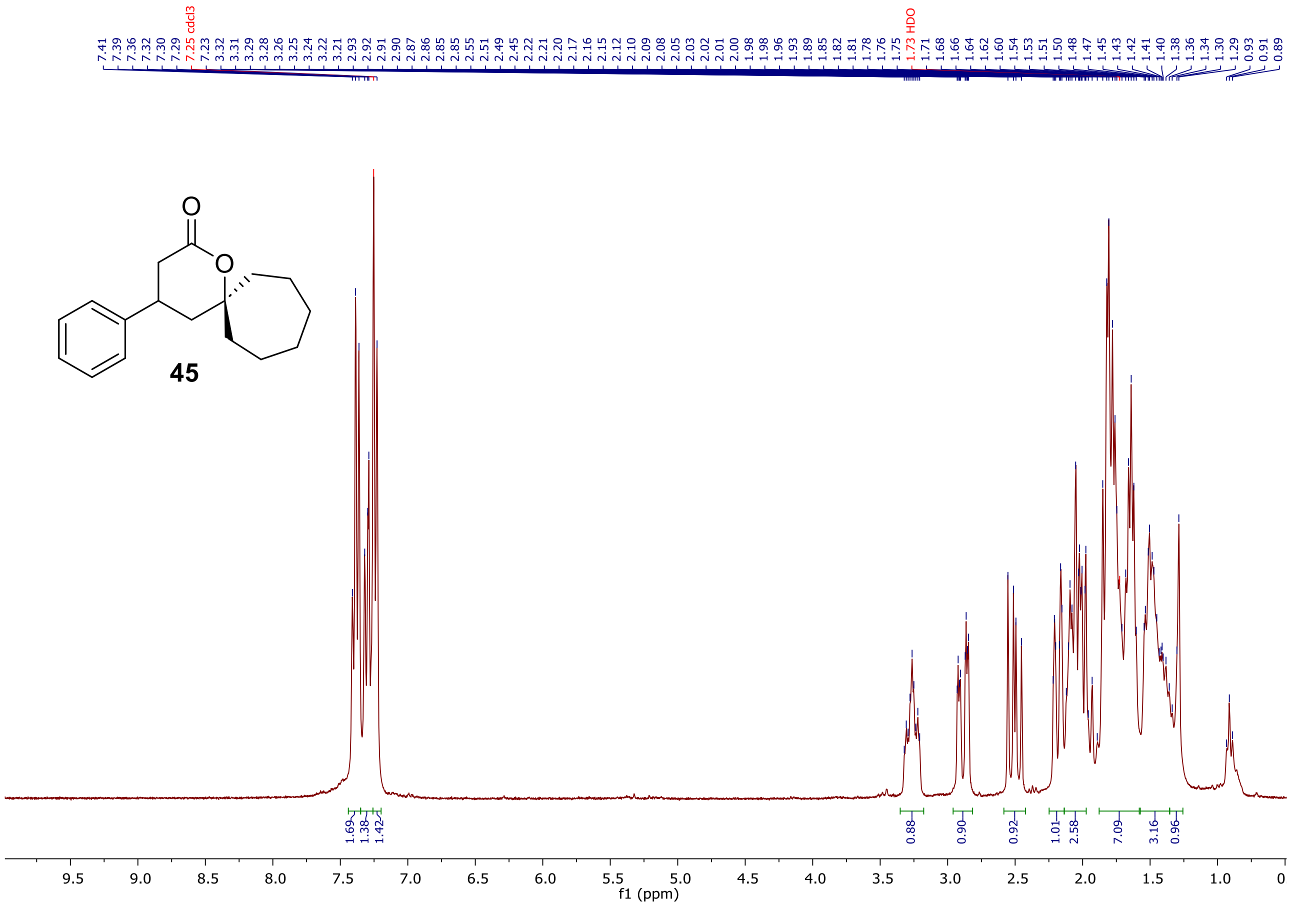
7.41 7.39 7.36 7.32 7.30 7.29 7.25 cdcl3 7.23 3.32 3.31 3.29 3.28 3.26 3.25 3.24 3.22 3.21 2.93 2.92 2.91 2.90 2.87 2.86 2.85 2.55 2.51 2.49 2.45 2.22 2.21 2.20 2.17 2.16 2.15 2.12 2.10 2.09 2.08 2.05 2.03 2.02 2.01 2.00 1.98 1.98 1.96 1.93 1.89 1.85 1.82 1.81 1.78 1.76 1.75 1.73 HDO 1.71 1.68 1.66 1.64 1.62 1.60 1.54 1.53 1.51 1.50 1.48 1.47 1.45 1.43 1.42 1.41 1.40 1.38 1.36 1.34 1.30 1.29 0.93 0.91 0.89
O
O
45
1.69
1.38
1.42
0.88
0.90
0.92
1.01
2.58
7.09
3.16
0.96
9.5 9.0 8.5 8.0 7.5 7.0 6.5 6.0 5.5 5.0 4.5 4.0 3.5 3.0 2.5 2.0 1.5 1.0 0
f1 (ppm)

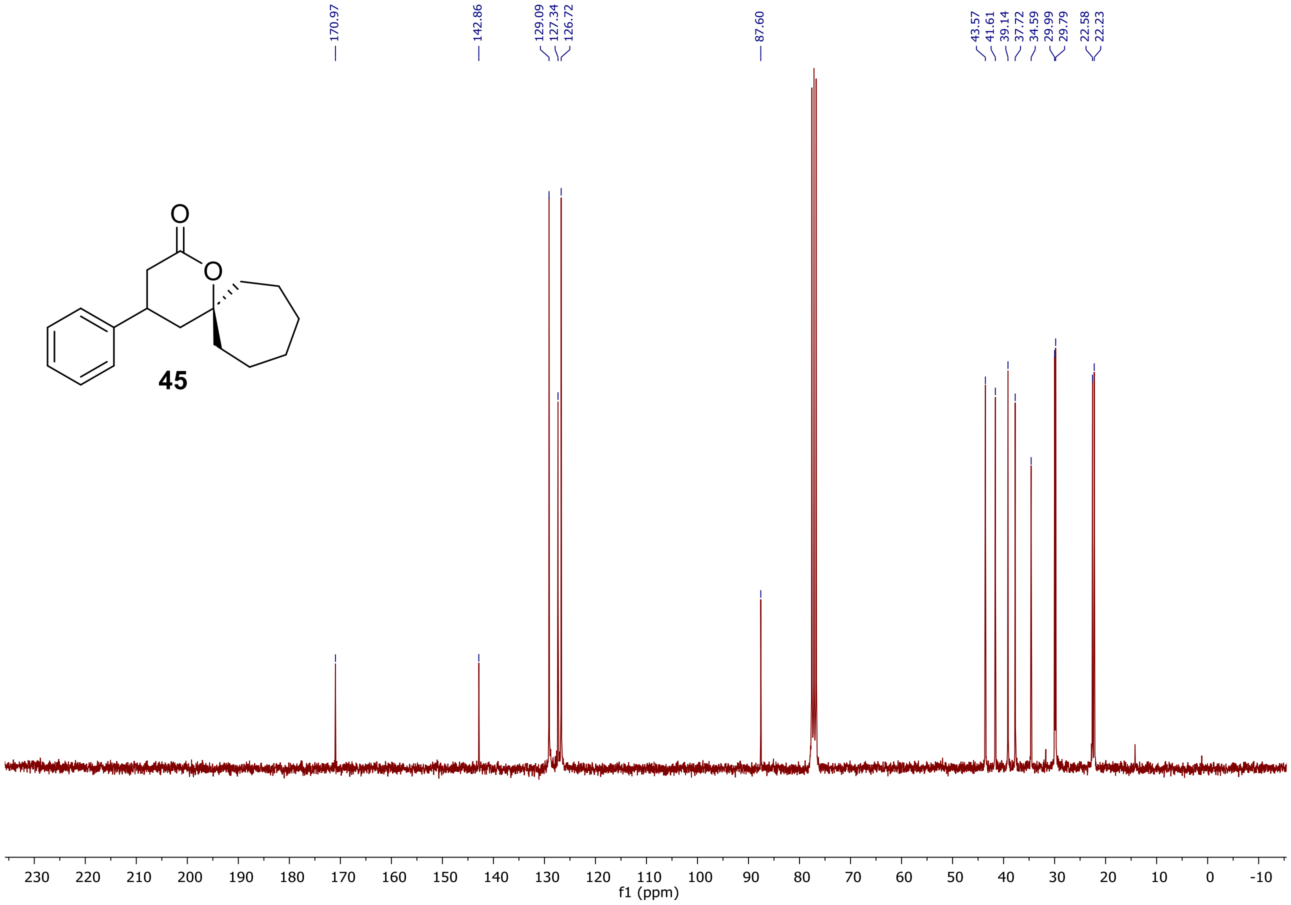
170.97
142.86
129.09 127.34 126.72
87.60
43.57 41.61 39.14 37.72 34.59 29.99 29.79 22.58 22.23
O
O
45
230 220 210 200 190 180 170 160 150 140 130 120 110 100 90 80 70 60 50 40 30 20 10 0 -10
f1 (ppm)

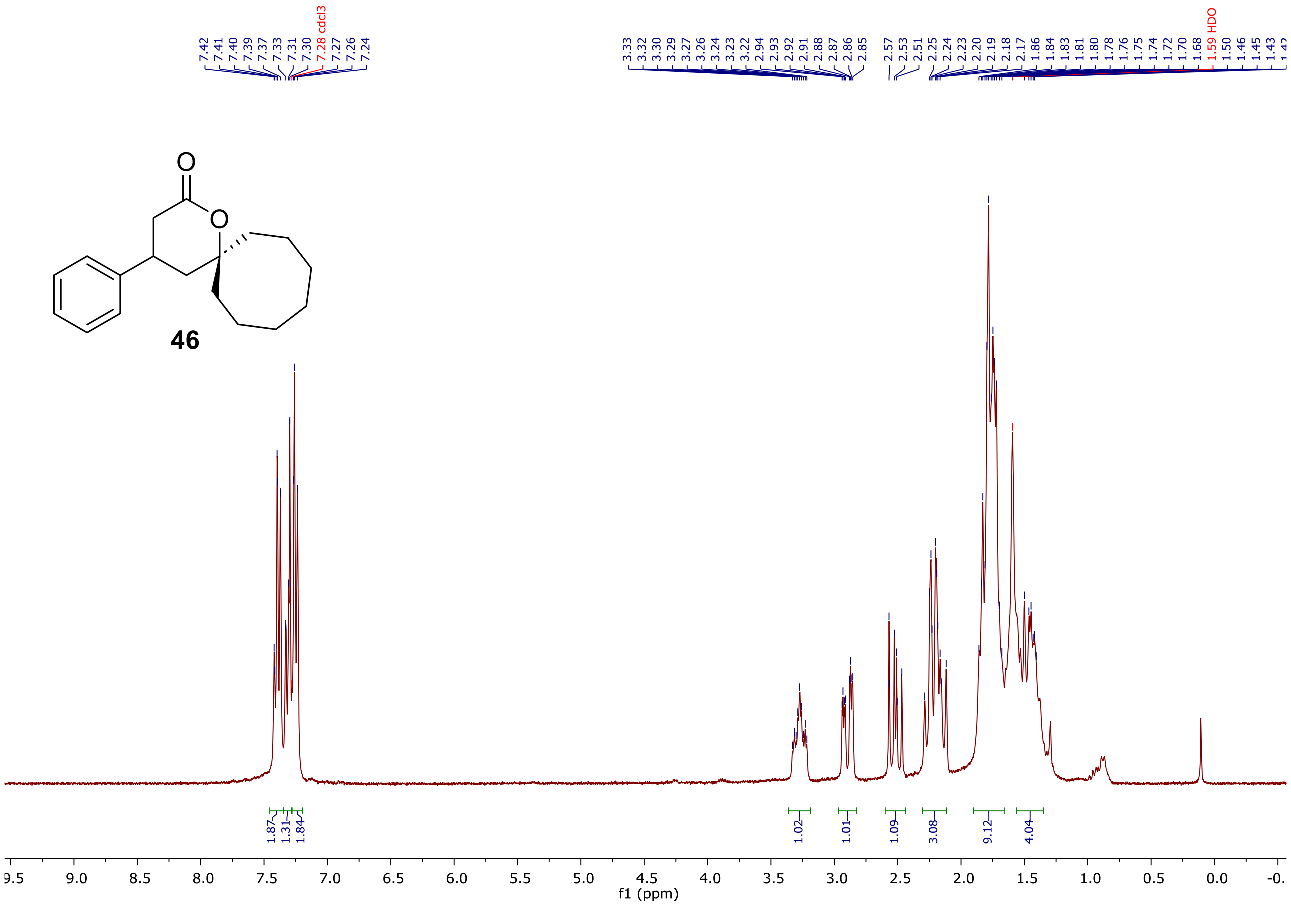
7.42 7.41 7.40 7.39 7.37 7.33 7.31 7.30 7.28 cdcl3 7.27 7.26 7.24
3.33 3.32 3.30 3.29 3.27 3.26 3.24 3.23 3.22 2.94 2.93 2.92 2.91 2.88 2.87 2.86 2.85
2.57 2.53 2.51 2.25 2.24 2.23 2.20 2.19 2.18 2.17 1.86 1.84 1.83 1.81 1.80 1.78 1.76 1.75 1.74 1.72 1.70 1.68 1.59 HDO 1.50 1.46 1.45 1.43
O
O
46
1.87 1.31 1.84
1.02 1.01 1.09 3.08 9.12 4.04
9.5 9.0 8.5 8.0 7.5 7.0 6.5 6.0 5.5 5.0 4.5 4.0 3.5 3.0 2.5 2.0 1.5 1.0 0.5 0.0
f1 (ppm)

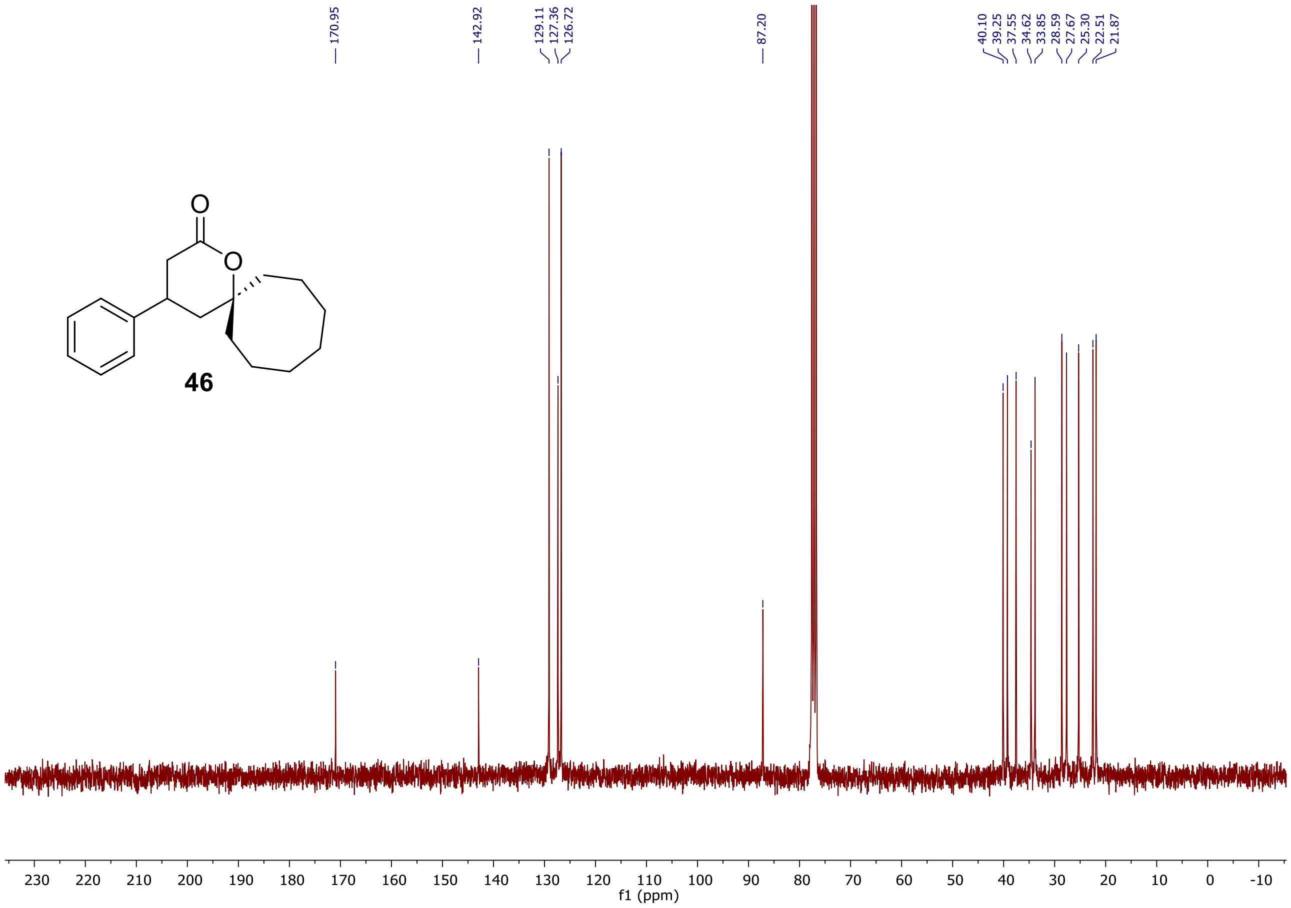
170.95
142.92
129.11 127.36 126.72
87.20
40.10 39.25 37.55 34.62 33.85 28.59 27.67 25.30 22.51 21.87
O
O
46
230 220 210 200 190 180 170 160 150 140 130 120 110 100 90 80 70 60 50 40 30 20 10 0 -10
f1 (ppm)

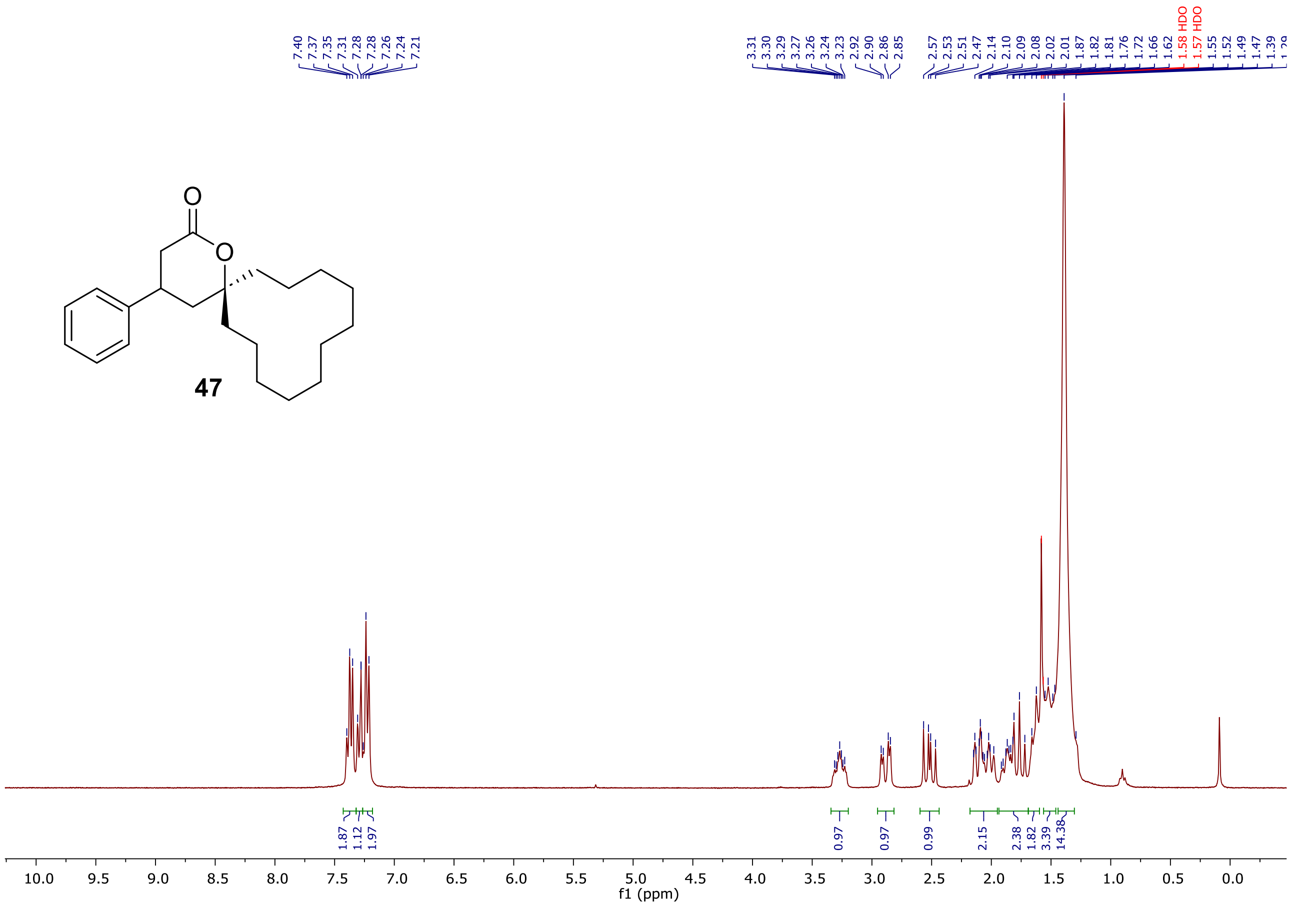
7.40
7.37
7.35
7.31
7.28
7.28
7.26
7.24
7.21
3.31
3.30
3.29
3.27
3.26
3.24
3.23
2.92
2.90
2.86
2.85
2.57
2.53
2.51
2.47
2.14
2.10
2.09
2.08
2.02
2.01
1.87
1.82
1.81
1.76
1.72
1.66
1.62
1.58 HDO
1.57 HDO
1.55
1.52
1.49
1.47
1.39
O
O
O
47
1.87
1.12
1.97
0.97
0.97
0.99
2.15
2.38
1.82
3.39
14.38
10.0
9.5
9.0
8.5
8.0
7.5
7.0
6.5
6.0
5.5
5.0
4.5
4.0
3.5
3.0
2.5
2.0
1.5
1.0
0.5
0.0
f1 (ppm)

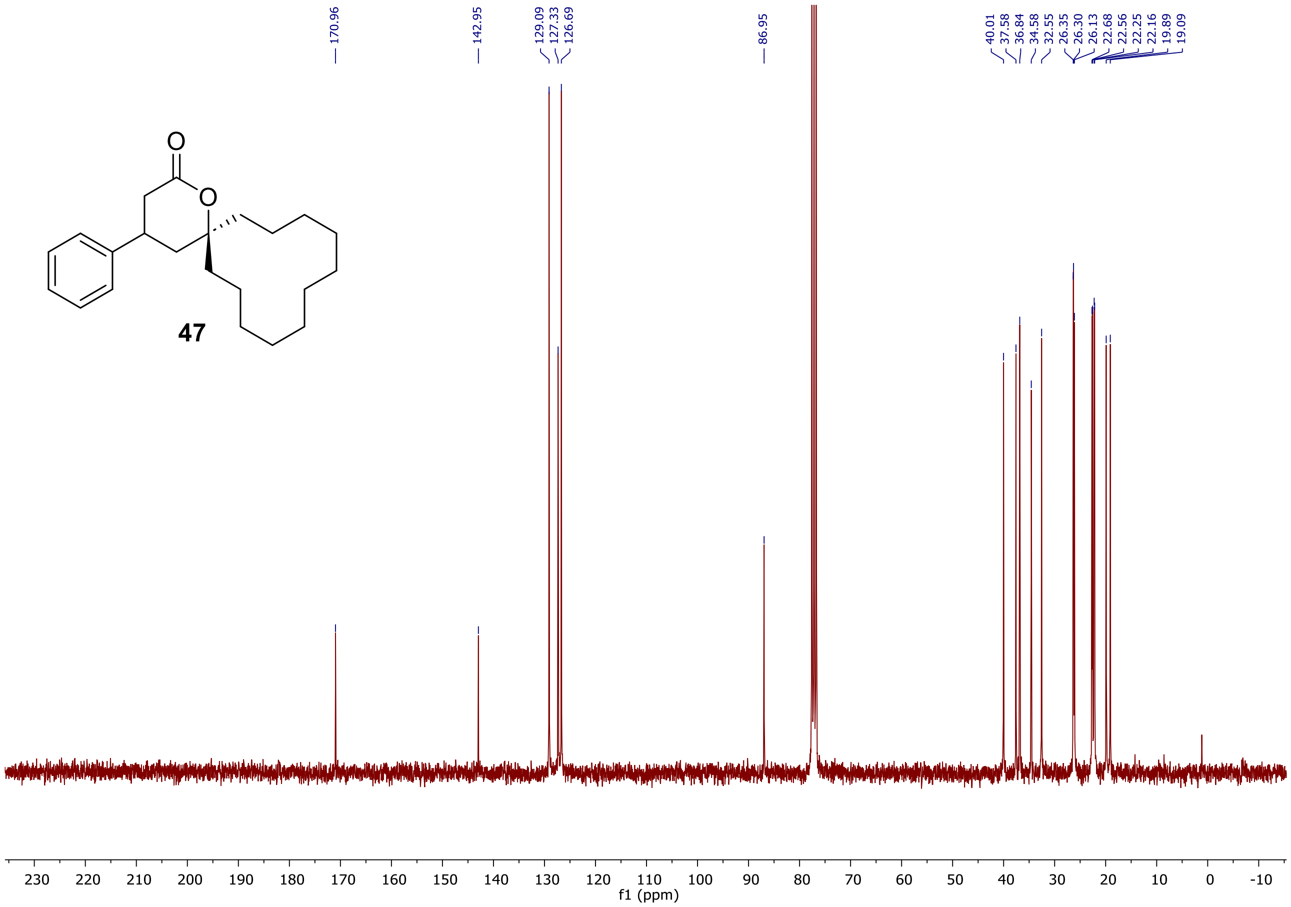
170.96
142.95
129.09
127.33
126.69
86.95
40.01
37.58
36.84
34.58
32.55
26.35
26.30
26.13
22.68
22.56
22.25
22.16
19.89
19.09
O
O
47
230
220
210
200
190
180
170
160
150
140
130
120
110
100
90
80
70
60
50
40
30
20
10
0
-10
f1 (ppm)

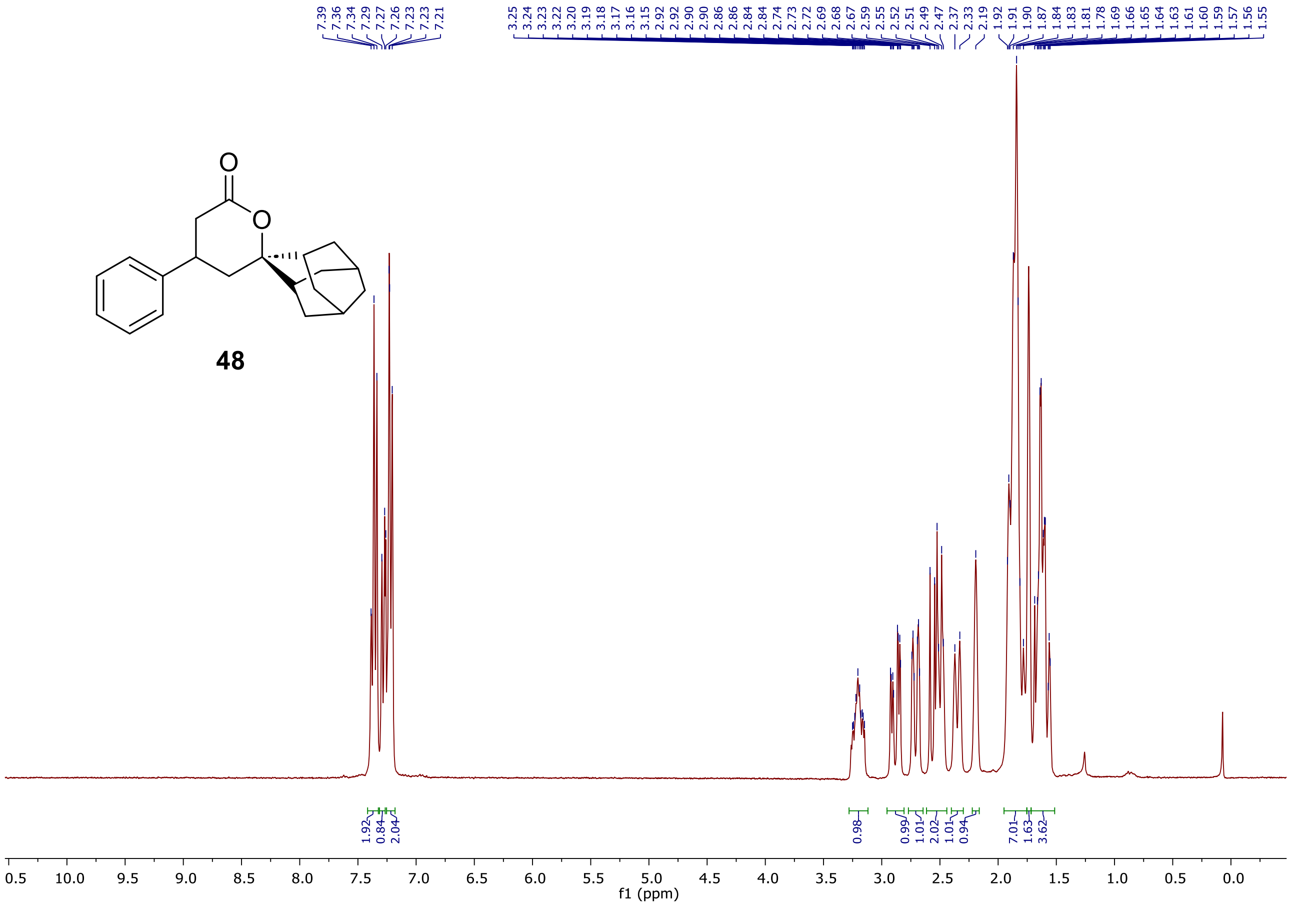
7.39 7.36 7.34 7.29 7.27 7.26 7.23 7.23 7.21
3.25 3.24 3.23 3.22 3.20 3.19 3.18 3.17 3.16 3.15 2.92 2.92 2.90 2.90 2.86 2.86 2.84 2.84 2.74 2.73 2.72 2.69 2.68 2.67 2.59 2.55 2.52 2.51 2.49 2.47 2.37 2.33 2.19 1.92 1.91 1.90 1.87 1.84 1.83 1.81 1.78 1.69 1.66 1.65 1.64 1.63 1.61 1.60 1.59 1.57 1.56 1.55
O
O
48
1.92 0.84 2.04
0.98 0.99 1.01 2.02 1.01 0.94 7.01 1.63 3.62
0.5 10.0 9.5 9.0 8.5 8.0 7.5 7.0 6.5 6.0 5.5 5.0 4.5 4.0 3.5 3.0 2.5 2.0 1.5 1.0 0.5 0.0
f1 (ppm)

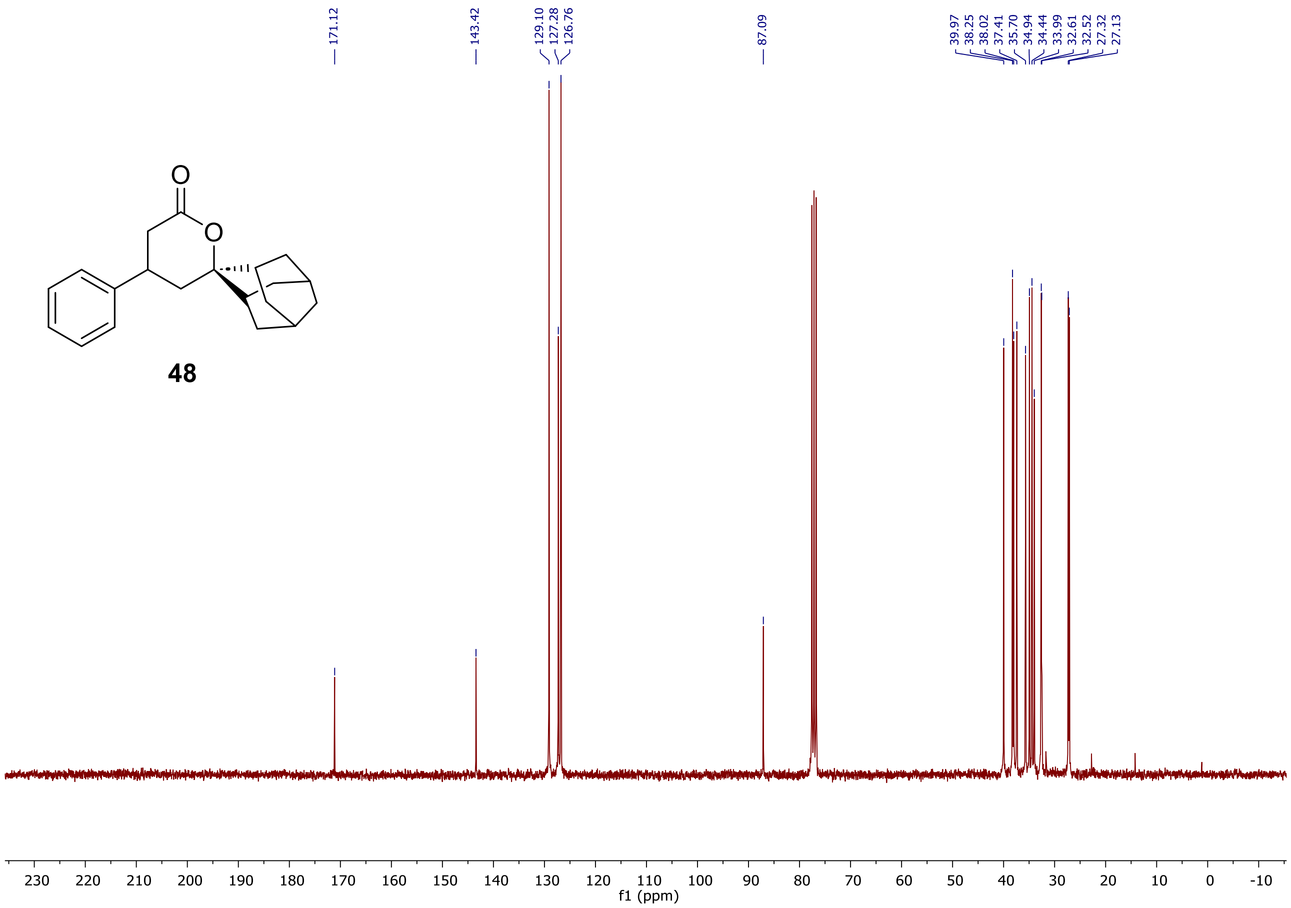
171.12
143.42
129.10 127.28 126.76
87.09
39.97 38.25 38.02 37.41 35.70 34.94 34.44 33.99 32.61 32.52 27.32 27.13
O
O
48
230 220 210 200 190 180 170 160 150 140 130 120 110 100 90 80 70 60 50 40 30 20 10 0 -10
f1 (ppm)

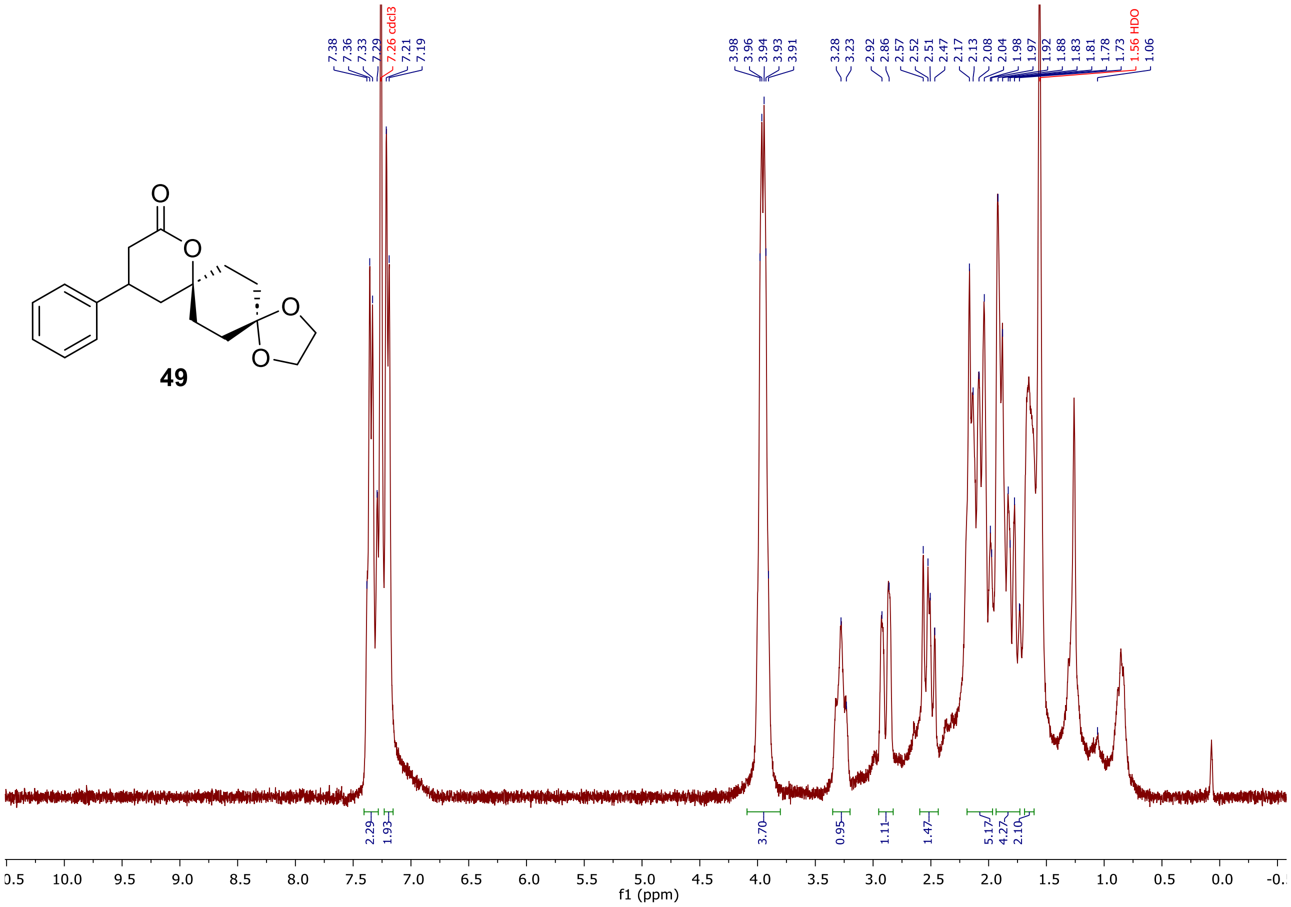
7.38
7.36
7.33
7.29
7.26 cdcl3
7.21
7.19
3.98
3.96
3.94
3.93
3.91
3.28
3.23
2.92
2.86
2.57
2.52
2.51
2.47
2.17
2.13
2.08
2.04
1.98
1.97
1.92
1.88
1.83
1.81
1.78
1.73
1.56 HDO
1.06
49
2.29
1.93
3.70
0.95
1.11
1.47
5.17
4.27
2.10
f1 (ppm)

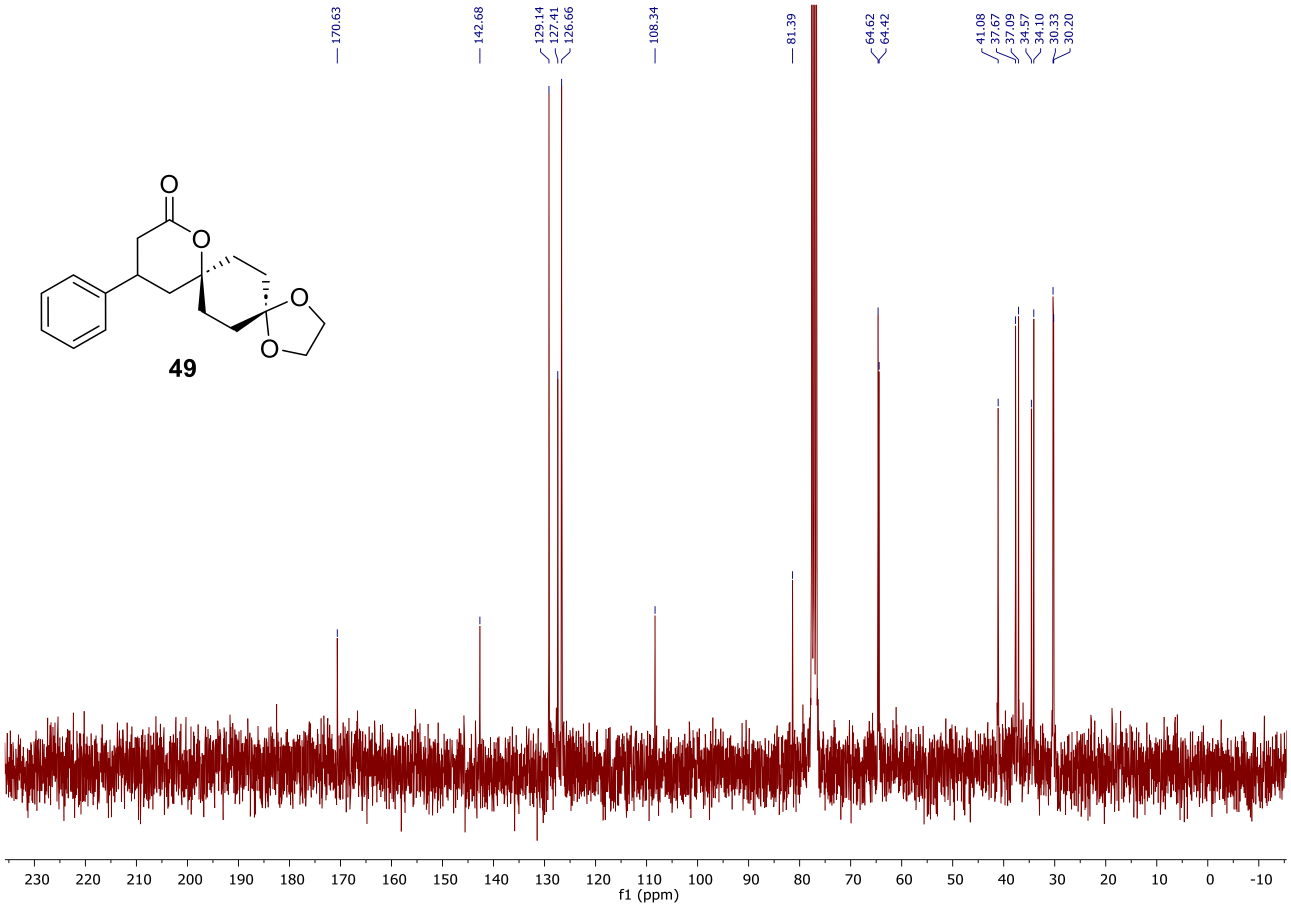
170.63
142.68
129.14
127.41
126.66
108.34
81.39
64.62
64.42
41.08
37.67
37.09
34.57
34.10
30.33
30.20
49
f1 (ppm)

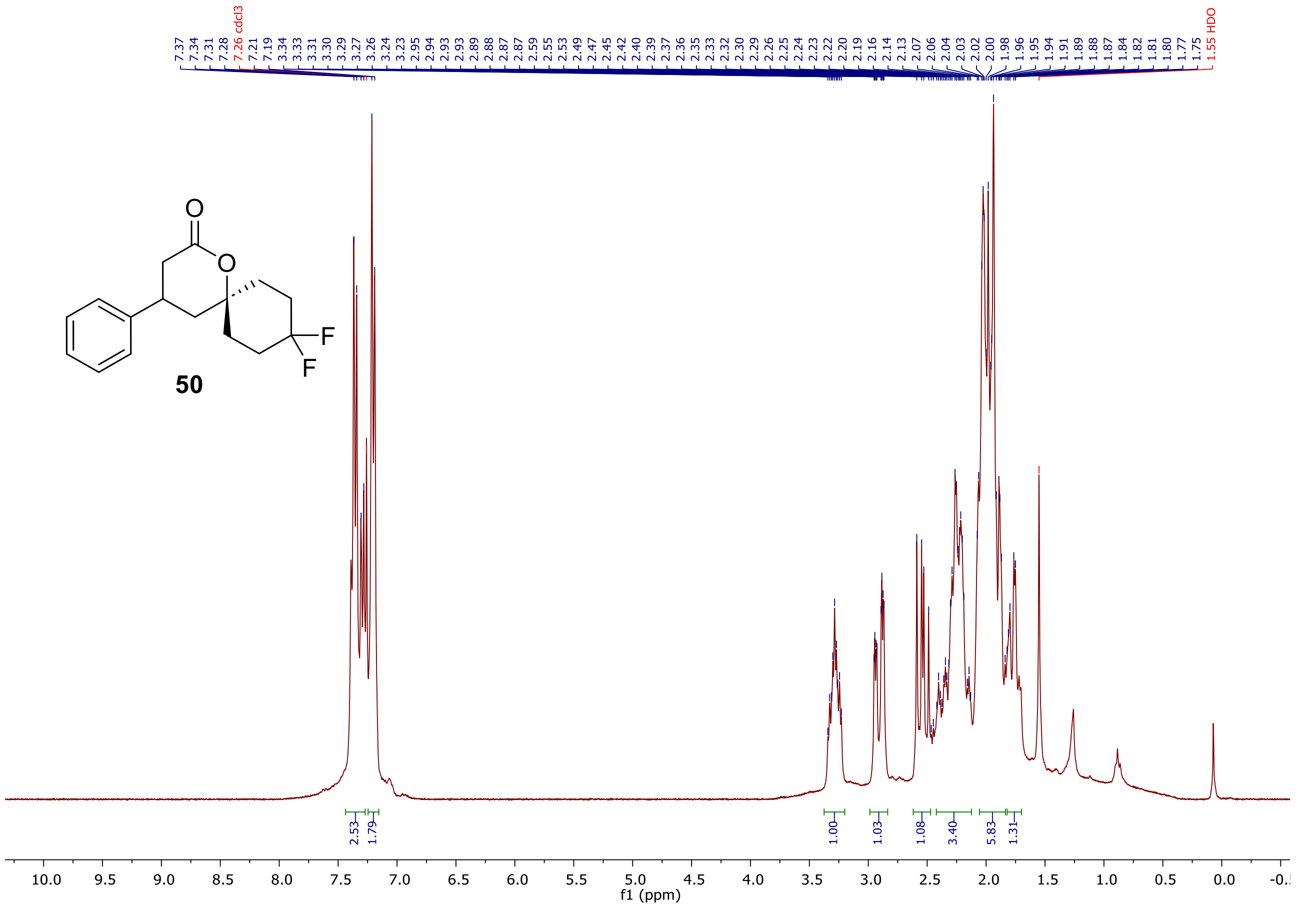
7.37 7.34 7.31 7.28 7.26 cdcl3 7.21 7.19 3.34 3.33 3.31 3.30 3.29 3.27 3.26 3.24 3.23 2.95 2.94 2.93 2.93 2.89 2.88 2.87 2.87 2.59 2.55 2.53 2.49 2.47 2.45 2.42 2.40 2.39 2.37 2.36 2.35 2.33 2.32 2.30 2.29 2.26 2.25 2.24 2.23 2.22 2.20 2.19 2.16 2.14 2.13 2.07 2.06 2.04 2.03 2.02 2.00 1.98 1.96 1.95 1.94 1.91 1.89 1.88 1.87 1.84 1.82 1.81 1.80 1.77 1.75 1.55 HDO
O
O
F
F
50
2.53 1.79 1.00 1.03 1.08 3.40 5.83 1.31
10.0 9.5 9.0 8.5 8.0 7.5 7.0 6.5 6.0 5.5 5.0 4.5 4.0 3.5 3.0 2.5 2.0 1.5 1.0 0.5 0.0
f1 (ppm)

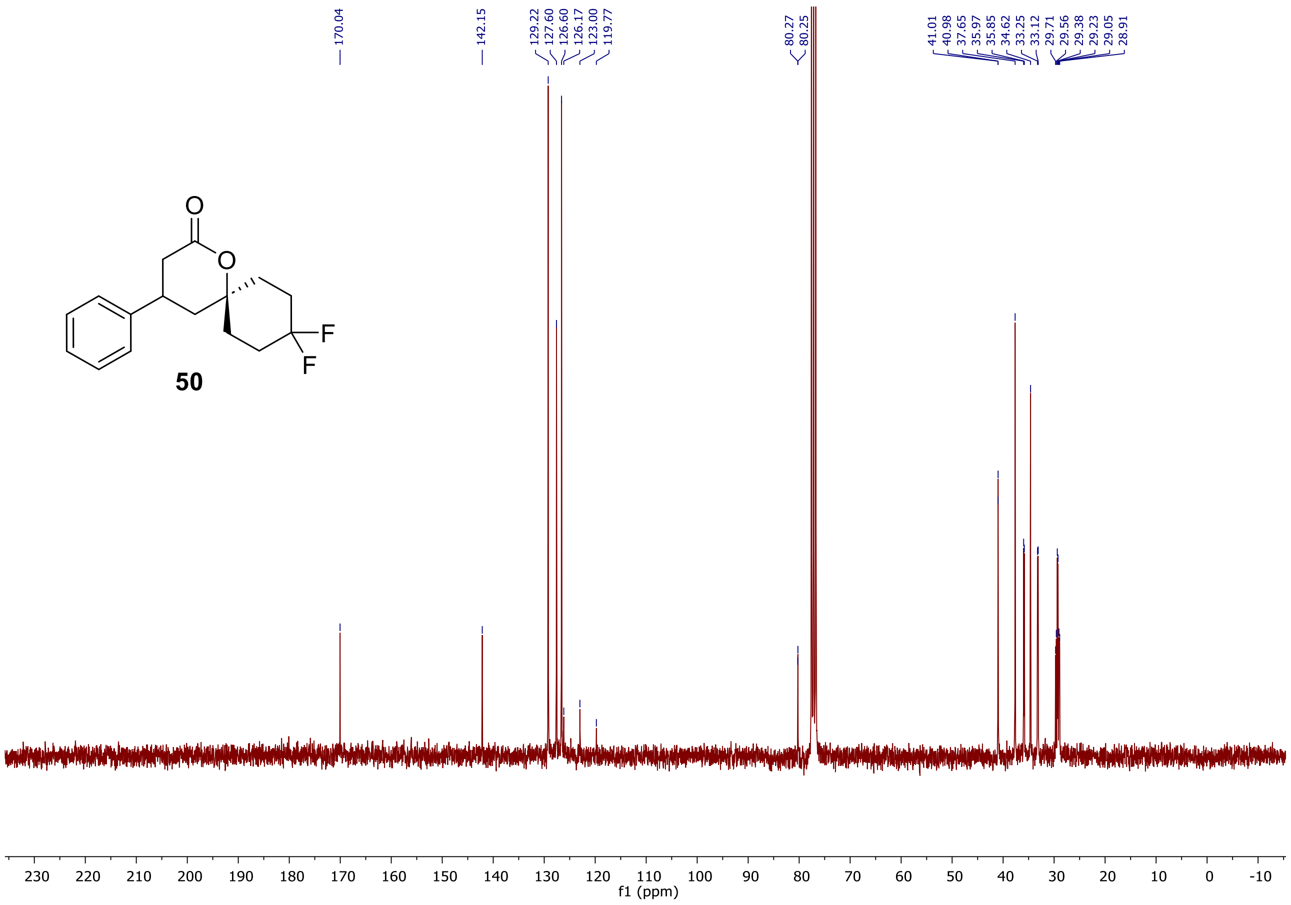
170.04 142.15 129.22 127.60 126.60 126.17 123.00 119.77 80.27 80.25 41.01 40.98 37.65 35.97 35.85 34.62 33.25 33.12 29.71 29.56 29.38 29.23 29.05 28.91
O
O
F
F
50
230 220 210 200 190 180 170 160 150 140 130 120 110 100 90 80 70 60 50 40 30 20 10 0 -10
f1 (ppm)

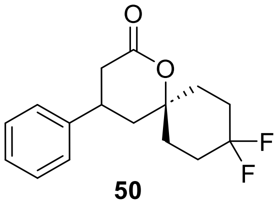

50

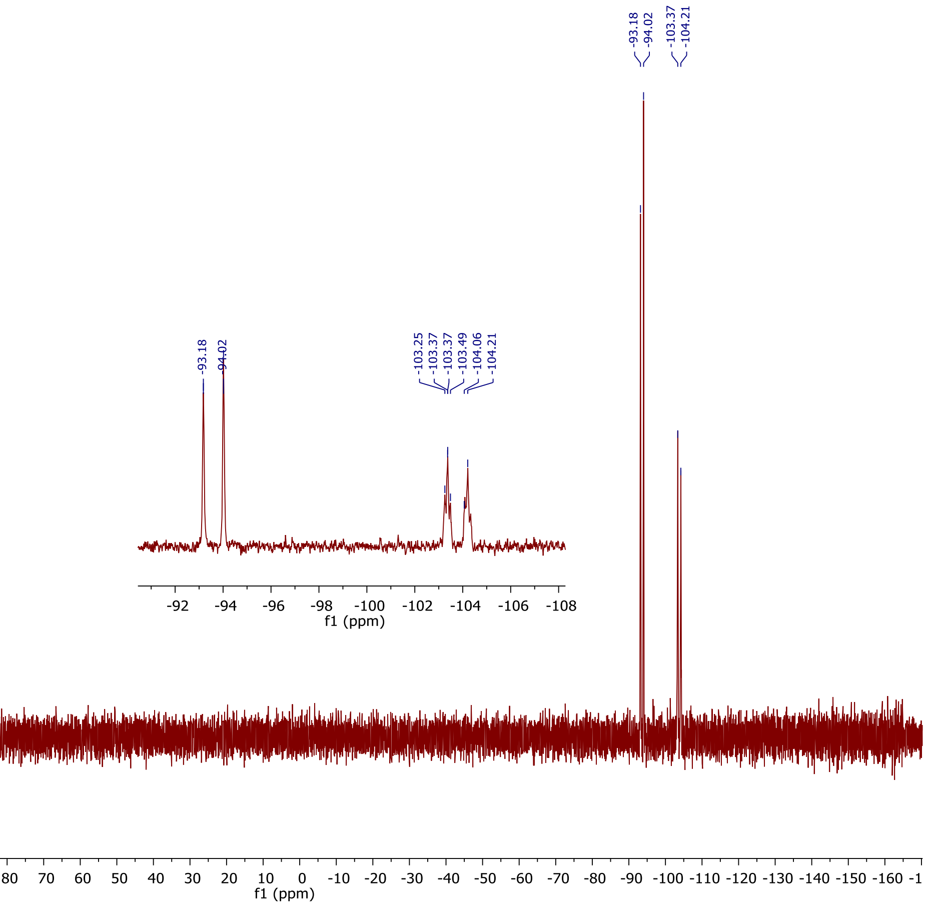

-93.18
-94.02
-103.37
-104.21
-103.25
-103.37
-103.37
-103.49
-104.06
-104.21
-92 -94 -96 -98 -100 -102 -104 -106 -108
f1 (ppm)
f1 (ppm)

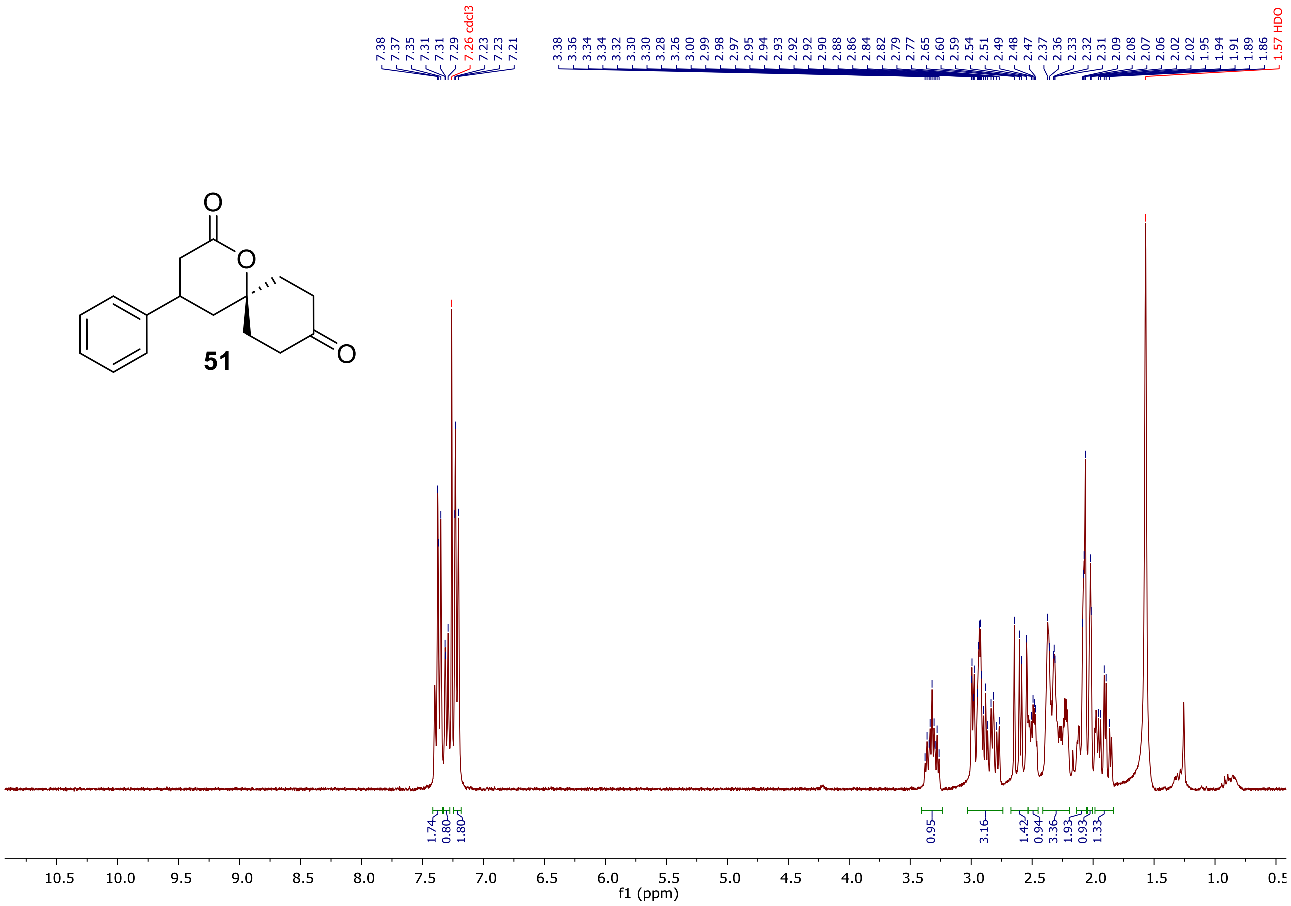
7.38
7.37
7.35
7.31
7.31
7.29
7.26 cdcl3
7.23
7.23
7.21
3.38
3.36
3.34
3.34
3.32
3.30
3.30
3.28
3.26
3.00
2.99
2.98
2.97
2.95
2.94
2.93
2.92
2.92
2.90
2.88
2.86
2.84
2.82
2.79
2.77
2.65
2.60
2.59
2.54
2.51
2.49
2.48
2.47
2.37
2.36
2.33
2.32
2.31
2.09
2.08
2.07
2.06
2.02
2.02
1.95
1.94
1.91
1.89
1.86
1.57 HDO
O
O
O
51
1.74
0.80
1.80
0.95
3.16
1.42
0.94
3.36
1.93
0.93
1.33
10.5
10.0
9.5
9.0
8.5
8.0
7.5
7.0
6.5
6.0
5.5
5.0
4.5
4.0
3.5
3.0
2.5
2.0
1.5
1.0
0.5
f1 (ppm)

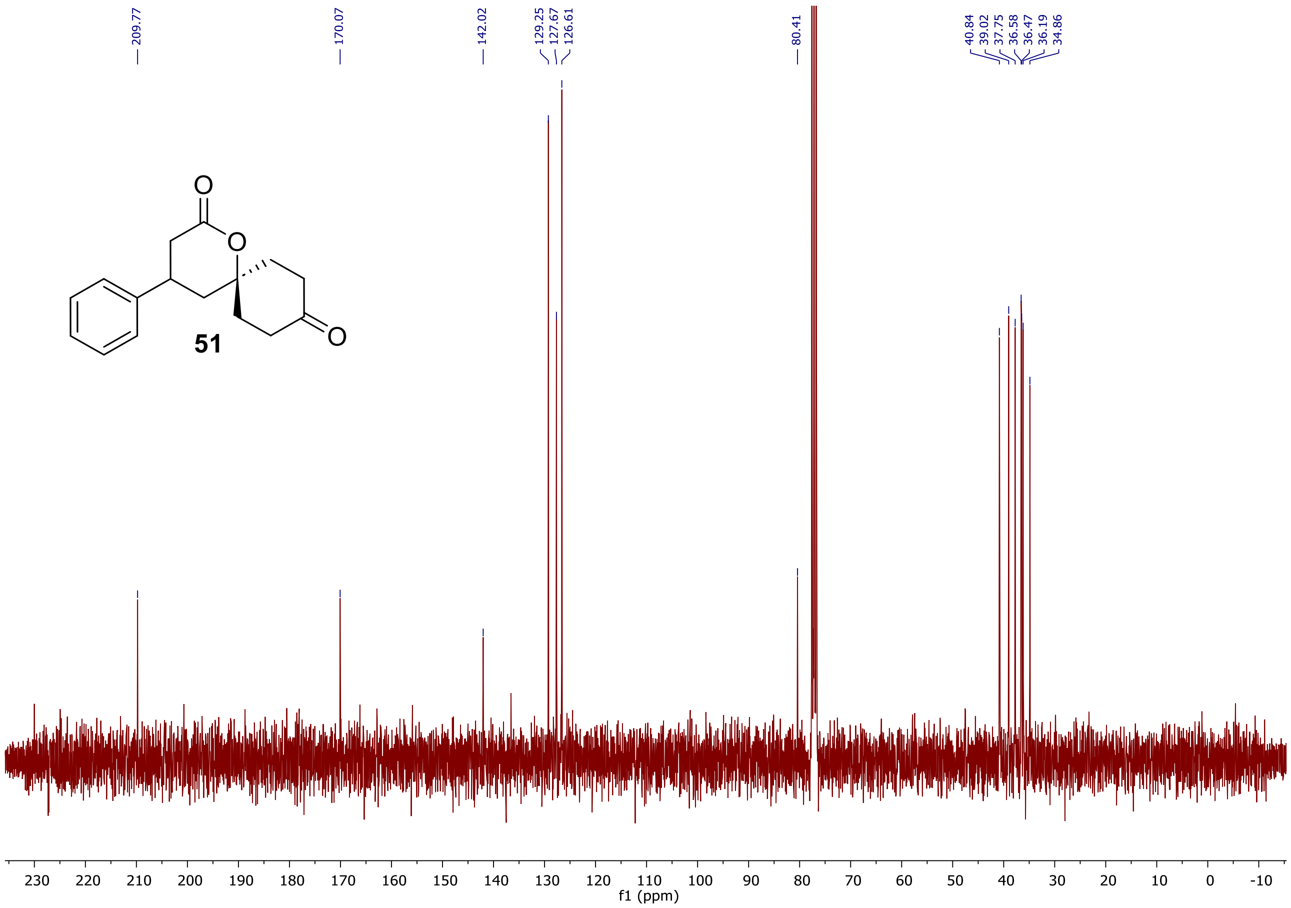
209.77
170.07
142.02
129.25
127.67
126.61
80.41
40.84
39.02
37.75
36.58
36.47
36.19
34.86
O
O
O
51
230
220
210
200
190
180
170
160
150
140
130
120
110
100
90
80
70
60
50
40
30
20
10
0
-10
f1 (ppm)

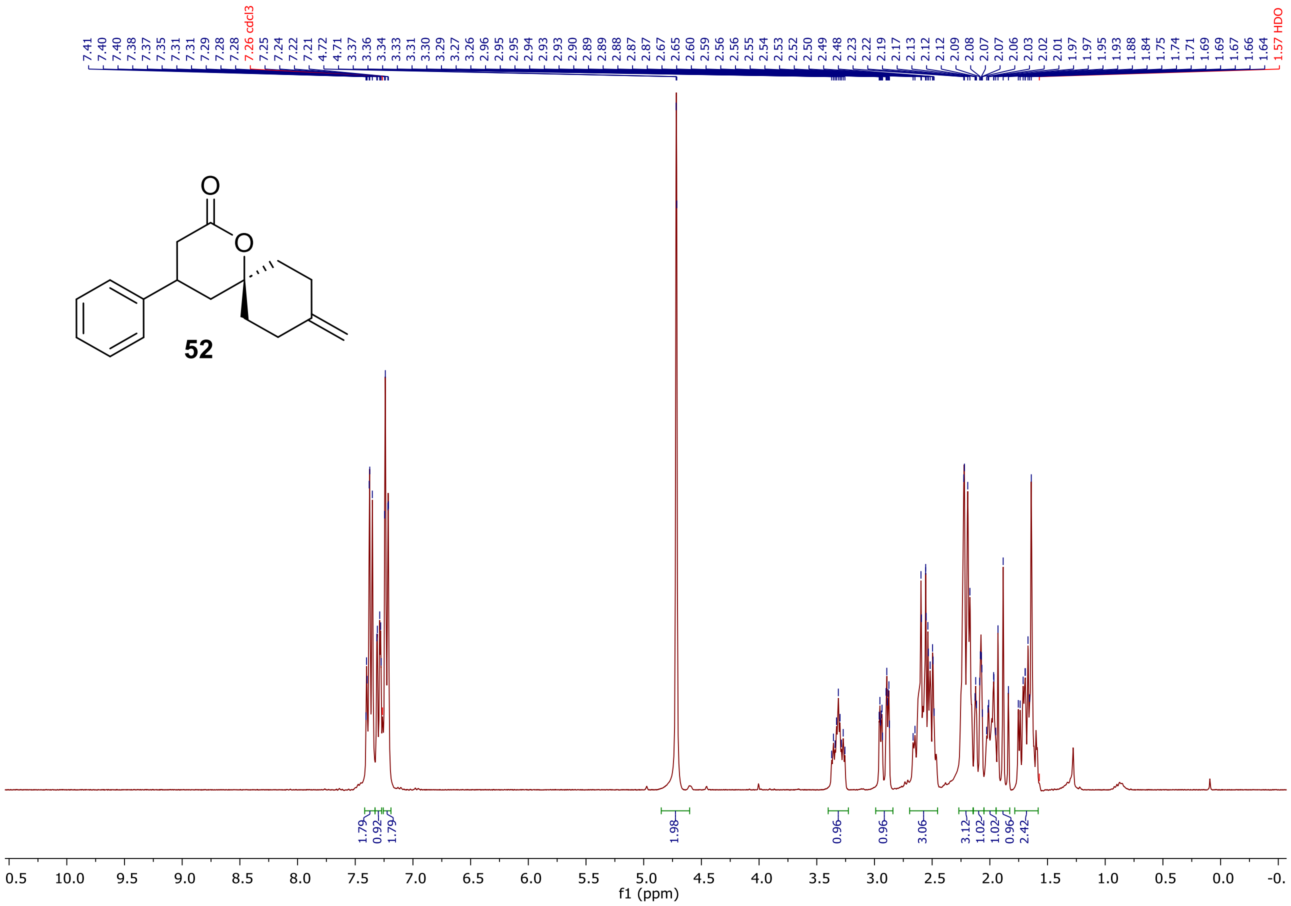
7.41 7.40 7.40 7.38 7.37 7.35 7.31 7.31 7.29 7.28 7.28 7.26 cdcl3 7.25 7.24 7.22 7.21 4.72 4.71 3.37 3.36 3.34 3.33 3.31 3.30 3.29 3.27 3.26 2.96 2.95 2.95 2.94 2.93 2.93 2.90 2.89 2.89 2.88 2.87 2.87 2.67 2.65 2.60 2.59 2.56 2.56 2.55 2.54 2.53 2.52 2.50 2.49 2.48 2.23 2.22 2.19 2.17 2.13 2.12 2.12 2.09 2.08 2.07 2.07 2.06 2.03 2.02 2.01 1.97 1.97 1.95 1.93 1.88 1.84 1.75 1.74 1.71 1.69 1.69 1.67 1.66 1.64 1.57 HDO
O
O
52
1.79 0.92 1.79 1.98 0.96 0.96 3.06 3.12 1.02 1.02 0.96 2.42
f1 (ppm)

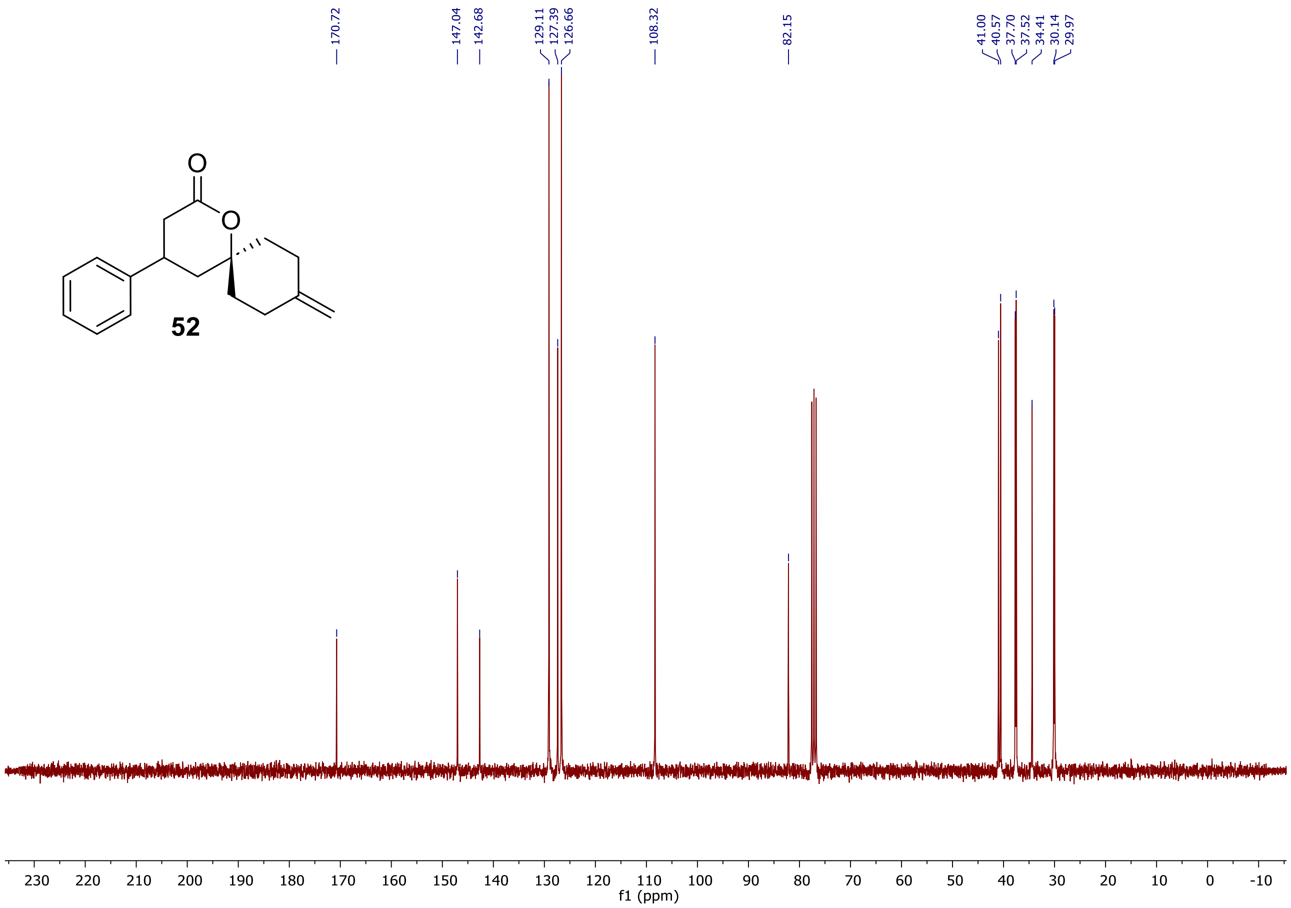
170.72 147.04 142.68 129.11 127.39 126.66 108.32 82.15 41.00 40.57 37.70 37.52 34.41 30.14 29.97
O
O
52
f1 (ppm)

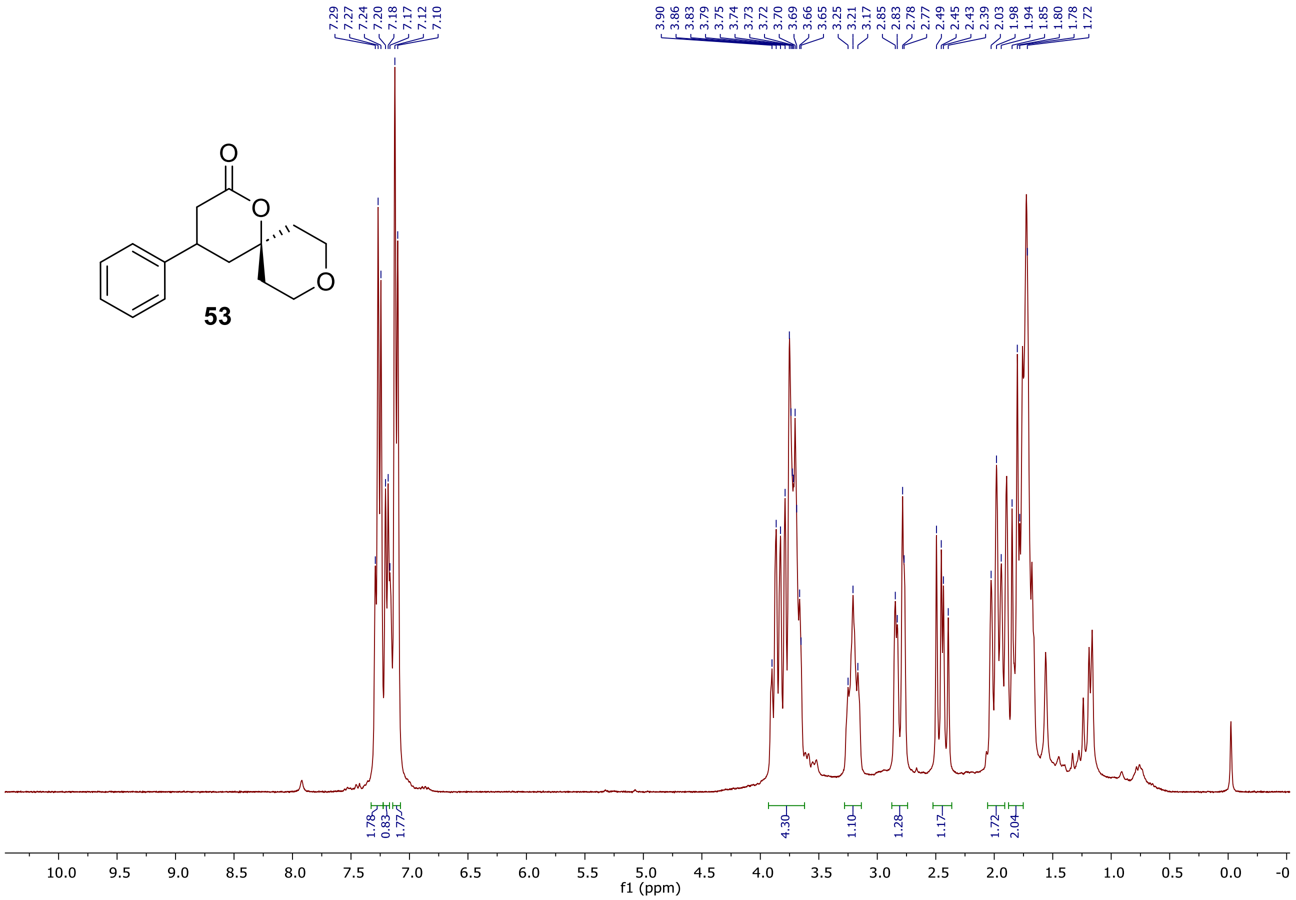
7.29
7.27
7.24
7.20
7.18
7.17
7.12
7.10
3.90
3.86
3.83
3.79
3.75
3.74
3.73
3.72
3.70
3.69
3.66
3.65
3.25
3.21
3.17
2.85
2.83
2.78
2.77
2.49
2.45
2.43
2.39
2.03
1.98
1.94
1.85
1.80
1.78
1.72
O
O
O
53
1.78
0.83
1.77
4.30
1.10
1.28
1.17
1.72
2.04
10.0 9.5 9.0 8.5 8.0 7.5 7.0 6.5 6.0 5.5 5.0 4.5 4.0 3.5 3.0 2.5 2.0 1.5 1.0 0.5 0.0 -0
f1 (ppm)

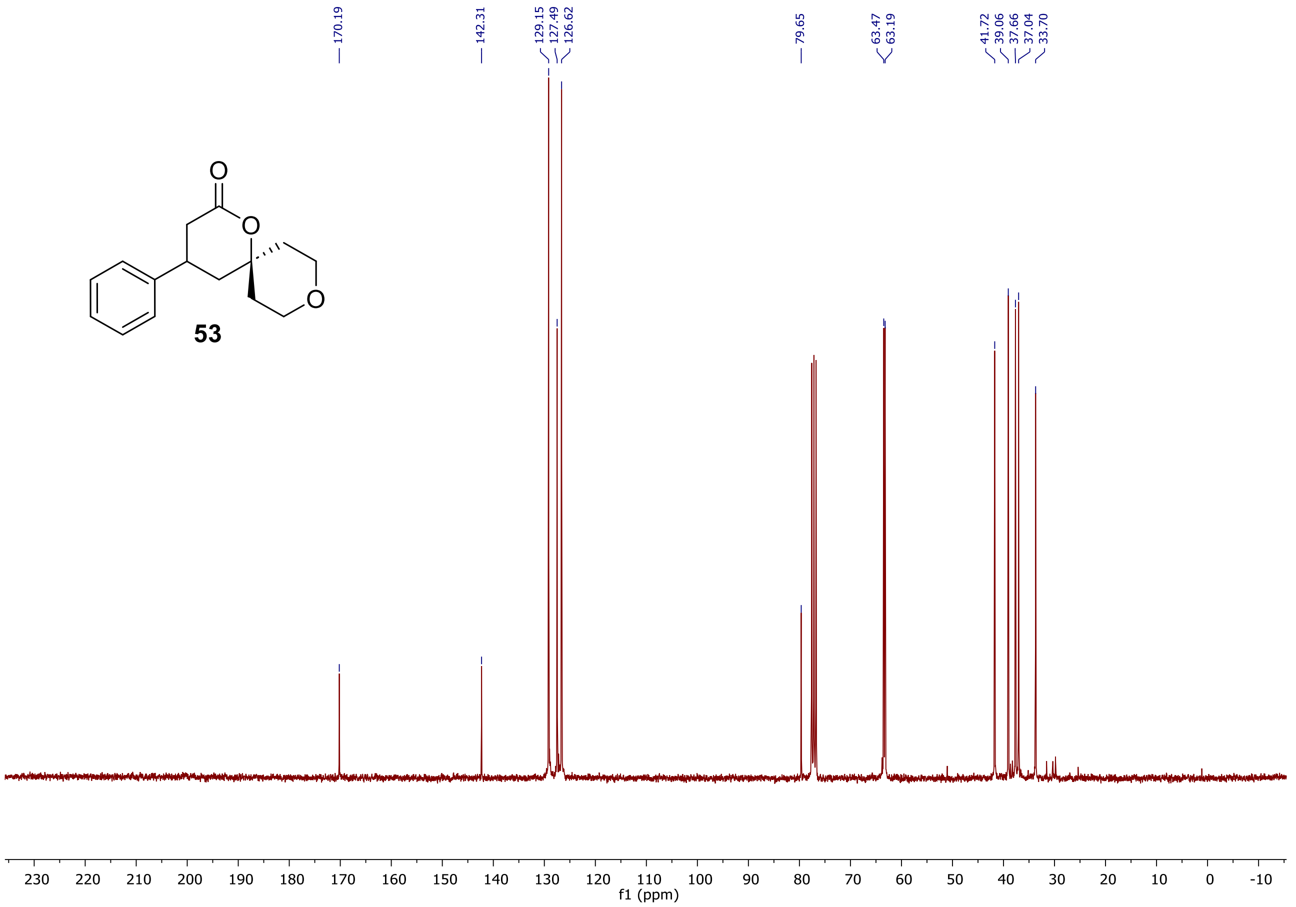
170.19
142.31
129.15
127.49
126.62
79.65
63.47
63.19
41.72
39.06
37.66
37.04
33.70
O
O
O
53
230 220 210 200 190 180 170 160 150 140 130 120 110 100 90 80 70 60 50 40 30 20 10 0 -10
f1 (ppm)

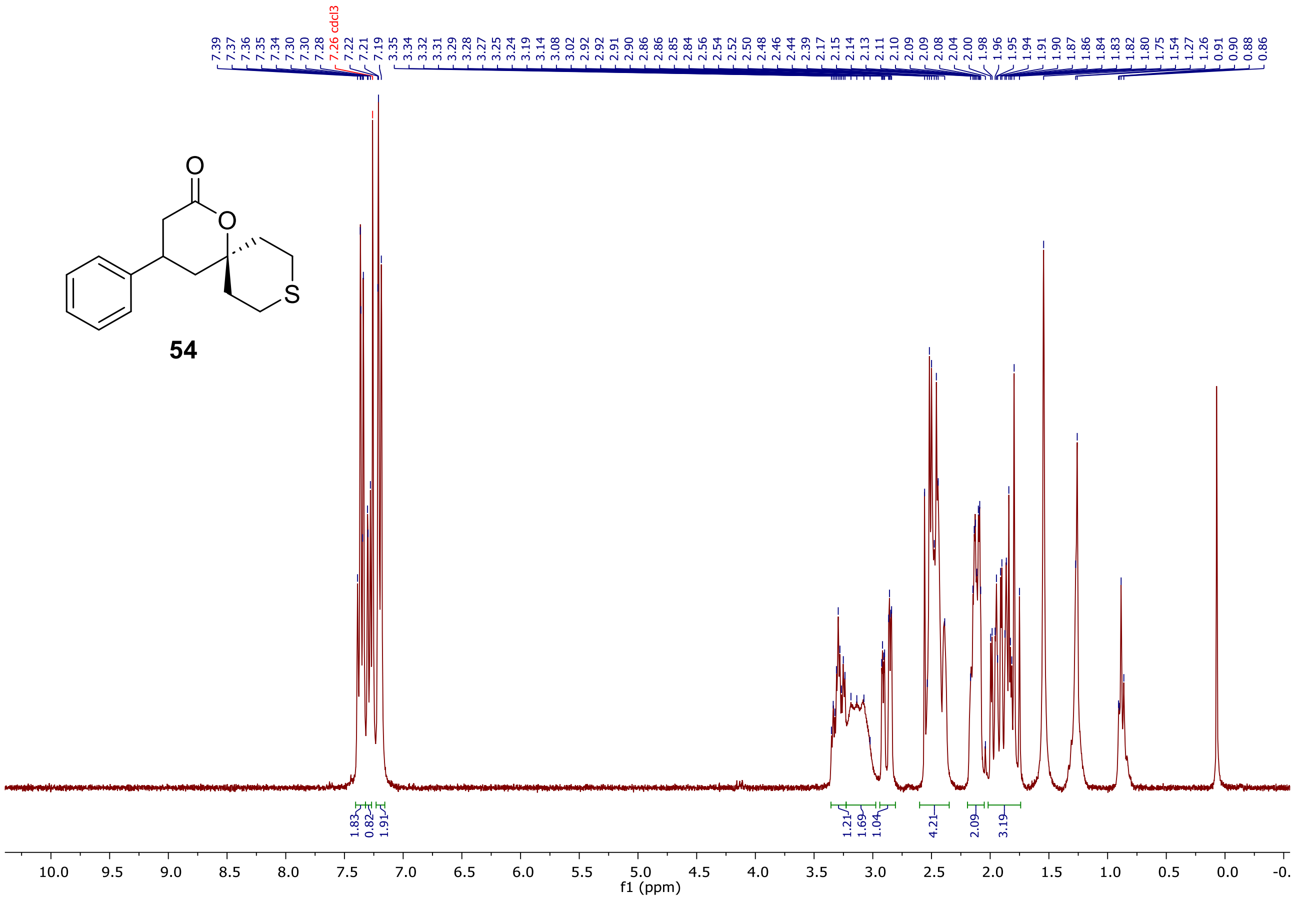
54
f1 (ppm)

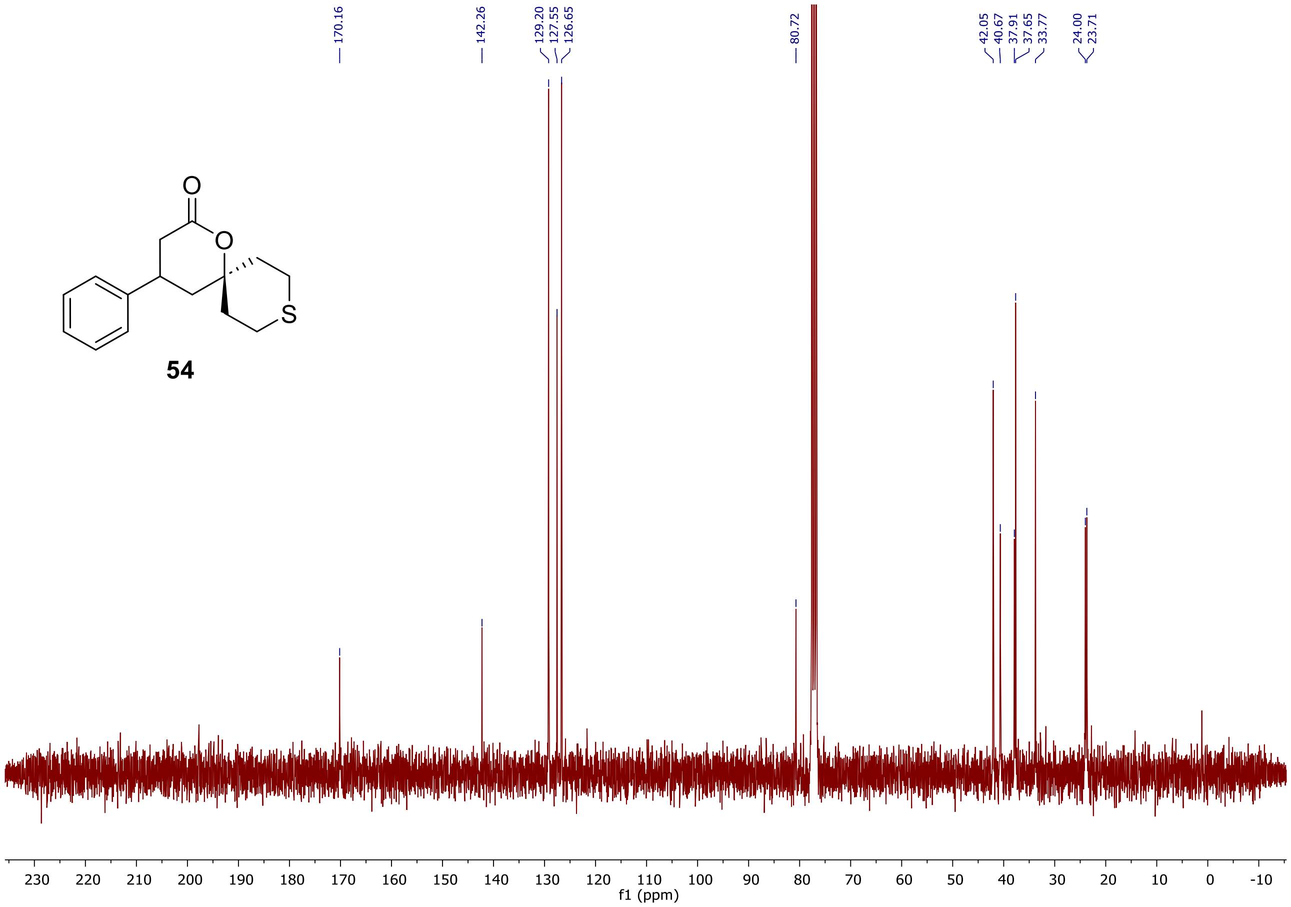
54
f1 (ppm)

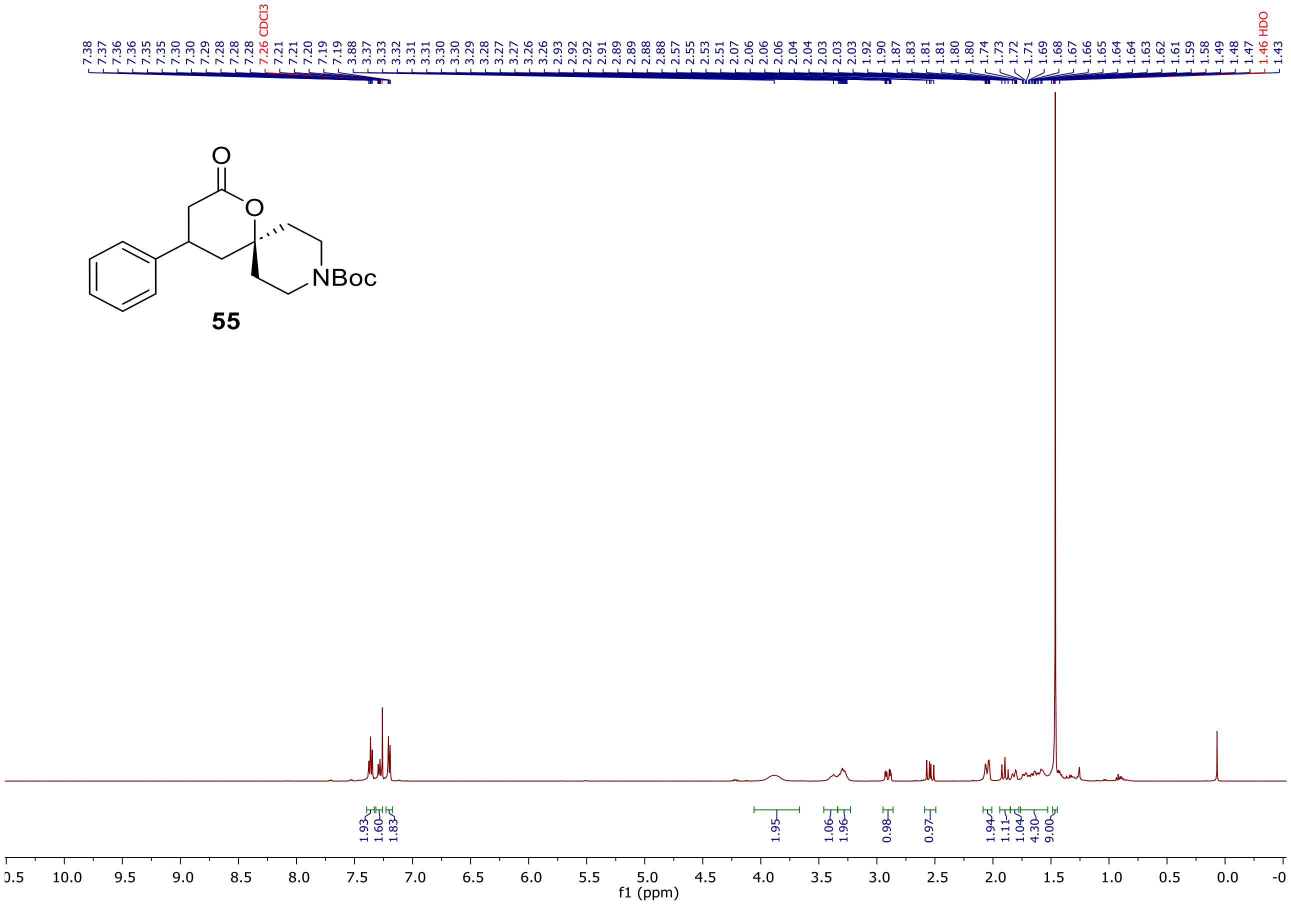

55

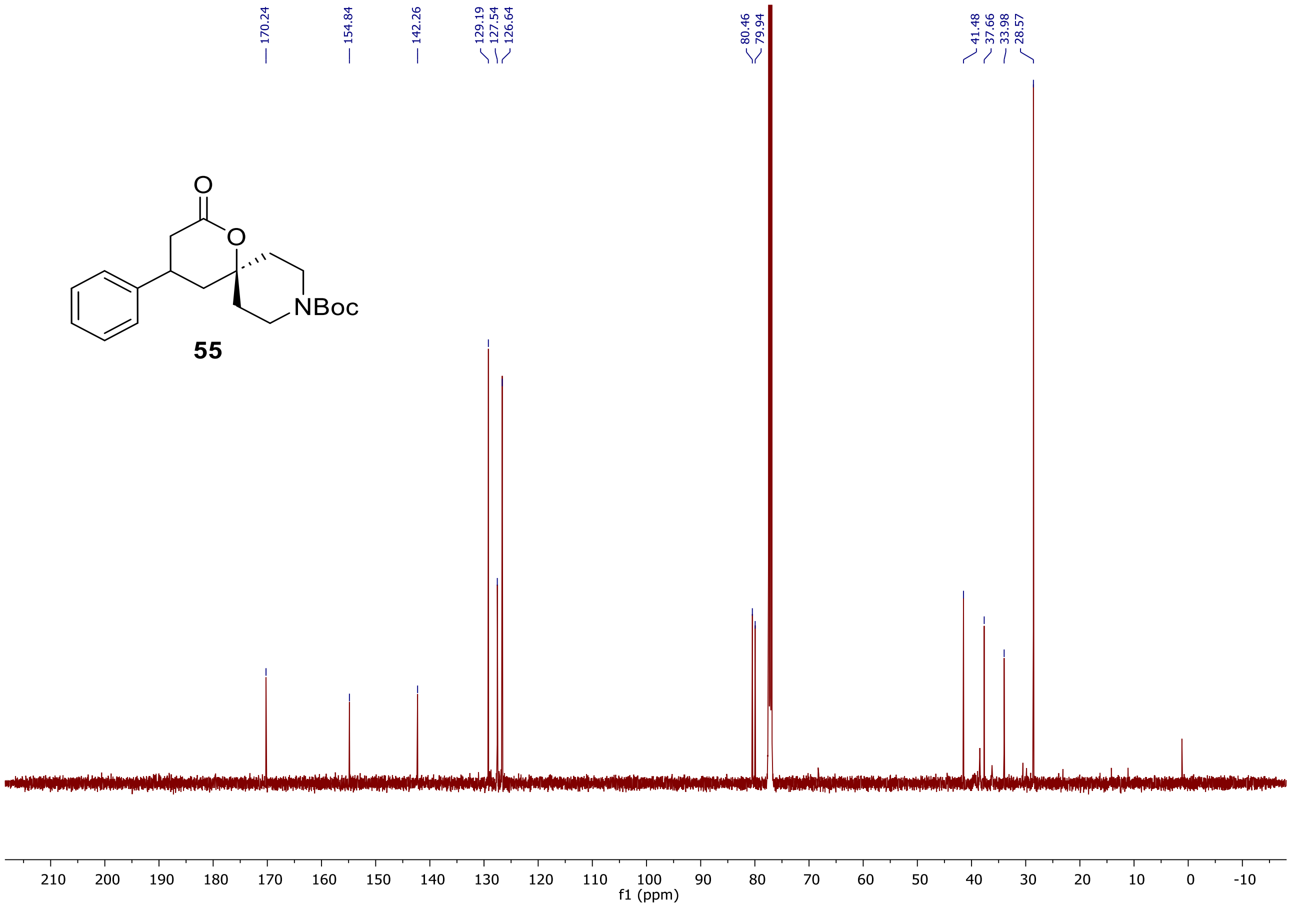

55

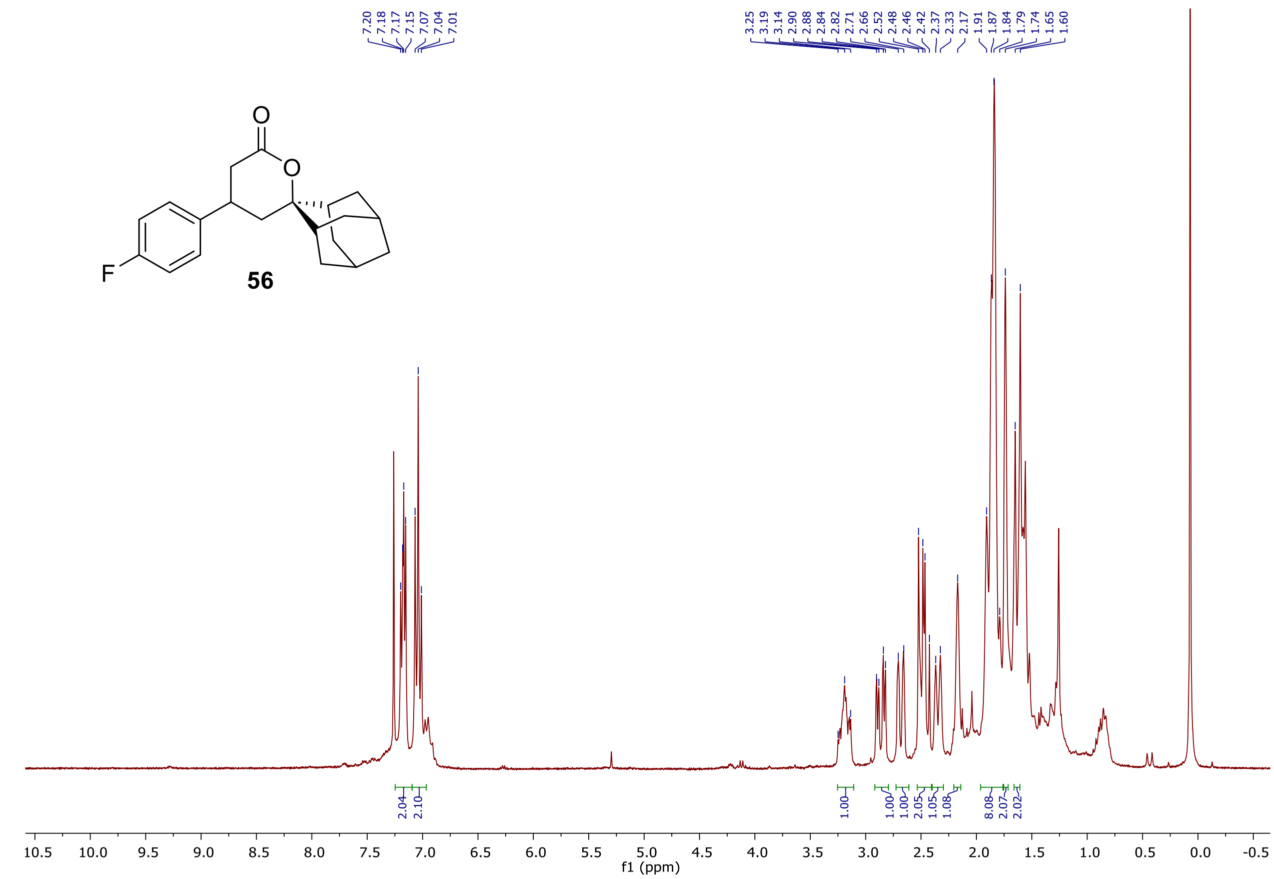
7.20
7.18
7.17
7.15
7.07
7.04
7.01
3.25
3.19
3.14
2.90
2.88
2.84
2.82
2.71
2.66
2.52
2.48
2.46
2.42
2.37
2.33
2.17
1.91
1.87
1.84
1.79
1.74
1.65
1.60
O
O
F
56
2.04
2.10
1.00
1.00
1.00
2.05
1.05
1.08
8.08
2.07
2.02
f1 (ppm)

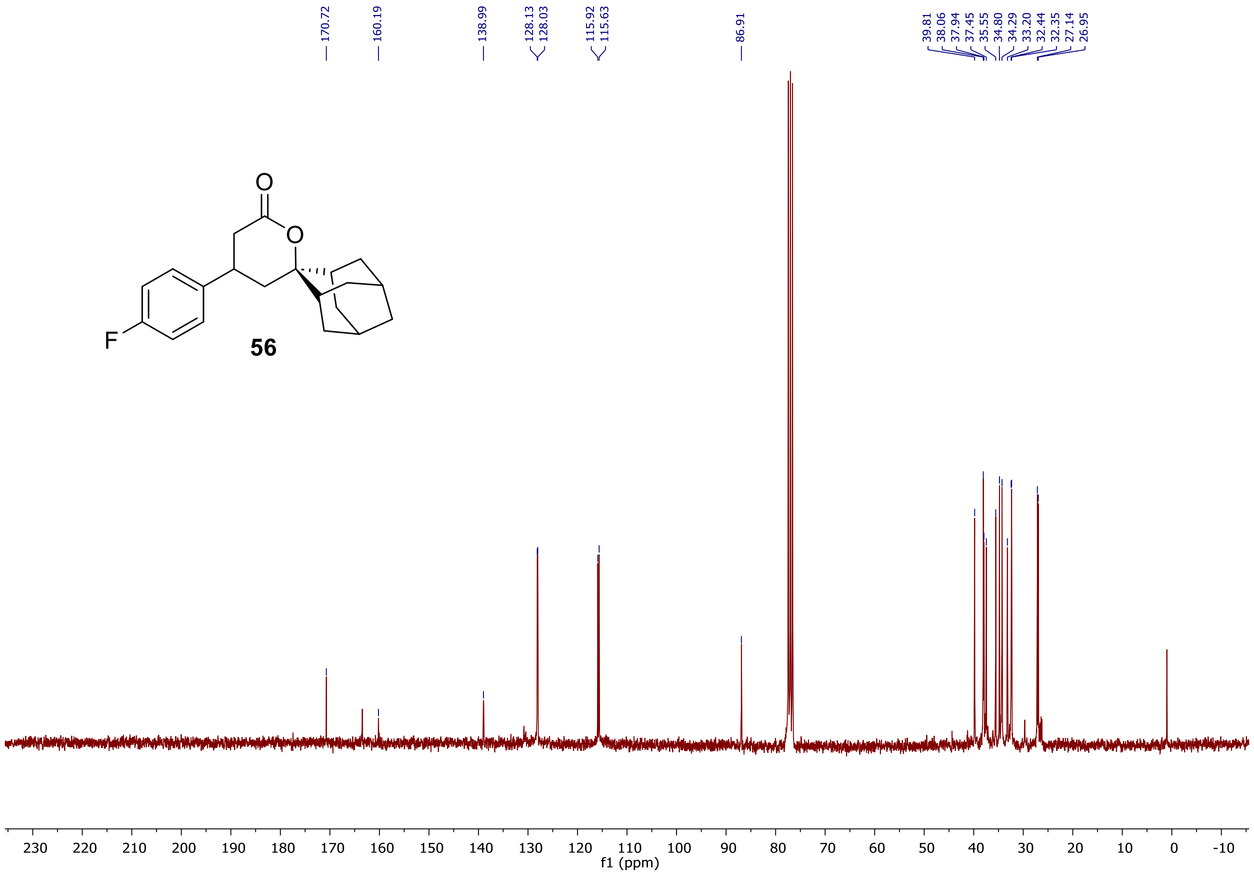
170.72
160.19
138.99
128.13
128.03
115.92
115.63
86.91
39.81
38.06
37.94
37.45
35.55
34.80
34.29
33.20
32.44
32.35
27.14
26.95
O
O
F
56
f1 (ppm)

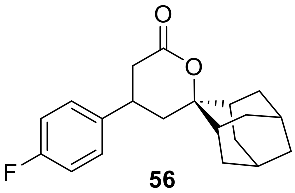

56

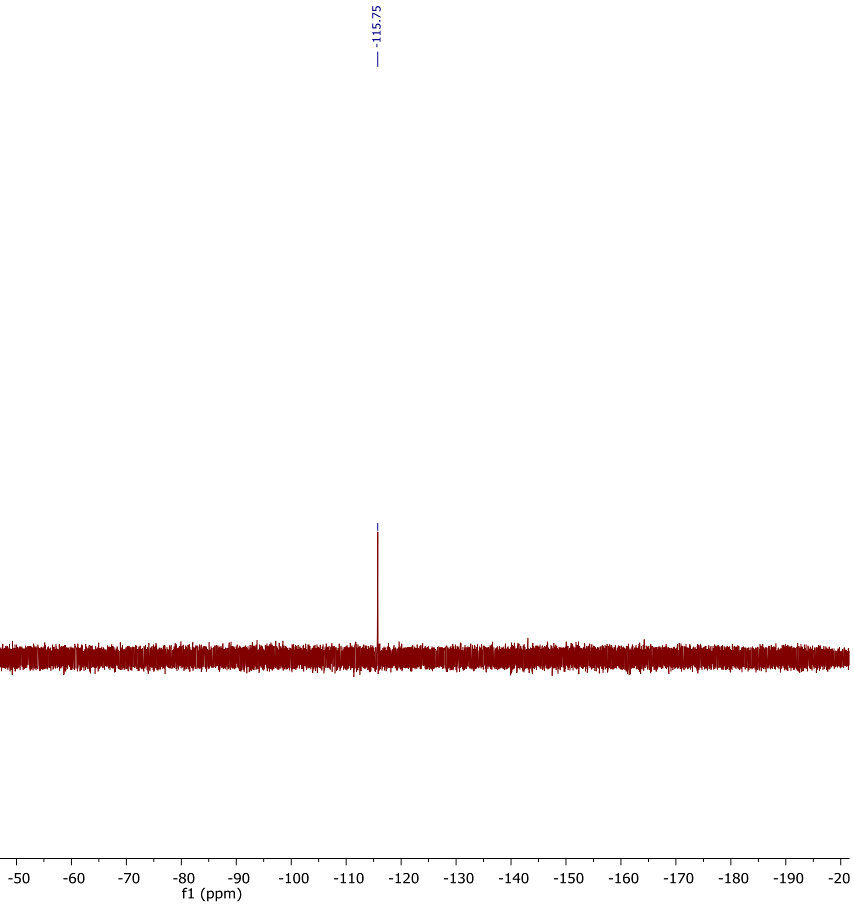

-115.75
30 20 10 0 -10 -20 -30 -40 -50 -60 -70 -80 -90 -100 -110 -120 -130 -140 -150 -160 -170 -180 -190 -20
f1 (ppm)

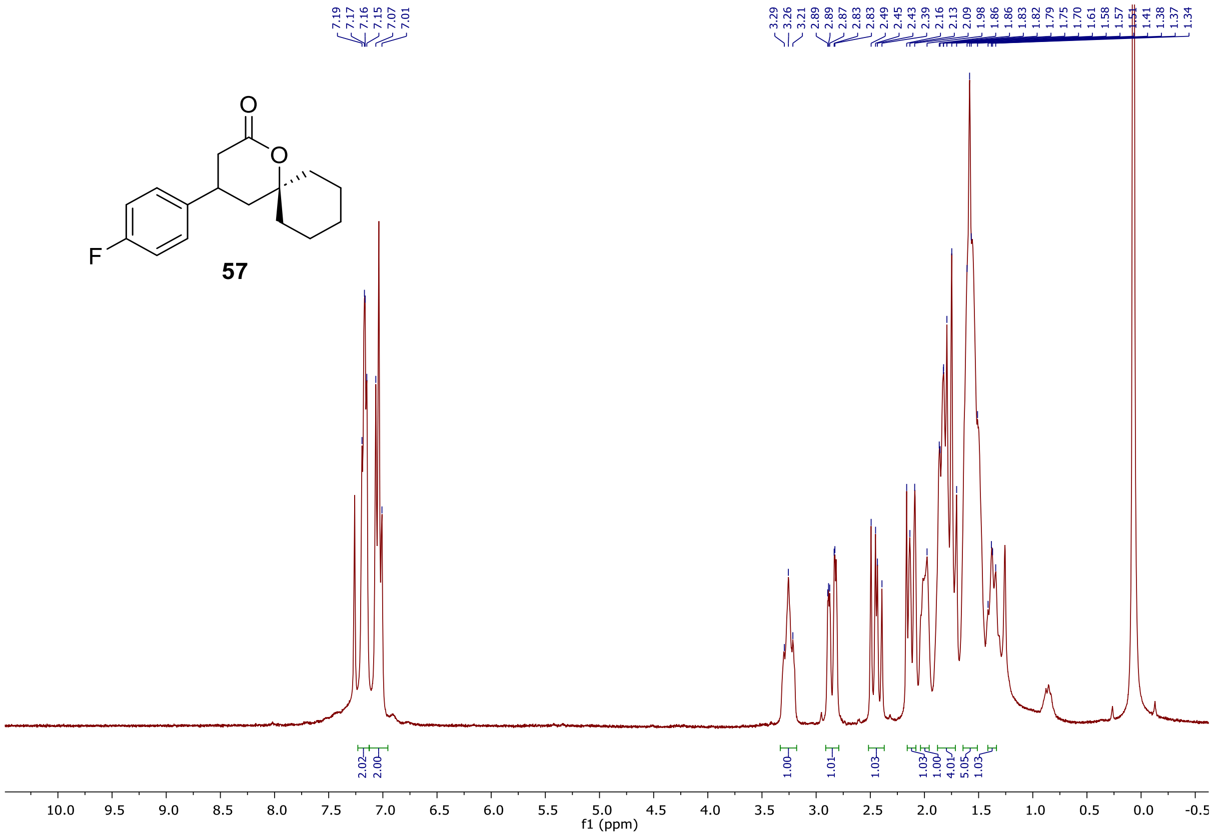
7.19
7.17
7.16
7.15
7.07
7.01
3.29
3.26
3.21
2.89
2.89
2.87
2.83
2.83
2.49
2.45
2.43
2.39
2.16
2.13
2.09
1.98
1.86
1.86
1.83
1.82
1.79
1.75
1.70
1.61
1.58
1.57
1.51
1.41
1.38
1.37
1.34
O
O
F
57
2.02
2.00
1.00
1.01
1.03
1.03
1.00
4.01
5.05
1.03
10.0
9.5
9.0
8.5
8.0
7.5
7.0
6.5
6.0
5.5
5.0
4.5
4.0
3.5
3.0
2.5
2.0
1.5
1.0
0.5
0.0
-0.5
f1 (ppm)

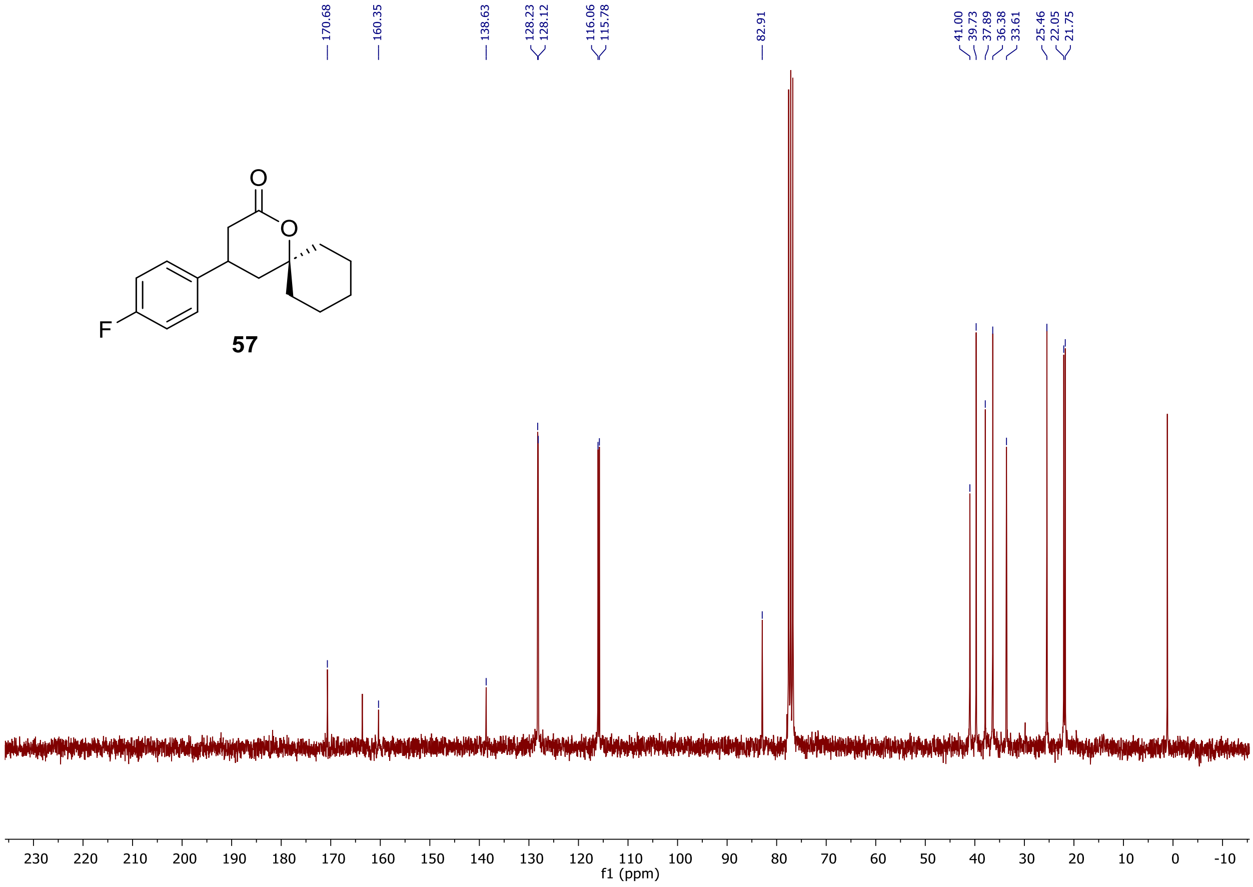
170.68
160.35
138.63
128.23
128.12
116.06
115.78
82.91
41.00
39.73
37.89
36.38
33.61
25.46
22.05
21.75
O
O
F
57
230
220
210
200
190
180
170
160
150
140
130
120
110
100
90
80
70
60
50
40
30
20
10
0
-10
f1 (ppm)

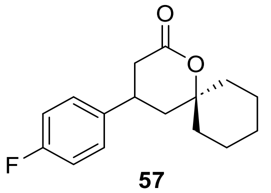

57

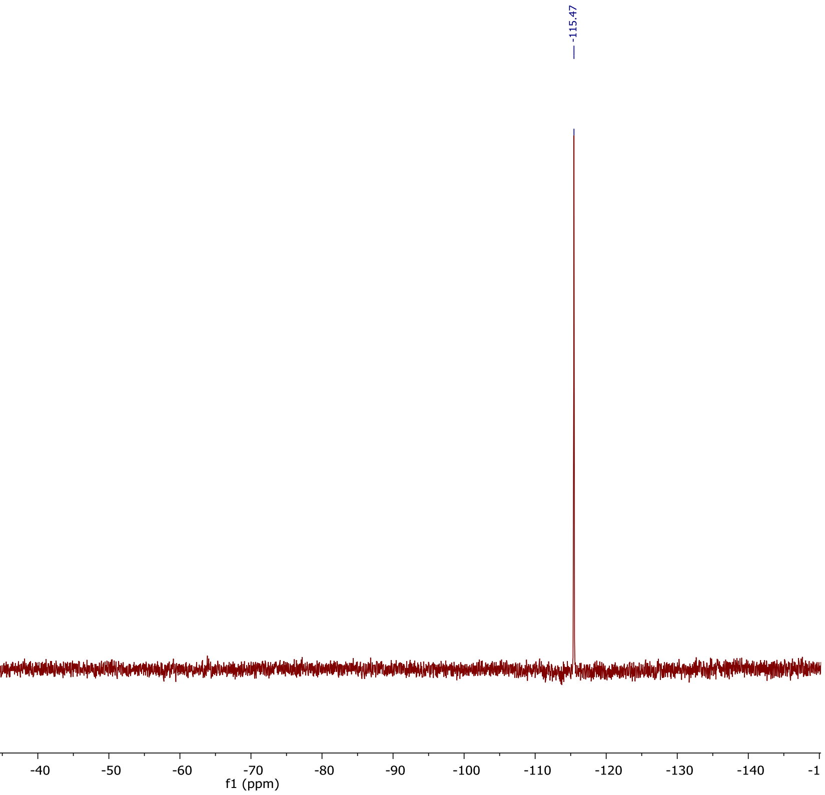

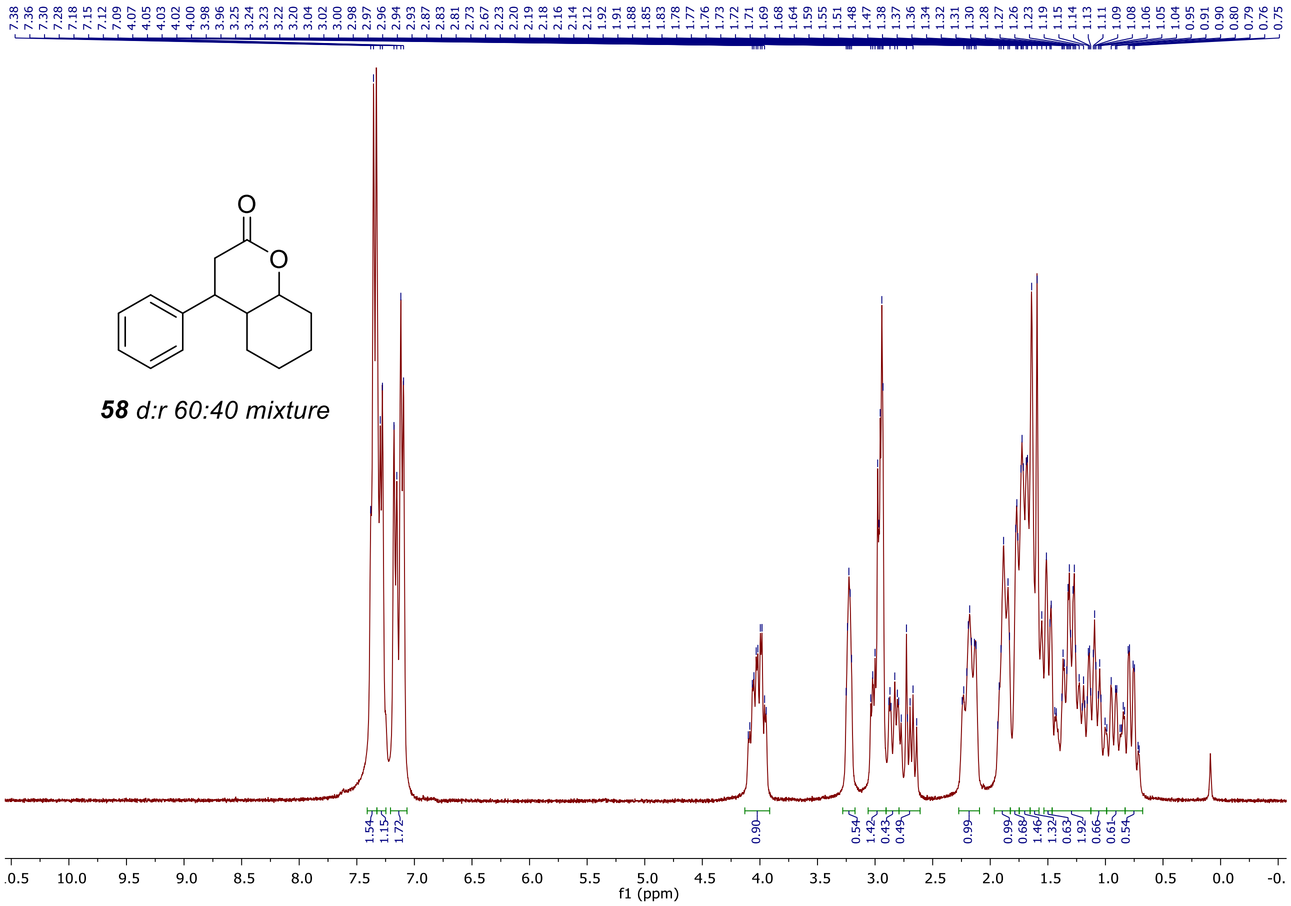
58 d:r 60:40 mixture
7.38 7.36 7.30 7.28 7.18 7.15 7.12 7.09 4.07 4.05 4.03 4.02 4.00 3.98 3.96 3.25 3.24 3.23 3.22 3.20 3.04 3.02 3.00 2.98 2.97 2.96 2.94 2.93 2.87 2.83 2.81 2.73 2.67 2.23 2.20 2.19 2.18 2.16 2.14 2.12 1.92 1.91 1.88 1.85 1.83 1.78 1.77 1.76 1.73 1.72 1.71 1.69 1.68 1.64 1.59 1.55 1.51 1.48 1.47 1.38 1.37 1.36 1.34 1.32 1.31 1.30 1.28 1.27 1.26 1.23 1.19 1.15 1.14 1.13 1.11 1.09 1.08 1.06 1.05 1.04 0.95 0.91 0.90 0.80 0.79 0.76 0.75
1.54 1.15 1.72 0.90 0.54 1.42 0.43 0.49 0.99 0.99 0.68 1.46 1.32 0.63 1.92 0.66 0.61 0.54
f1 (ppm)

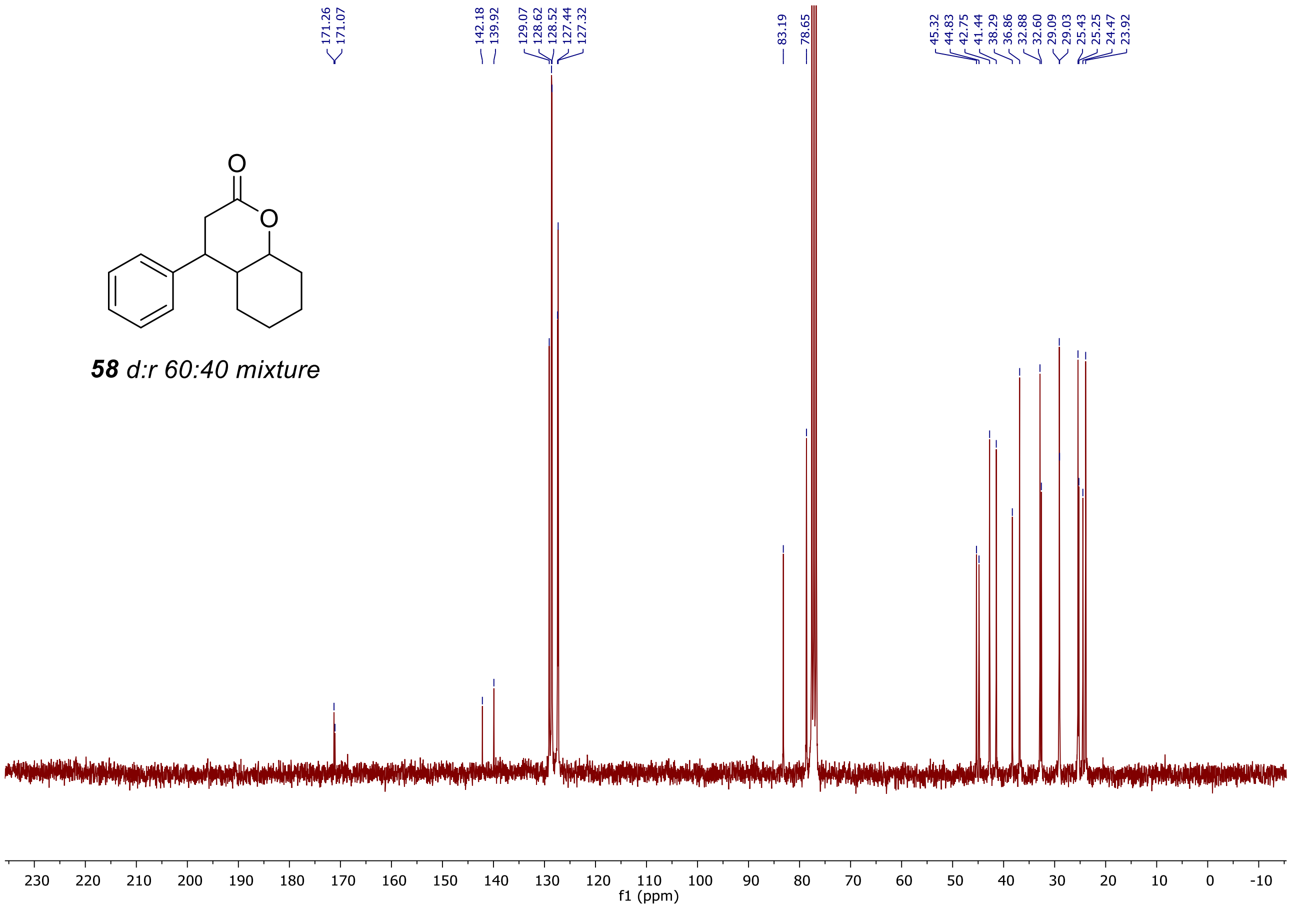
58 d:r 60:40 mixture
171.26 171.07 142.18 139.92 129.07 128.62 128.52 127.44 127.32 83.19 78.65 45.32 44.83 42.75 41.44 38.29 36.86 32.88 32.60 29.09 29.03 25.43 25.25 24.47 23.92
f1 (ppm)

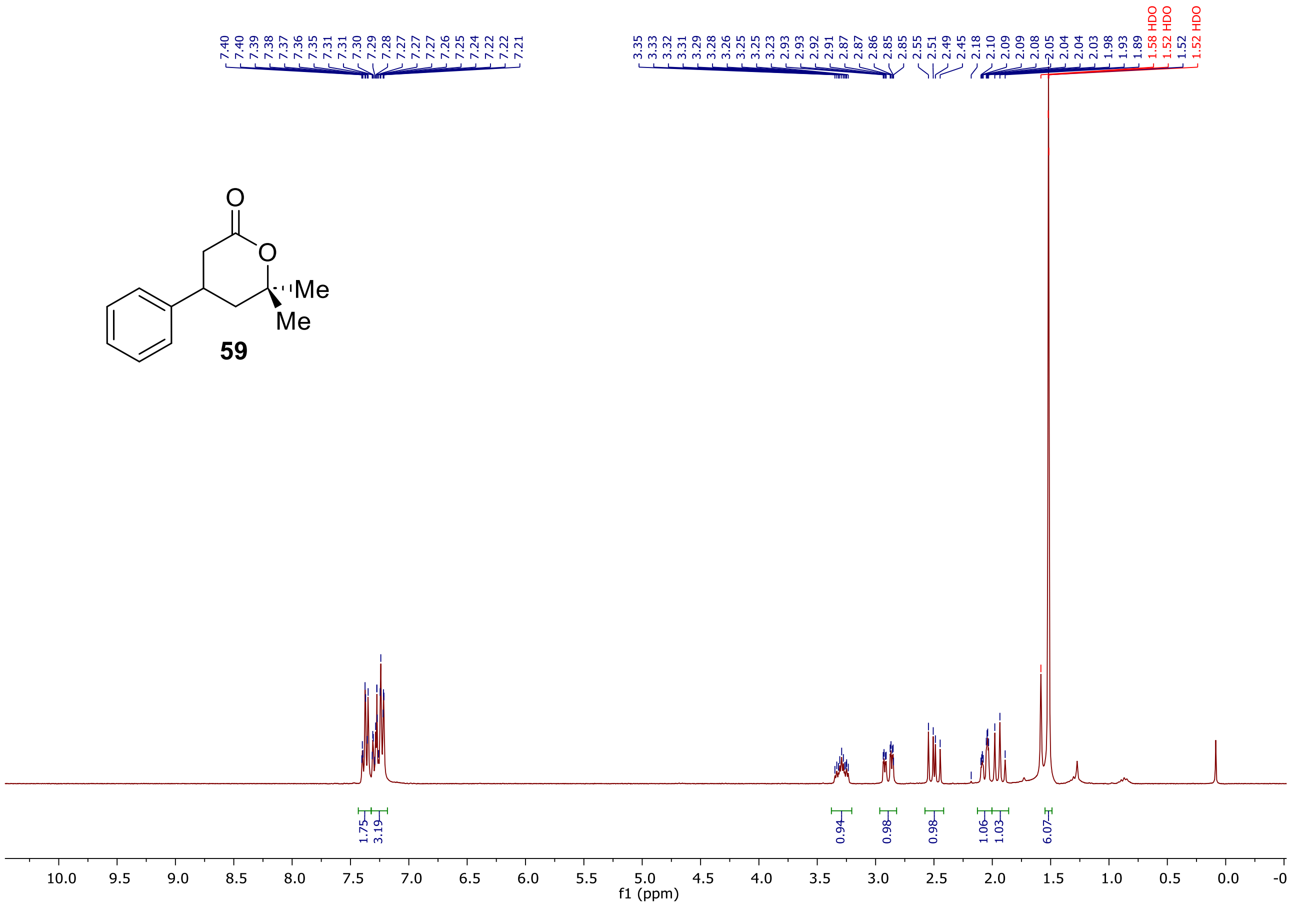
7.40 7.40 7.39 7.38 7.37 7.36 7.35 7.31 7.30 7.29 7.28 7.27 7.27 7.27 7.26 7.25 7.24 7.22 7.22 7.21
3.35 3.33 3.32 3.31 3.29 3.28 3.26 3.25 3.25 3.23 2.93 2.93 2.93 2.92 2.91 2.87 2.86 2.85 2.85 2.55 2.51 2.49 2.45 2.18 2.10 2.09 2.09 2.08 2.05 2.04 2.04 2.03 1.98 1.93 1.89
1.58 HDO
1.52 HDO
1.52
1.52 HDO
O
O
Me
Me
59
1.75 3.19 0.94 0.98 0.98 1.06 1.03 6.07
f1 (ppm)

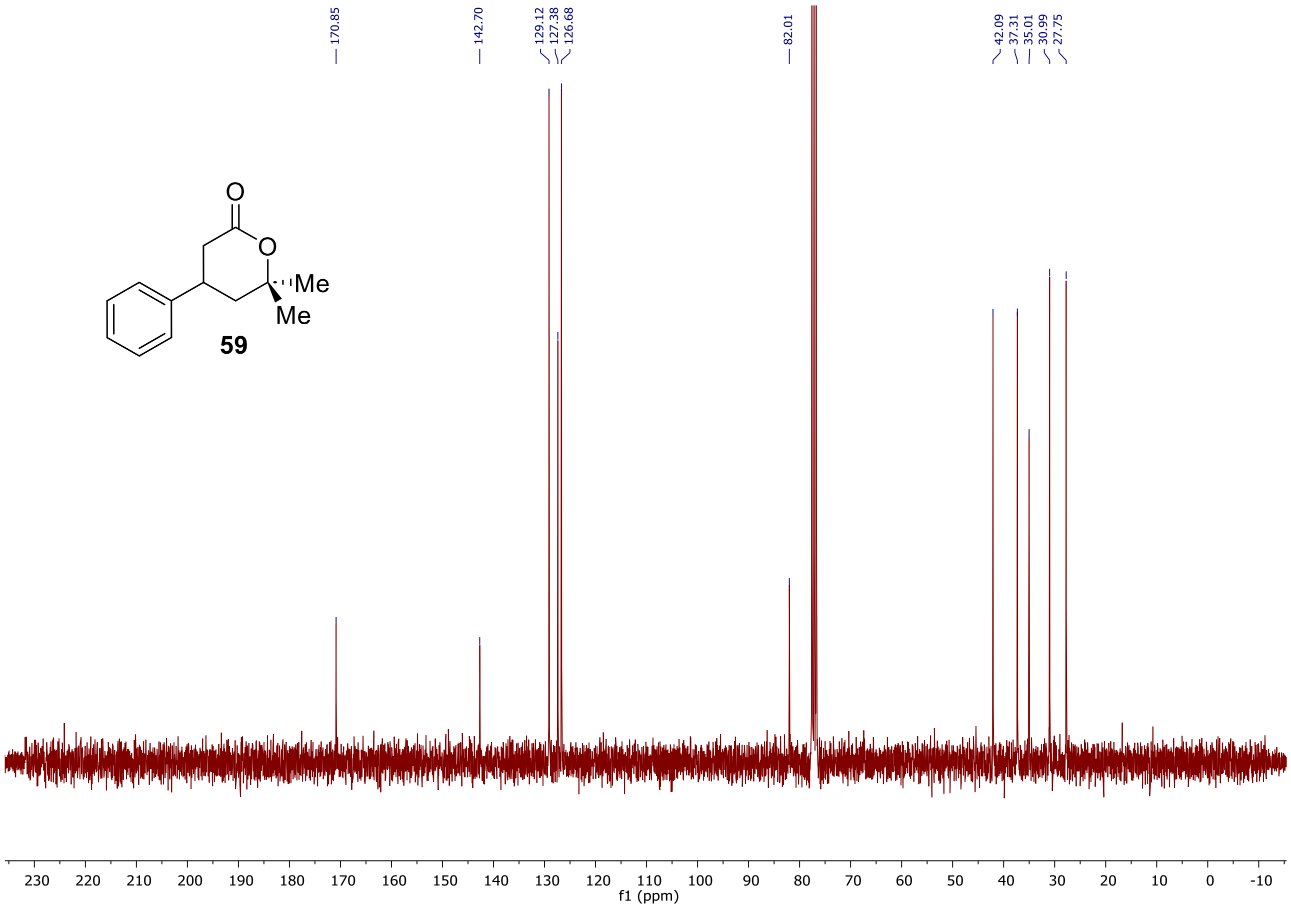
170.85
142.70
129.12 127.38 126.68
82.01
42.09 37.31 35.01 30.99 27.75
O
O
Me
Me
59
f1 (ppm)

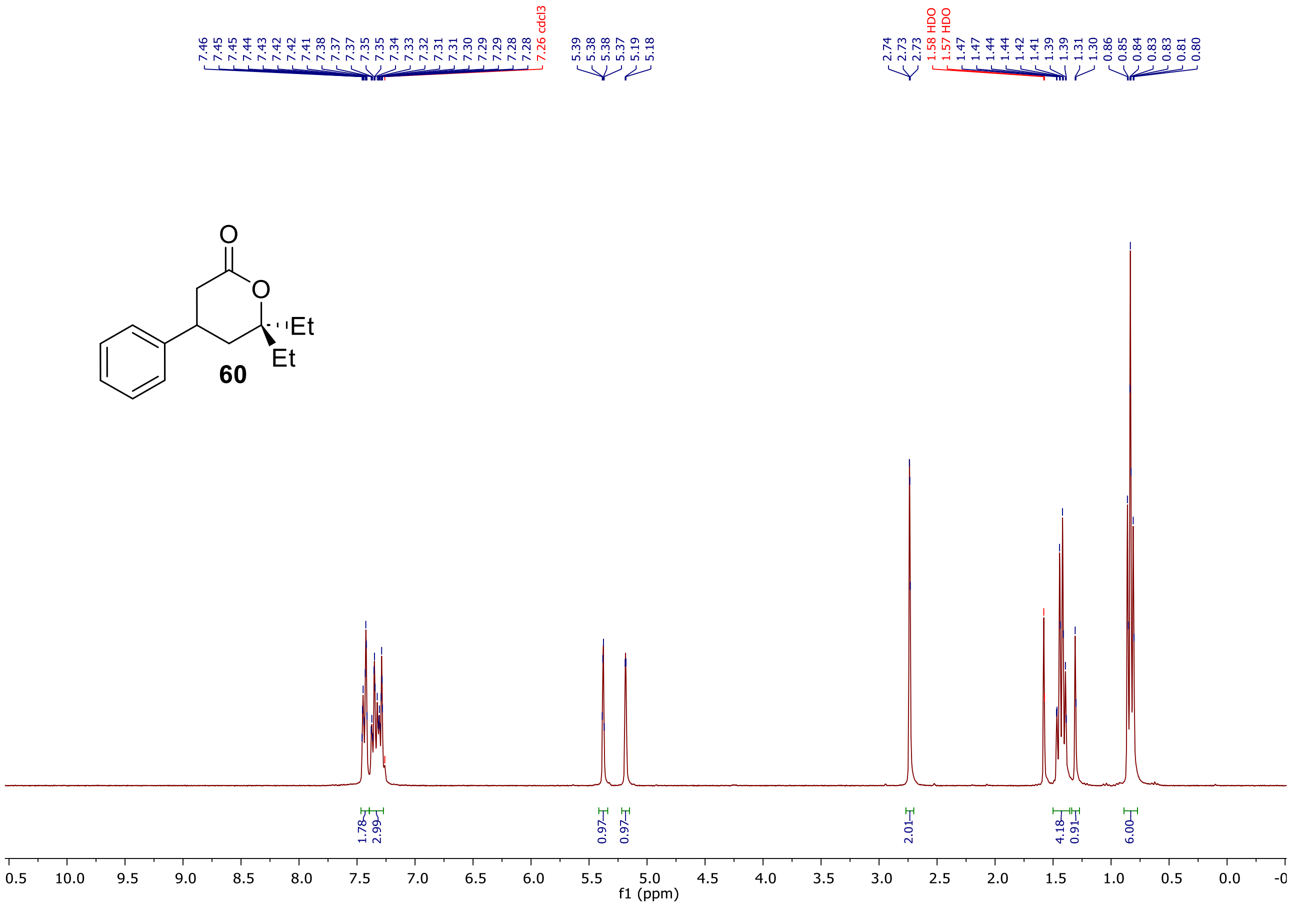
7.46 7.45 7.45 7.44 7.43 7.42 7.42 7.41 7.38 7.37 7.37 7.35 7.35 7.34 7.33 7.32 7.31 7.31 7.30 7.29 7.29 7.28 7.28 7.26 cdcl3
5.39 5.38 5.38 5.37 5.19 5.18
2.74 2.73 2.73 1.58 HDO 1.57 HDO 1.47 1.47 1.44 1.44 1.42 1.41 1.39 1.39 1.31 1.30 0.86 0.85 0.84 0.83 0.83 0.81 0.80
O
O
Et
Et
60
1.78 2.99 0.97 0.97 2.01 4.18 0.91 6.00
f1 (ppm)

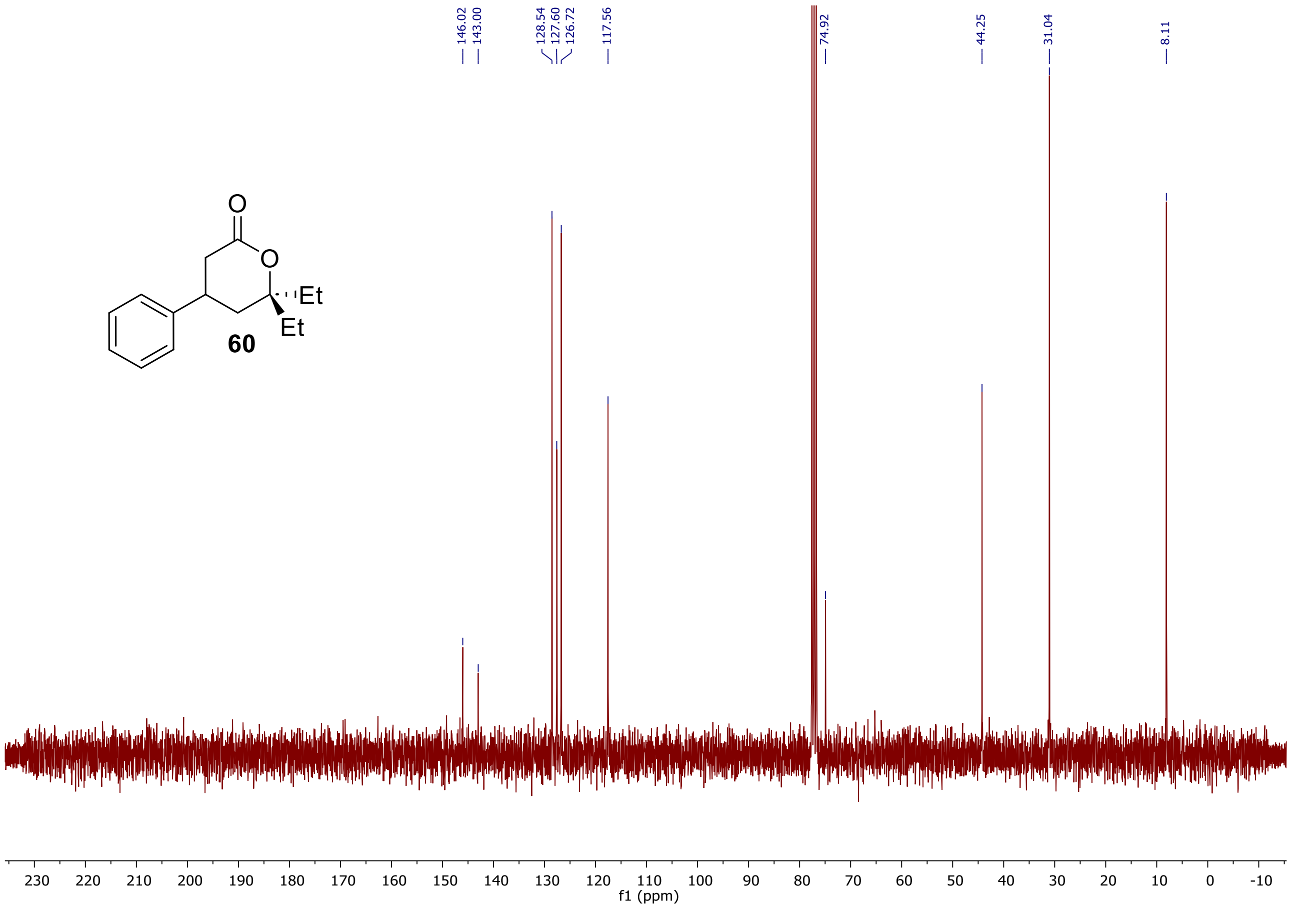
146.02 143.00
128.54 127.60 126.72
117.56
74.92
44.25
31.04
8.11
O
O
Et
Et
60
f1 (ppm)

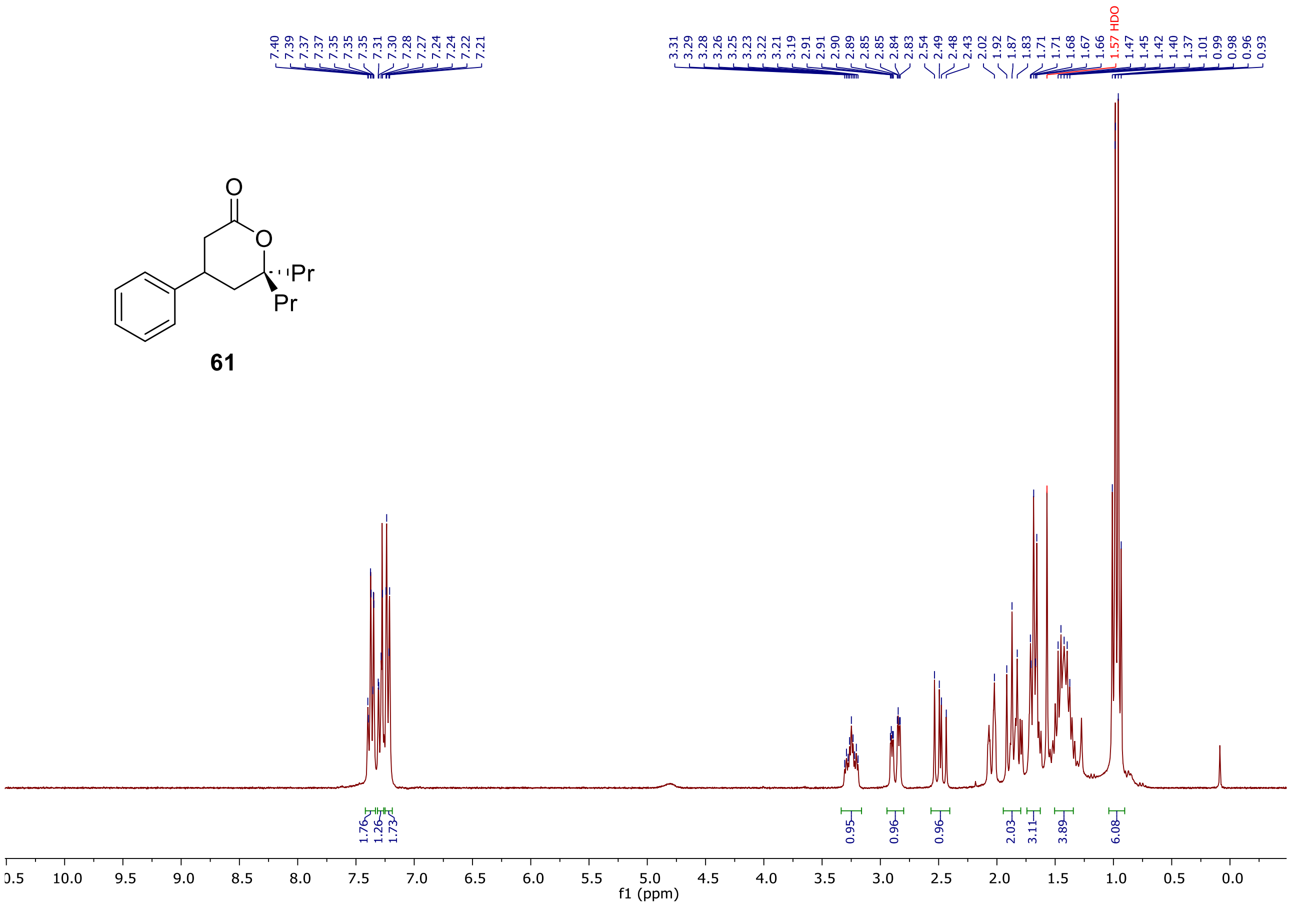
7.40
7.39
7.37
7.37
7.35
7.35
7.35
7.31
7.30
7.28
7.27
7.24
7.24
7.22
7.21
3.31
3.29
3.28
3.26
3.25
3.23
3.22
3.21
3.19
2.91
2.91
2.90
2.89
2.85
2.85
2.84
2.83
2.54
2.49
2.48
2.43
2.02
1.92
1.87
1.83
1.71
1.71
1.68
1.67
1.66
1.57 HDO
1.47
1.45
1.42
1.40
1.37
1.01
0.99
0.98
0.96
0.93
O
O
Pr
Pr
61
1.76
1.26
1.73
0.95
0.96
0.96
2.03
3.11
3.89
6.08
f1 (ppm)

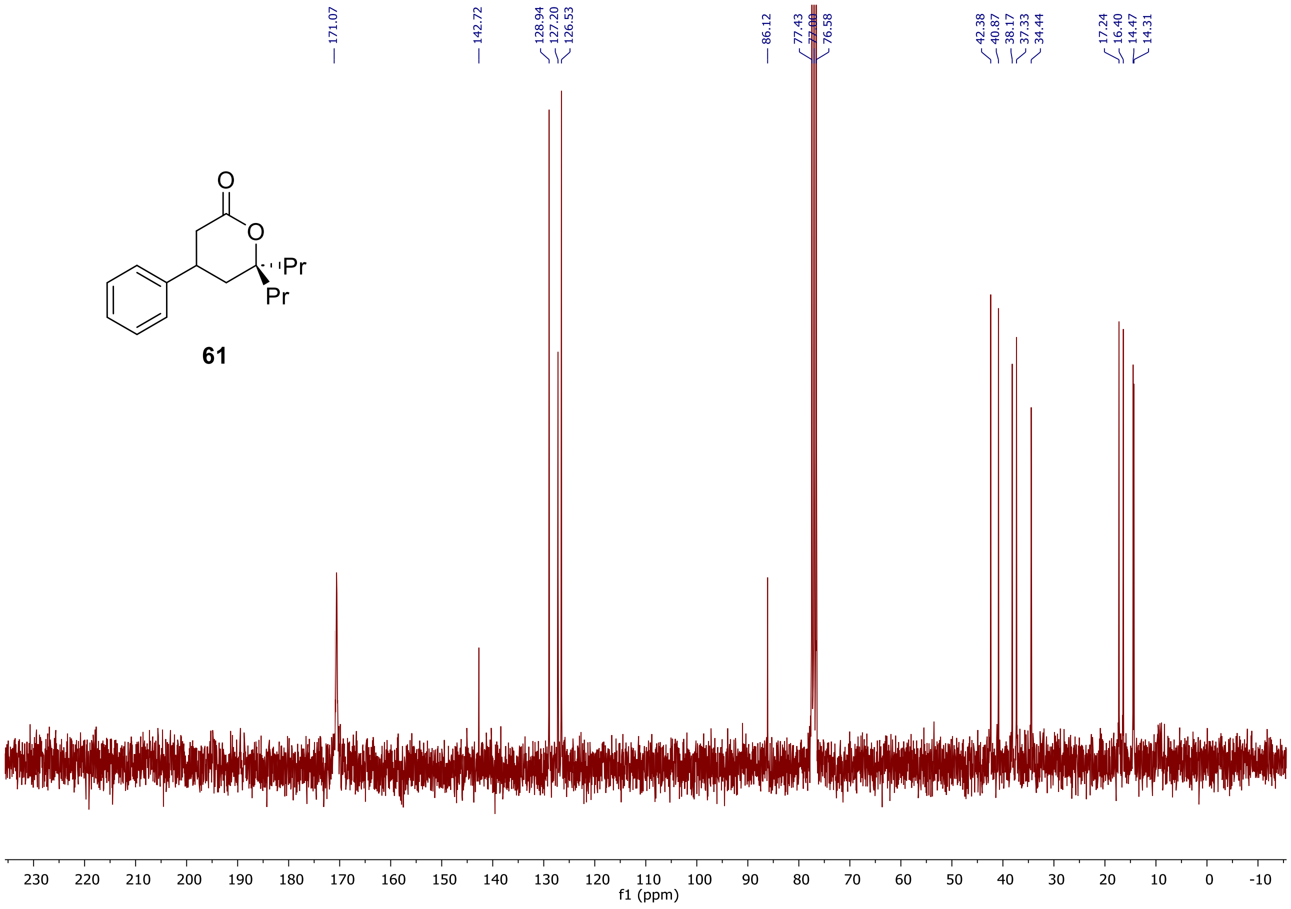
171.07
142.72
128.94
127.20
126.53
86.12
77.43
77.00
76.58
42.38
40.87
38.17
37.33
34.44
17.24
16.40
14.47
14.31
O
O
Pr
Pr
61
f1 (ppm)

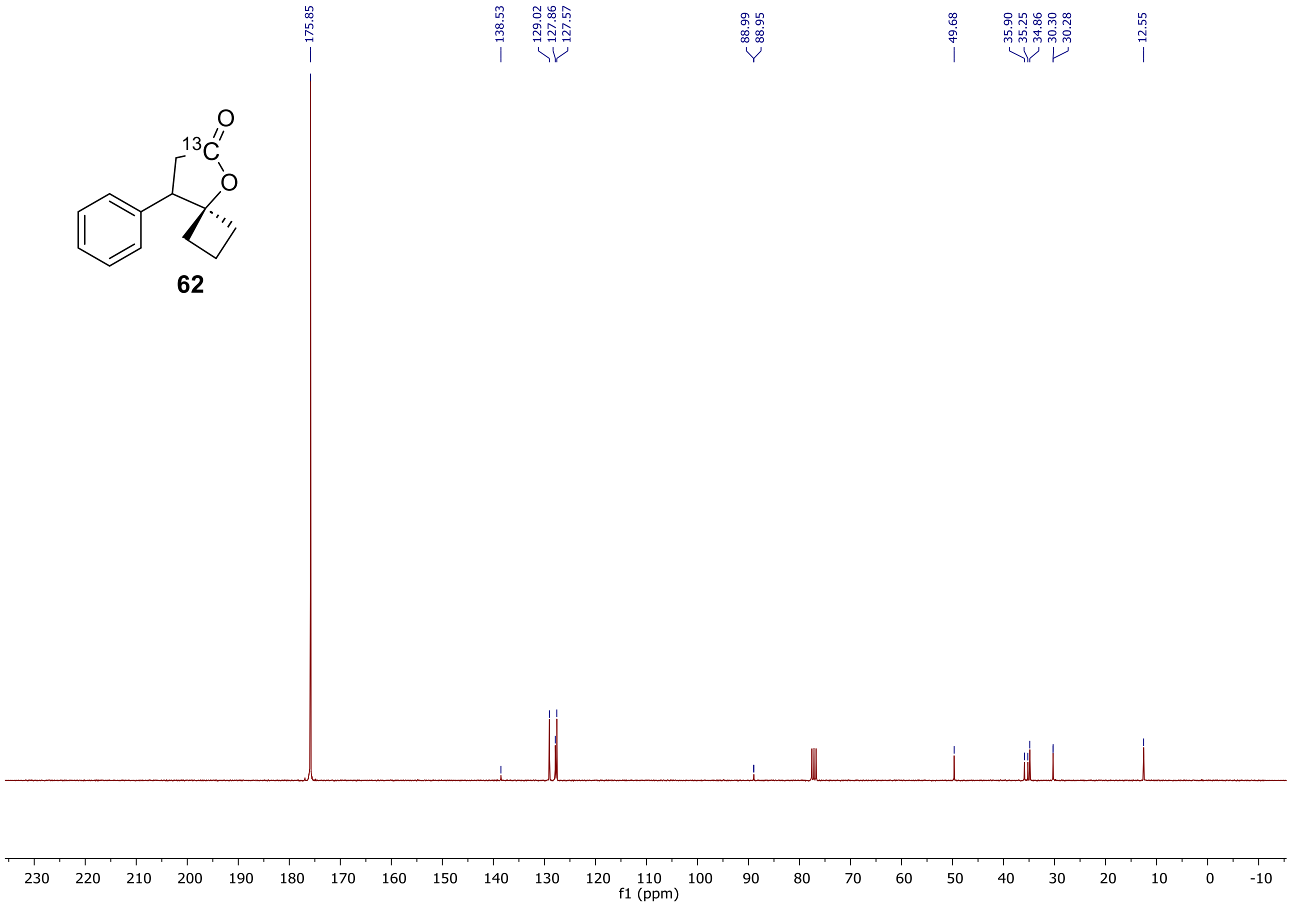
175.85
138.53
129.02
127.86
127.57
88.99
88.95
49.68
35.90
35.25
34.86
30.30
30.28
12.55
62
230 220 210 200 190 180 170 160 150 140 130 120 110 100 90 80 70 60 50 40 30 20 10 0 -10
f1 (ppm)

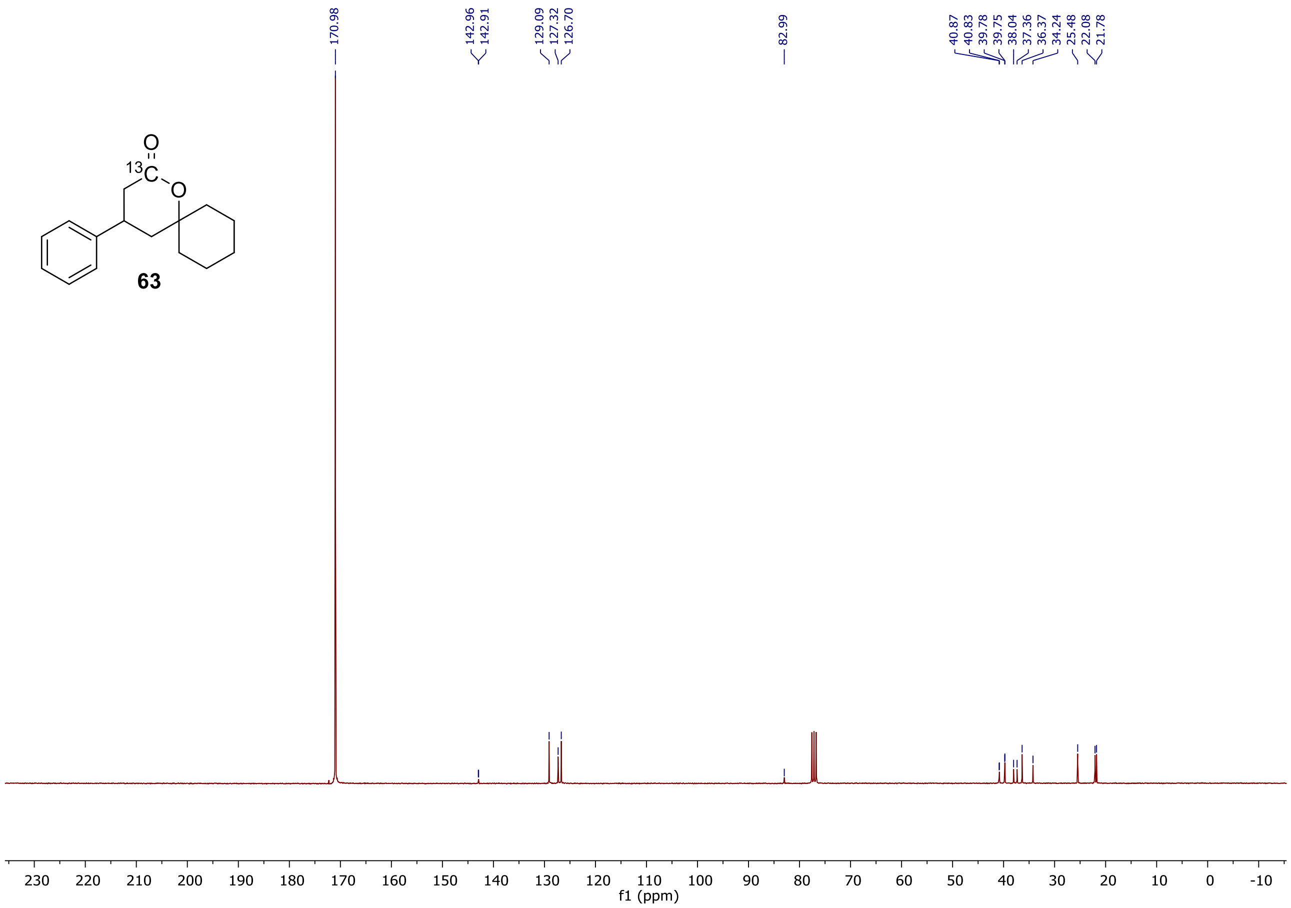
170.98
142.96
142.91
129.09
127.32
126.70
82.99
40.87
40.83
39.78
39.75
38.04
37.36
36.37
34.24
25.48
22.08
21.78
63
230 220 210 200 190 180 170 160 150 140 130 120 110 100 90 80 70 60 50 40 30 20 10 0 -10
f1 (ppm)

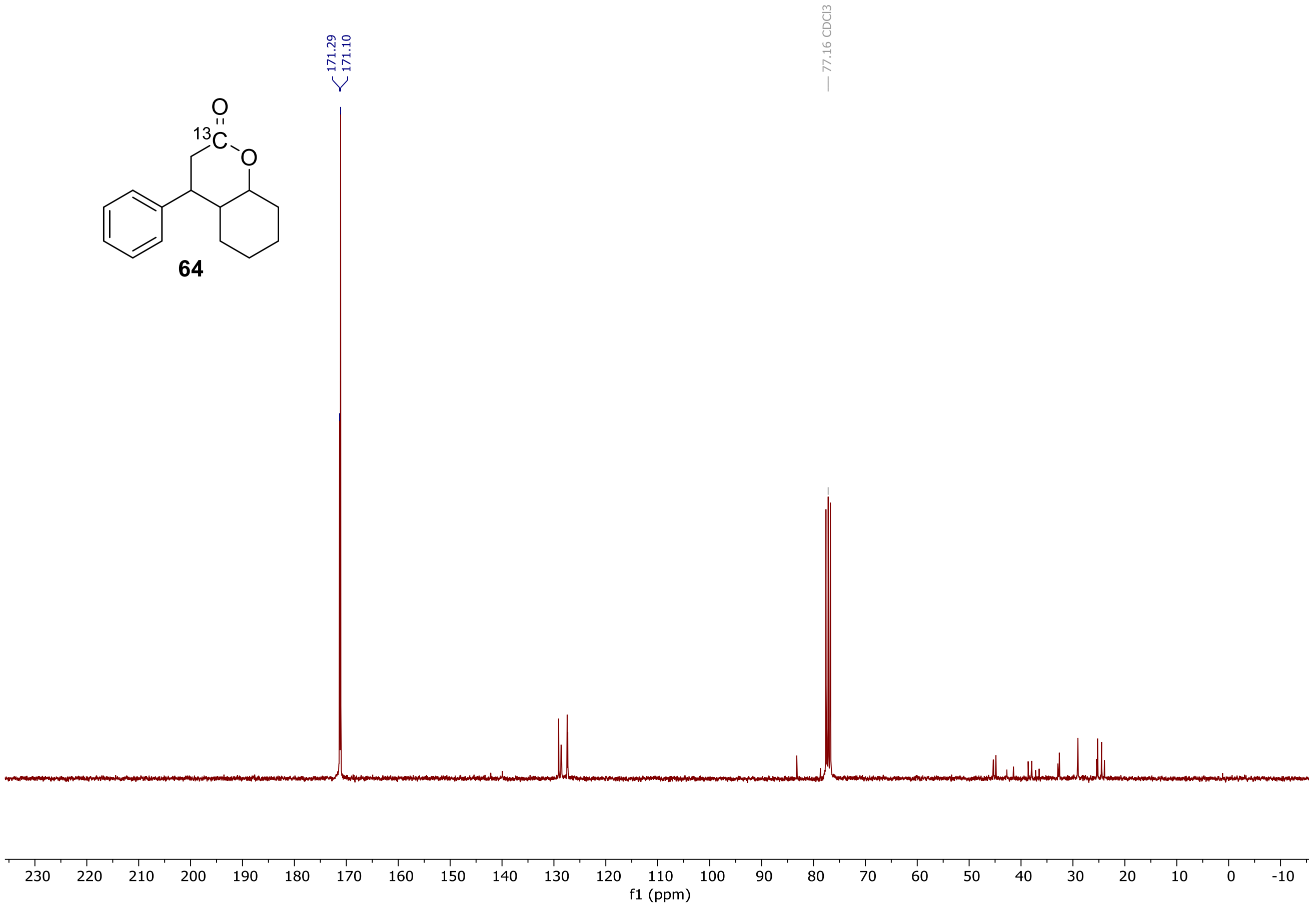
171.29
171.10
77.16 CDCl3
13C
O
64
230 220 210 200 190 180 170 160 150 140 130 120 110 100 90 80 70 60 50 40 30 20 10 0 -10
f1 (ppm)

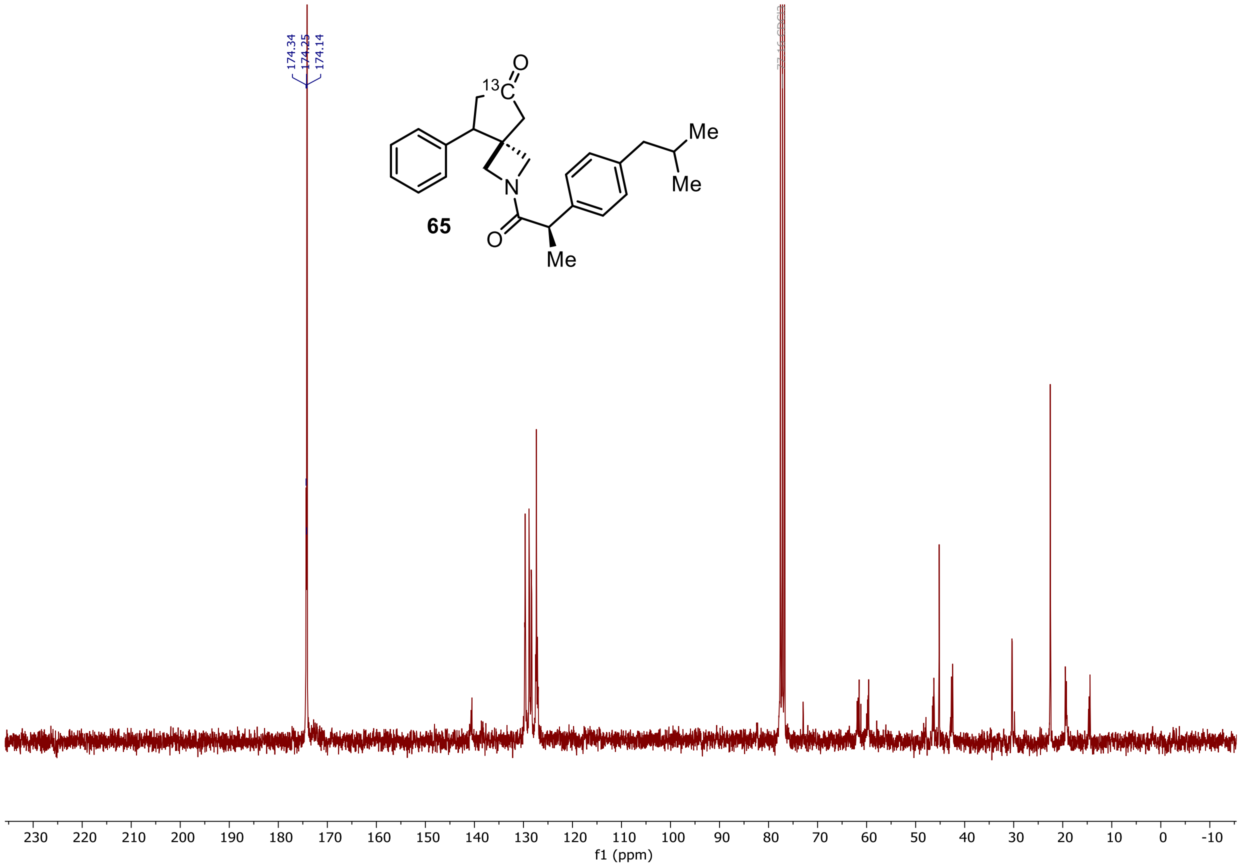
174.34
174.25
174.14
13C
O
N
Me
Me
Me
65
230 220 210 200 190 180 170 160 150 140 130 120 110 100 90 80 70 60 50 40 30 20 10 0 -10
f1 (ppm)

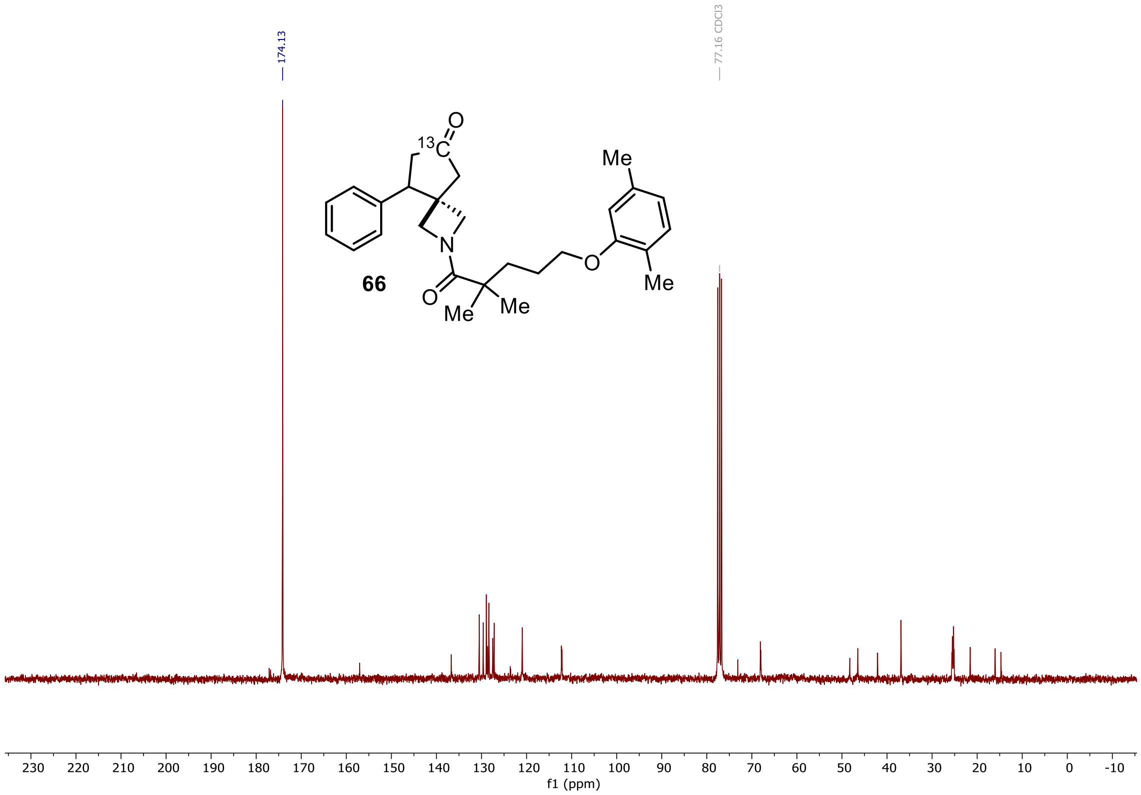
174.13
77.16 CDCl3
13C
O
Me
N
O
Me
Me
Me
66
230
220
210
200
190
180
170
160
150
140
130
120
110
100
90
80
70
60
50
40
30
20
10
0
-10
f1 (ppm)

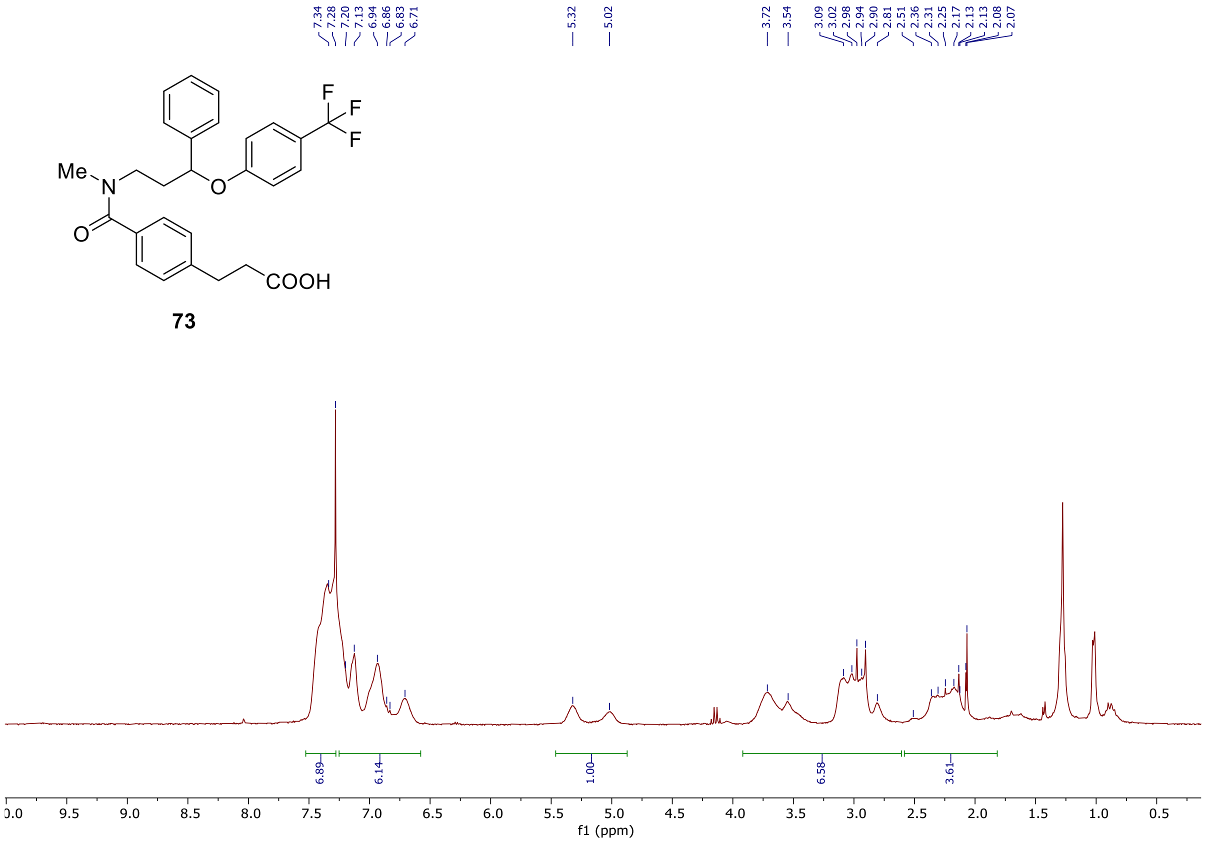
7.34 7.28 7.20 7.13 6.94 6.86 6.83 6.71
5.32 5.02
3.72 3.54 3.09 3.02 2.98 2.94 2.90 2.81 2.51 2.36 2.31 2.25 2.17 2.13 2.13 2.08 2.07
F F F
Me N O
O COOH
73
6.89 6.14 1.00 6.58 3.61
f1 (ppm)

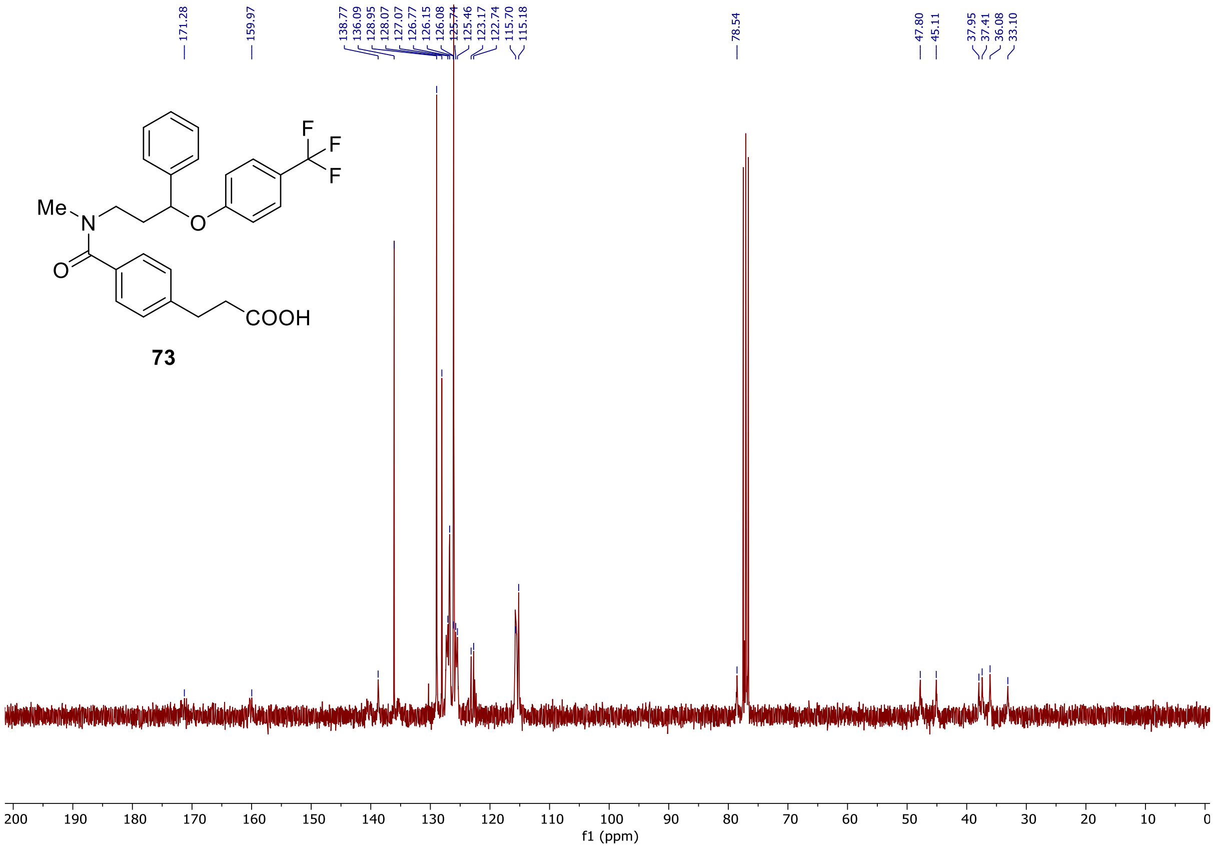
171.28 159.97
138.77 136.09 128.95 128.07 127.07 126.77 126.15 126.08 125.74 125.46 123.17 122.74 115.70 115.18
78.54
47.80 45.11 37.95 37.41 36.08 33.10
F F F
Me N O
O COOH
73
f1 (ppm)

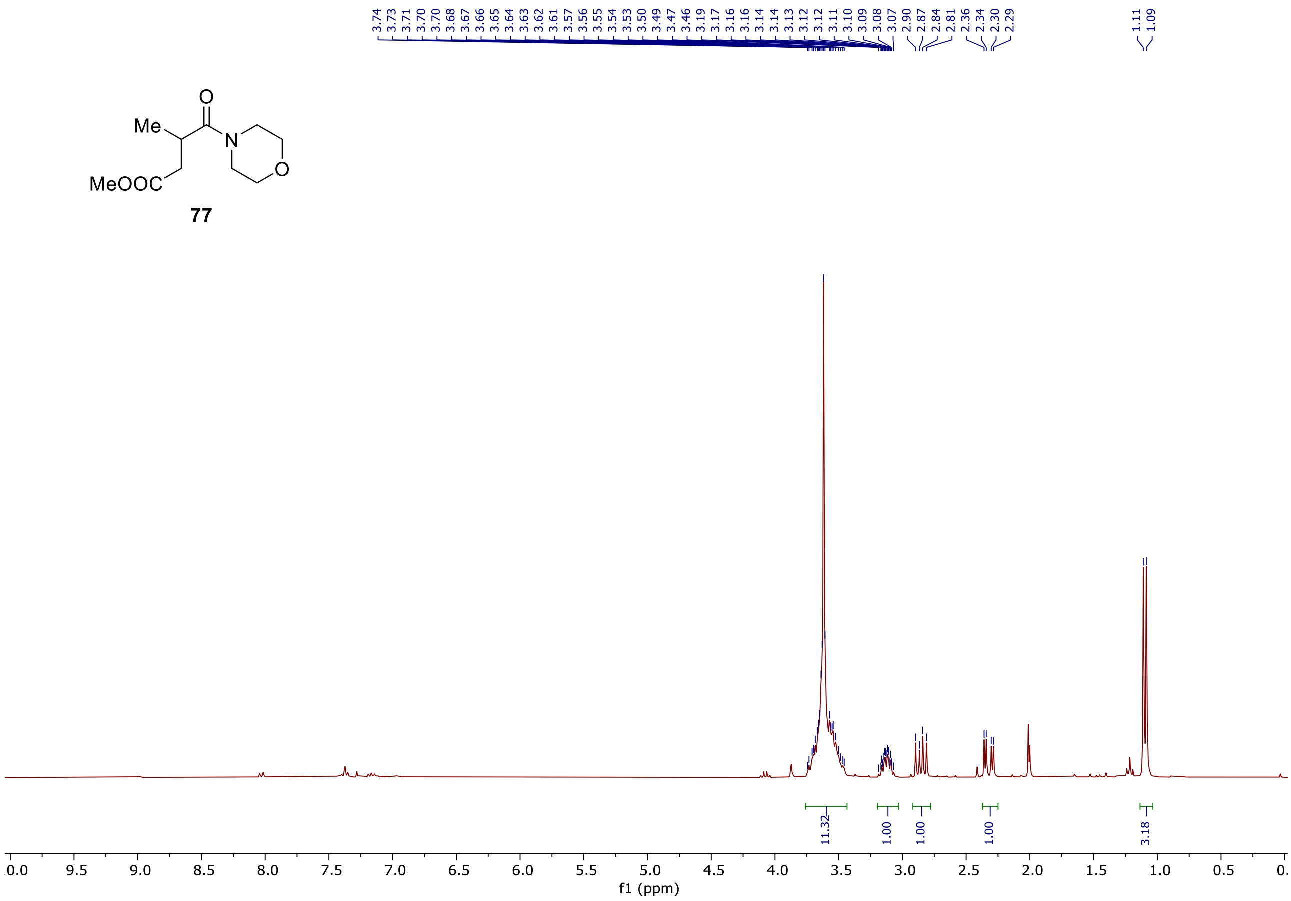

77

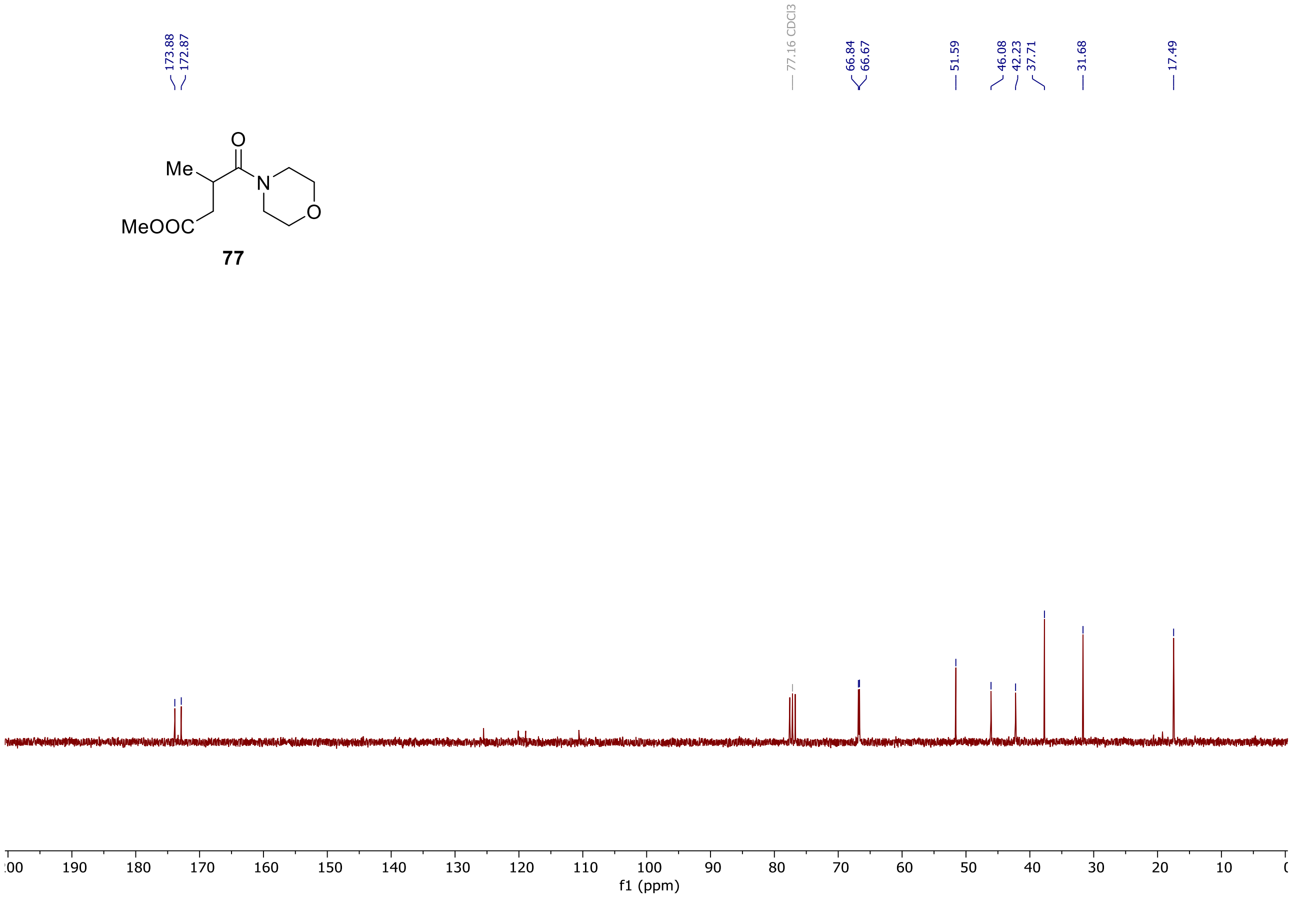

77

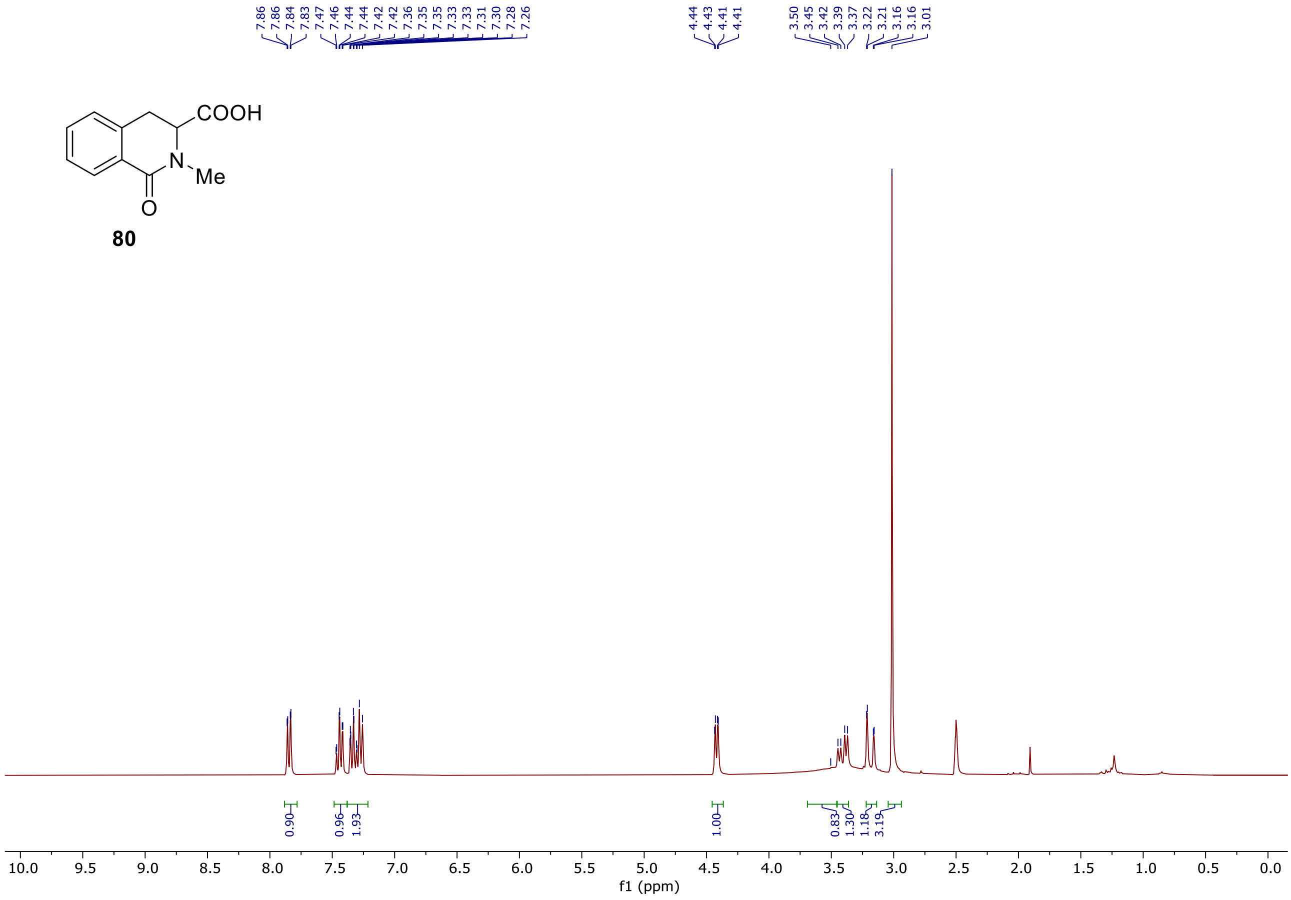
7.86
7.86
7.84
7.83
7.47
7.46
7.44
7.44
7.42
7.42
7.36
7.35
7.35
7.33
7.33
7.31
7.30
7.28
7.26
4.44
4.43
4.41
4.41
3.50
3.45
3.42
3.39
3.37
3.22
3.21
3.16
3.16
3.01
COOH
N
Me
O
80
0.90
0.96
1.93
1.00
0.83
1.30
1.18
3.19
f1 (ppm)

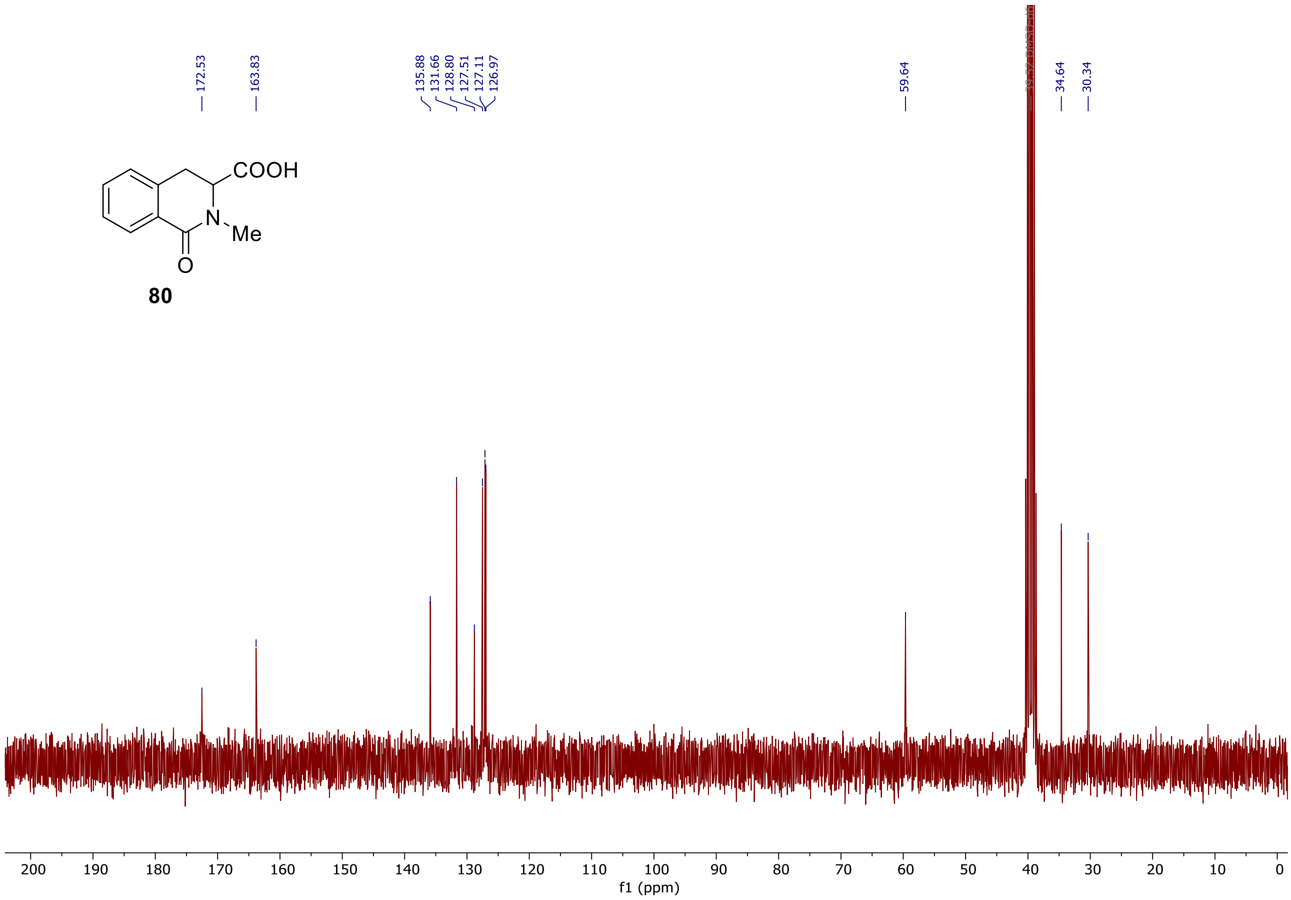
172.53
163.83
135.88
131.66
128.80
127.51
127.11
126.97
59.64
34.64
30.34
COOH
N
Me
O
80
f1 (ppm)
